# Developmental mechanisms of macroevolutionary change in the tetrapod axis: A case study of Sauropterygia

**DOI:** 10.1111/evo.13217

**Published:** 2017-03-21

**Authors:** Laura C. Soul, Roger B. J. Benson

**Affiliations:** ^1^ Department of Paleobiology, National Museum of Natural HistorySmithsonian InstitutionWashingtonDistrict of Columbia20013; ^2^ Department of Earth SciencesUniversity of Oxford,OX1 3ANUnited Kingdom

**Keywords:** Axial body plan, homeotic effects, macroevolution, phylogenetic comparative methods, Sauropterygia, somitogenesis

## Abstract

Understanding how developmental processes change on macroevolutionary timescales to generate body plan disparity is fundamental to the study of vertebrate evolution. Adult morphology of the vertebral column directly reflects the mechanisms that generate vertebral counts (somitogenesis) and their regionalisation (homeotic effects) during embryonic development. Sauropterygians were a group of Mesozoic marine reptiles that exhibited an extremely high disparity of presacral vertebral/somite counts. Using phylogenetic comparative methods, we demonstrate that somitogenesis and homeotic effects evolved in a co‐ordinated way among sauropterygians, contrasting with the wider pattern in tetrapods, in which somitogenetic and homeotic shifts are uncorrelated. Changes in sauropterygian body proportions were primarily enabled by homeotic shifts, with a lesser, but important, contribution from differences in postpatterning growth among somites. High body plan plasticity was present in Triassic sauropterygians and was maintained among their Jurassic and Cretaceous descendants. The extreme disparity in the body plan of plesiosaurian sauropterygians did not result from accelerated rates of evolutionary change in neck length, but instead reflect this ancestral versatility of sauropterygian axial development. Our results highlight variation in modes of axial development among tetrapods, and show that heterogeneous statistical models can uncover novel macroevolutionary patterns for animal body plans and the developmental mechanisms that control them.

Sauropterygians are the longest persisting clade of secondarily aquatic tetrapods, with a time range spanning almost the entire duration of the Mesozoic (>180 million years [myr]; Motani 2009; Kelley and Pyenson 2015). They were among the earliest scientific discoveries of extinct fossil reptiles (Conybeare 1822, 1824), and a long subsequent history of collection and study has led to a rich, global fossil record (Rieppel 2000a; Ketchum and Benson 2010). Sauroptergyians possessed a functionally enigmatic locomotor design (Godfrey 1984; Liu et al. 2015), characterized by the acquisition of a stiff trunk at an early stage of their evolution. Propulsion was provided predominantly by the limbs (Storrs 1993; Liu et al. 2015), which were modified to hydrofoil‐like flippers in plesiosaurian sauropterygians as an adaptation to pelagic life. This unique locomotor plan departs from the tail‐propelled, fish‐shaped body forms seen in other speciose groups of marine tetrapods, including ichthyosaurs, mosasauroids, and cetaceans (e.g., Motani 2005; Lindgren et al. 2007). Nevertheless, some plesiosaurians had short necks and large heads that converge on the gross precaudal body proportions of fish‐like members of other groups (e.g., Romer and Lewis 1959; Hampe 1992).

Based on ecomorphological traits such as tooth morphology and body proportions, it is likely that sauropterygians and other Mesozoic marine tetrapods filled some of the same niches as today's marine mammals, and especially those of some odontocetes (Massare 1987; Collin and Janis 1997). However, sauropterygians were early diverging diapsid reptiles (e.g., Laurin and Reisz 1995; Rieppel 1998; Neenan et al. 2013; Motani et al. 2015), and therefore show numerous structural and biological differences to marine mammals. These differences include the absence of the apparent developmental constraints that limit variation in the vertebral counts of mammals (Narita and Kuratani 2005; Müller et al. 2010; Asher et al. 2011). Extremes of long‐ and short‐necked body proportions evolved in several independent lineages among sauropterygians, and especially among Plesiosauria (Andrews 1913; Bakker 1993; O'Keefe 2002; O'Keefe and Carrano 2005; Benson and Druckenmiller 2014). Placodonts, the shortest necked sauropterygians, had necks comprising as few as six vertebrae and measuring as little as 14% of the trunk length (Rieppel 1995), whereas elasmosaurid plesiosaurians had as many as 76 cervical vertebrae and neck lengths nearly 400% of the trunk length (Kubo et al. 2012). This high level of variation provides a model system for testing macroevolutionary hypotheses relating to vertebral development and body plan evolution in vertebrates, by quantifying patterns of evolutionary change in body proportions and vertebral counts in the context of a well‐constrained phylogeny (Benson and Druckenmiller 2014; Jiang et al. 2014).

## DEVELOPMENTAL AND MACROEVOLUTIONARY MECHANISMS OF AXIAL ORGANIZATION

The ontogenetic processes determining axial organization are largely understood in the context of developmental biology (Iimura et al. 2009; Ten Tusscher 2013). Vertebral counts, the regionalisation of those counts (e.g., into cervical [neck] and dorsal [trunk] portions), and the relative sizes of those regions in adult vertebrates can be inferred from observations of adult osteology (Müller et al. 2010; Ward and Mehta 2010; Böhmer et al. 2015; Head and Polly 2015). Elongation or shortening of the adult axial column has been shown to broadly occur in either one of two regions: precaudal (in sarcopterygians) or caudal (in actinopterygians) (Ward and Mehta 2014). The number of somites (and consequently the number of vertebrae) is controlled by somitogenesis, which acts independently in the two regions (Ward and Brainerd 2007). Among tetrapods, most variation in axial organization occurs through differential elongation of the neck and trunk, which are especially variable among extant lepidosaurs (Kusumi et al. 2013; Ward and Mehta 2014). It is for this reason, in addition to the low prevalence of preservation of the full caudal series in sauropterygians, that we focus on the presacral region in our study. Differences in vertebral formula and the relative lengths of presacral axial regions among taxa result from changes in three key underlying mechanisms: (1) Somitogenesis, which determines the number of presacral vertebrae formed during embryological patterning; (2) Changes in Hox gene expression domains, or homeotic effects, that shift the boundaries between axial regions resulting in different proportions of presacral units patterned as cervical or dorsal vertebrae; and (3) Differential postpatterning growth of somites among axial regions that determines the relative lengths of vertebrae within axial regions.

**Figure 1:**
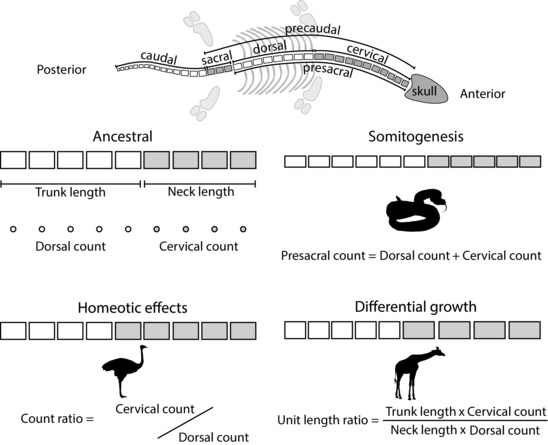
Schematic showing the axial regions referred to within the text and an example of the expected change in relative length and number of vertebrae from a hypothetical ancestral state under three different mechanisms; somitogenesis, homeotic effects, and post‐patterning differential growth.

Somitogenesis is the process by which repeated axial segments are generated in the embryo, by budding from the anterior mesoderm layer. This process is controlled by a molecular oscillator that ‘ticks’, periodically triggering budding of a new somite (Dequéant and Pourquié 2008). If the clock is fast then many relatively small somites will be produced, as opposed to fewer relatively large somites if the clock is slow (Gomez et al. 2008). Homeotic effects relate to the relative timings of Hox gene activation during formation of the presomitic mesoderm, and control the positions of the boundaries between regions (e.g., cervical, dorsal) of the vertebral column, therefore determining the eventual proportion of somites assigned to each region (Iimura et al. 2009). Somitic growth occurs in the postembryonic stage and differential growth of somites among body regions can result in evolutionary change in body proportions in the absence of homeotic change (Parra‐Olea and Wake 2001; Head and Polly 2007). This is the predominant mechanism of body proportion changes among mammals, which have extremely low variance in their counts of cervical and dorsal vertebrae (only sloths and manatees show variation from seven cervical vertebrae; Galis 1999; Narita and Kuratani 2005; Buchholtz and Stepien 2009; Hautier et al. 2010; Varela‐lasheras et al. 2011) but wide variation in proportional neck length (e.g., the elongated neck of *Giraffa camelopardalis*; Fig. 1).

A comparative study of fossil and modern taxa has shown that evolutionary changes in presacral vertebral counts are not correlated with changes in the proportional number of cervical vertebrae in the presacral portion of the axial column in amniotes (Muller et al. 2010). This indicates that changes in somitogenesis and homeotic effects are uncorrelated during evolution. This result is consistent with the observation that somitogenesis and axial regionalization occur at different times during development, are induced through separate regions of the presomitic mesoderm, and therefore are not deterministically linked during ontogeny (Wellik and Capecchi 2003; McIntyre et al. 2007; Wellik 2007; Gomez and Pourquié 2009, but see Dubrulle et al. 2001). However, even in the absence of strict developmental constraints, functional constraints on body plan viability should lead to the co‐ordination of changes in somitogenesis and homeotic effects. Here, we are interested in how these mechanisms might act on macroevolutionary timescales, separately or in unison, to generate adaptive body plan configurations.

Relative rates of change in different morphological traits through time can provide information on the timings of important shifts in those traits, their relationships to one another, and their function (Price et al. 2010; Holzman et al. 2012). We therefore focus on using phylogenetic comparative methods to estimate rates of change in continuous traits (presacral count, neck to trunk length ratio, cervical to dorsal count ratio, and average single cervical to single dorsal length ratio) that can be used as proxies for somitogenesis, homeotic effects, and postpatterning growth of somites. Previous work has highlighted the utility of these continuous traits in indicating how somitogenesis and homeotic effects vary within and between major clades of vertebrates (Müller et al. 2010; Ward and Mehta 2010, 2014; Bergmann and Irschick 2011). Here, we show that comparisons of rates of evolution through time—which make use of phylogeny and heterogeneous statistical models—can be used as explicit quantitative tests of key hypotheses regarding the developmental mechanisms of macroevolutionary change in axial organization and body proportions, shedding light onto how evolution along lineages has given rise to the vast disparity of observed tetrapod axial configurations.

## Methods

### PHENOTYPIC DATA

We assembled a dataset of cervical vertebral counts, dorsal vertebral counts, neck length measurements, and trunk length ( = dorsal series length) measurements (Fig. 1) spanning the Triassic–Cretaceous evolutionary history of sauropterygians (Supplementary Information). These data were acquired through direct specimen observations, plus measurement from photographs in our comparative dataset and the literature using ImageJ (Abràmoff et al. 2004). From a total of 88 taxa for which at least some data were available, most were missing at least one measurement due to incomplete preservation. Cervical vertebral counts were most frequently known, and were observed in a total of 77 taxa that were included in our phylogenetic framework (described below). This resulted in count ratio and presacral count as the most frequently observed input variables of those that we used in our analyses, in a total of 63 taxa.

Cervicals were defined as those vertebrae functionally belonging to the neck, determined preferentially by the position of the pectoral girdle in articulated skeletons, or by the rib morphology (cervical vertebrae have short ribs with expanded distal ends; dorsal vertebrae have long, curving ribs; the atlas‐axis complex was counted as two vertebrae). When the pectoral girdle was not present or had moved from life position, and where the ribs were also disarticulated, the first dorsal vertebra was counted as the first element in which the rib formed a contact with both the centrum and the neural arch (i.e., the first pectoral vertebra; Seeley 1874; Welles 1943). The dorsal series was determined to end immediately anterior to the sacrum. Sacral vertebrae have rib facets shared between the centrum and neural arch, and connect to short, robust ribs that articulate with, or would have articulated with, the pelvic girdle. Where some uncertainty existed in counts of cervical or dorsal vertebrae, for example when the cervical/dorsal transition could not be unambiguously determined due to damage, we randomly selected a count from the range of possible counts. We conducted this randomization 100 times for each ambiguous measurement to generate 100 datasets encompassing uncertainty in precise vertebral counts.

Our protocol resulted in some counts that differ from those previously reported in the literature. For example, Sato et al. (2010) identified 50–51 cervical vertebrae in the Triassic pistosaurian *Yunguisaurus*. However, the 45th and more posterior presacral vertebrae are located posterior to the pectoral girdle and have long, curving ribs that enclose the trunk (Fig. 8 in Sato et al. 2013). Therefore, we counted only 44 cervical vertebrae in *Yunguisaurus*. The cervical vertebral counts used by O'Keefe (2002) include both the atlas and axis as one unit, whereas we counted these separately.

We used our measurements to generate four input variables for further analysis (Fig. 1). (1) Length ratio–the ratio of neck length to trunk length, used as a measure of body proportions. We view this as quantifying relative neck length, normalized for trunk length, which can be used a measure of body size in sauropterygians (Benson et al. 2012). (2) Count ratio–the ratio of the cervical vertebral count to dorsal vertebral count, used as a measure of homeotic domain sizes during embryological patterning. Our approach differs slightly from that of Müller et al. (2010), who used the proportion of cervical vertebrae included in the presacal vertebral series (i.e., cervical count/(cervical count + dorsal count)), rather than the ratio (i.e., cervical count/dorsal count); the proportion introduces statistical artefacts at values close to 0 and 1. (3) Unit length ratio–the ratio of the average length of a cervical vertebra to the average length of a dorsal vertebra, used as a measure of the differential postpatterning growth of axial regions. (4) Presacral count (Müller et al. 2010)–the total number of cervical and dorsal vertebrae, used as a measure of the number of somites generated during embryological patterning.

Increases and decreases in the values of measured traits are asymmetrical on a proportional scale. In other words, the absolute change required to double a value is greater than that required to halve a value. For some traits (e.g., body size; Brown (1995)), a proportional scale is more appropriate than an absolute scale because the variance associated with larger values is greater than that for smaller values. A proportional scale is achieved by log‐transformation of trait values. However, it was not clear a *priori* whether this was most appropriate for the traits we analysed. To determine this we asked whether the magnitude of evolutionary changes (represented by the absolute values of standardized phylogenetically independent contrasts) correlated with the estimated trait values at nodes in the phylogeny (i.e., whether large values of traits have higher variance than small values do). We found no significant correlation for any of the traits (Supplementary Information). A second problem exists regarding ratio data specifically, for which increases and decreases in the value of the denominator have asymmetrical effects on the absolute value of the ratio. However, we observed that trunk length and dorsal vertebral count (the denominators of our ratio traits) both have relatively low variance compared to neck length and cervical vertebral count (their numerators), partly addressing this problem. Ratios are ubiquitous in studies of phenotypic evolution, not least because the absolute sizes of structures and modules of organisms are often less informative than their relative values when compared to body size. In our case the evolutionary changes we were interested in could not be accessed without ratios, and log‐transformation was not demonstrably appropriate for those traits. We cannot propose a full solution to this problem here but present analyses of untransformed trait values in the main manuscript, and additionally report results of analyses with the variables log‐transformed in the Appendix and in Fig. S10 for comparison.

### PHYLOGENY

Knowledge of phylogenetic relationships within Sauropterygia has advanced rapidly in recent years (Rieppel 2000a; O'Keefe 2001; Druckenmiller and Russell 2008; Ketchum and Benson 2010; Neenan et al. 2013; Benson and Druckenmiller 2014; Jiang et al. 2014). Among Triassic taxa, these advances have demonstrated that placodonts form a clade with other sauropterygians (Rieppel 2000b). Within Plesiosauria they have revealed that previous hypotheses of geologically long‐lived clades of long‐necked “plesiosauroids” and short‐necked “pliosauroids” in fact mask the repeated convergent evolution of end‐member morphotypes throughout plesiosaurian evolution (Bakker 1993; Carpenter 1996; O'Keefe 2002; see also White 1940).

The phylogenetic framework used in the present study combines information from the datasets of Jiang et al. (2014; Triassic sauropterygians) and Benson and Druckenmiller (2014; Jurassic–Cretaceous plesiosaurians). Our topology for Plesiosauria was based on a more inclusive matrix using the character list of Benson and Druckenmiller (2014), but including more taxa. Scores for the additional taxa included in this matrix were presented by Otero (2016) [Polycotylidae] and Serratos et al. (in press) [Elasmosauridae]. Tree searches were performed in PAUP* 4.0b10 for Macintosh (Swofford 2002). Initial exploration for shortest length tree islands was conducted using four independent randomizations of the Parsimony Ratchet implemented by PAUPRat (Nixon 1999; Sikes and Lewis 2001). The resulting subset of most parsimonious trees (MPTs) was then used as the starting point for TBR (tree bisection and reconnection) branch swapping. We selected 100 of these most parsimonious cladograms at random from the full set of MPTs and combined them with the most recent comprehensive hypotheses of nonplesiosaurian relationships (Jiang et al. 2014) to generate a set of 100 composite trees, including 139 taxa and spanning the entire evolutionary history of Sauropterygia (Supplementary Information). Adequate trait data were not available for all the taxa included in the phylogeny, we therefore downsampled the number of tips in the tree before analysis, and this procedure removed areas of local phylogenetic uncertainty that are only relevant when a larger sample of taxa is considered.

We accounted for the influence of topological uncertainty on our results by completing all the analyses described below on this set of 100 trees. Trees were scaled to geological time using an algorithm based on the Hedman (2010) method of estimating probable node ages based on the age of consecutive outgroups (Lloyd et al. 2016). Uncertainty in precise taxon ages was accommodated by selecting different occurrence dates for each topology from a random uniform distribution across the narrowest interval from which each taxon was known. A representative topology is shown in Figure S1 and the full set of trees and range data are included in the Supplementary Information. The Hedman (2010) approach resulted in a divergence time for the most recent common ancestor of Plesiosauria that ranged in age from 238.9–234.9 Ma. However, definite plesiosaurians are only known from the earliest Jurassic and younger deposits (Benson et al. 2012) and may not have originated until late in the Triassic. Therefore, we generated a set of time‐scaled trees in which the basal node of Plesiosauria was constrained to appear no earlier than the start of the Rhaetian (208.5 Ma), and Triassic branches within Plesiosauria were compressed isotropically to accommodate this. Analyses were performed on both these constrained trees and unconstrained trees. Results figures for the constrained trees are presented in the main manuscript, corresponding figures for the unconstrained trees are included in the Supplementary Material.

### ANALYSES

To quantify rates of evolutionary change in the number of somites, homeotic effects and body proportions, and to ask about the relationships among these processes, we used two phylogenetic comparative methods that use the evolutionary changes inferred across a phylogeny in a set of univariate traits observed at the tips of the phylogeny (Fig. 1). All analyses were performed in R version 3.3.0 (R Core Team 2015).

The first method quantifies evolutionary rate variation in each univariate trait individually. This is achieved using a Bayesian Monte Carlo Markov chain approach to fit a variable‐rate Brownian motion model, in which the Brownian variance parameter (σ^2^) is an estimate of the rate of evolution (Hansen 1997; Hunt 2012). This method, AUTEUR (Accommodating Uncertainty in Trait Evolution Using R), was originally presented by Eastman et al. (2011) and is implemented in the R package geiger version 2.0.6 (Pennell et al. 2014). A key strength of this approach is that variation in the rate of phenotypic evolution can be estimated without the user having to specify the positions of rate changes on the tree in advance. We assessed mixing and convergence using coda version 0.18–1 in R (Plummer et al. 2006). For each analysis (i.e., for each univariate trait on each tree) we combined the results from two independent Markov chains that ran for 5 million generations, and discarded the first quarter as burn‐in. Effective sample sizes were all greater than 1000.

A second method was used to test for correlation between the patterns of evolutionary change seen among individual univariate traits, using standardised phylogenetic independent contrasts (PICs; Felsenstein 1985). Standardized PICs (in which the contrasts are divided by their expected standard deviations) of our variables were computed using the package ape (Paradis et al. 2004). Each contrast corresponds to a node of the tree, and can be considered as a point estimate of the evolutionary rate at that node (Freckleton and Harvey 2006), with a sign that represents the direction of the shift in trait value estimated to have occurred at that node (Felsenstein 1985). Statistical analyses of PIC‐transformed variables represent analysis of data corrected for phylogenetic autocorrelation, and can be used to investigate the relationships between changes in phenotypic trait values on the branches of a phylogeny using regression tests. We used ordinary least squares regression (OLS) of PIC‐transformed variables for two purposes. (1) To quantify the relative contributions of homeotic effects and differential postpatterning growth to evolutionary change in adult body proportions by examining the relationships between (i) length ratio and count ratio and (ii) length ratio and unit length ratio. (2) To ask whether homeotic shifts were correlated with evolutionary changes in somitogenesis by examining the relationship between count ratio and presacral count (Fig. 1).

## Results

### RATES OF TRAIT EVOLUTION

**Figure 2:**
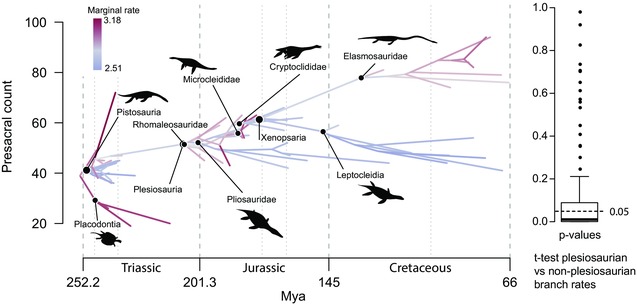
Rates of evolutionary change in presacral count (somitogenesis). Phenogram showing the rates of presacral count evolution through time mapped onto a trait space for an example topology and boxplot of the *P* values for a *t*‐test for a significant difference between plesiosaurian and nonplesiosaurian posterior rates, across all 100 topologies. Red branches correspond to faster evolutionary rates, blue to slower, and gray to those that do not deviate from the median. Silhouettes representing an example body plan for each subclade are shown.

Rates of presacral count evolution vary considerably across the phylogeny (Fig. 2, Fig. S2). Within 20 million years of divergence from their most recent common ancestor, presacral counts of Triassic sauropterygians occupied a wider range than those attained across most groups of Jurassic–Cretaceous plesiosaurians. This is evident from the occurrence of low presacral counts (19–26) among placodonts and high counts (41–72) among Triassic pistosaurians, which together span an almost fourfold range (Fig. 2). Among plesiosaurians, presacral counts span an approximately twofold range, and only the Cretaceous elasmosaurids achieved presacral counts outside the range of Triassic sauropterygians, with high values from 74 to 94. Rates of presacral count evolution are a proxy for evolutionary change in the number of somites generated during embryonic development. For trees in which the basal node of Plesiosauria was constrained to the Rhaetian, per branch median posterior rates of presacral count evolution from 100 trees varied between σ^2^ = 1.33–4.67 vertebrae/Ma. Relatively high rates occurred among deep branches within both Sauropterygia and Plesiosauria (Fig. S1: especially Plesiosauroidea), with slow‐downs in several subclades (Pachypleurosauridae, Pliosauridae, Cryptoclididae, and Leptocleidia). Higher rates were maintained in the extremely short‐necked Placodontia, early‐diverging Rhomaleosauridae, and the extremely long‐necked Microcleididae and Elasmosauridae. There was little consistent support across trees for any rate shifts at particular nodes, apart from the node ancestral to *Yunguisaurus*, which has an unusually high presacral count compared to other pistosaurs (Cheng et al. 2006; Sato et al. 2010). Nevertheless, a multiple‐rate Brownian motion model is strongly supported for this trait, and it is possible to perform statistical comparisons of the rates on branches within subclades. Median posterior rates on Triassic (nonplesiosaurian) branches were significantly higher than those of Jurassic‐Cretaceous (plesiosaurian) branches on 69% of trees. However, a separate, more conservative, permutation test of the full posterior distributions (cf Eastman et al. 2011) showed that they overlap, and did not support different rates between the two groups for any tree (Fig. 2, Fig. S7).

When analyses were performed on the set of 100 trees in which the root node for Plesiosauria was unconstrained, per branch median posterior rates of presacral count evolution varied from σ^2^ = 1.44–4.33 vertebrae/Ma. Median posterior rates were significantly higher in nonplesiosaurian than plesiosaurian taxa for 68% of trees, approximately the same proportion as for the constrained trees (Fig. S2). Overall, patterns of rate variation were largely similar to those obtained using the constrained trees, with the exception that elevated rates were not inferred for the internal branches around the origin of Plesiosauria (Fig. S2).

Notably, within Plesiosauria, low rates are seen among short‐necked taxa (and among deep branches when Plesiosauria is not constrained to originate in the Rhaetian; Fig. S2). The low rates of presacral count evolution seen in the typically short‐necked plesiosaurian groups Pliosauridae and Leptocleidia are not a statistical artefact of low trait values: these groups have higher presacral counts than many Triassic sauropterygians, particularly placodonts, which have higher rates of evolution. In general longer necked plesiosaurians show higher rates of evolutionary change in presacral counts compared to contemporaneous short‐necked plesiosaurians. The same pattern of higher than average median rates in longer necked clades does not apply for nonplesiosaurian taxa.

**Figure 3:**
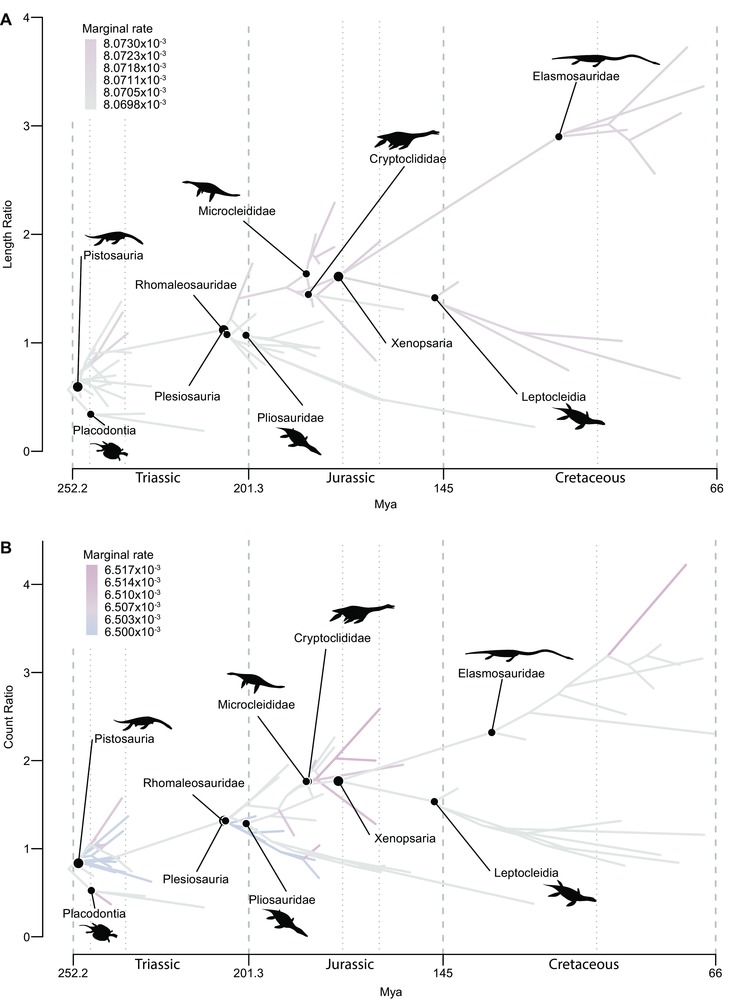
Rates of evolutionary change in body proportions and cervical/dorsal vertebral counts (homeotic shifts). Phenogram showing the rates of (A) length ratio and (B) count ratio evolution through time mapped onto their trait spaces for an example topology. Most branch rates do not differ substantially from the median rate (grey), particularly in A. Those that do are red where they are slightly faster than the median and blue where they are slightly slower than the median. Silhouettes representing an example body plan for each subclade are shown.

Despite their high disparity in presacral vertebral counts, Triassic sauropterygians achieved a relatively narrow range of body proportions (length ratio) and of the ratio of cervical vertebral counts to dorsal vertebral counts (count ratio) compared to plesiosaurians (Figs. 3, S3). In particular, Plesiosauria includes “plesiosauromorph” taxa within Microcleididae, Cryptoclididae, and Elasmosauridae that have proportionally longer necks and higher cervical counts than any Triassic sauropterygian (Fig. 3). Rates of length ratio evolution represent the rates of change of body proportions, whereas rates of count ratio evolution are a proxy for evolutionary change in homeotic controls on regionalization of the axial column. Variation in estimated rates of evolution of these two traits among sauropterygians was considerably less pronounced than for presacral count. Small variations in branch rates across the trees were supported, under the relaxed‐clock model that we implemented (Fig. 3). However, these changes did not lead to any significant differences in comparisons of the evolutionary rates between subclades that were consistent across the set of 100 trees, and a multiple‐rate Brownian motion model was not statistically supported. This contrasts with the pattern seen in total presacral vertebral counts, which show higher median rates among nonplesiosaurian (Triassic) taxa more frequently (Figs. 2, S2).

### PHYLOGENETIC INDEPENDENT CONTRASTS

**Figure 4:**
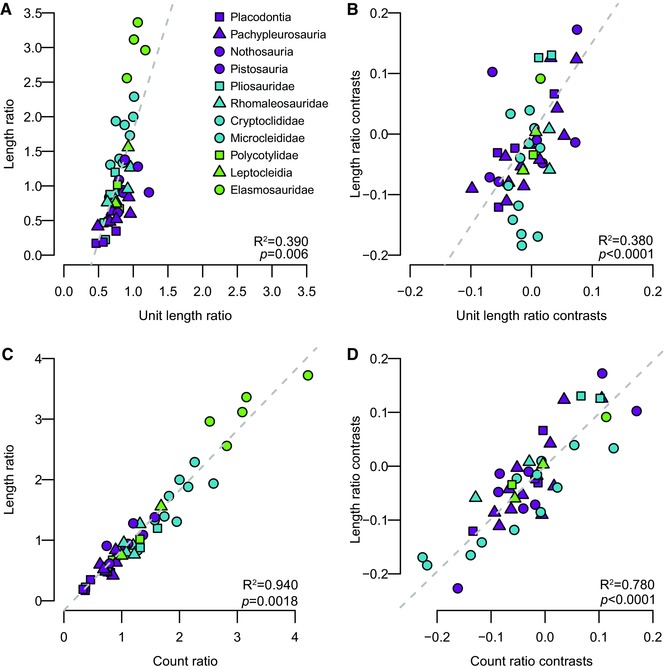
Relative contributions of differential postpatterning growth (A and B: unit length ratio) and homeotic shifts (C and D: count ratio) to evolutionary changes in body proportions (length ratio). Scatterplots and best fit lines for an example topology for ordinary least squares regression of (A) raw length ratio against unit length ratio; (B) standardized PICs of length ratio against standardized PICs of unit length ratio; (C) raw length ratio against count ratio; (D) standardised PICs of length ratio against standardized PICs of count ratio. All scales are equal on *x* and *y* axes to highlight the difference in slope between regressions.

Regressions of the standardized phylogenetic independent contrasts (PICs) of count ratio and unit length ratio against those of length ratio indicate the relative contributions of homeotic shifts and differential postpatterning growth of somites to evolutionary change in body proportions. All coefficients of determination were positive and strongly statistically significant (*P* < 0.0001; Fig. 4). When the origin of Plesiosauria is constrained to occur in the Rhaetian, PICs of unit length ratio explain a median of 37.7% of the variation in PICs of length ratio (0.311 < *R* ^2^ < 0.430 [across 100 phylogenies]; Fig. 4B), whereas PICs of count ratio explain a median of 75.8% of that variation (0.691 < *R* ^2^ < 0.818; Fig. 4D). The results of (nonphylogenetic) regressions of the observed values of length ratio against those of count ratio show that count ratio explains 94% of the variation in length ratio on average (Fig. 4C; these nonphylogenetic results indicate that long‐necked plesiosaurs have proportionally higher cervical counts, but they do not indicate the pattern of change along evolutionary lineages that is responsible for this correlation). When PICs of length ratio are regressed against PICs of count ratio and unit length ratio in a multivariate analysis, they account for a median of 94.5% of the variation (0.930 < *R* ^2^ < 0.958). Similar results are obtained when the age of Plesiosauria is not constrained (median 33.4% [unit length ratio], 76.1% [count ratio] and 94.2% [count ratio + unit length ratio] of the variation in body proportions explained; Fig. S4). Taken together, these results indicate that homeotic shifts were important controls on sauropterygian body plan evolution, and that changes in the lengths of individual vertebrae caused by differential postpatterning growth of somites among regions of the axial column played a lesser, but nevertheless significant role.

**Figure 5:**
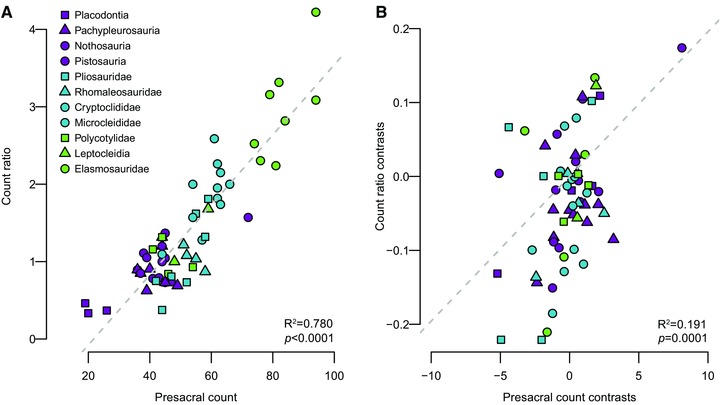
Correlations between cervical/dorsal vertebral count ratio (homeotic effects) and presacral vertebral count (somitogenesis). Scatterplot and best fit lines for an example topology for ordinary least squares regression of (A) raw count ratio against presacral count; (B) standardized PICs of count ratio against standardized contrasts of presacral count.

Regressions of the PICs of count ratio against those of presacral count can be used to test for a macroevolutionary relationship between homeotic shifts and somitogenetic change (Müller et al. 2010). A positive correlation between the two would indicate that these processes generally changed with similar relative magnitude and direction along evolving lineages at the time granularity of our study. We found a weak but significant positive correlation (*P* < 0.01 for all trees; Fig. S6) in which PICs of presacral count explained a median of 21% of the variation in those of count ratio (0.127 < *R* ^2^ < 0.297 [across 100 phylogenies]; Fig. 5). The results on unconstrained trees were almost identical (median 21% of variation explained, 0.123 < *R* ^2^ < 0.297 [across 100 phylogenies]; e.g., Fig. S5).

Regression or correlation tests between the full set of PICs for presacral counts and cervical/dorsal count ratios represent a homogeneous test of the evolutionary relationship between somitogenesis and homeotic effects, in the sense that they assume that a single relationship applies across all lineages. However, it is possible that a heterogeneous model applies, in which taxa from different time periods or clades have different relationships between evolutionary changes in the variables of interest, obscuring the relationship when all data are analysed together. This can be tested by examining standardised PIC correlations for nonplesiosaurian (exclusively Triassic) and plesiosaurian (largely post Triassic) taxa separately. In fact, similarly to the results for rates of evolution in count ratio and length ratio in the previous analysis, we find little support for a heterogeneous model of evolution. The correlations for both subsets show similar strengths and significance levels to analyses of all data together: nonplesiosaurians show marginally higher (but not significantly different, see ANCOVA results below) correlations ranging from 0.130 < *R* ^2^ < 0.378 with a median of 24.5% variation explained and all of these correlations are statistically significant. Plesiosaurians show similar correlations ranging from 0.082 < *R* ^2^ < 0.352 and all are statistically significant (Fig. S6). For trees with an unconstrained origin of Plesiosauria, these values were 0.139 < *R* ^2^ < 0.375, all significant (nonplesiosaurian), 0.068 < *R* ^2^ < 0.365, all significant (plesiosaurian) (Fig. S6). An analysis of covariance that included time period (Triassic or Jurassic‐Cretaceous) as an interaction term showed that the regression slopes for Triassic and Jurassic‐Cretaceous taxa are not significantly different for any of the topologies (at α = 0.05, results in Supplementary Information).

**Figure 6:**
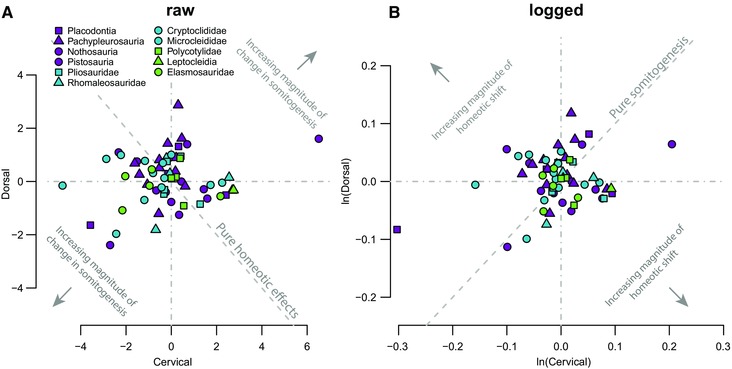
Rarity of (A) homeotic shifts without evolutionary change in somitogenesis and (B) evolutionary change in somitogenesis without homeotic shifts. Scatterplots of standardized PICs of (A) raw dorsal count against raw cervical count and (B) logged dorsal count against logged cervical count. Dashed gray lines show the expectation for the position of points that correspond to pure homeotic or pure somitogenetic change, and the further away from the origin a point lies on this diagonal, the higher the magnitude of this change. The larger the perpendicular distance of a point from the diagonal in each scatterplot, the larger the magnitude of a co‐occurring evolutionary change in the alternative mechanism.

To visualize the interactions between evolutionary change in somitogenesis and homeotic effects we compared standardized contrasts of cervical and dorsal counts. The dashed gray line in Fig. 6A shows the expected relationship between the PICs of cervical and dorsal count when only homeotic change has occurred (i.e., with constant counts of total presacral vertebrae), and the dashed gray line in Fig. 6B shows the expected relationship between the PICs of log‐transformed cervical and dorsal counts when only change in somitogenesis has occurred (i.e., proportional change, with a constant ratio of cervical/dorsal vertebrae). Strikingly, few points lie on or close to the expectation for either pure homeotic shifts or pure somitogenetic change (Fig. 6). All pure homeotic shifts are low in magnitude (Fig. 6A). Across all topologies the majority of pure somitogenetic changes are low in magnitude with the exception of the datapoint corresponding to the node between the pistosaurian taxa *Diandongosaurus* (Shang et al. 2011) and *Yunguisaurus* (see comment in previous results section). This shows that large homeotic shifts without a coincident change in the number of somites do not occur, and that changes in somitogenesis without a homeotic shift are rare.

## Discussion

Across vertebrates as a whole the relative sizes of axial regions are highly variable. In sarcopterygians, and among amniotes (the fully terrestrialized tetrapods) in particular, evolutionary elongation or shortening of the axis is concentrated among the precaudal regions (e.g., cervical, dorsal, thoracic) (Müller et al. 2010; Ward and Mehta 2010, 2014). Evolutionary variation in precaudal regions was maintained in the several groups that subsequently transitioned back to an aquatic mode of life (Callaway and Nicholls 1997; Thewissen and Williams 2002) and sauropterygians provide a clear example of this. Relative proportions of axial regions are of great functional relevance to both locomotor modes and feeding strategies. Understanding the developmental processes that govern axial organization, and how they interact on macroevolutionary timescales to generate such disparate body proportions is therefore of fundamental relevance to understanding vertebrate evolution as a whole. Sauropterygia provides a useful system to relate developmental processes to morphology, and perhaps subsequently to function. They also provide an interesting comparison with evolutionary variation in axial organization in mammals, which fill similar aquatic niches (Kelley and Pyenson 2015) but have a highly conserved cervical count (Galis 1999; Varela‐lasheras et al. 2011).

Evolutionary variation in relative neck length can be generated either by a change in the relative number of vertebrae in each axial region (homeotic effects), or by a change in the average length of cervical vertebral centra as compared to the dorsal vertebral centra (differential postpatterning growth). We find that evolutionary changes in the body proportions of sauropterygians resulted primarily from changes in the relative numbers of cervical/dorsal vertebrae (Fig. 4D), indicating that most sauropterygian body plan disparity resulted from homeotic shifts rather than differential postpatterning growth of the cervical and dorsal regions. This is unlike the pattern seen in mammals. However, differential growth did occur and played a role in sauropterygian evolution, contributing approximately 38% of variation in body proportions along phylogenetic lineages (Fig. 4B). This finding is consistent with previous studies showing that within tetrapods, diapsids display greater evolutionary variability in presacral vertebral count ratios than synapsids (Müller et al. 2010; Ward and Mehta 2014; Böhmer et al. 2015). However, it also demonstrates that postpatterning growth plays an important role in generating body plan variation, even within clades that show a high evolutionary capacity for varying somite count and axial regionalisation.

We also show that rates of axial evolution varied among lineages within Sauropterygia. Perhaps surprisingly given the high disparity in axial body proportions seen in plesiosaurian sauropterygians compared to their Triassic relatives, rates of evolution of body proportions and vertebral count ratios vary little among clades or time periods (Fig. 3). In contrast, rates of evolution of presacral counts vary significantly across sauropterygian phylogeny. High rates occur principally on deep branches within Sauropterygia and Plesiosauria, and the total presacral counts of some long and short‐necked Triassic sauropterygians (placodonts and pistosaurians) show equal or faster median rates than those of most plesiosaurian lineages (Fig. 2). This indicates that considerable evolutionary variation in somite counts accrued during the early evolution of Sauropterygia, approaching the level of variation observed in extant squamates (Bergmann and Irschick 2011). Therefore, although evolutionary change in presacral counts facilitated the origins of end‐member long‐ and short‐necked morphotypes in Jurassic and Cretaceous plesiosaurians, these extremes did not result from unusually high rates of change in body proportions but instead resulted from directional change over long timespans (i.e., divergent trends). This may have been aided by somewhat elevated rates of change in numbers of somites in the clades with the most extreme long‐necked morphologies (Fig. 2: microcleidids, elasmosaurids). This suggests that these disparate plesiosaurian body plans were made possible by inheritance of high evolutionary plasticity from their Triassic ancestors, maintained throughout their long evolutionary history. Surprisingly, we find no evidence for any release of constraint in body proportions associated with the Late Triassic environmental transition to deep water.

We find that pure changes in somitogenesis (in the absence of homeotic shifts) are rare and pure homeotic shifts (in the absence of changes in somite count) are absent (Fig. 6). This observation is consistent with our finding of a weak correlation between somitogenesis and homeotic effects (Fig. 5). In fact, most evolution of the axial column of sauropterygians results from somitogenesis and homeotic effects acting together in a co‐ordinated way to produce relative changes in neck and trunk length. In this sense, there is clearly some interaction between the two processes. Macroevolutionary associations between somitogenesis and homeotic effects are most likely mediated by selection for functional body plans rather than by strict developmental linkages, which had received some support previously (Dubrulle et al. 2001; Zakany et al. 2001) but are generally rejected (Wellik and Capecchi 2003; McIntyre et al. 2007; Wellik 2007; Gomez and Pourquié 2009).

Plesiosaurian sauropterygians are unique among secondarily aquatic tetrapods in having maintained high plasticity in axial body proportions following an ecological shift to deeper water environments and obligate pelagic lifestyles, and plesiosaurian body proportions differ substantially from those of other marine tetrapods. This may have been facilitated by a combination of factors. First, obligate immersion in water results in high and permanent buoyancy forces that lessen the requirement for the neck to be supported against gravity; this is common to all pelagic tetrapods (e.g., plesiosaurians, whales, ichthyosaurs). In addition to this, however, and uniquely among secondarily aquatic tetrapods, plesiosaurians possessed lift‐based limb‐driven propulsion and stabilization, enabled by the presence of four large flippers (Halstead 1989; Long et al. 2006; Liu et al. 2015). This combination allowed body size increases and elongation of the neck in some plesiosaurians (e.g., microcleidids, elasmosaurids), which was not seen in other groups of secondarily aquatic diapsids (which used axial‐based locomotion and evolved fish‐like body plans, e.g., ichthyosaurs; Motani 2005). Secondarily aquatic mammals, while in some cases showing extreme size increases (e.g., whales), retain the ancestral mammalian count of seven cervical vertebra, which has been shown to be developmentally constrained in almost all mammals (hypothesized to be due to links between cervical somites and other developmental processes, see, e.g., Buchholtz and Stepien 2009; Varela‐lasheras et al. 2011; Buchholtz 2014), and perhaps in the total‐group of mammals more generally (Synapsida; Müller et al. 2010; Buchholtz 2014). Furthermore, secondarily aquatic mammals, similarly to ichthyosaurs, use axial undulation for their locomotion, representing a functional constraint that may have prohibited the evolution of substantially long necks in these groups.

Variation in the relevance of different developmental modes to macroevolutionary change in body plans occurs among tetrapod clades. For instance, changes in somite count, in the absence of changes in primaxial Hox gene expression, were important in the origin of snakes (Head and Polly 2015) and important homeotic shifts have occurred during archosaur evolution (Böhmer et al. 2015). Our analyses show that the relative importance of the developmental mechanisms that generated differences in axial body proportions also varied within the evolutionary history of Sauropterygia. The evolutionary capacity to substantially change body proportions through homeotic effects was maintained consistently throughout their evolutionary history, whereas changes in somite counts were more prevalent among early sauropterygians, and showed more variation on a local phylogenetic scale. This observation provides an impetus toward further application of heterogeneous phylogenetic models of evolution in both detailed studies of individual clades and large‐scale studies, which so far have used homogeneous models (Müller et al. 2010), or nonphylogenetic approaches (Ward and Mehta 2010, 2014).

Associate Editor: A. Evans

Handling Editor: P. Tiffin

## Supporting information


**Figure S1**.

**Figure S2**.

**Figure S3**.

**Figure S4**.

**Figure S5**.

**Figure S6**.

**Figure S7**.

**Figure S8**.

**Figure S9**.

**Figure S10**.

**Comparative‐dataset**.

**Phylogenies‐and‐results**.

**R‐script‐and figures**.

## Appendix

Analysis of log‐transformed trait values

The main analyses involving length ratio and count ratio were repeated with natural log length ratio and natural log count ratio. Estimates of logged length ratio rates showed elevated rates in Triassic taxa relative to later branches. It is unclear to what extent this is an artefact of logging the ratio, which–given that there was not good evidence for a relationship between relative neck length and the magnitude of its variance–may exaggerate rate estimates in shorter necked taxa, many of which were found in the Triassic (Fig. S10). Logged count ratio rate estimates similarly showed a pattern of higher than average rates in the early part of the tree, followed by consistent rates in plesiosaurian taxa.

Phylogenetic independent contrast analyses on logged ratios gave results consistent with the main analyses (Fig. S10). PICs of logged length ratio showed a significant correlation with logged unit length ratio on all trees (0.501 < *R* ^2^ < 0.649) and a very good correlation with count ratio that was significant on all trees (0.634 < *R* ^2^ < 0.802). As in the main analyses, PICs of logged count ratio against presacral count showed a weak but always significant correlation (0.125 < *R* ^2^ < 0.296).

Analyses of logged length and count ratios are broadly consistent with the conclusions presented in the main text. The higher rates supported in Triassic taxa may be exaggerated by using logged variables, even so this pattern is consistent with our overall conclusion that the high body plan disparity of plesiosaurian sauropterygians was a result of inheritance of evolutionary plasticity of the presacral region from their Triassic ancestors.
