# Supplementary figures and images for: Developmental mechanisms of macroevolutionary change in the tetrapod axis: A case study of Sauropterygia
Source: Evolution. 2017 Mar 21;71(5):1164–77. doi: 10.1111/evo.13217 (PMC5485078; doi:10.1111/evo.13217)

A

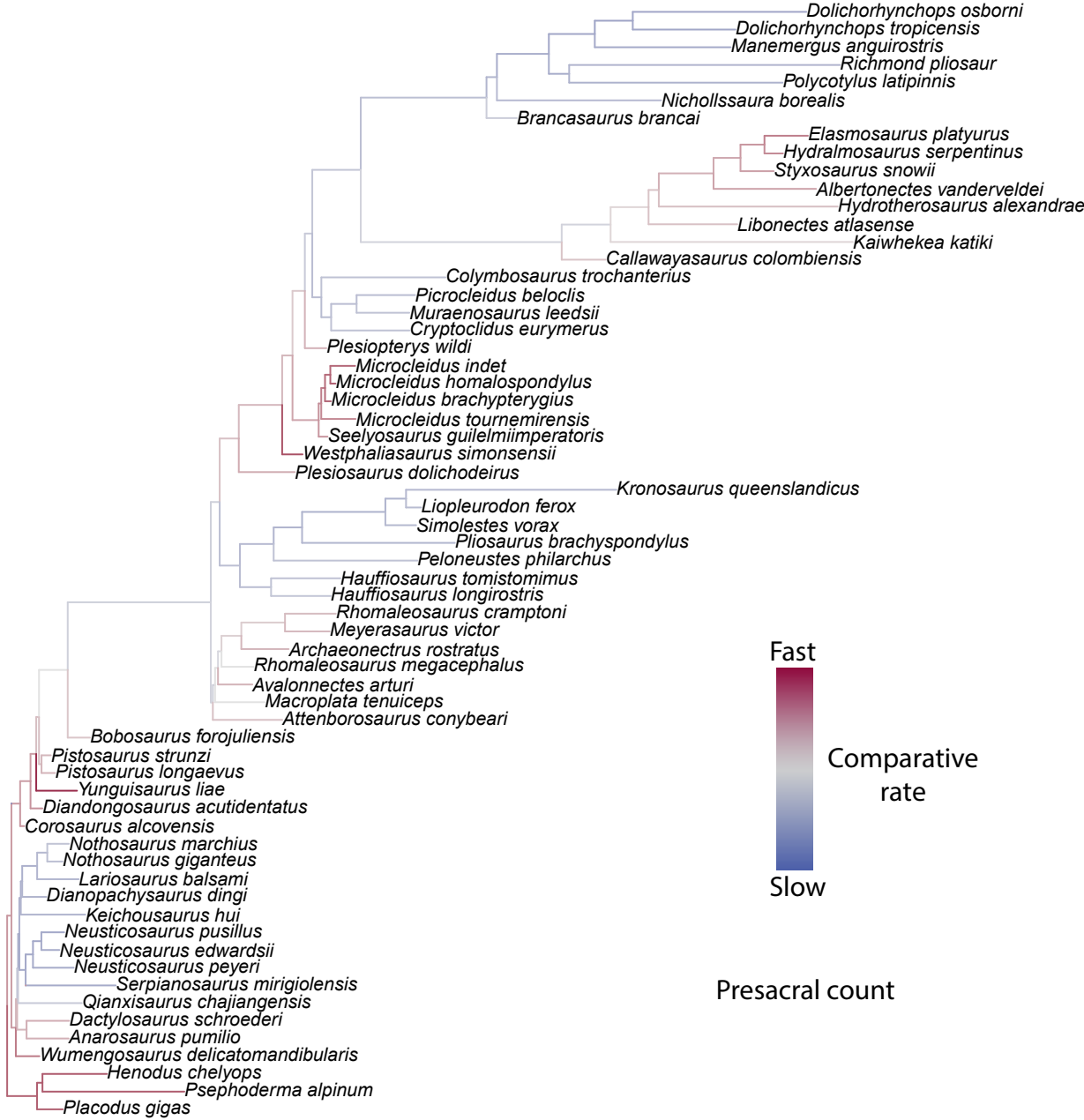

Fast

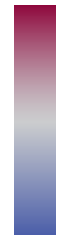

Slow

Comparative  
rate

Presacral count

B

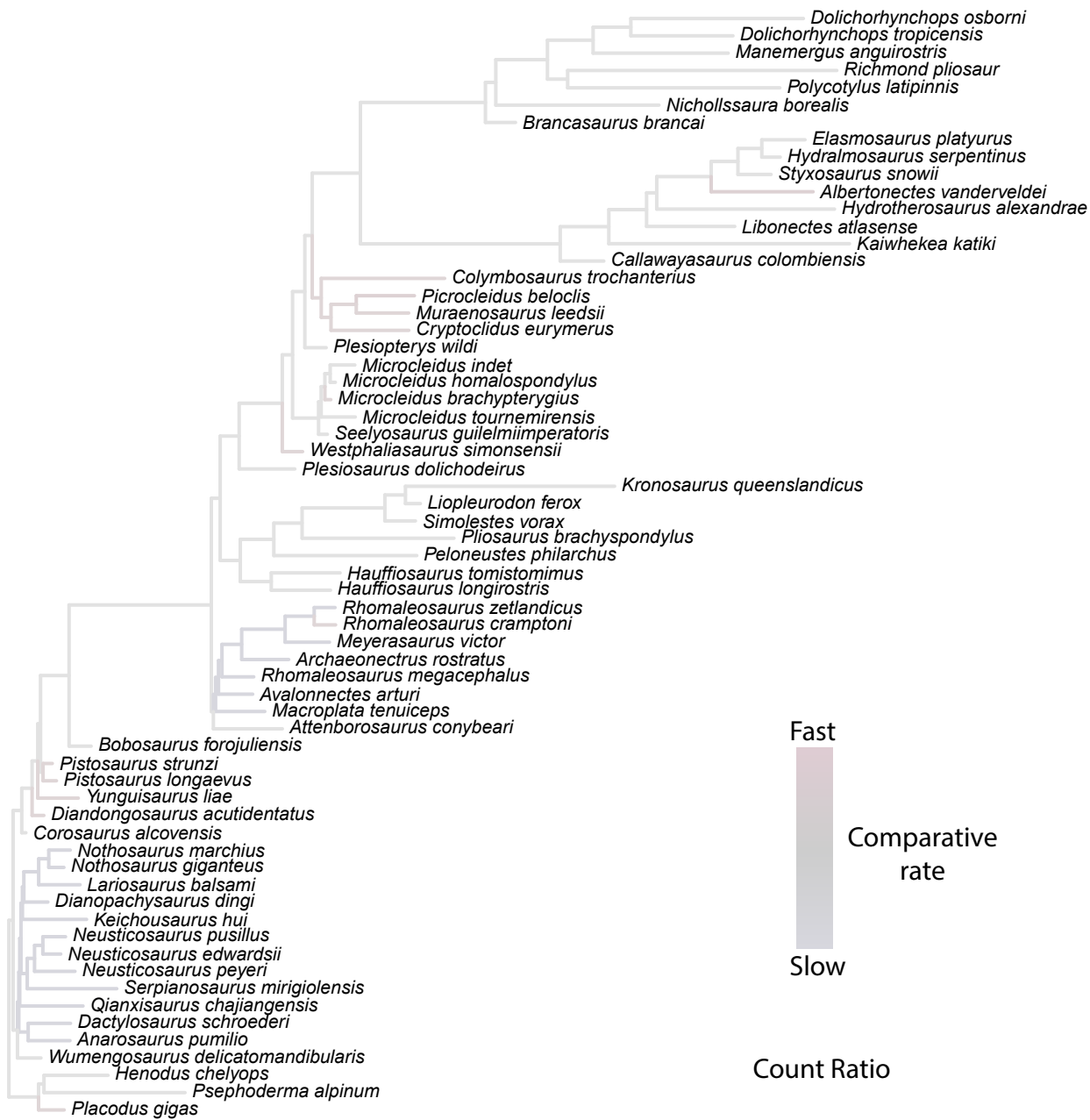

C

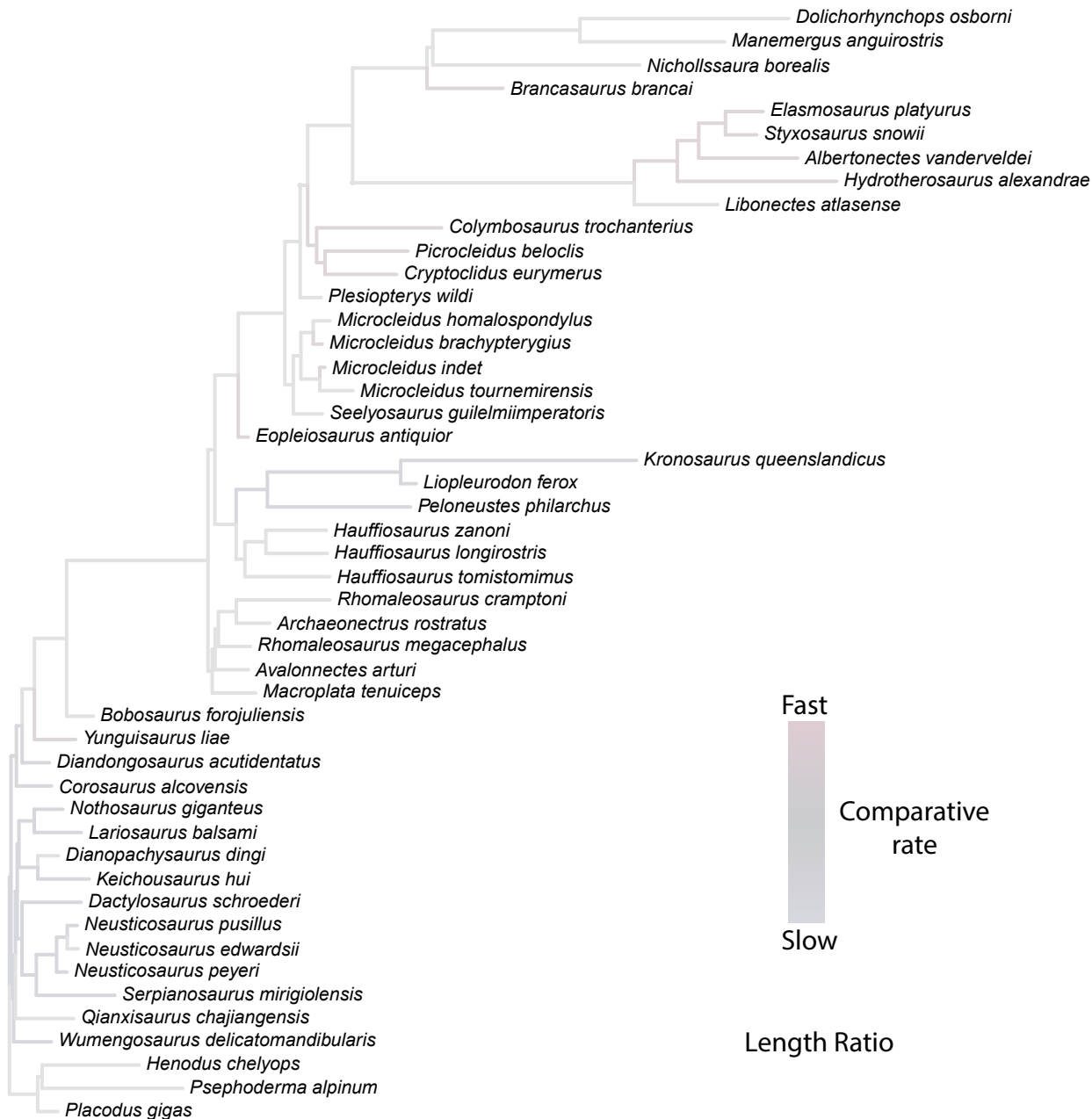

Supplement: Supplementary file 1 — Figure S1. [file EVO-71-1164-s001.pdf]

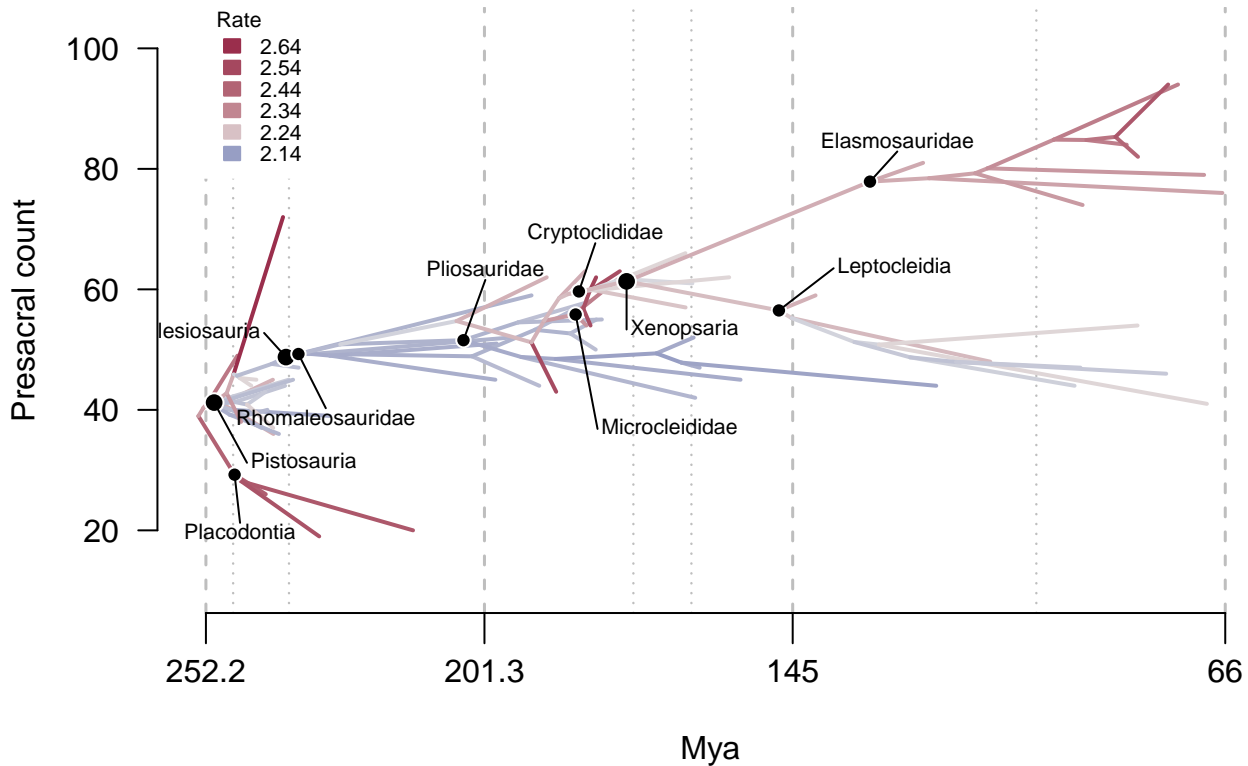

Supplement: Supplementary file 2 — Figure S2. [file EVO-71-1164-s002.pdf]

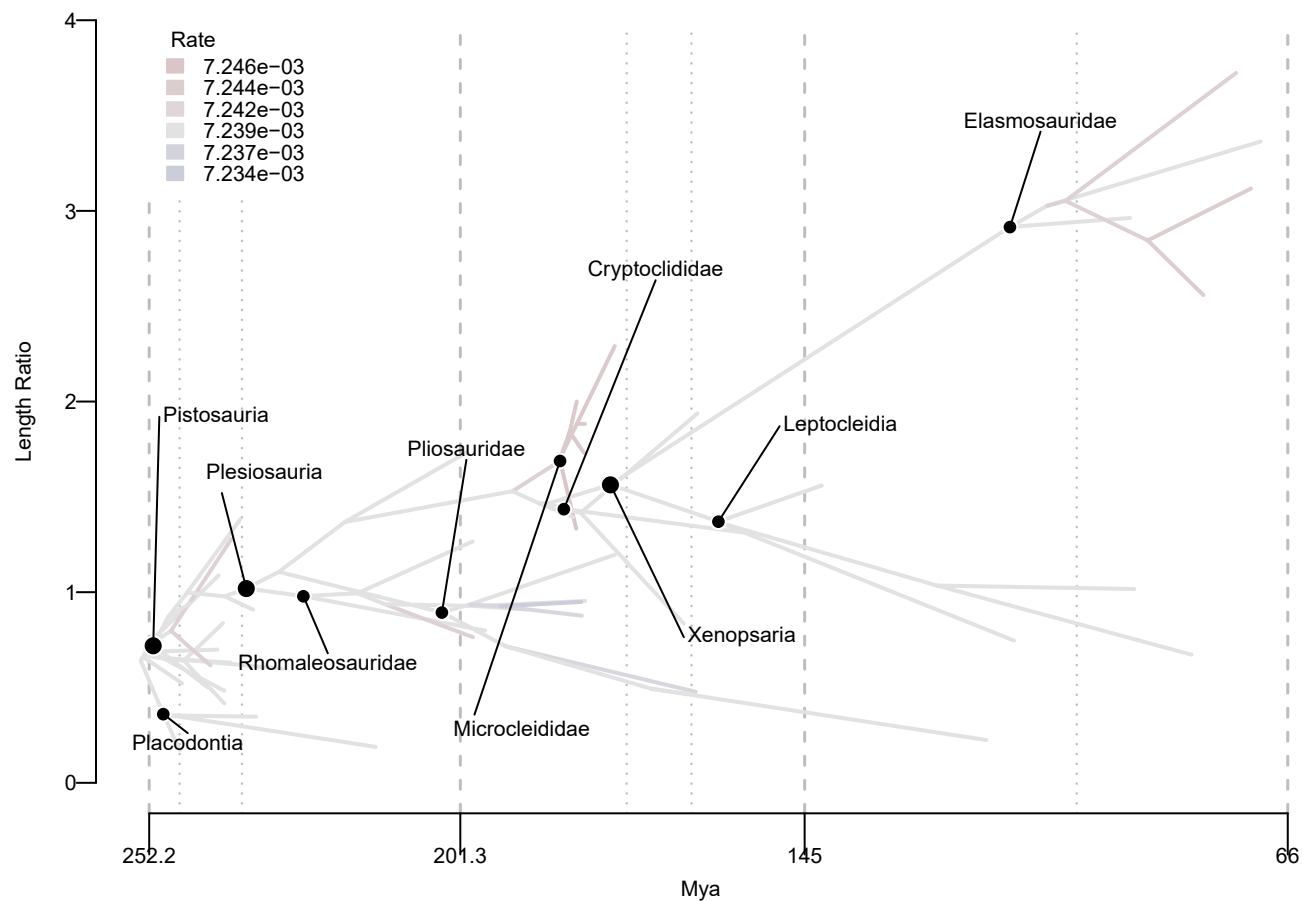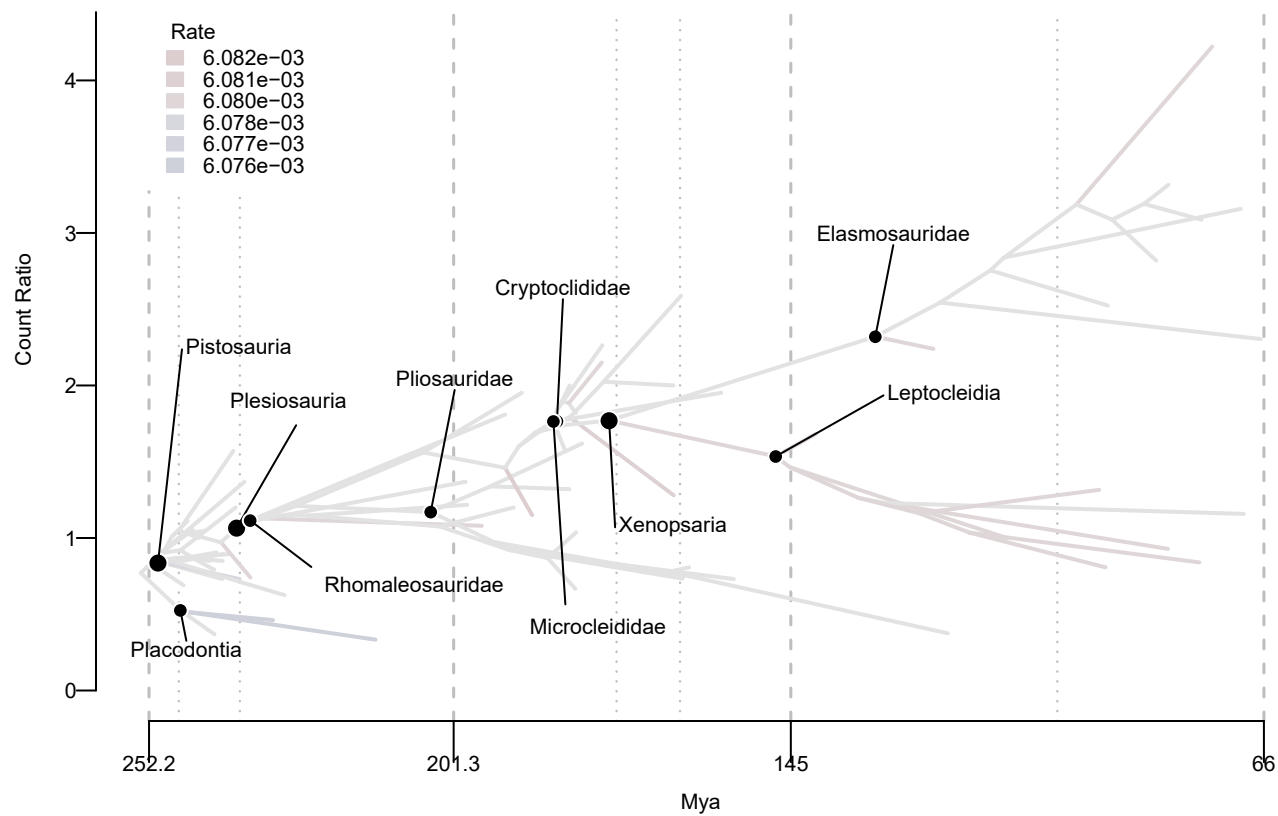

Supplement: Supplementary file 3 — Figure S3. [file EVO-71-1164-s003.pdf]

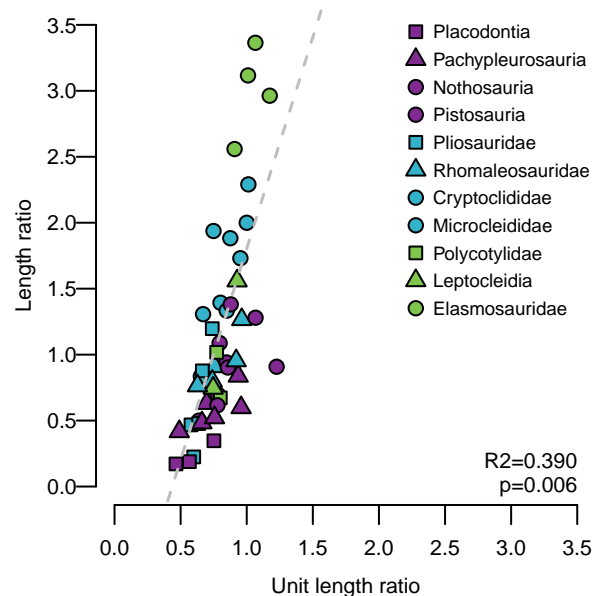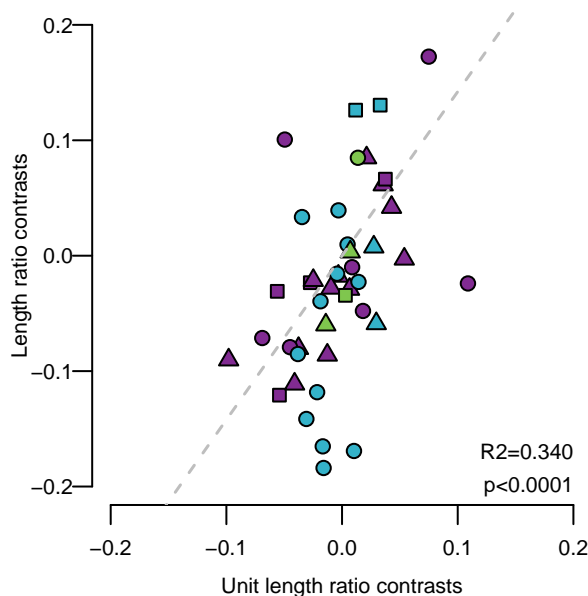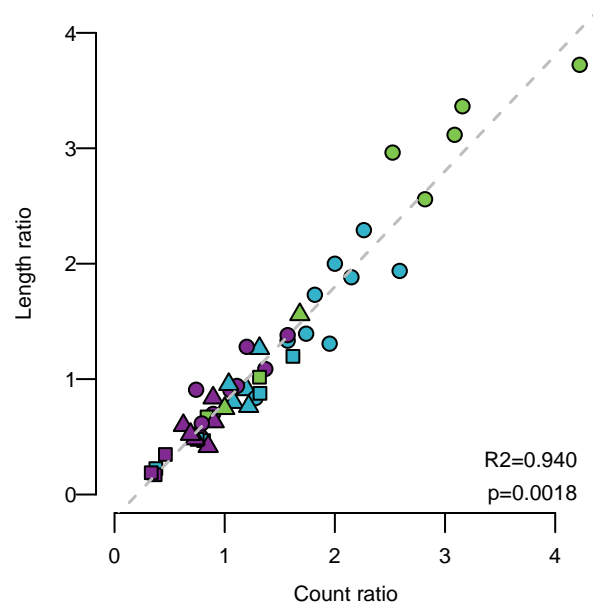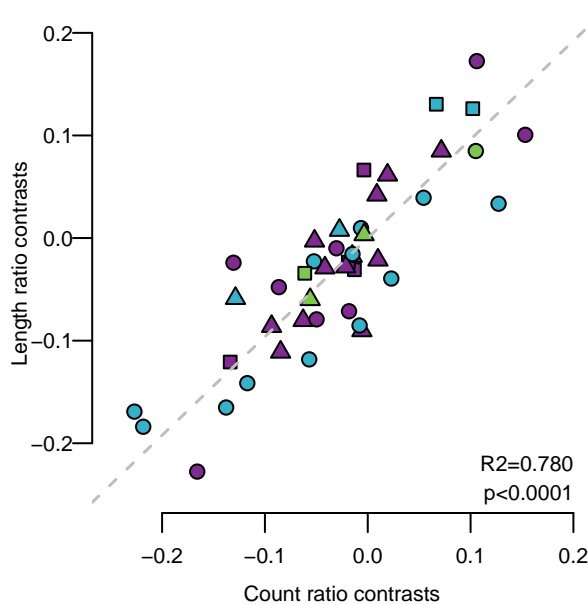

Supplement: Supplementary file 4 — Figure S4. [file EVO-71-1164-s004.pdf]

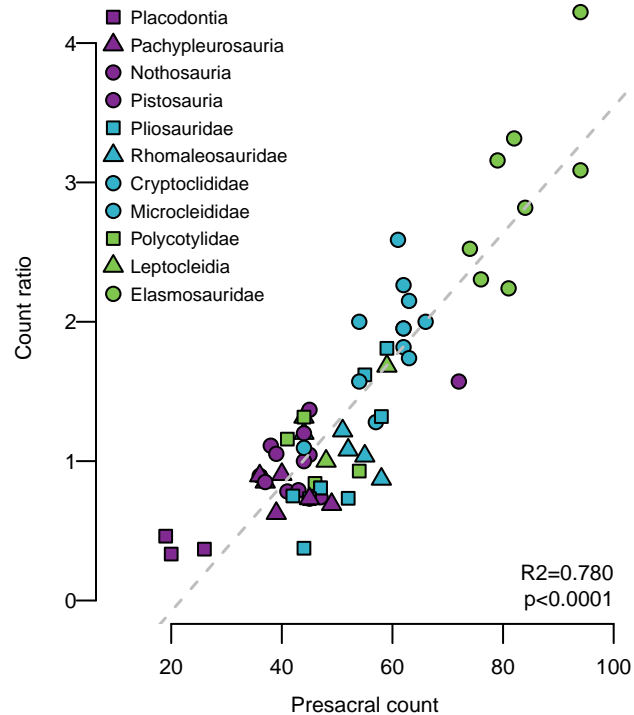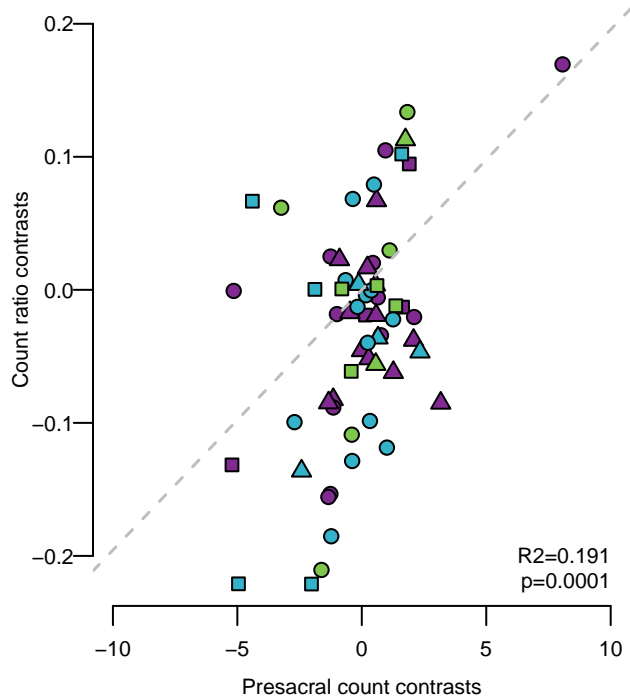

Supplement: Supplementary file 5 — Figure S5. [file EVO-71-1164-s005.pdf]

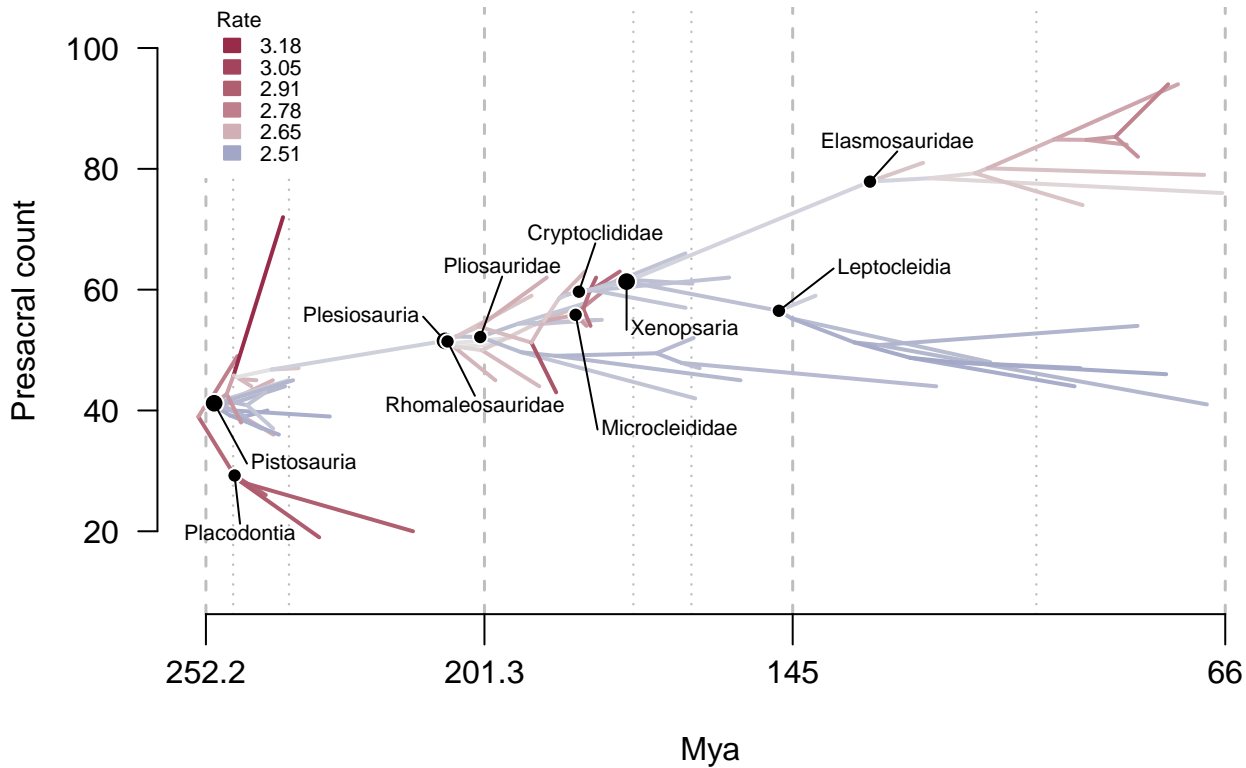

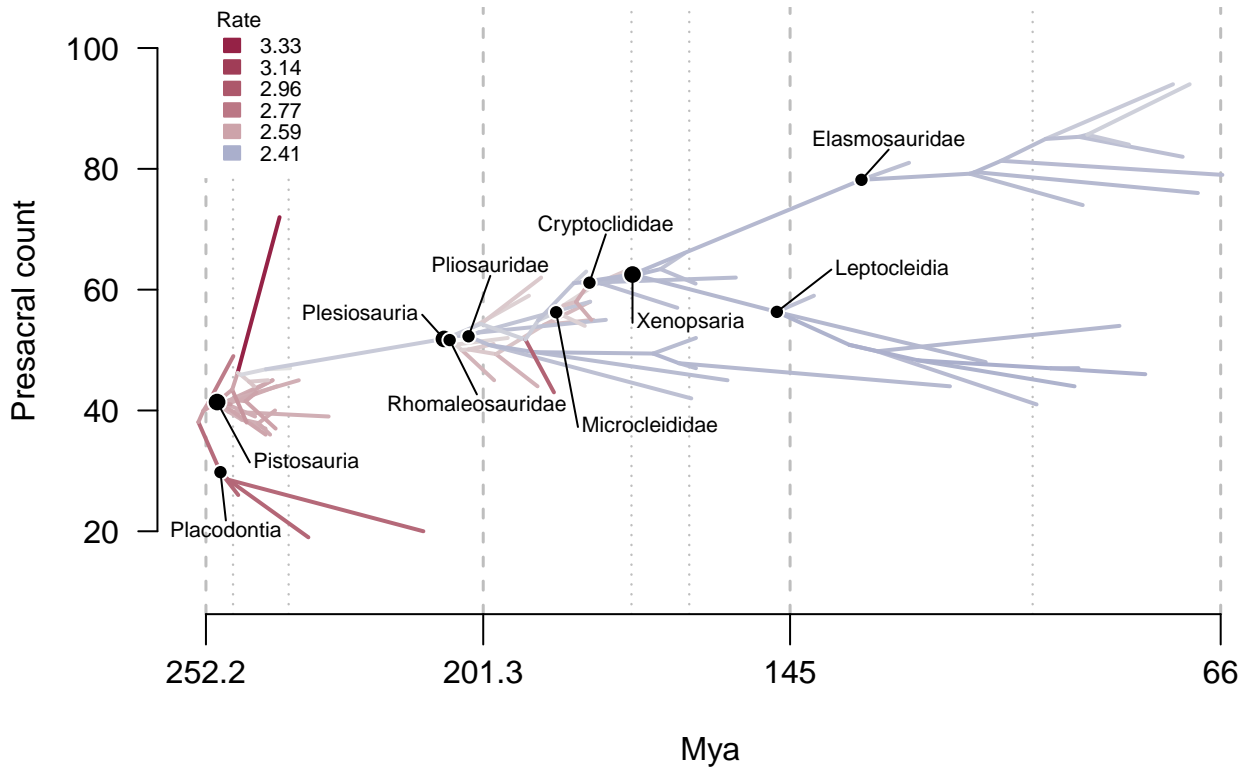

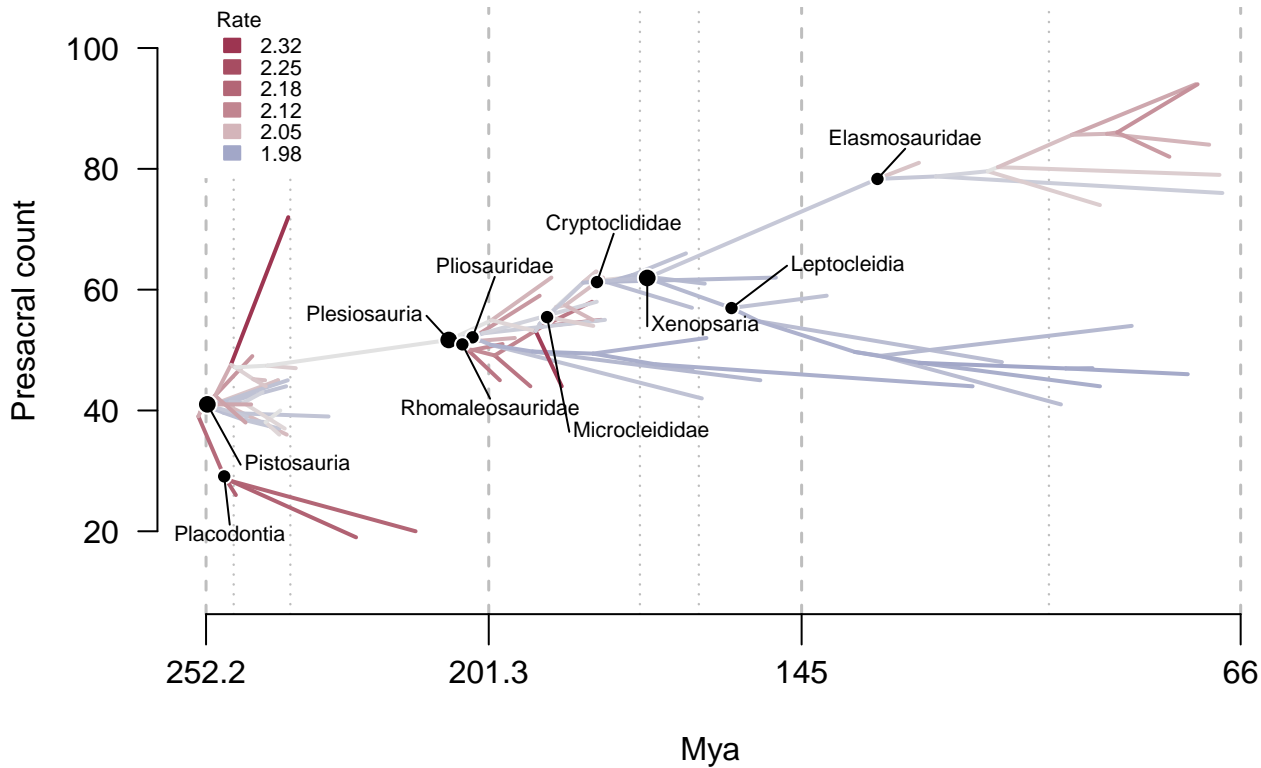

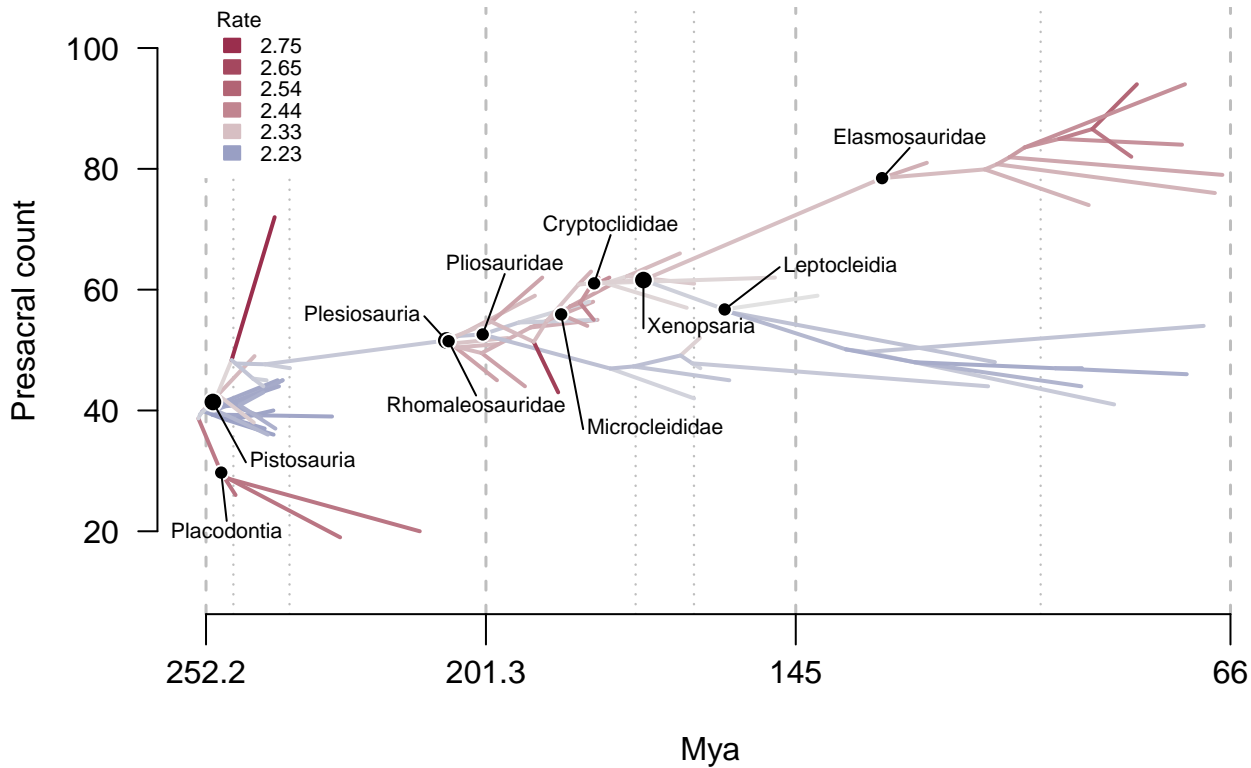

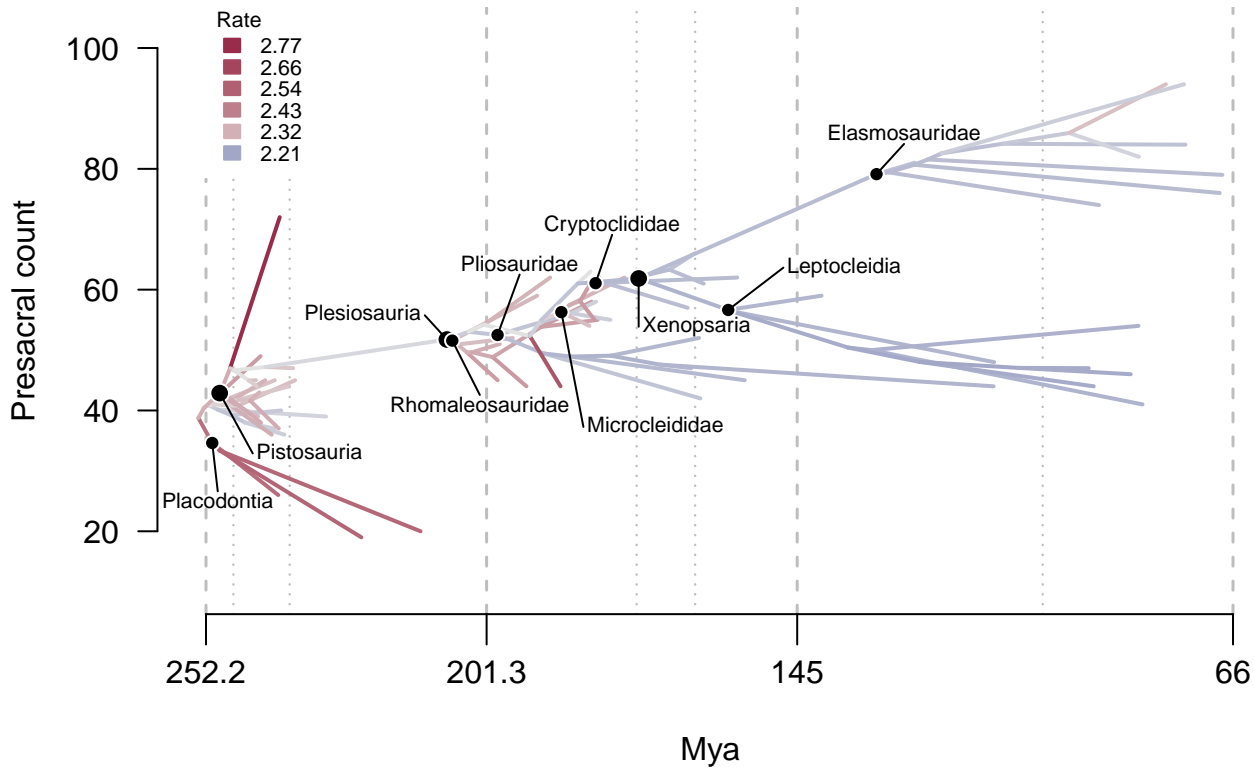

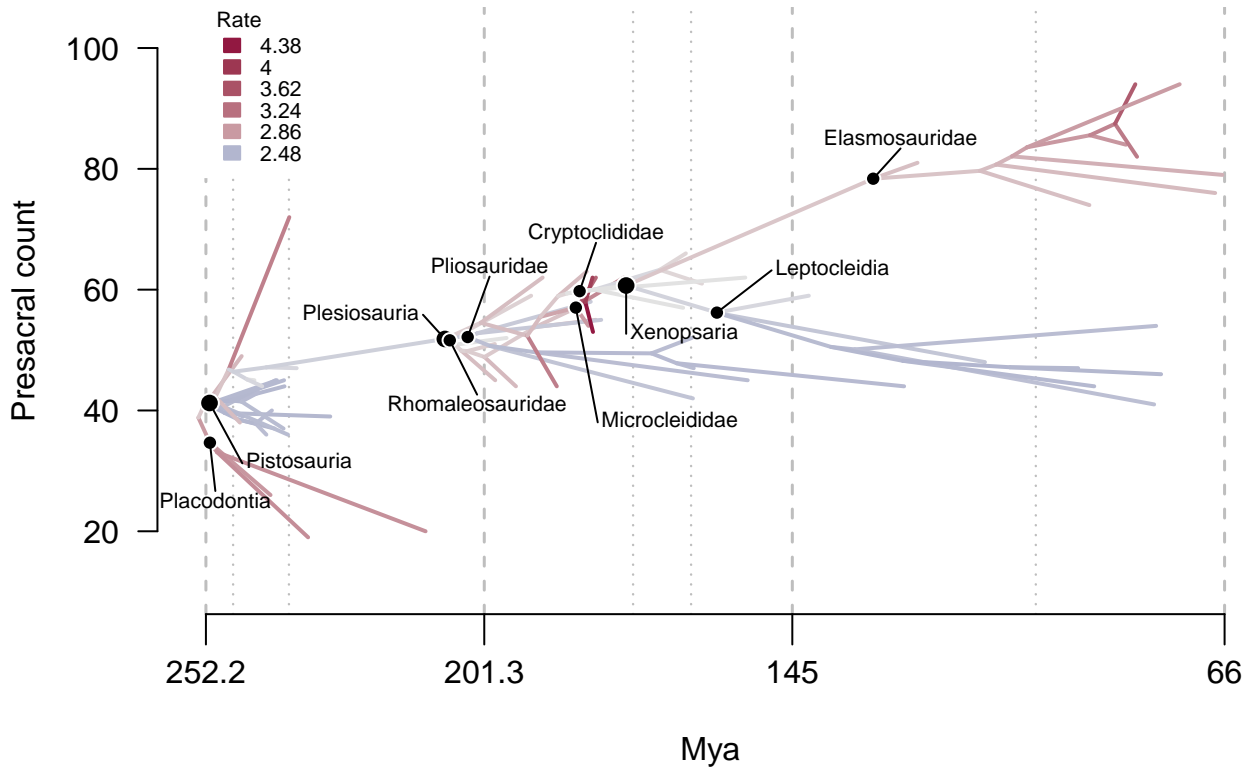

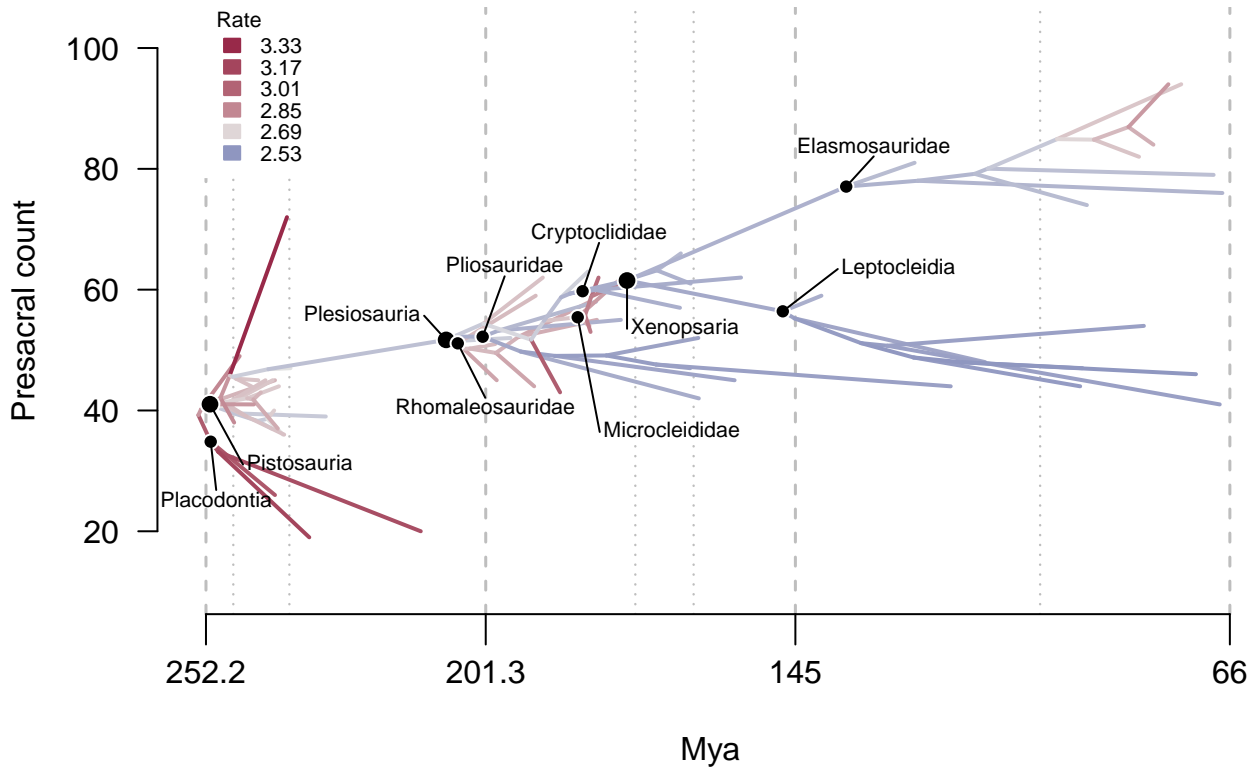

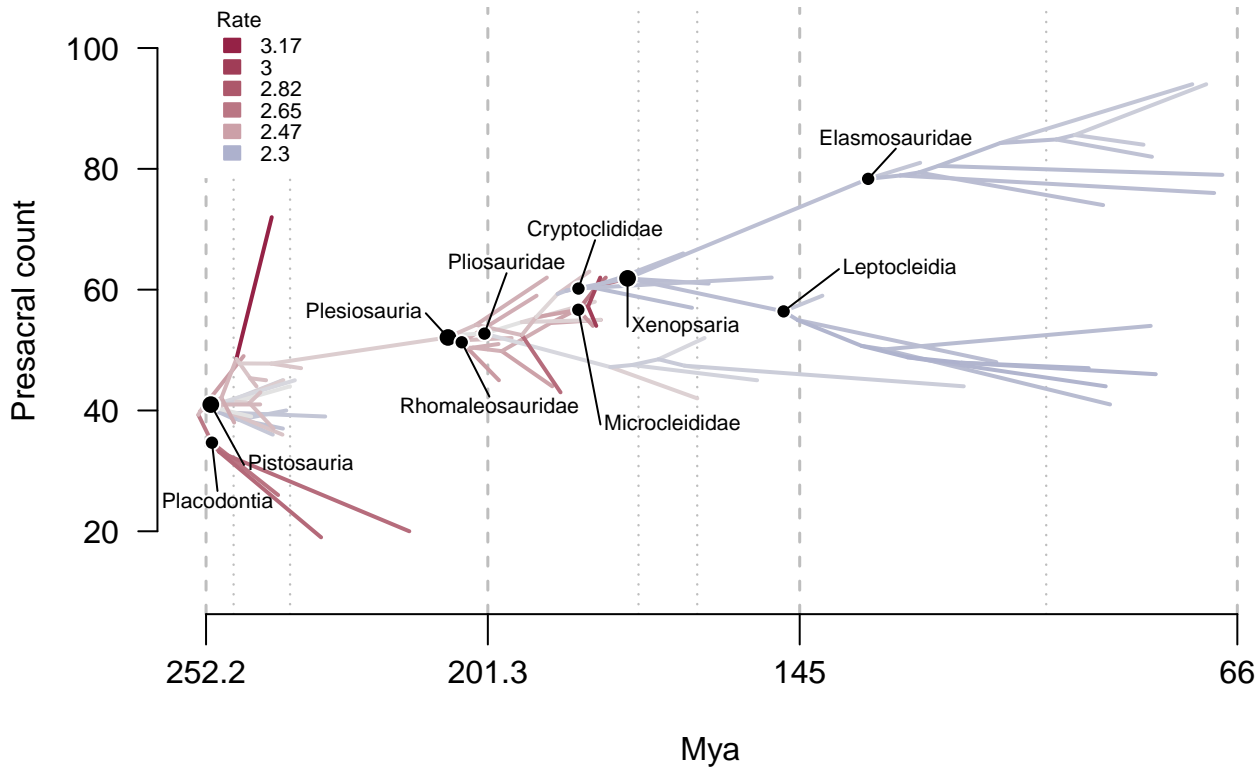

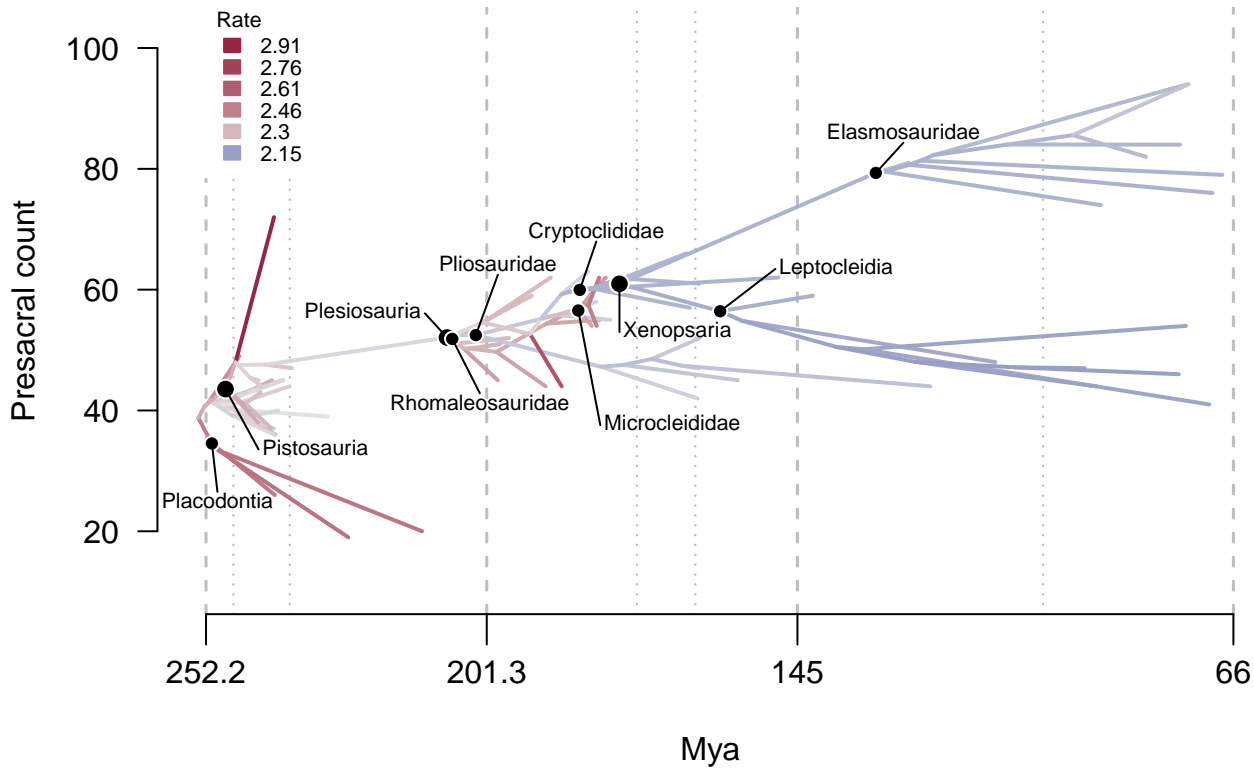

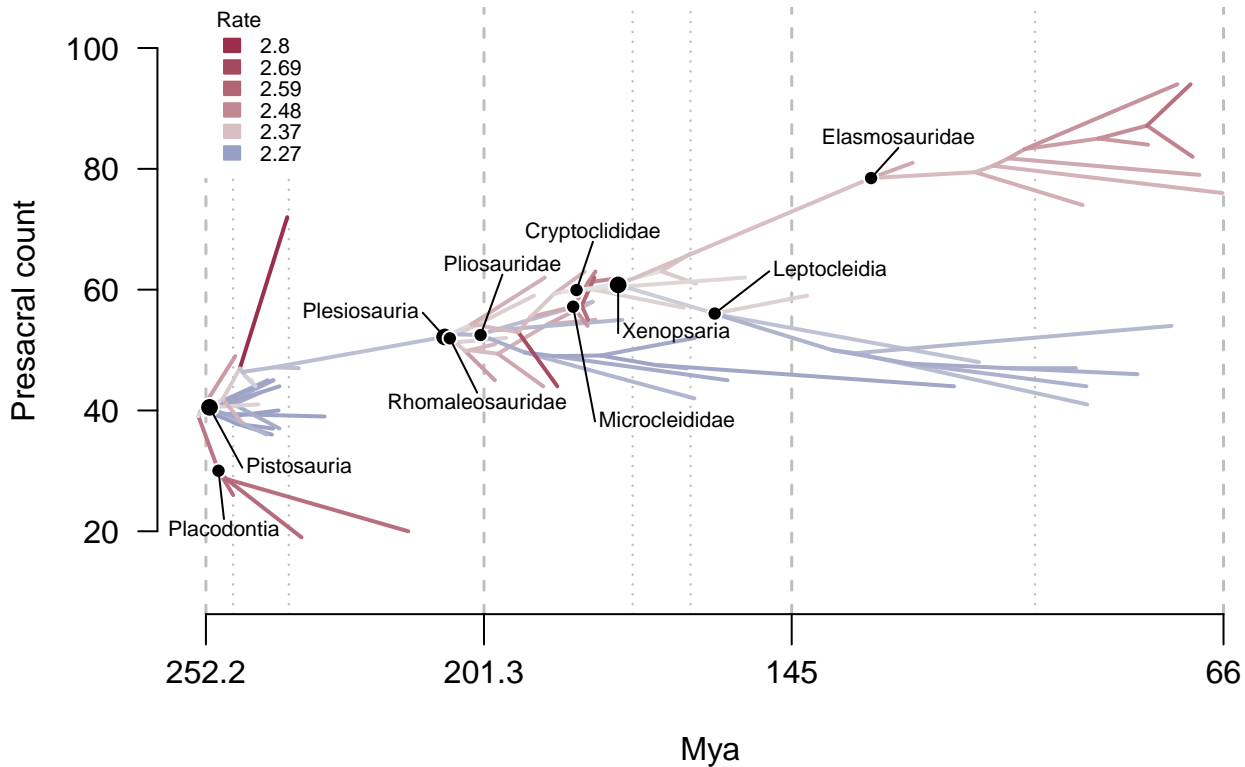

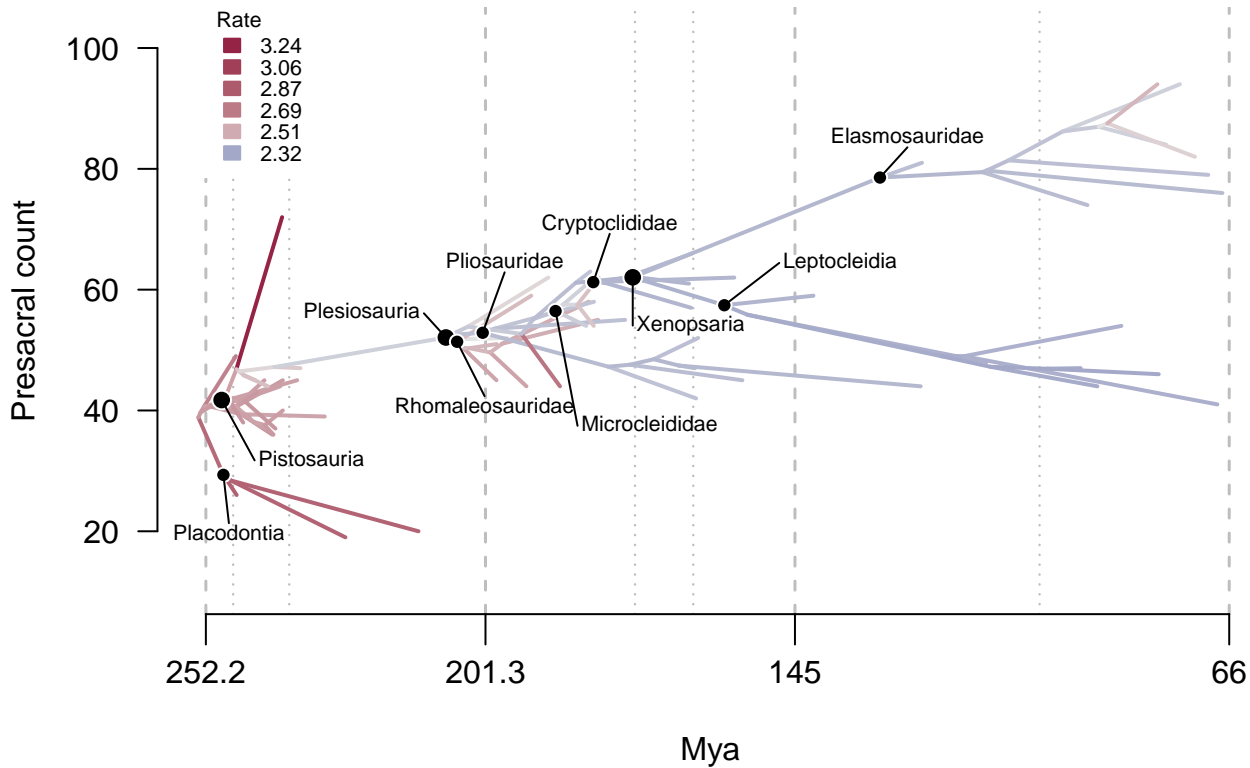

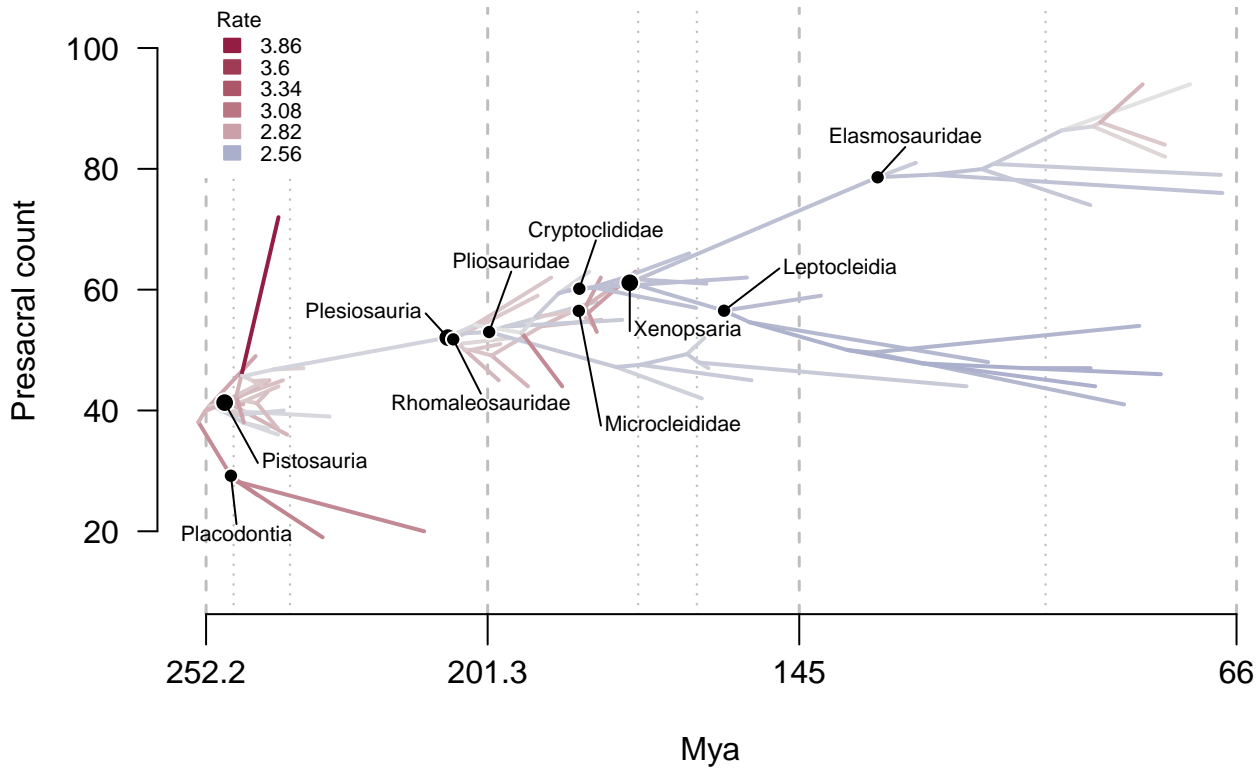

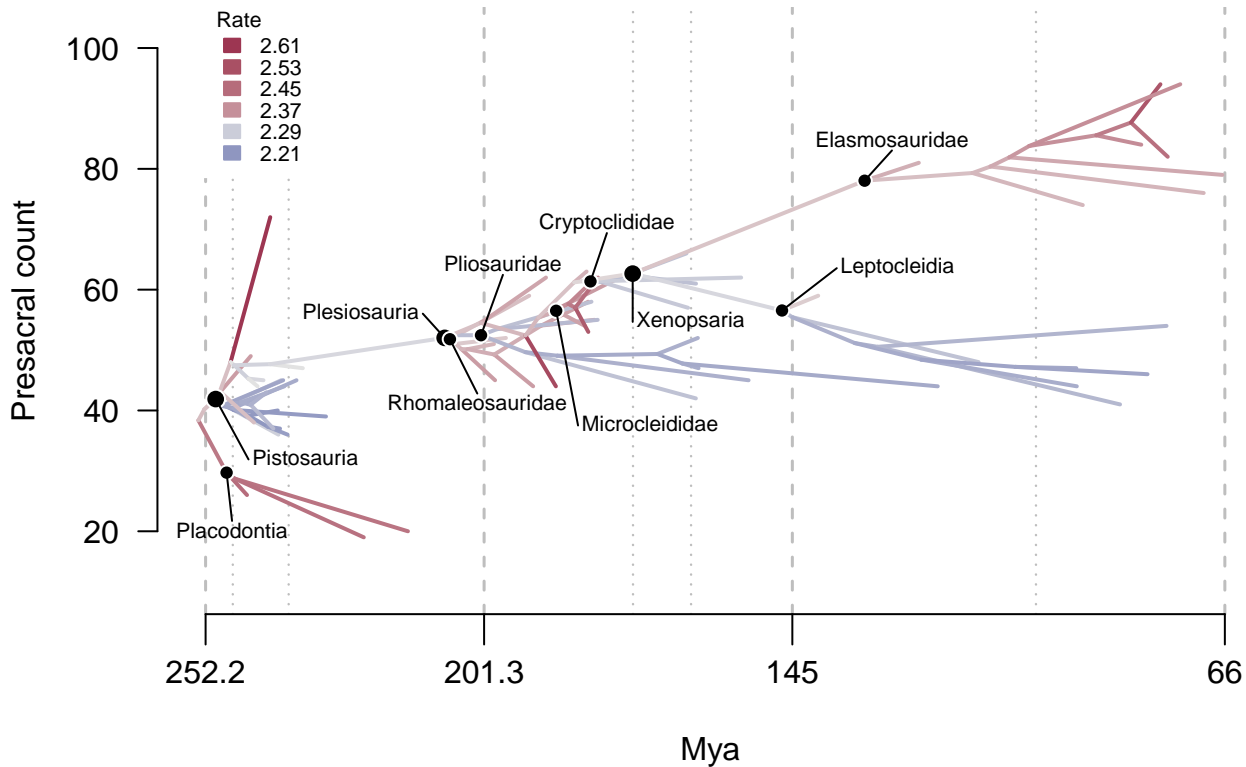

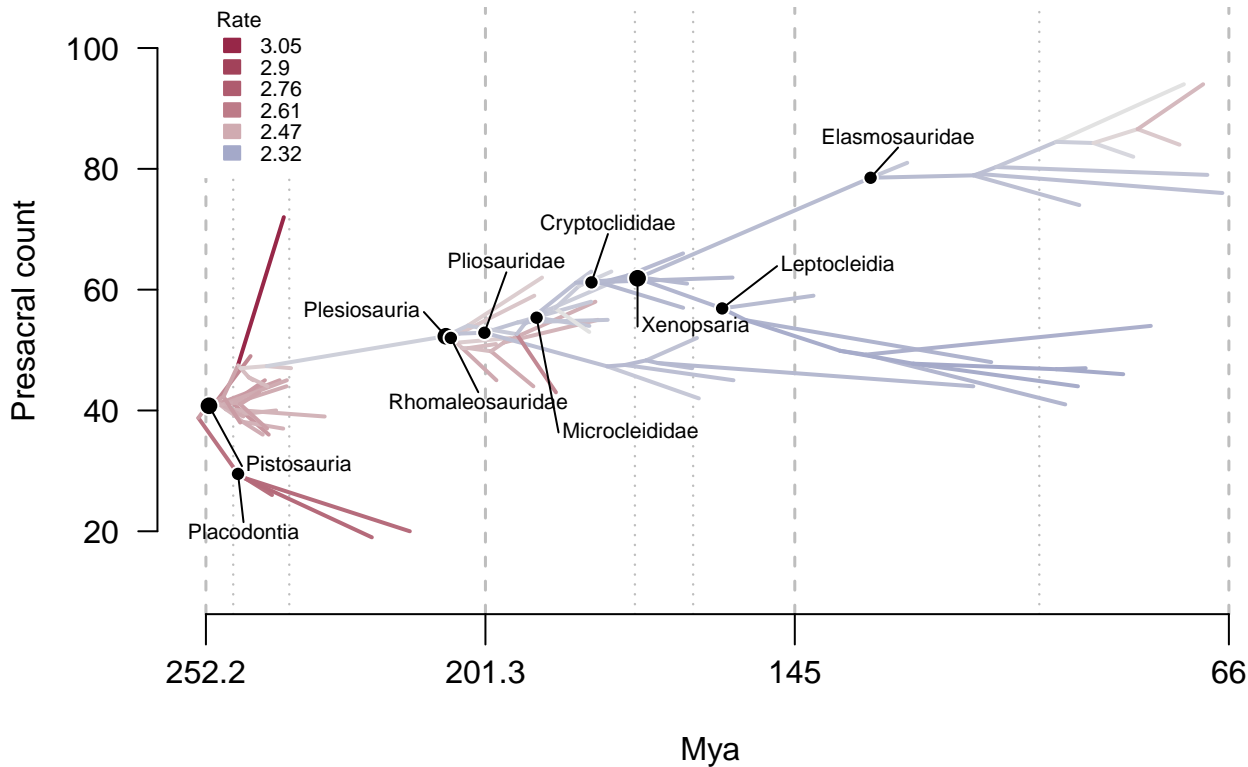

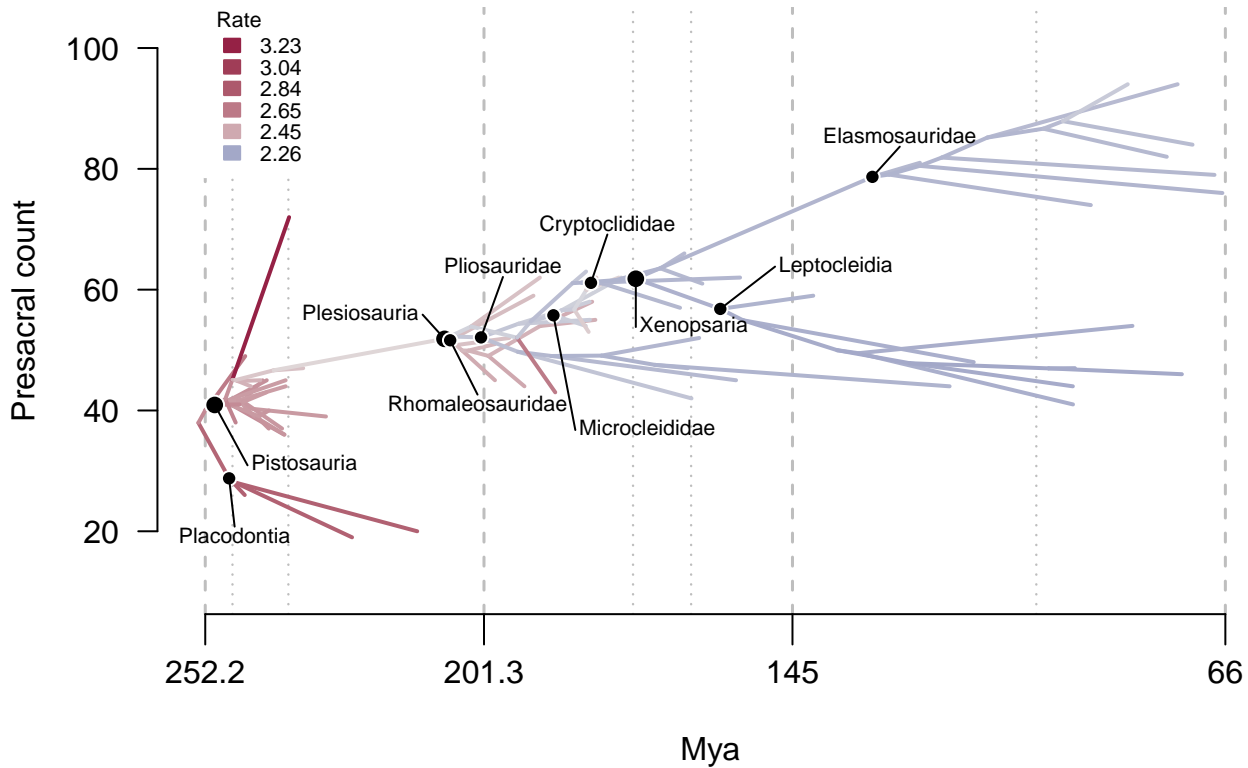

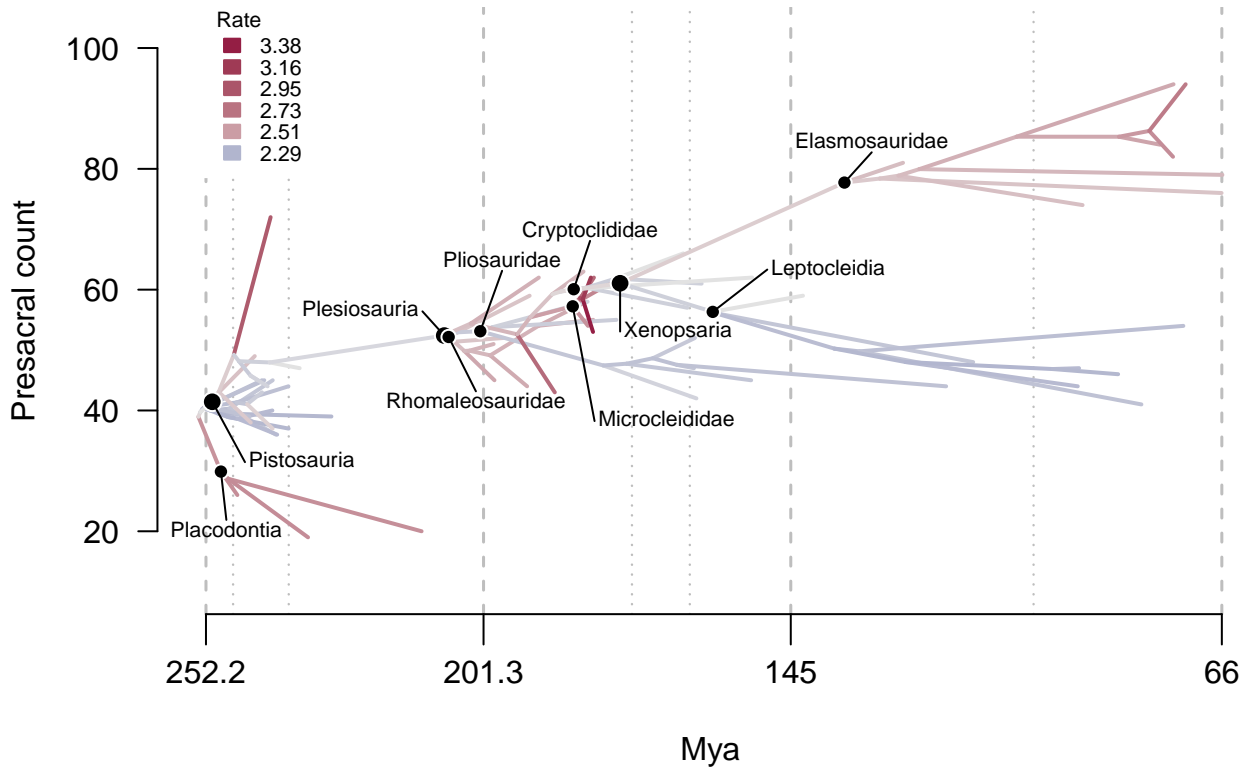

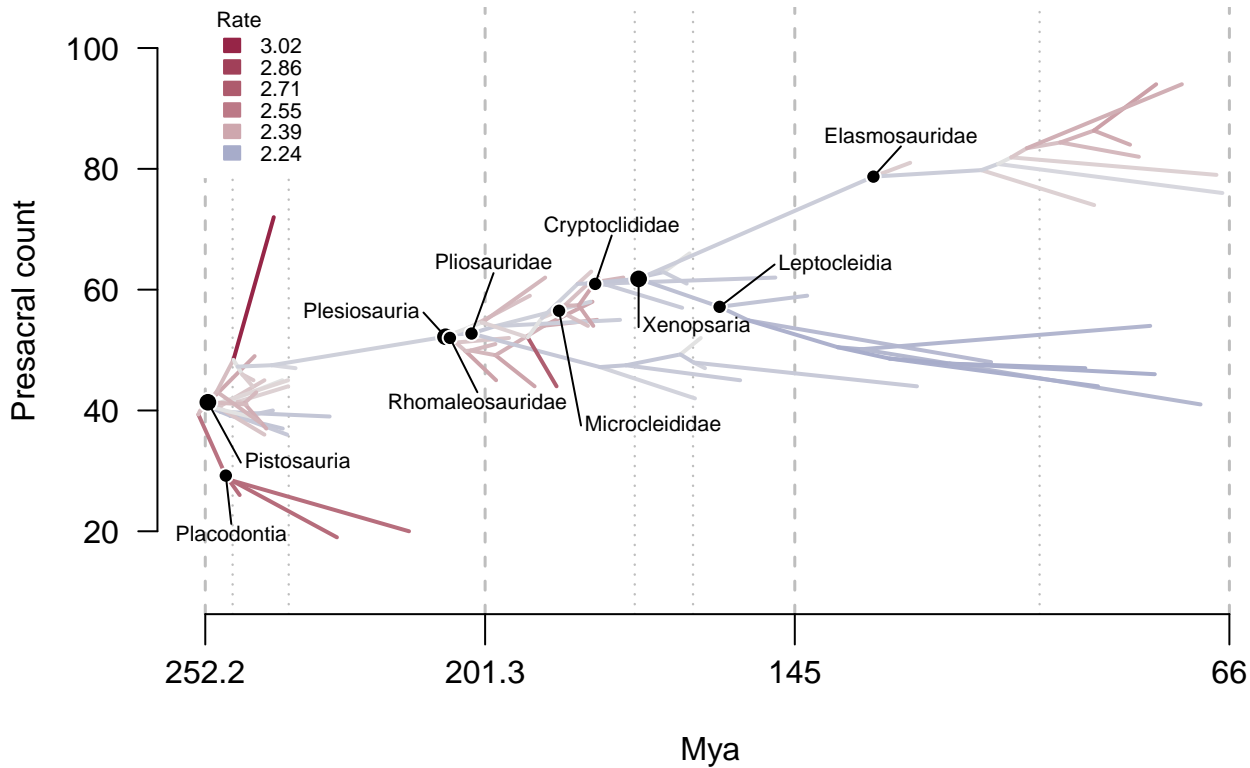

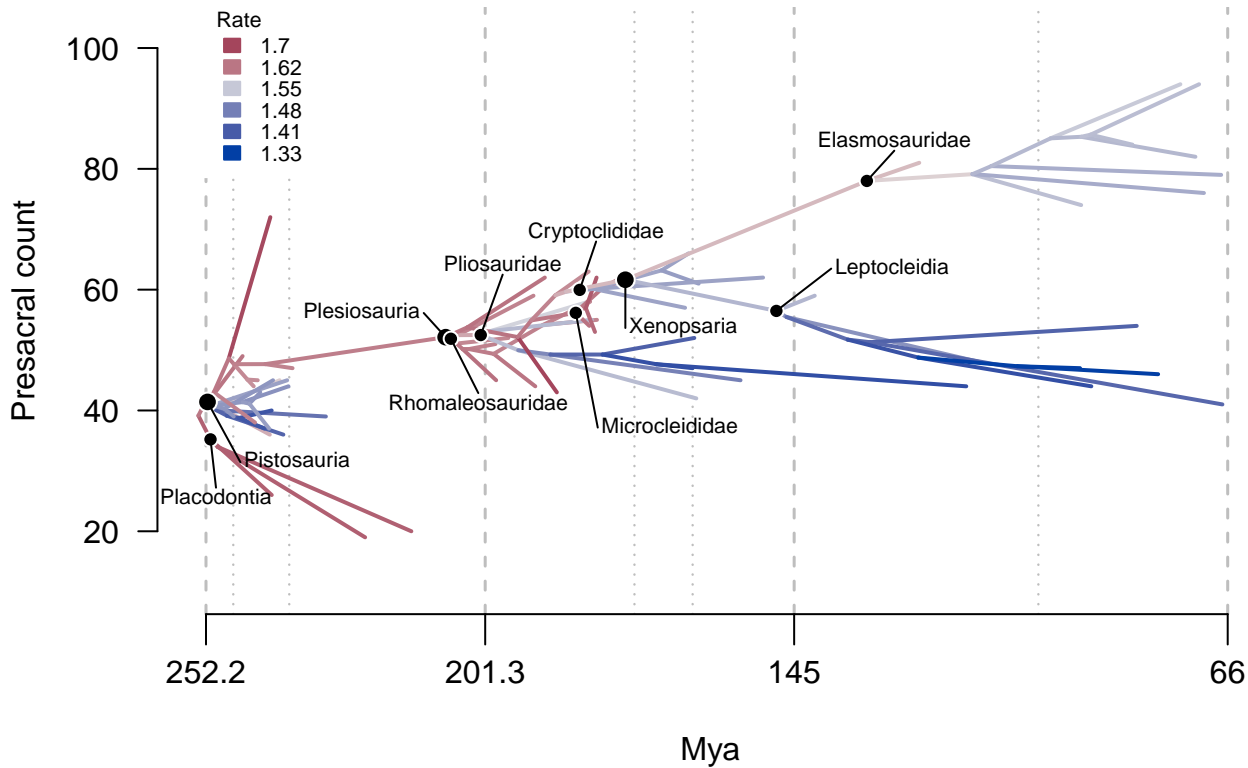

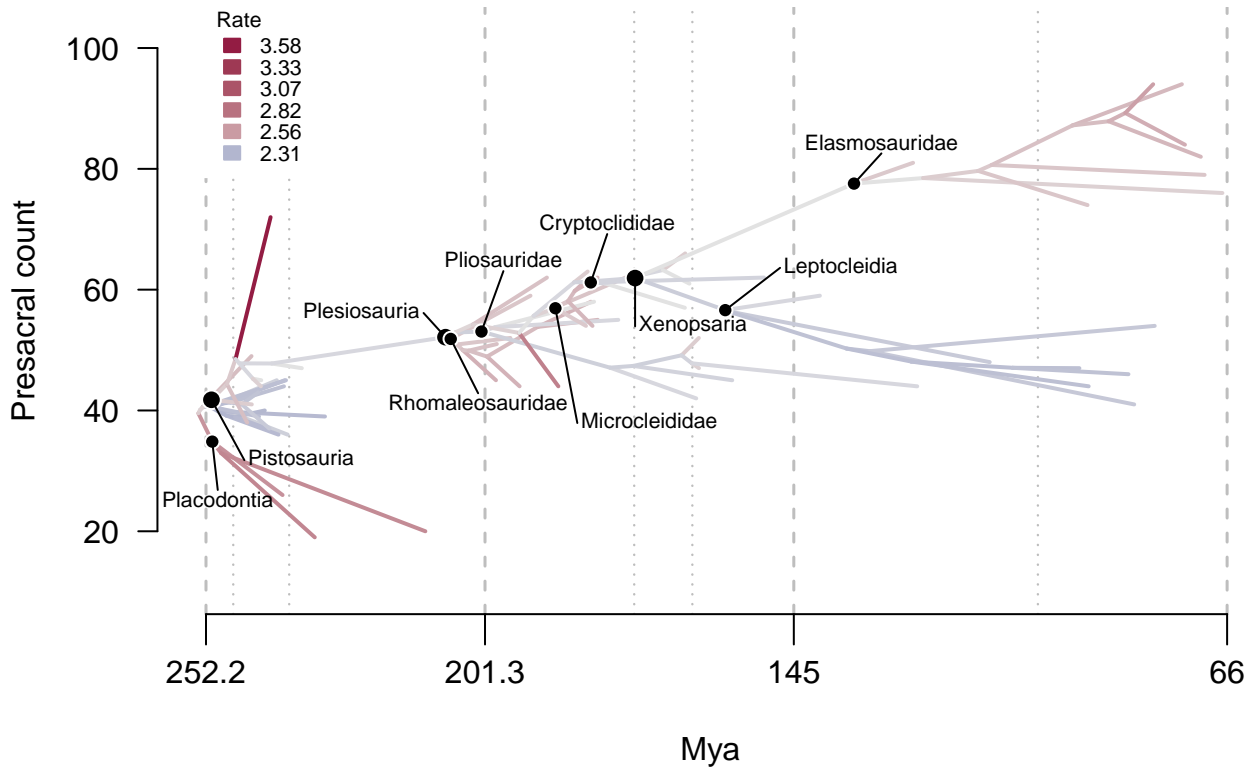

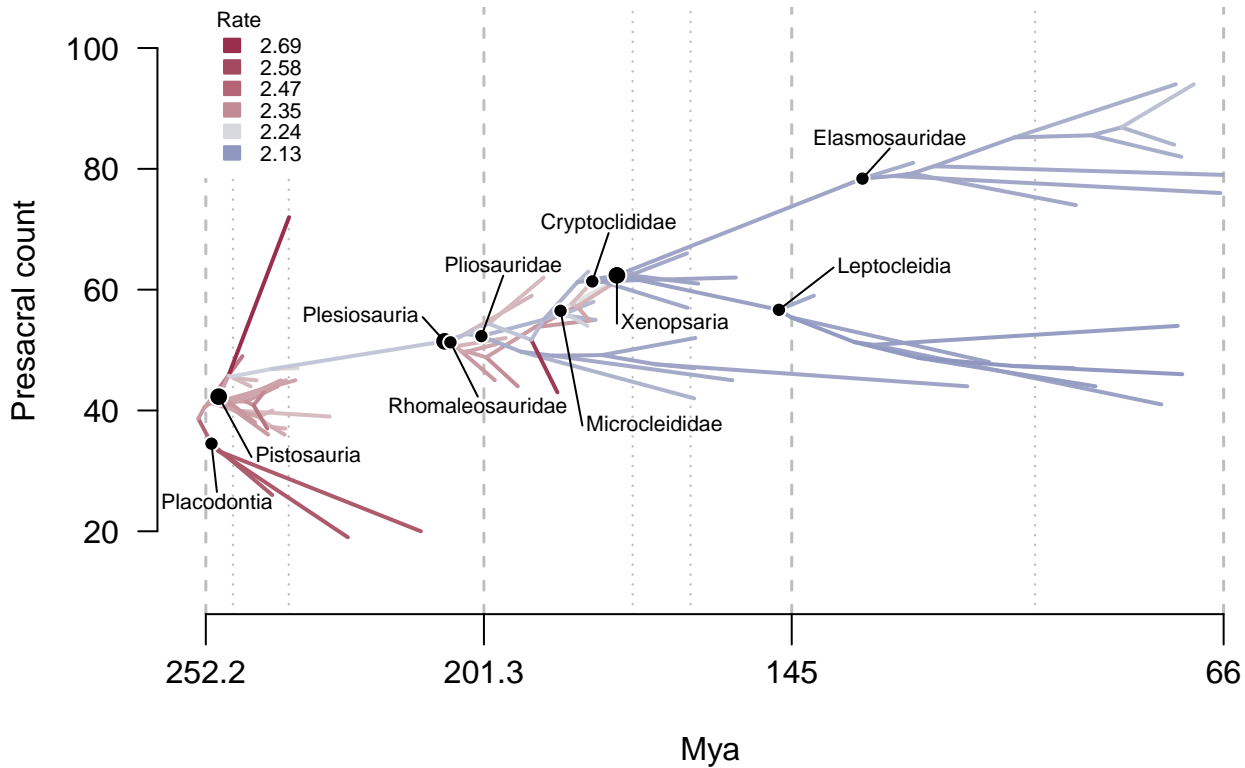

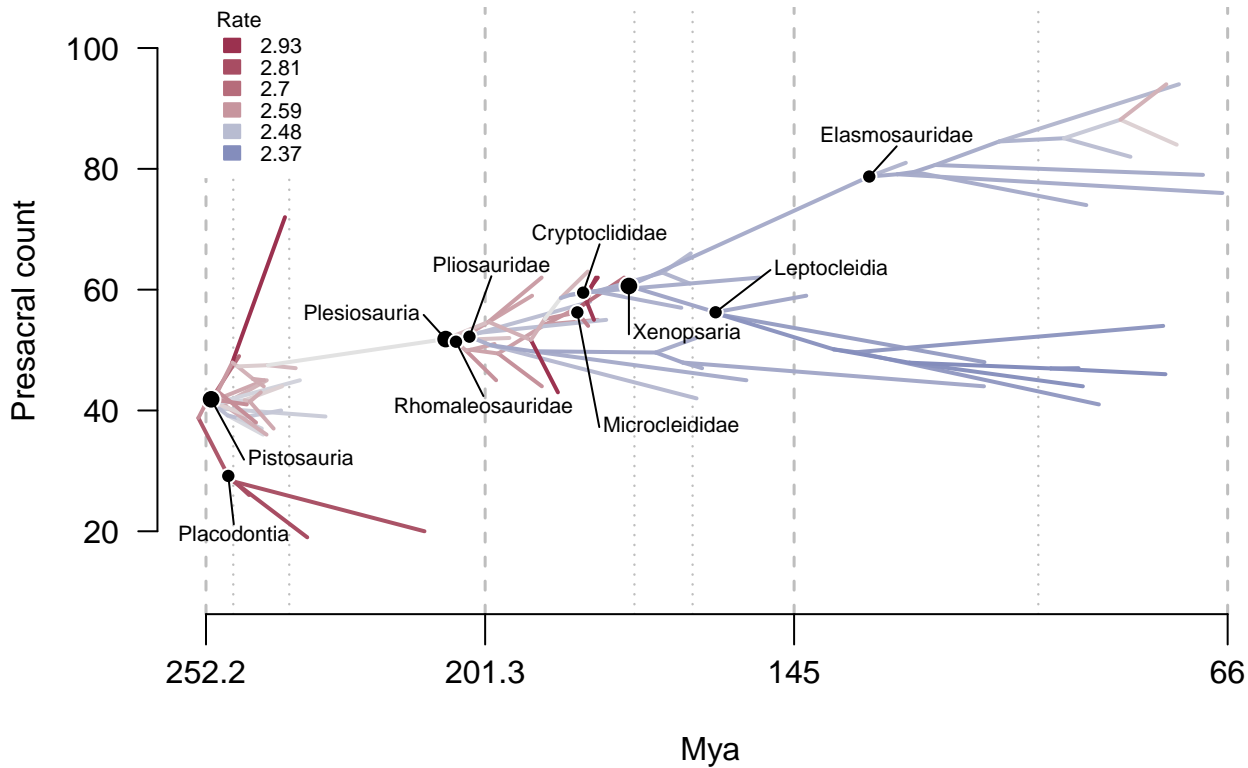

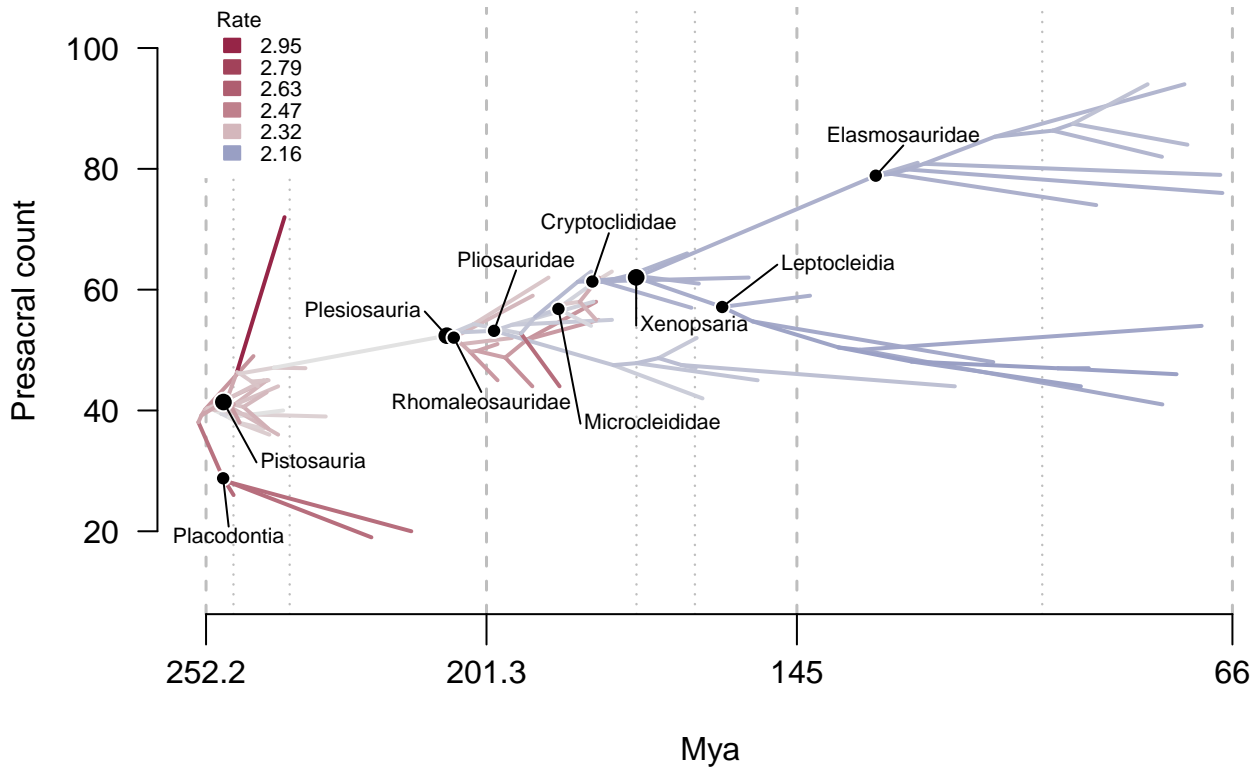

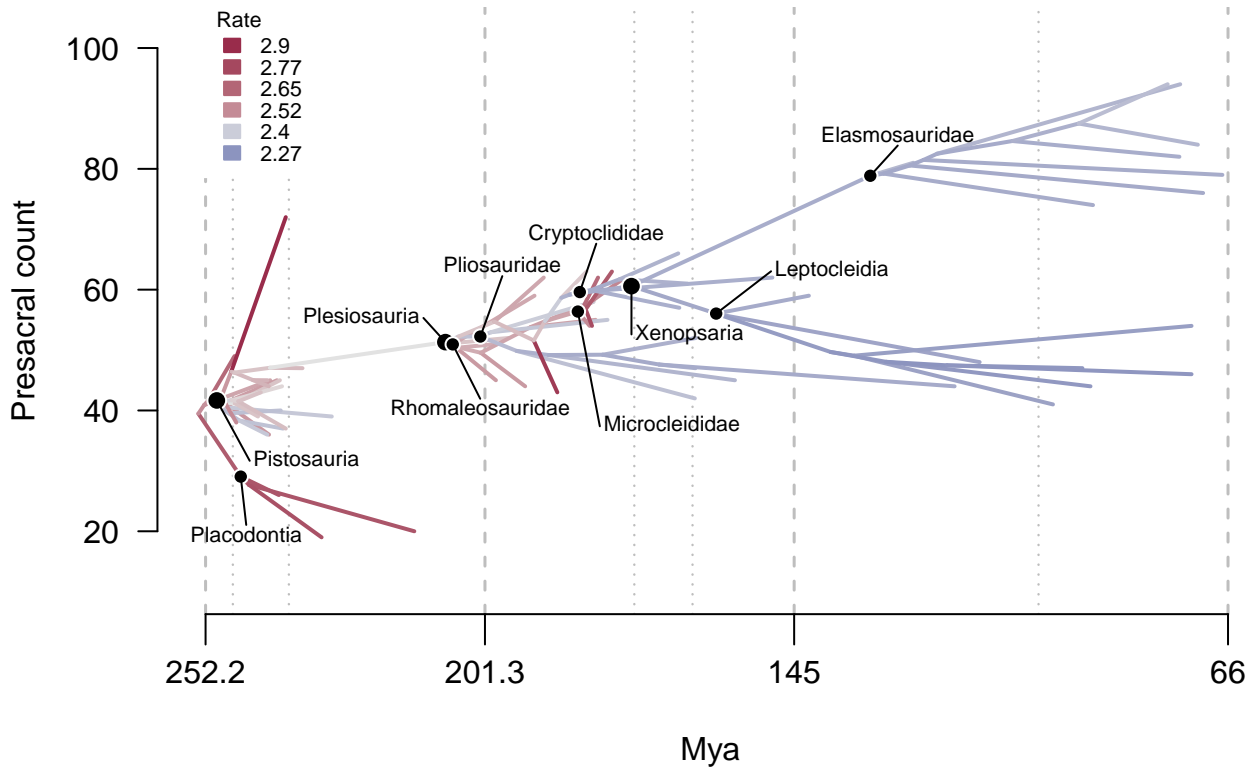

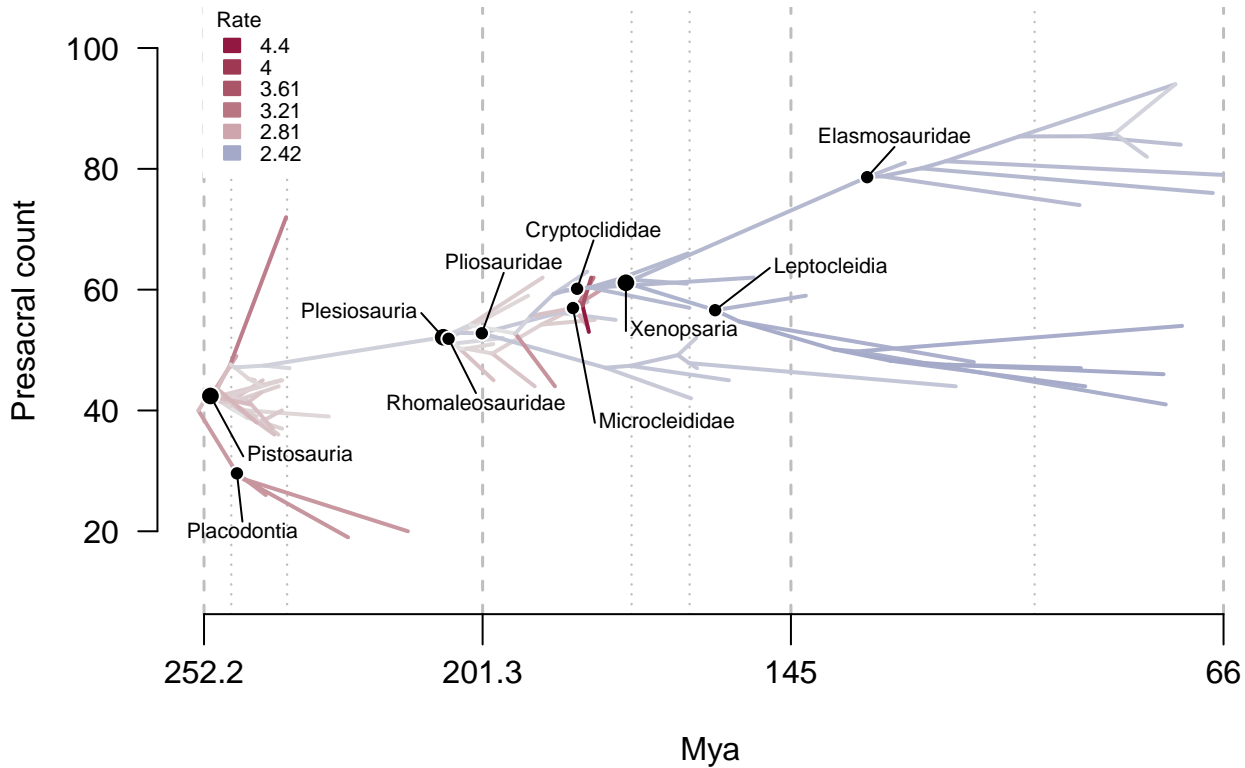

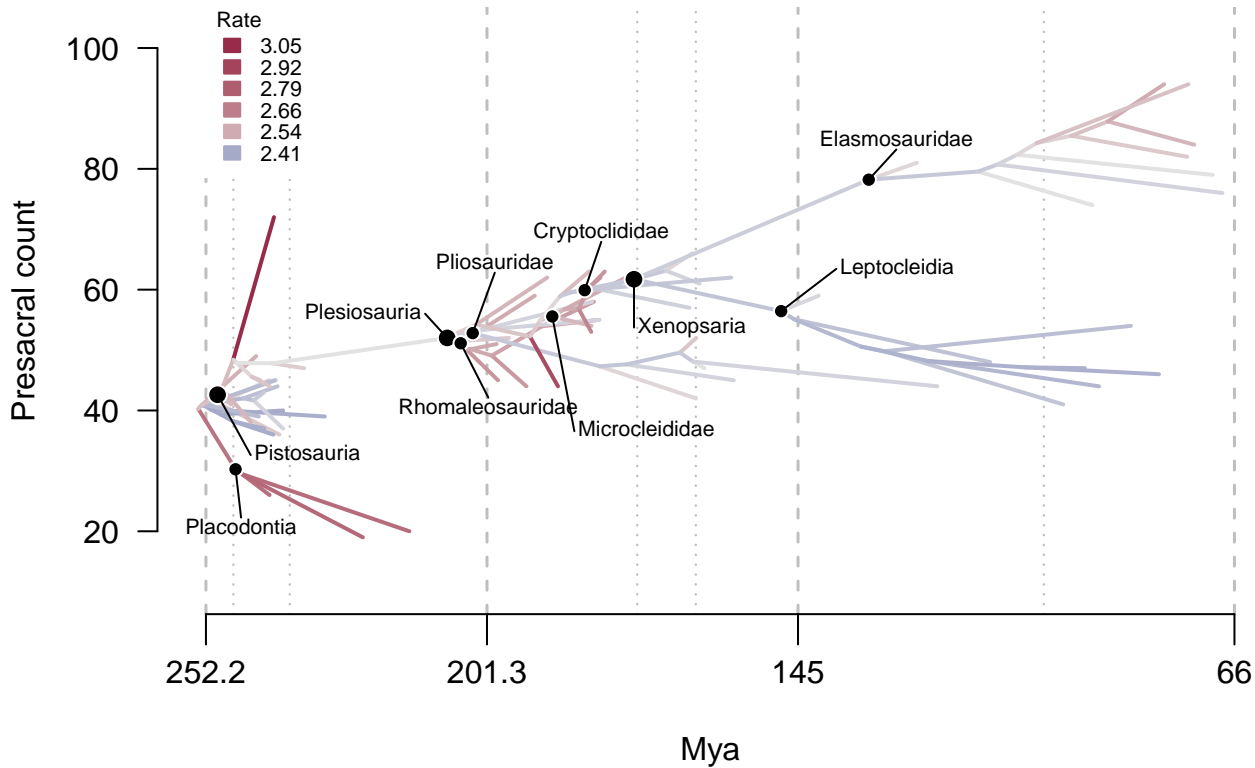

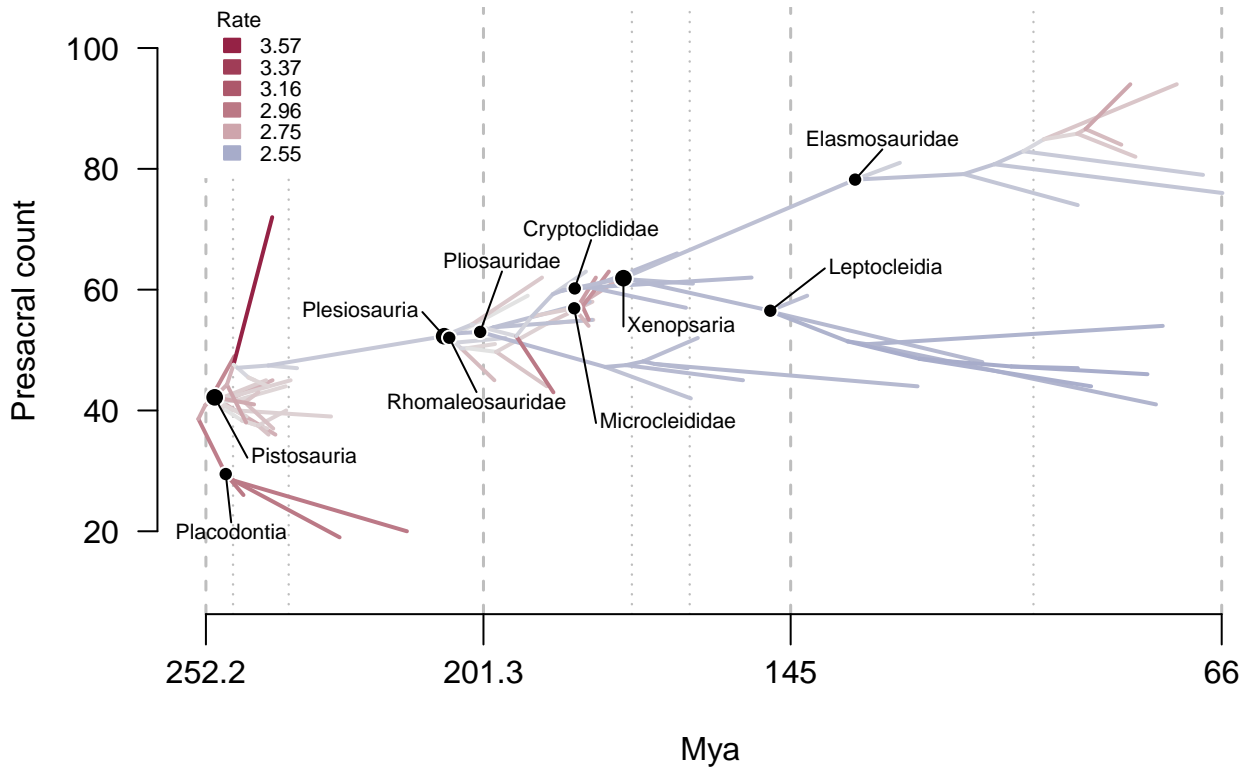

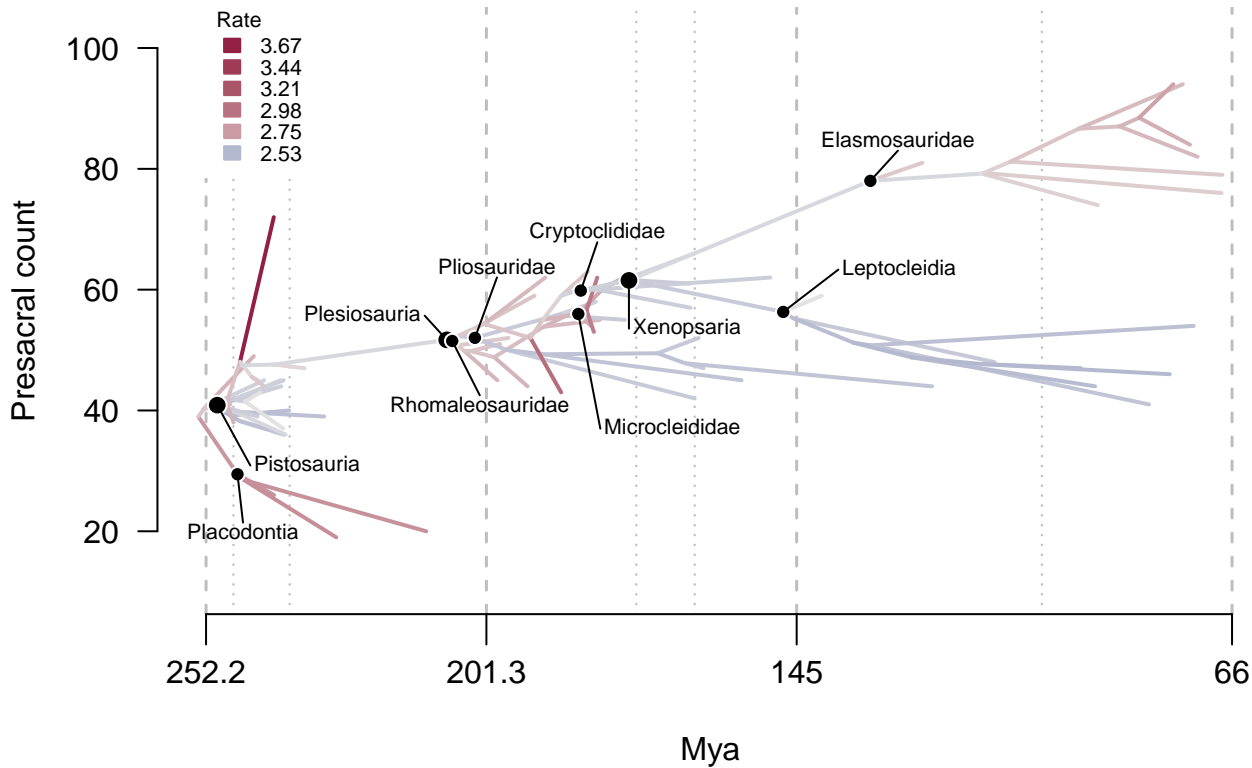

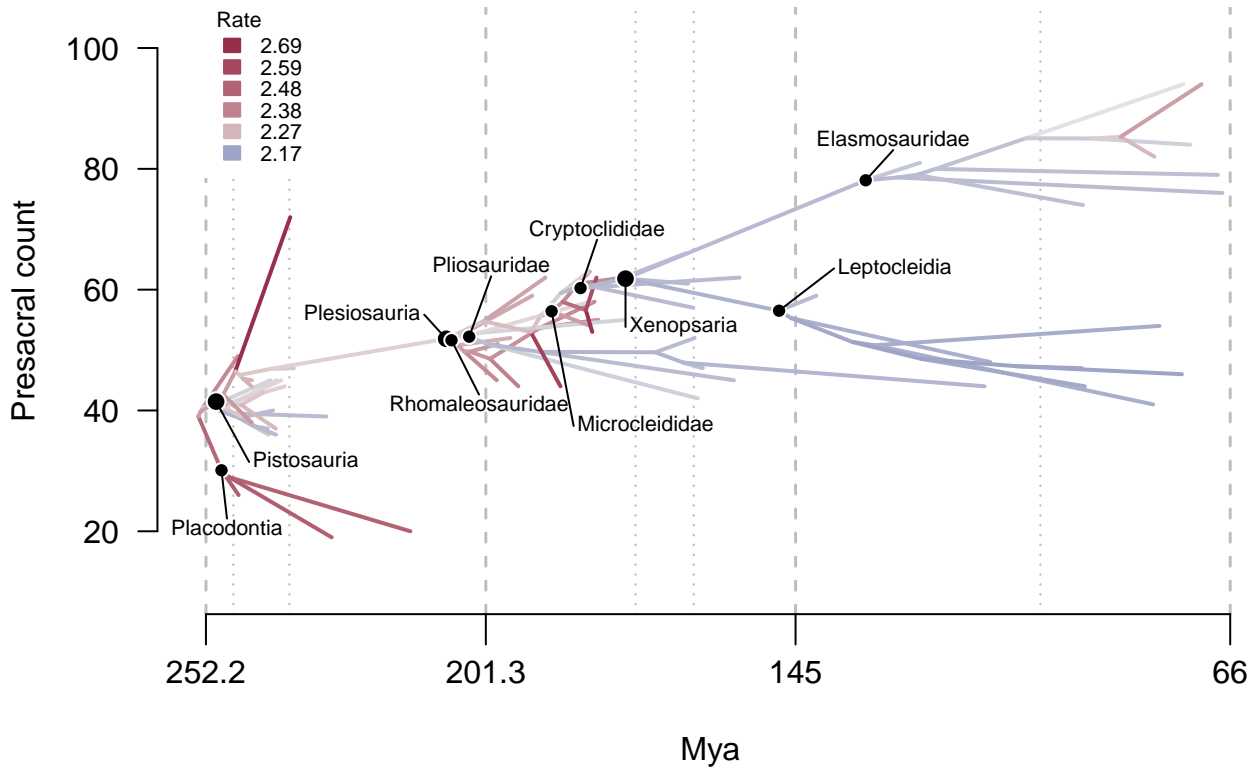

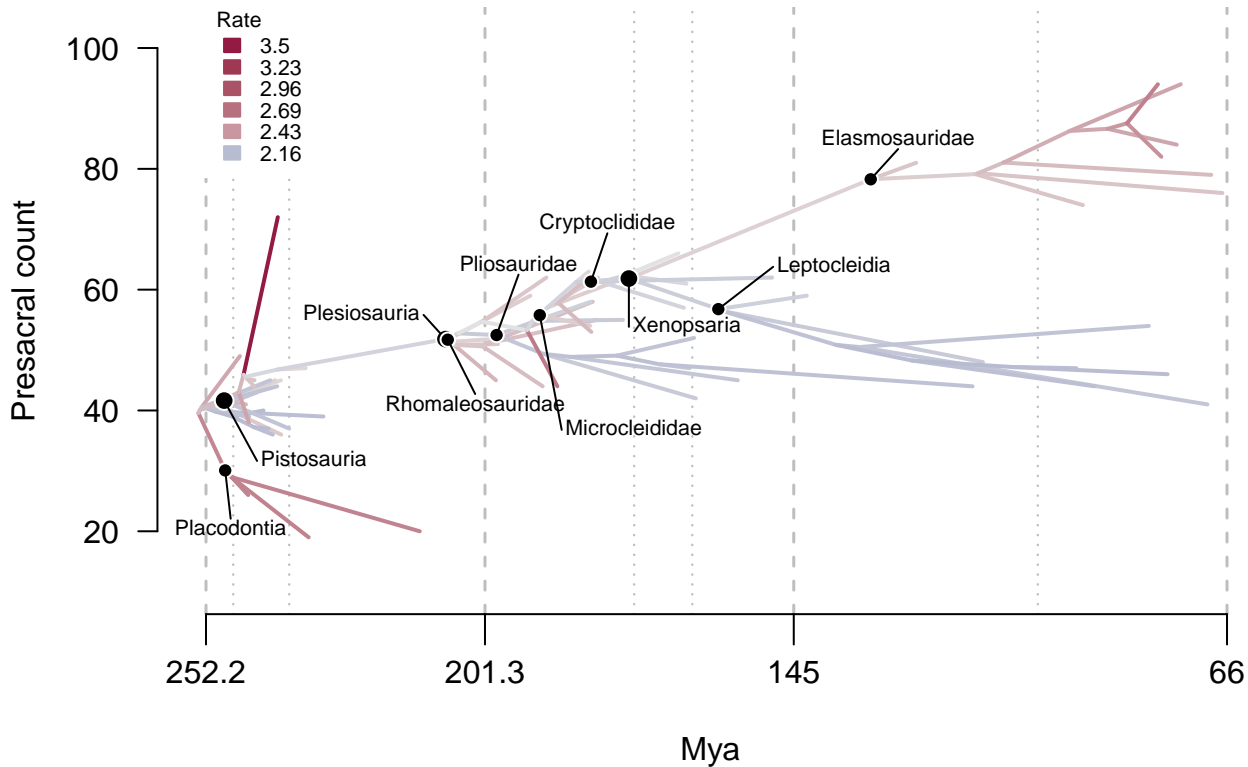

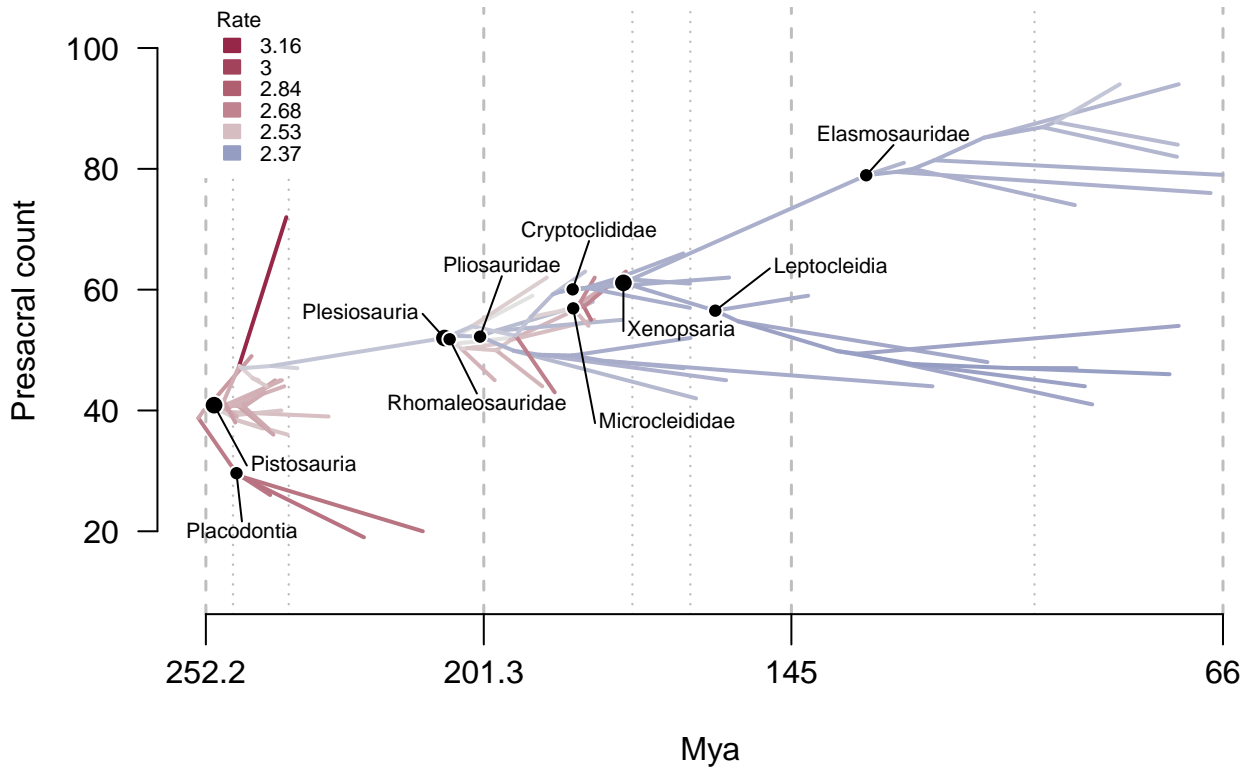

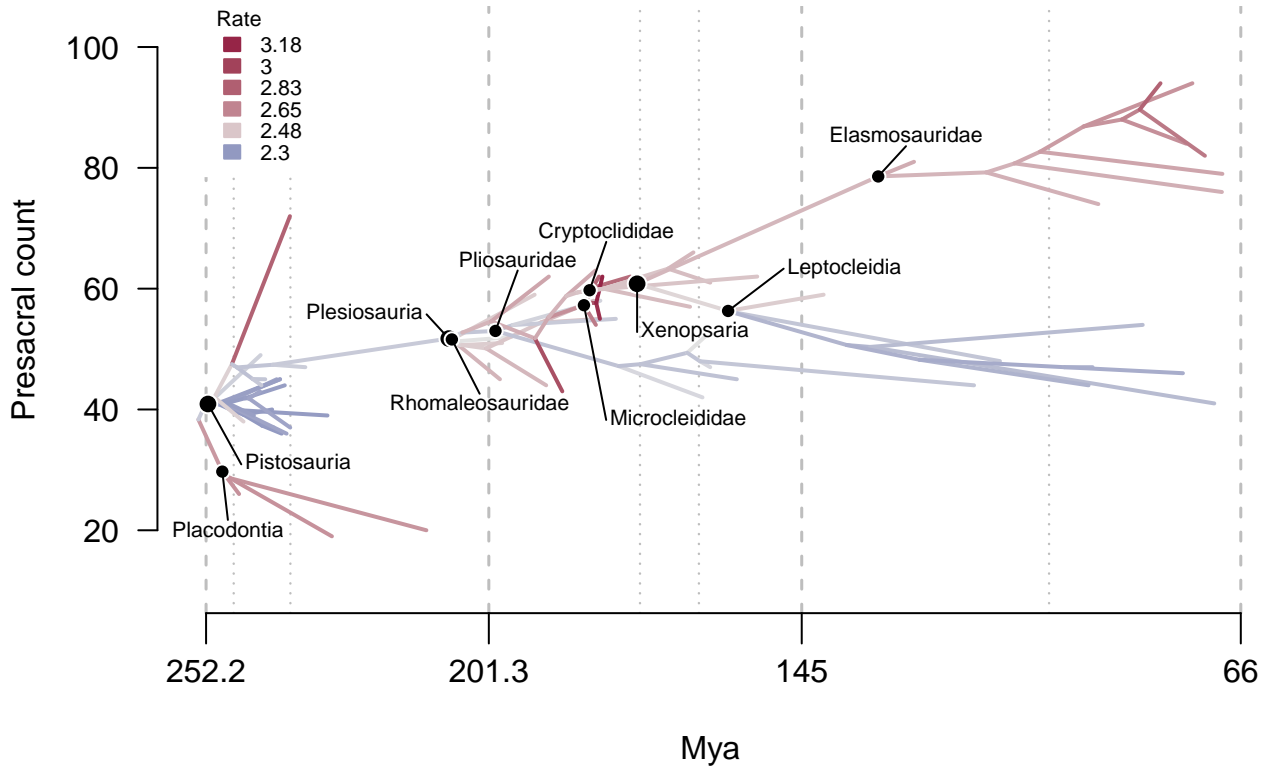

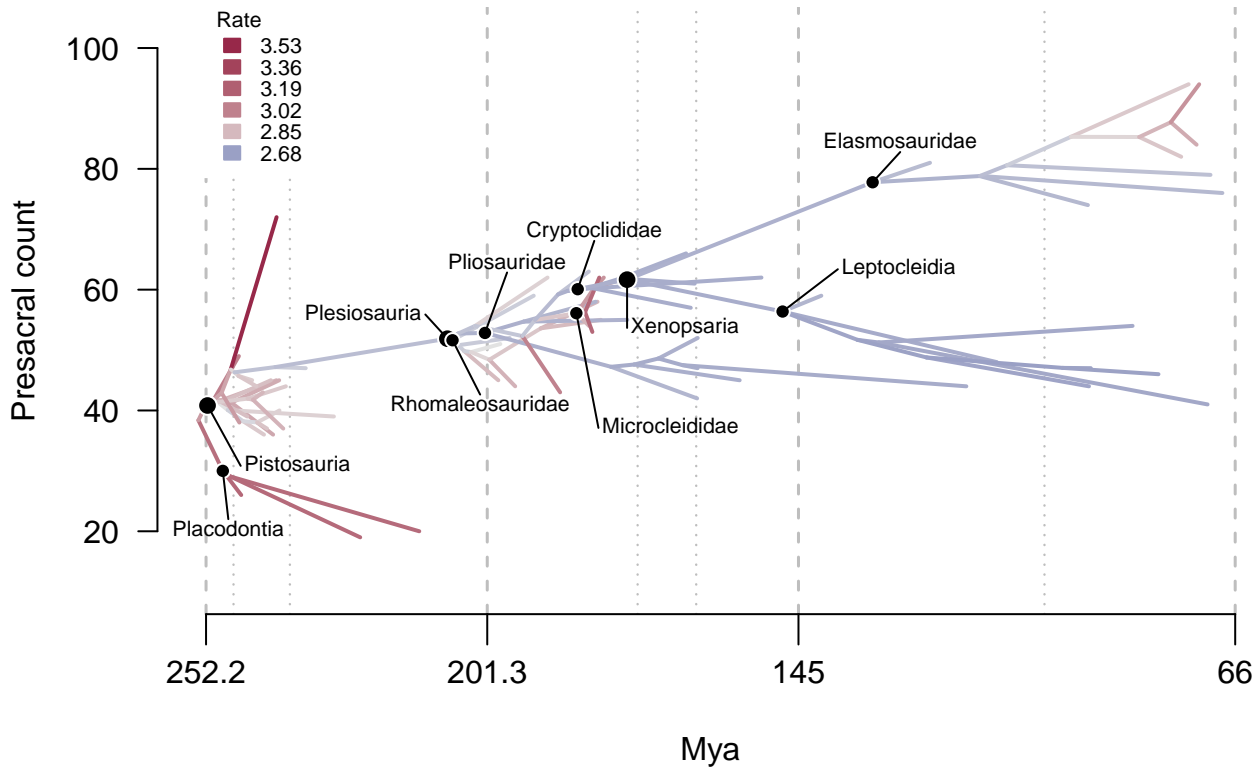

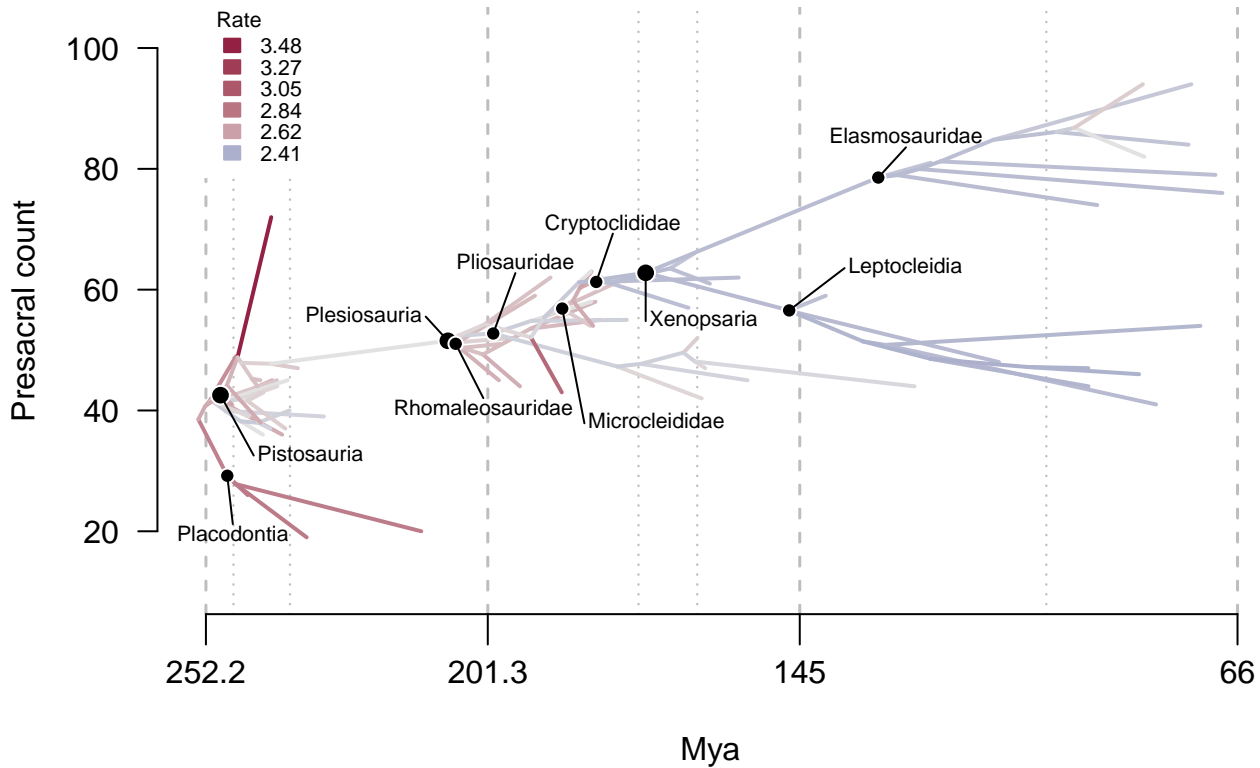

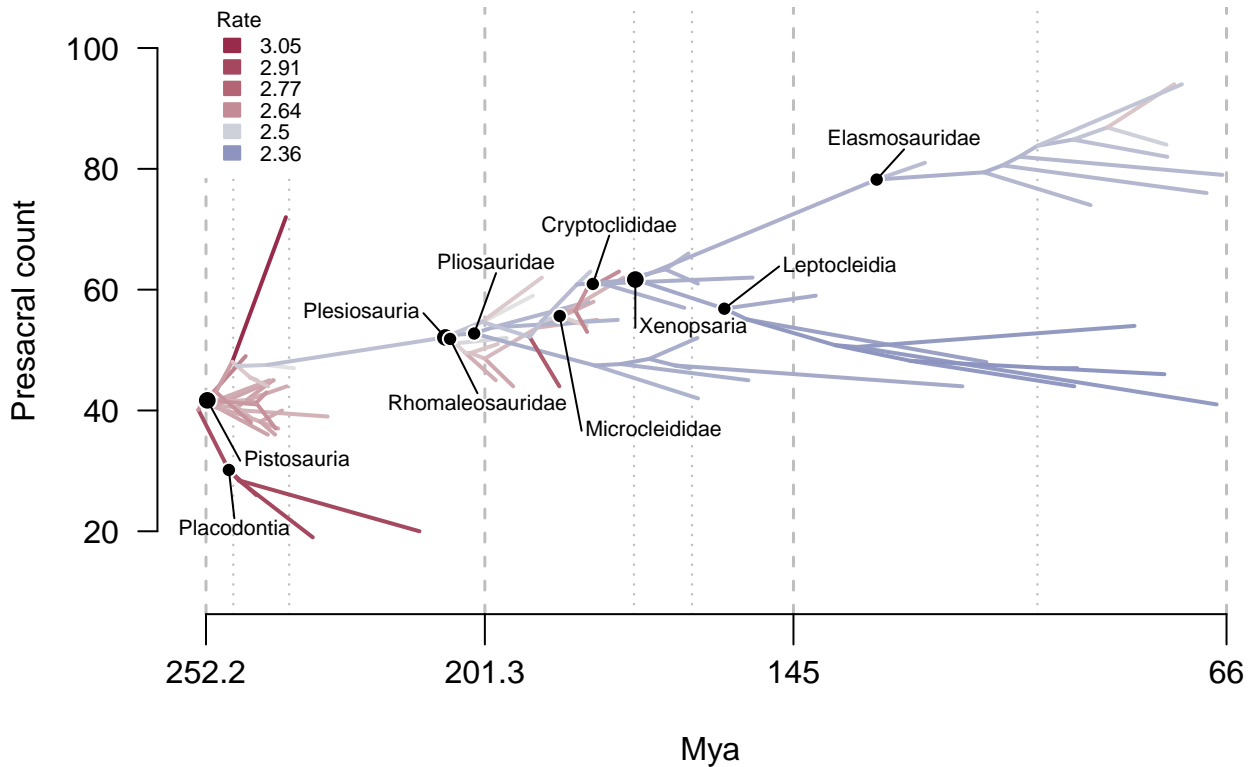

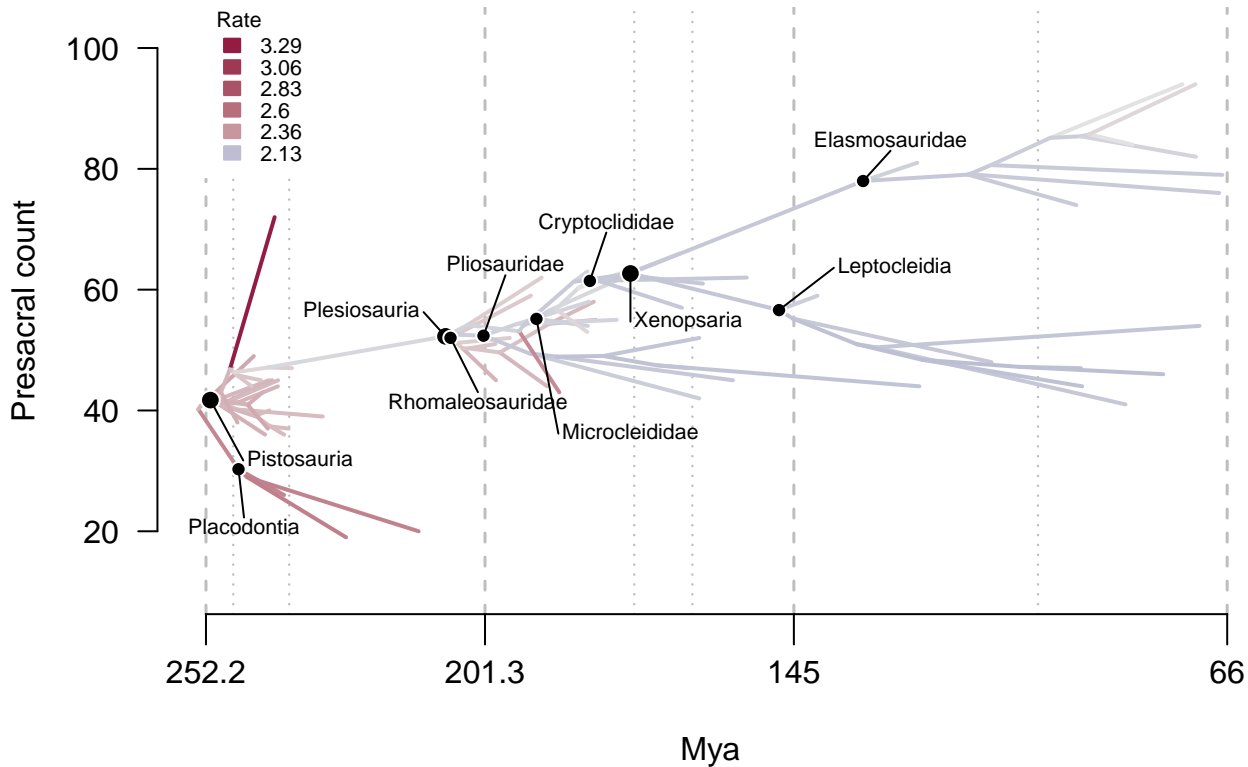

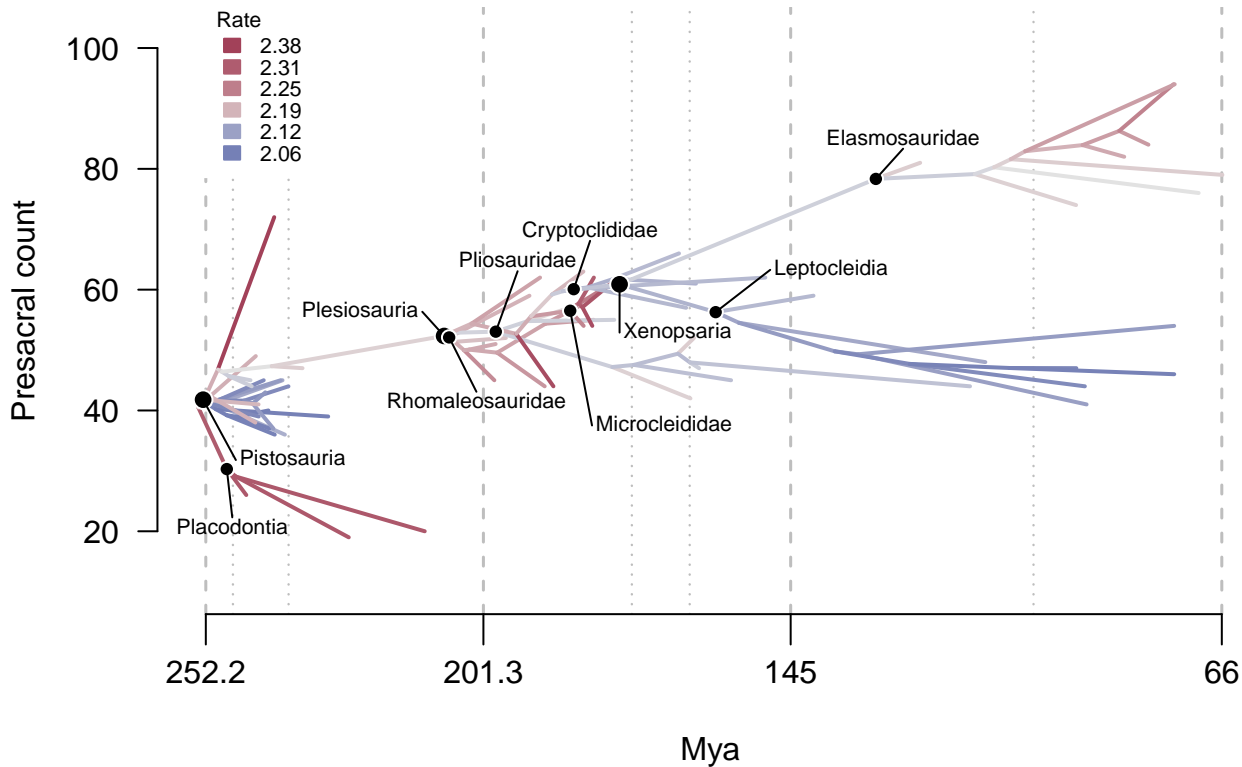

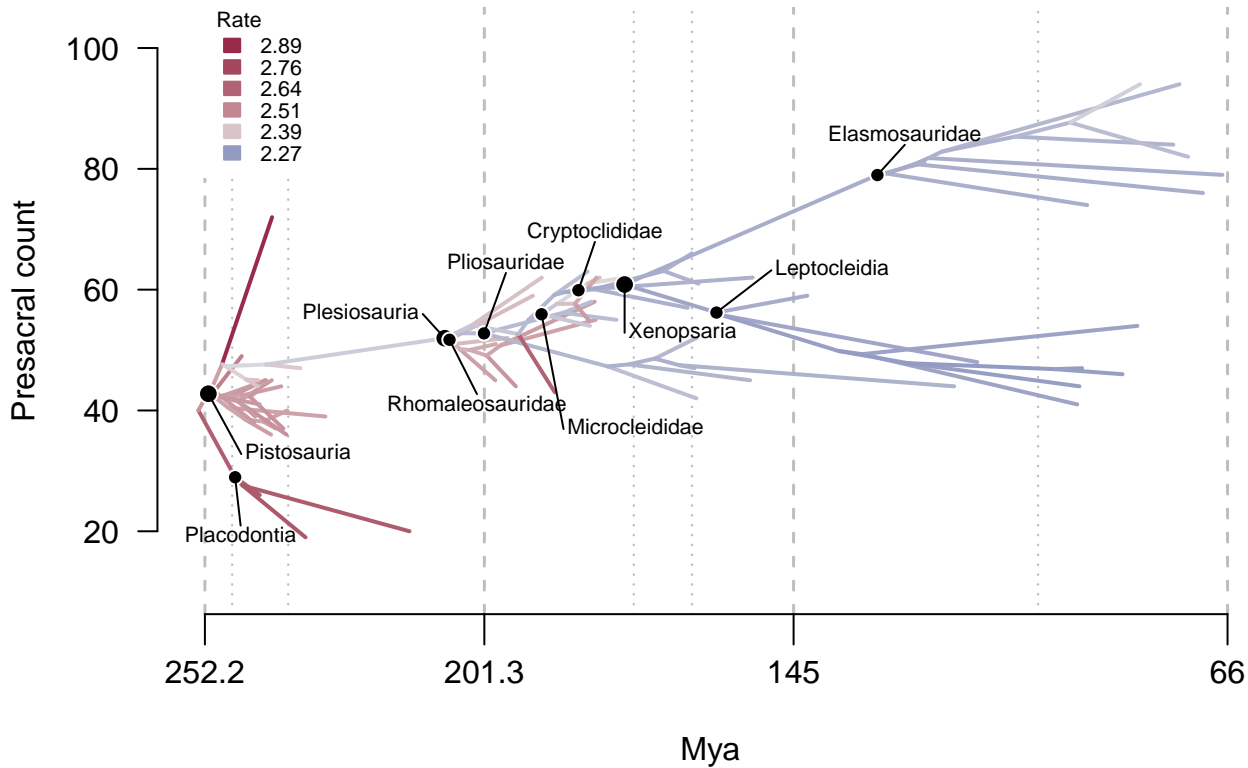

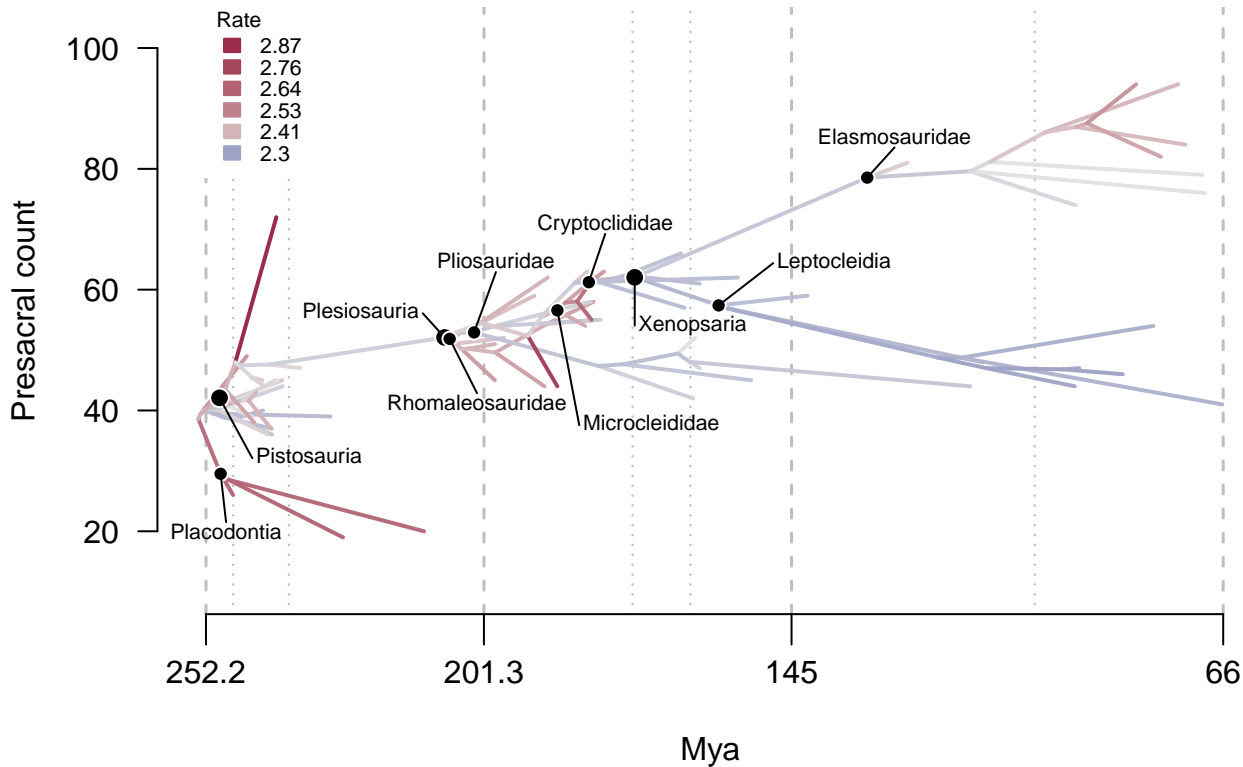

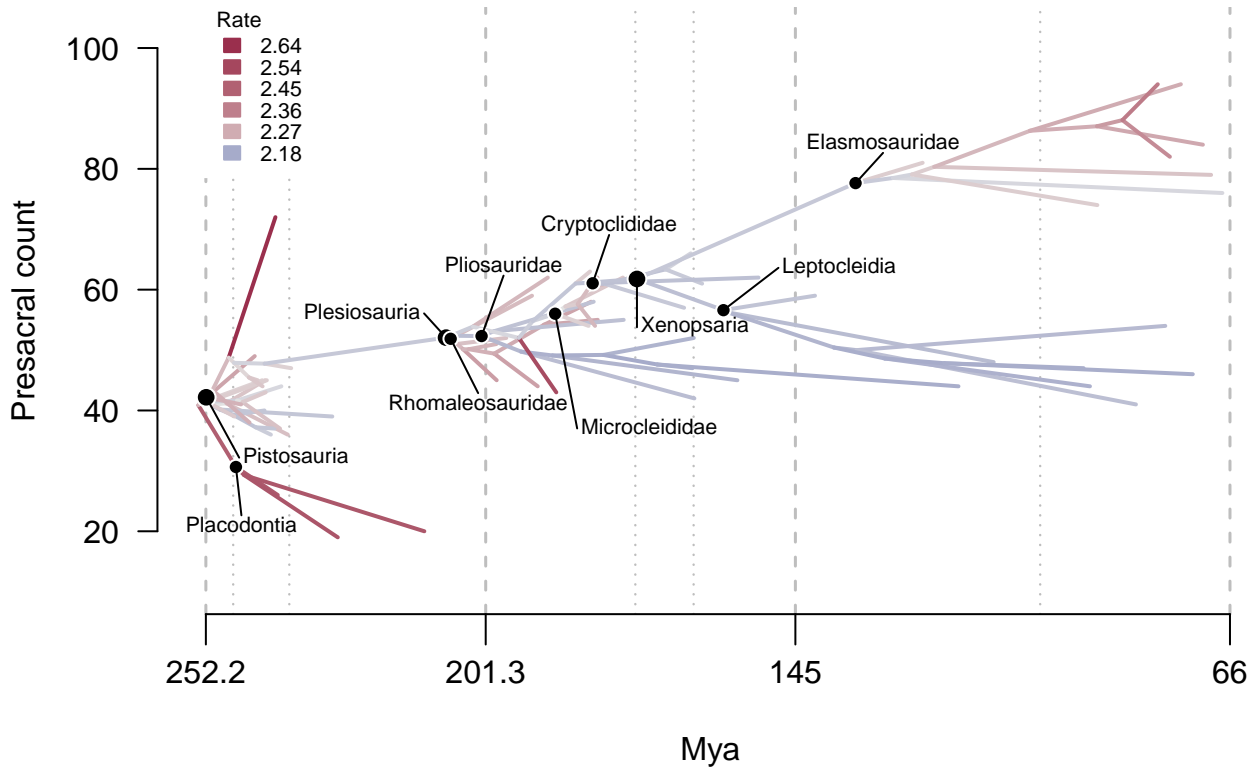

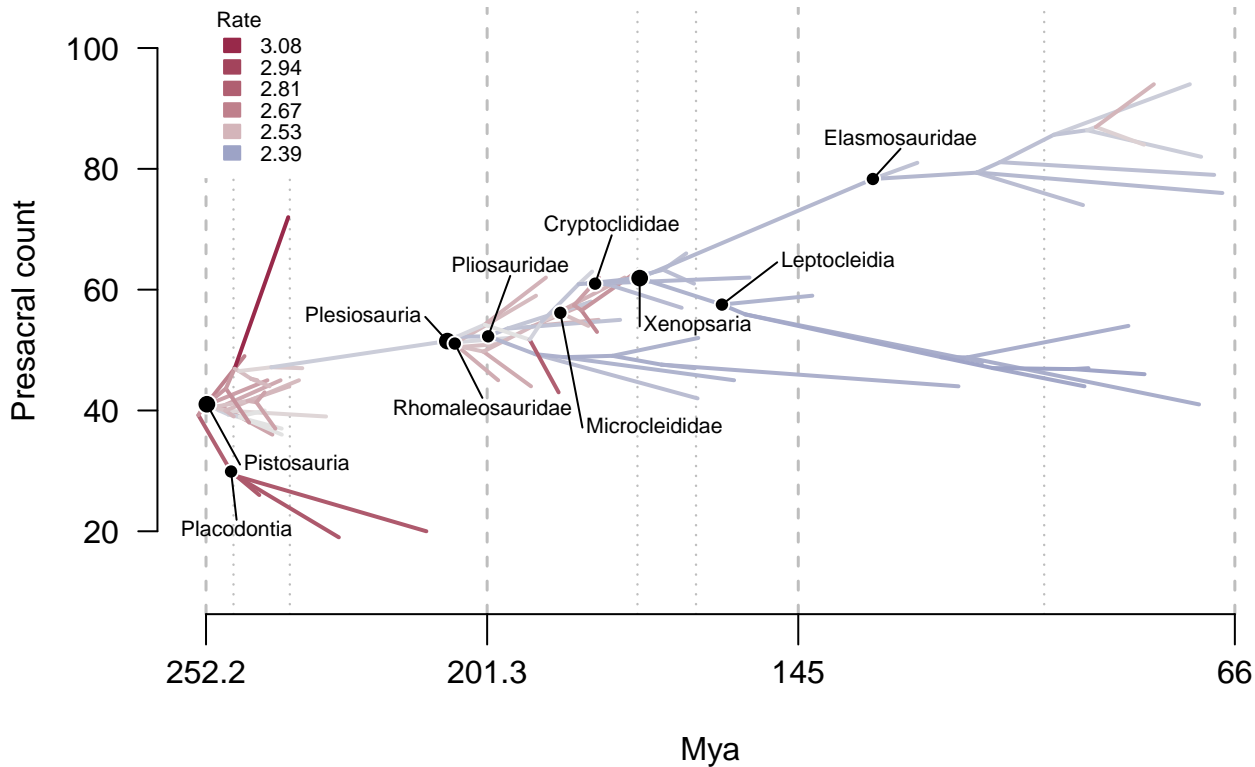

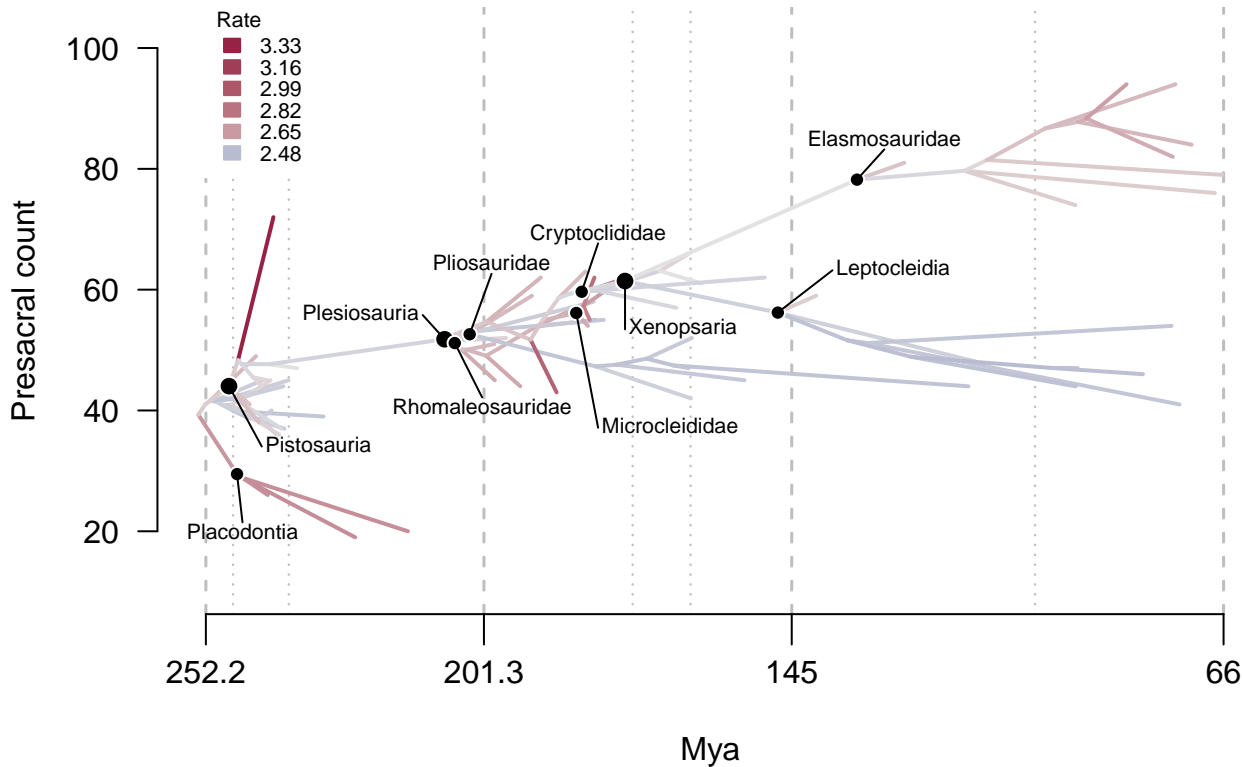

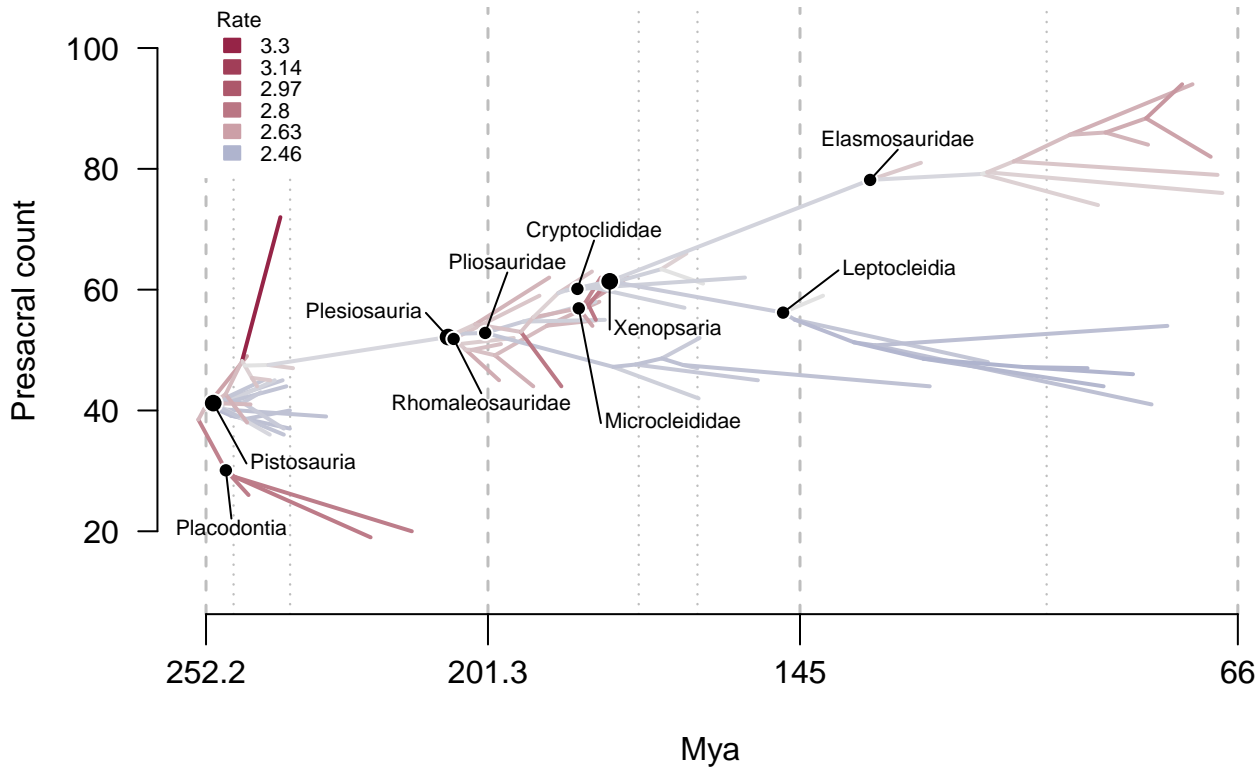

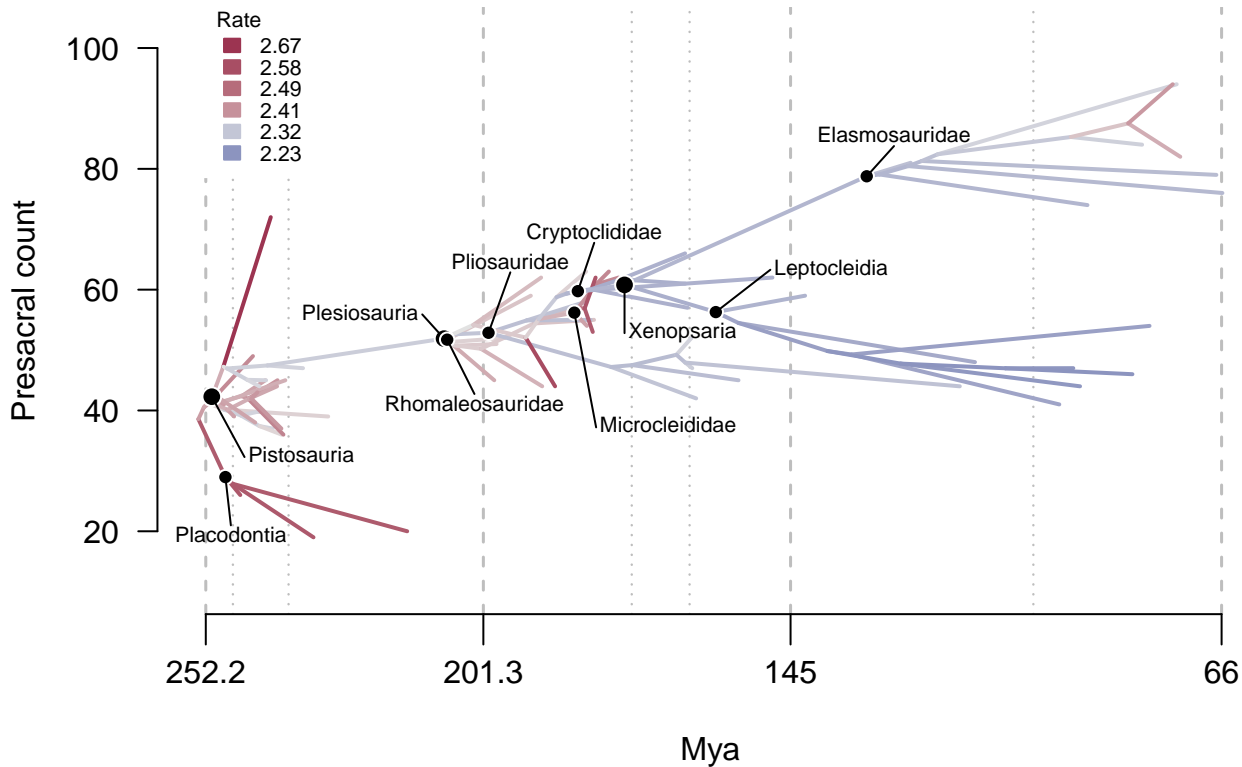

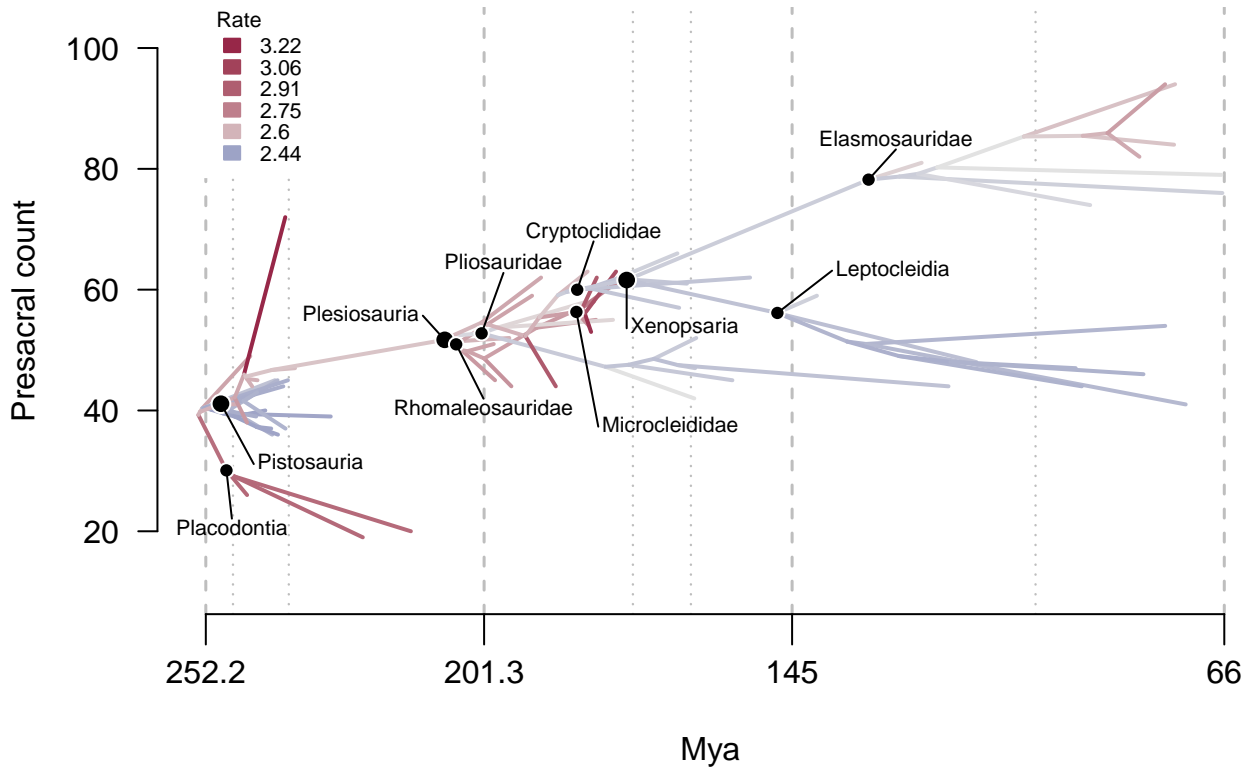

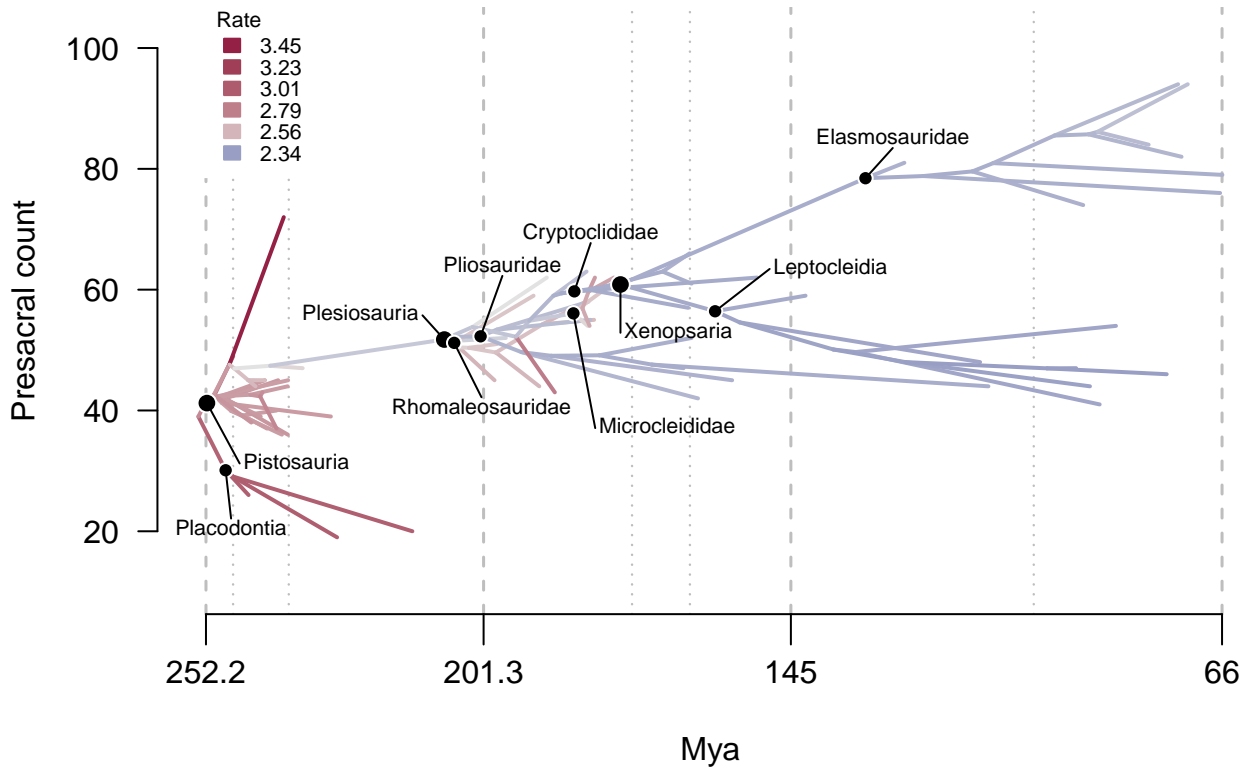

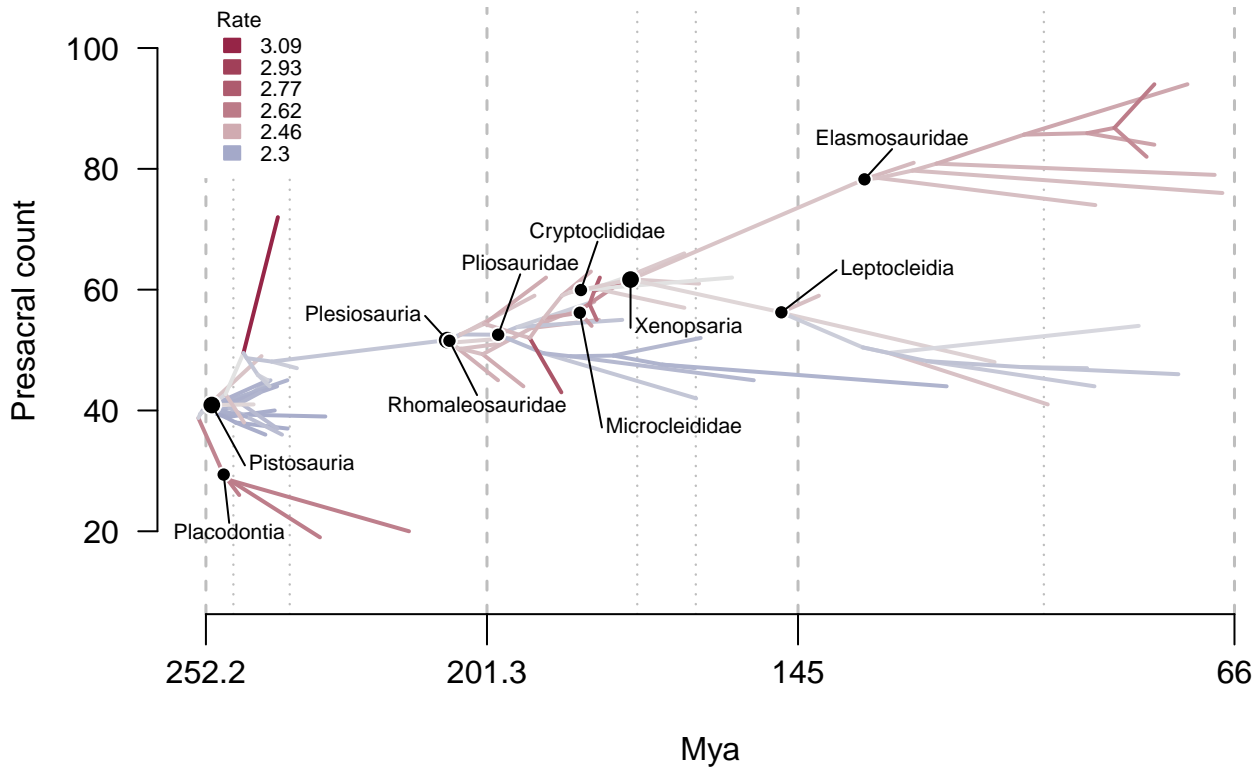

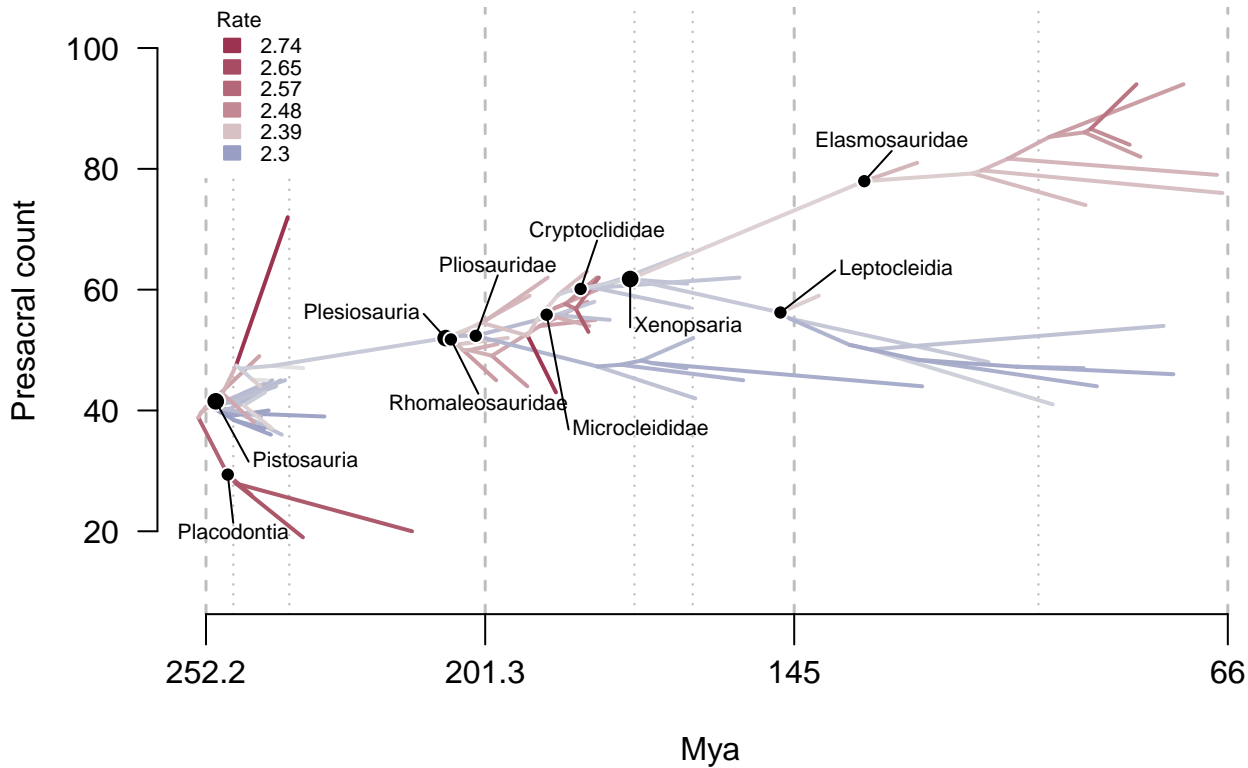

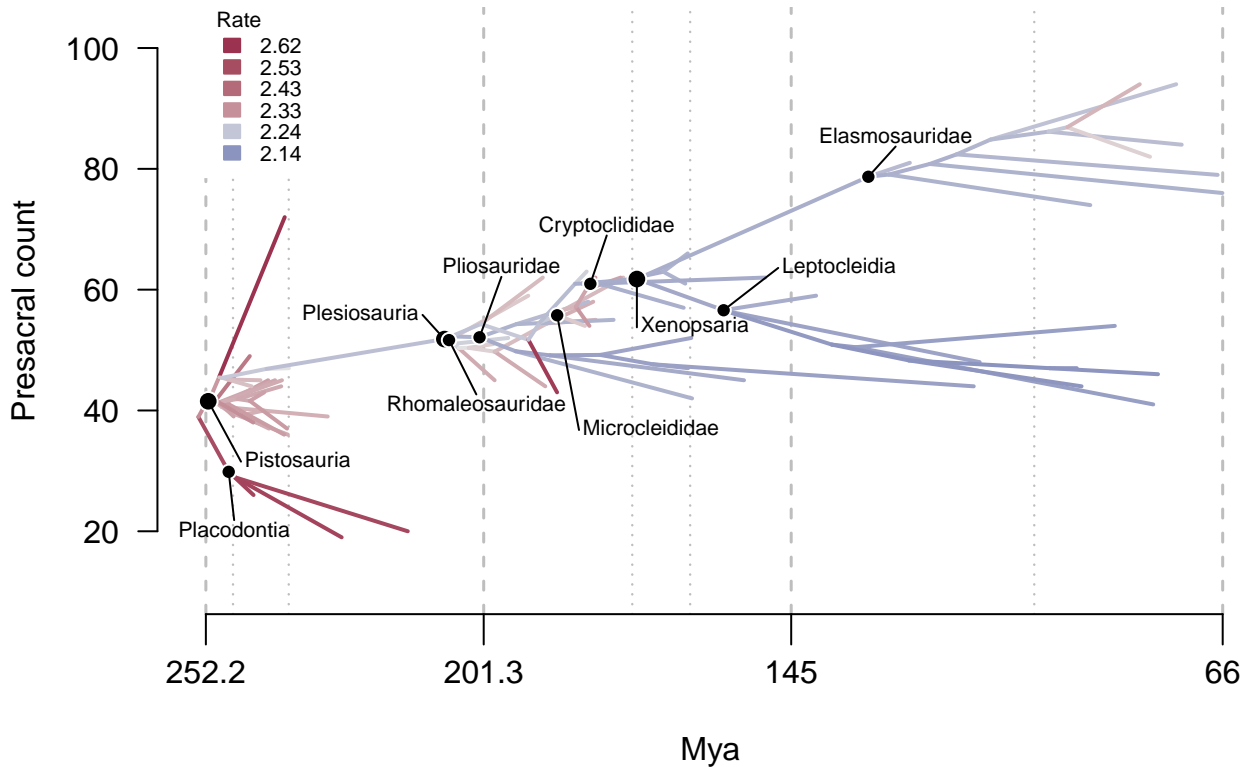

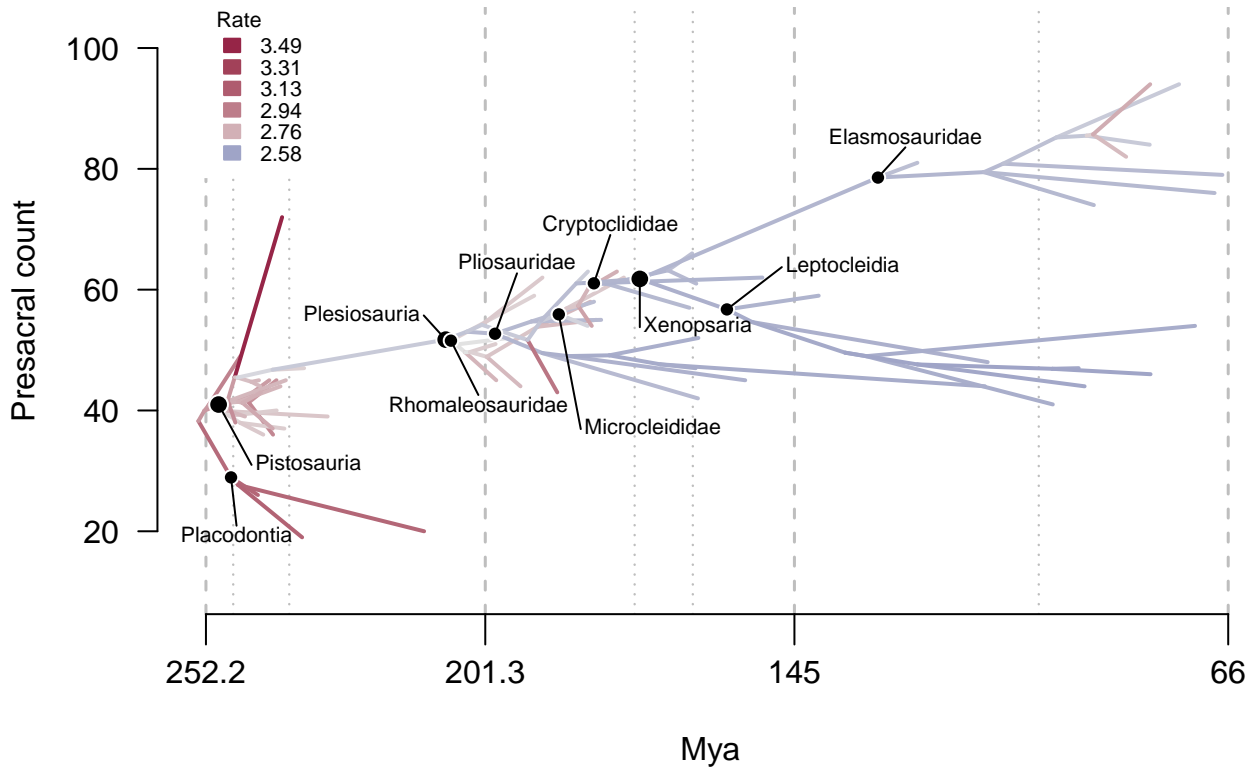

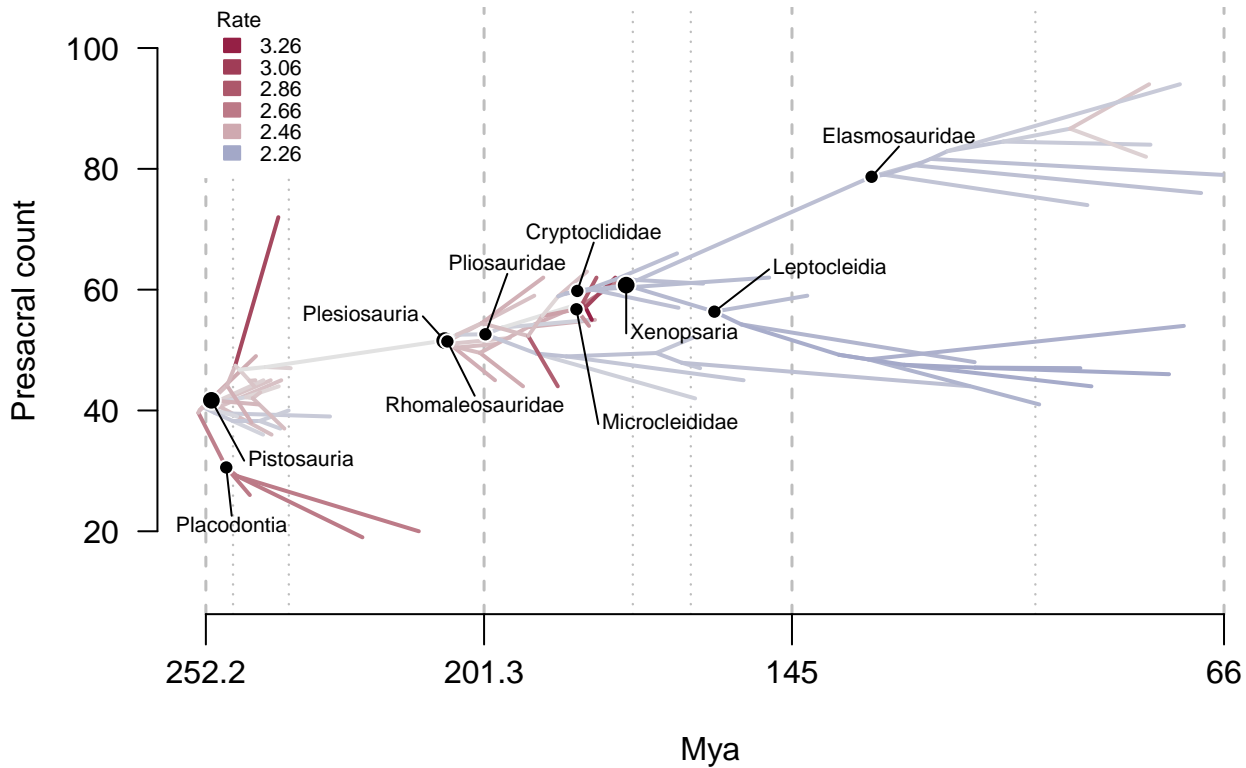

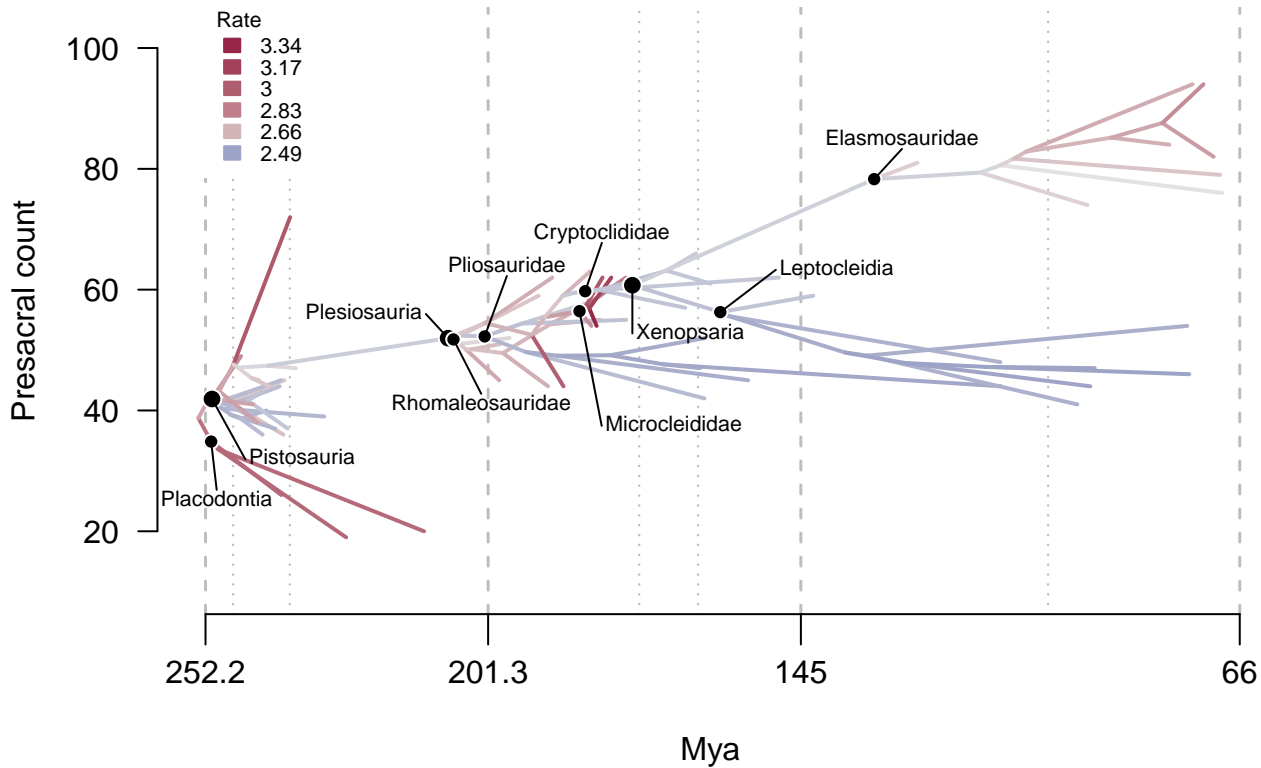

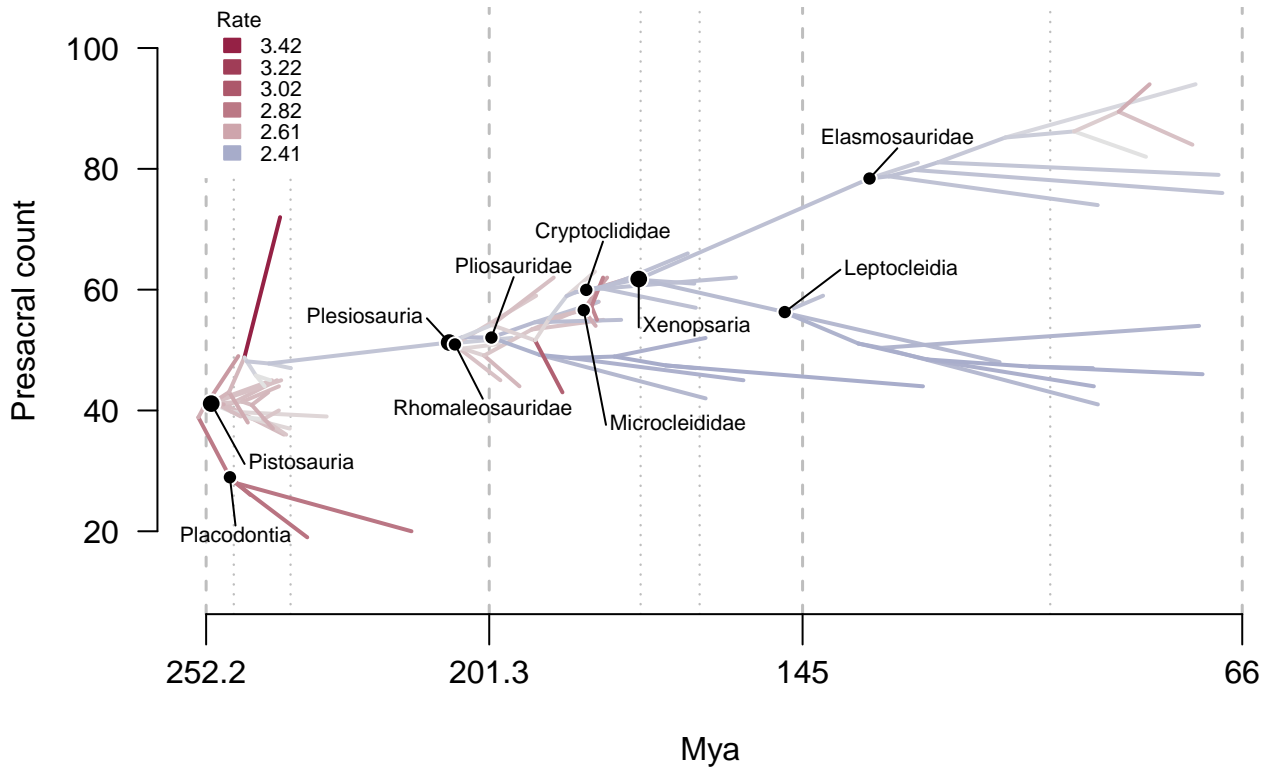

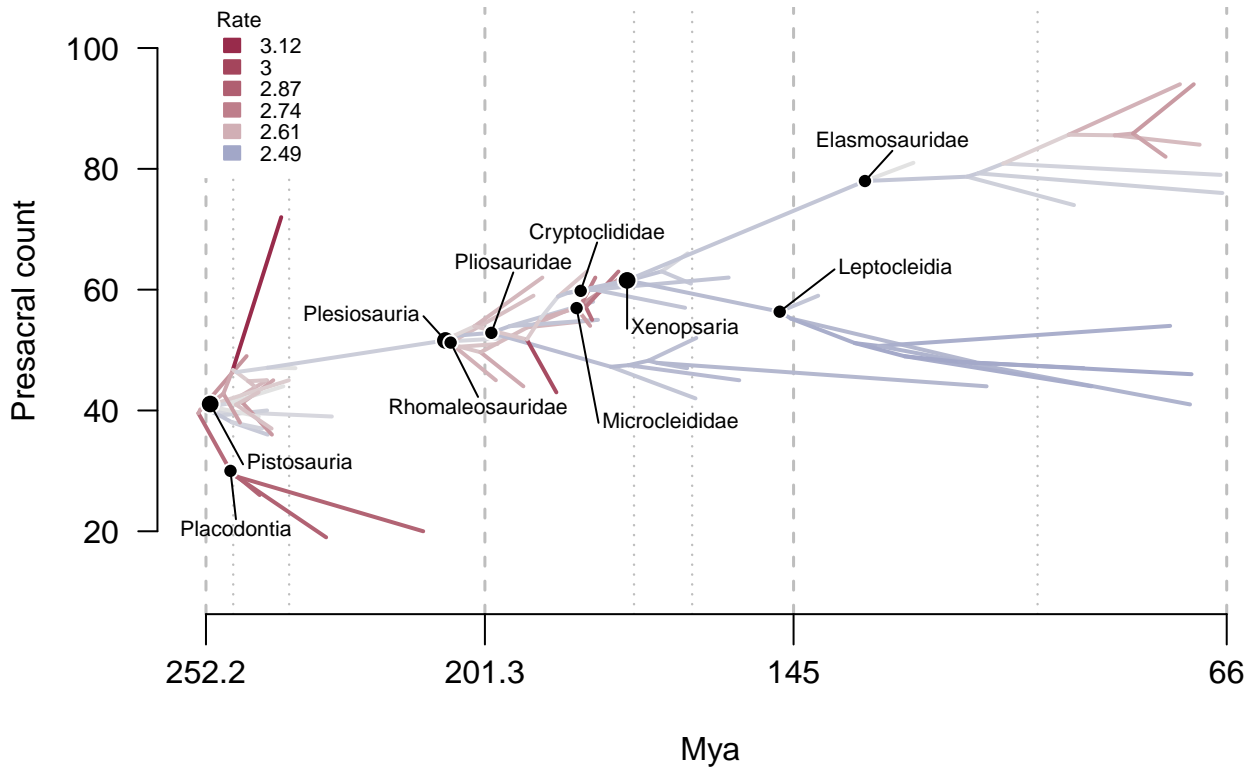

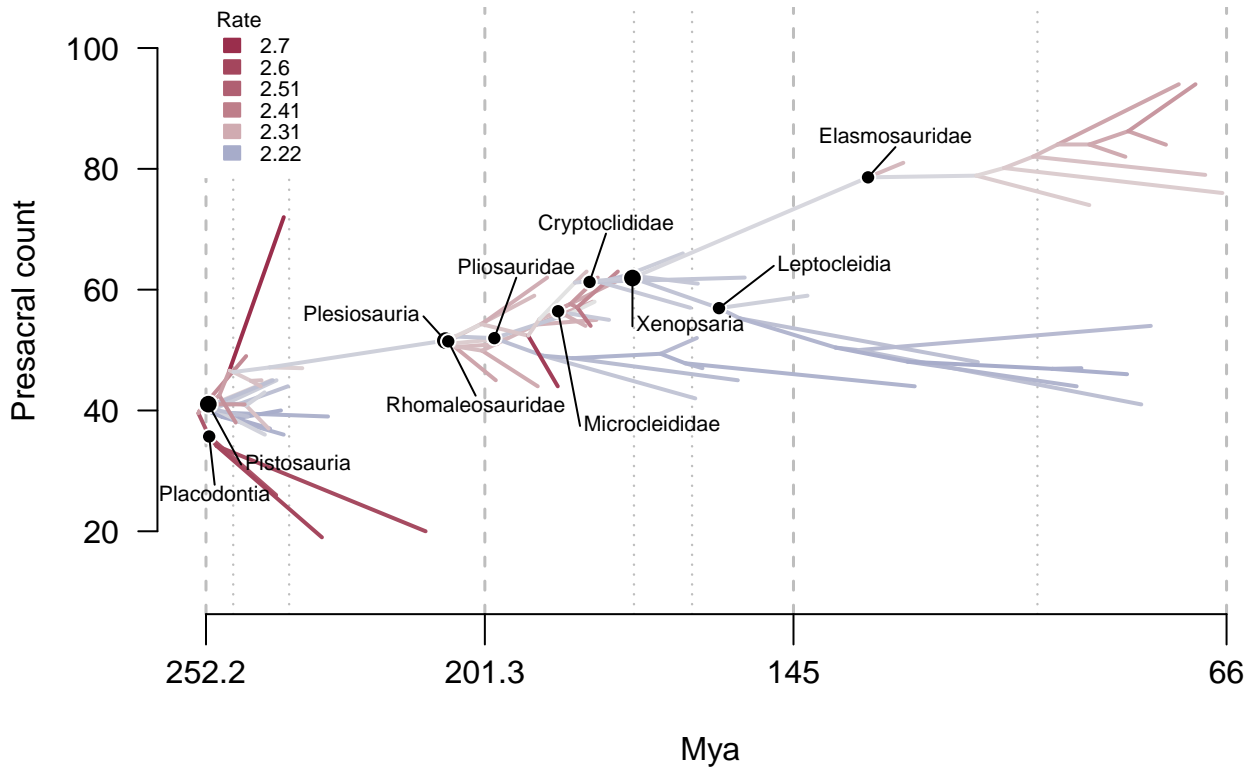

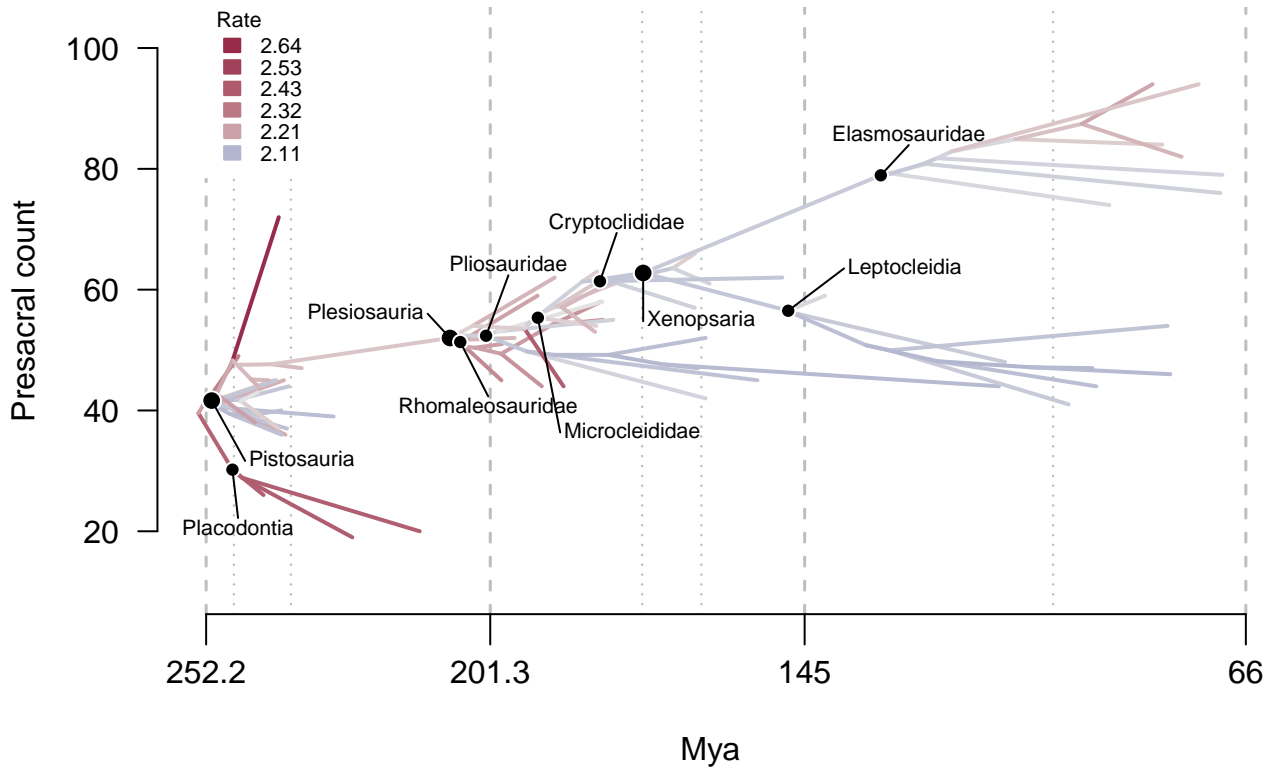

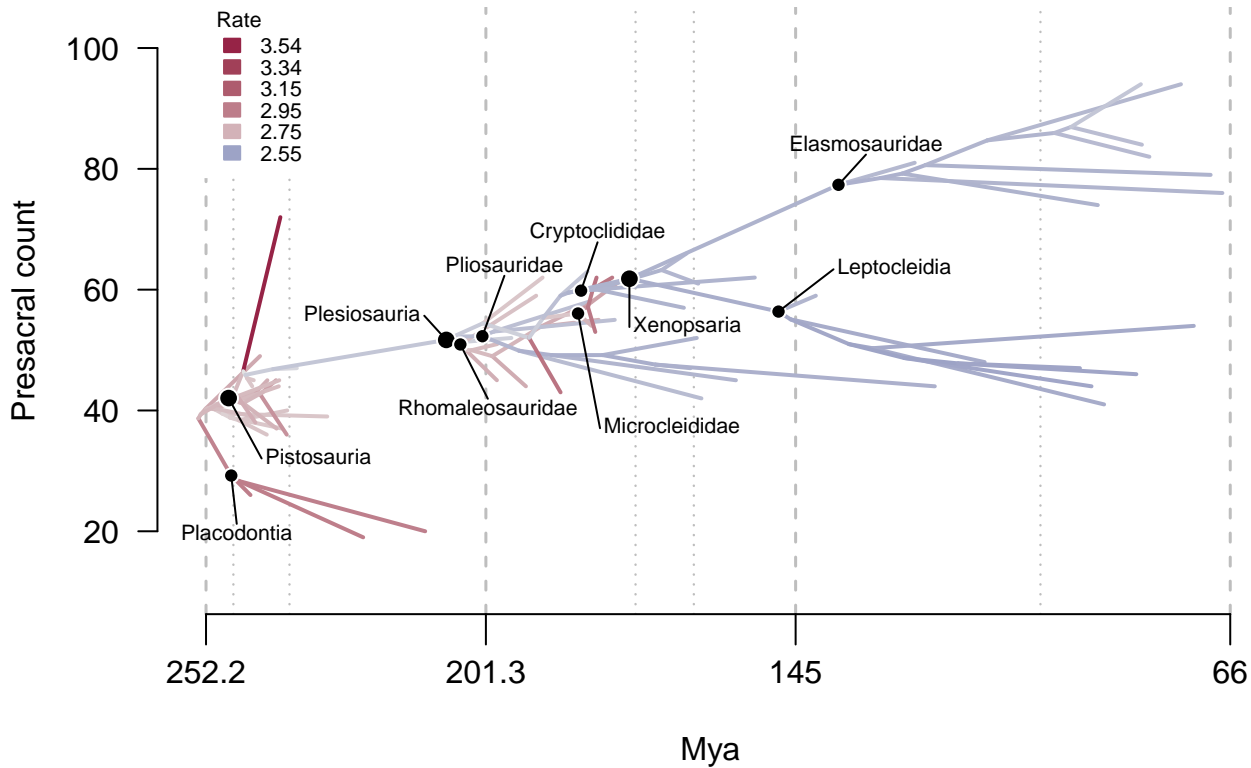

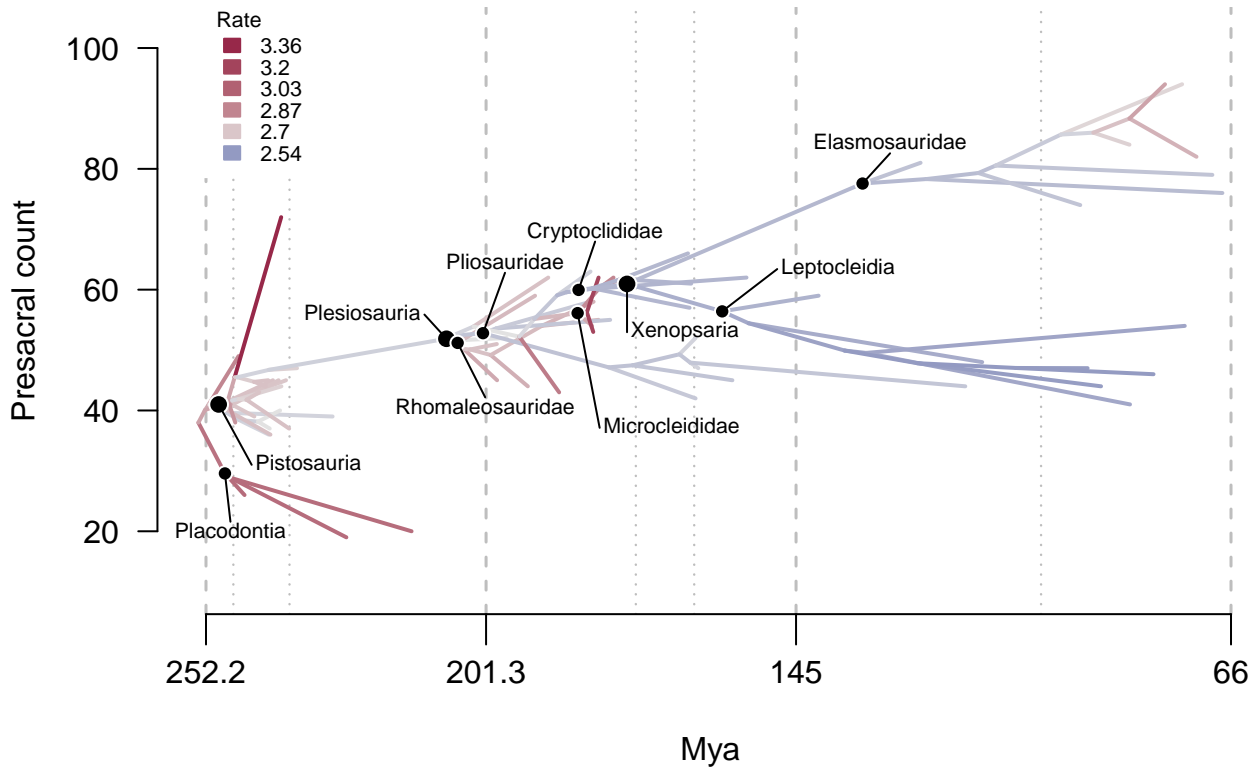

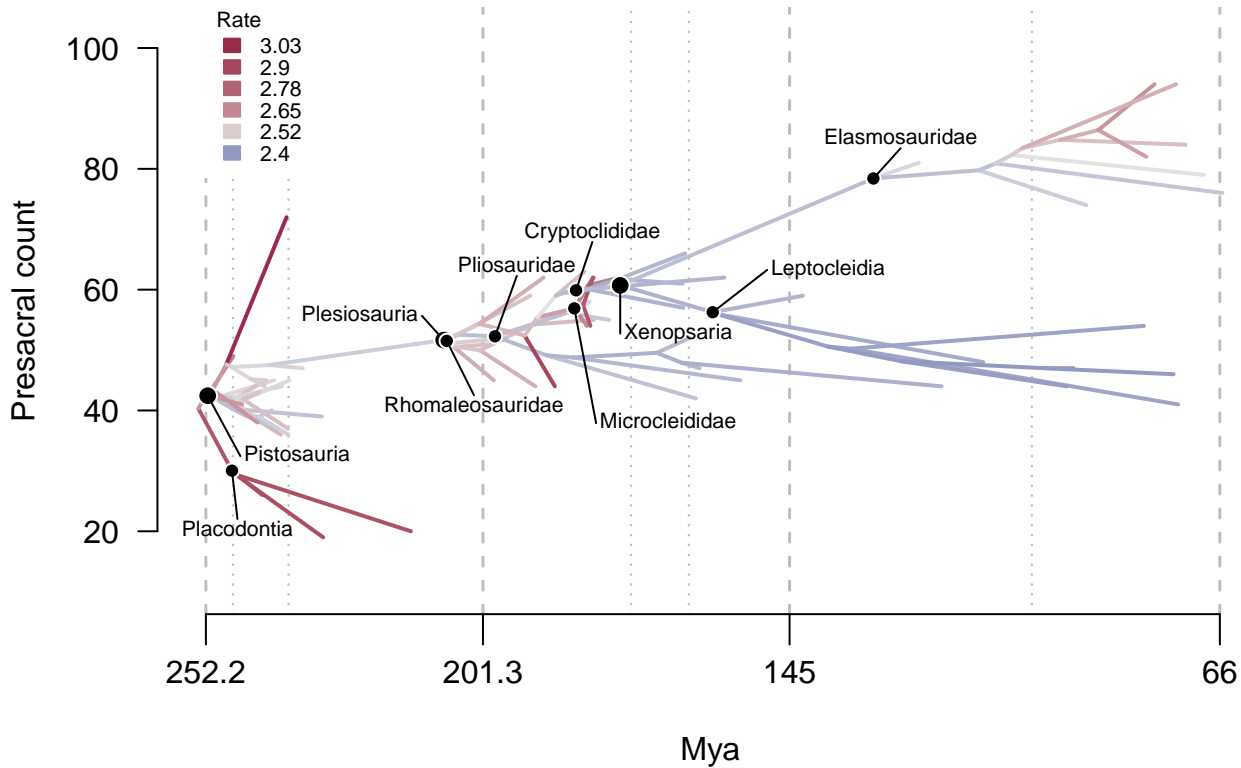

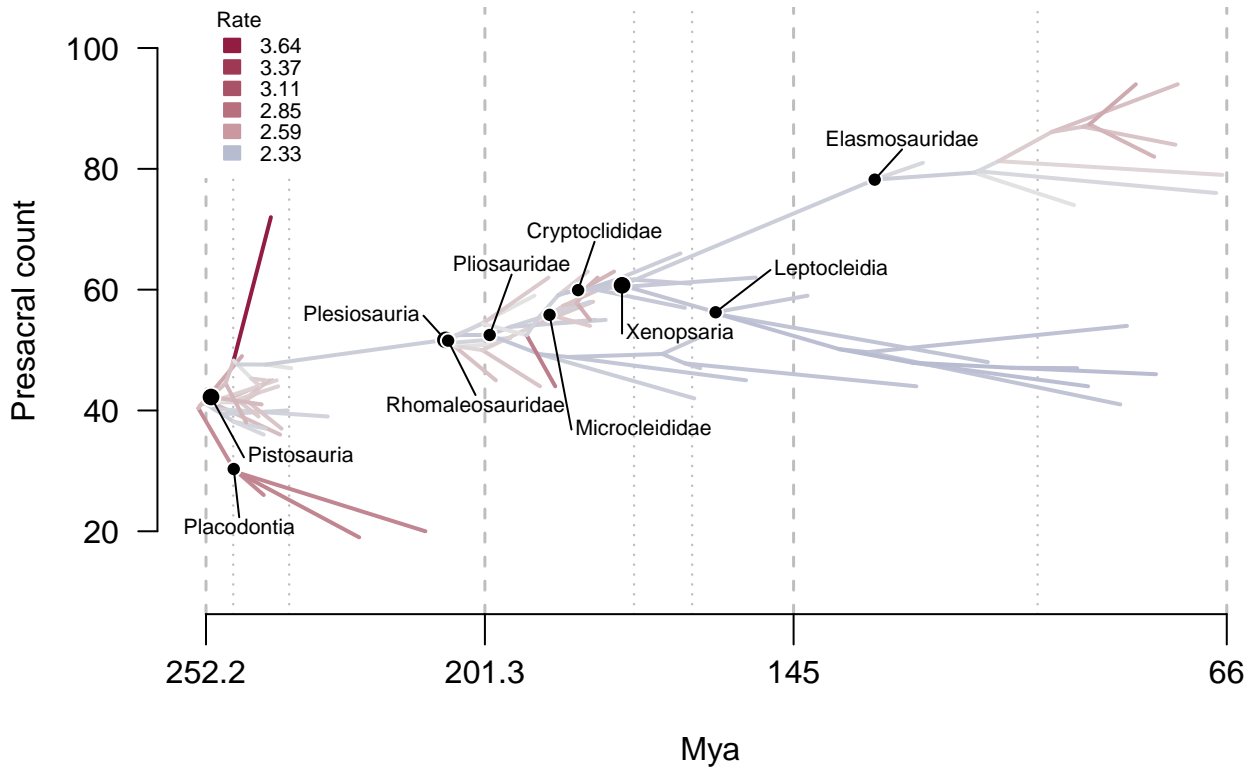

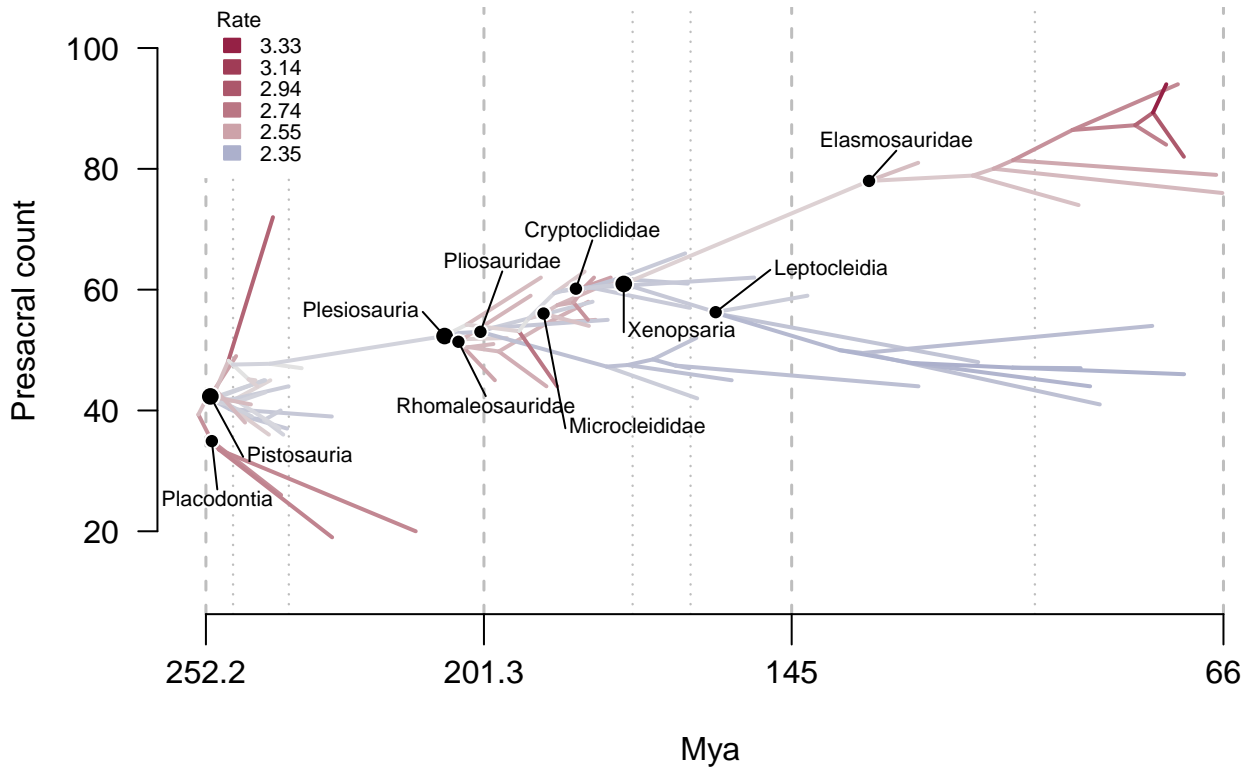

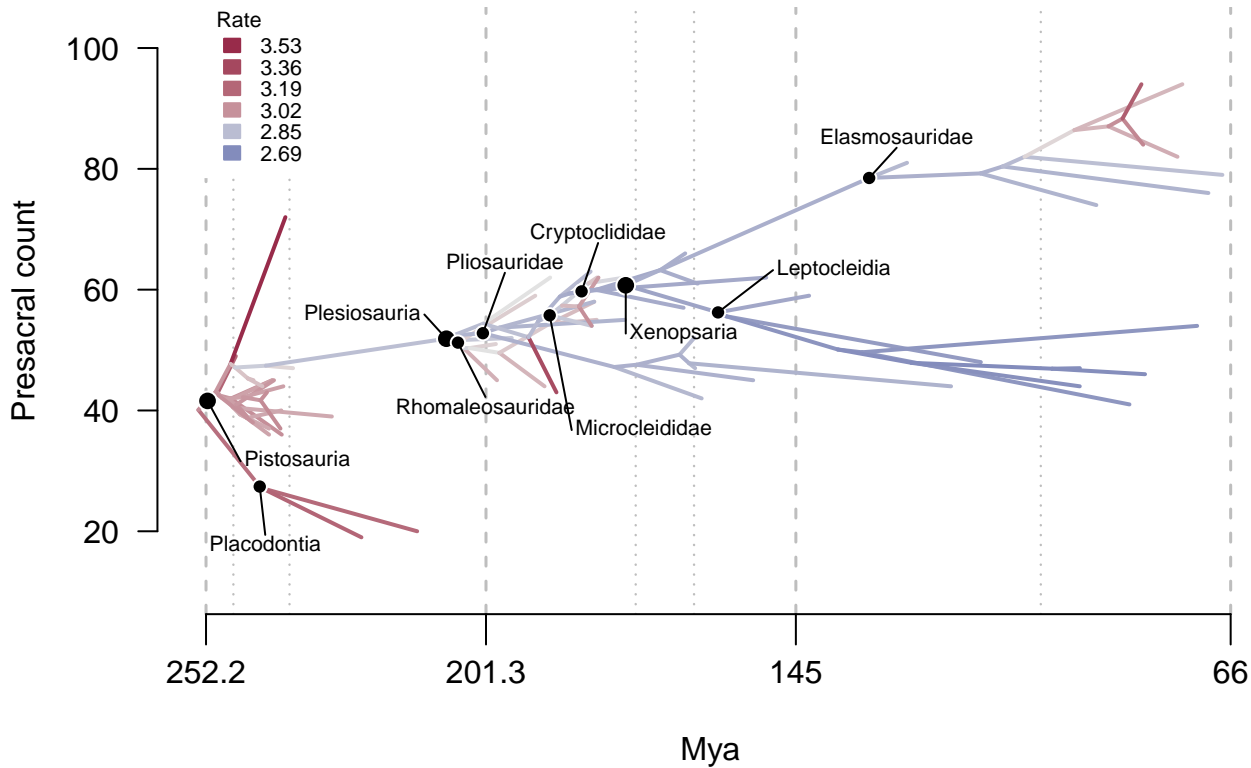

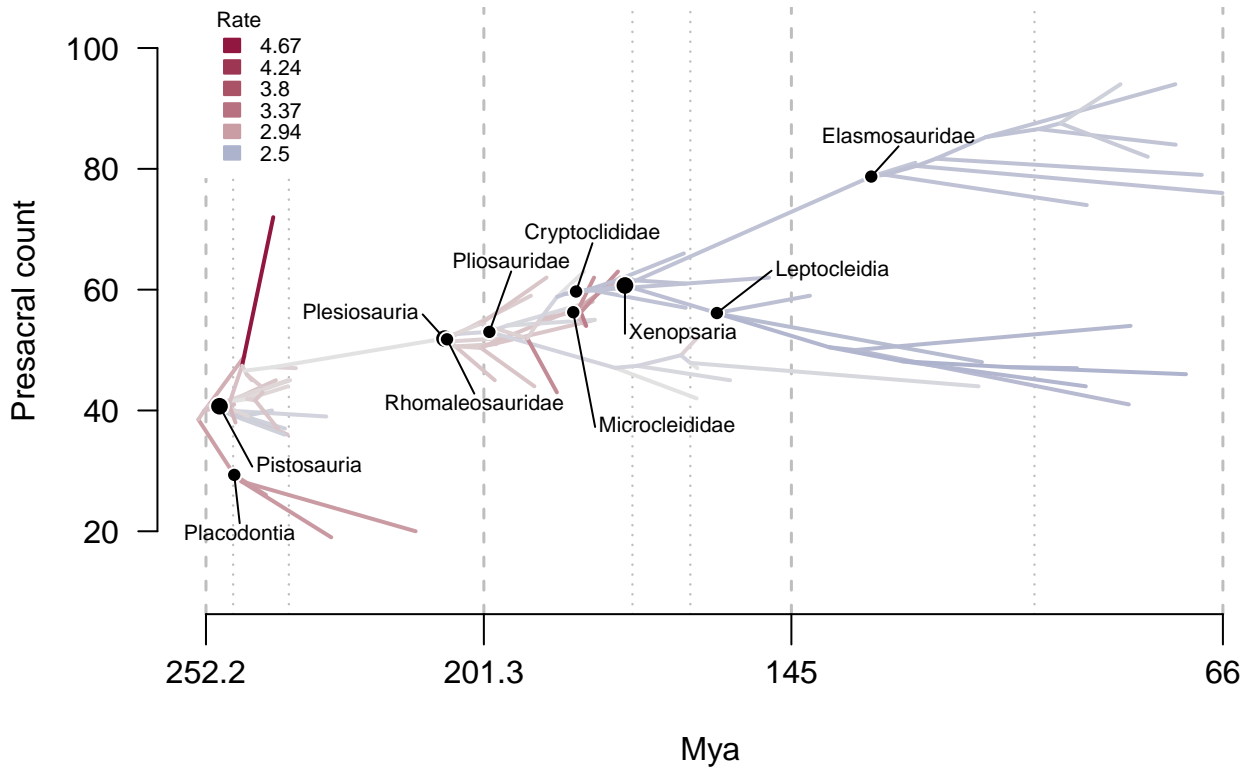

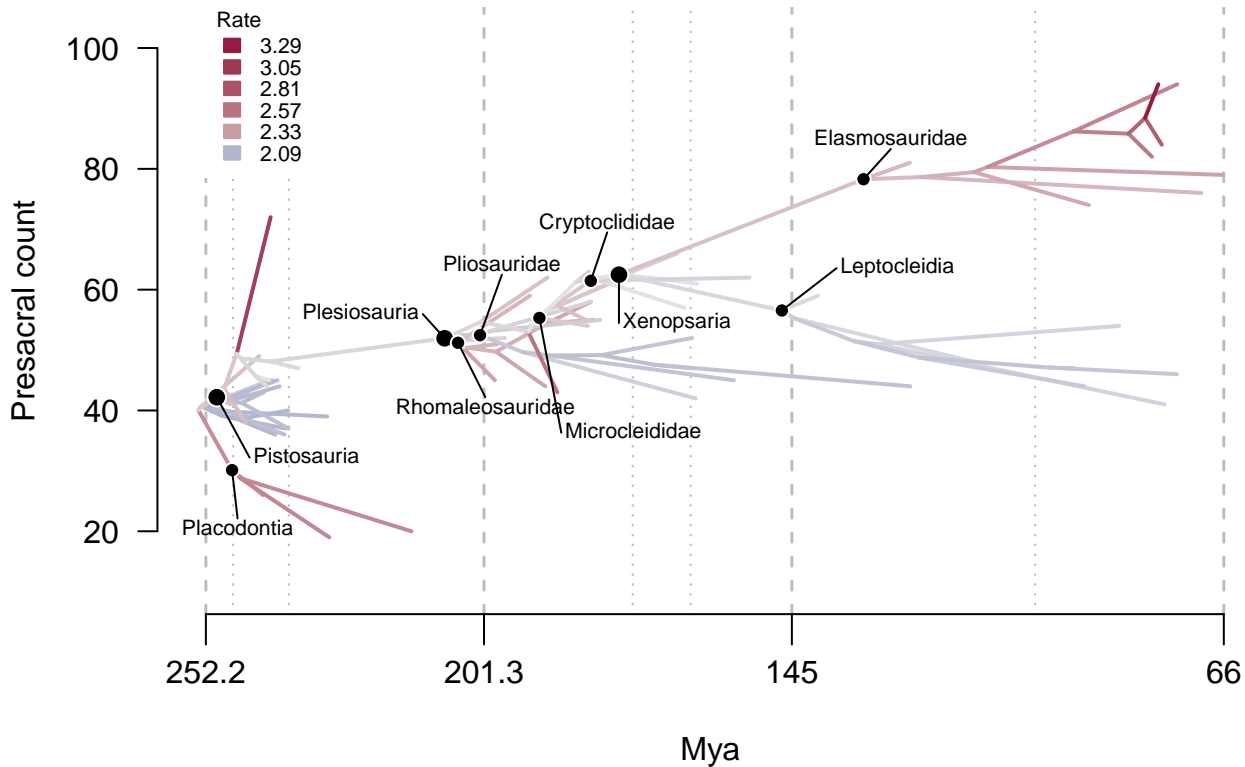

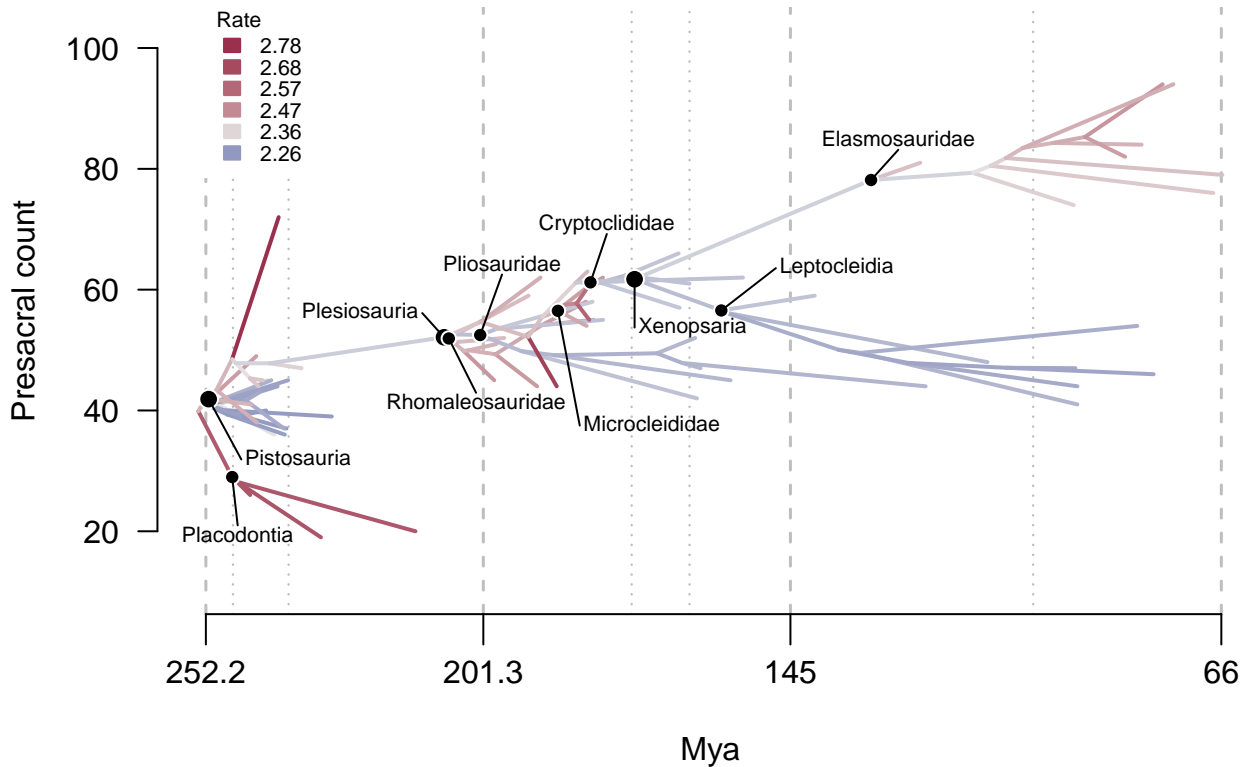

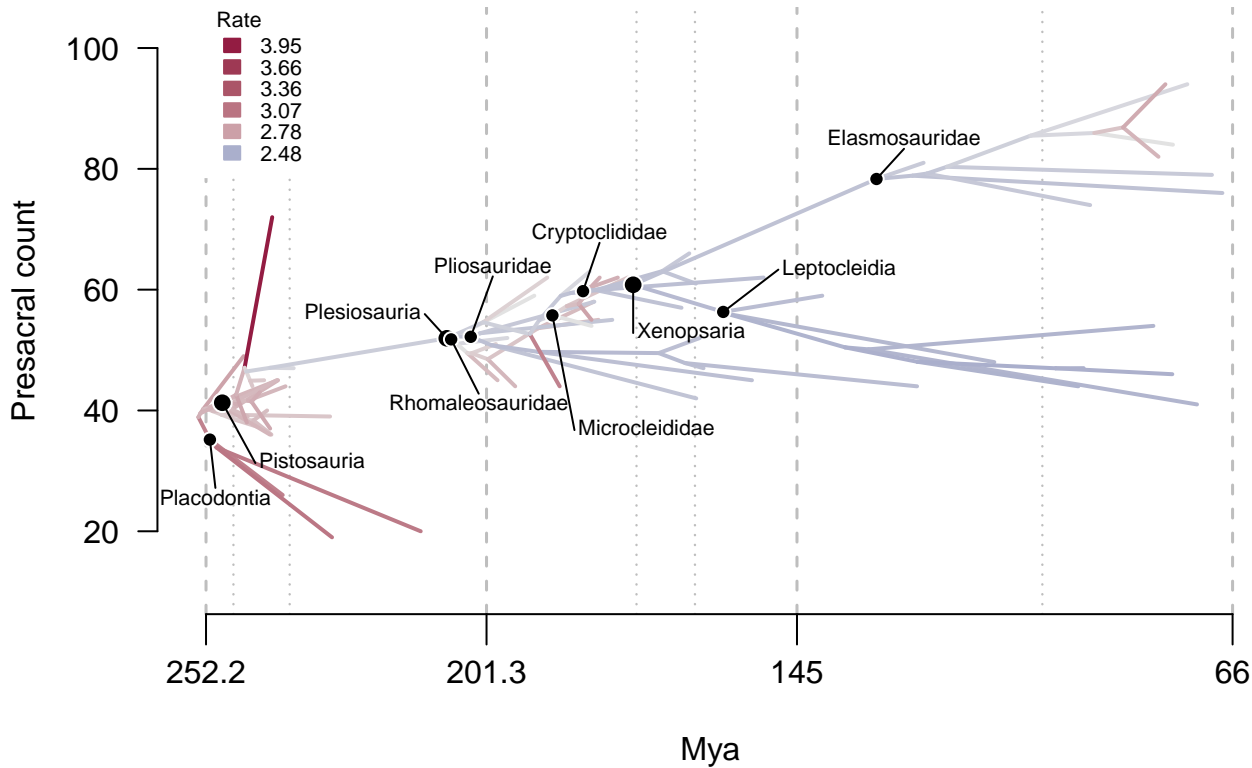

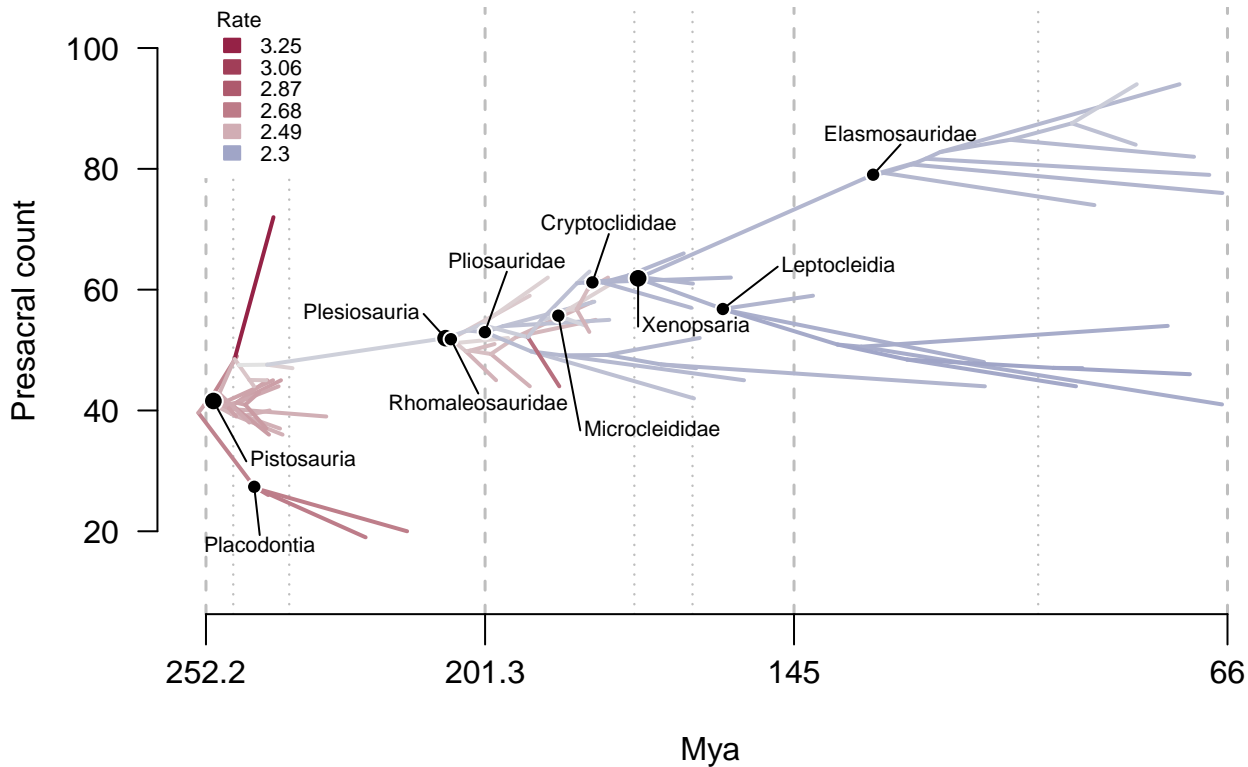

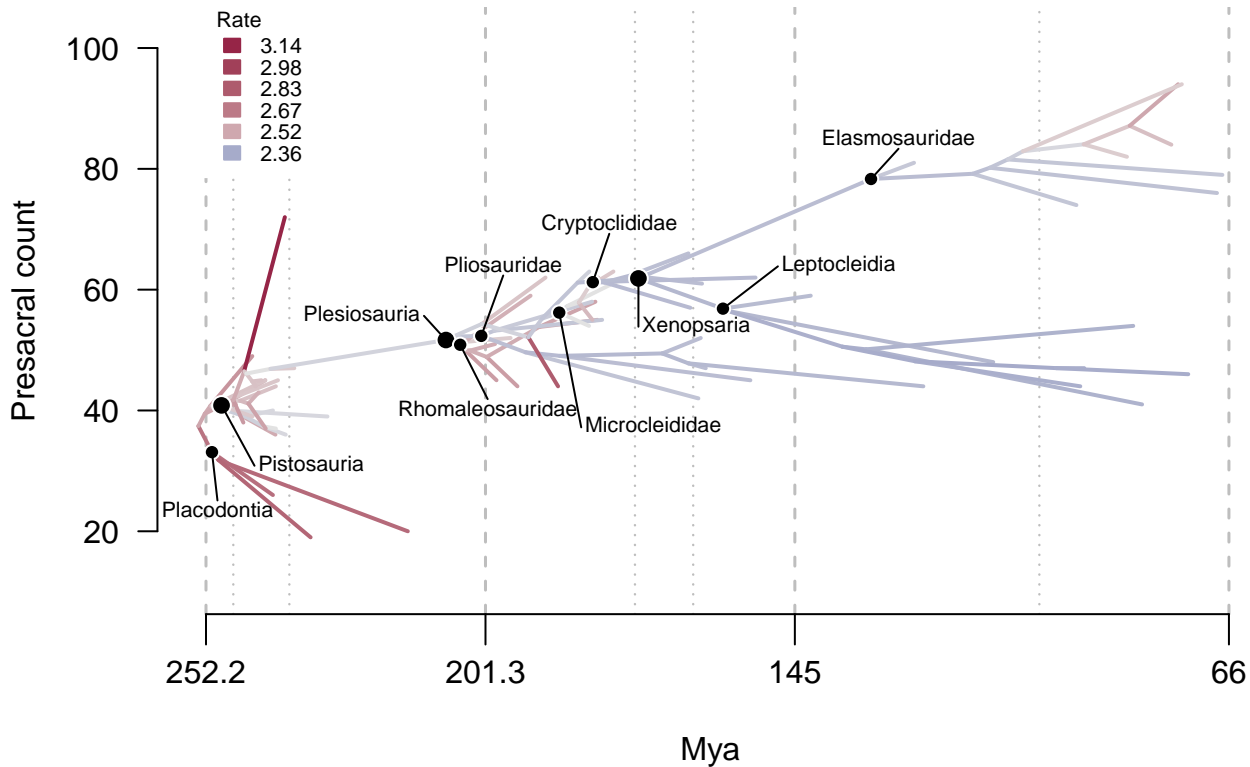

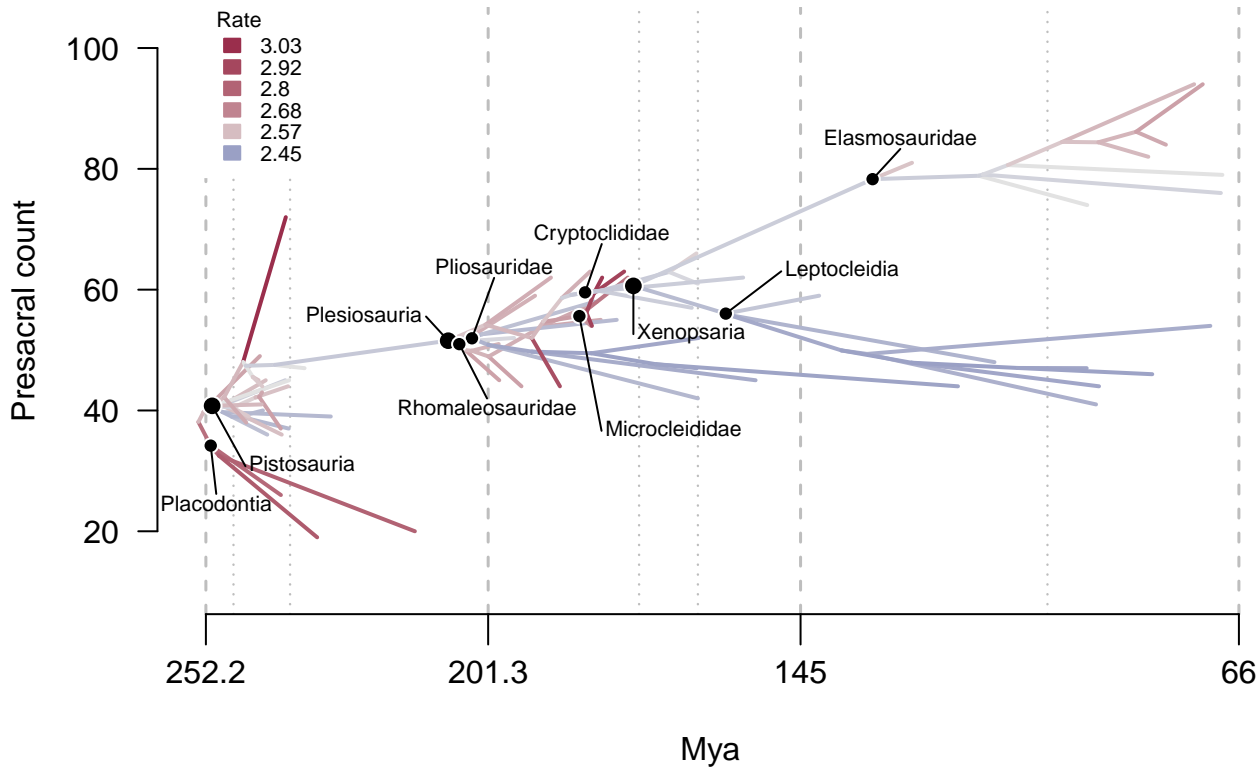

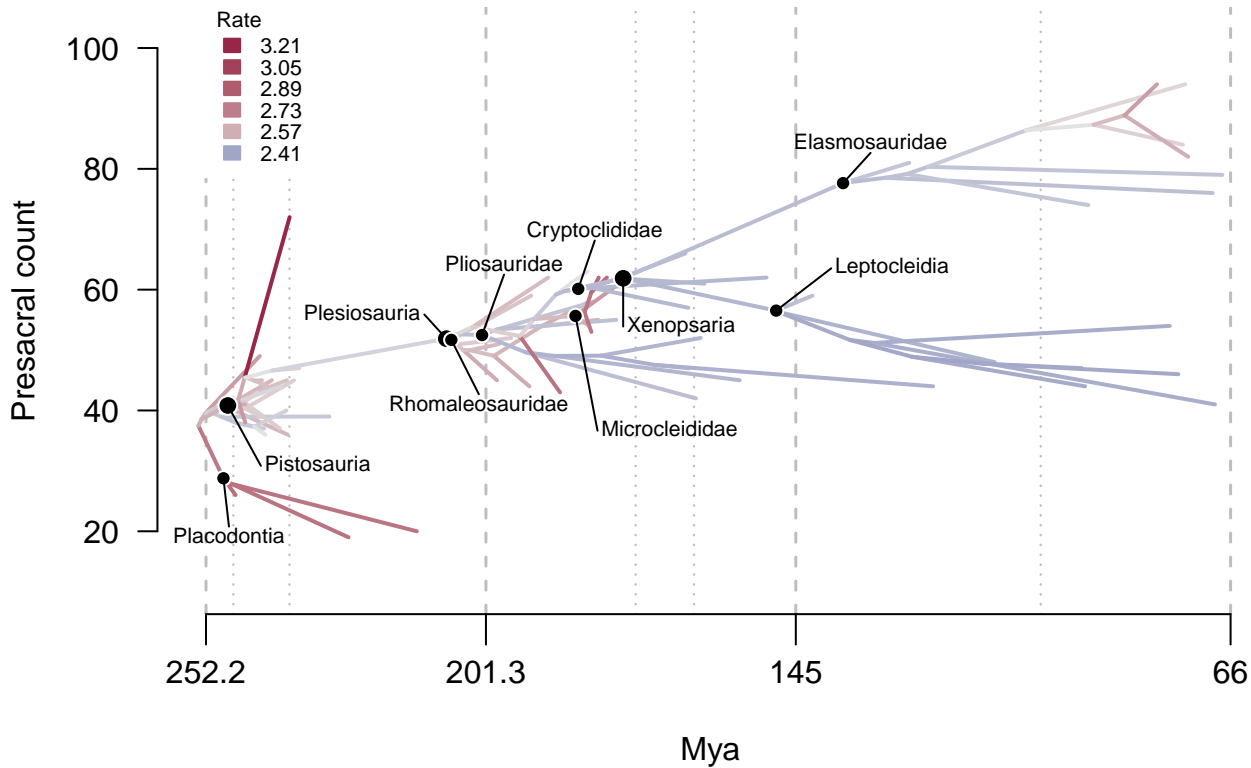

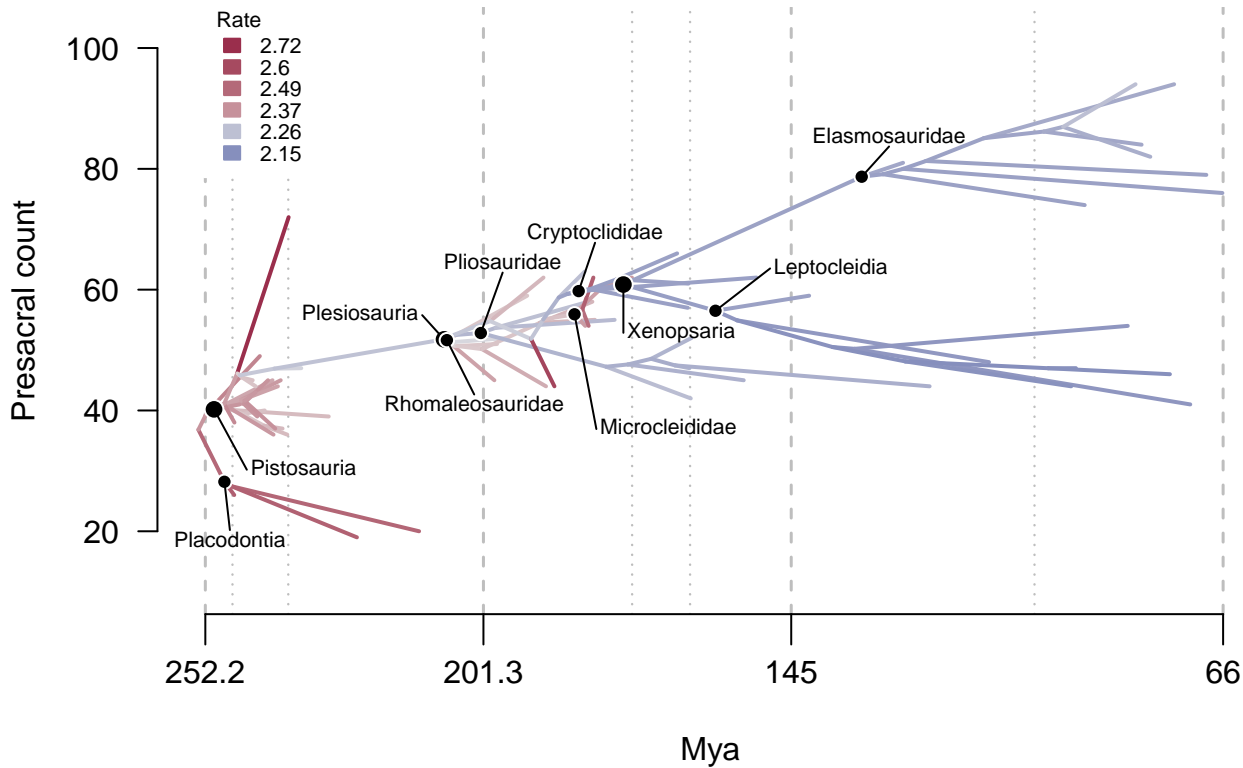

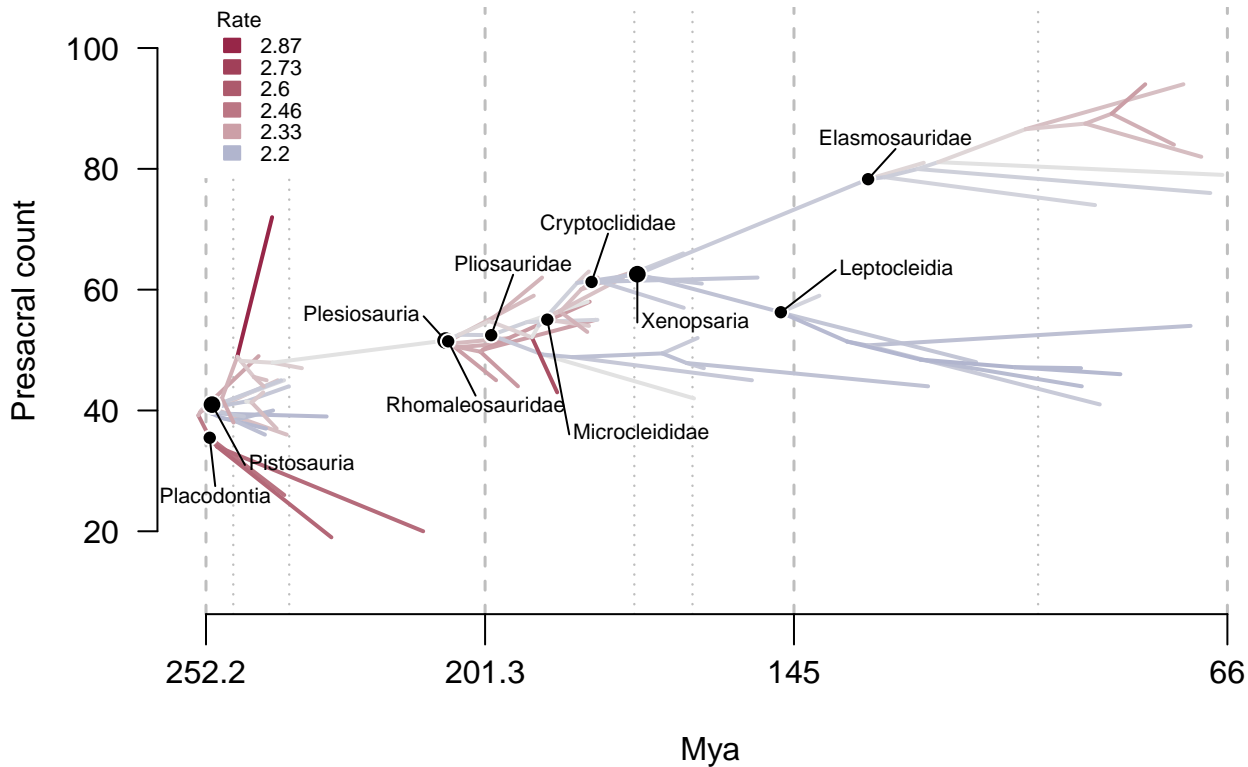

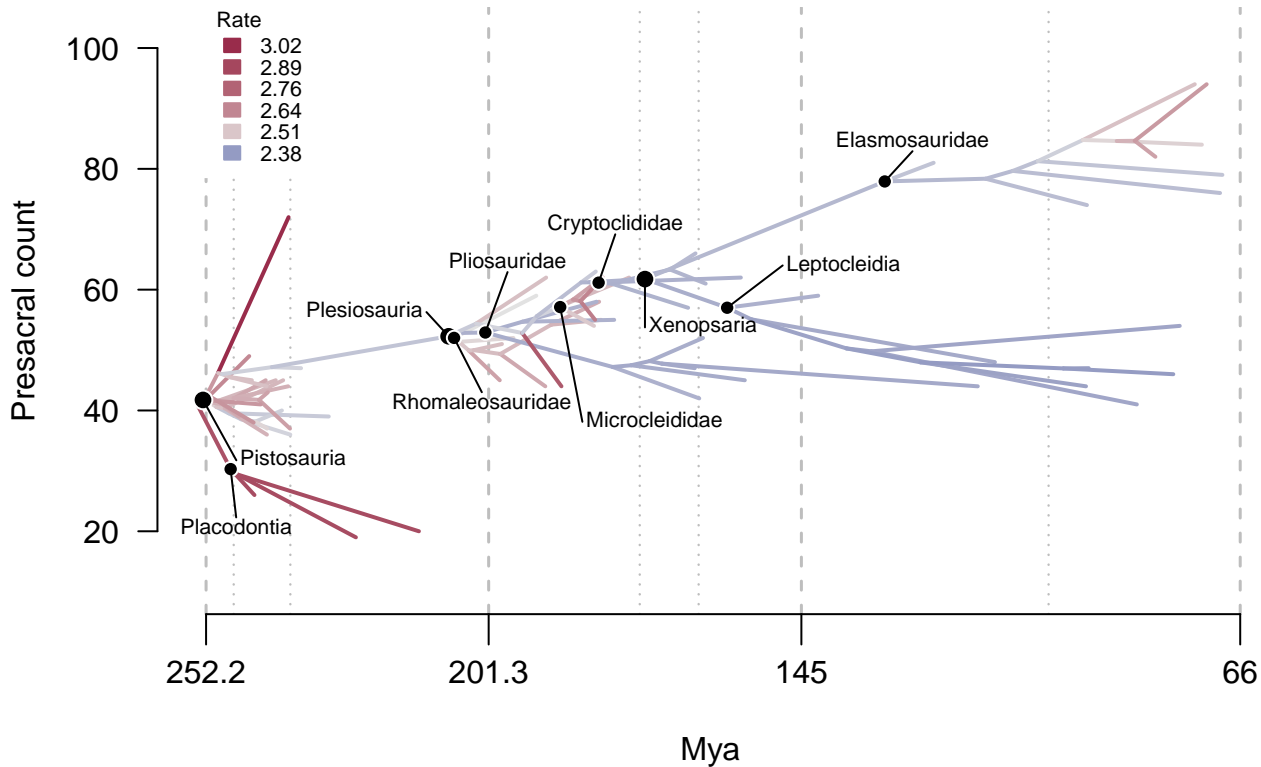

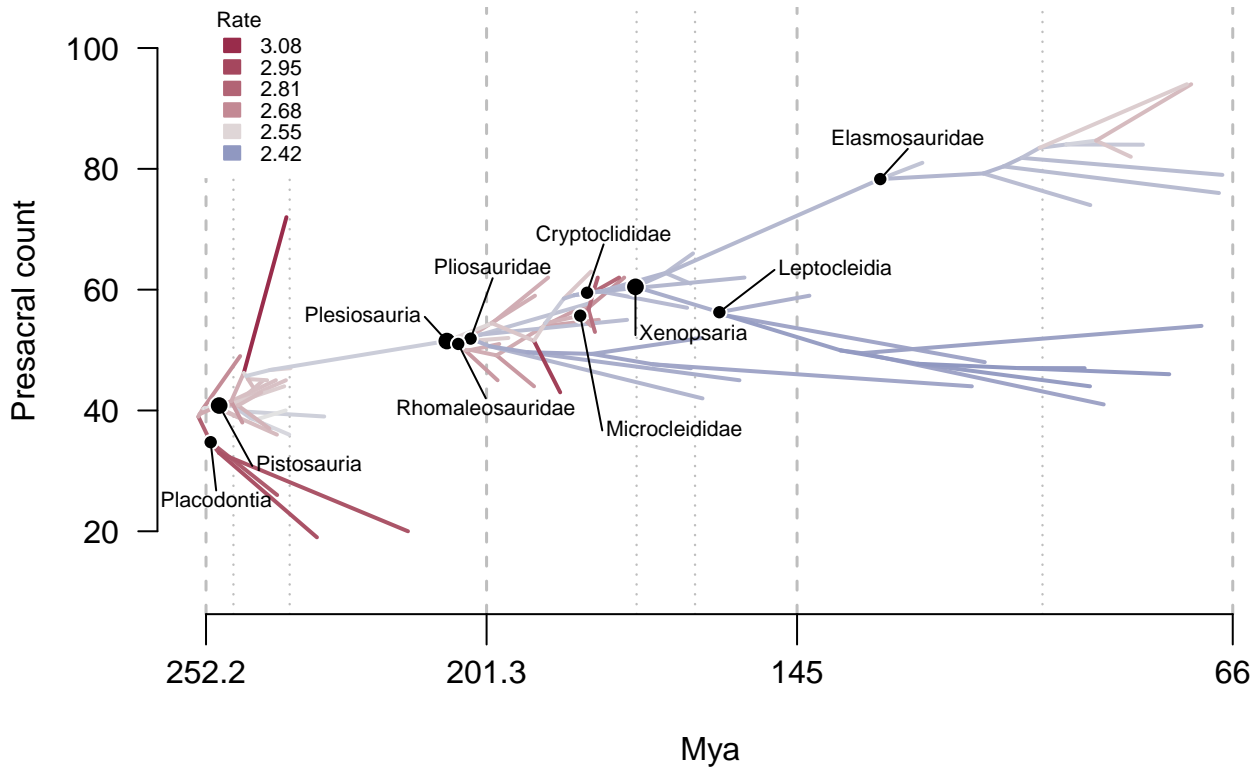

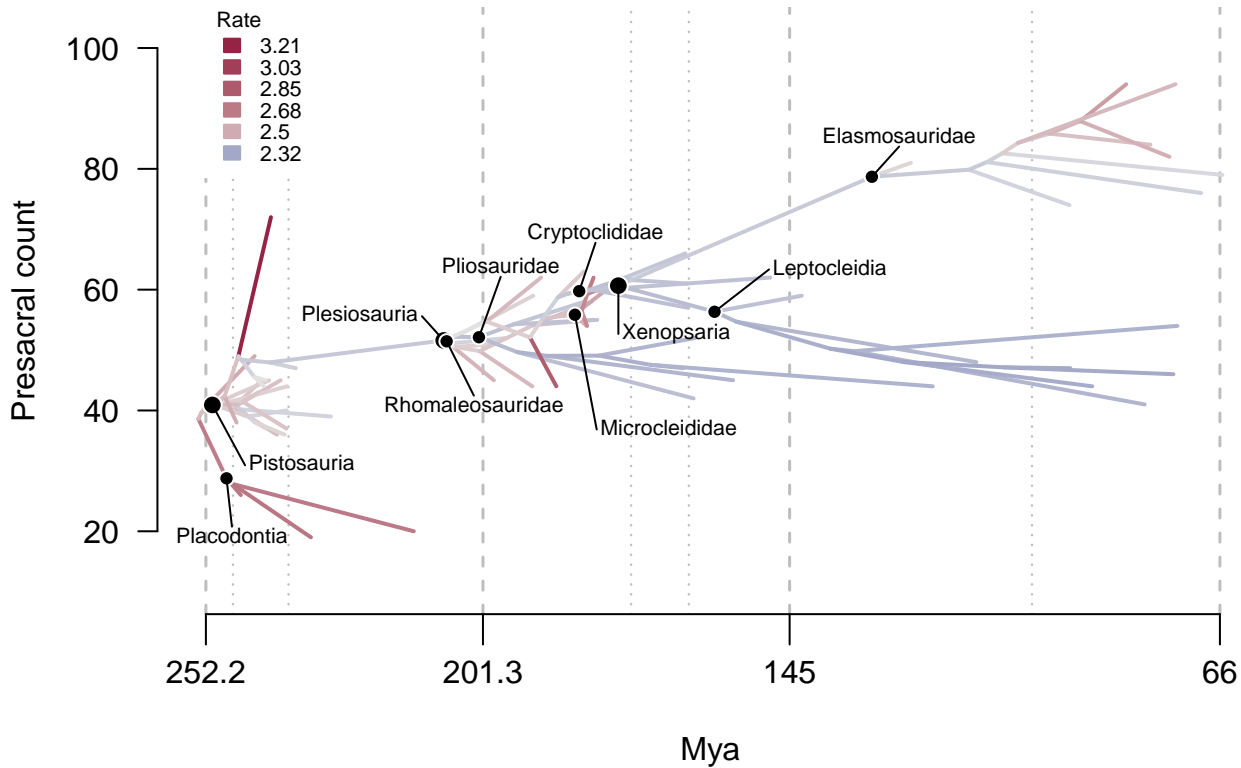

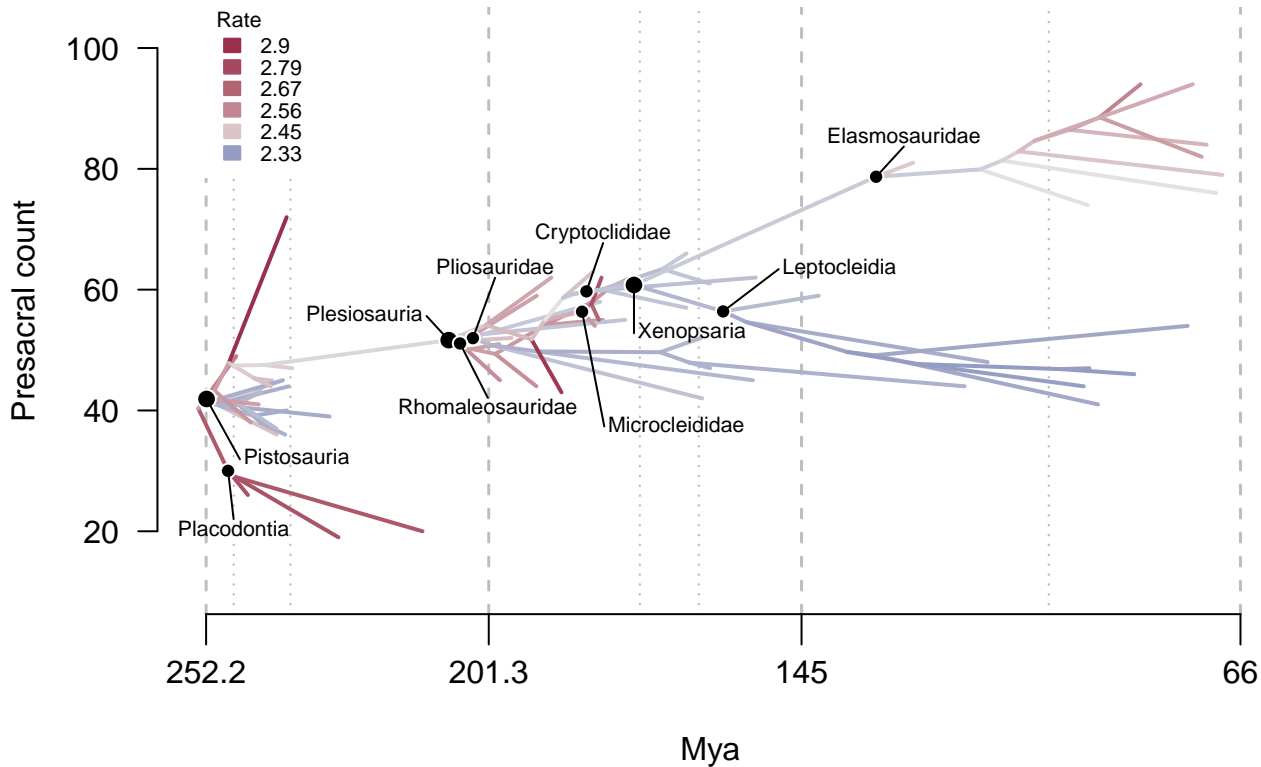

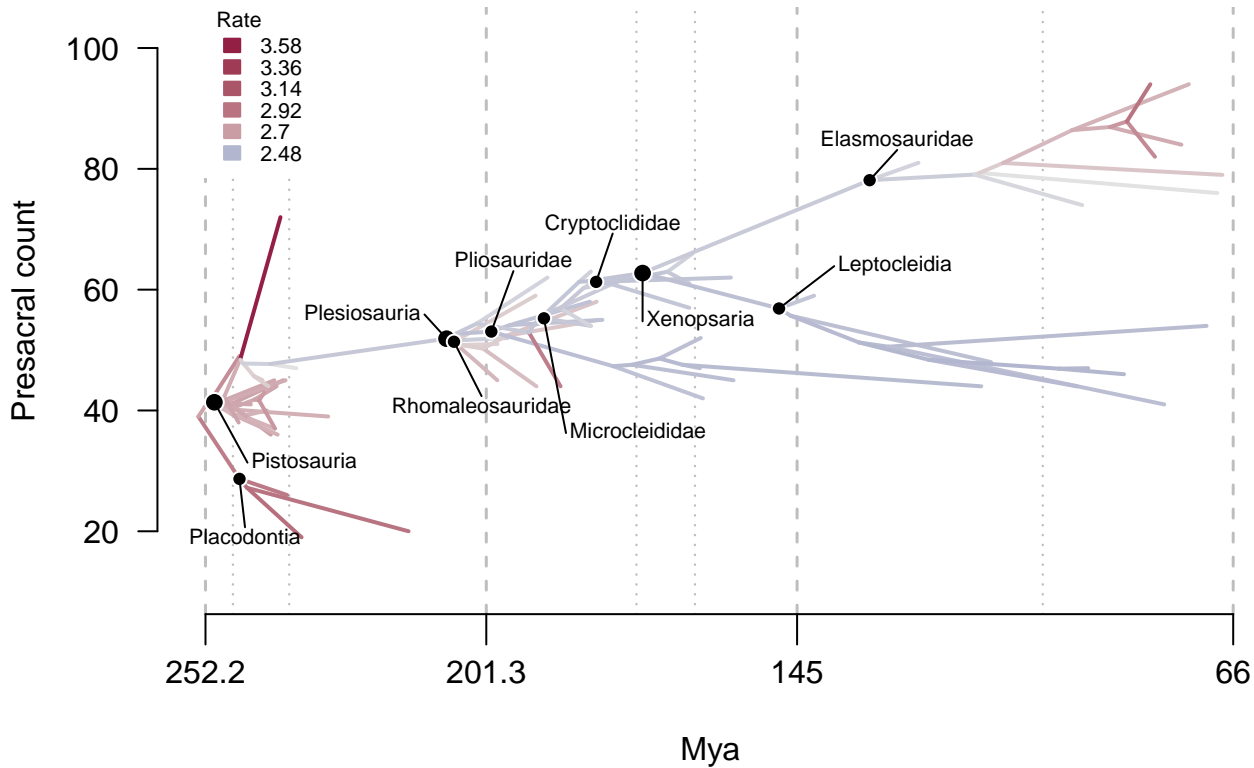

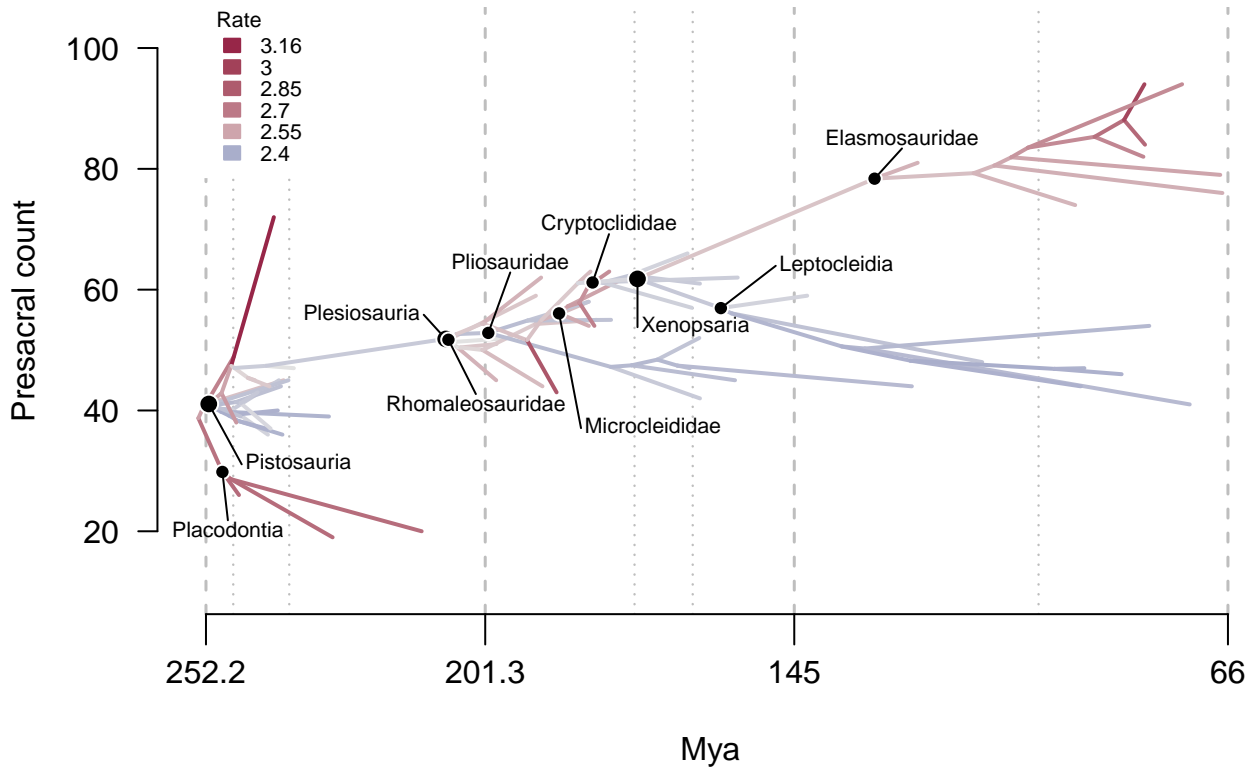

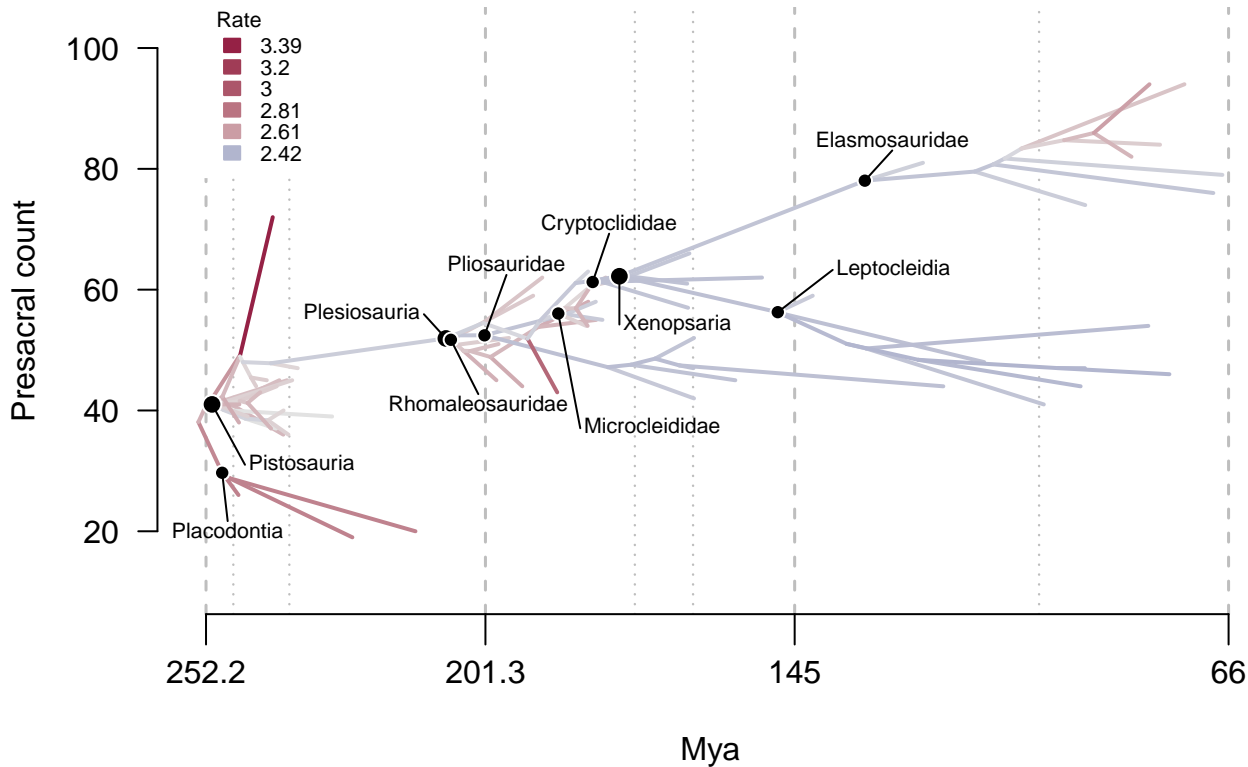

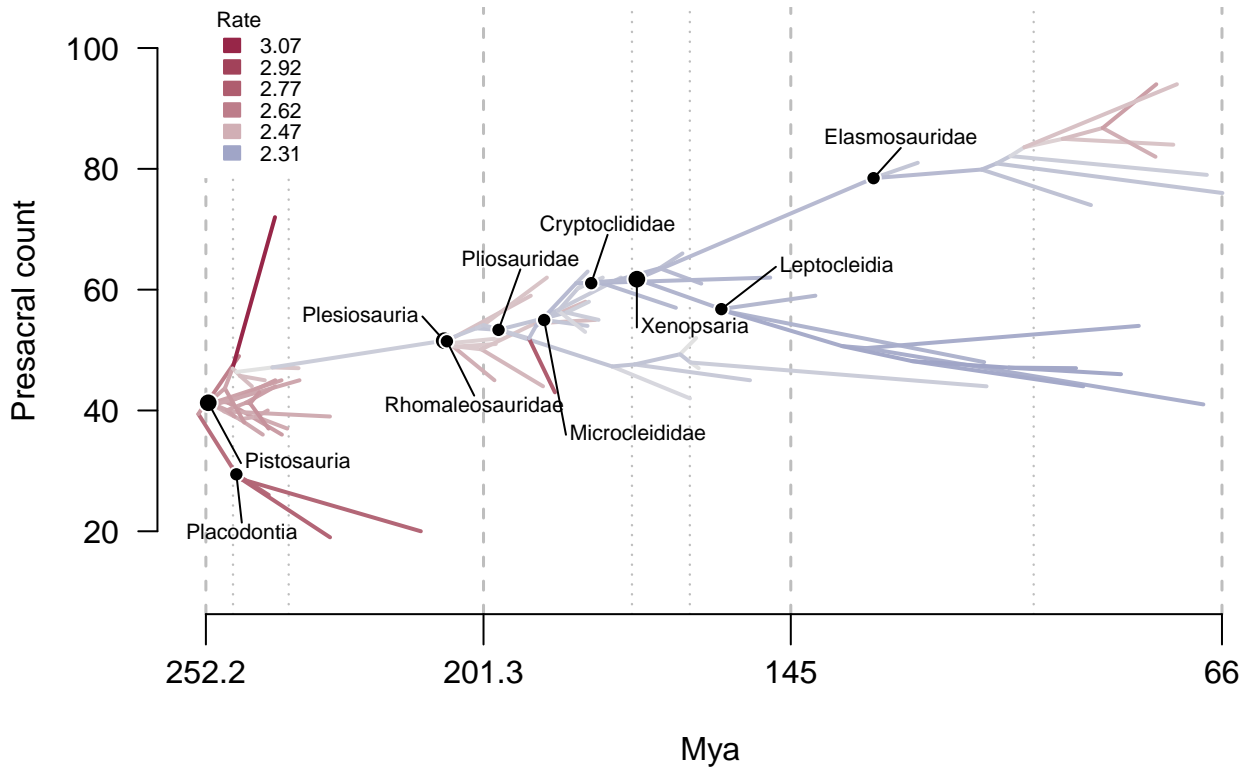

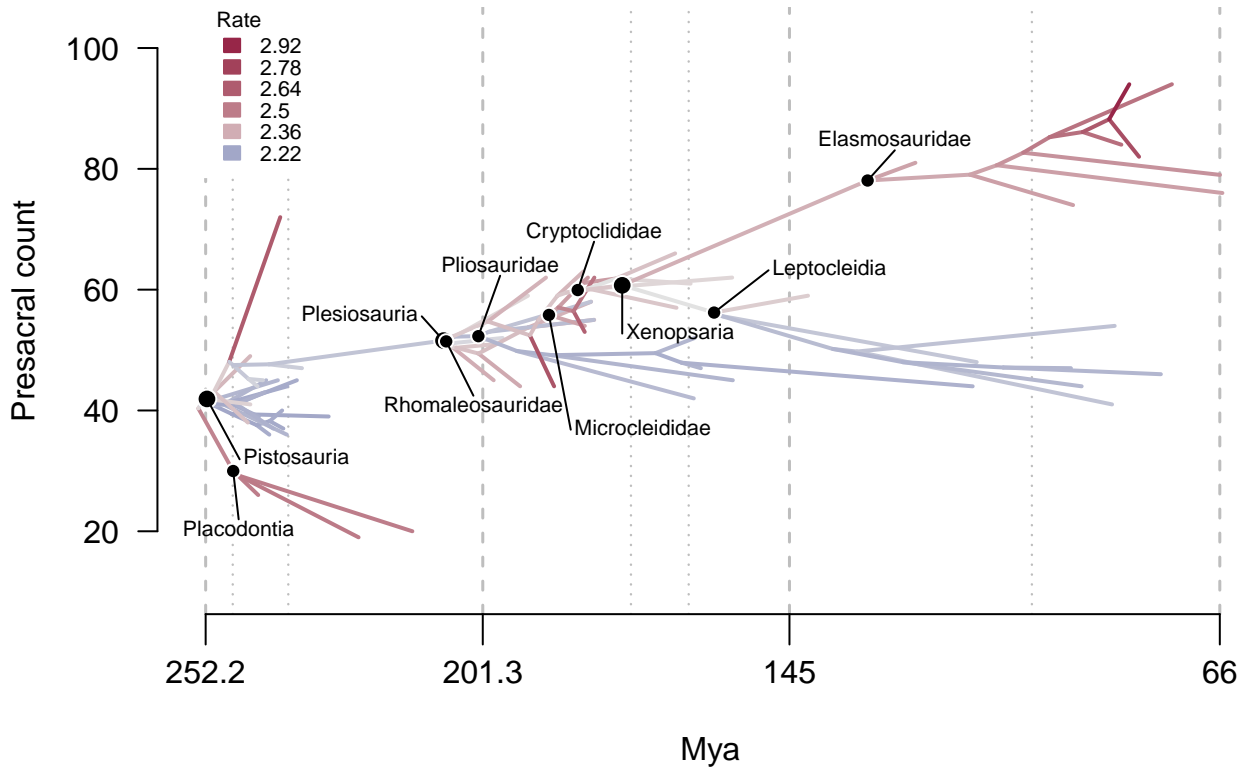

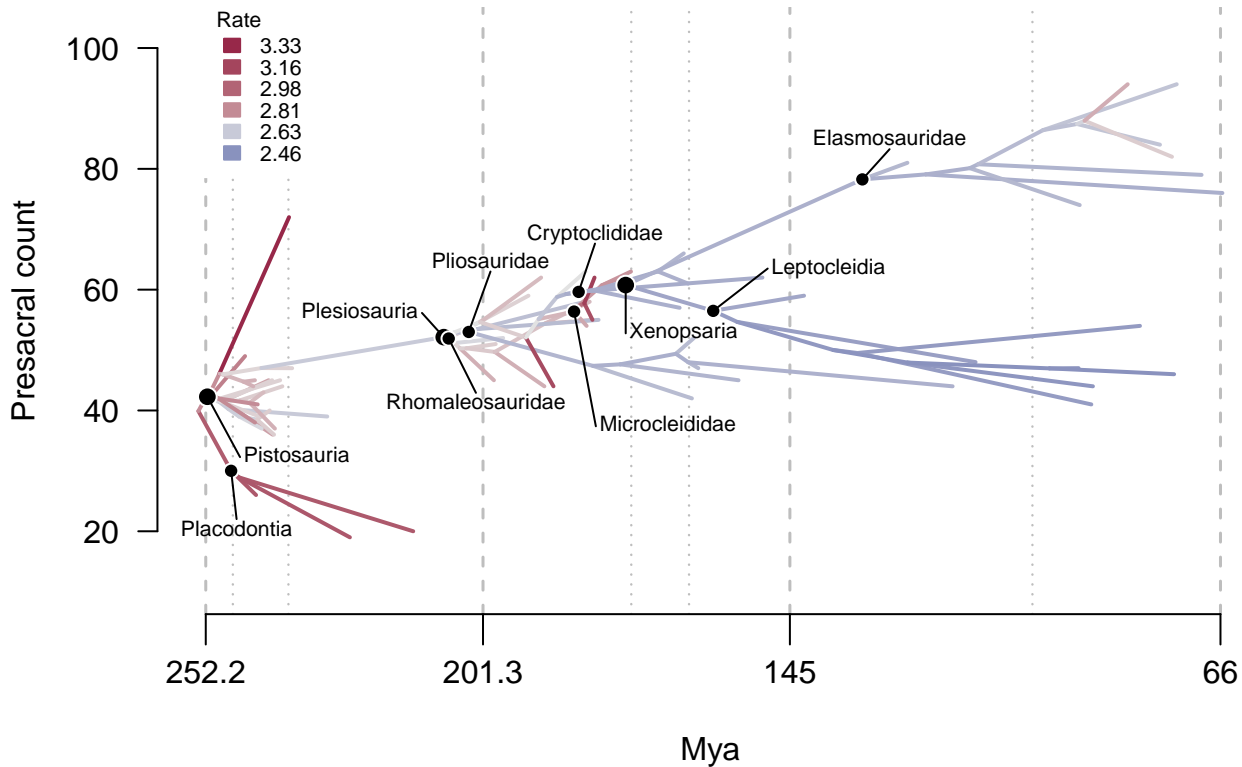

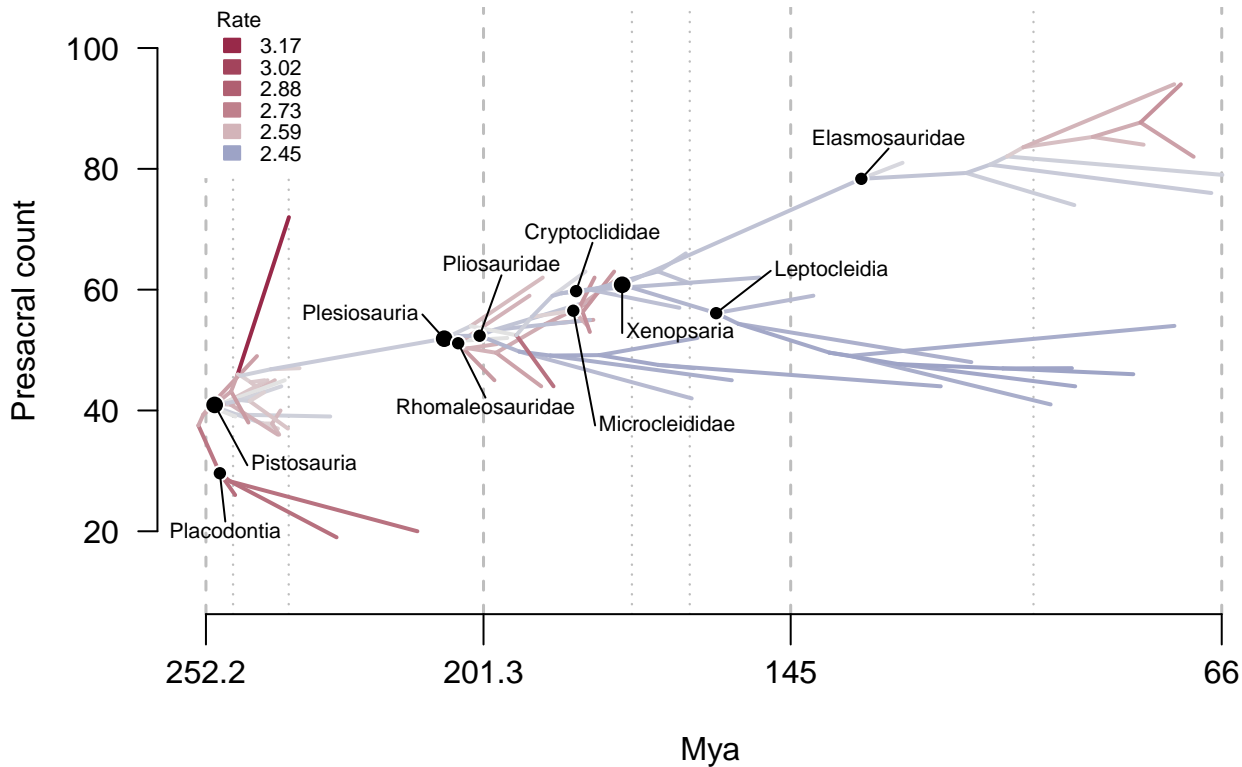

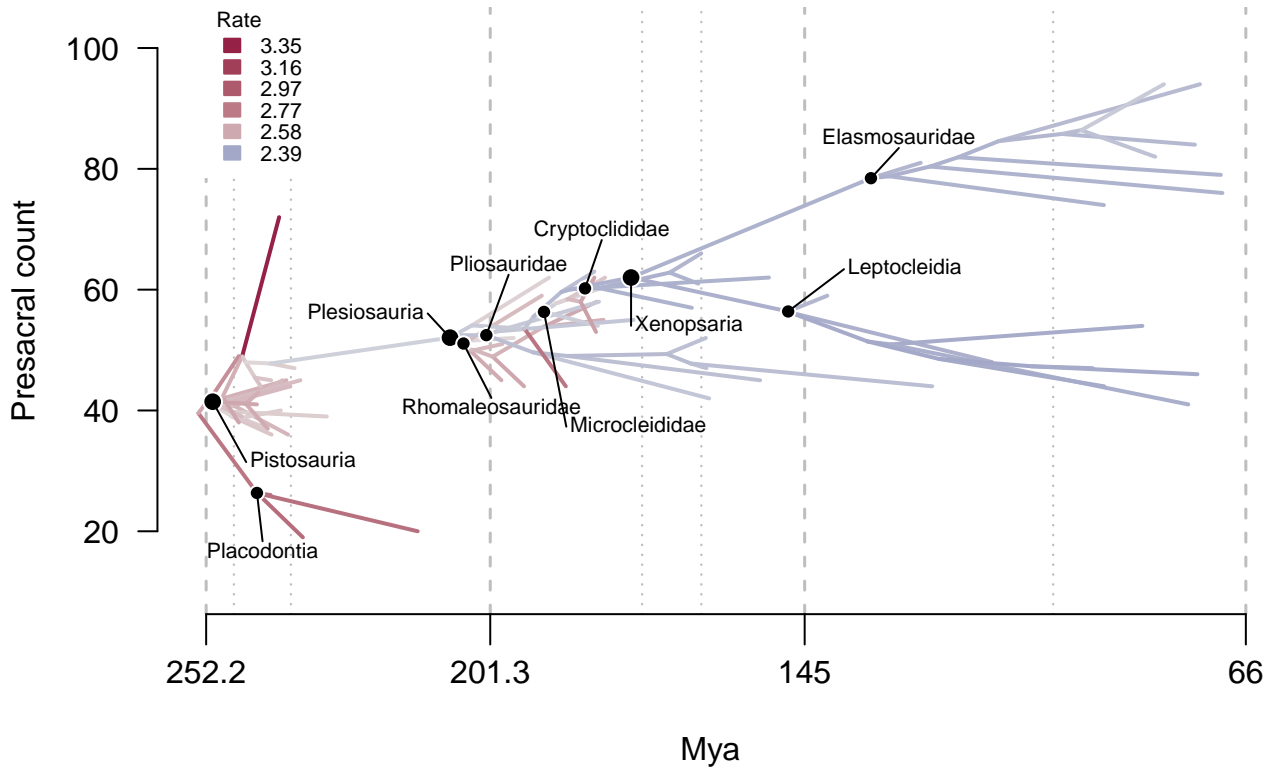

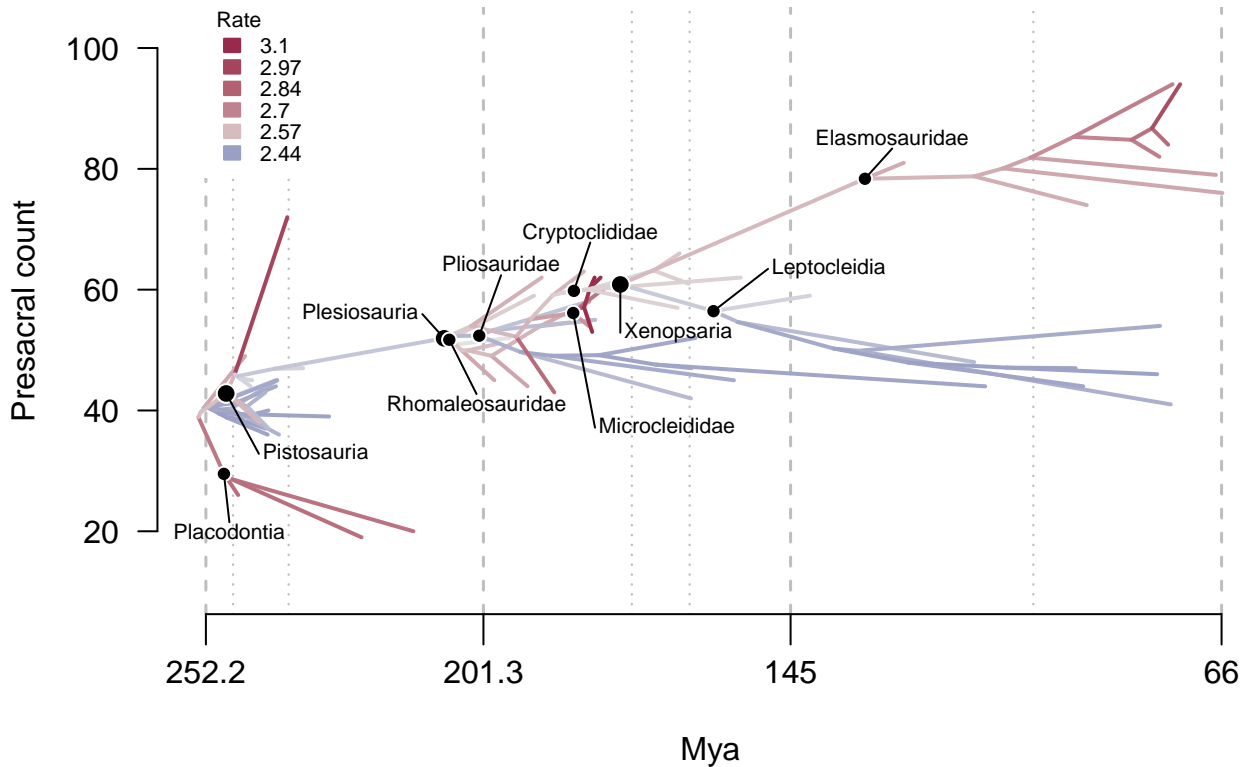

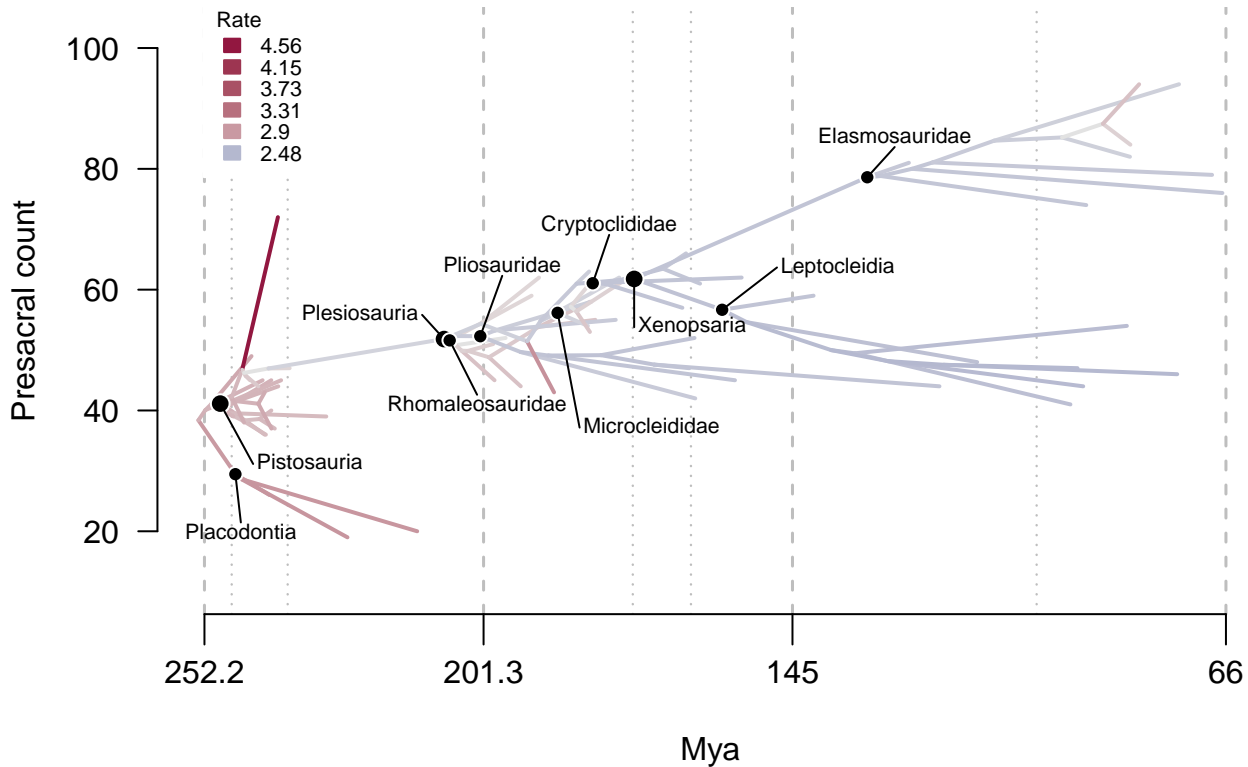

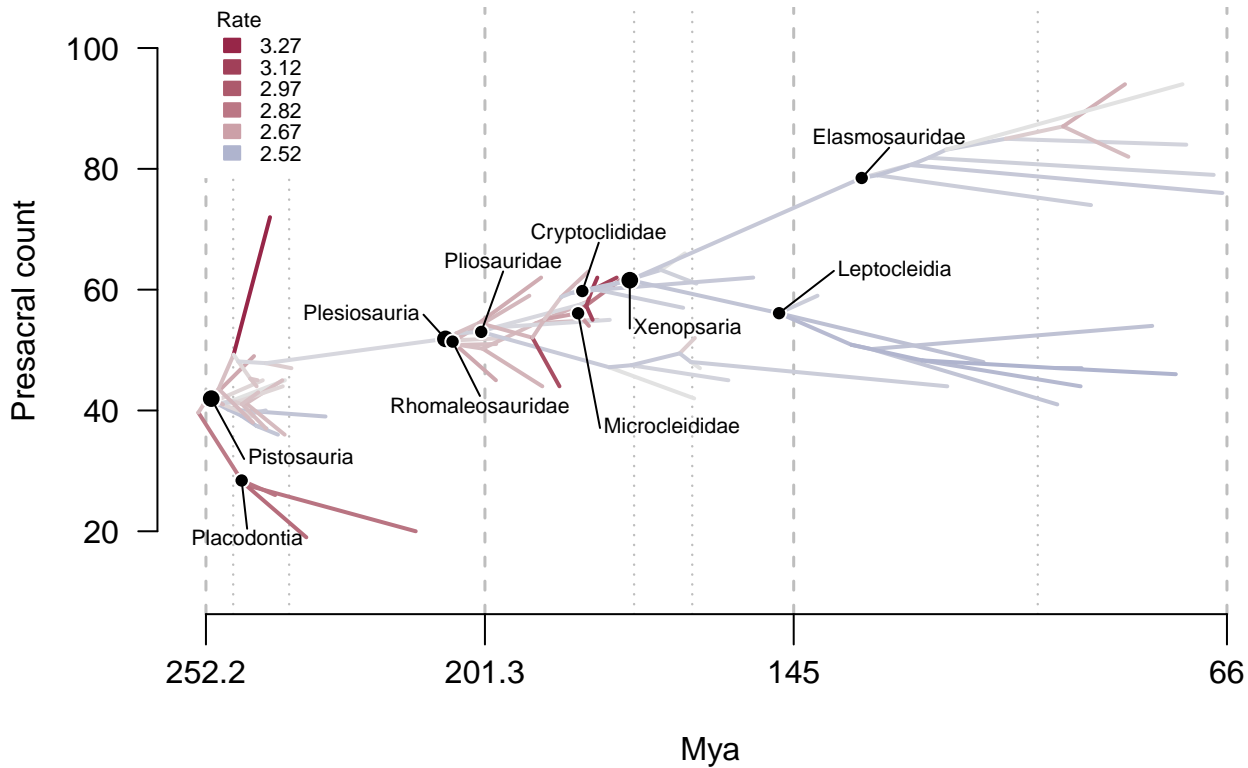

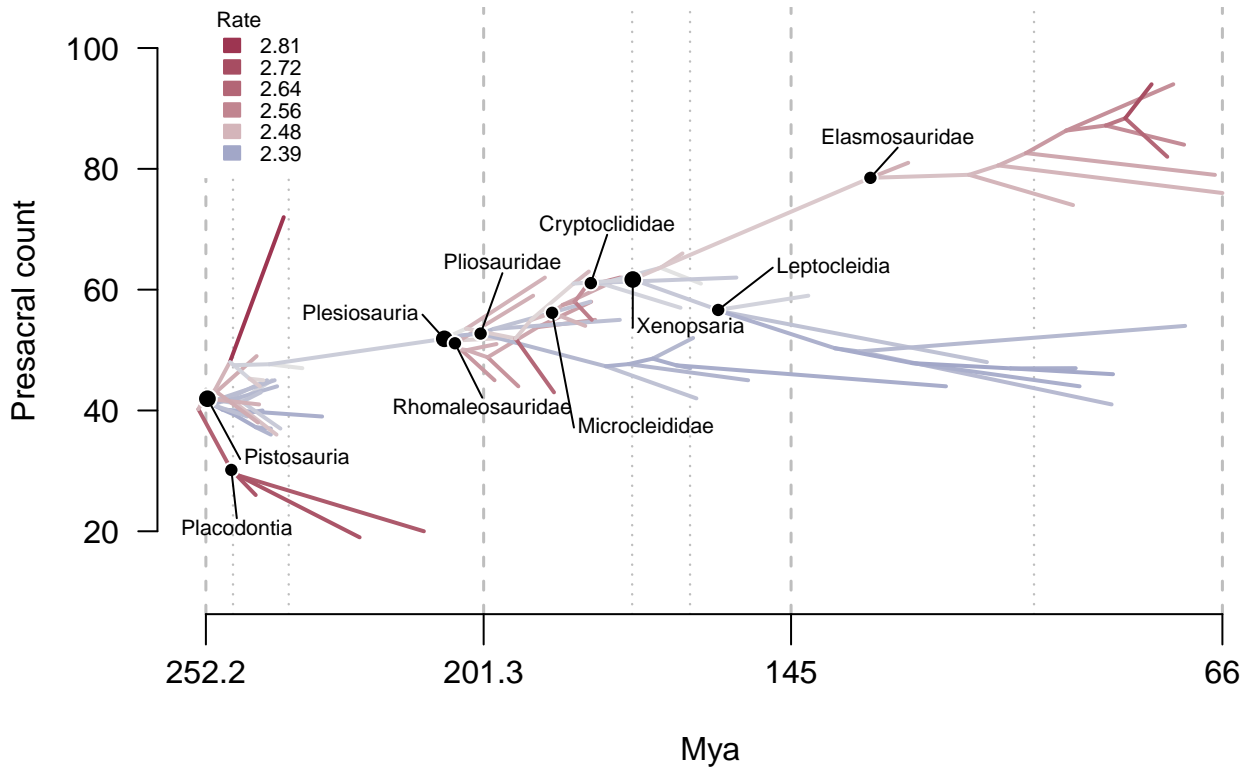

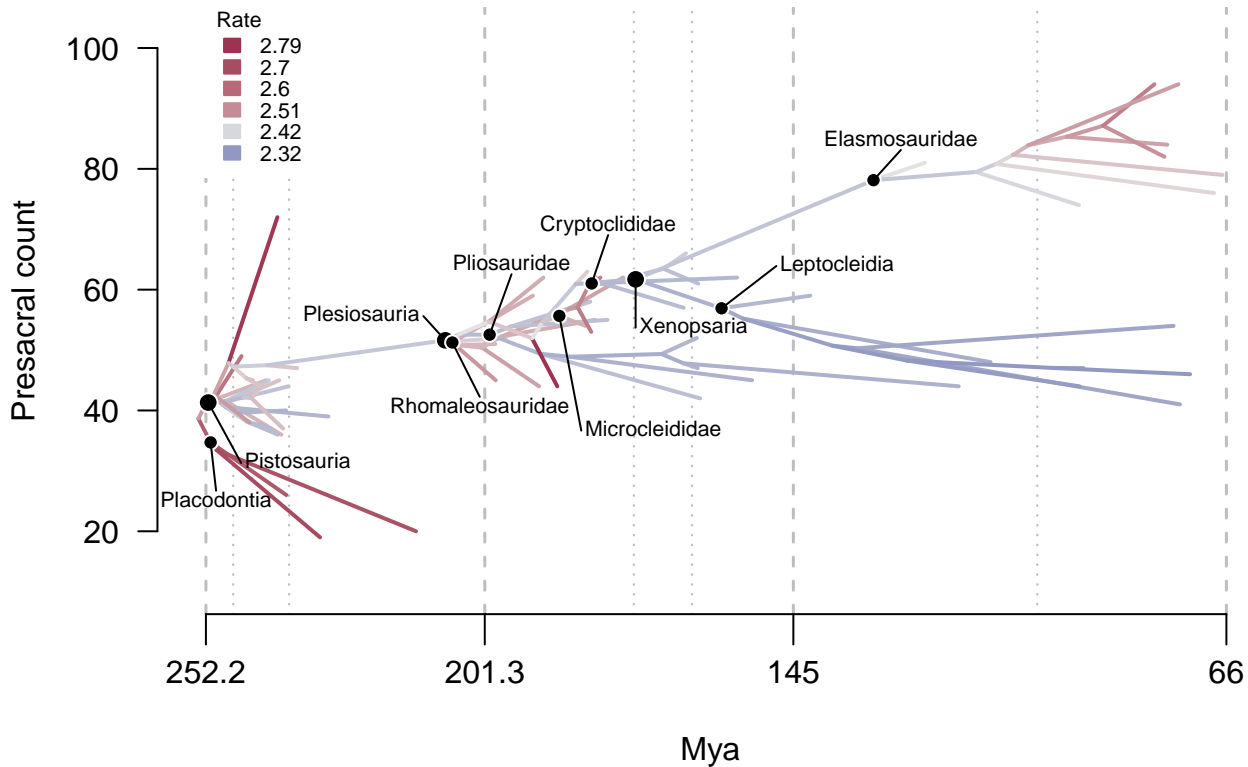

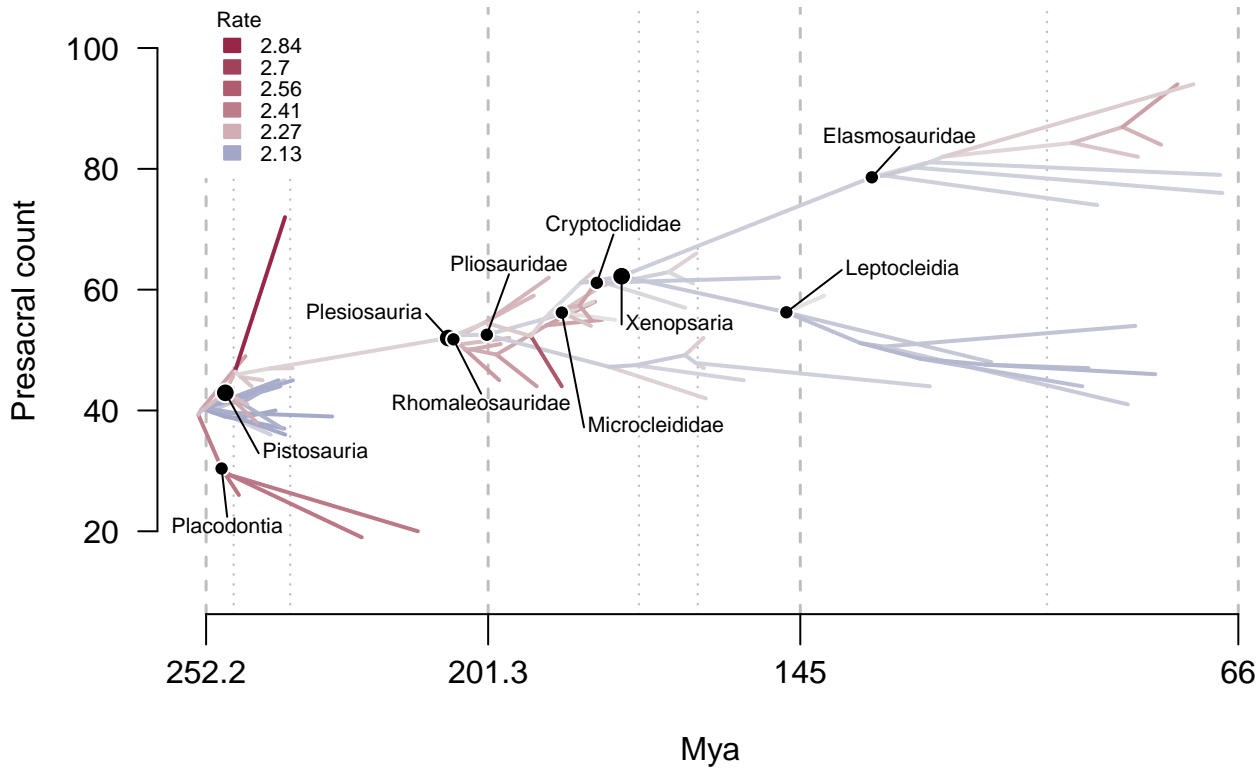

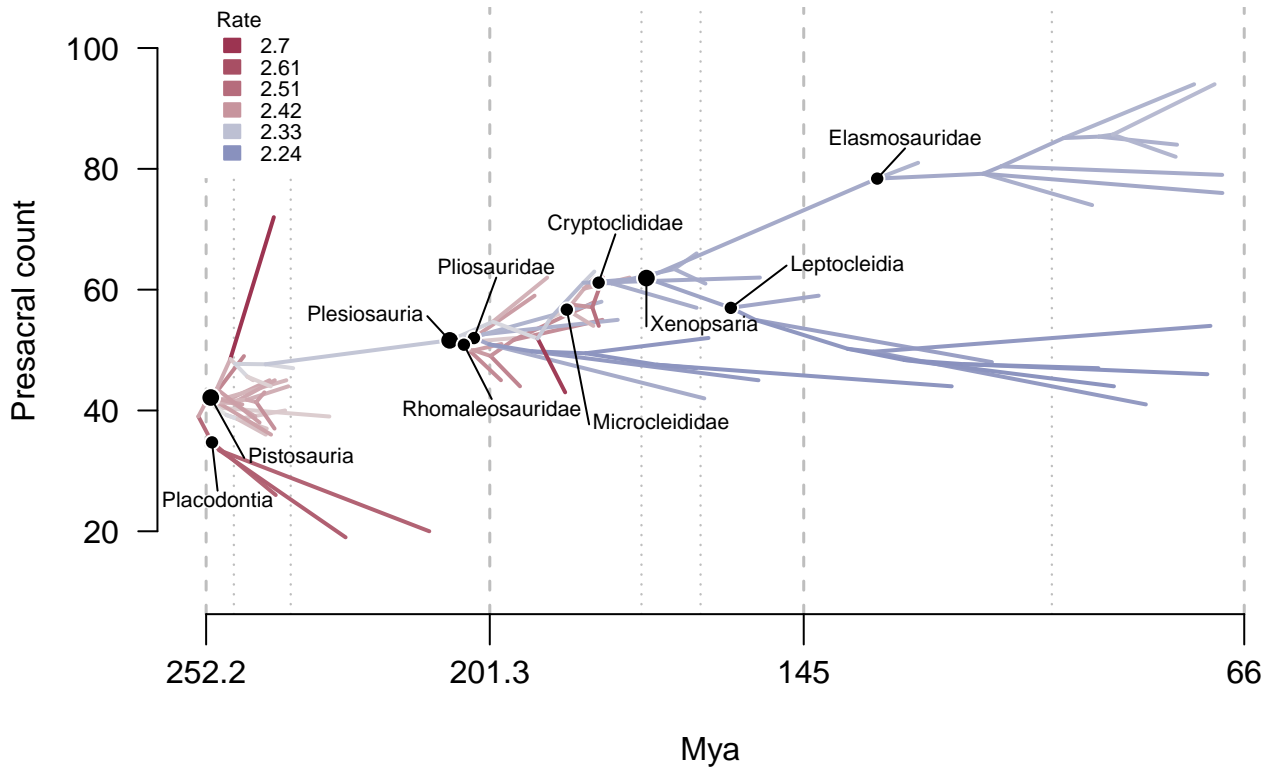

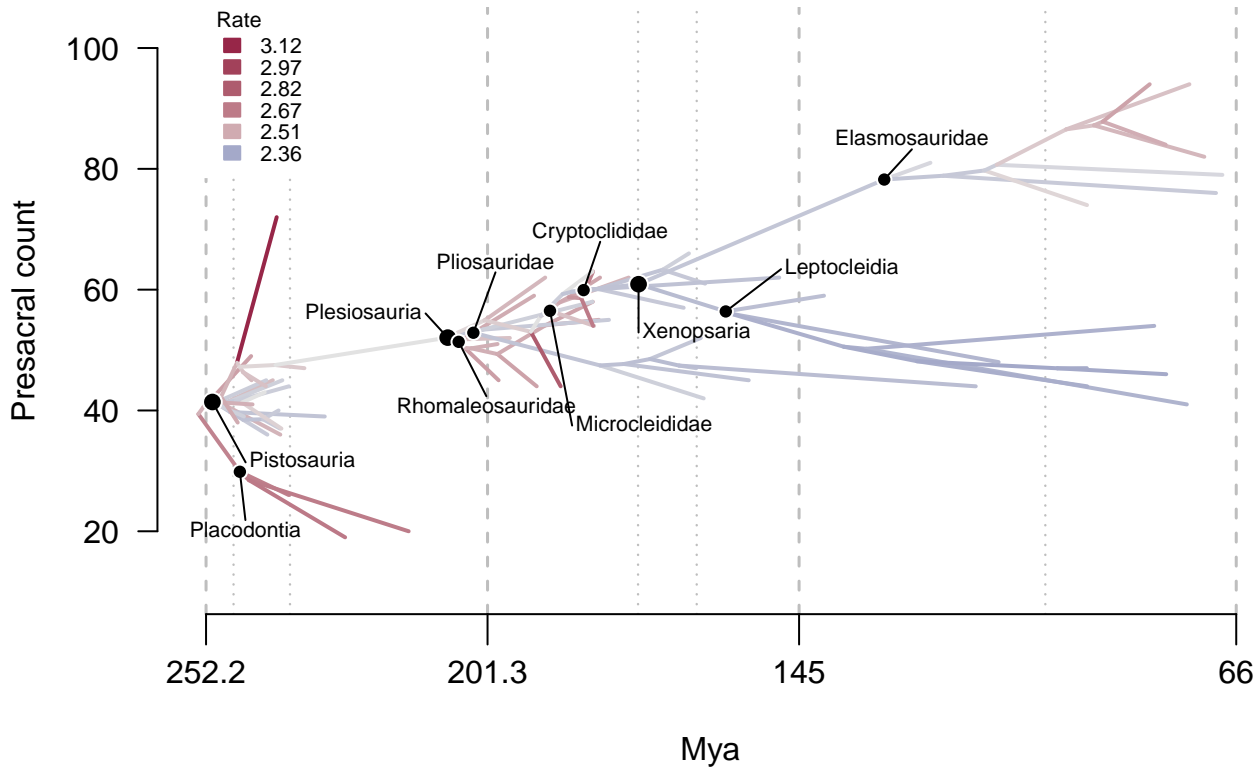

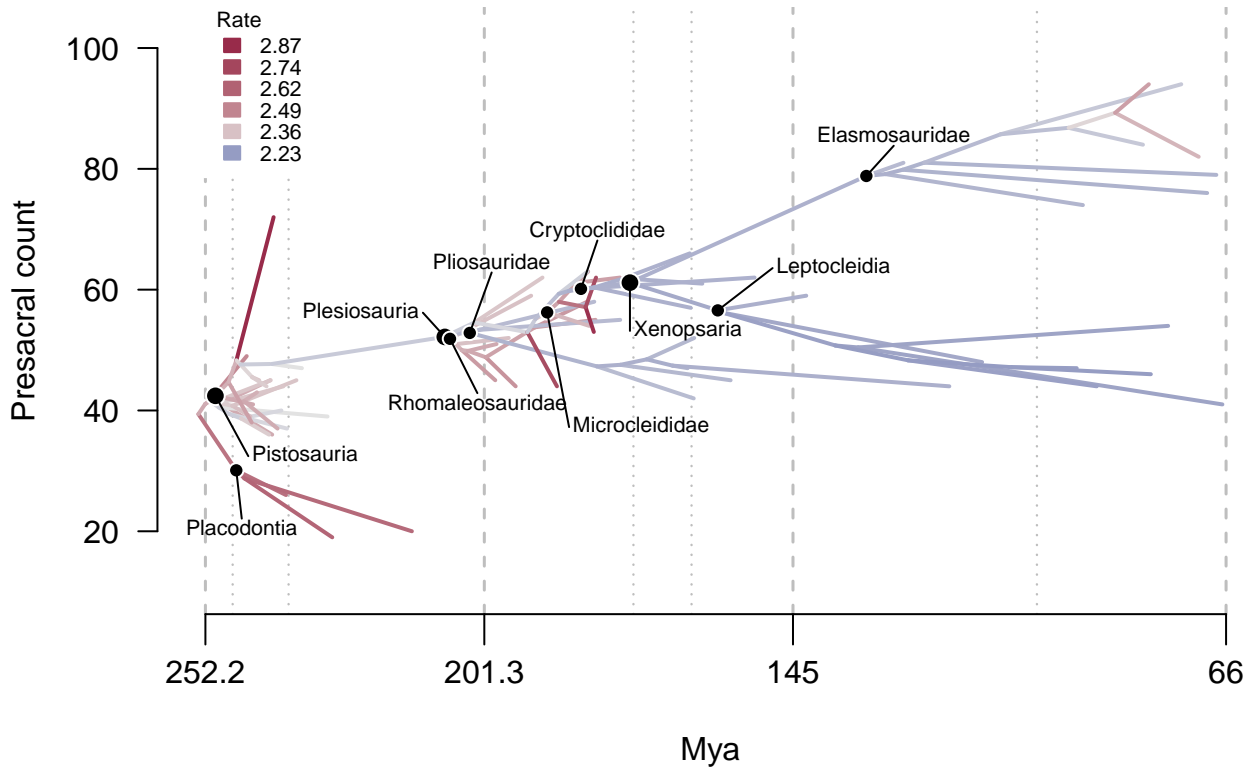

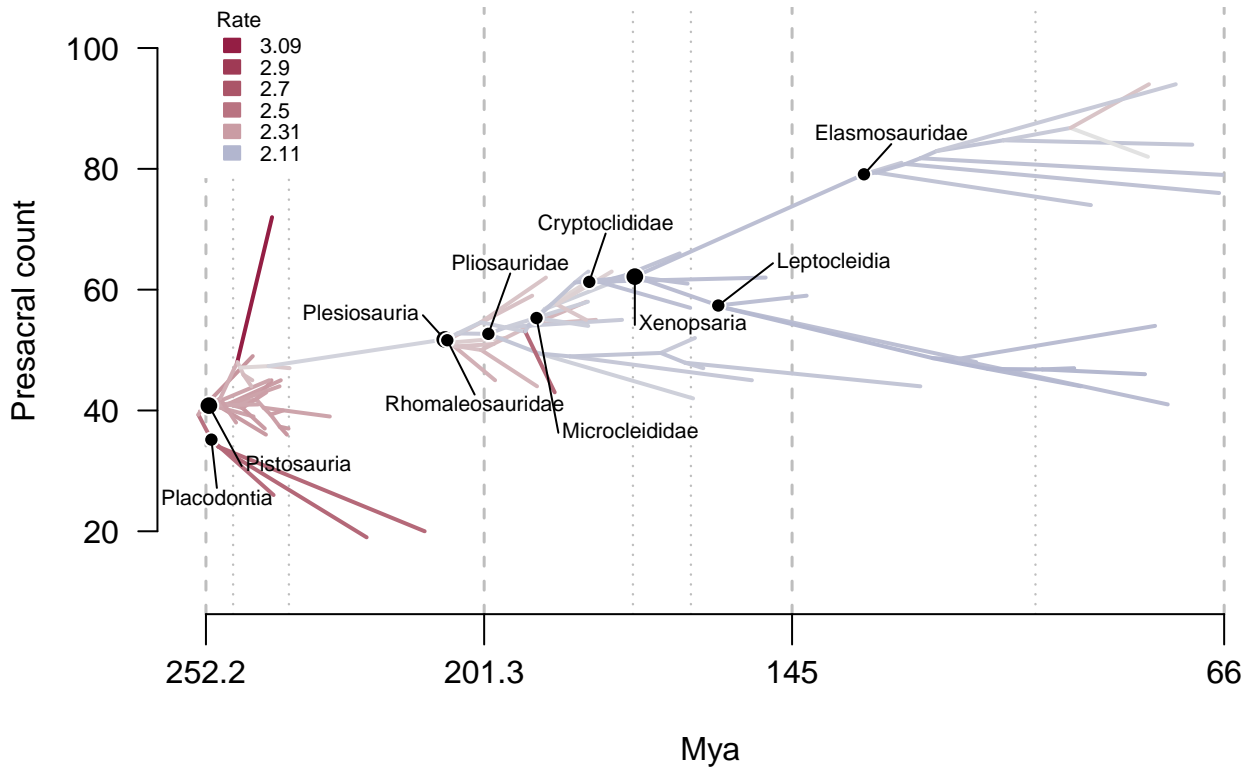

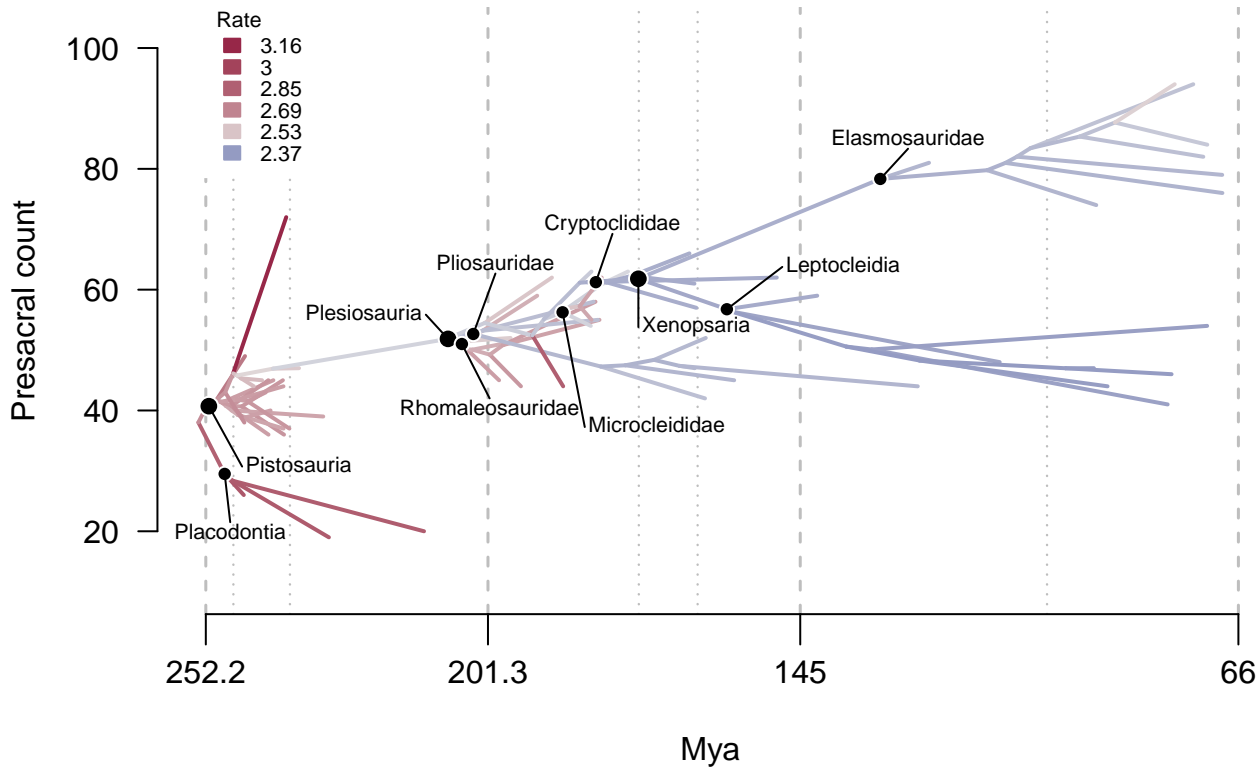

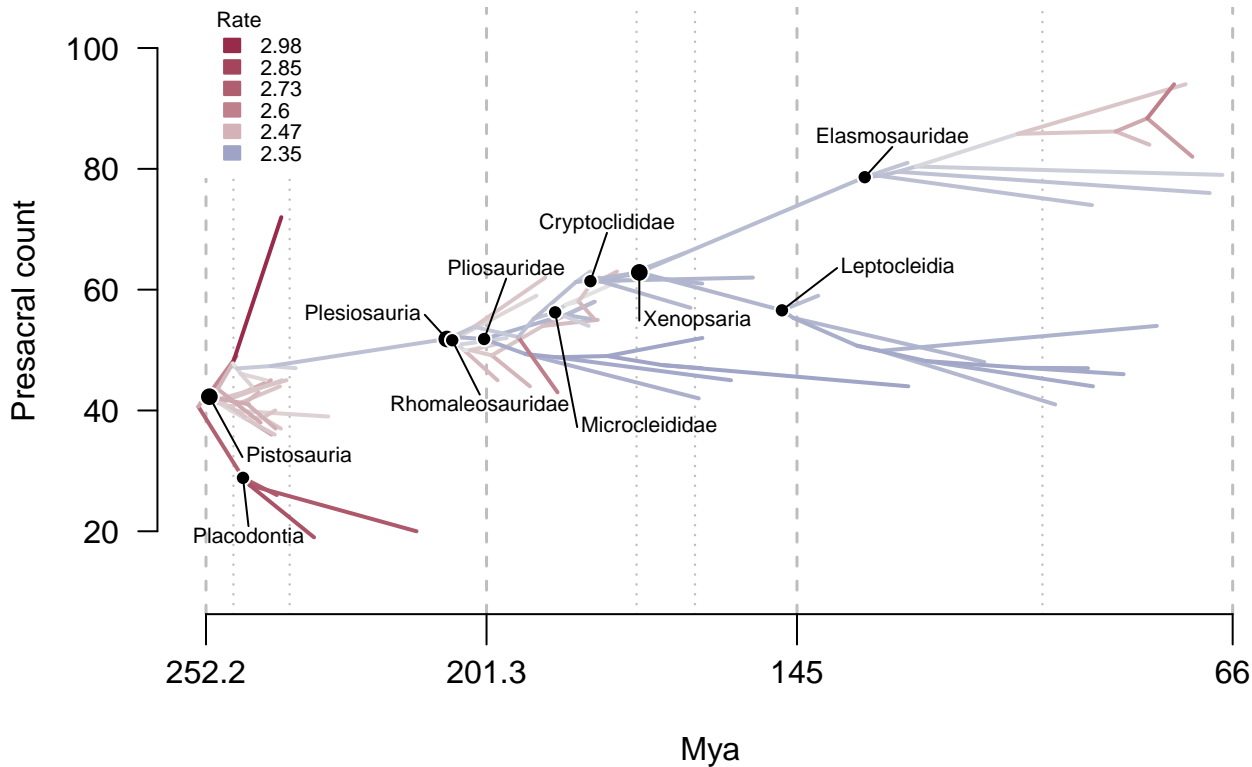

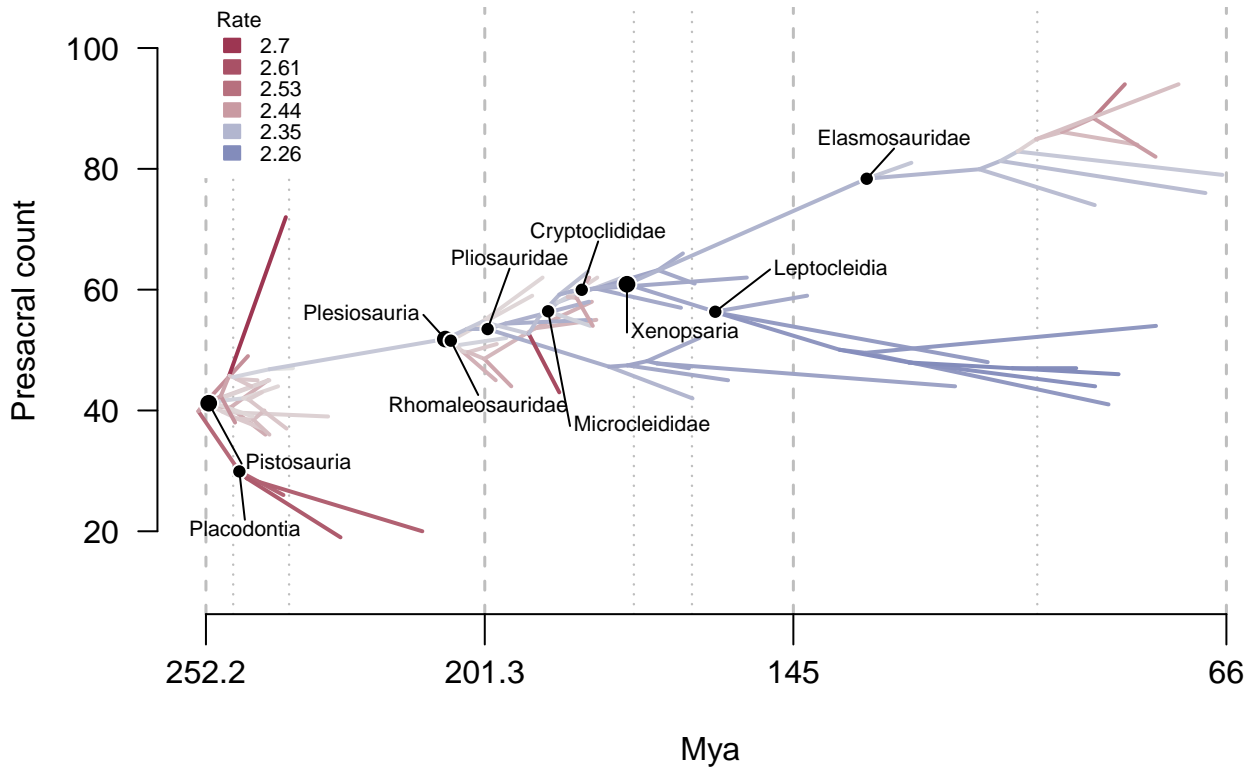

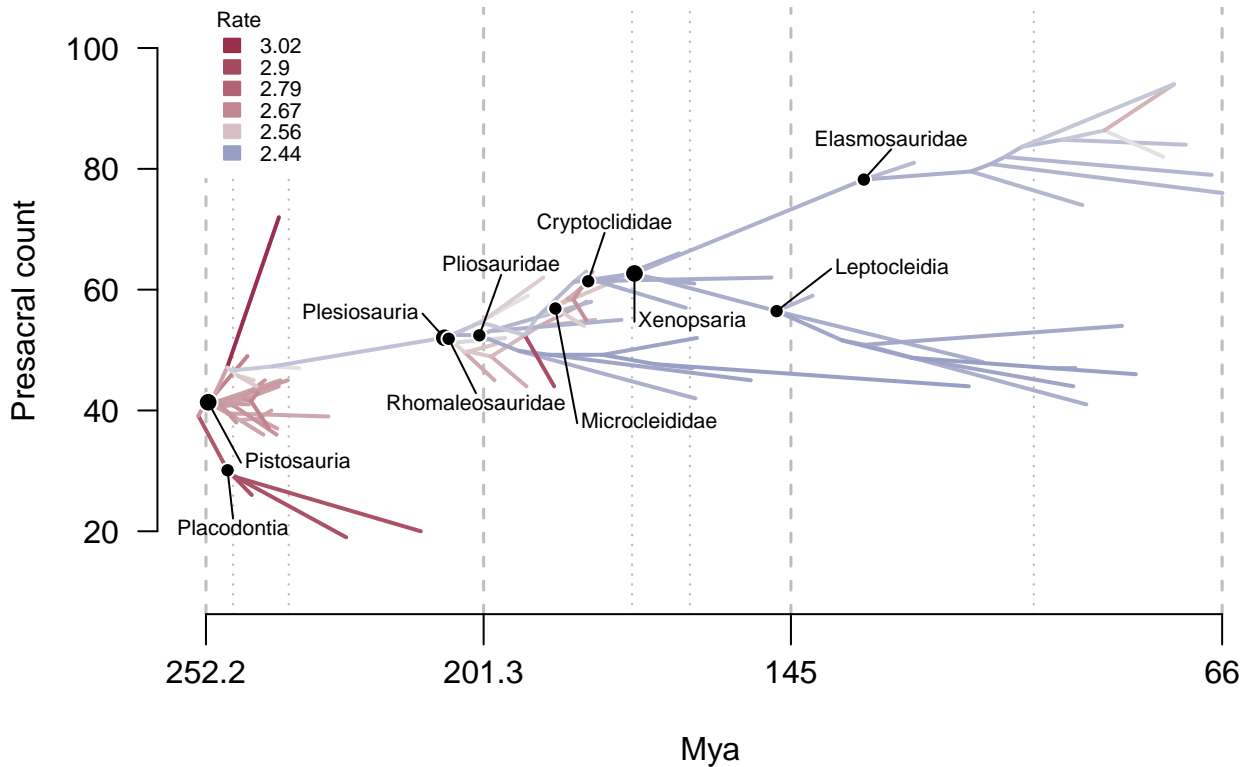

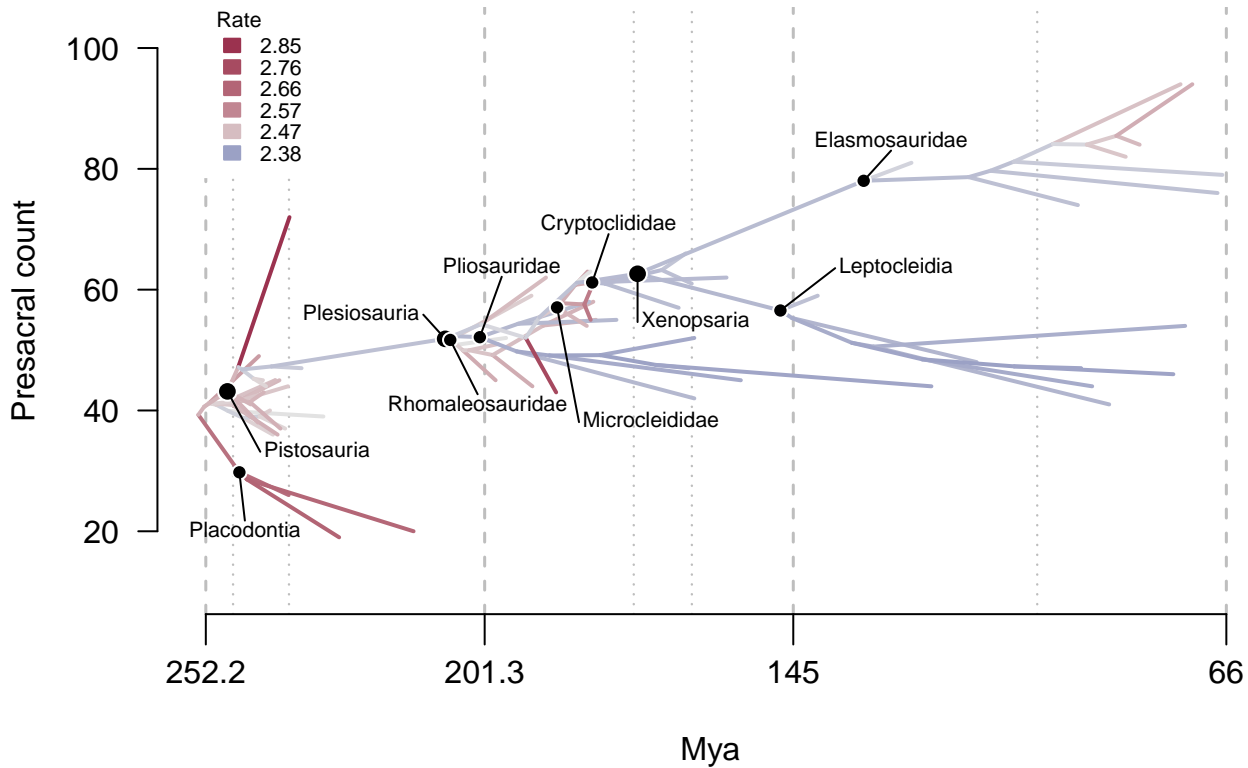

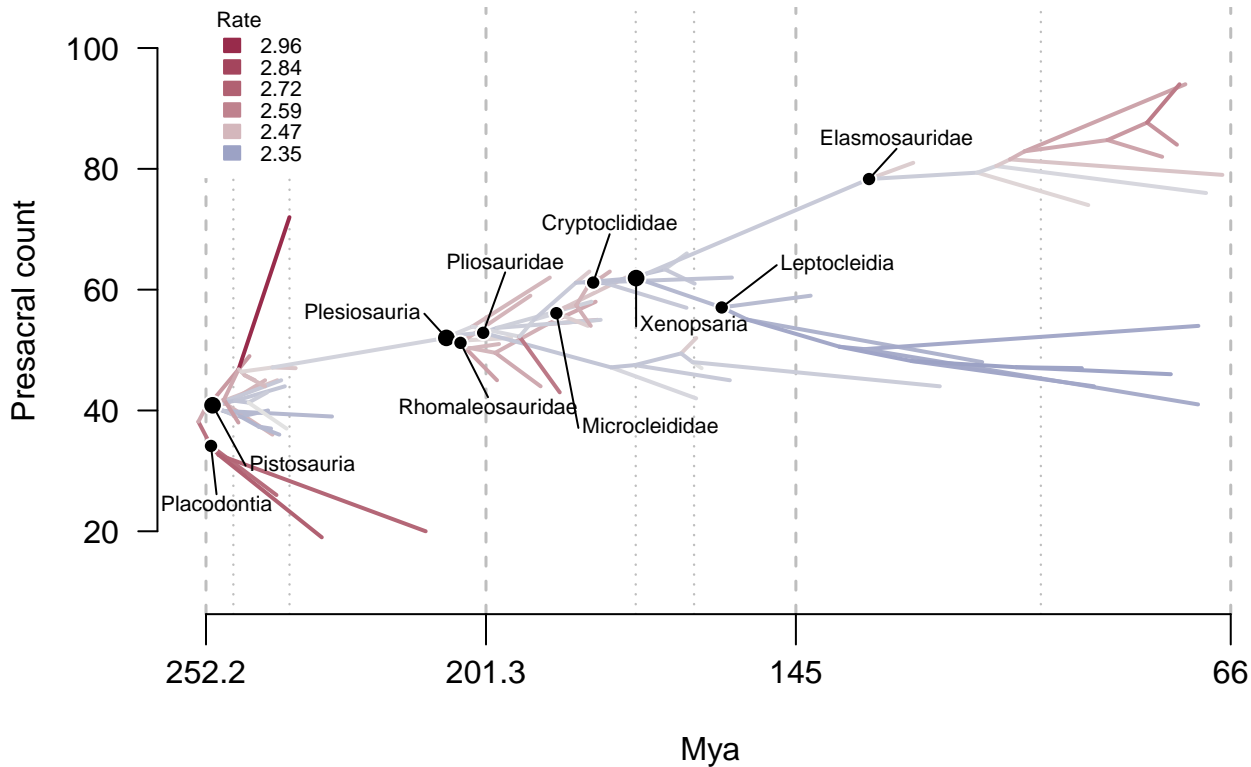

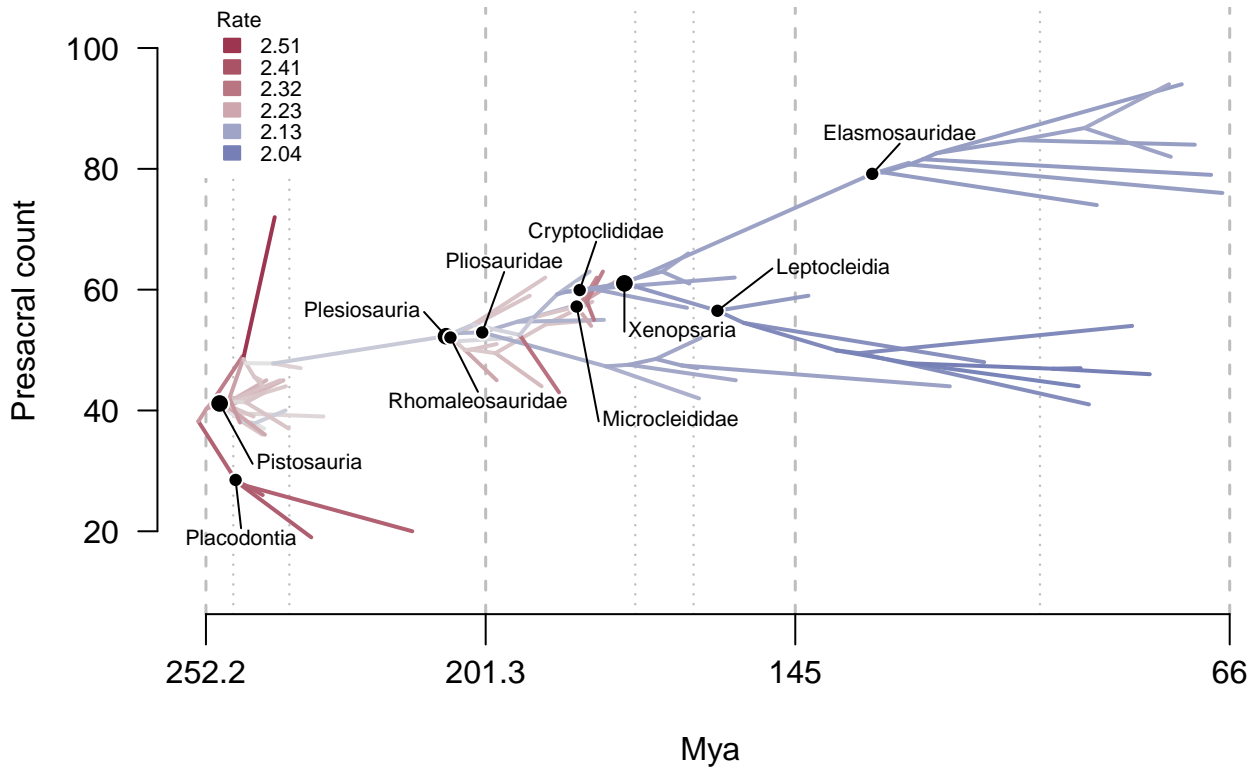

Supplement: Supplementary file 7 — Figure S7. [file EVO-71-1164-s007.pdf]

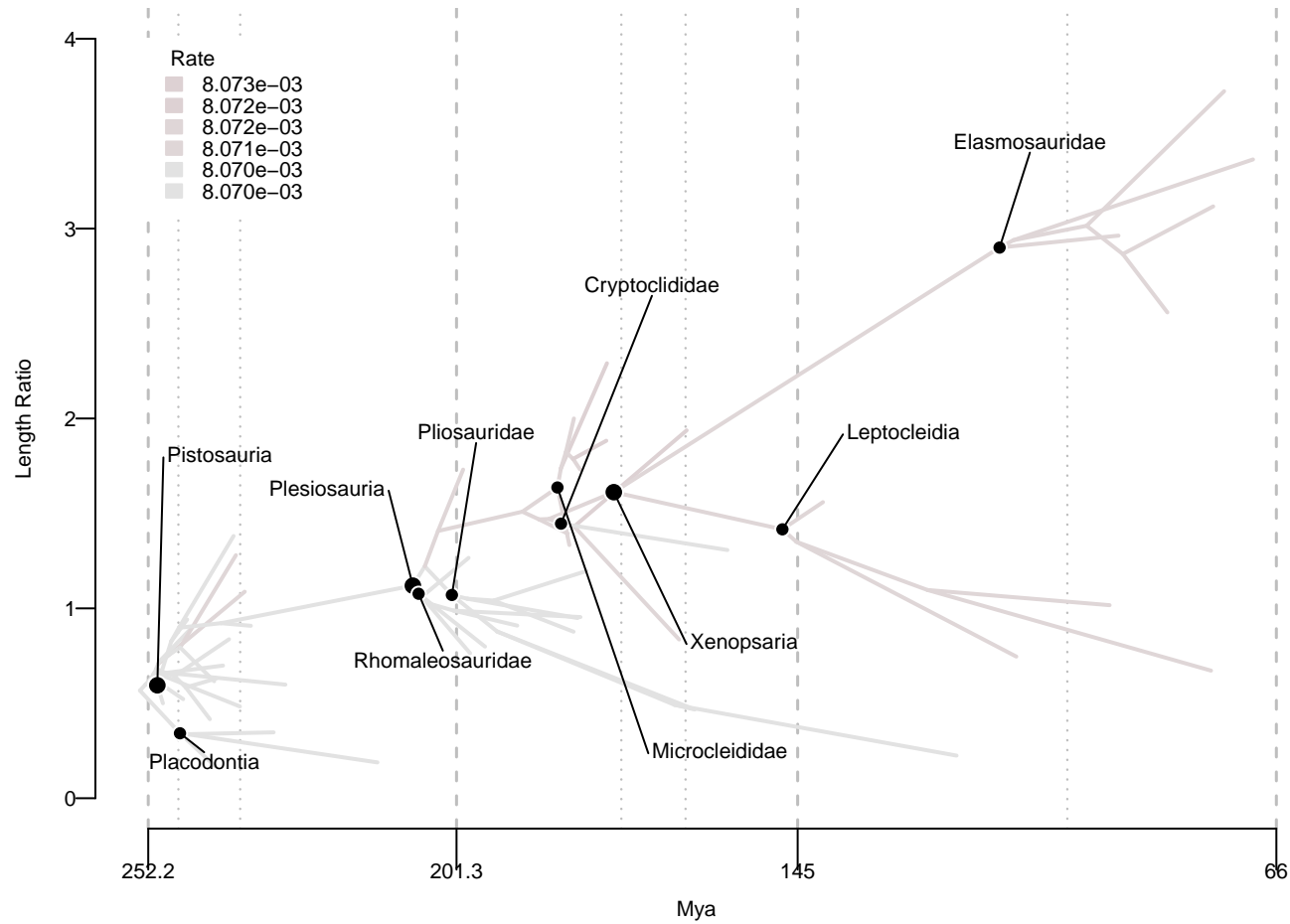

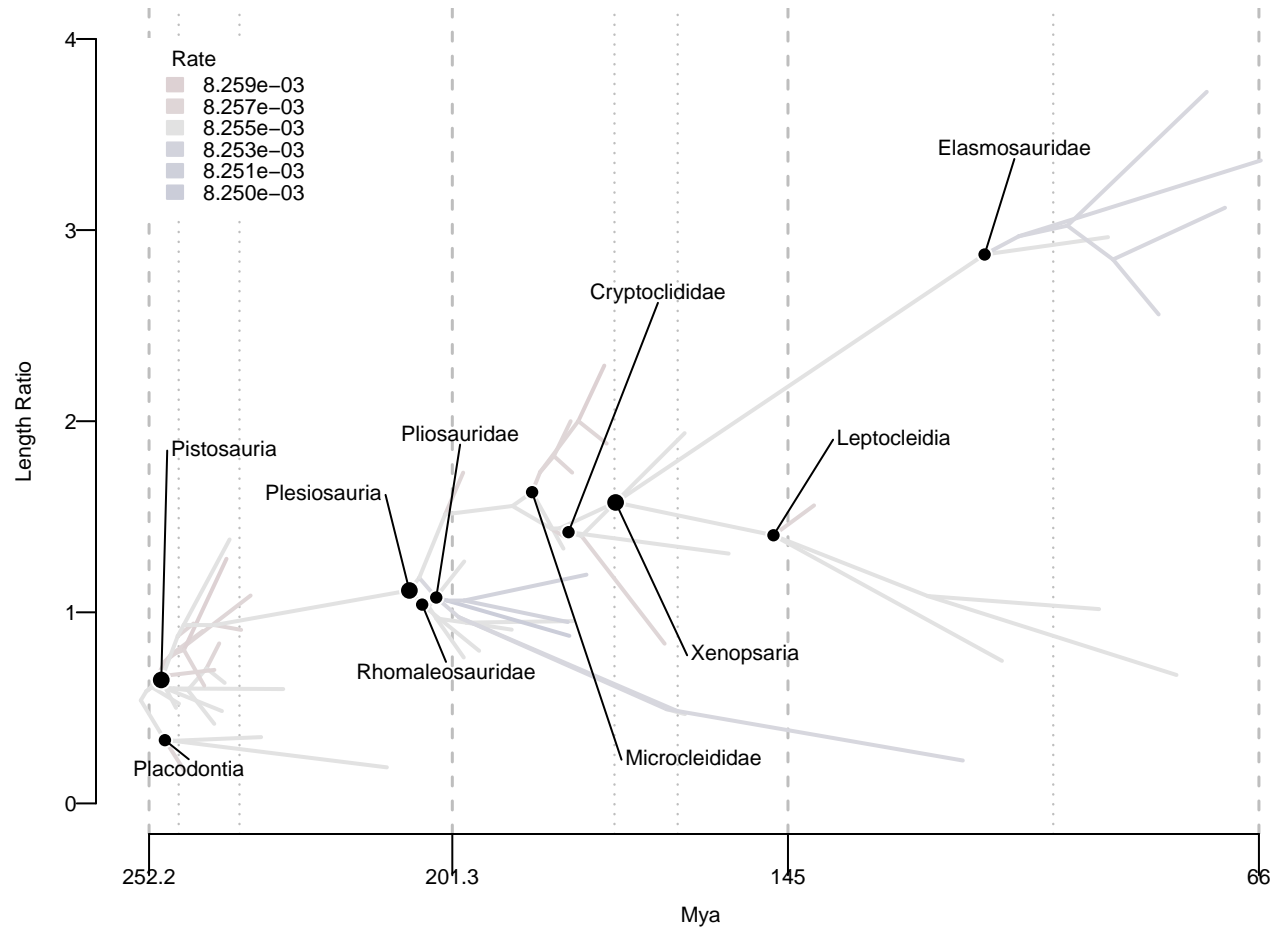

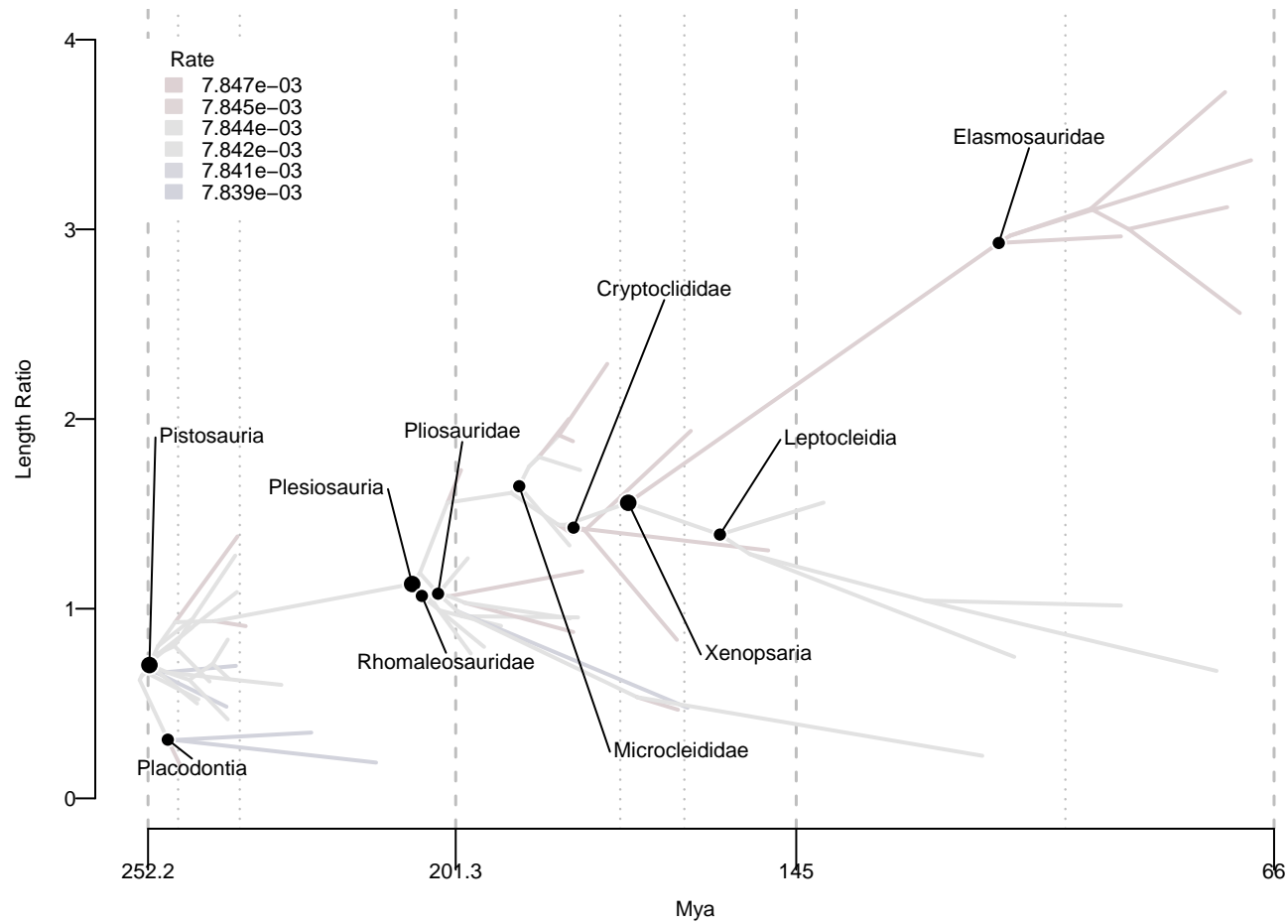

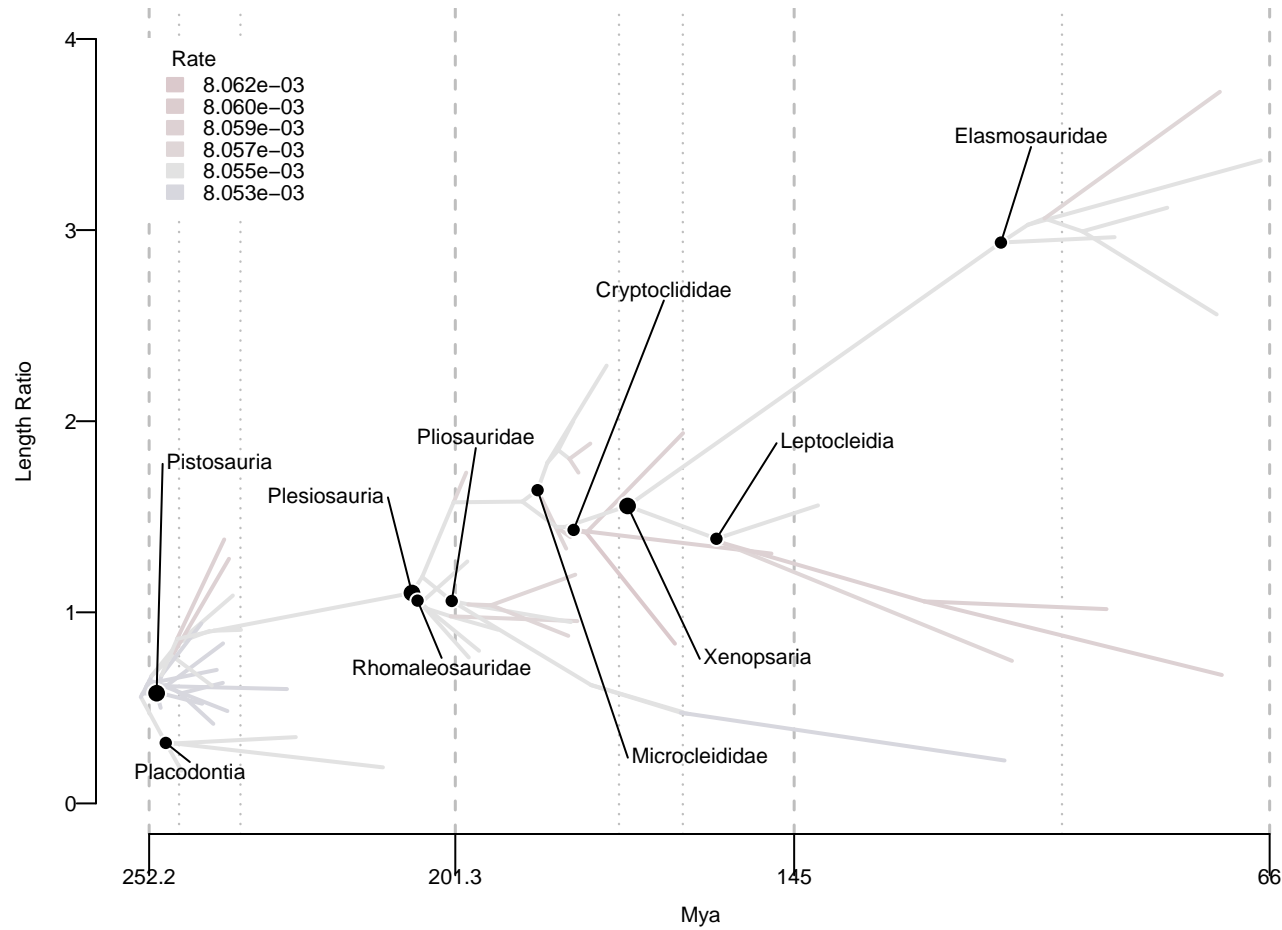

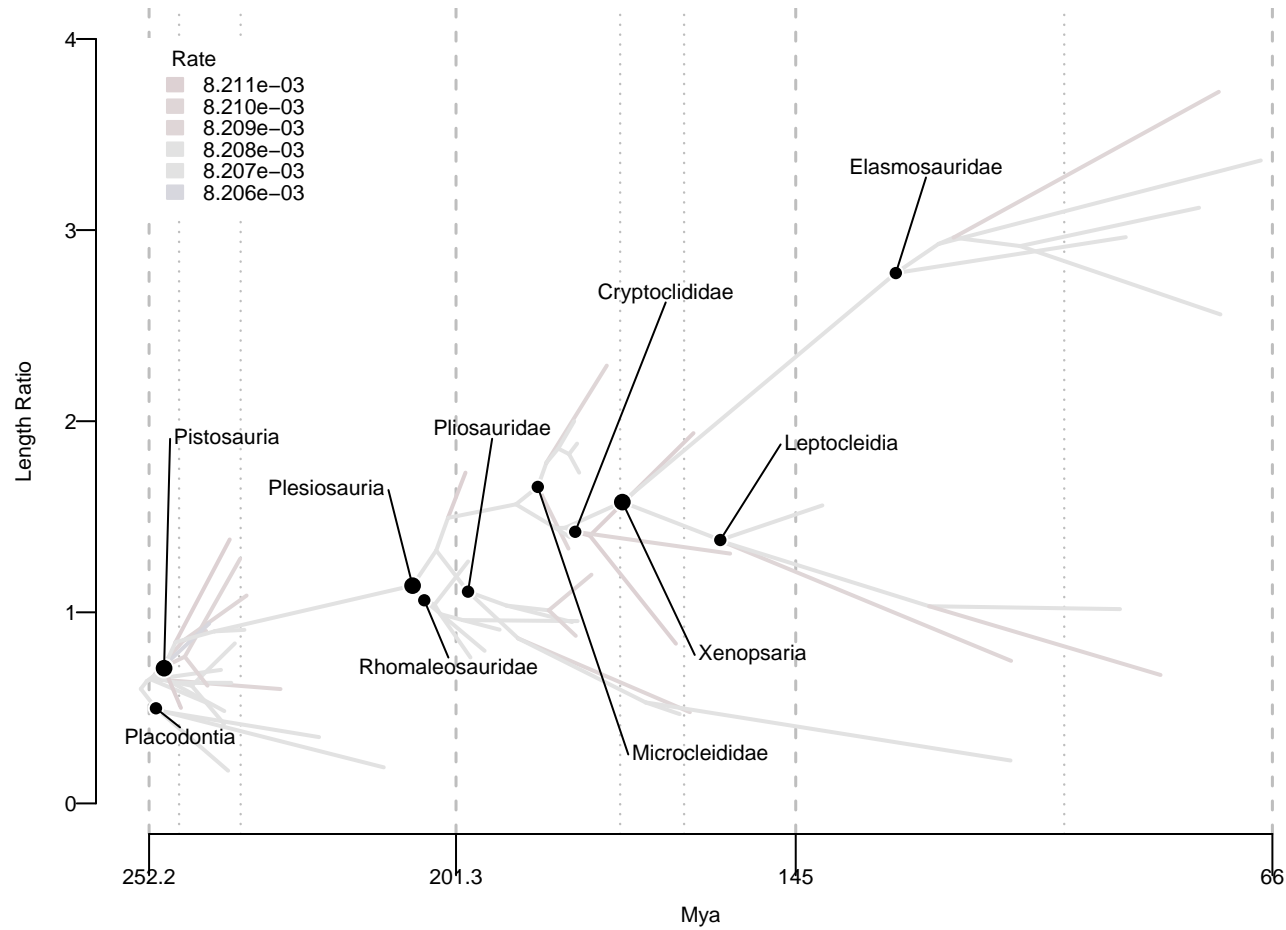

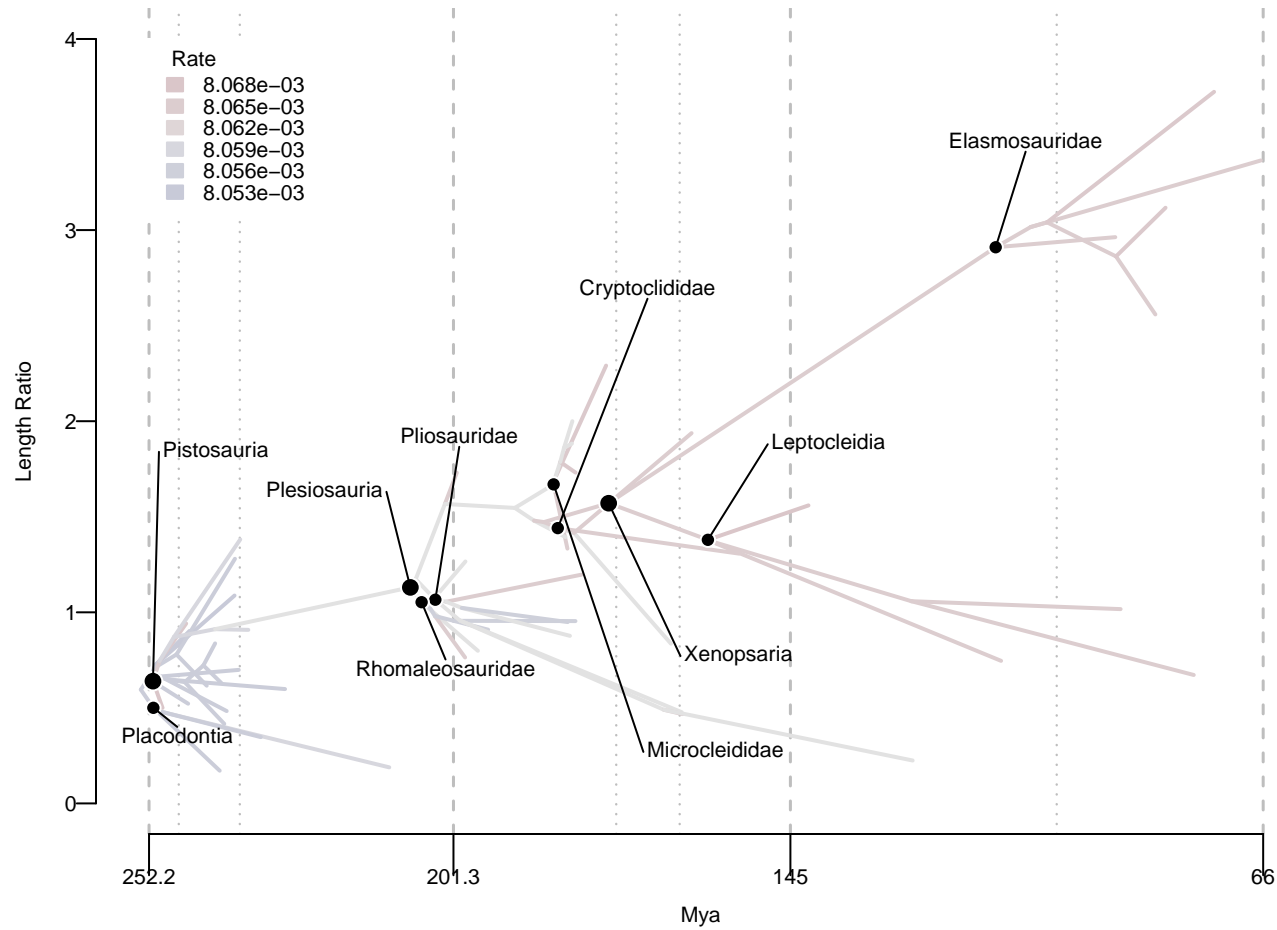

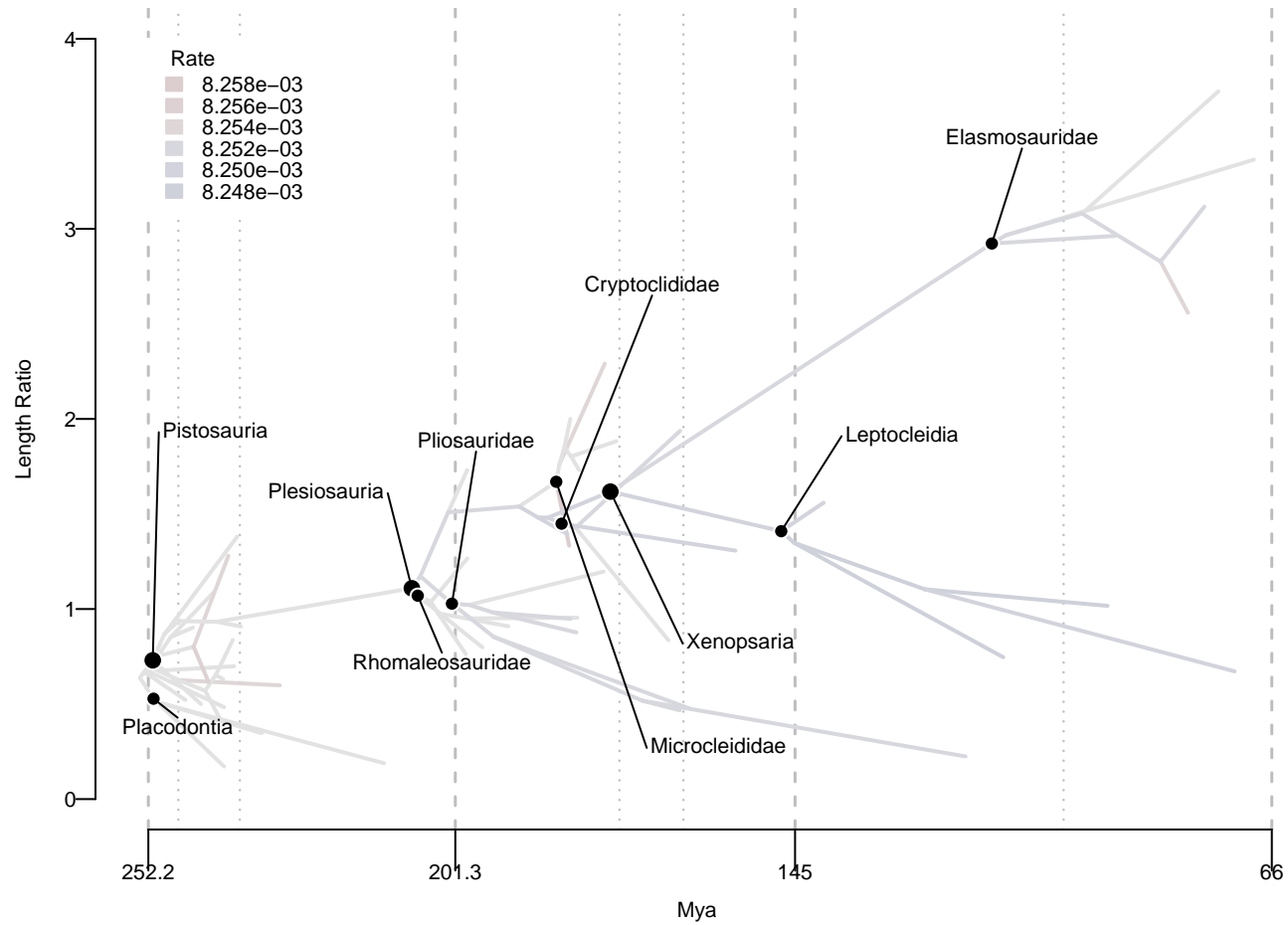

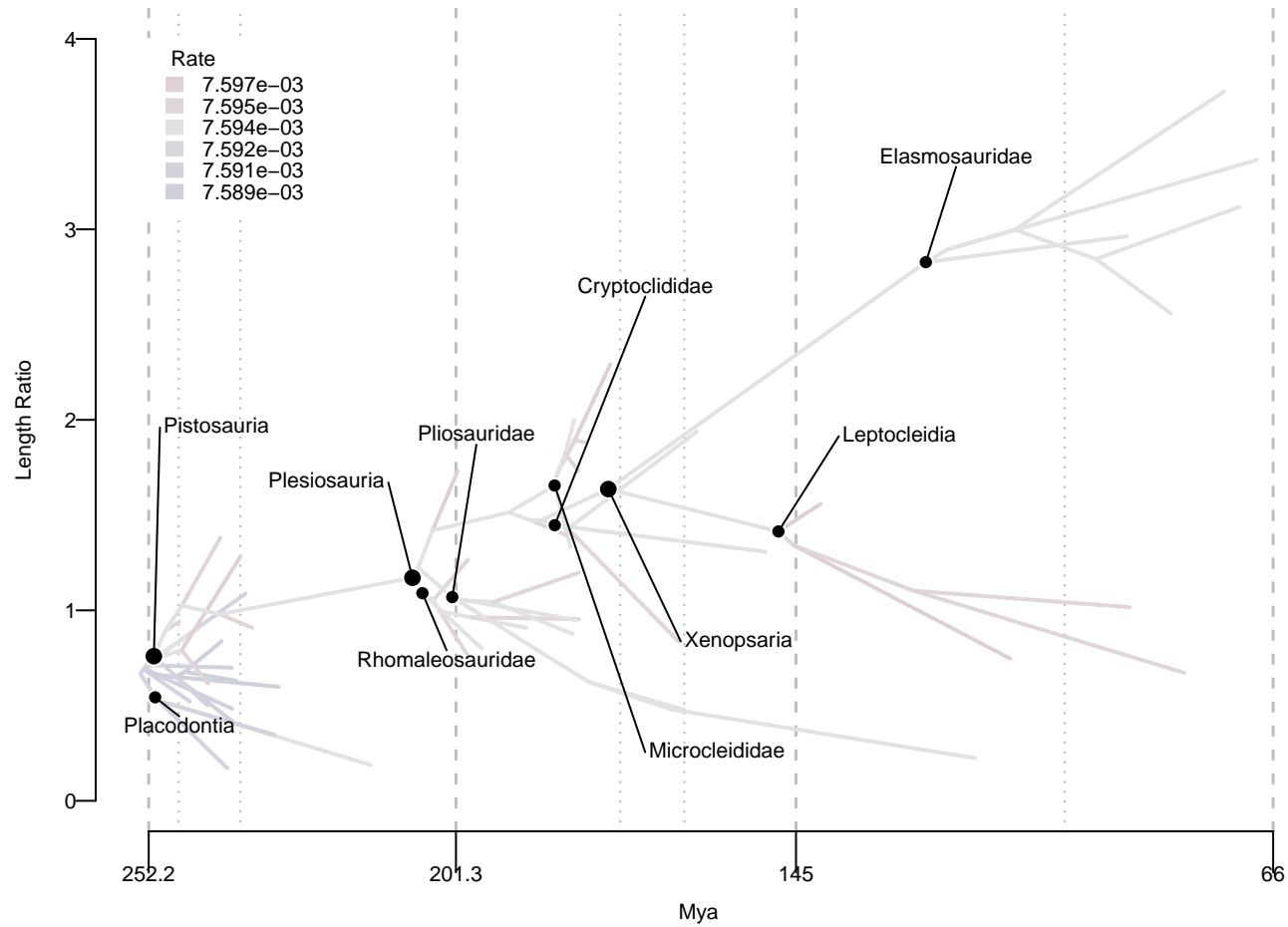

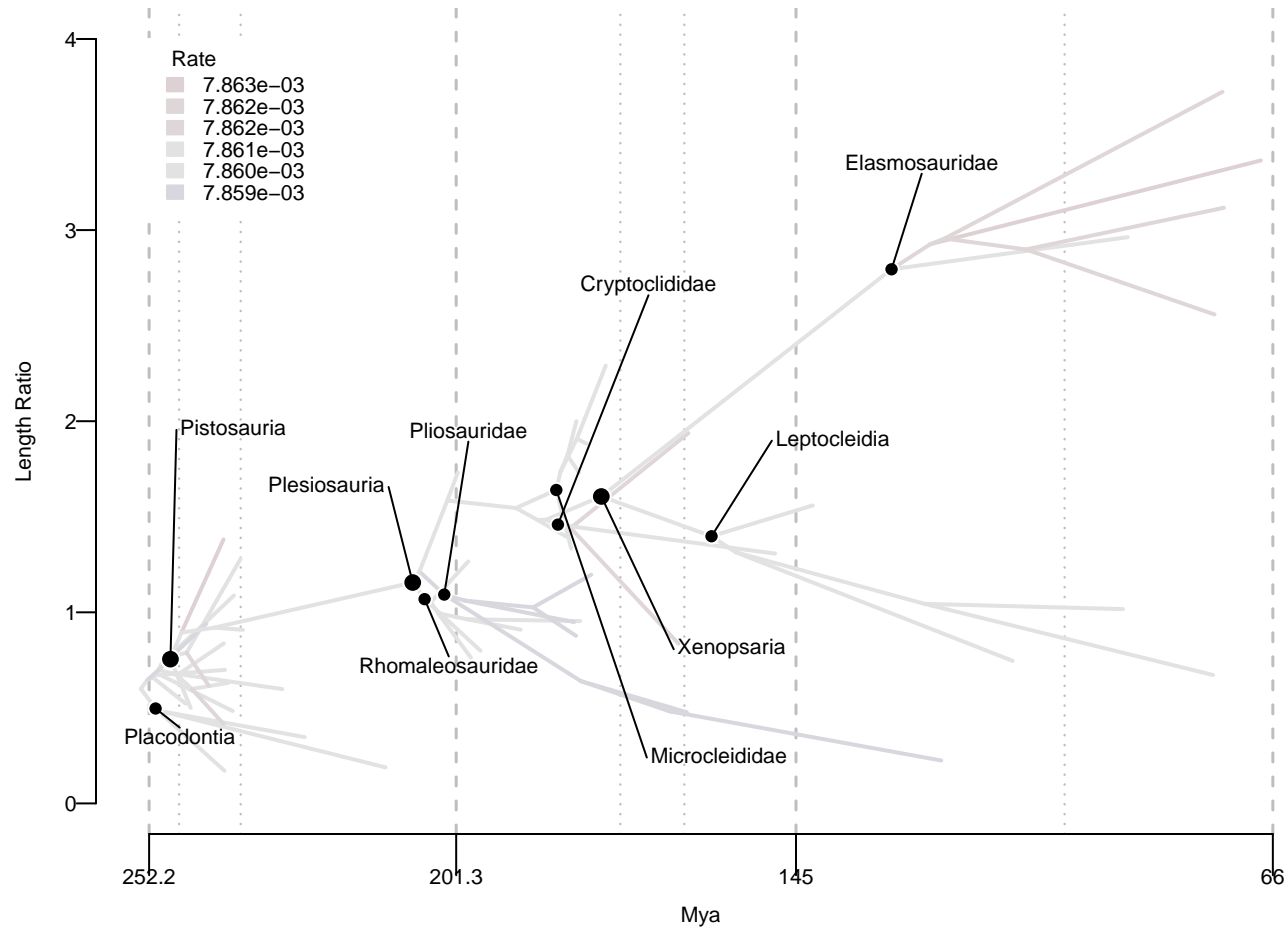

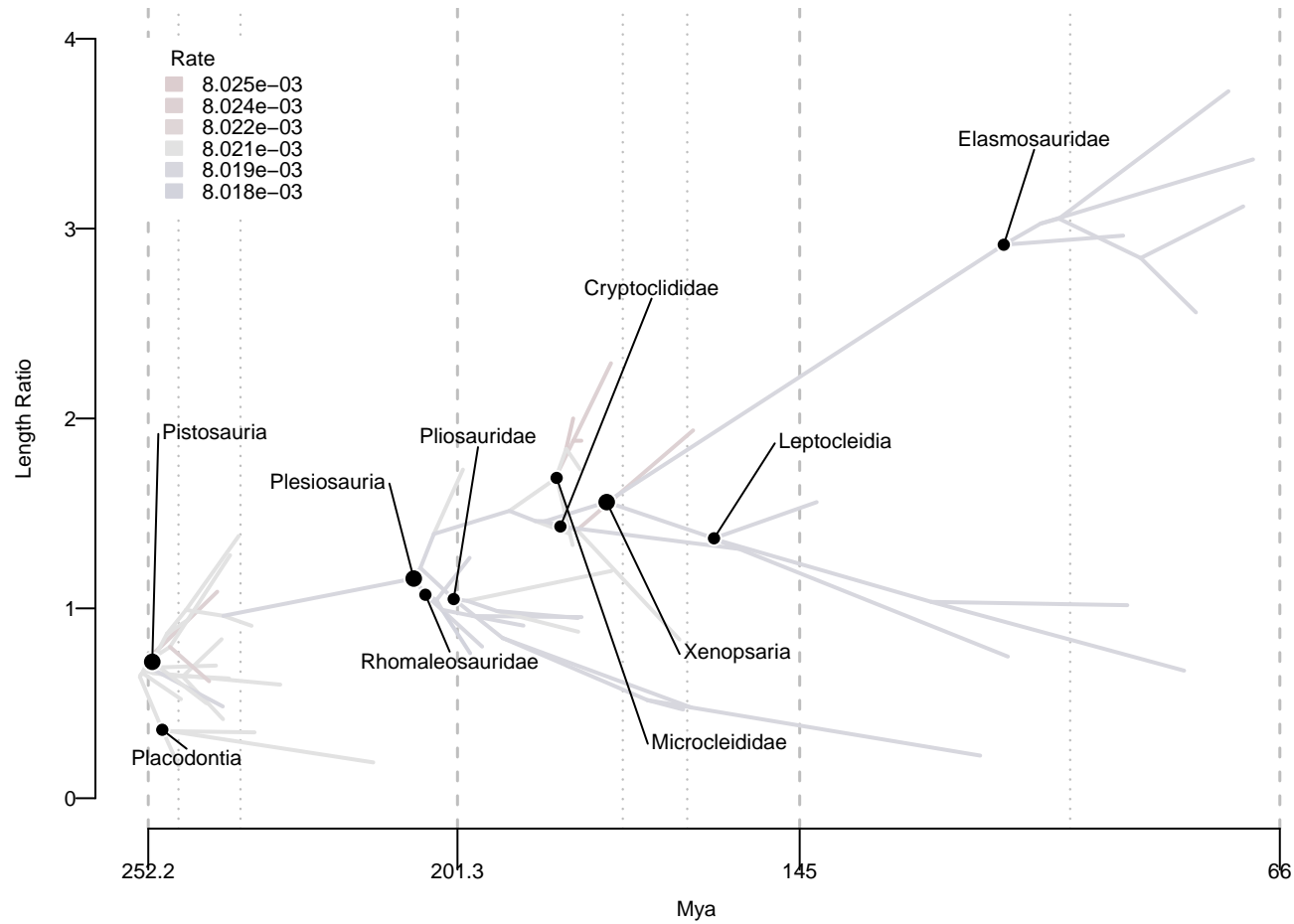

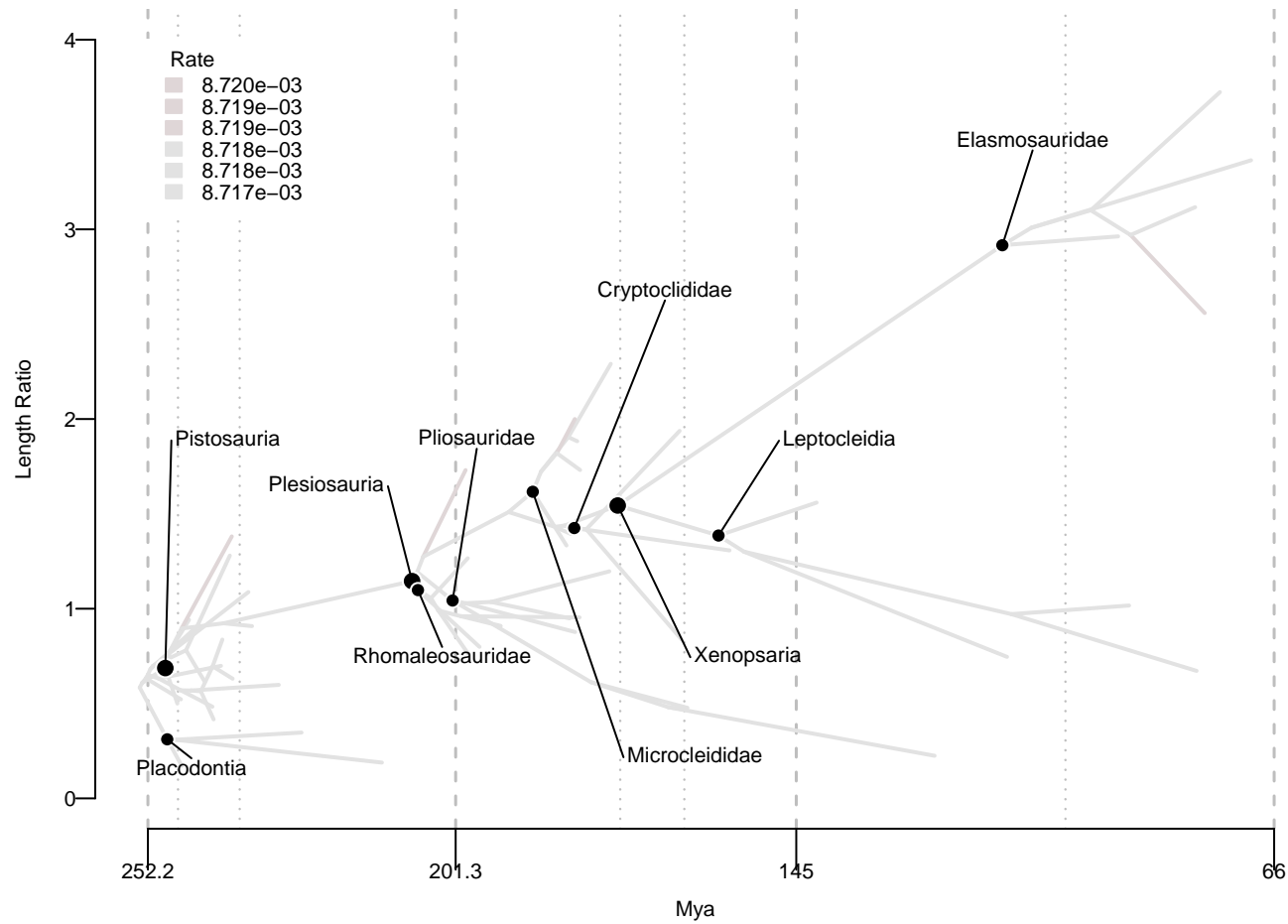

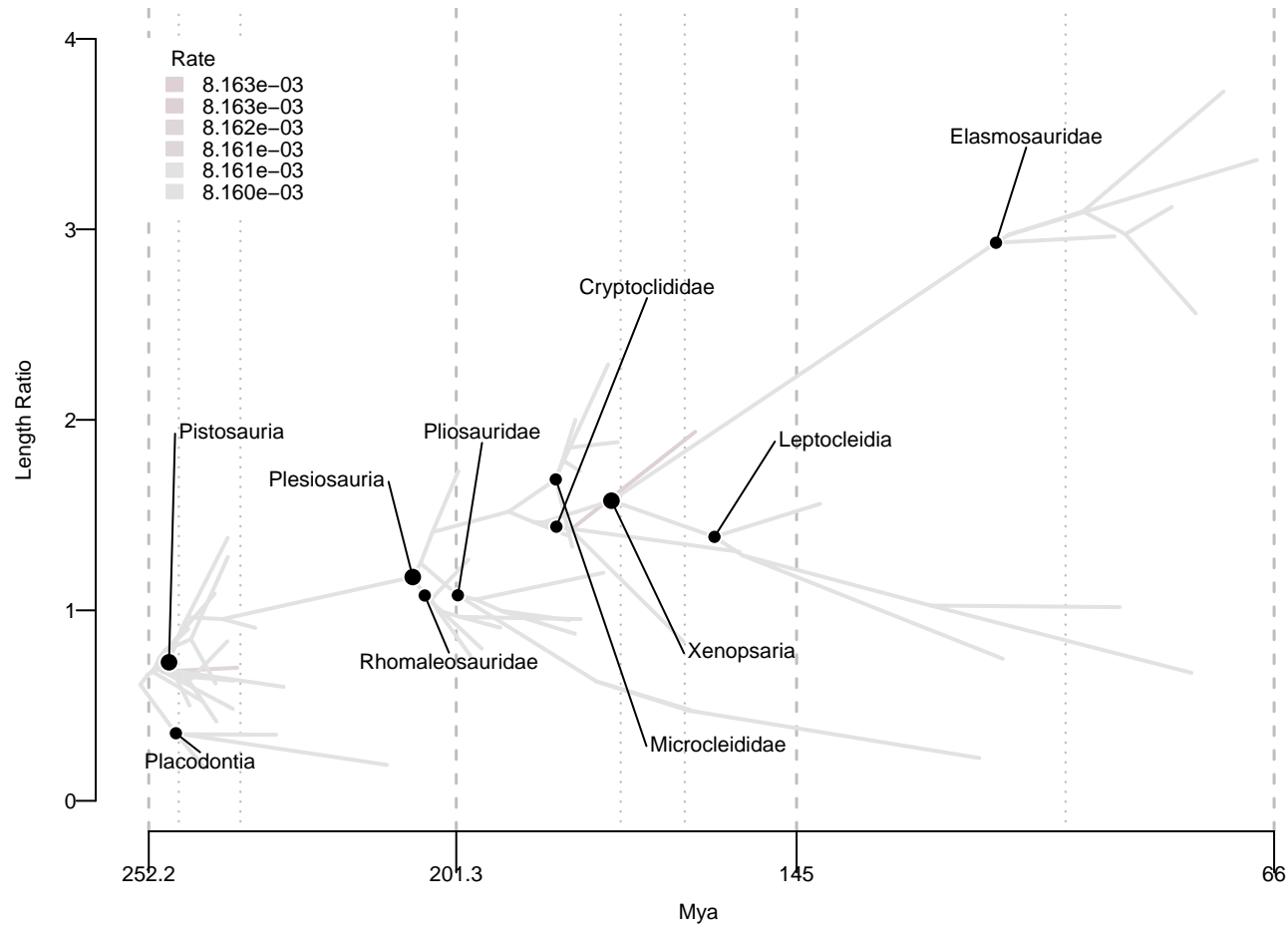

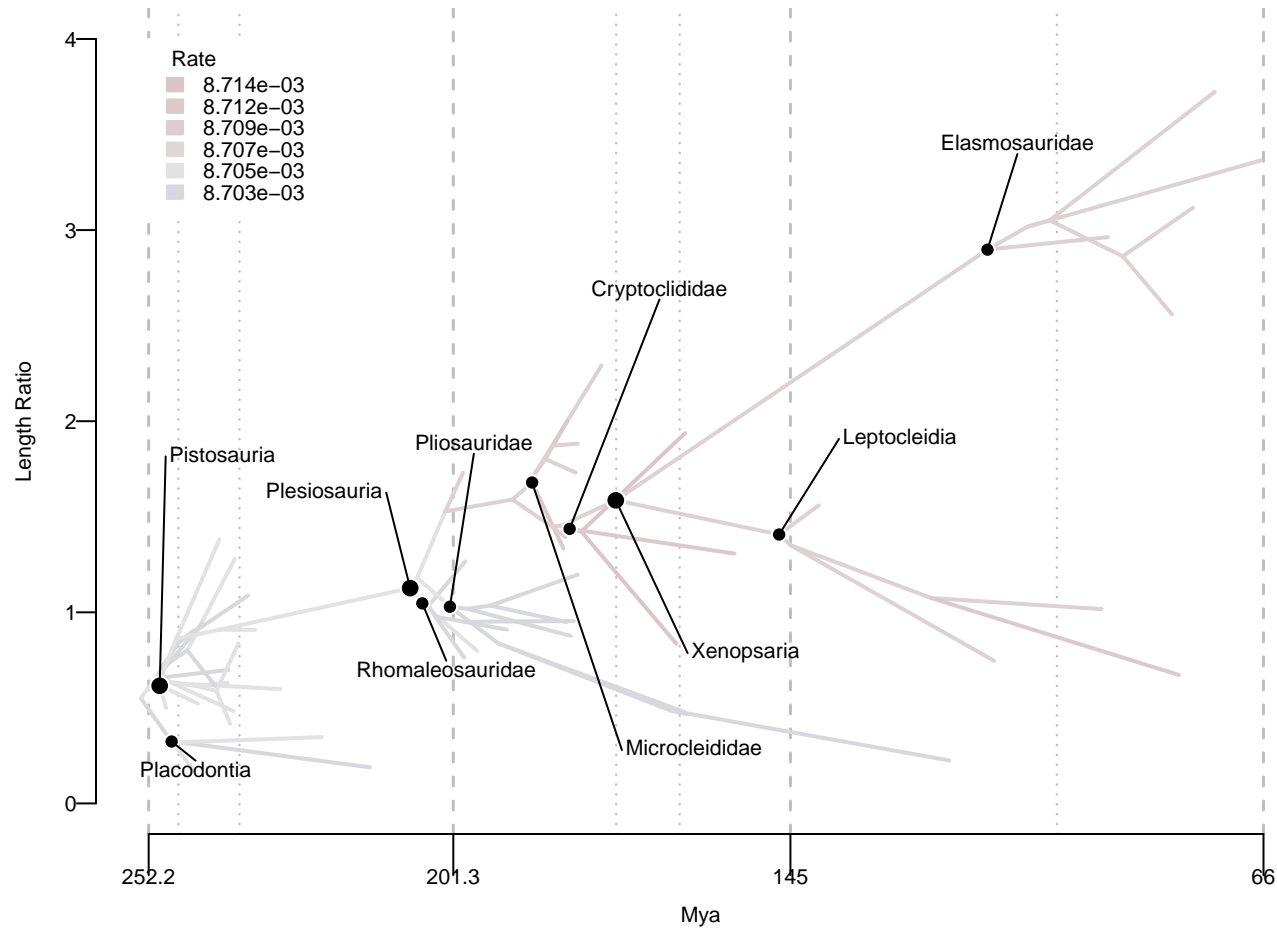

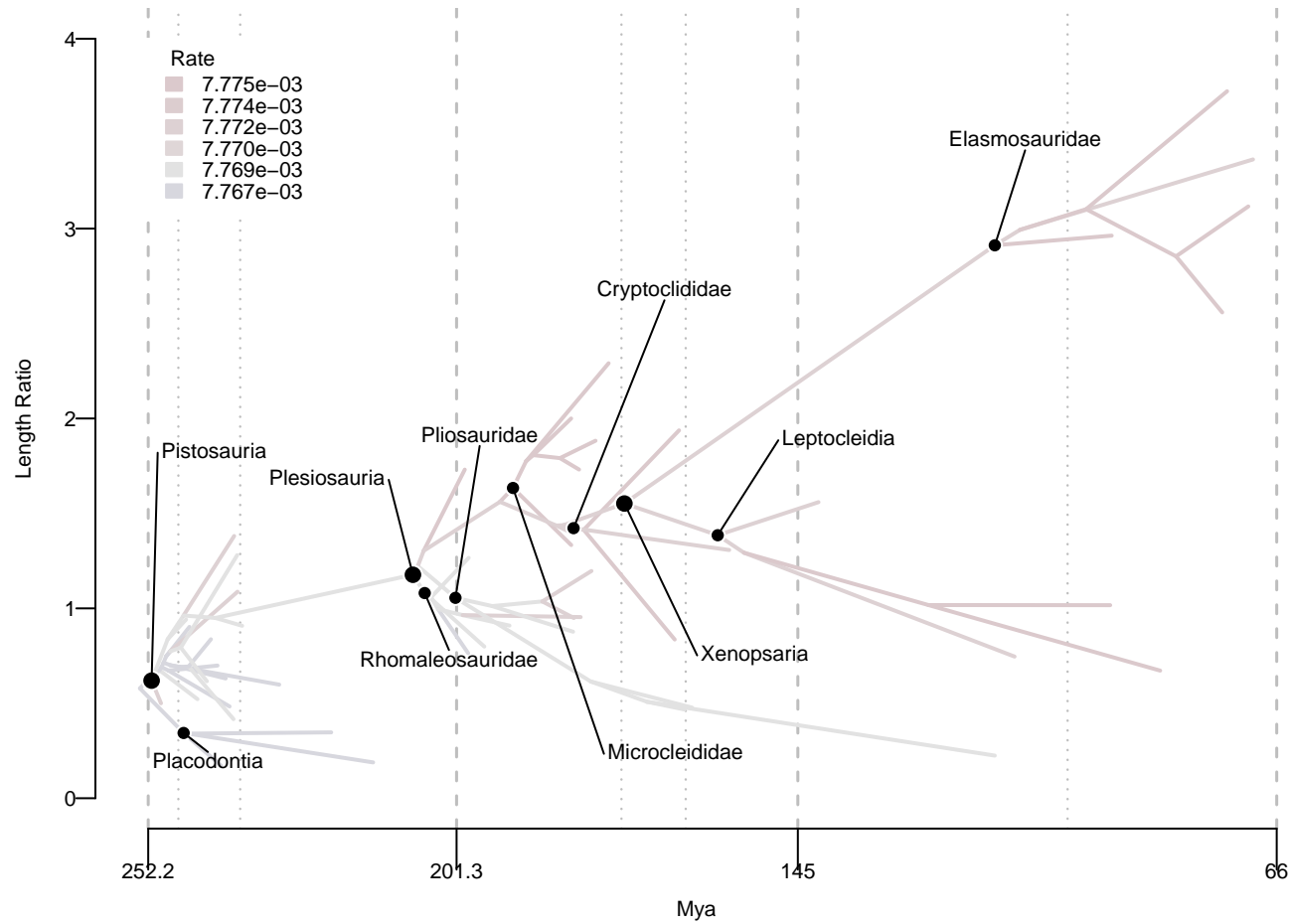

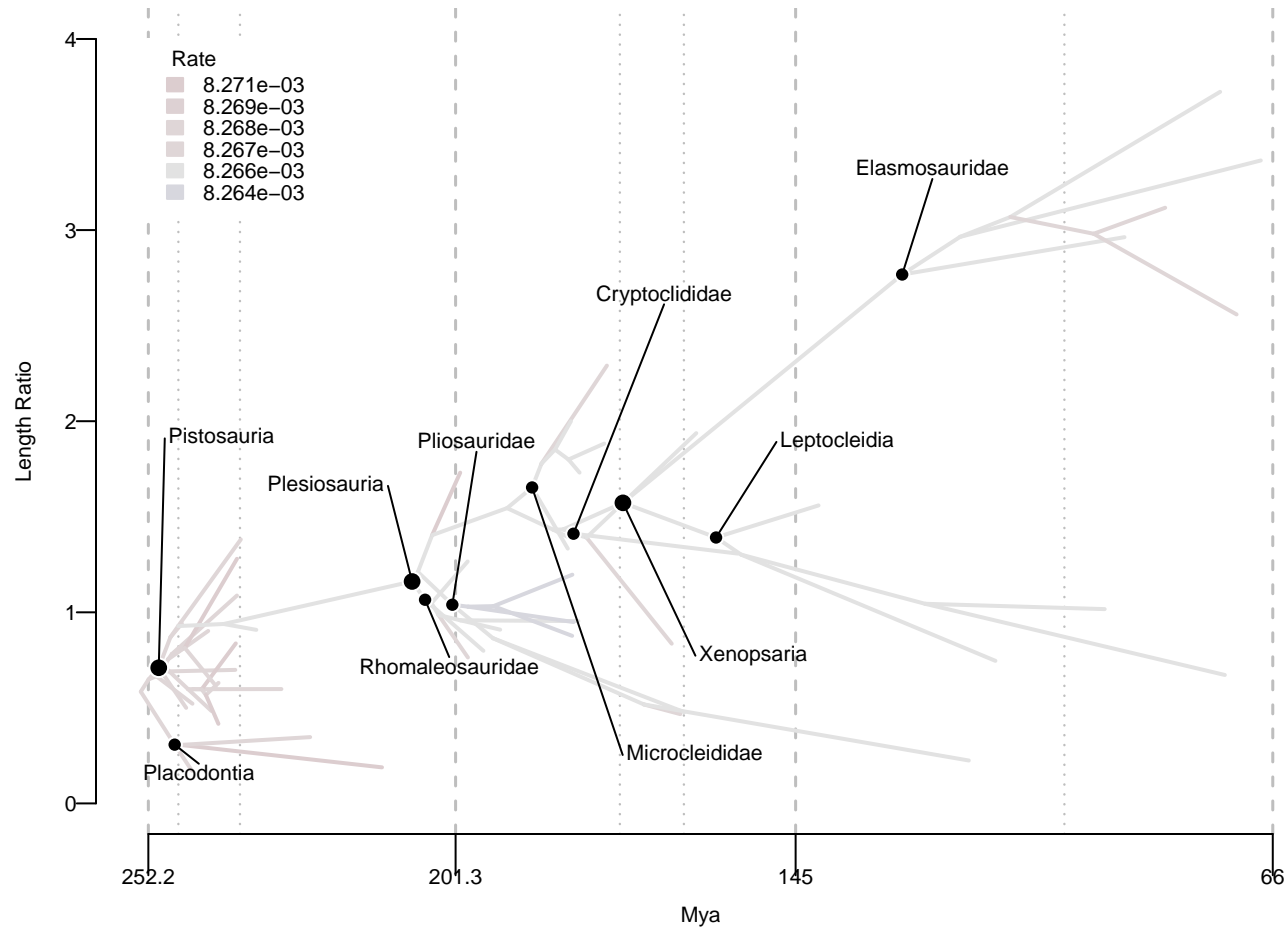

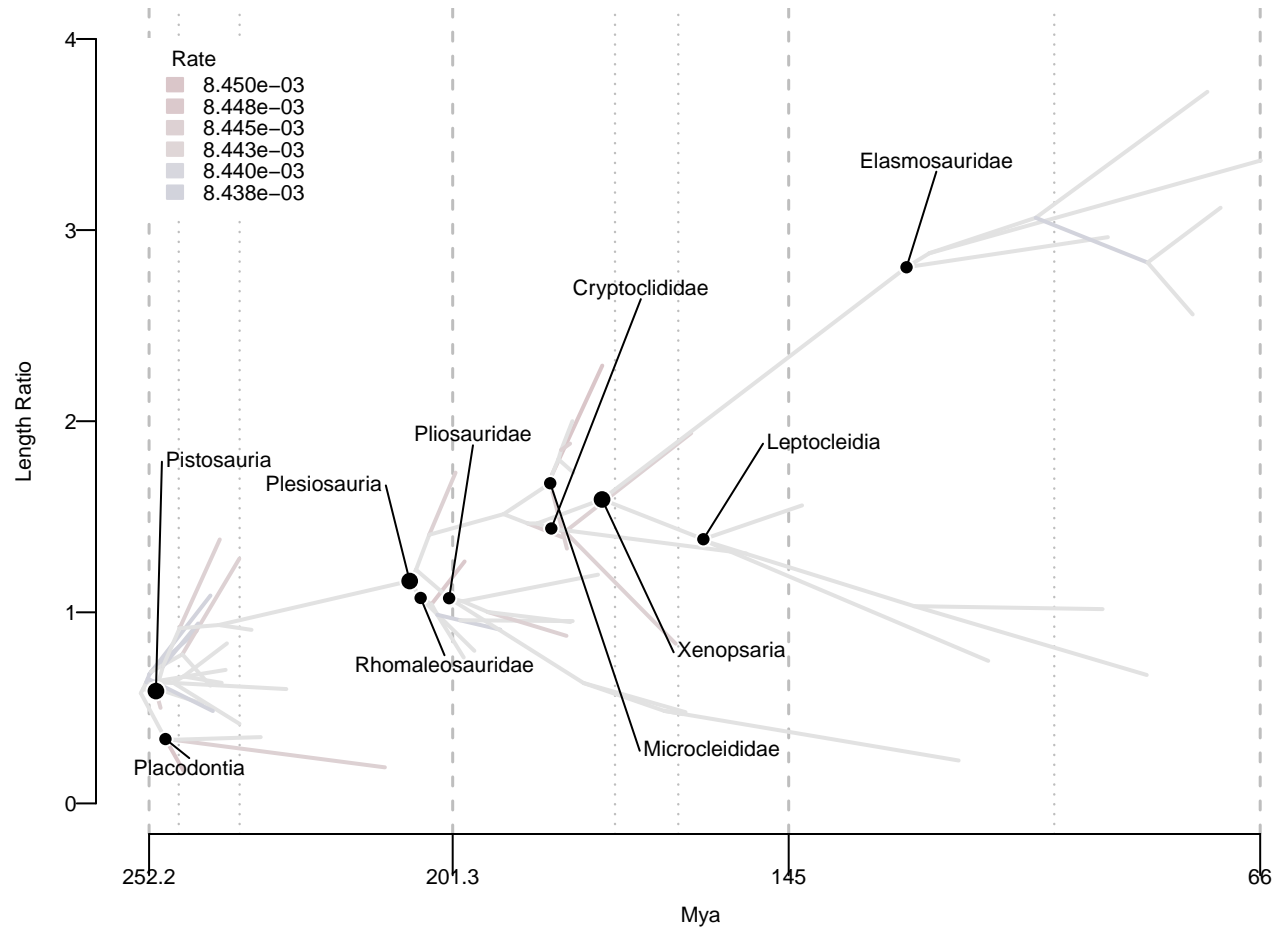

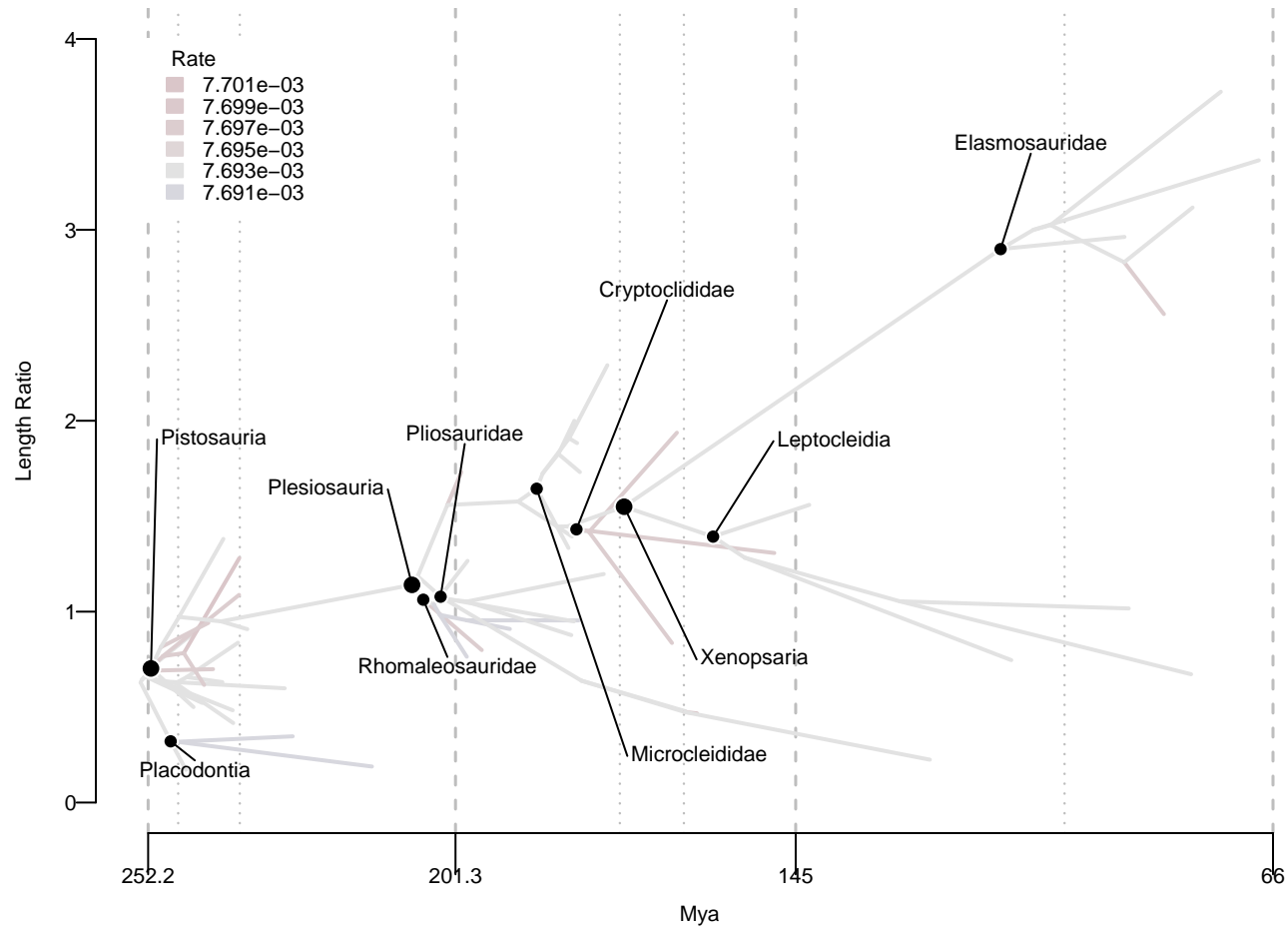

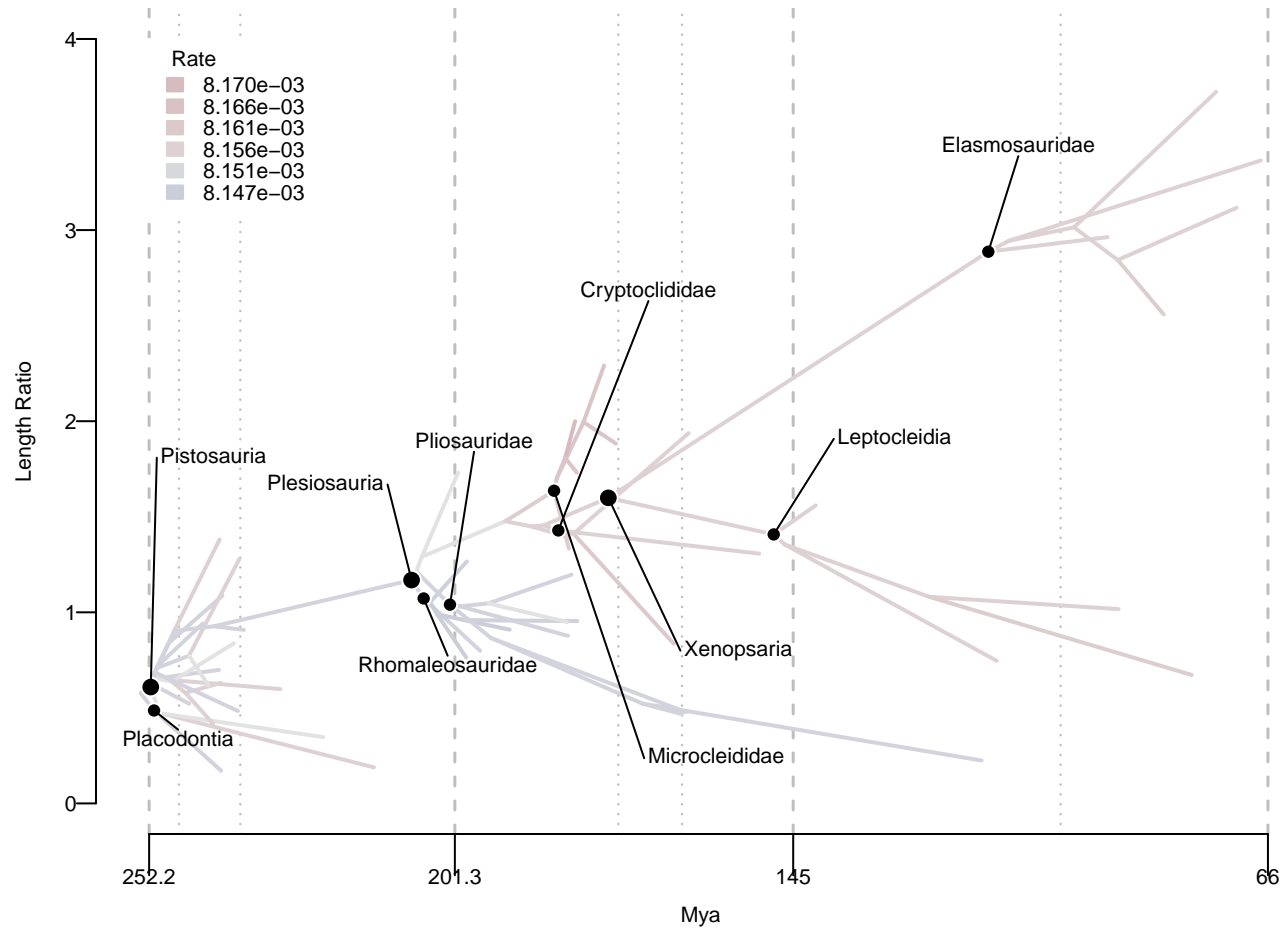

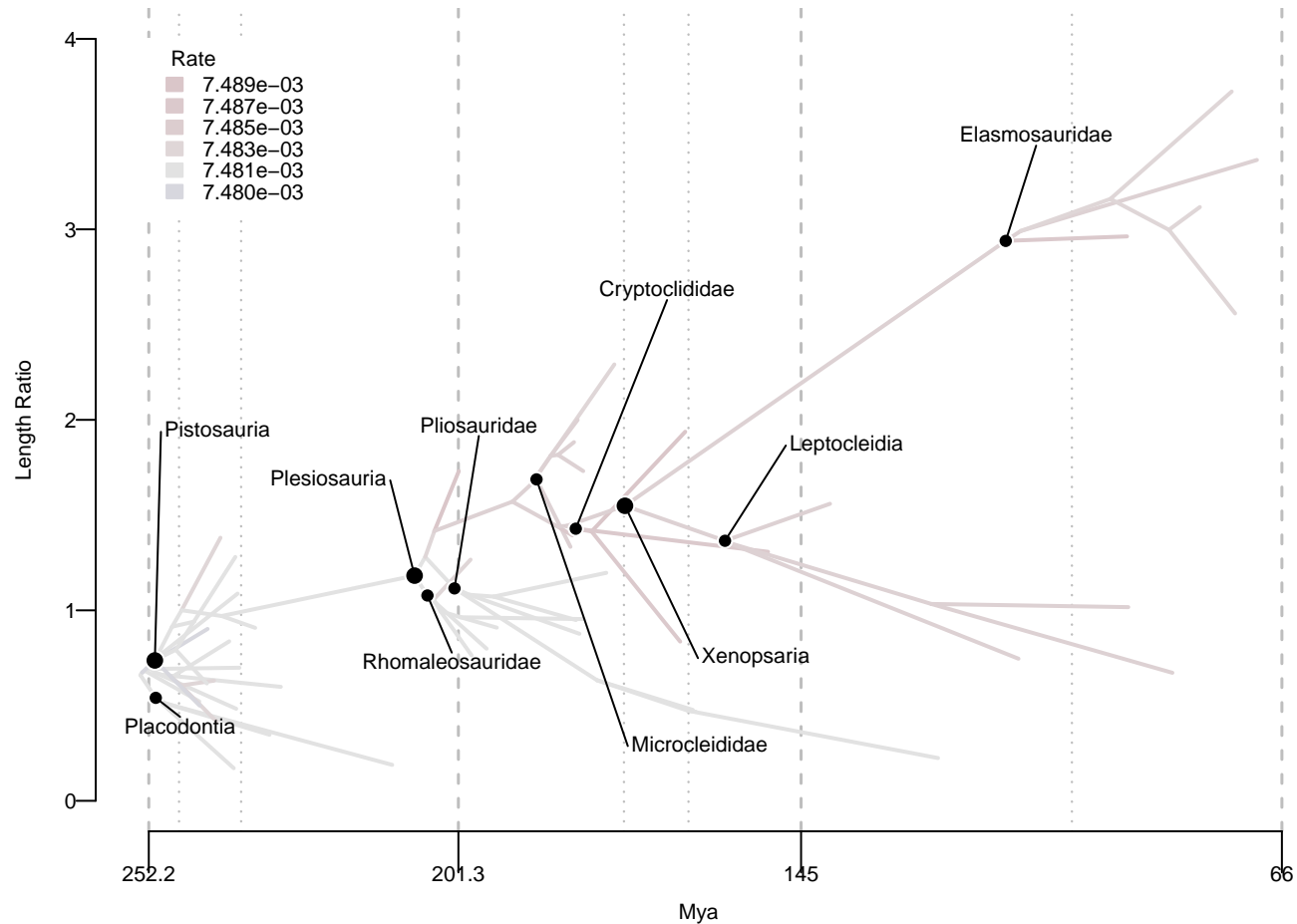

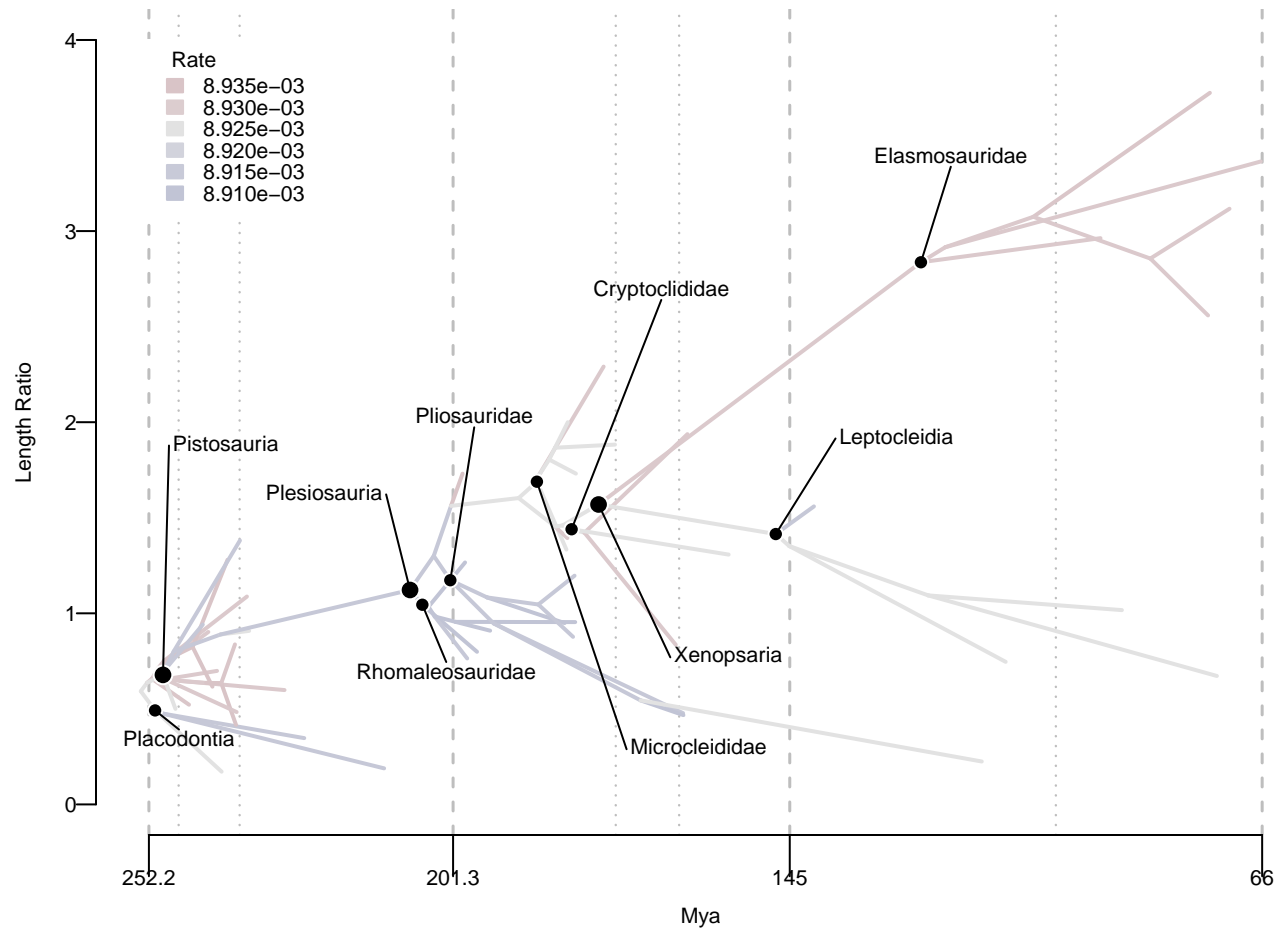

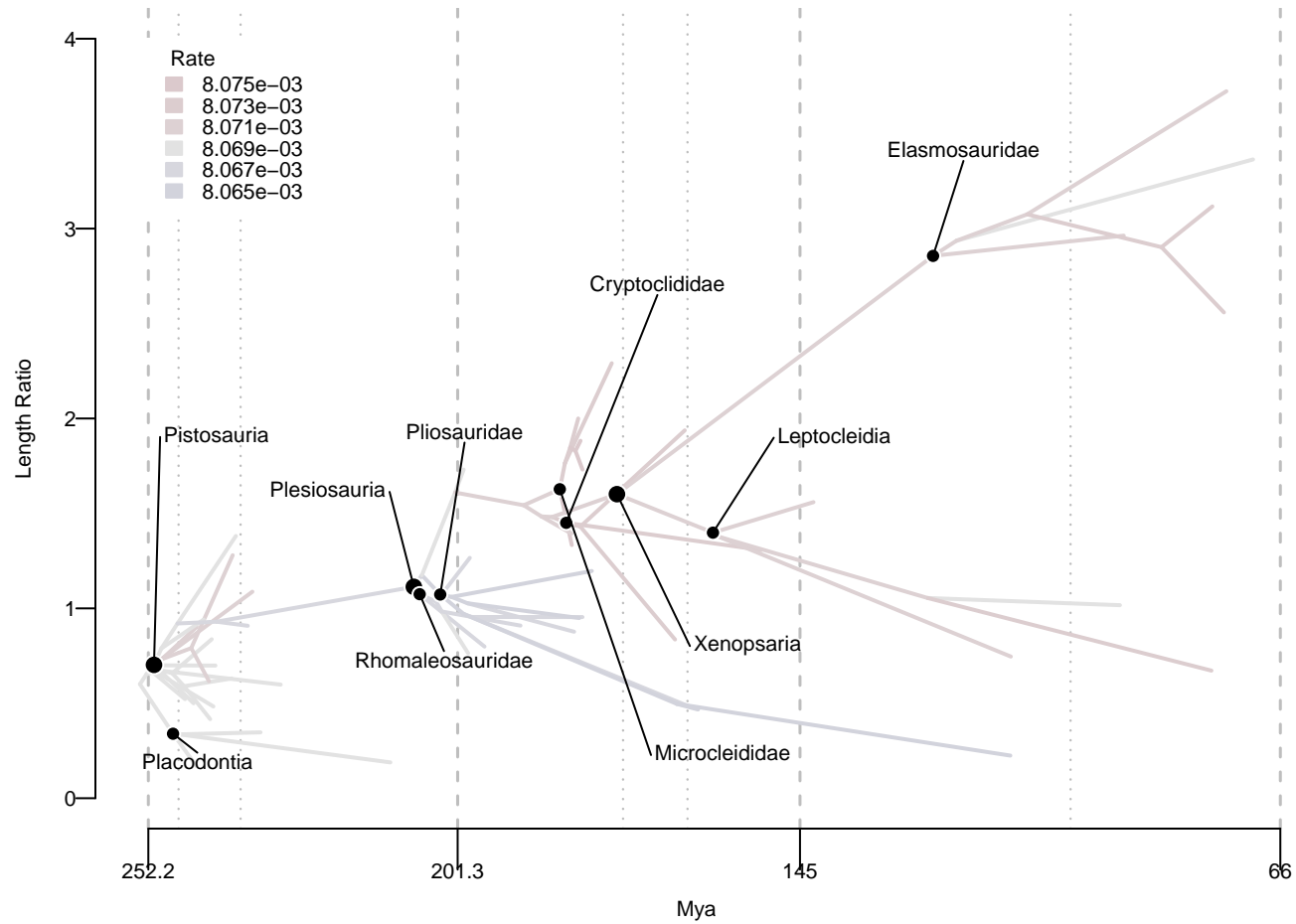

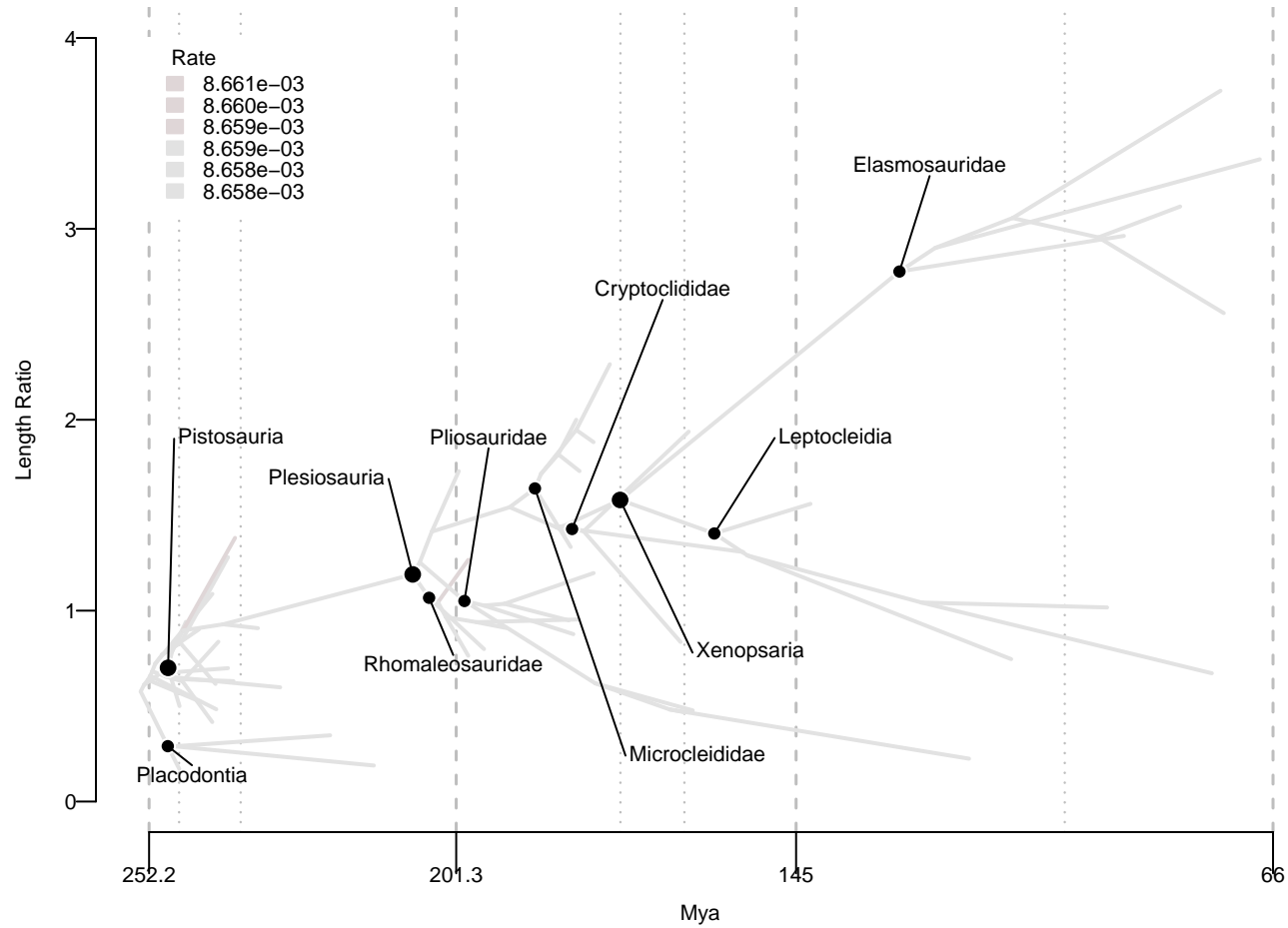

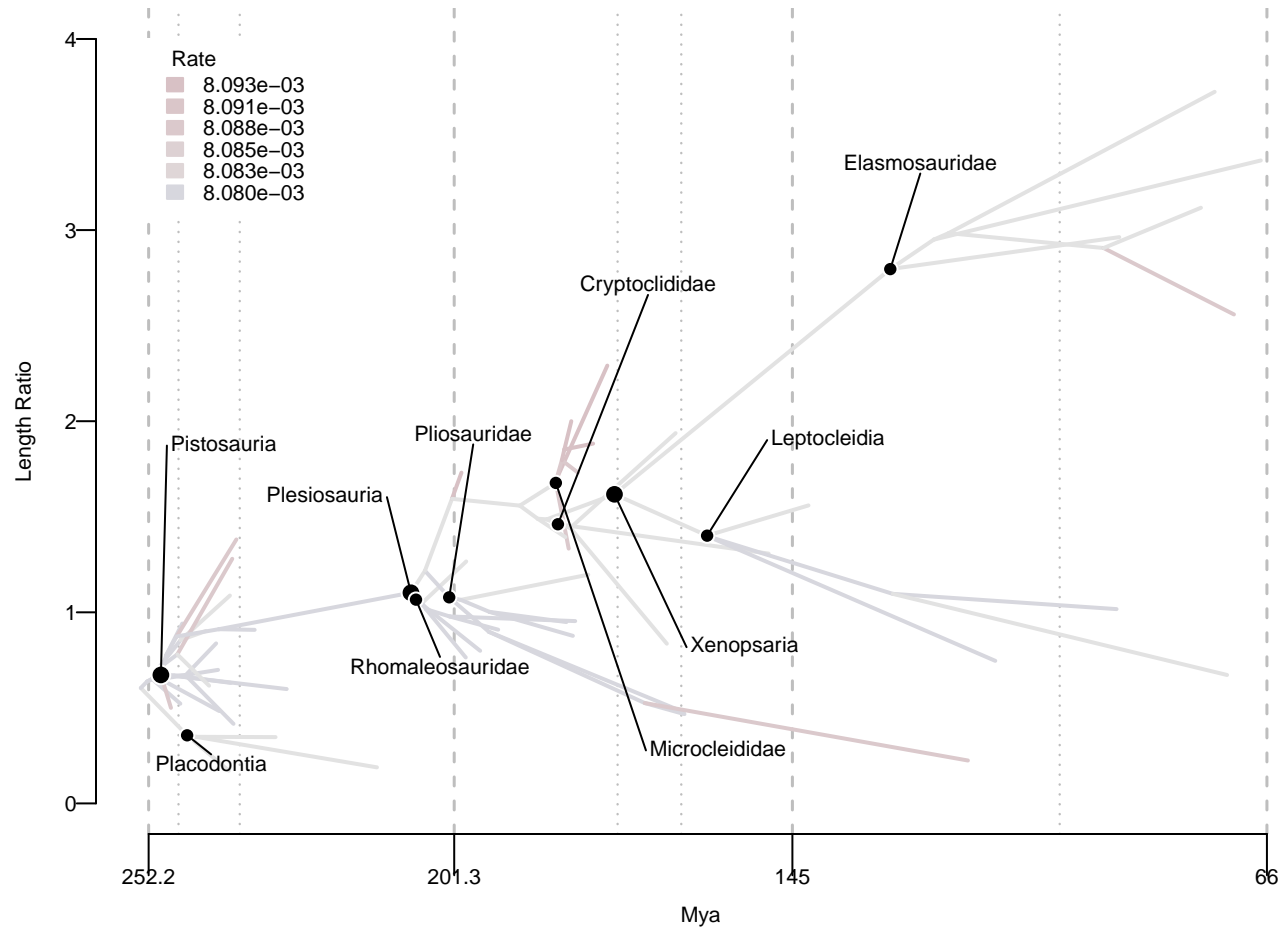

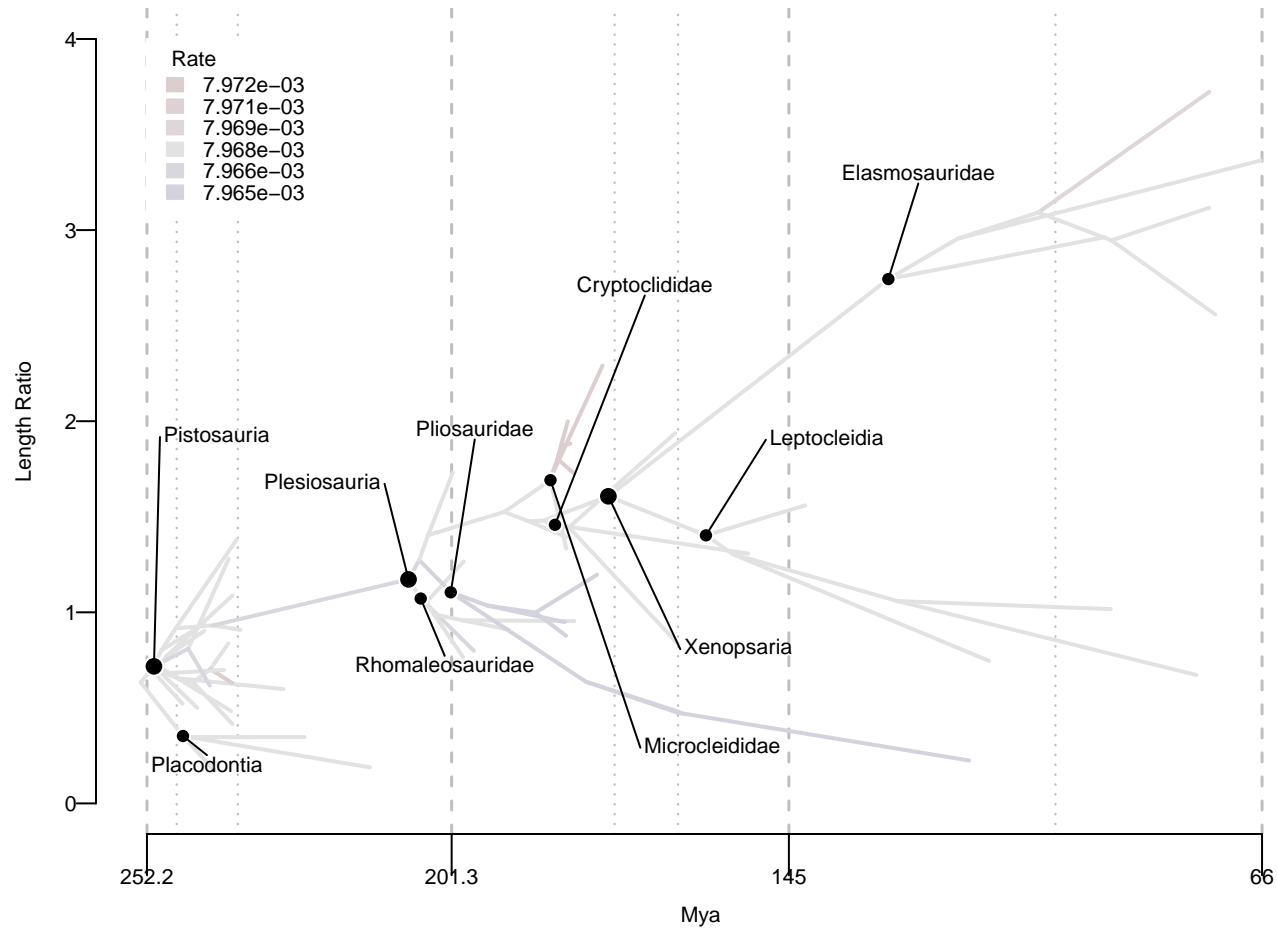

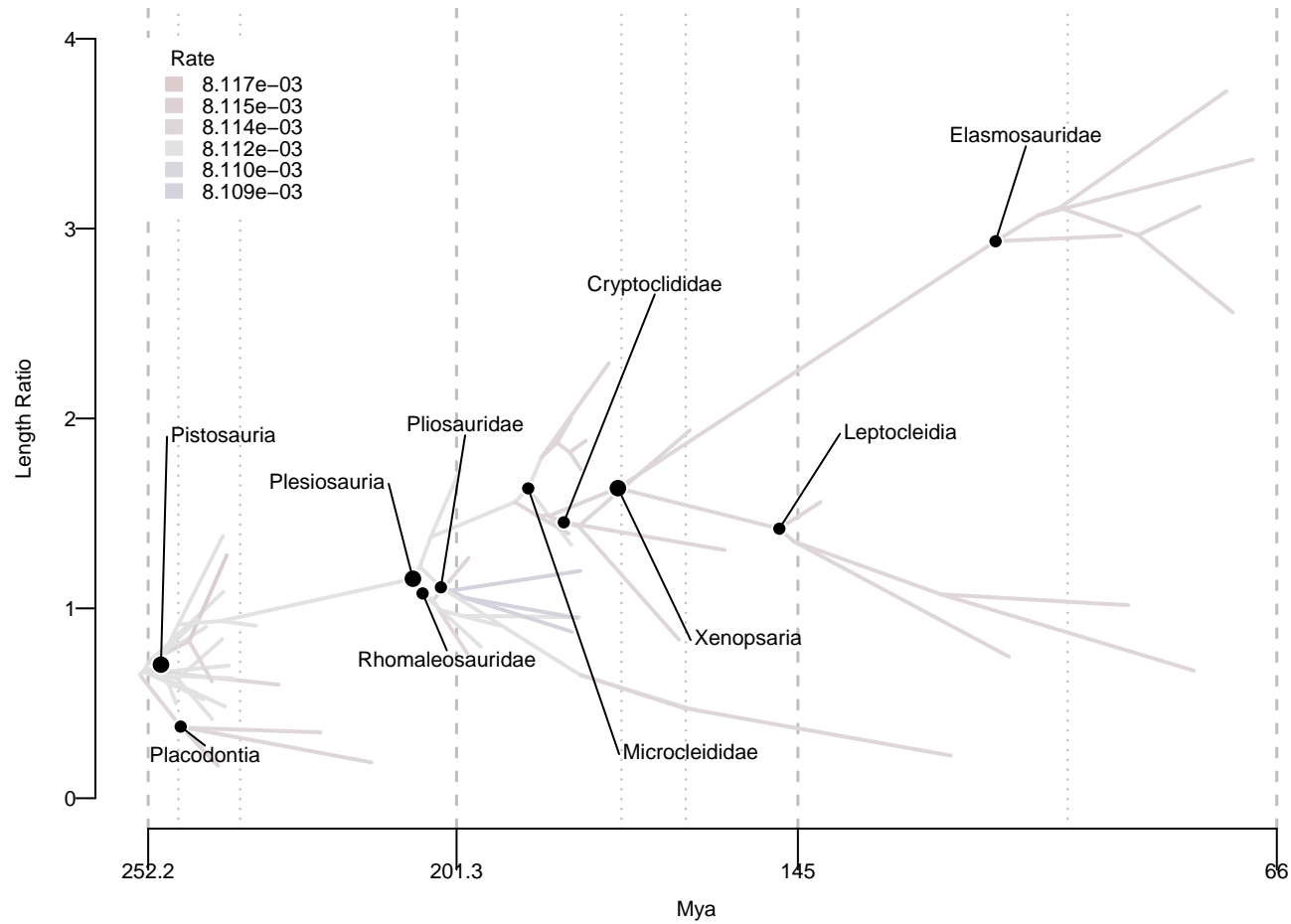

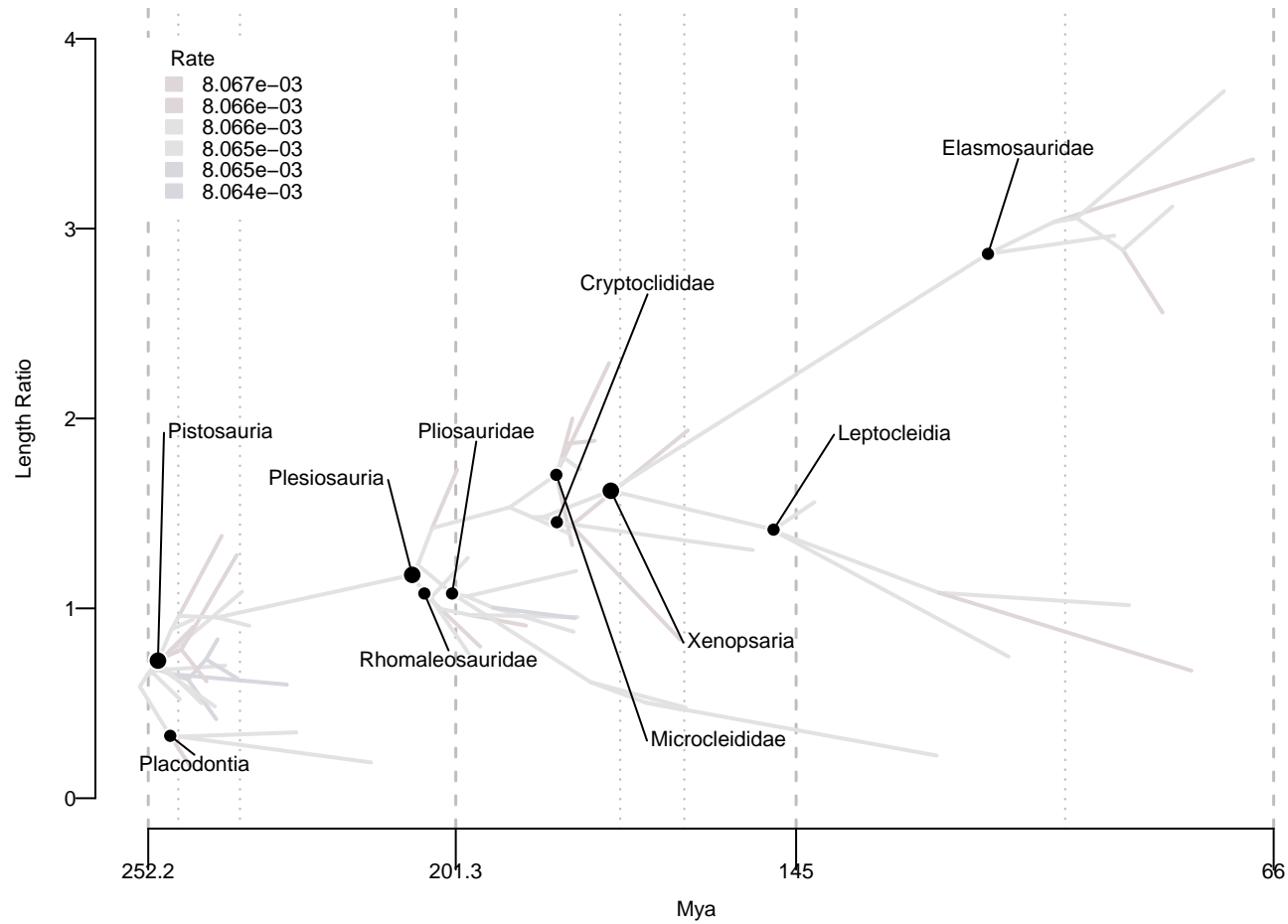

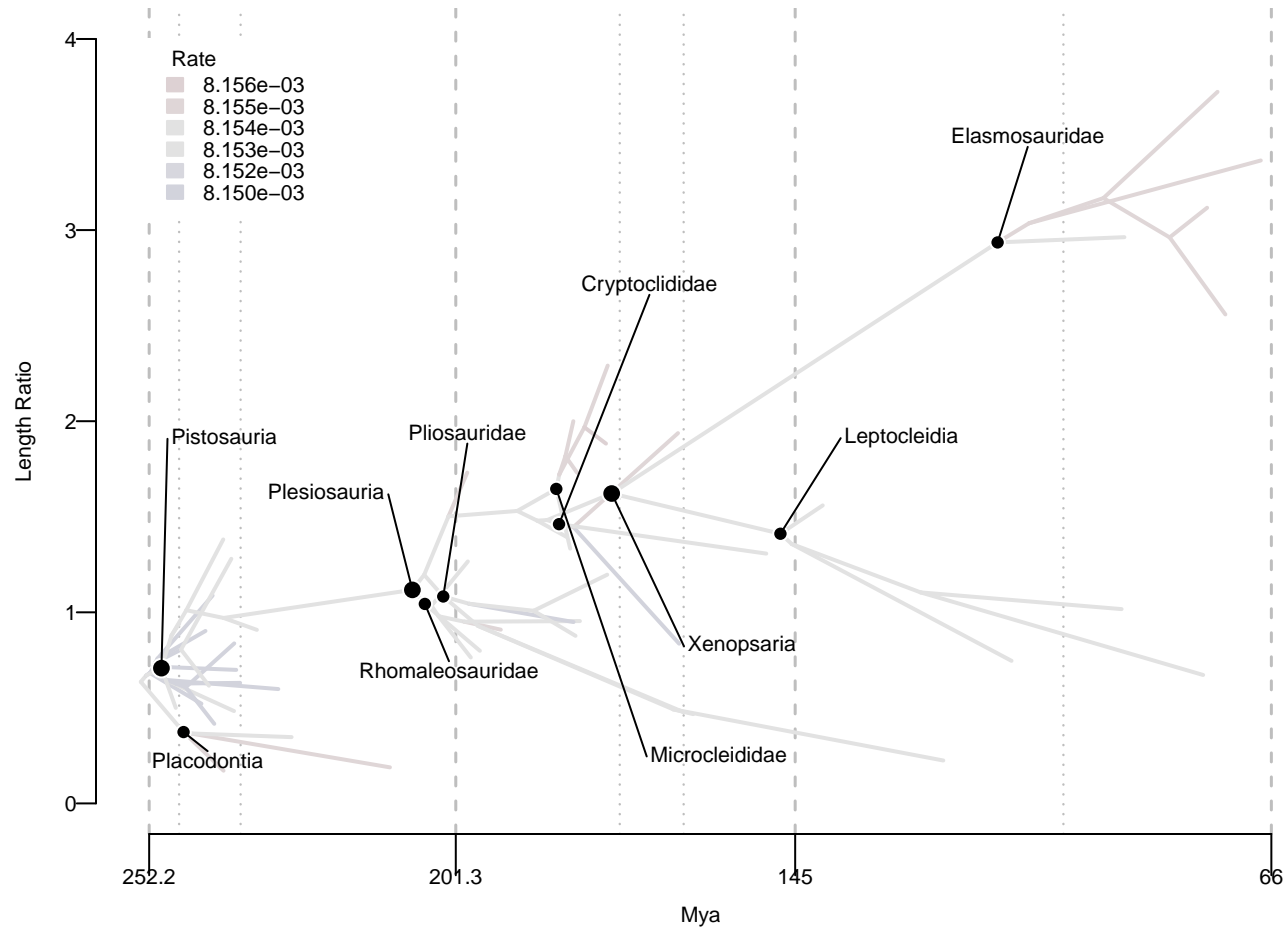

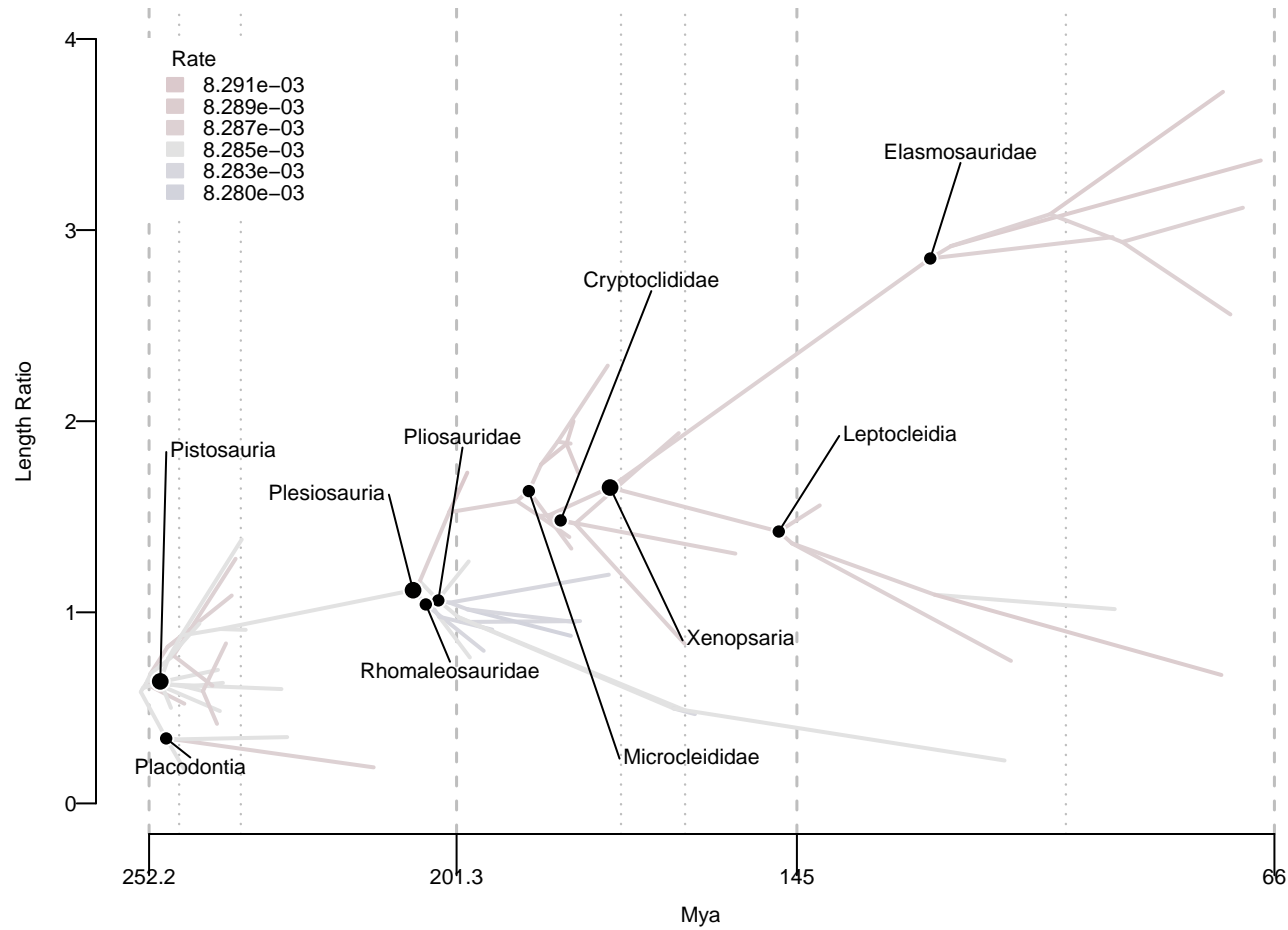

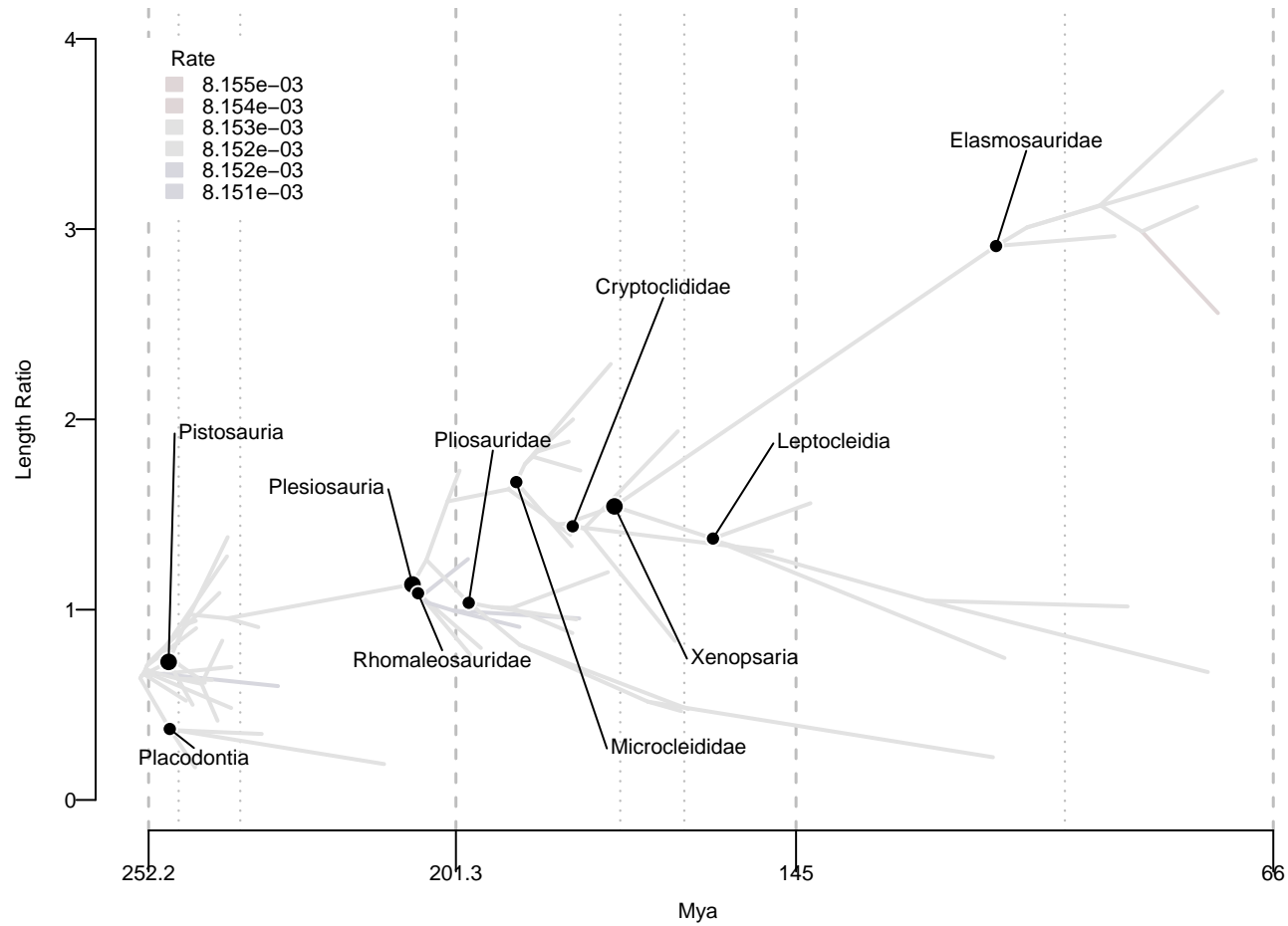

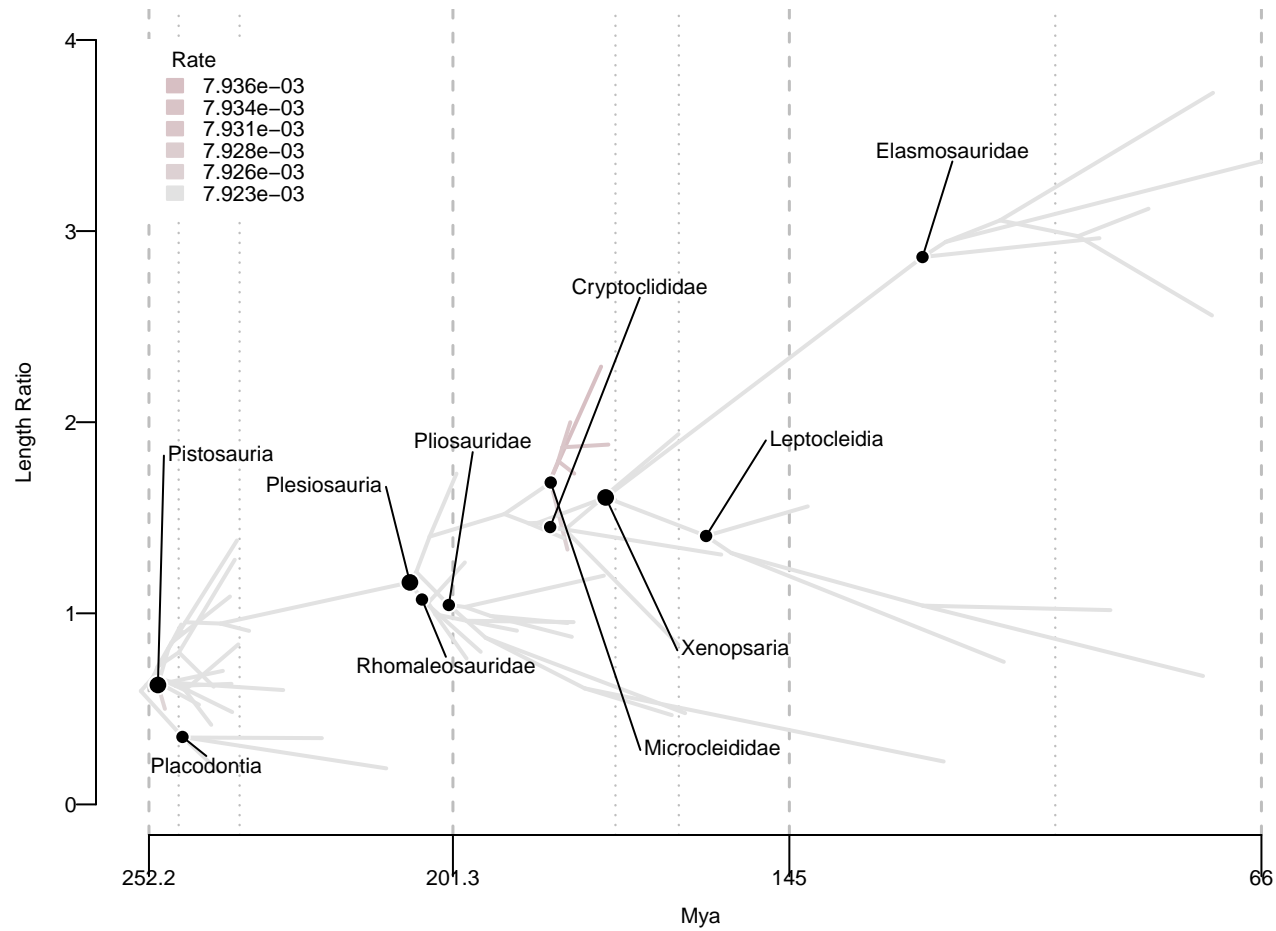

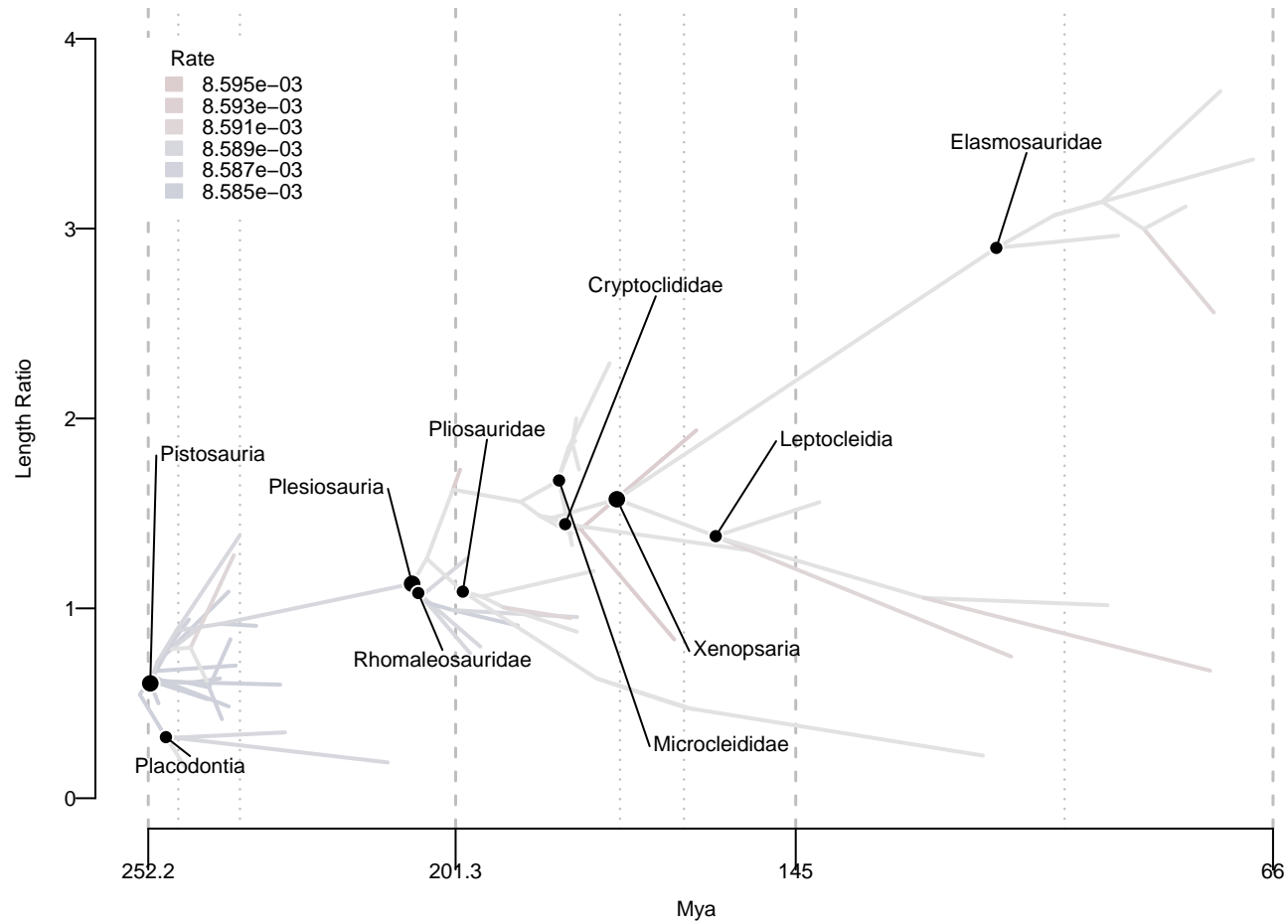

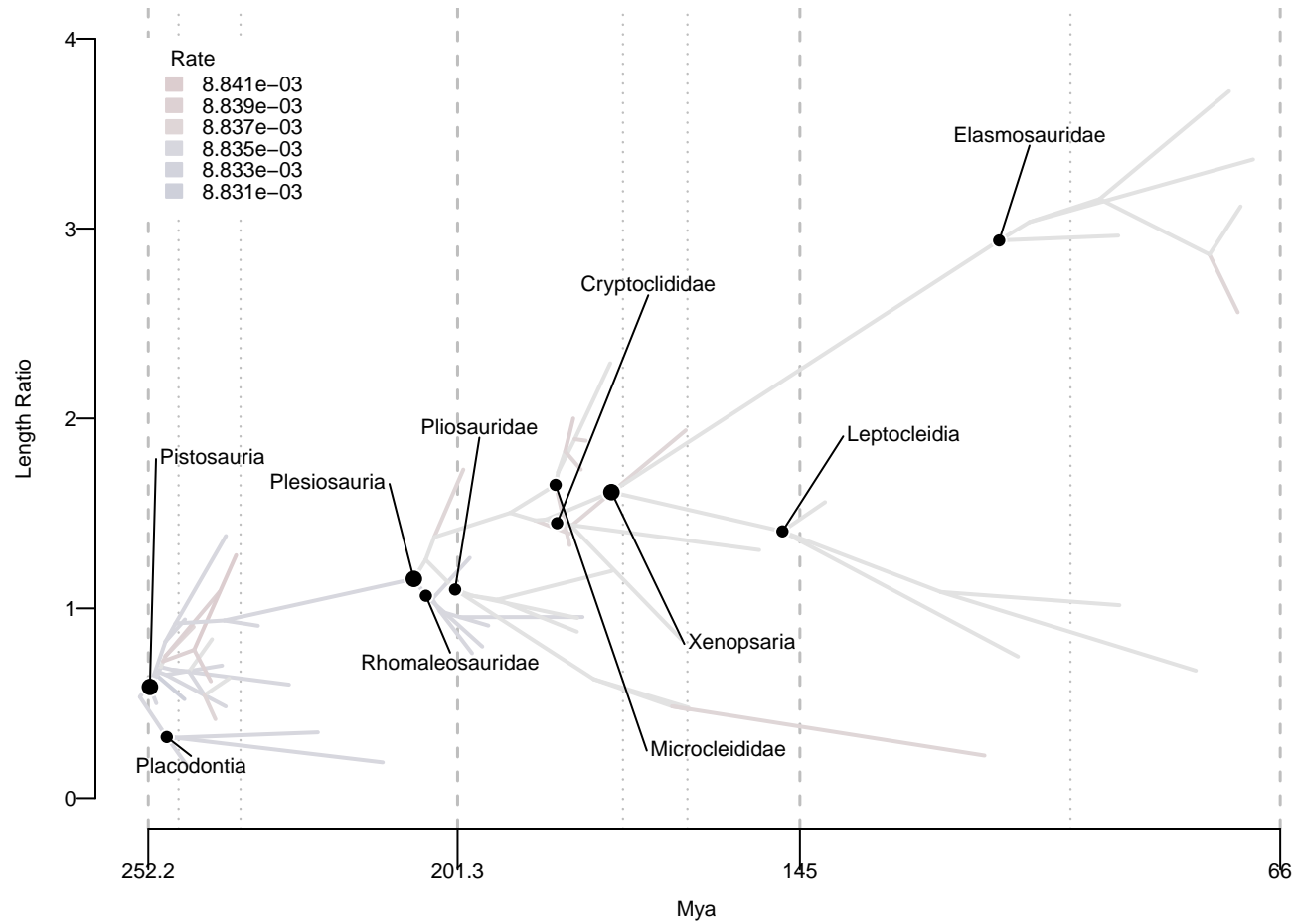

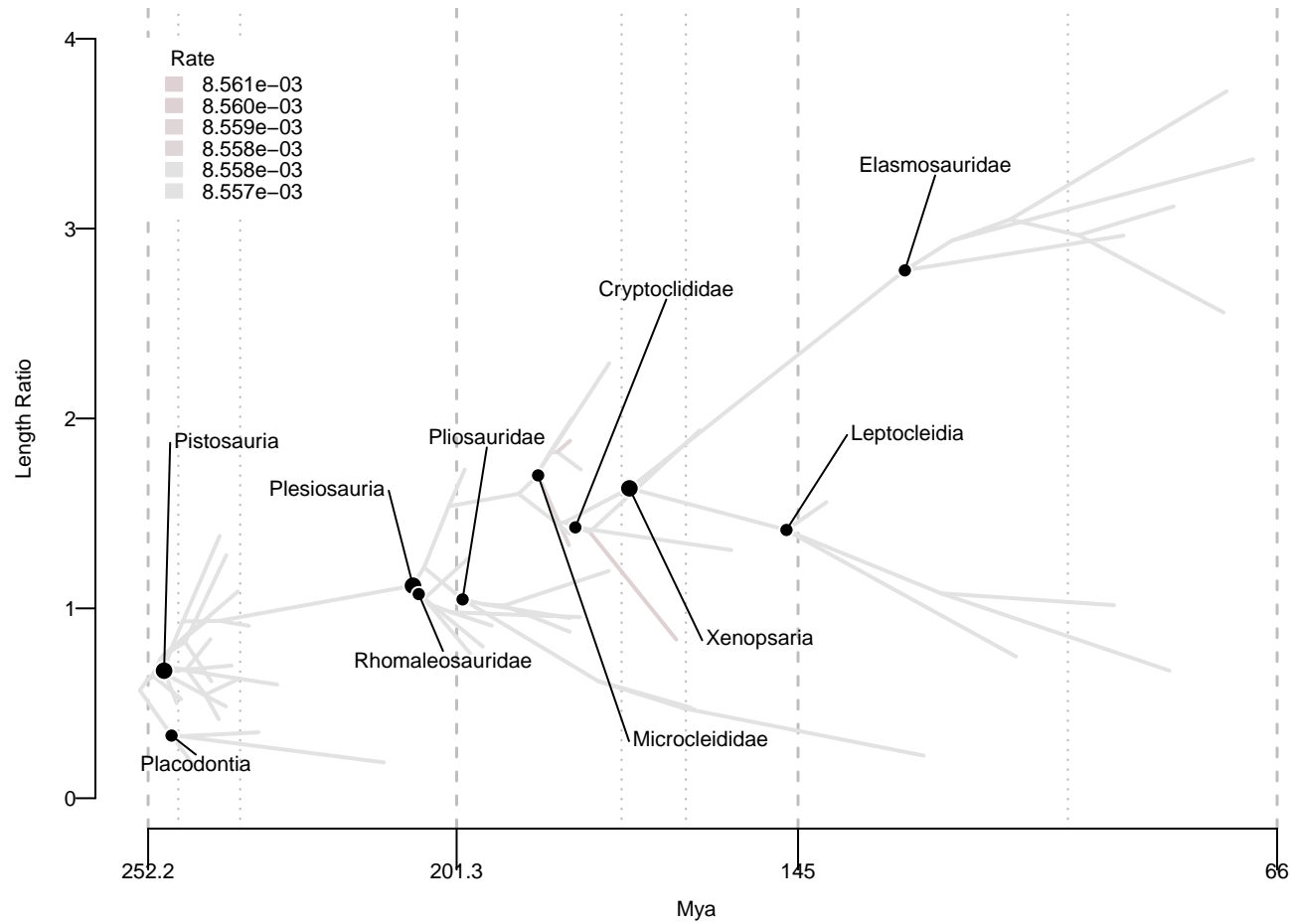

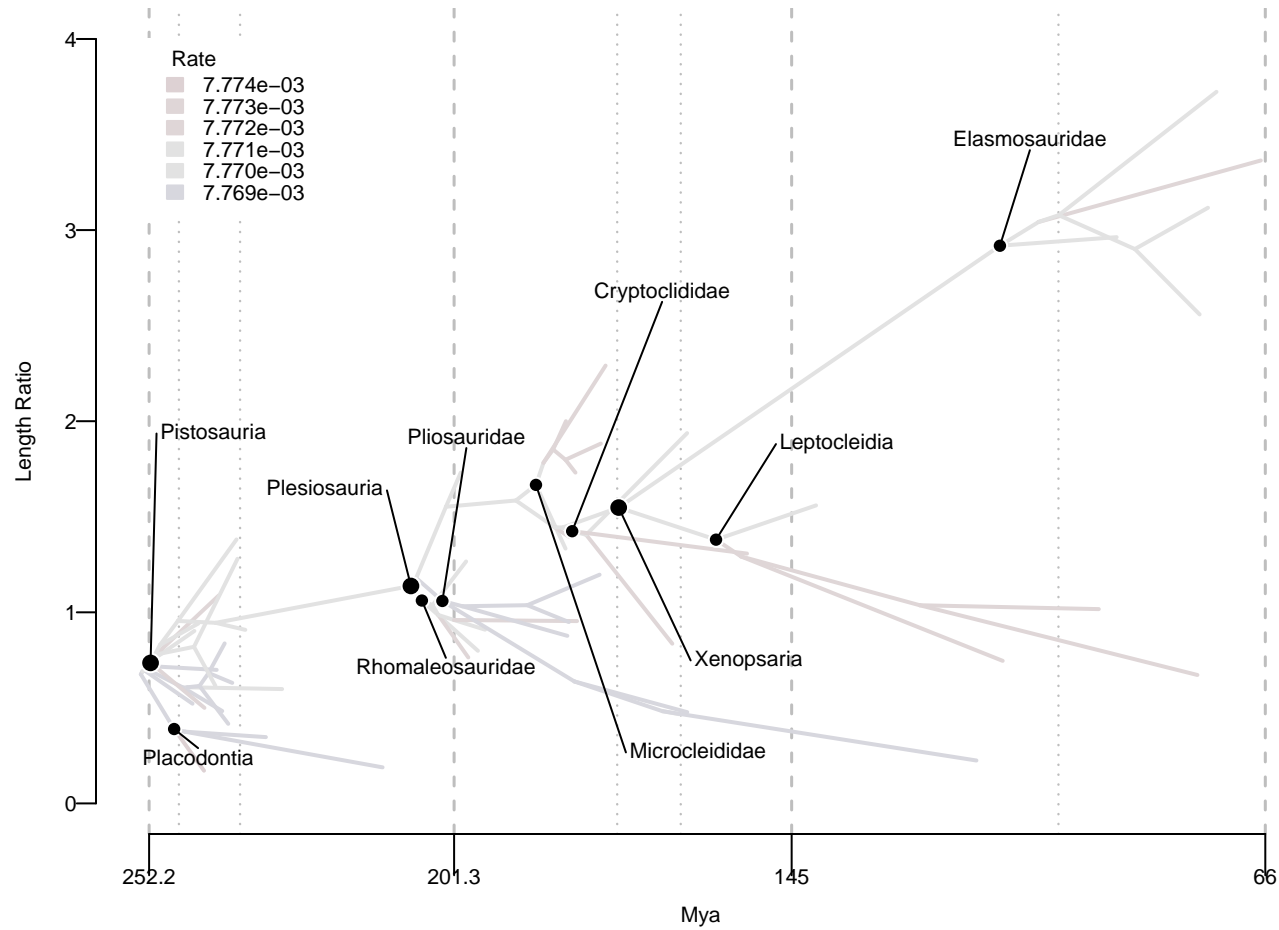

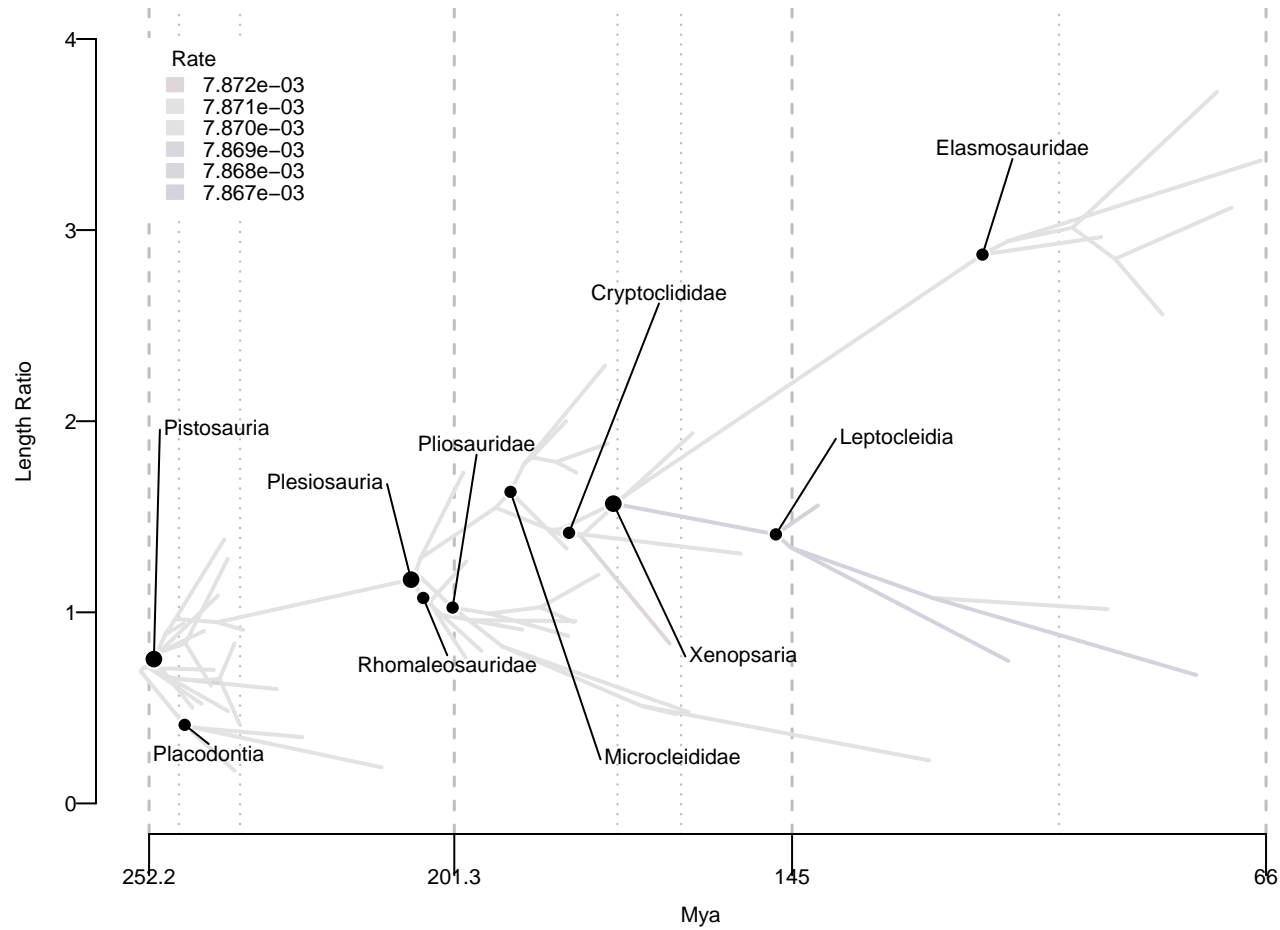

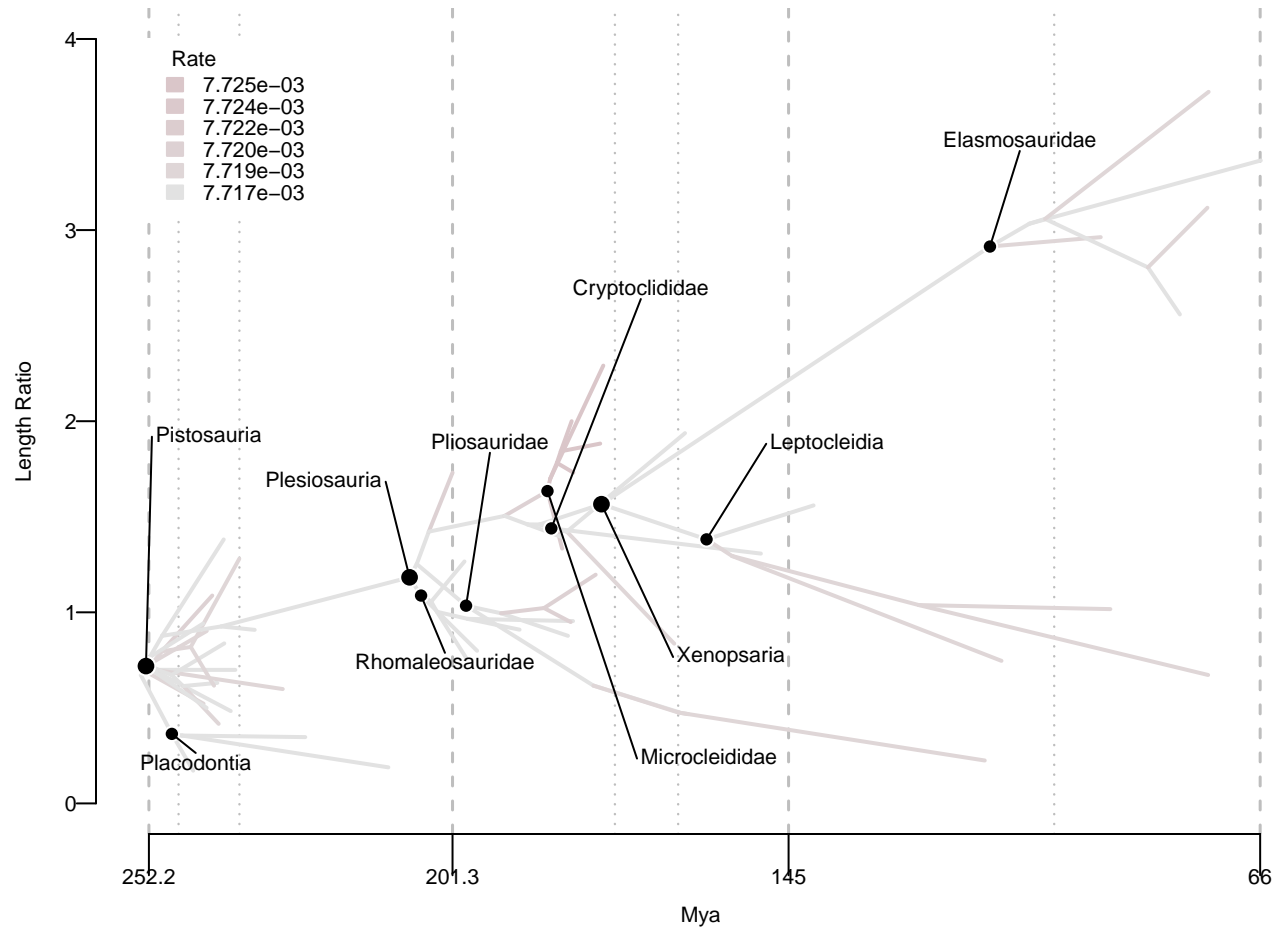

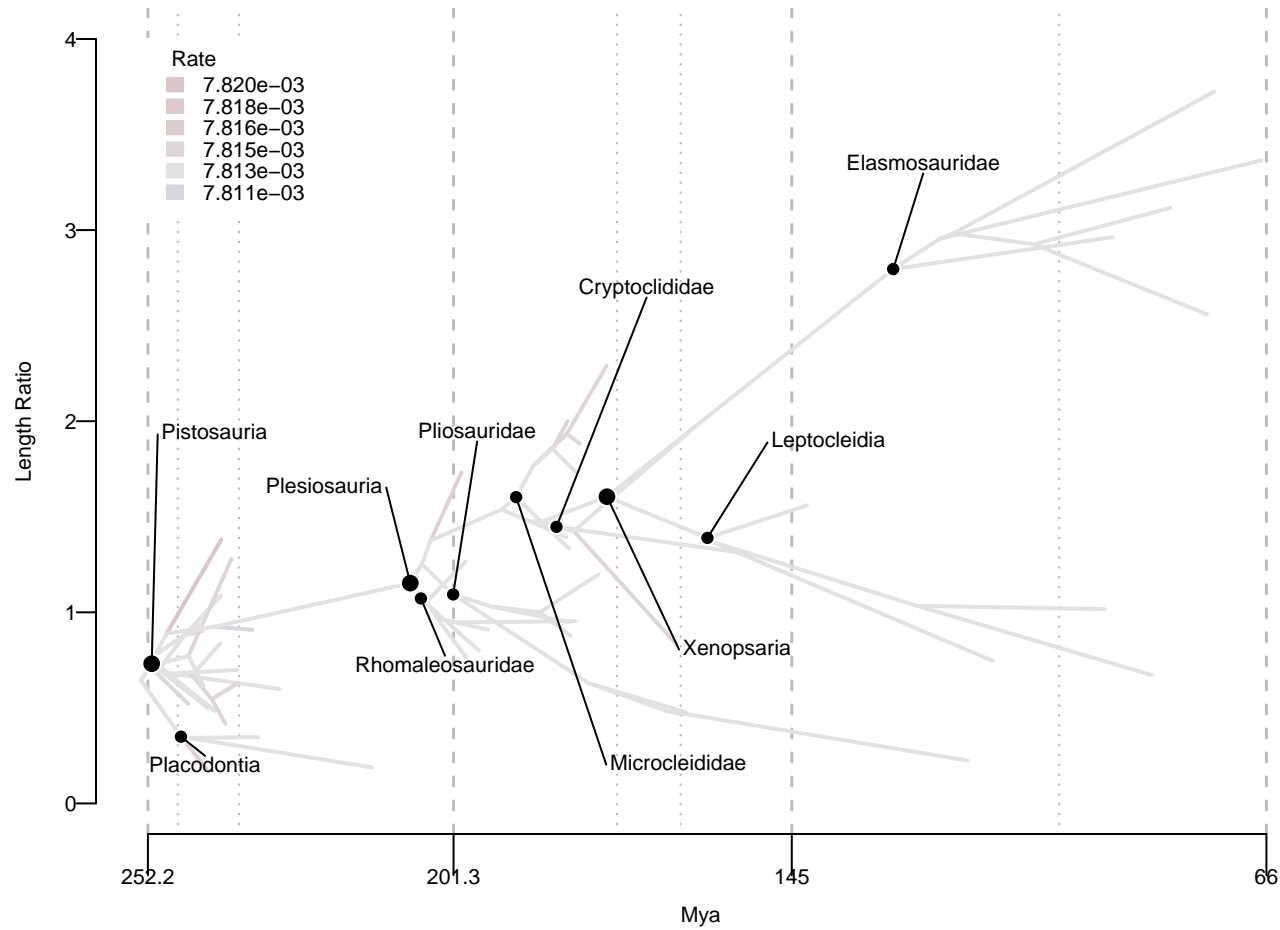

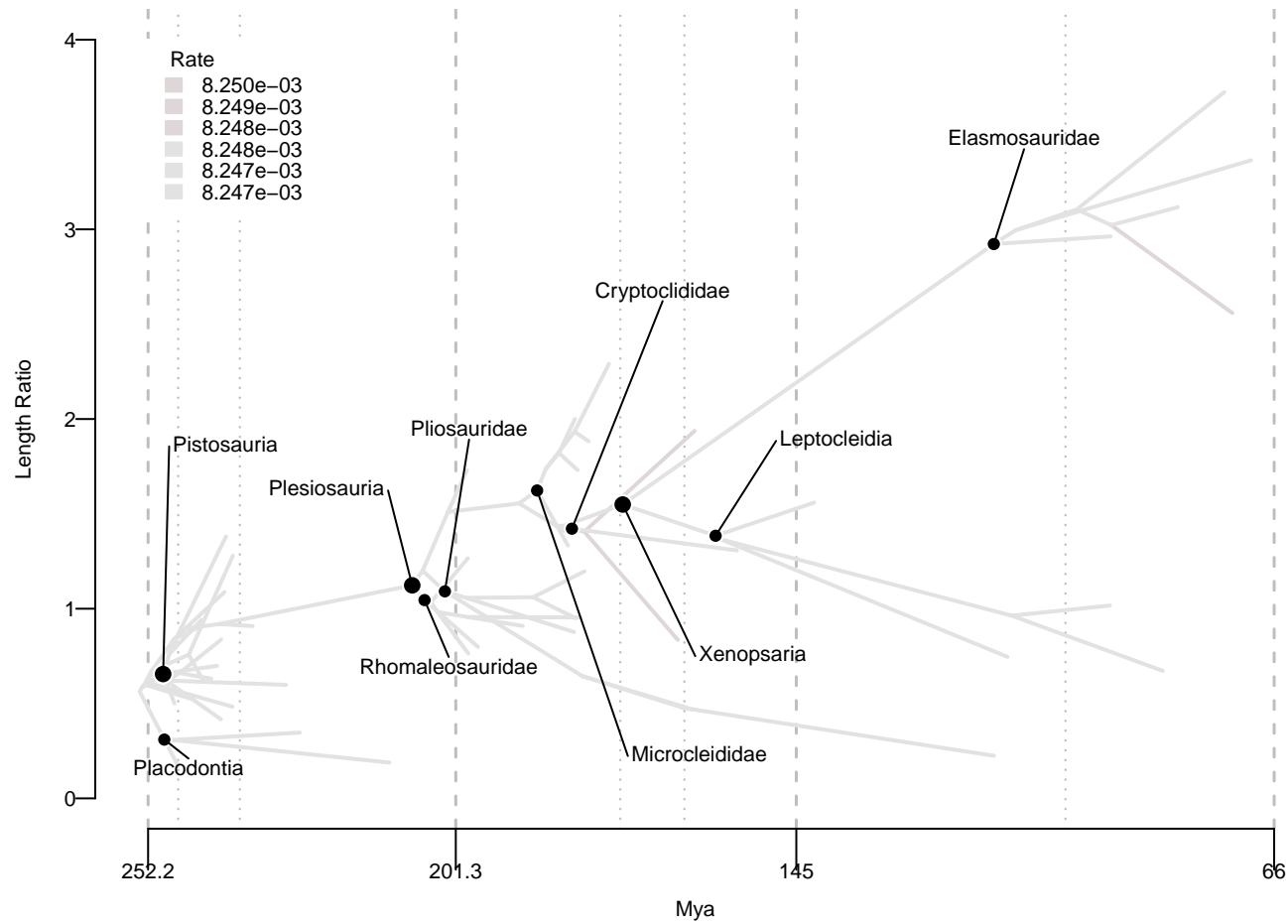

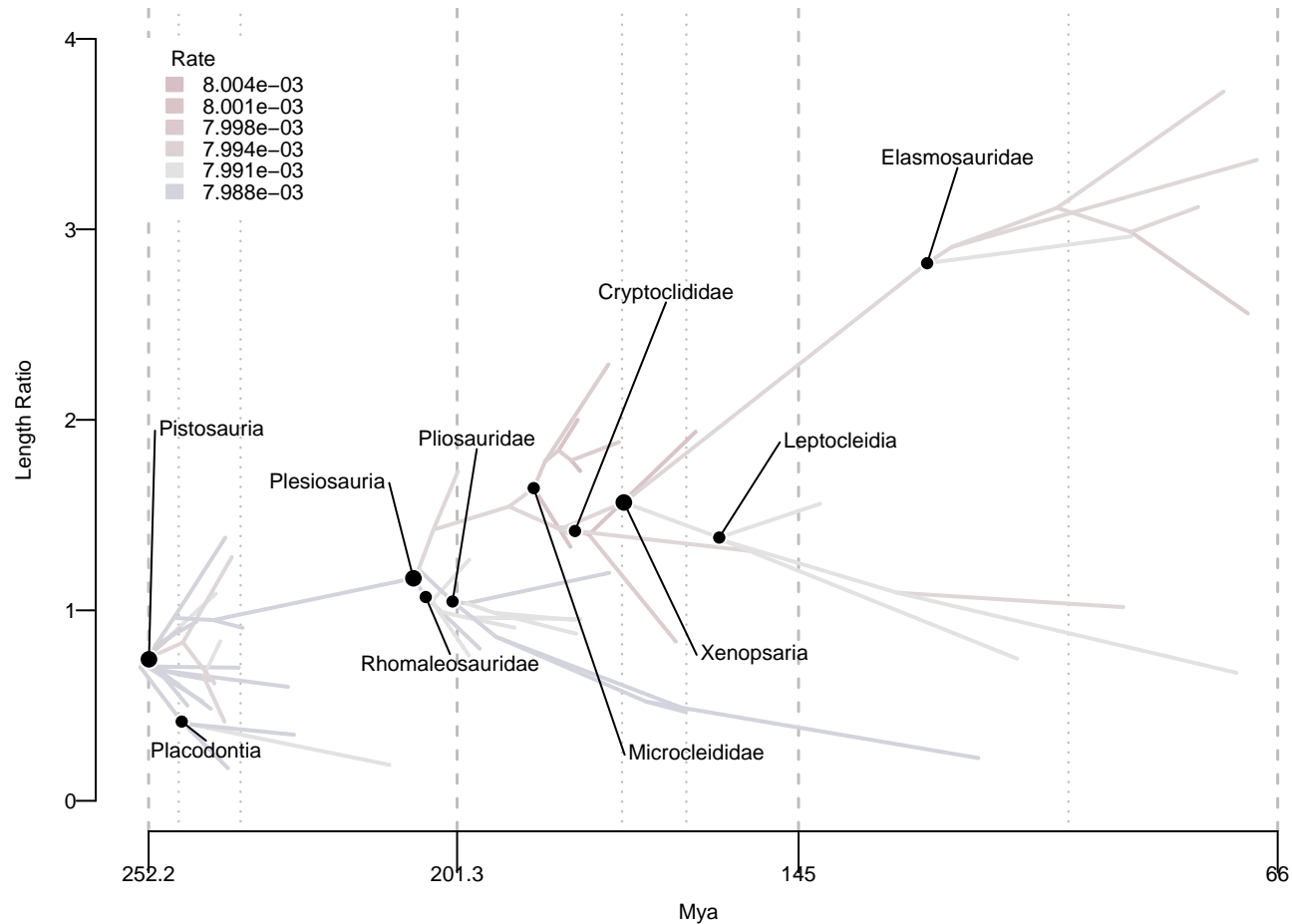

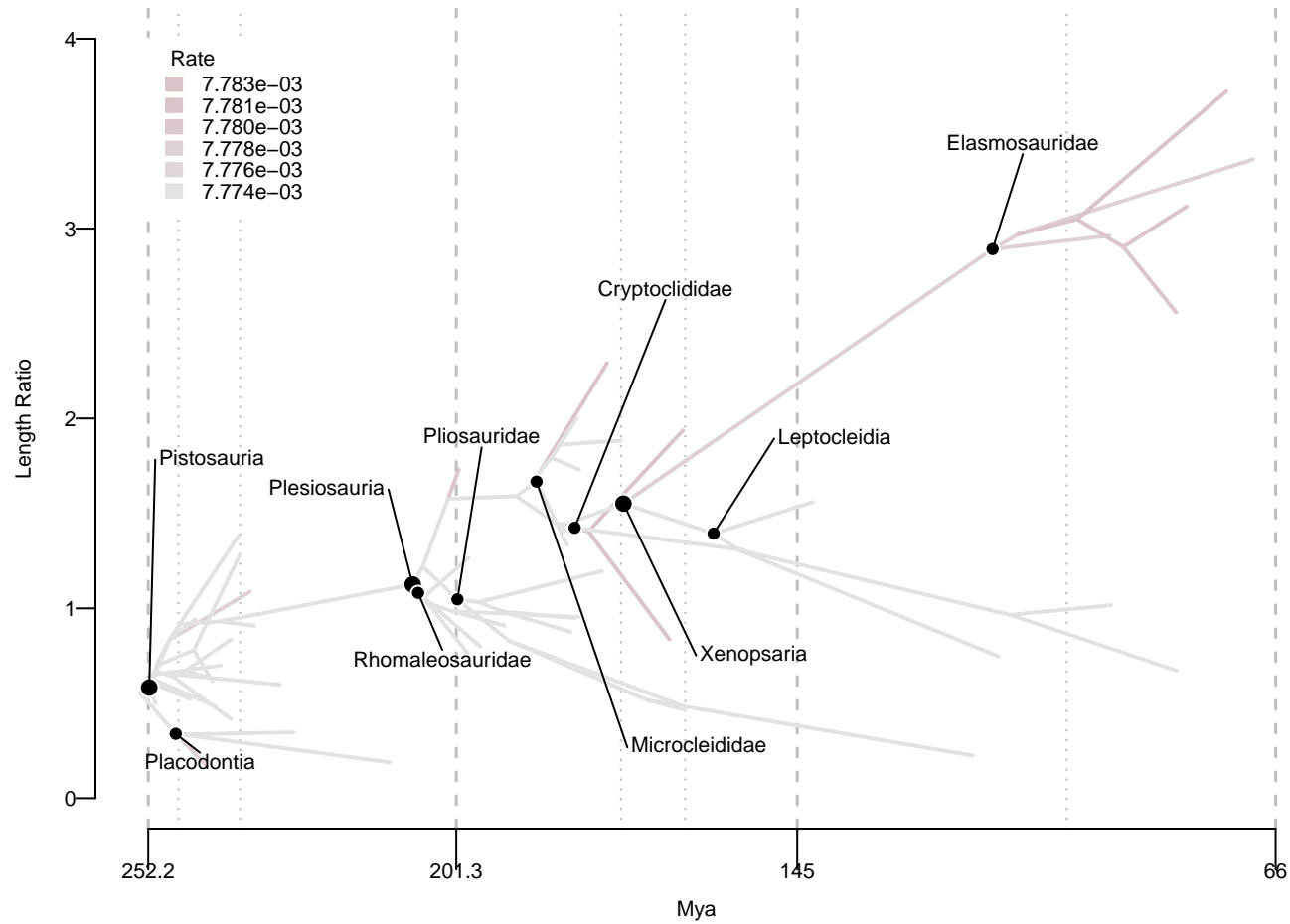

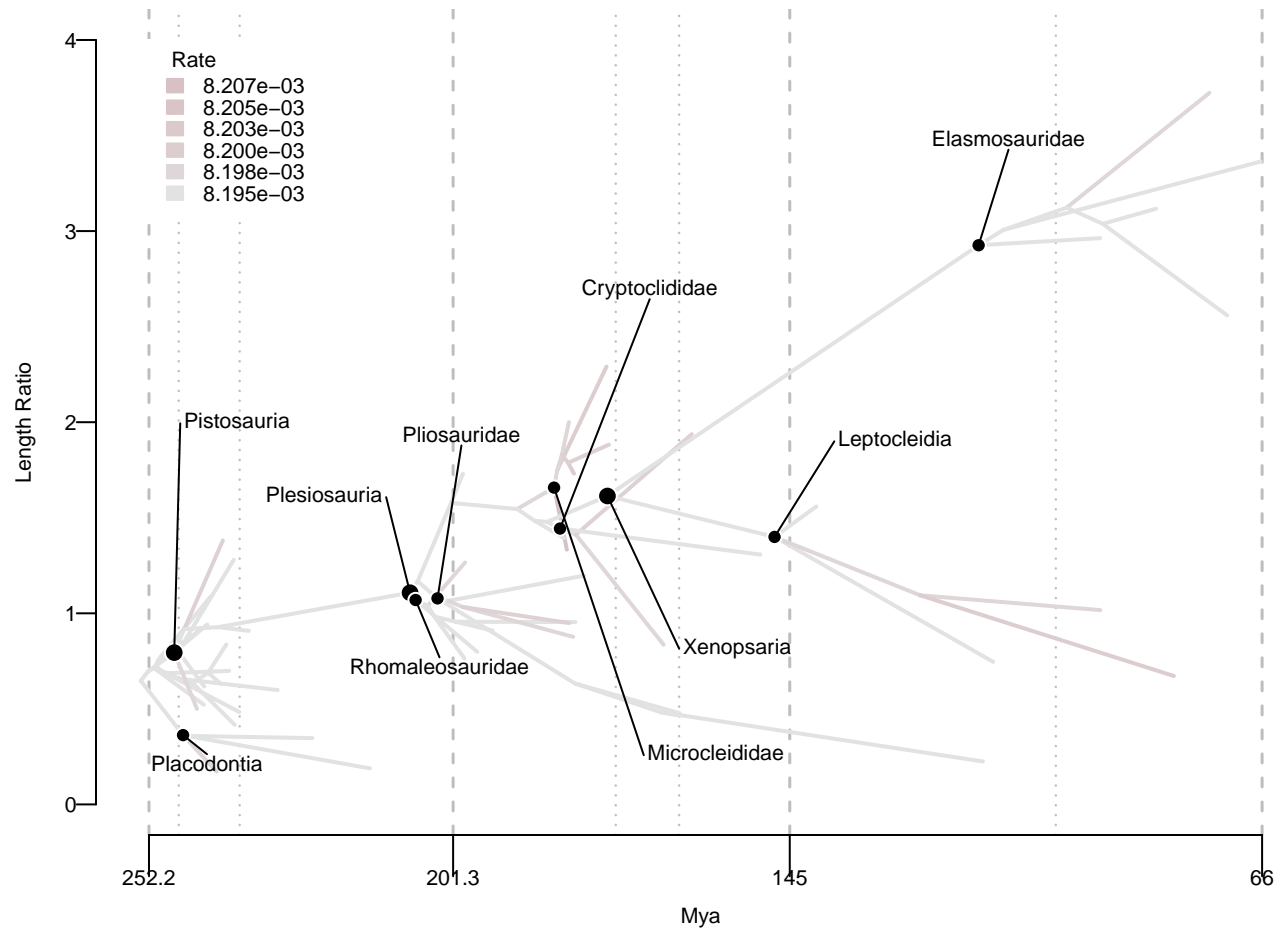

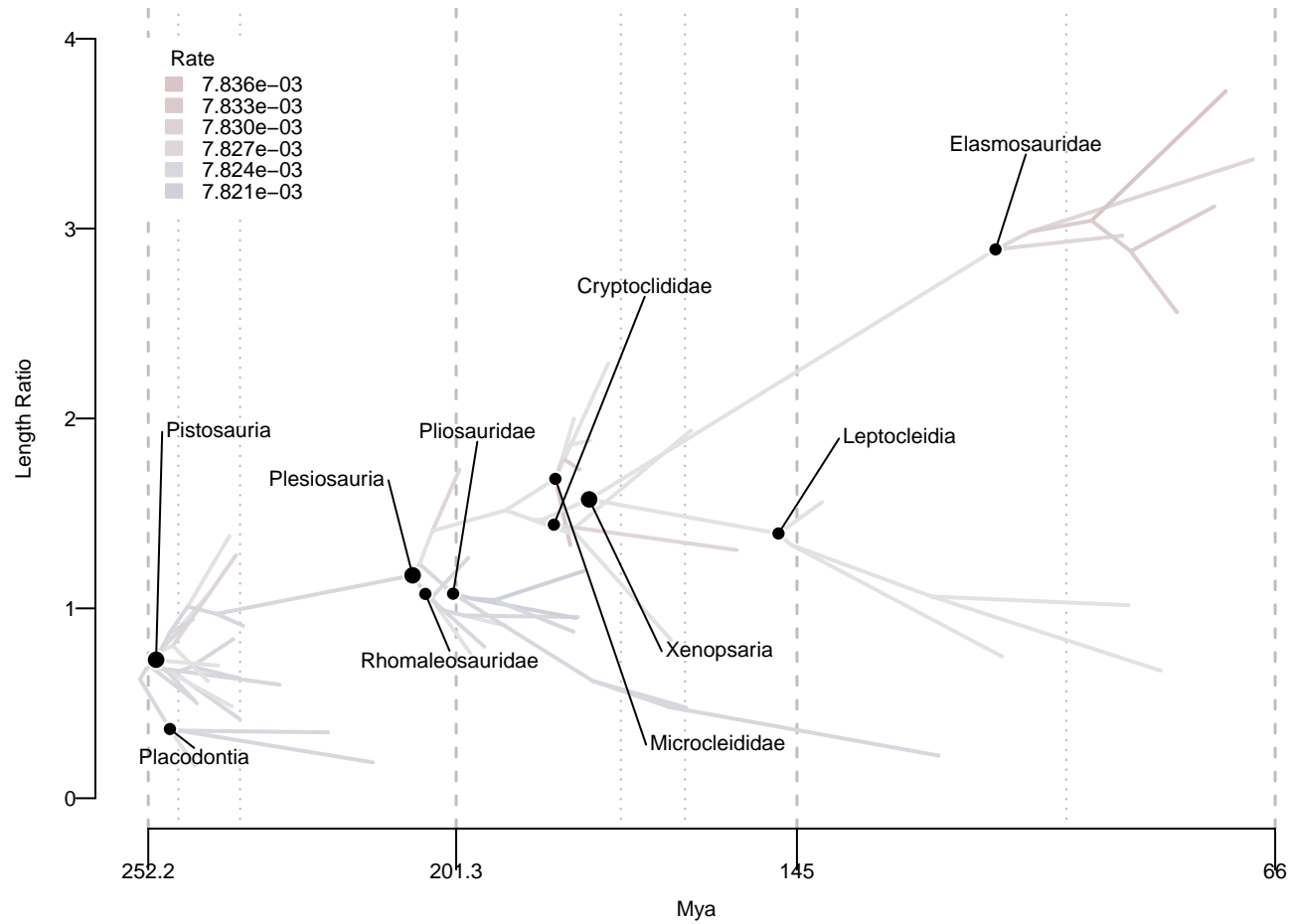

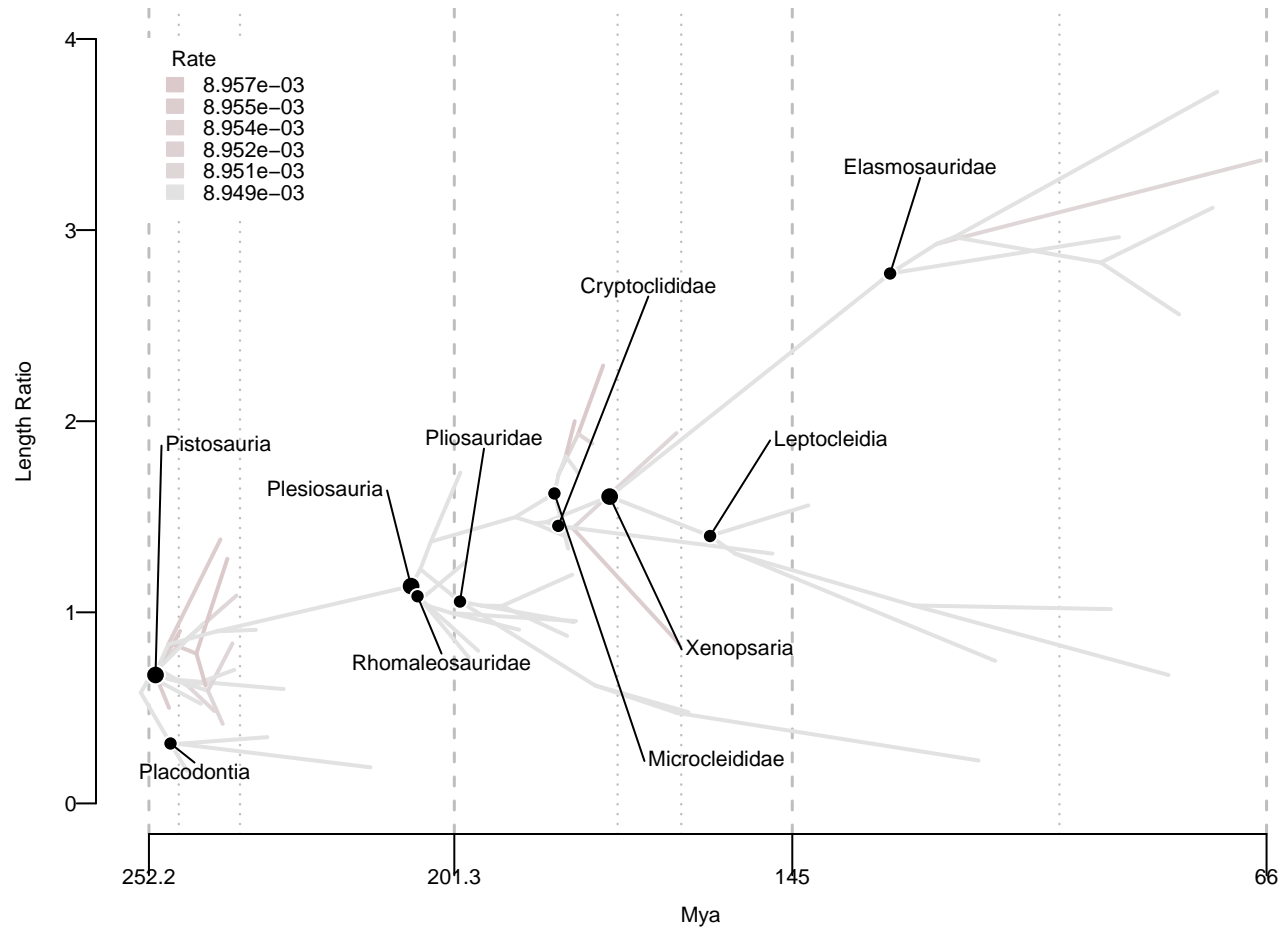

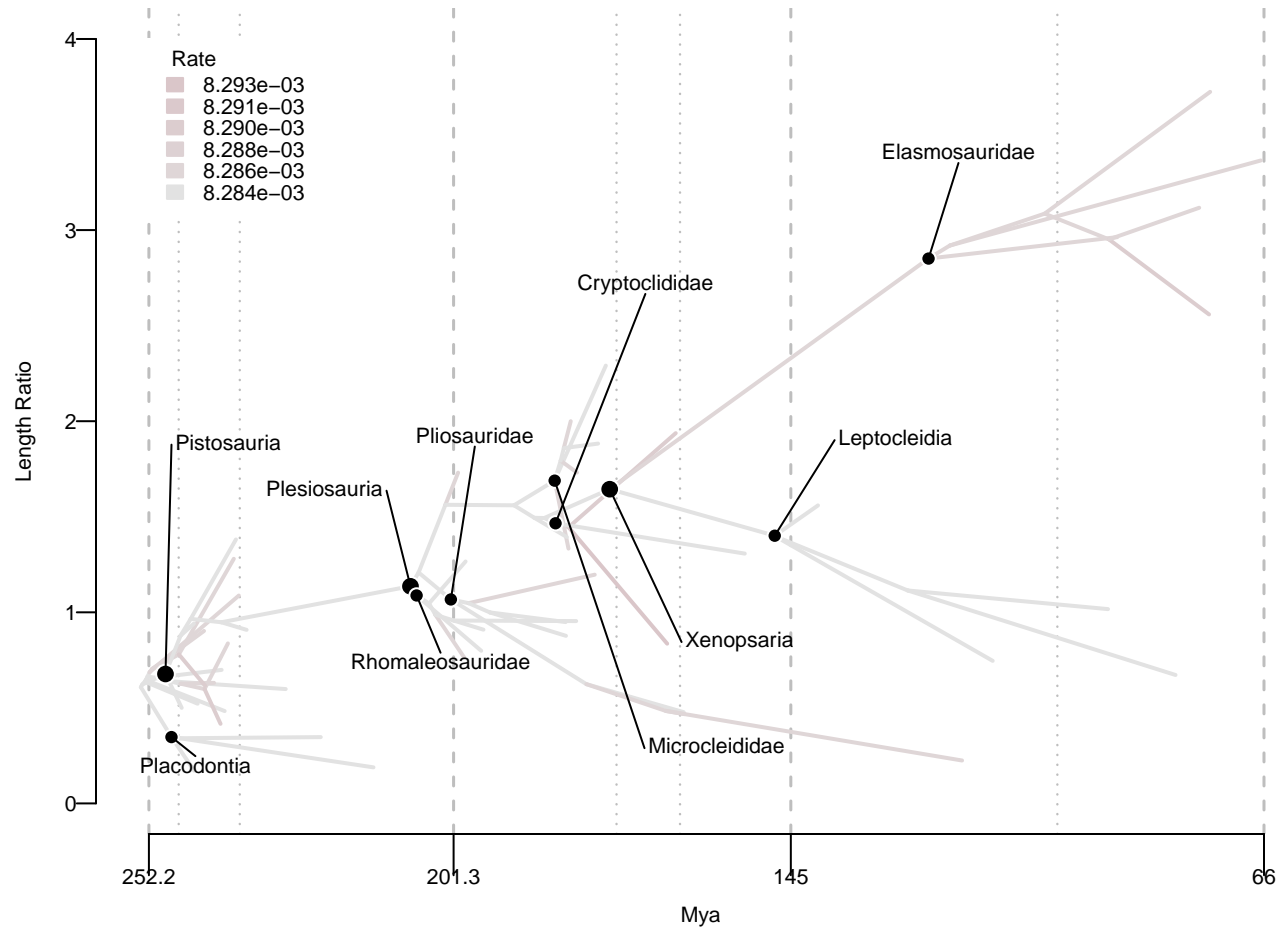

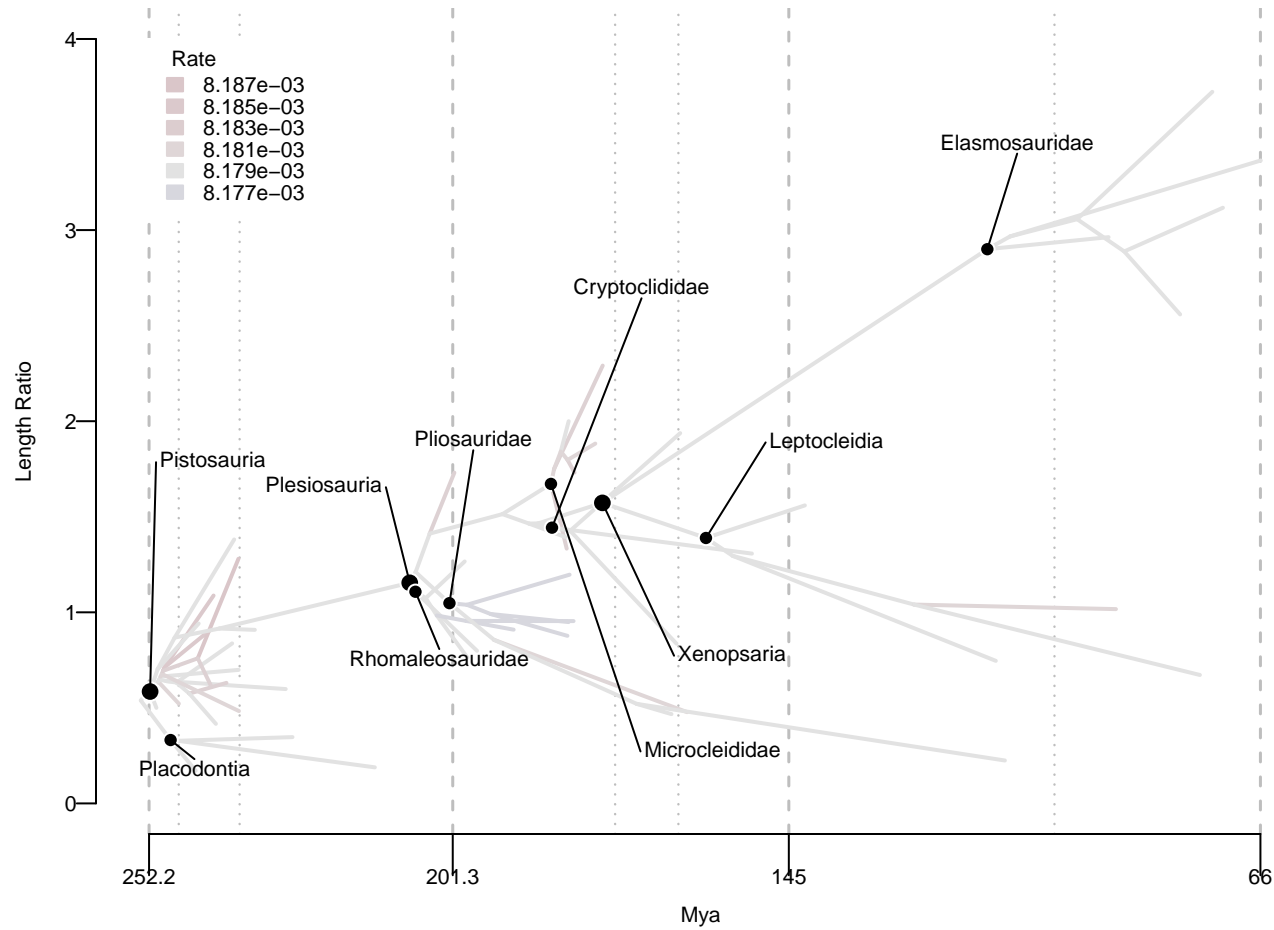

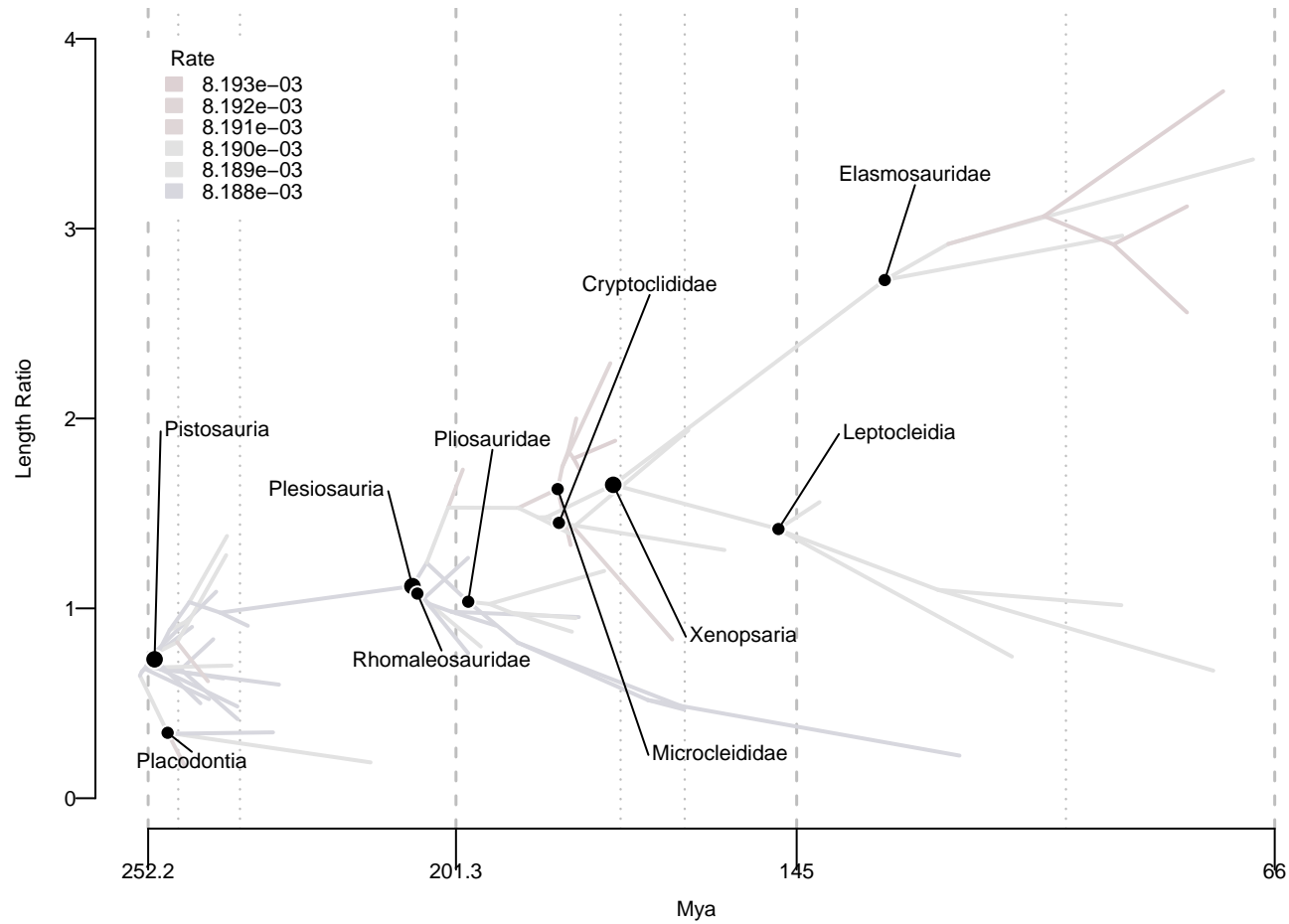

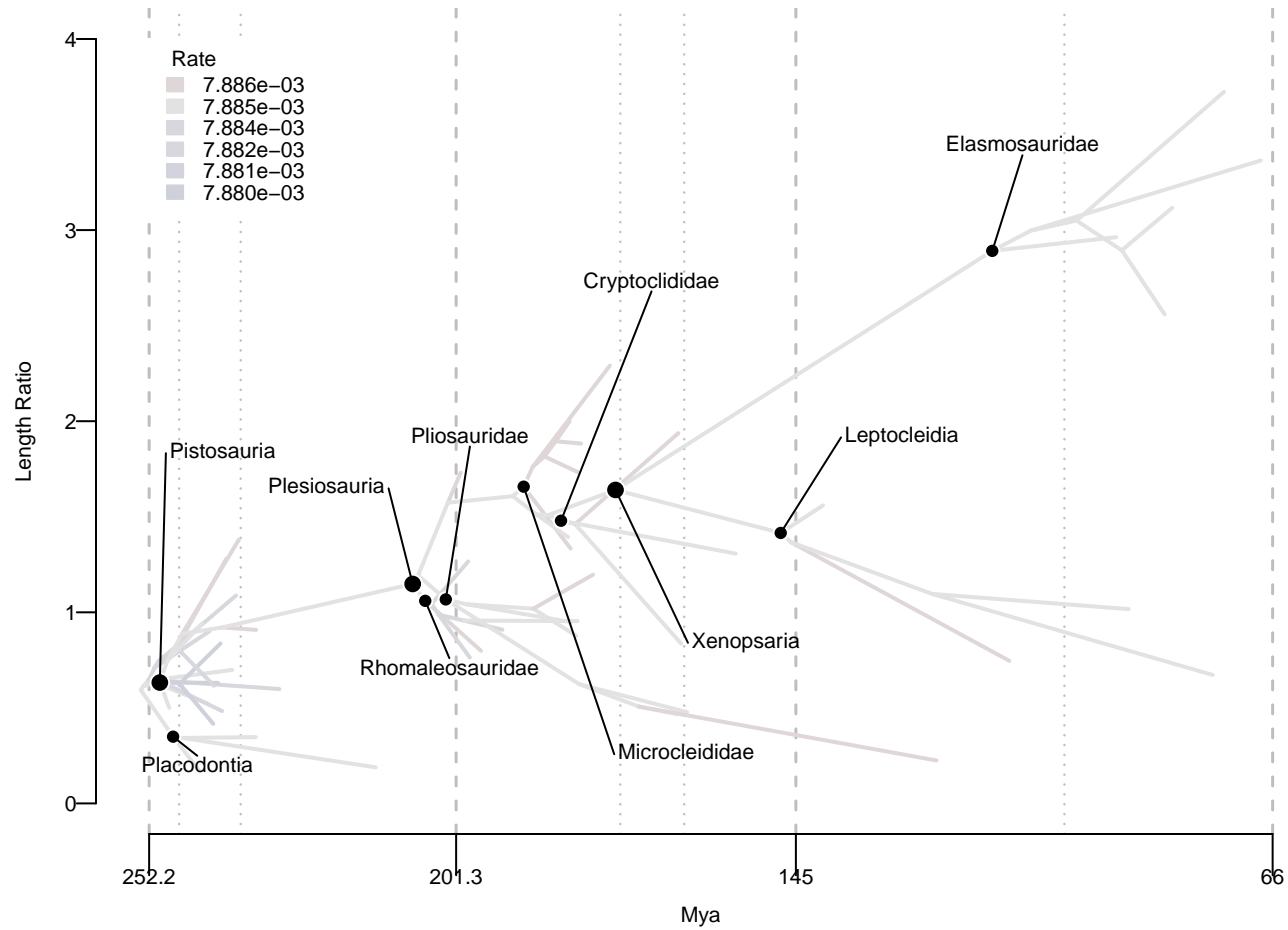

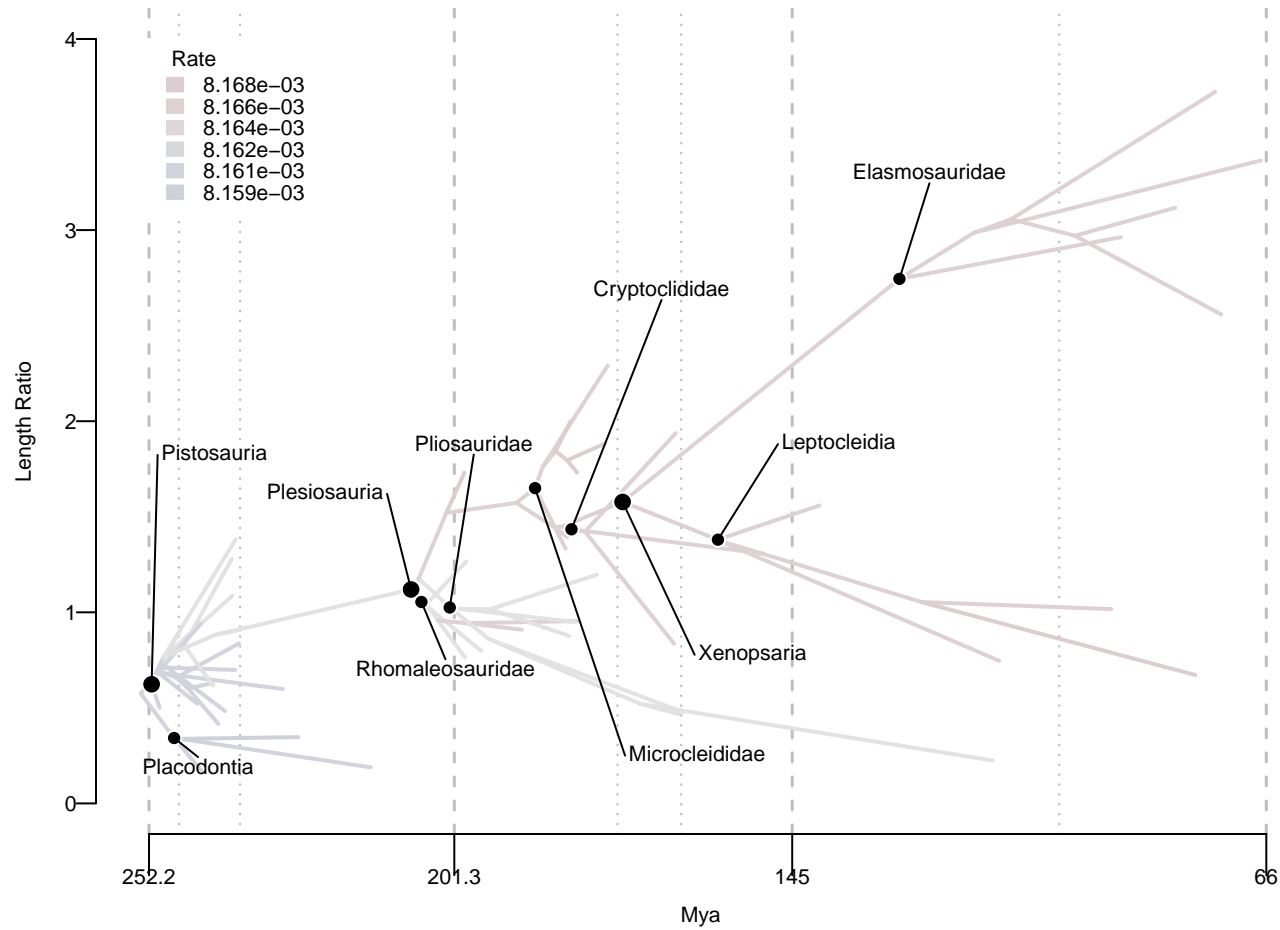

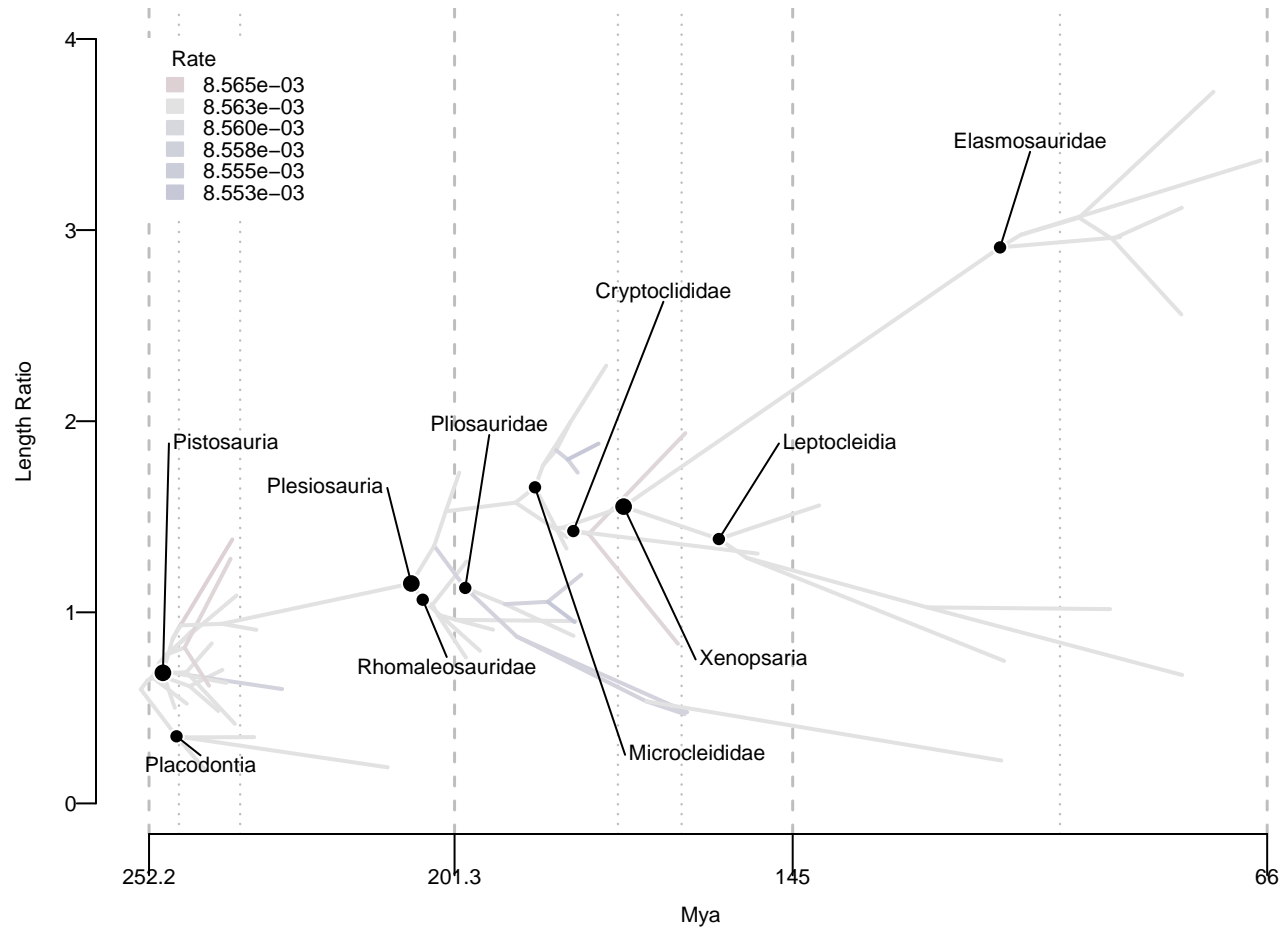

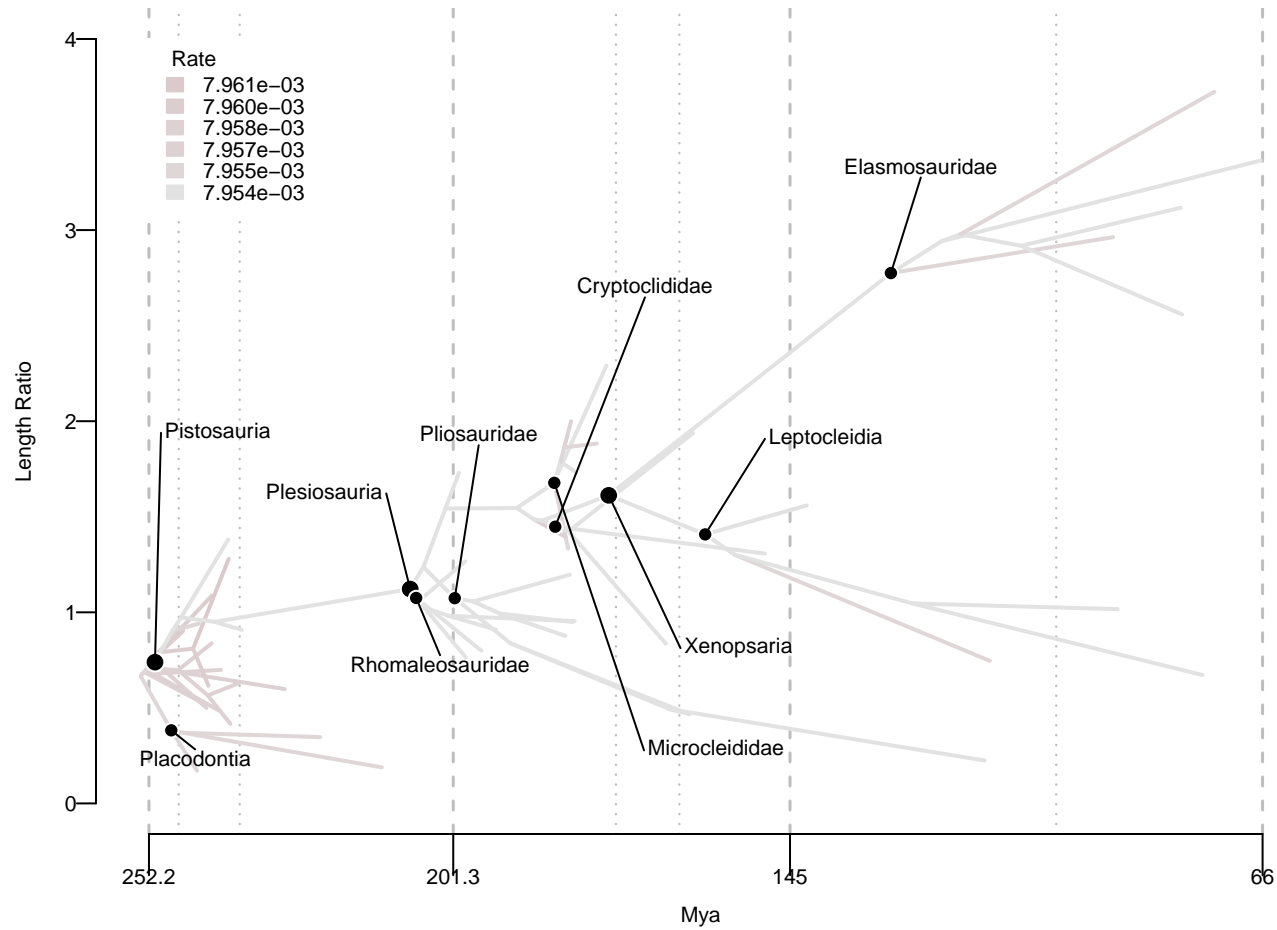

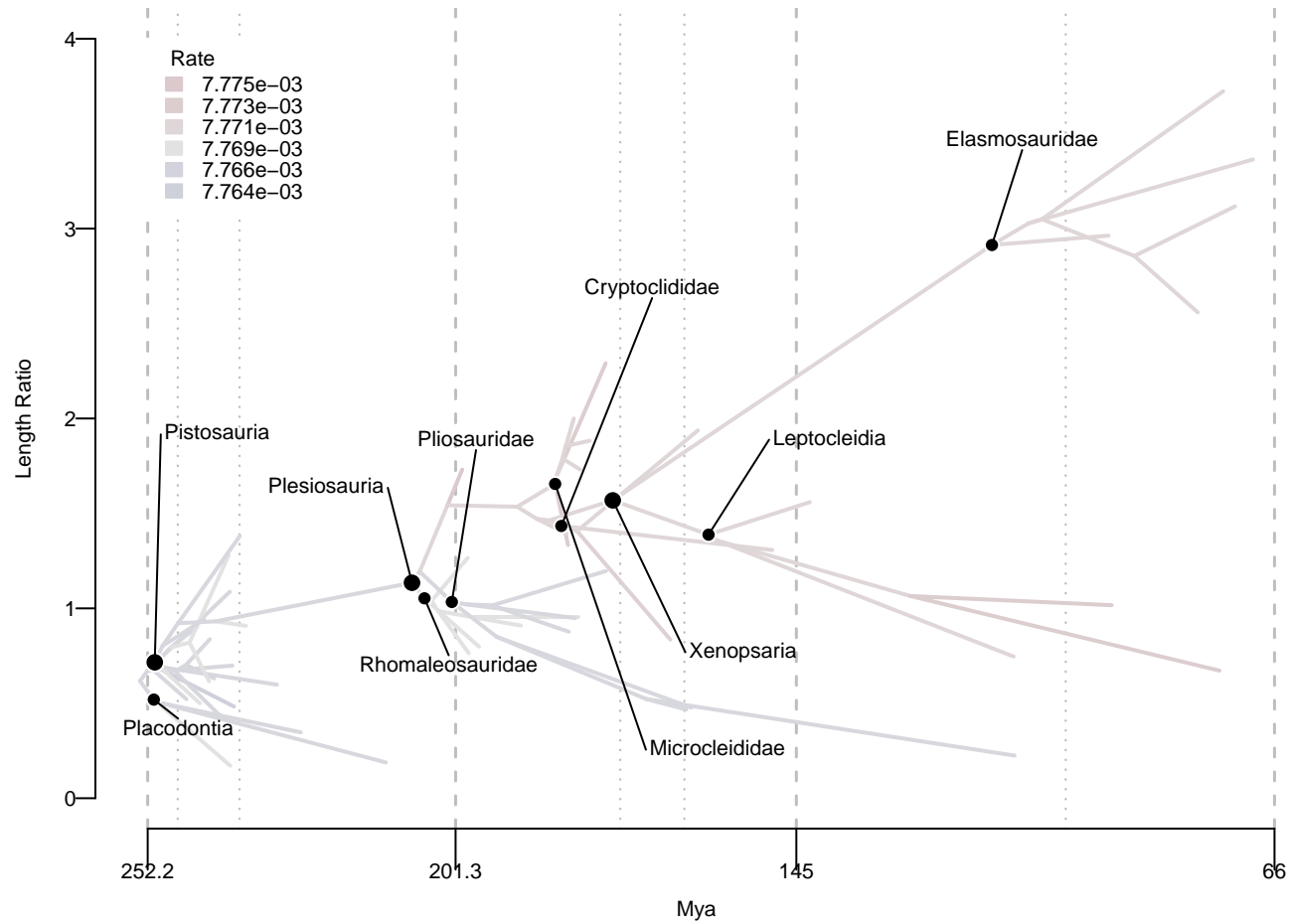

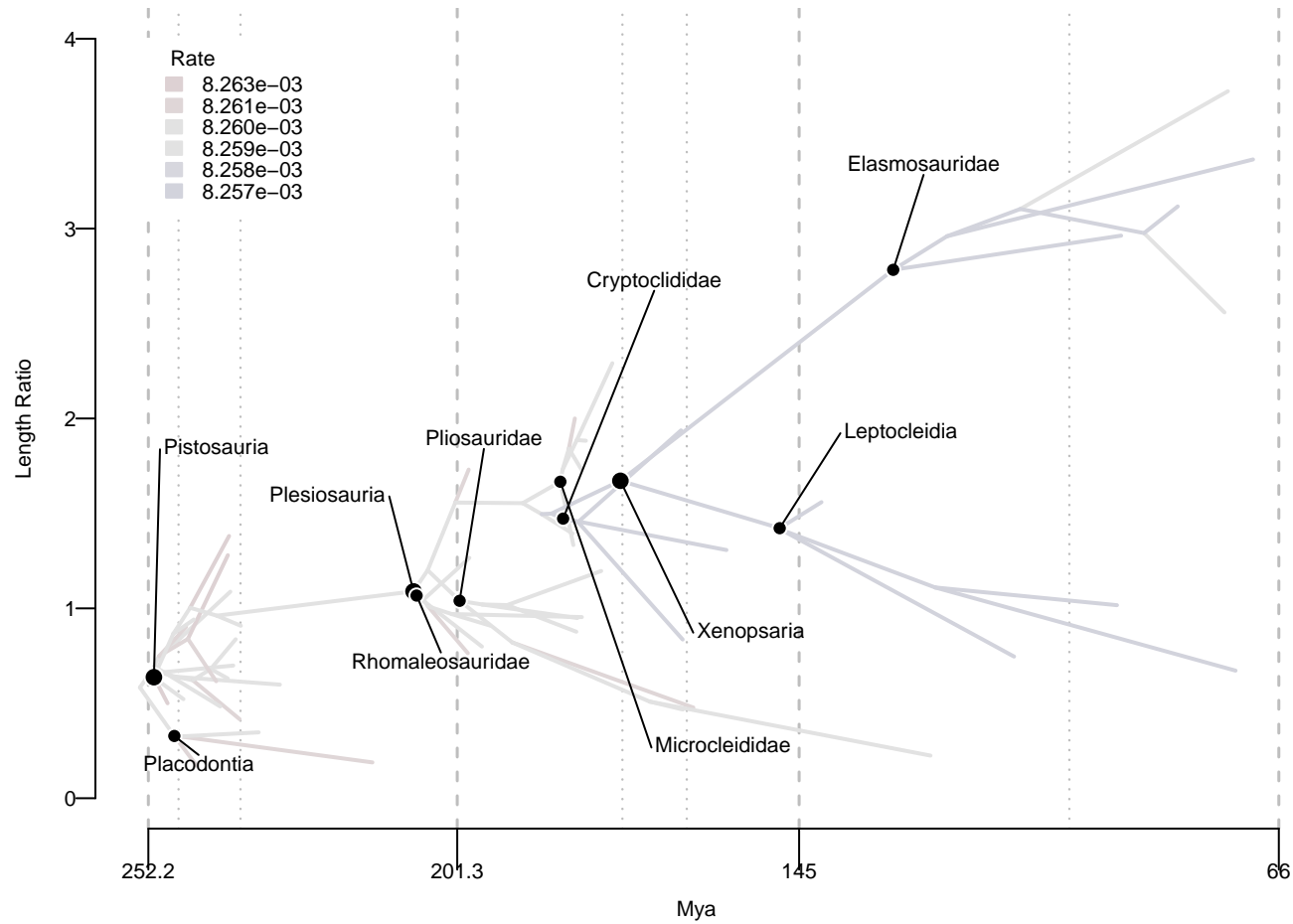

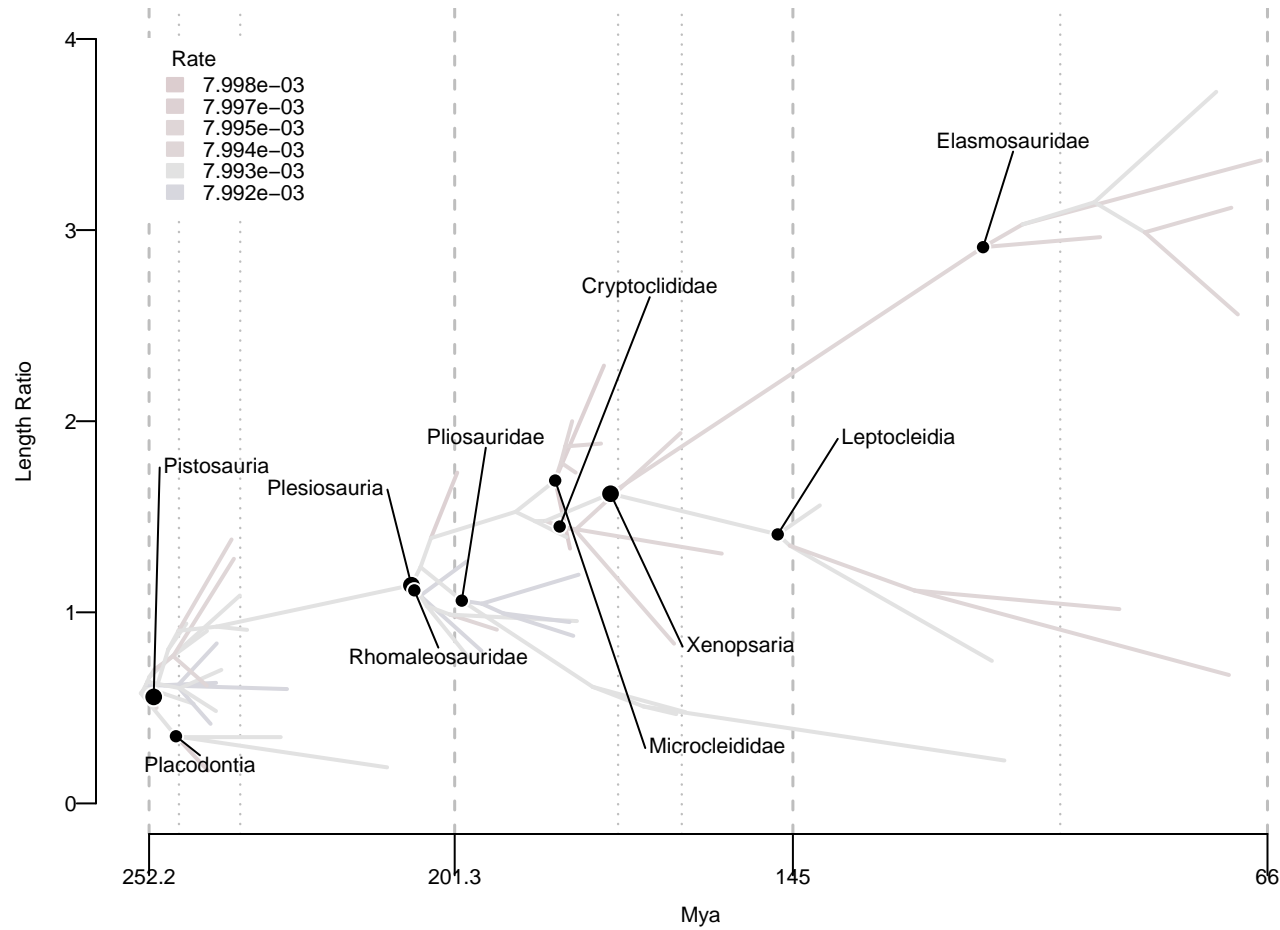

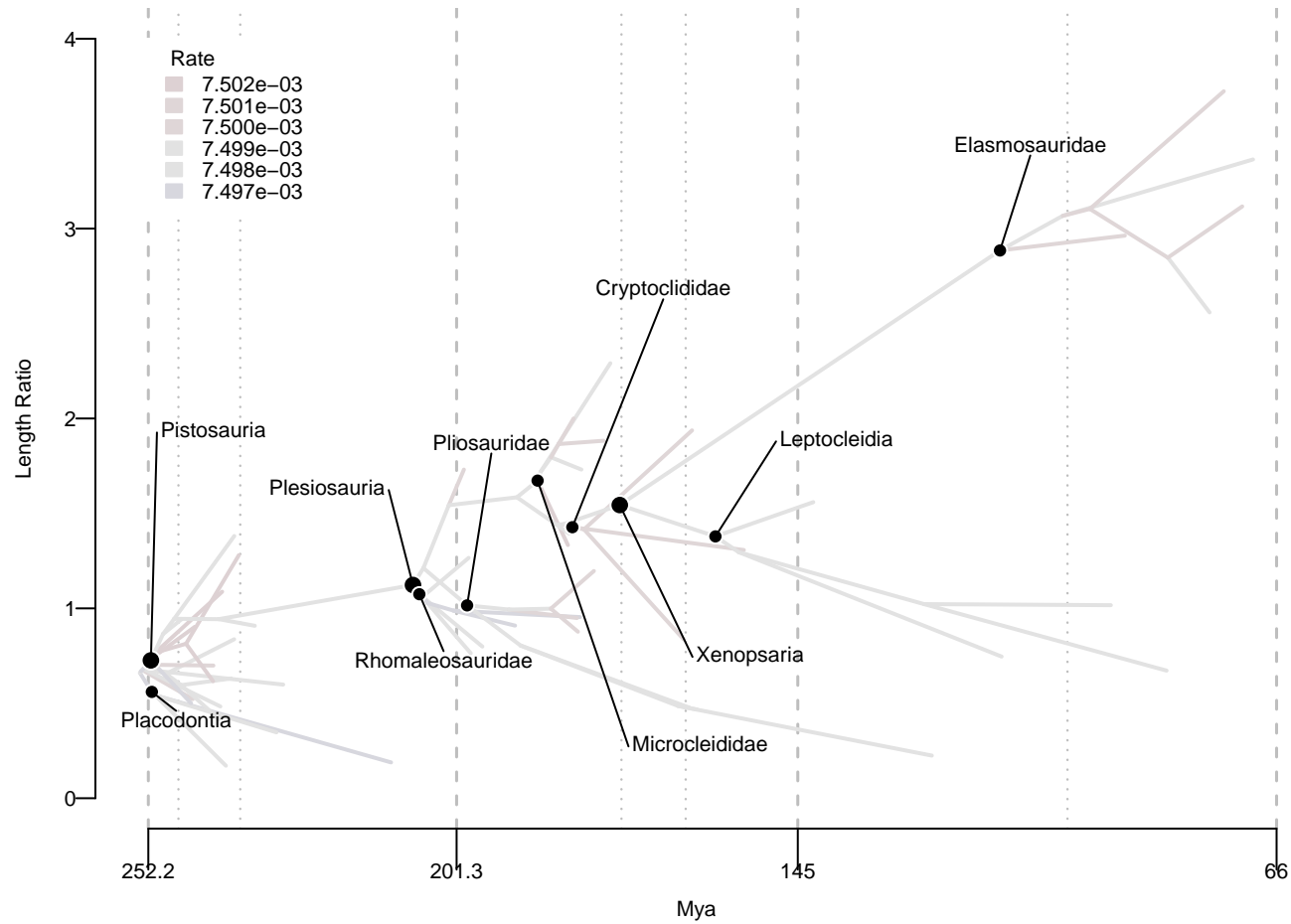

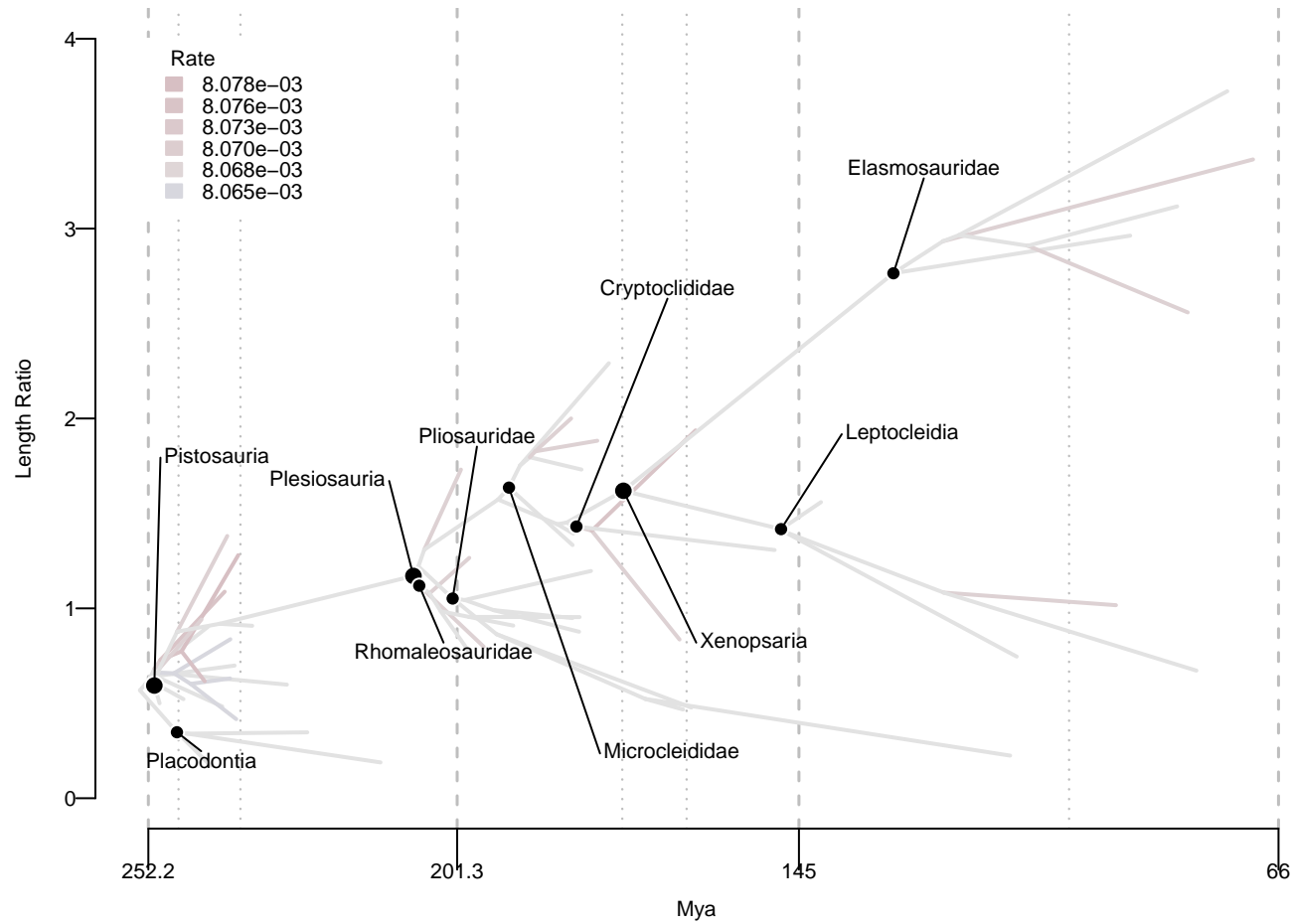

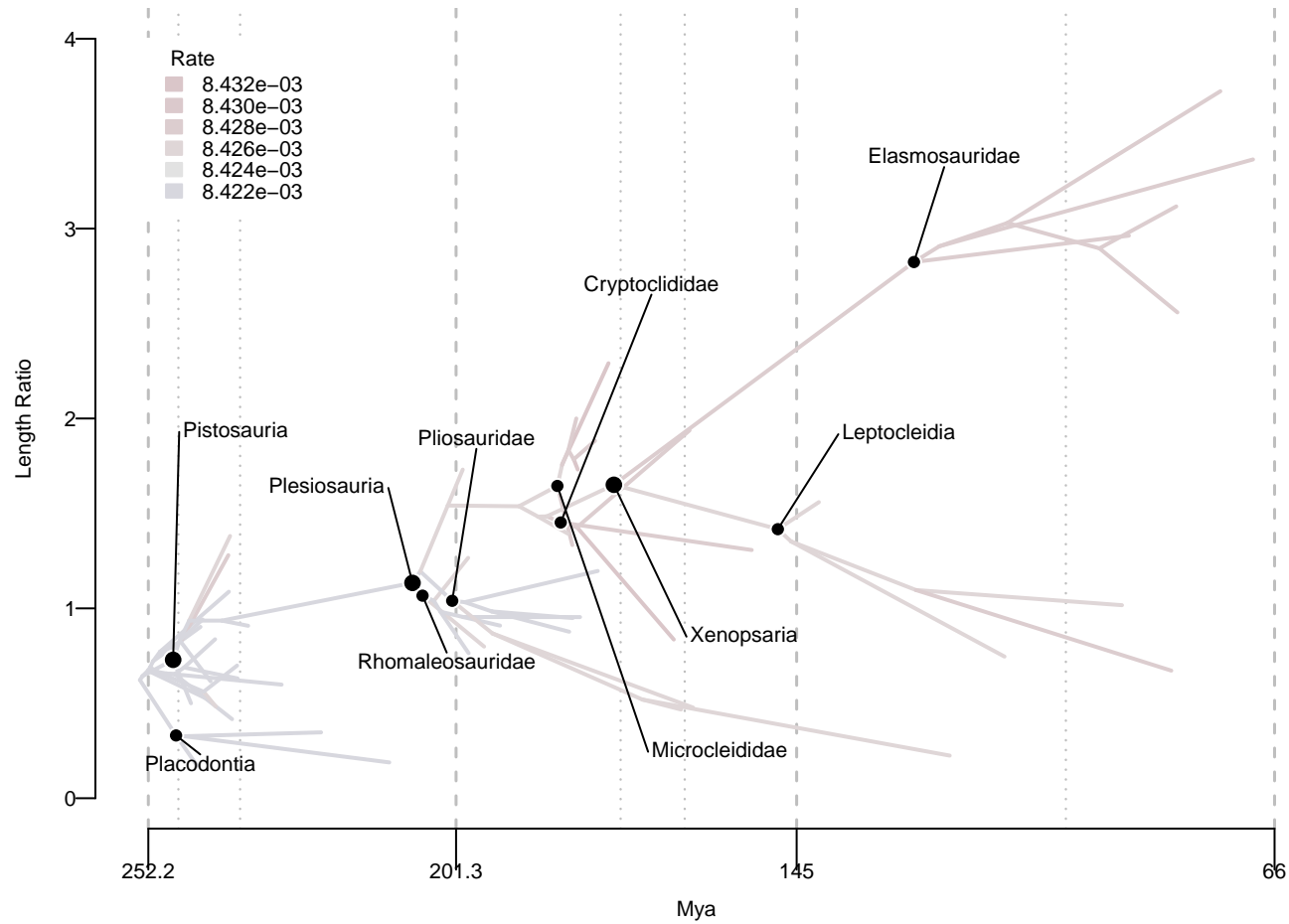

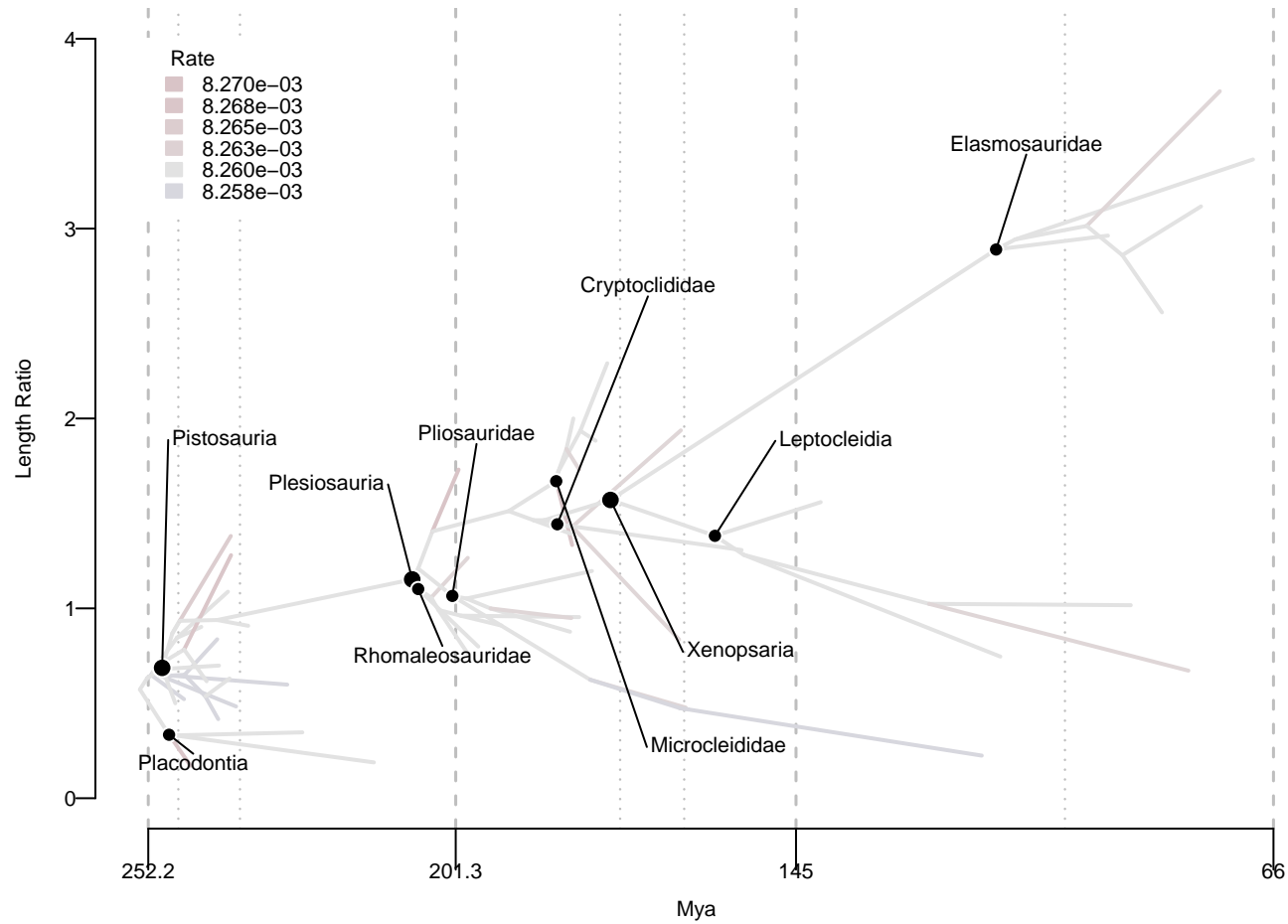

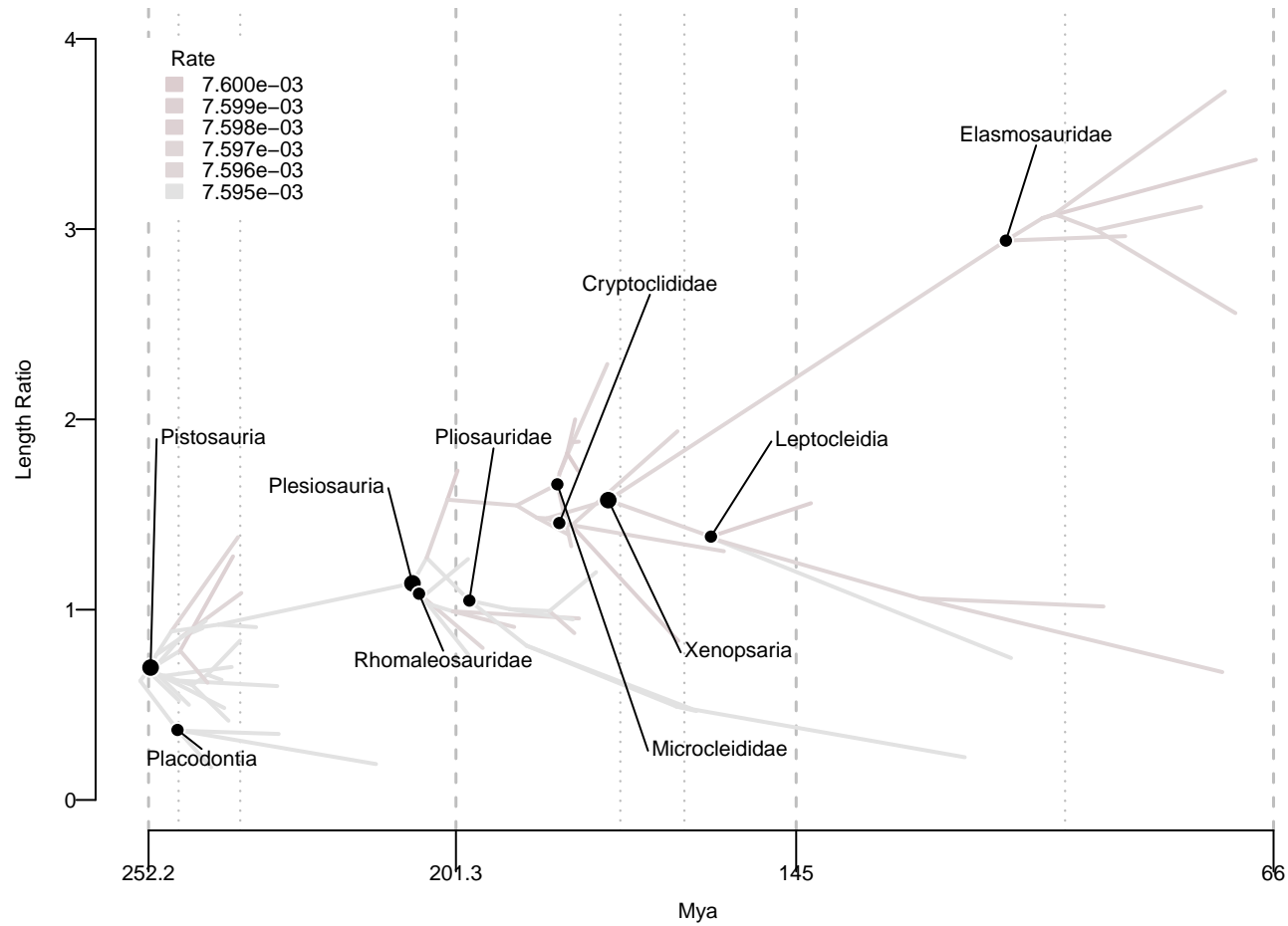

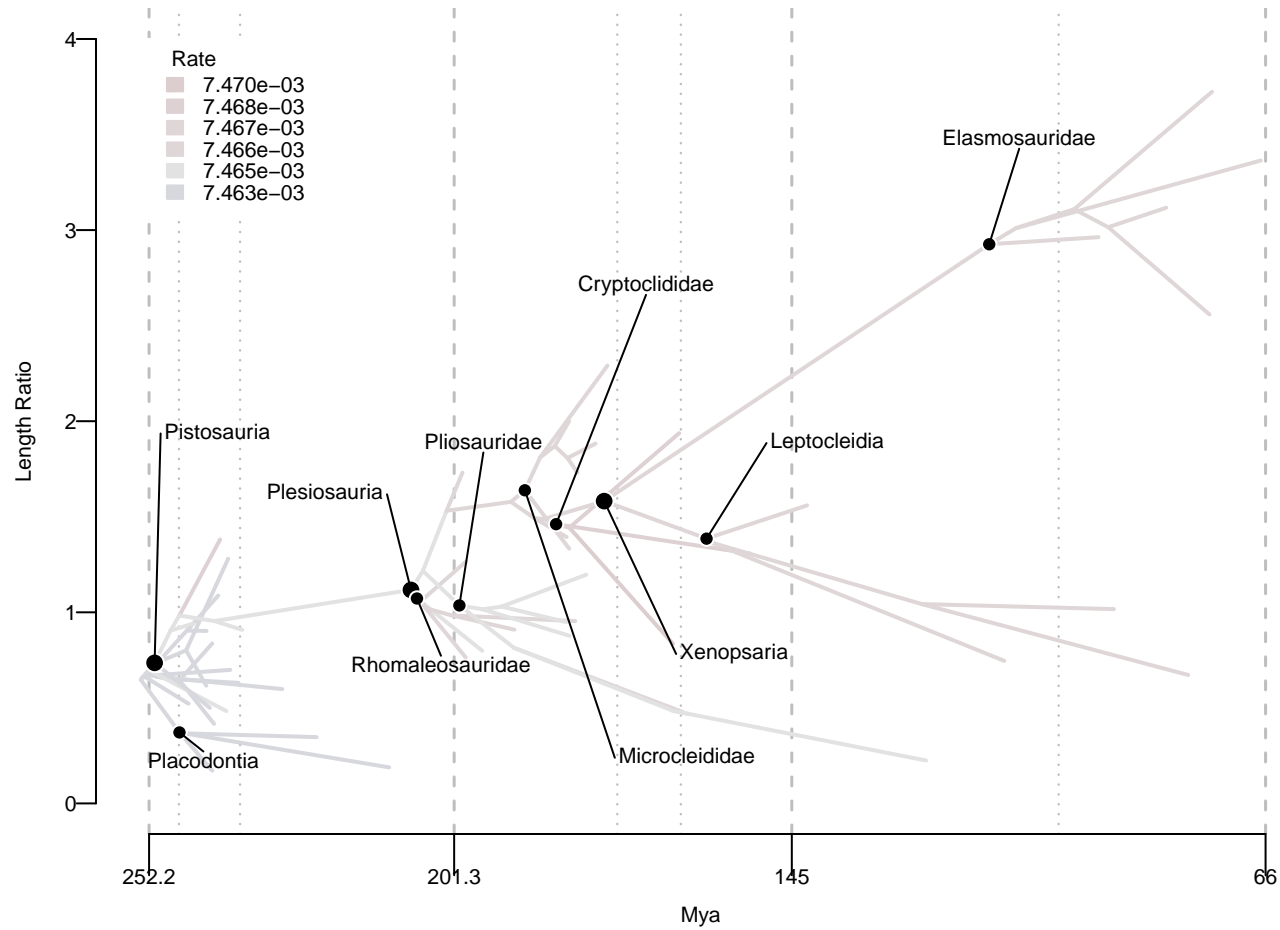

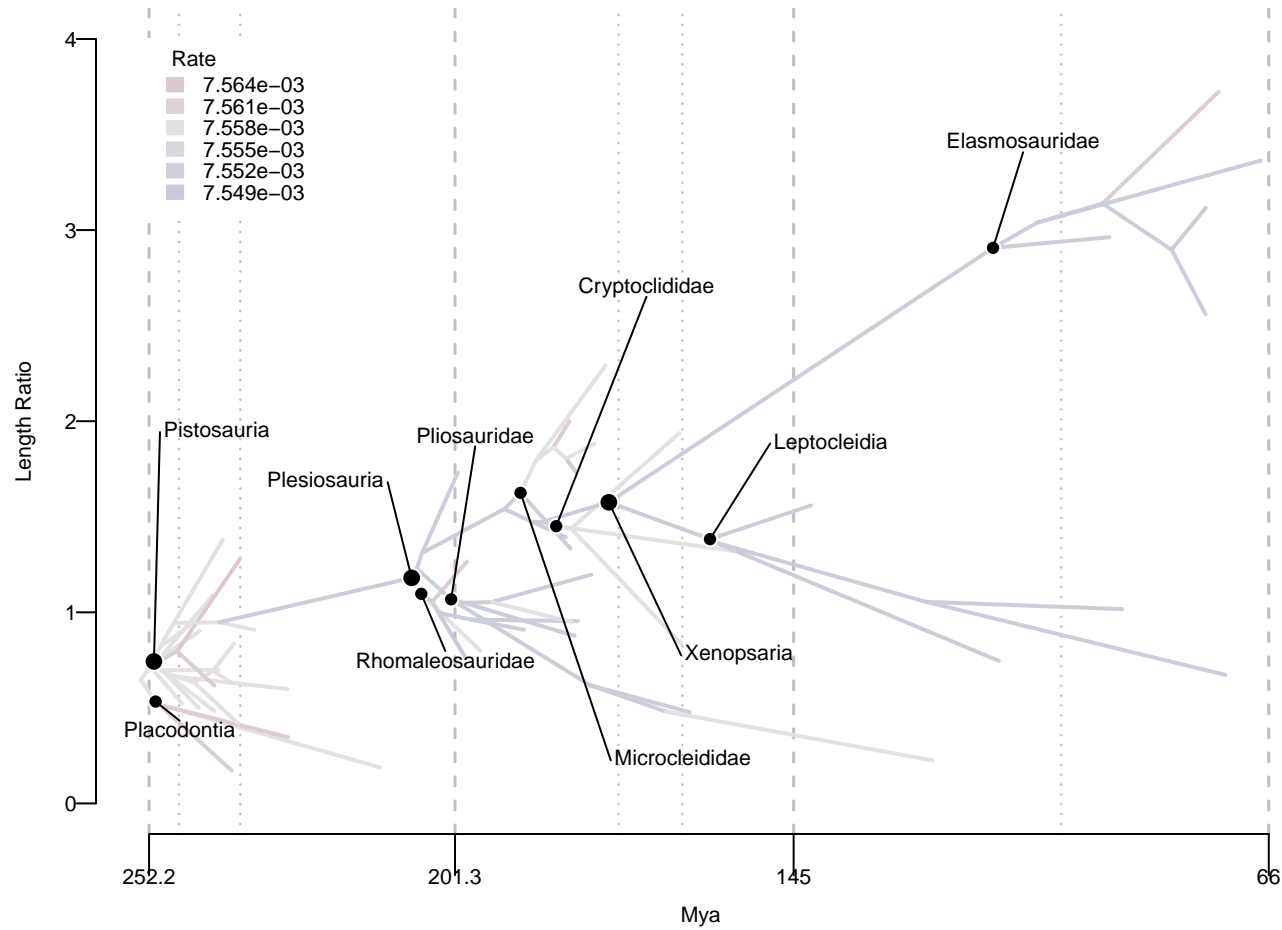

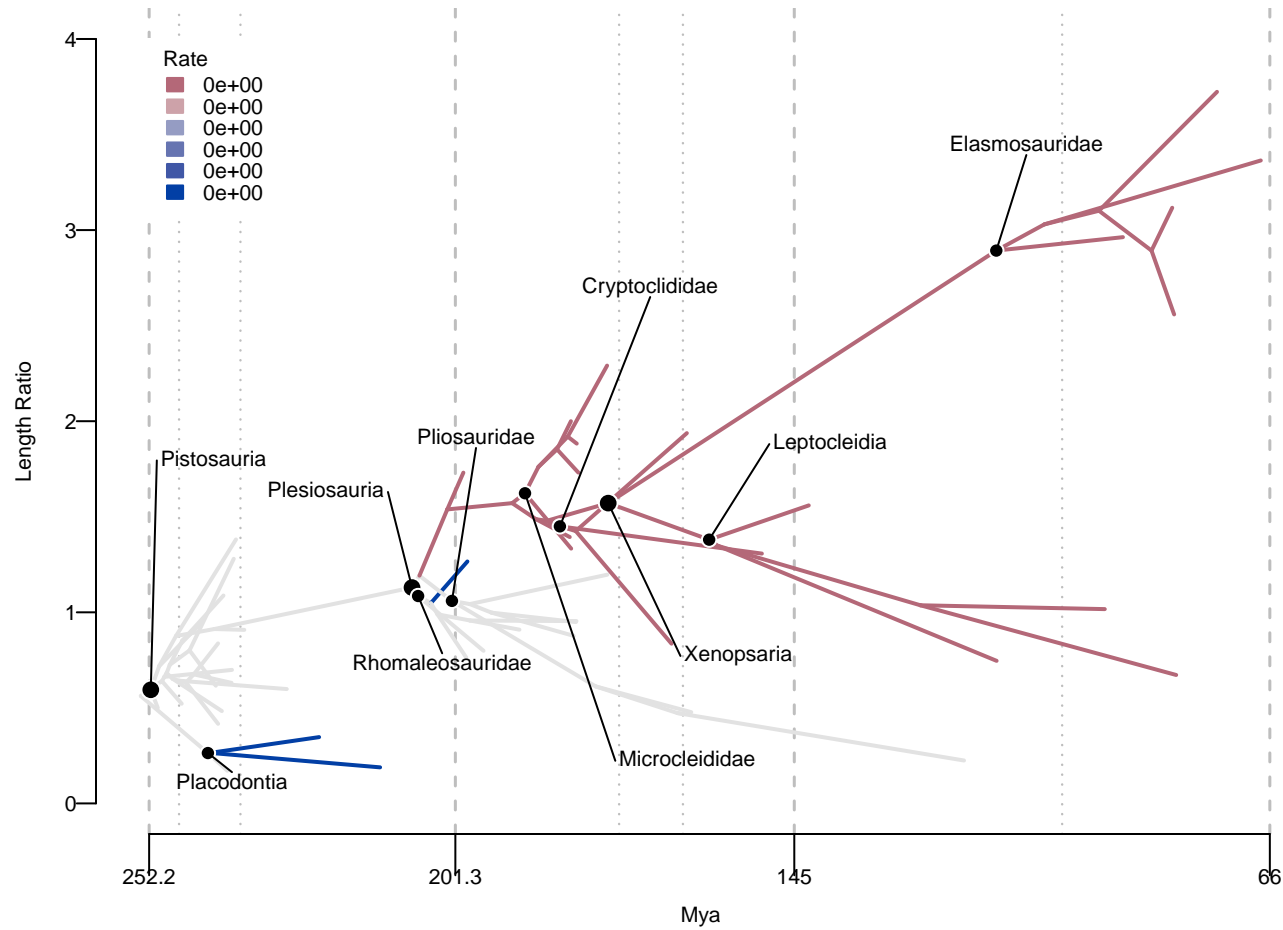

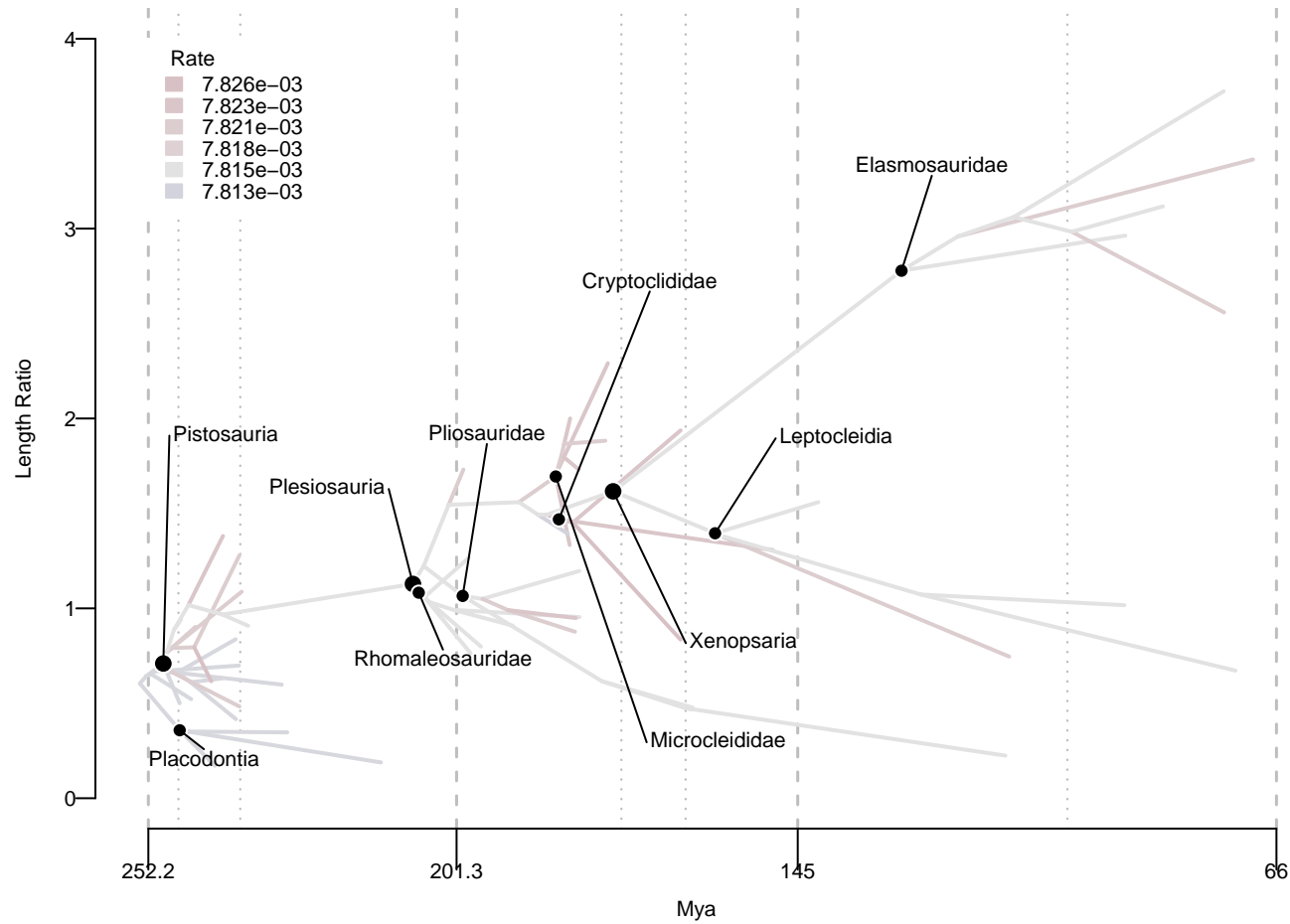

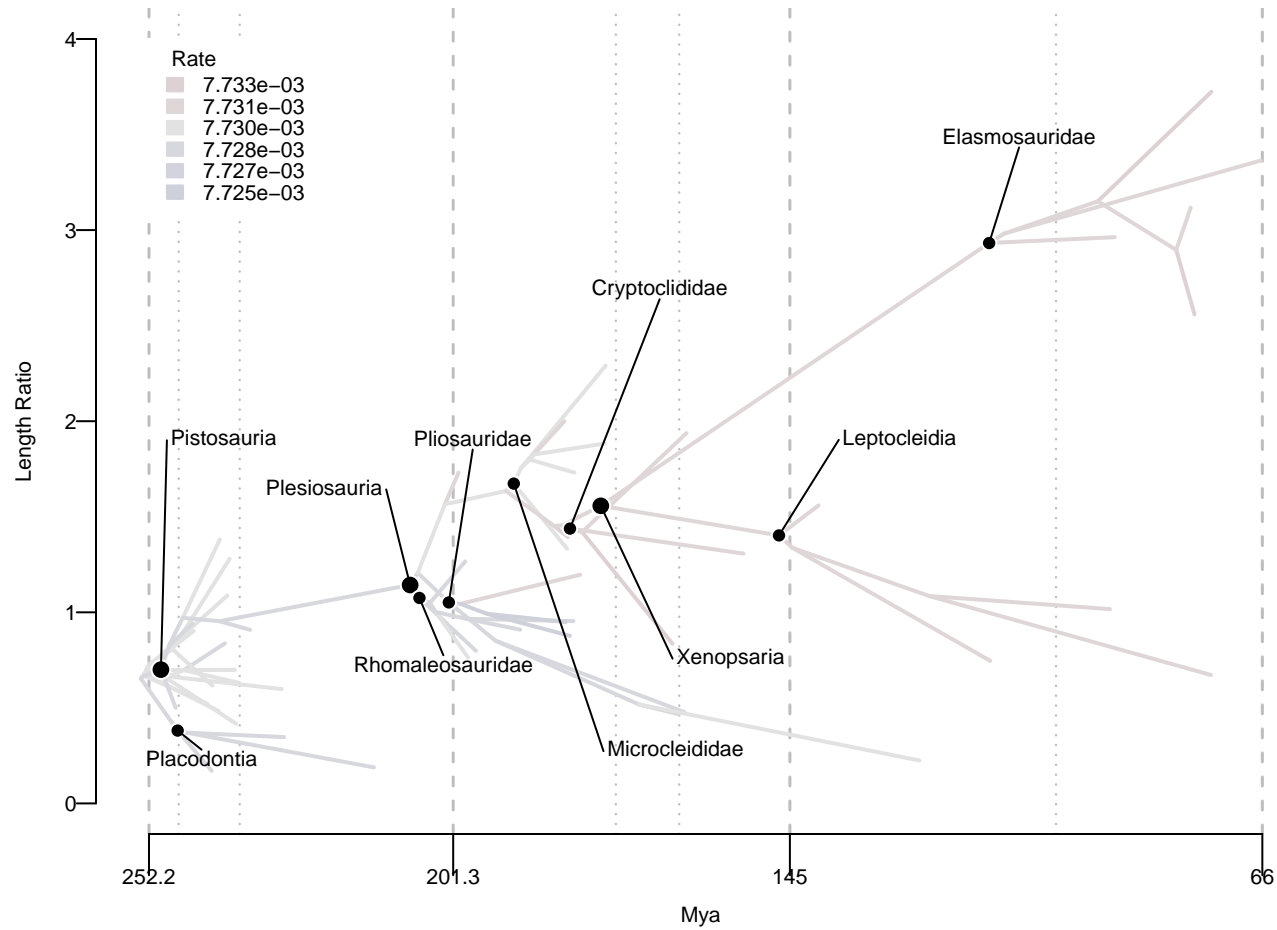

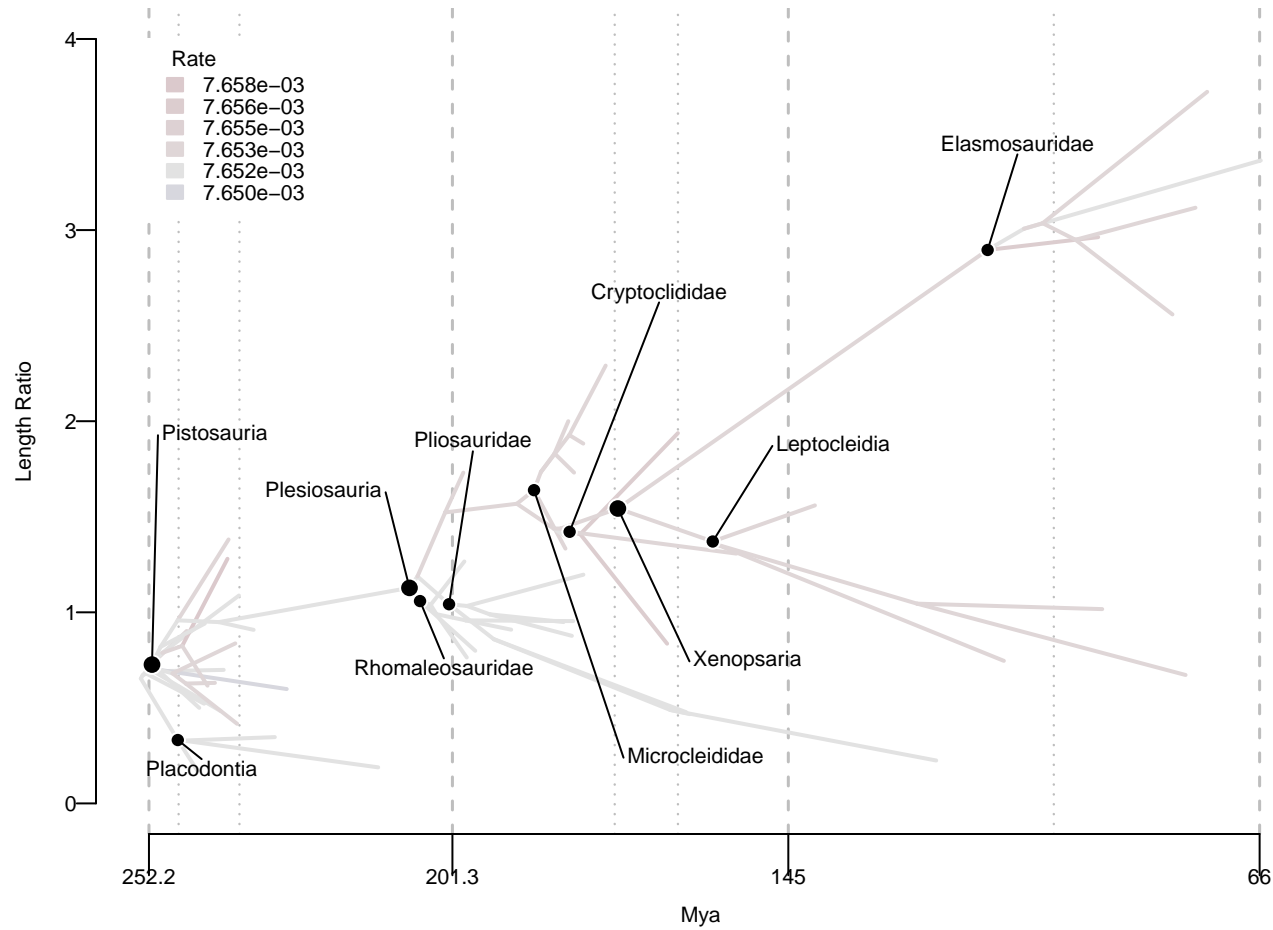

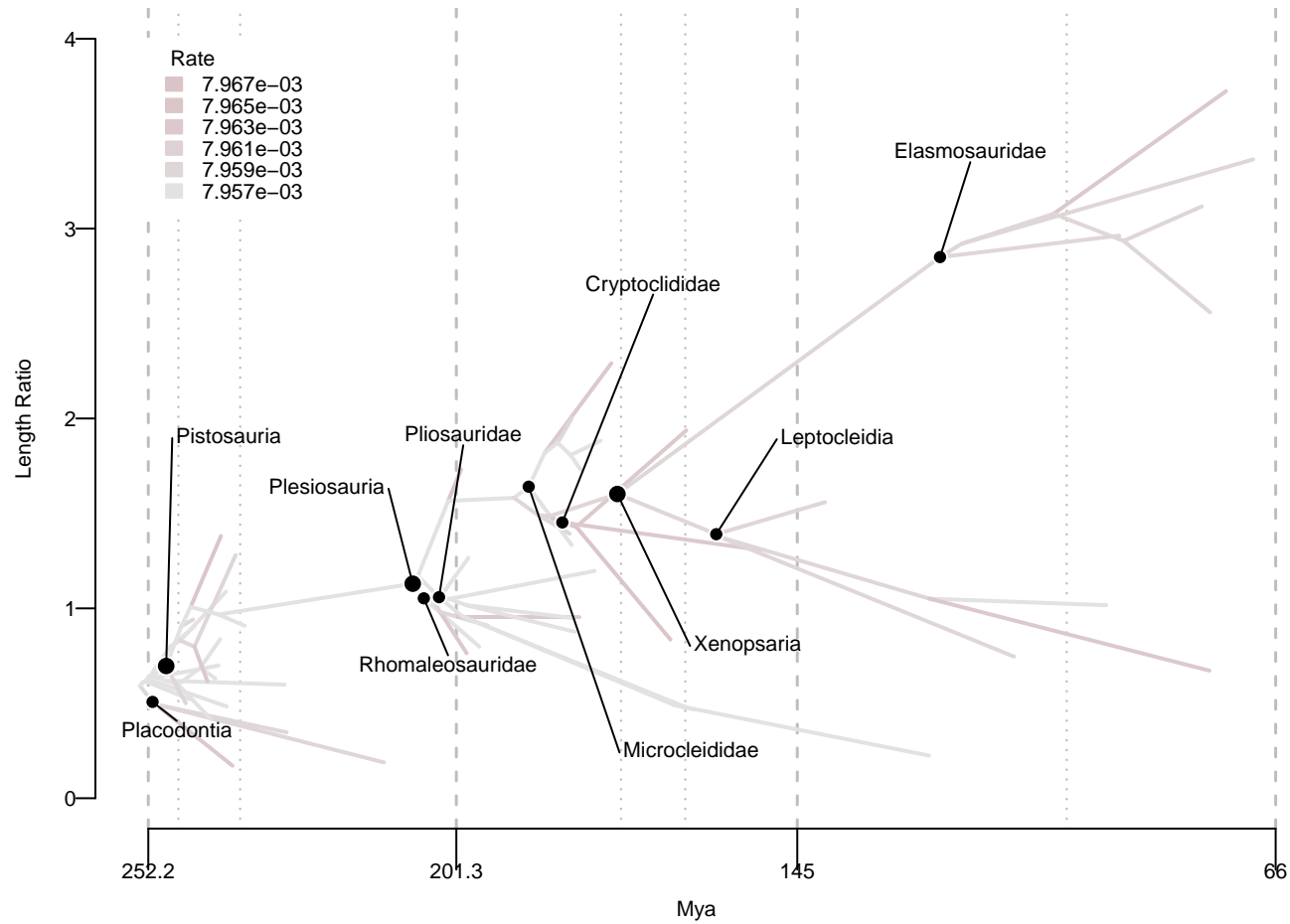

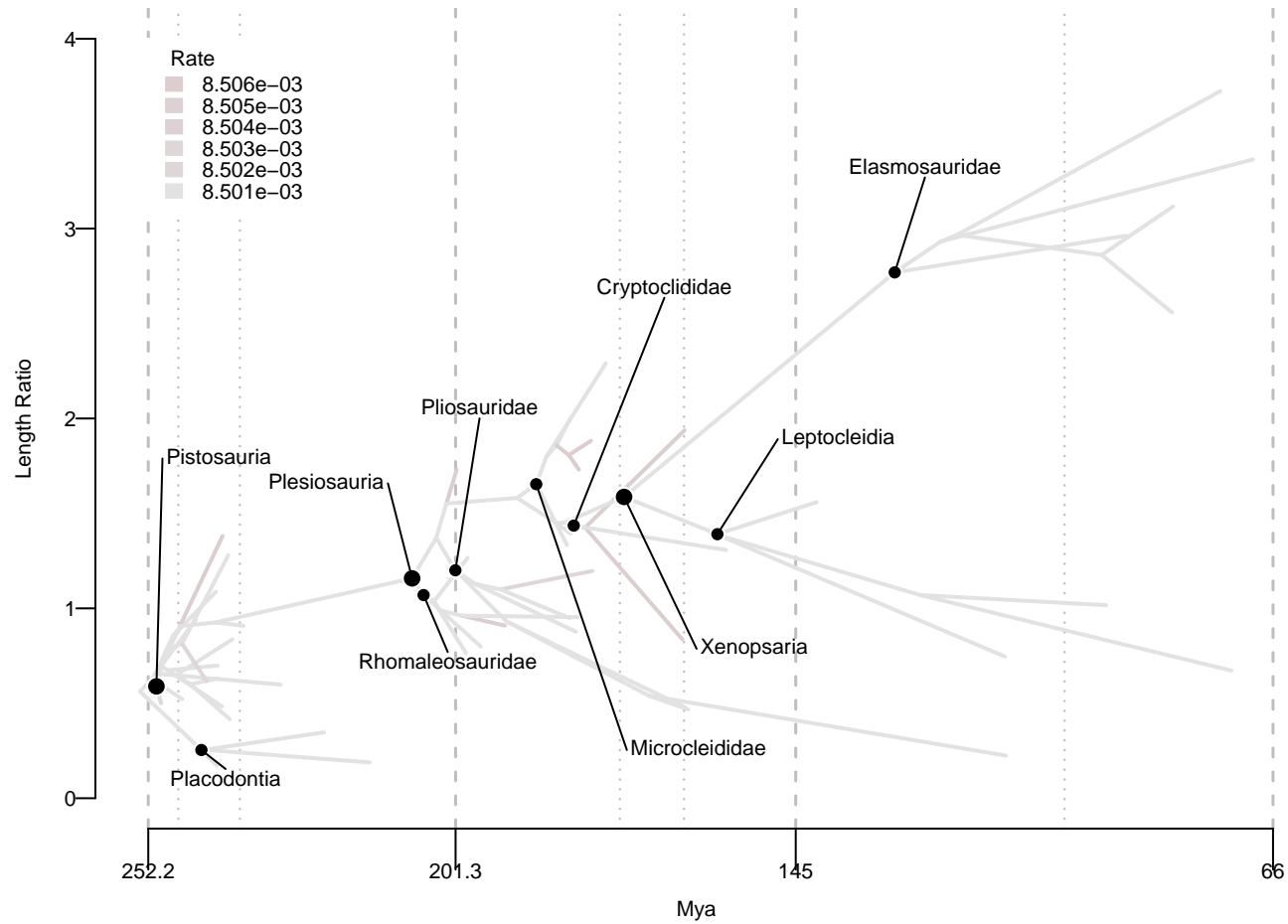

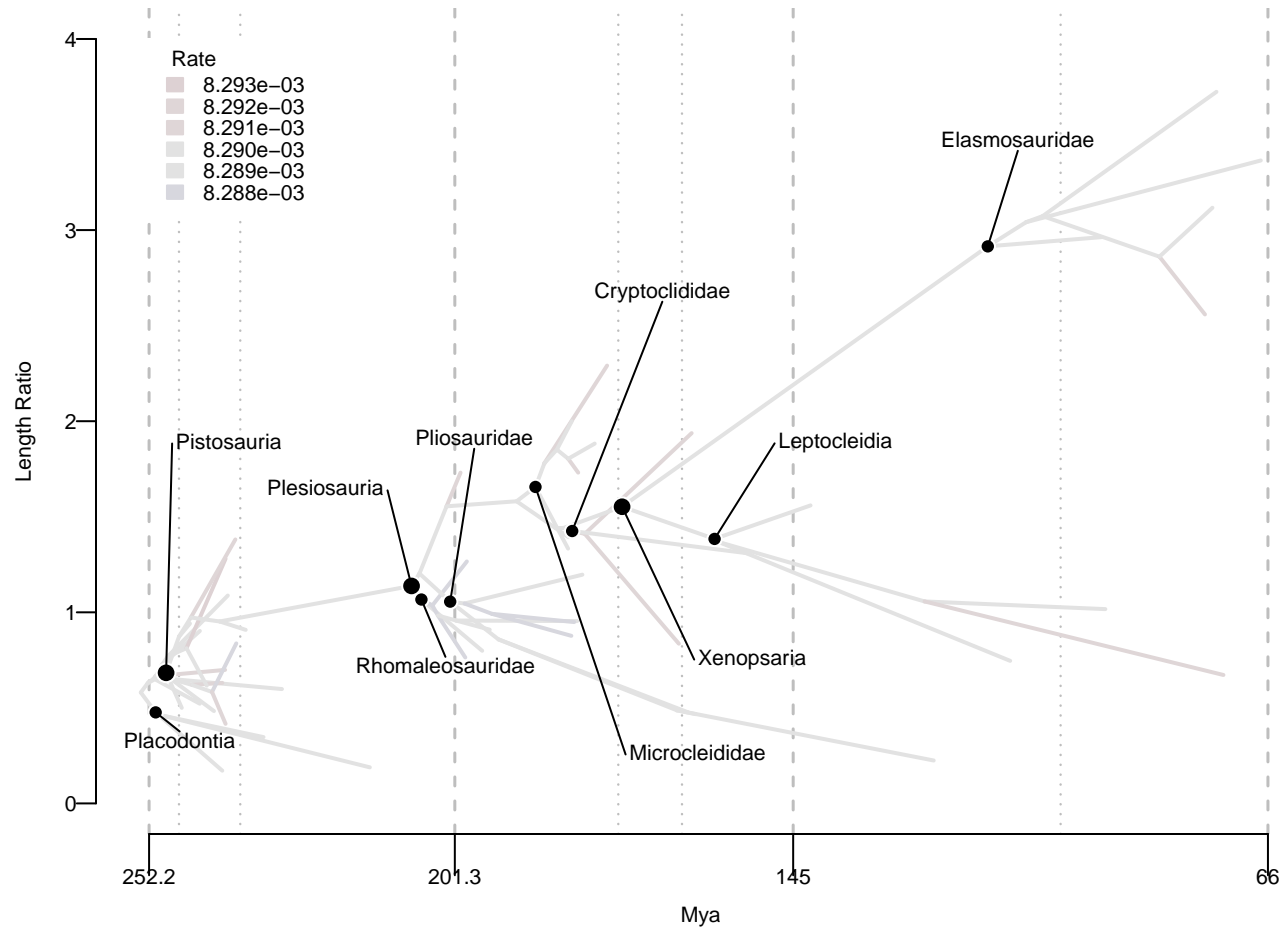

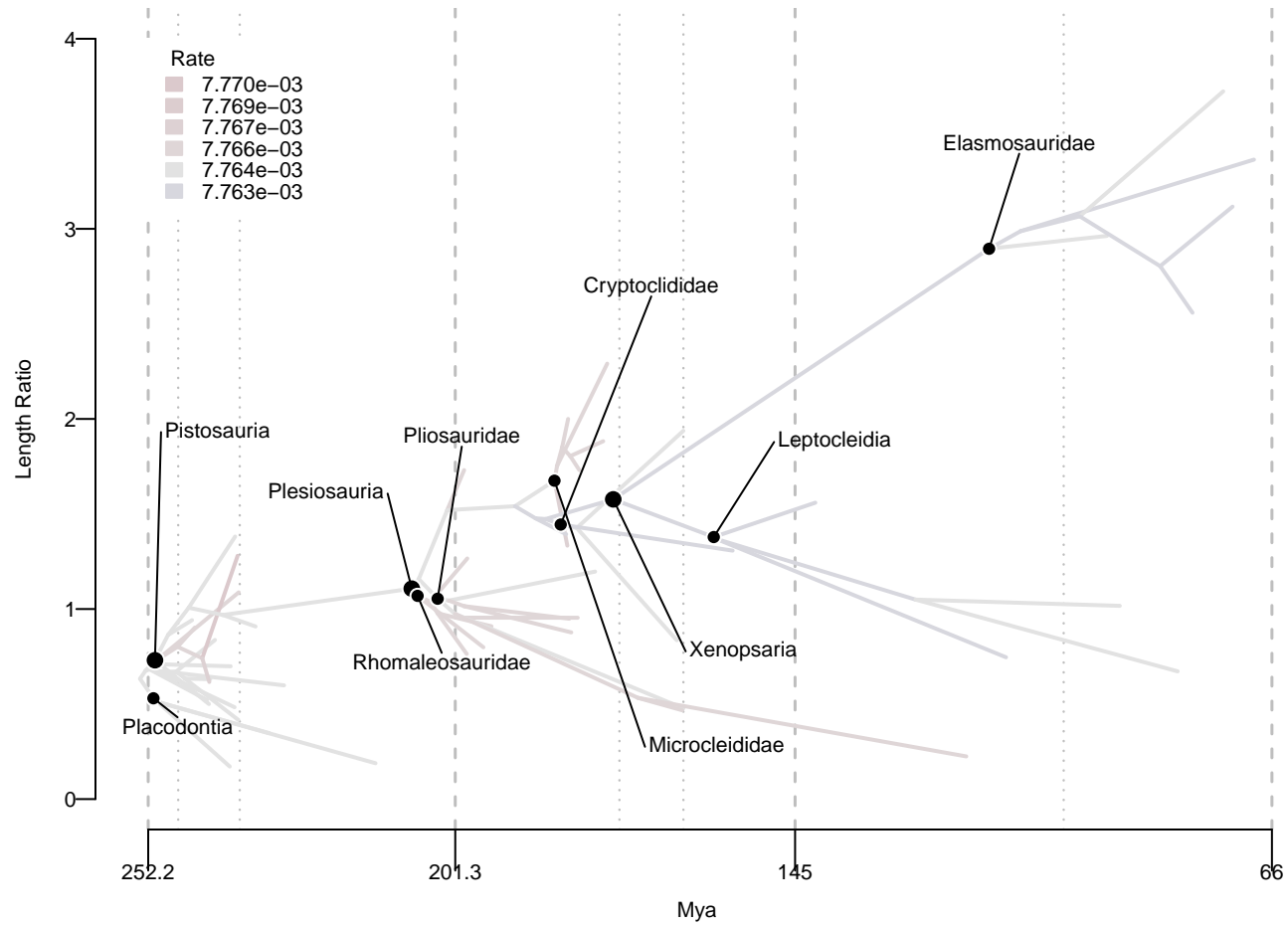

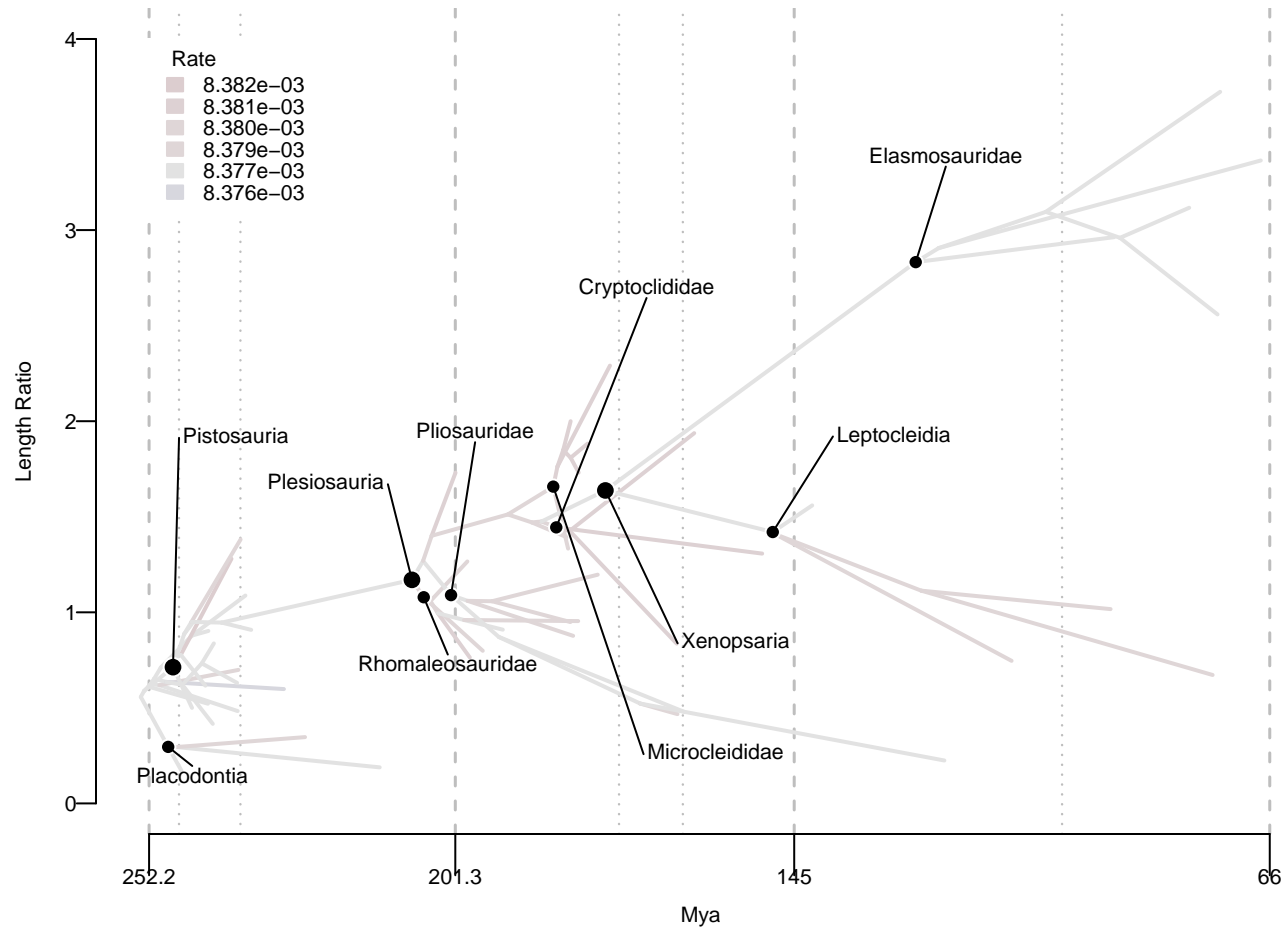

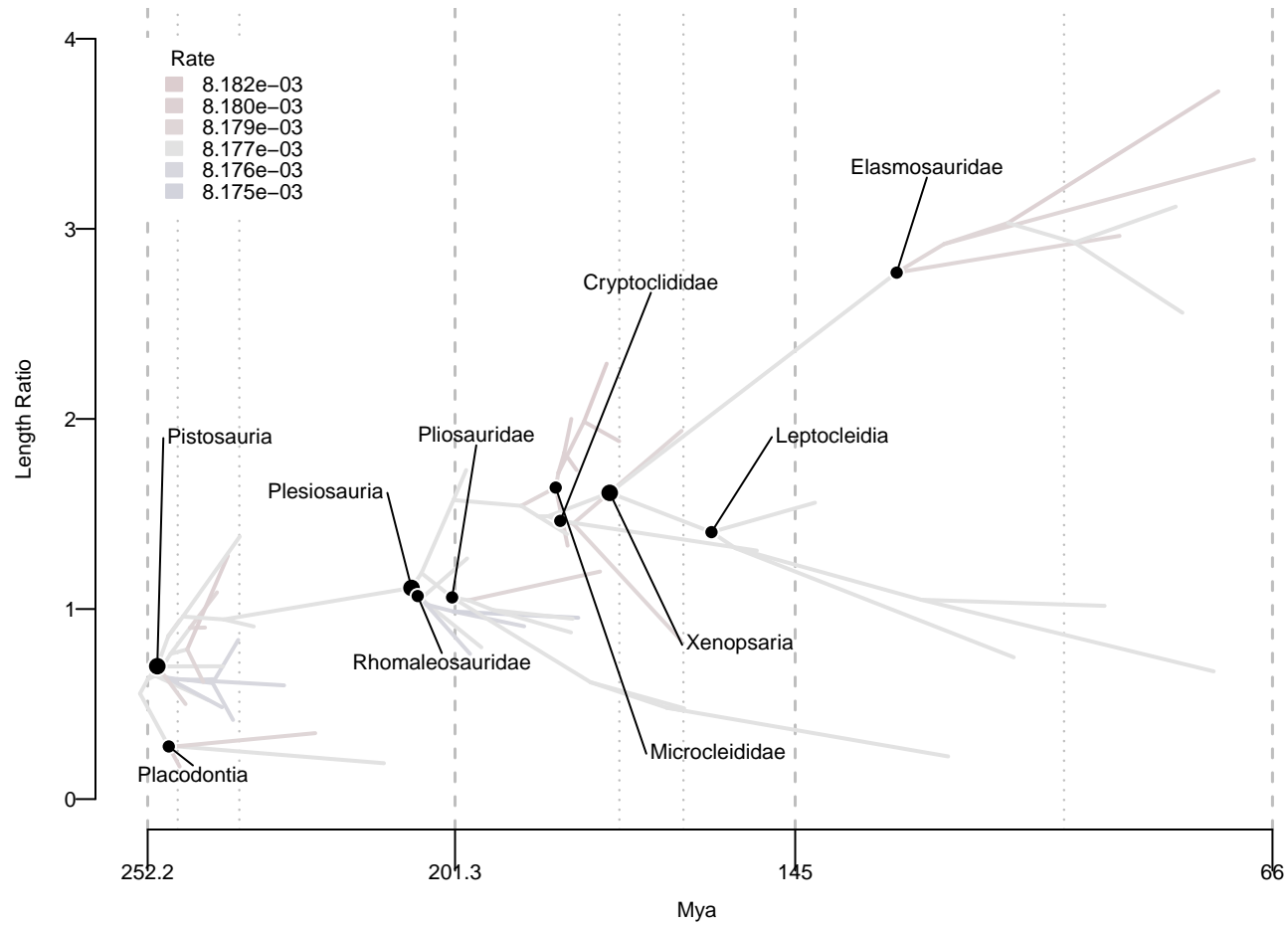

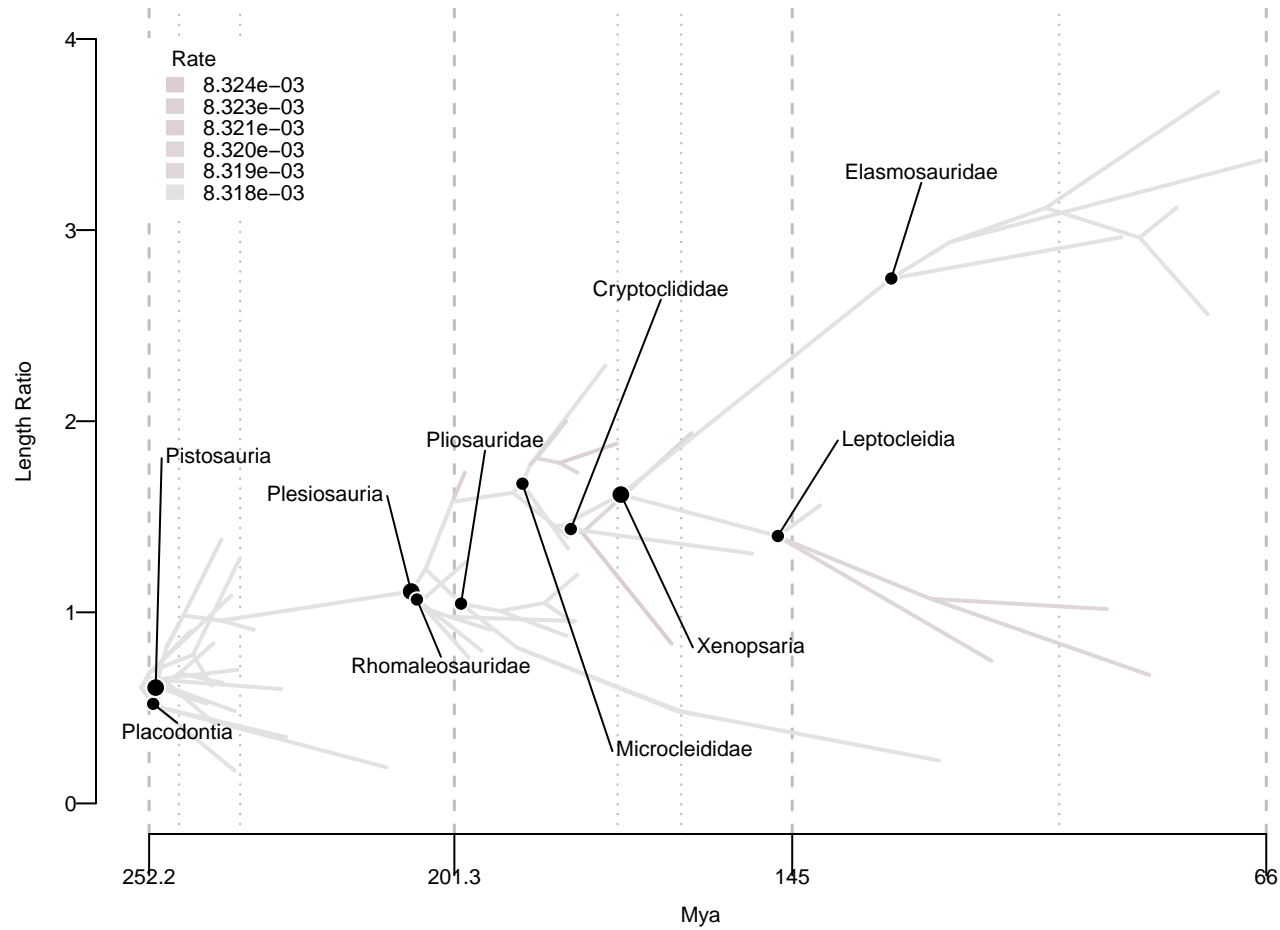

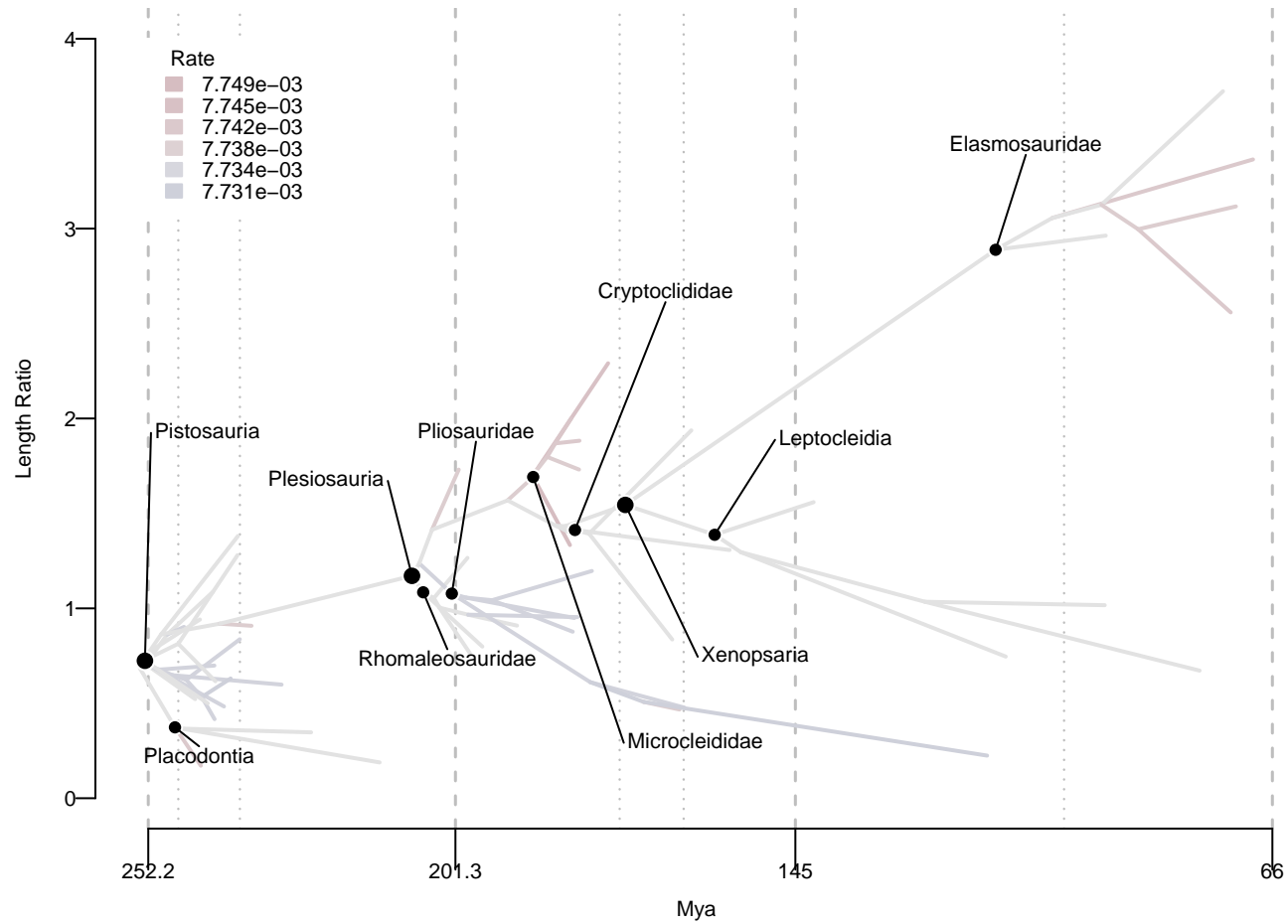

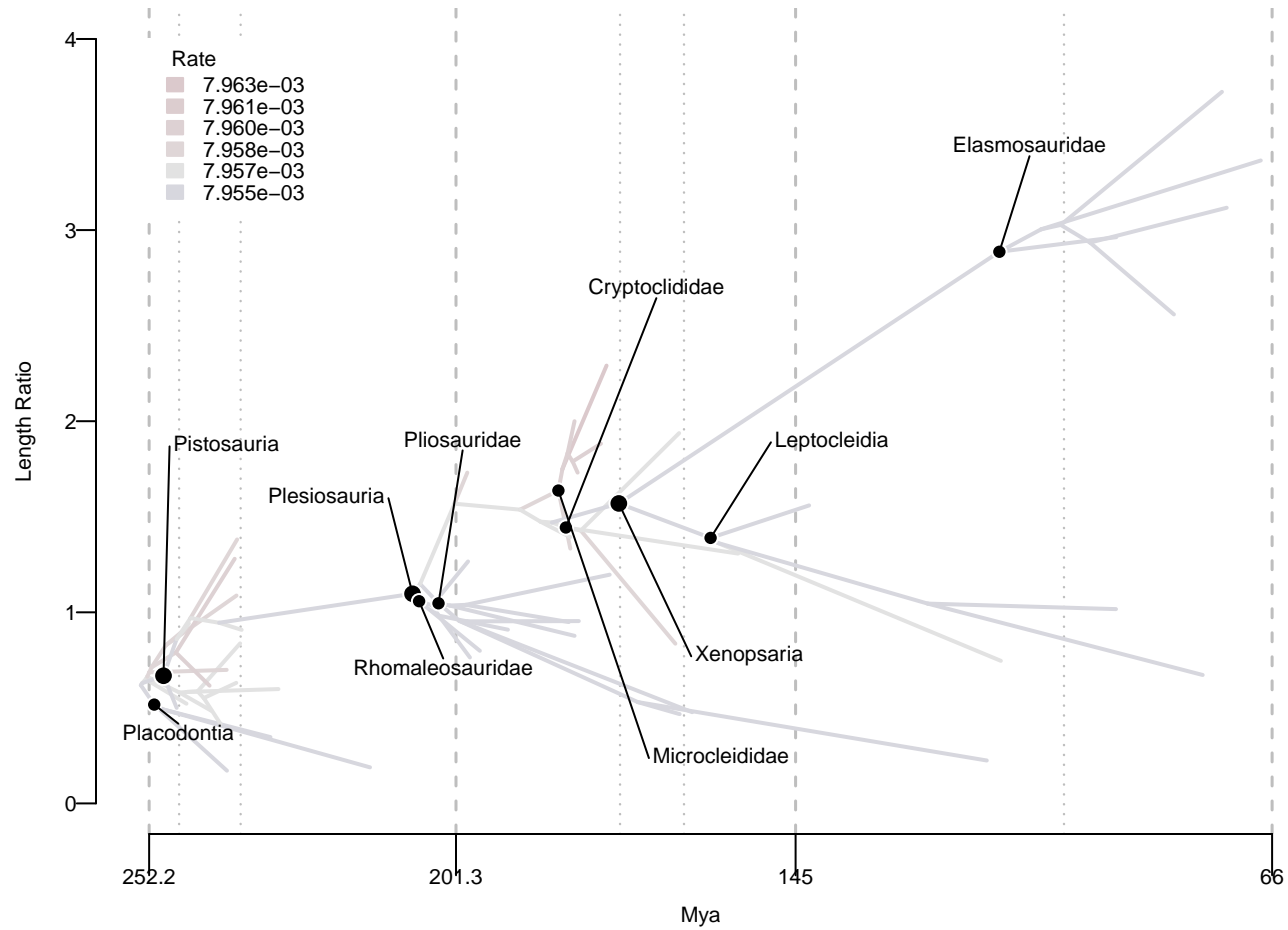

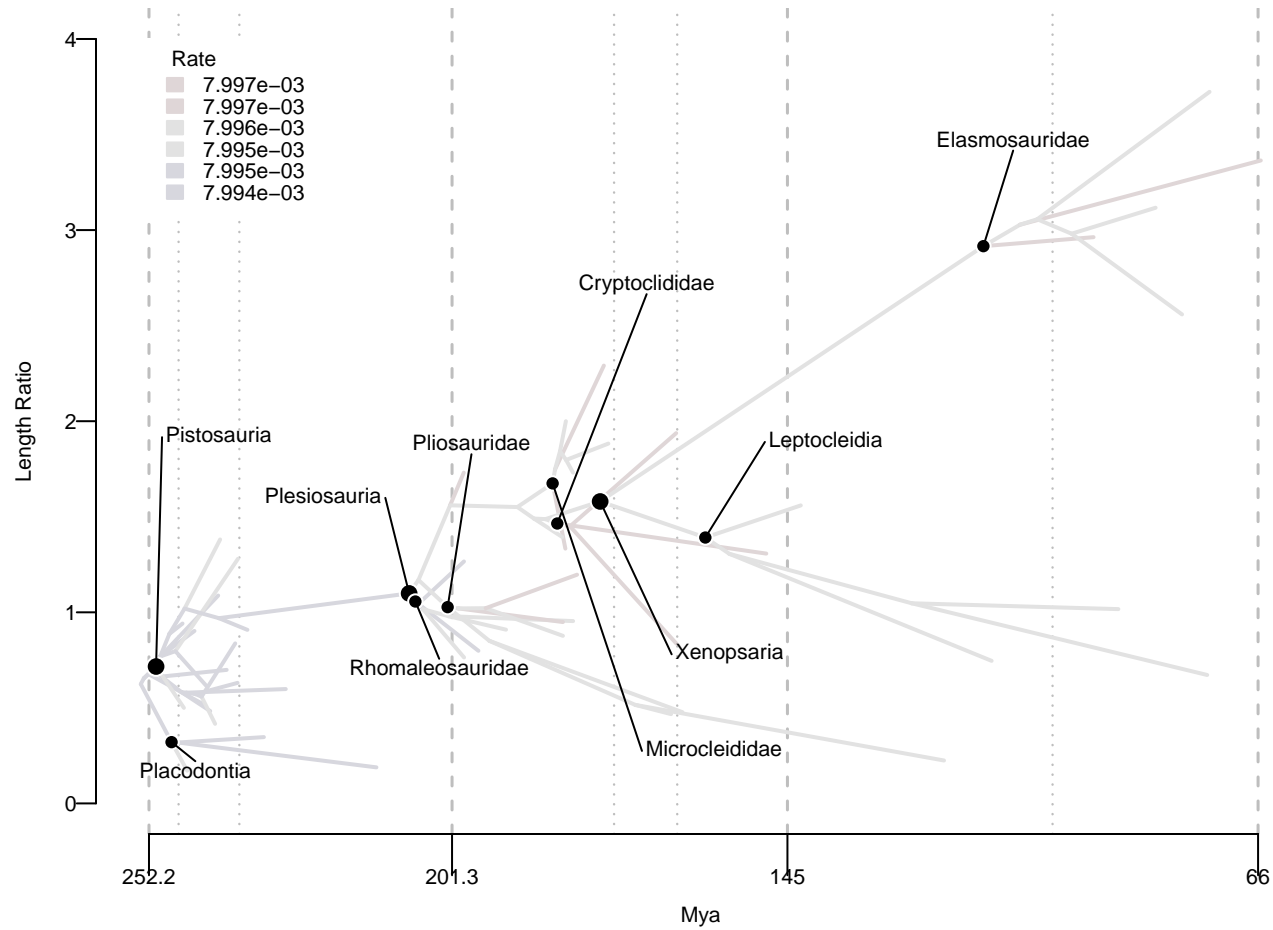

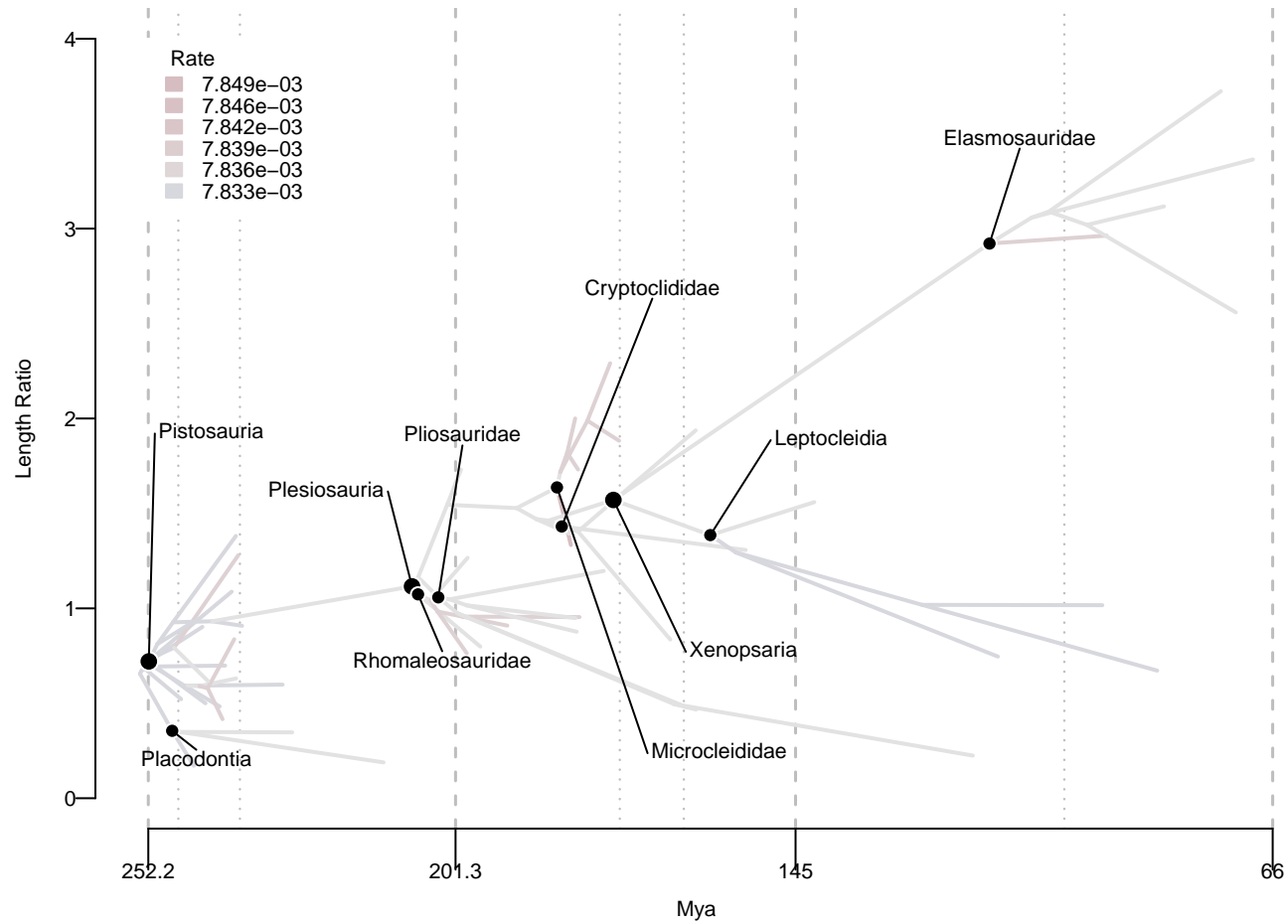

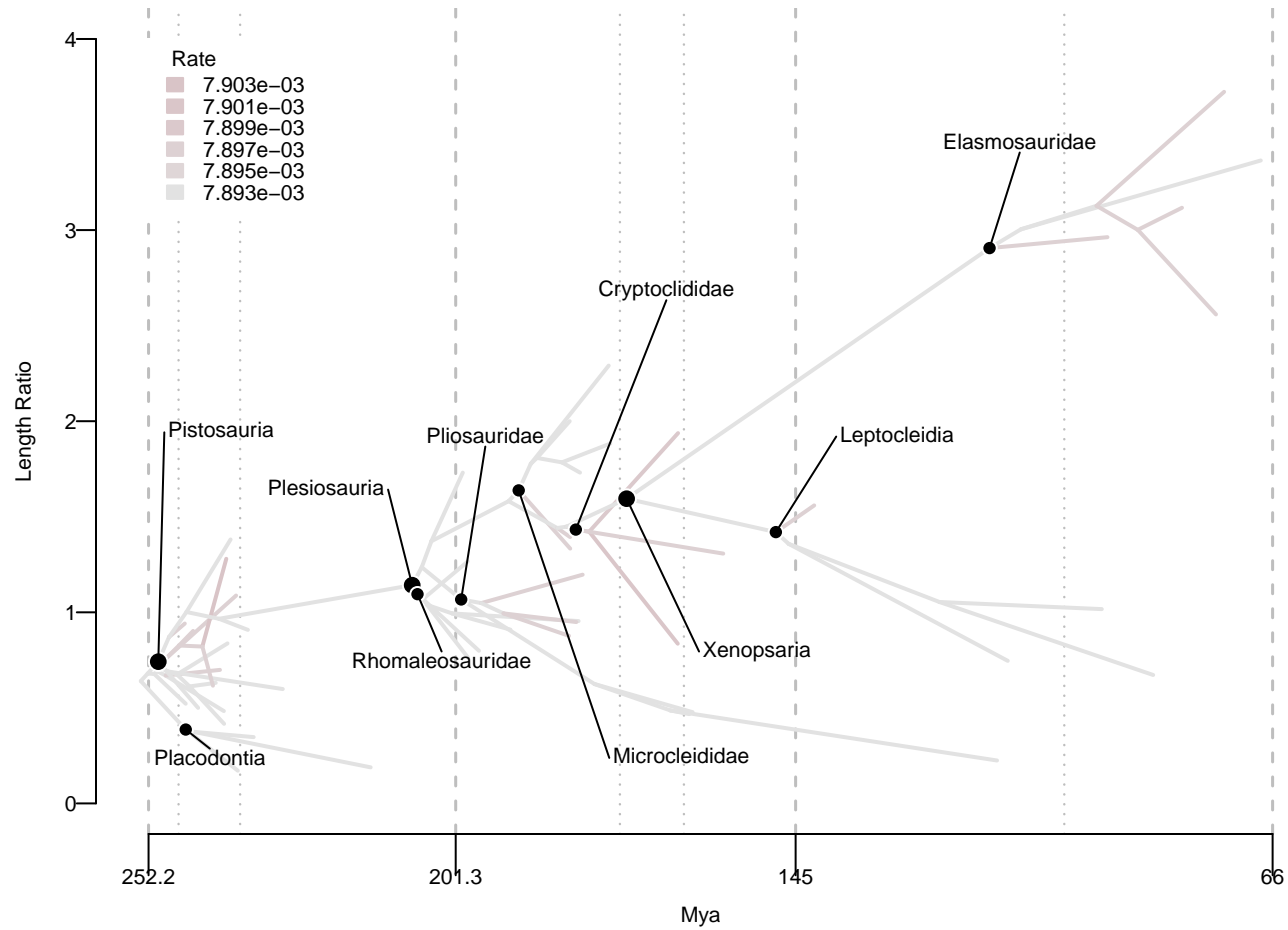

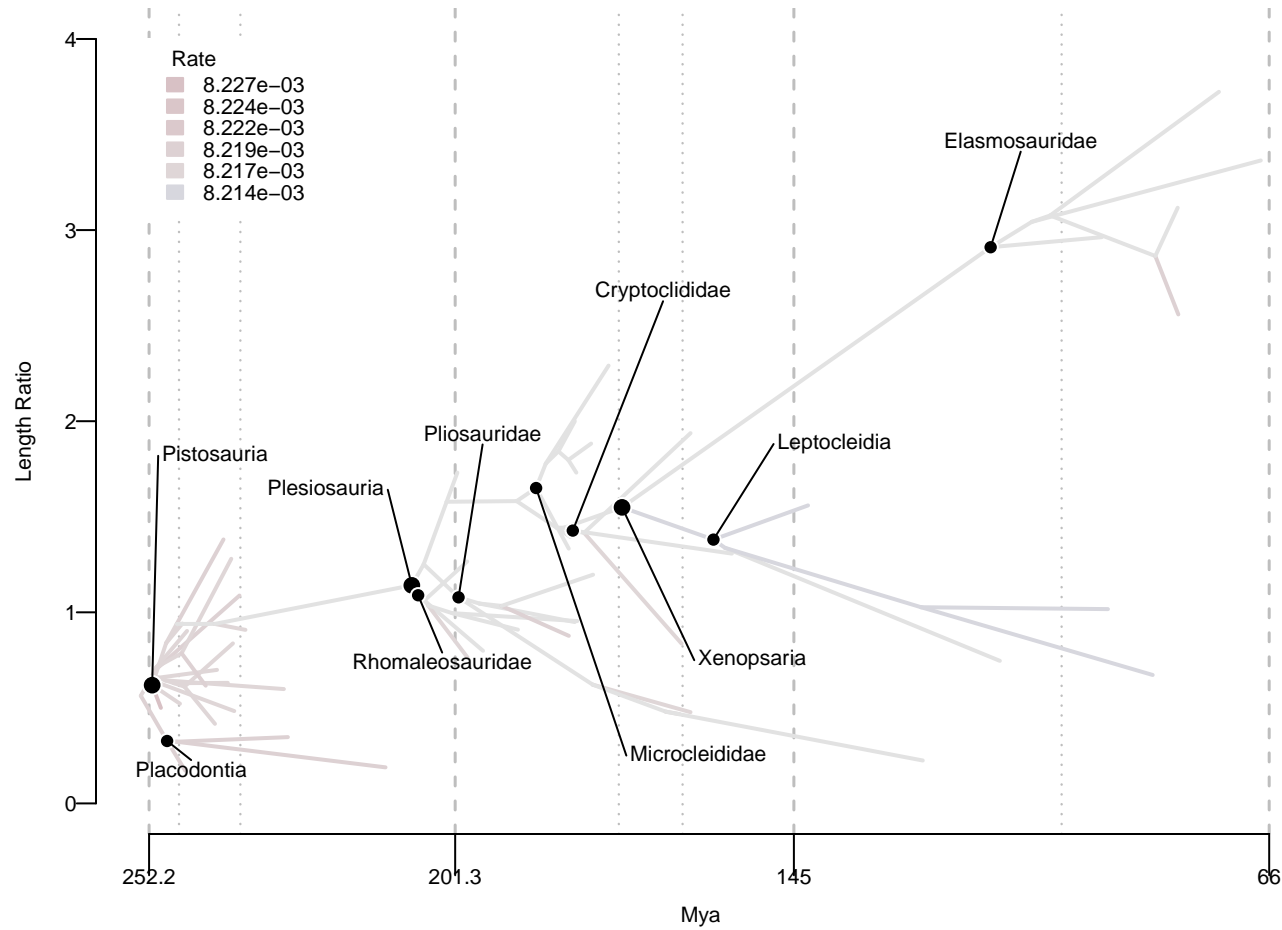

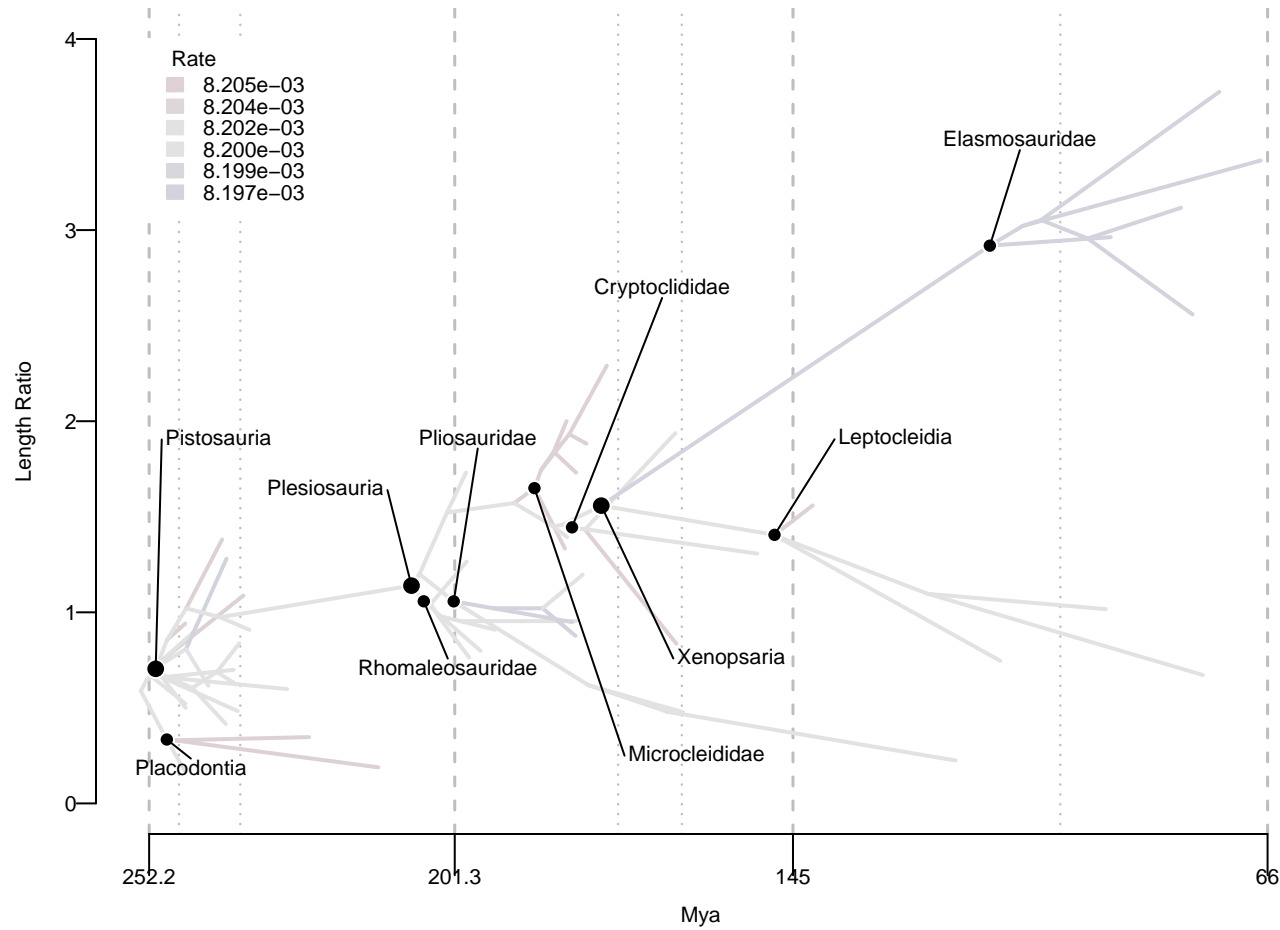

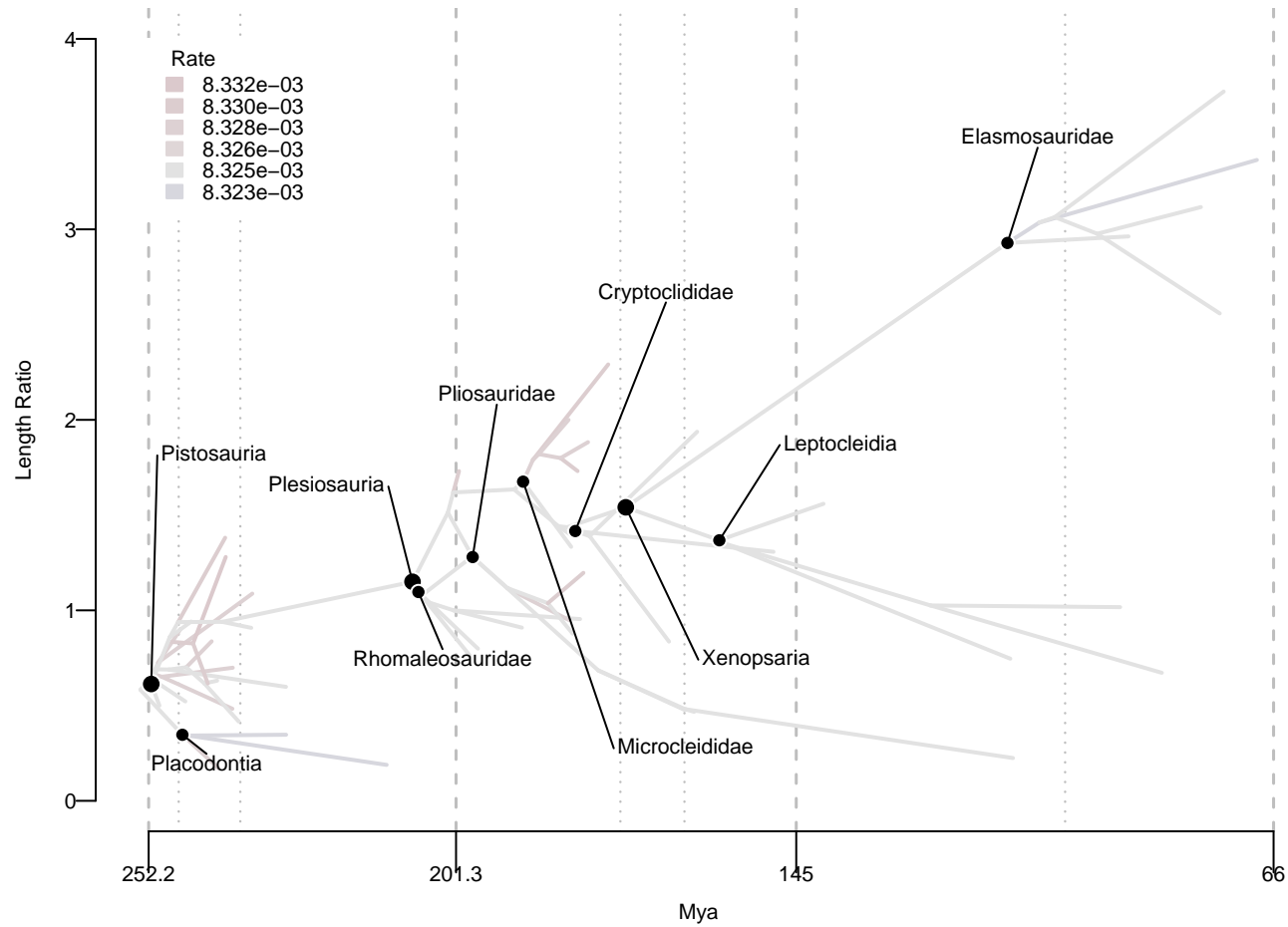

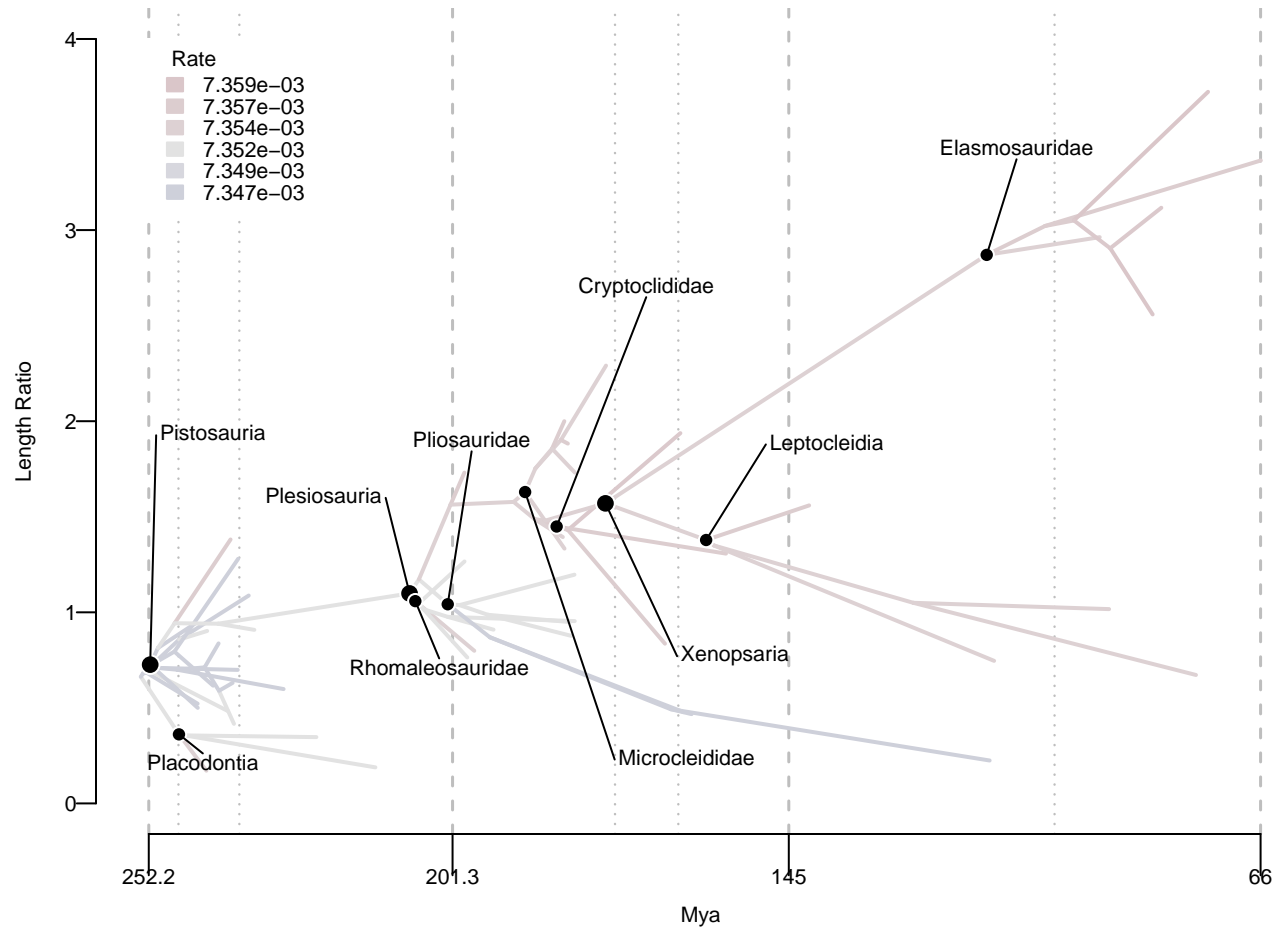

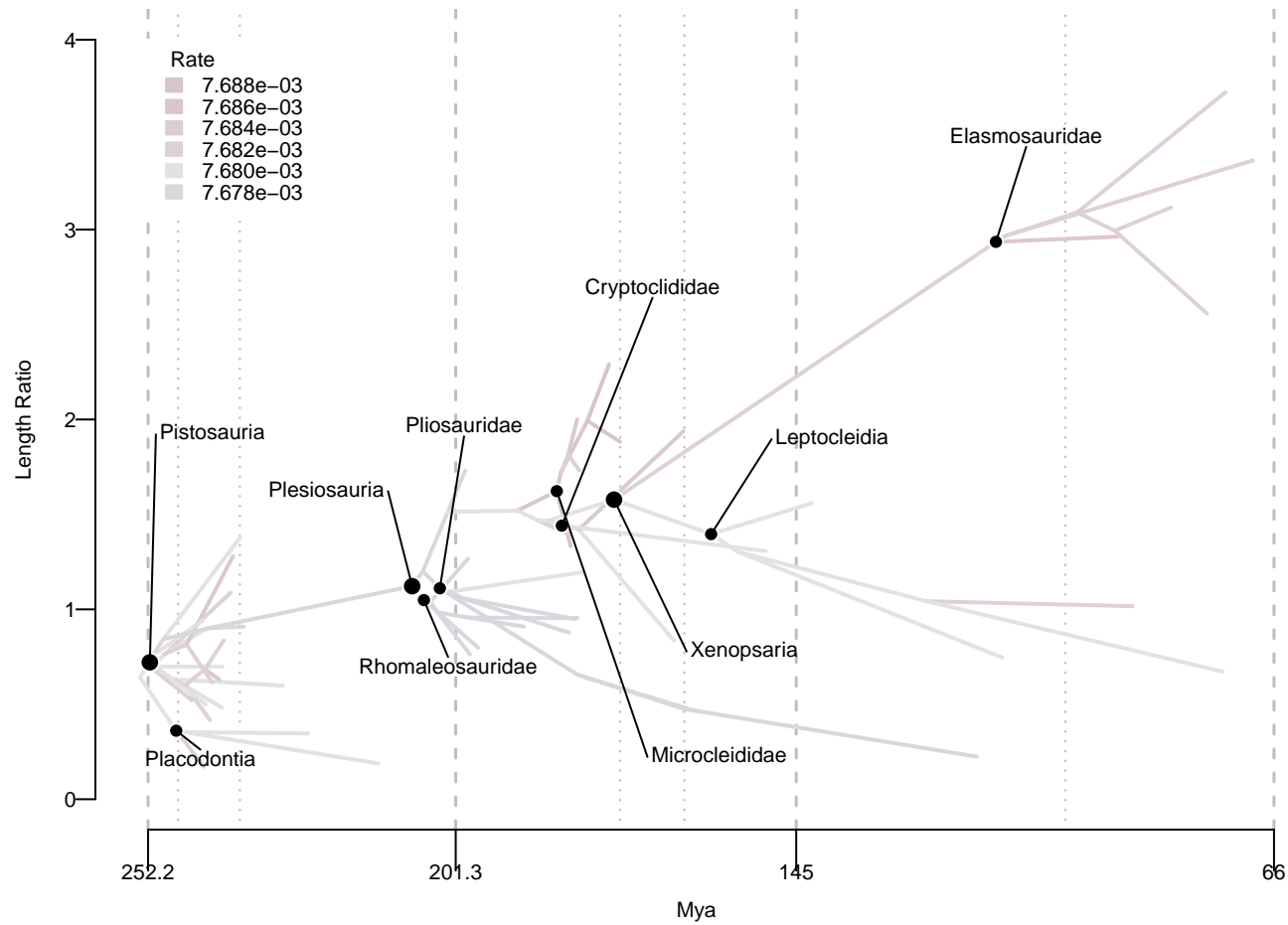

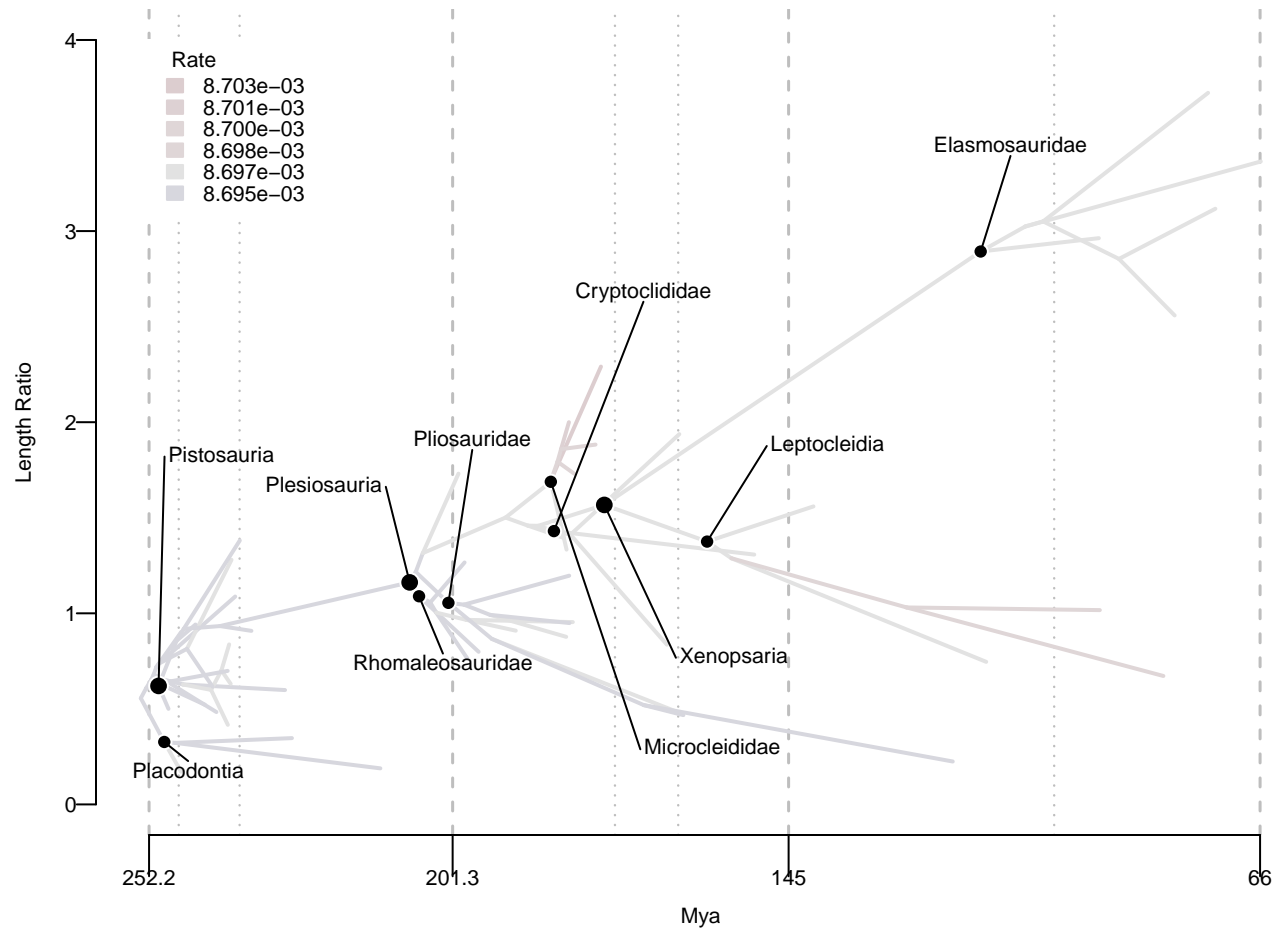

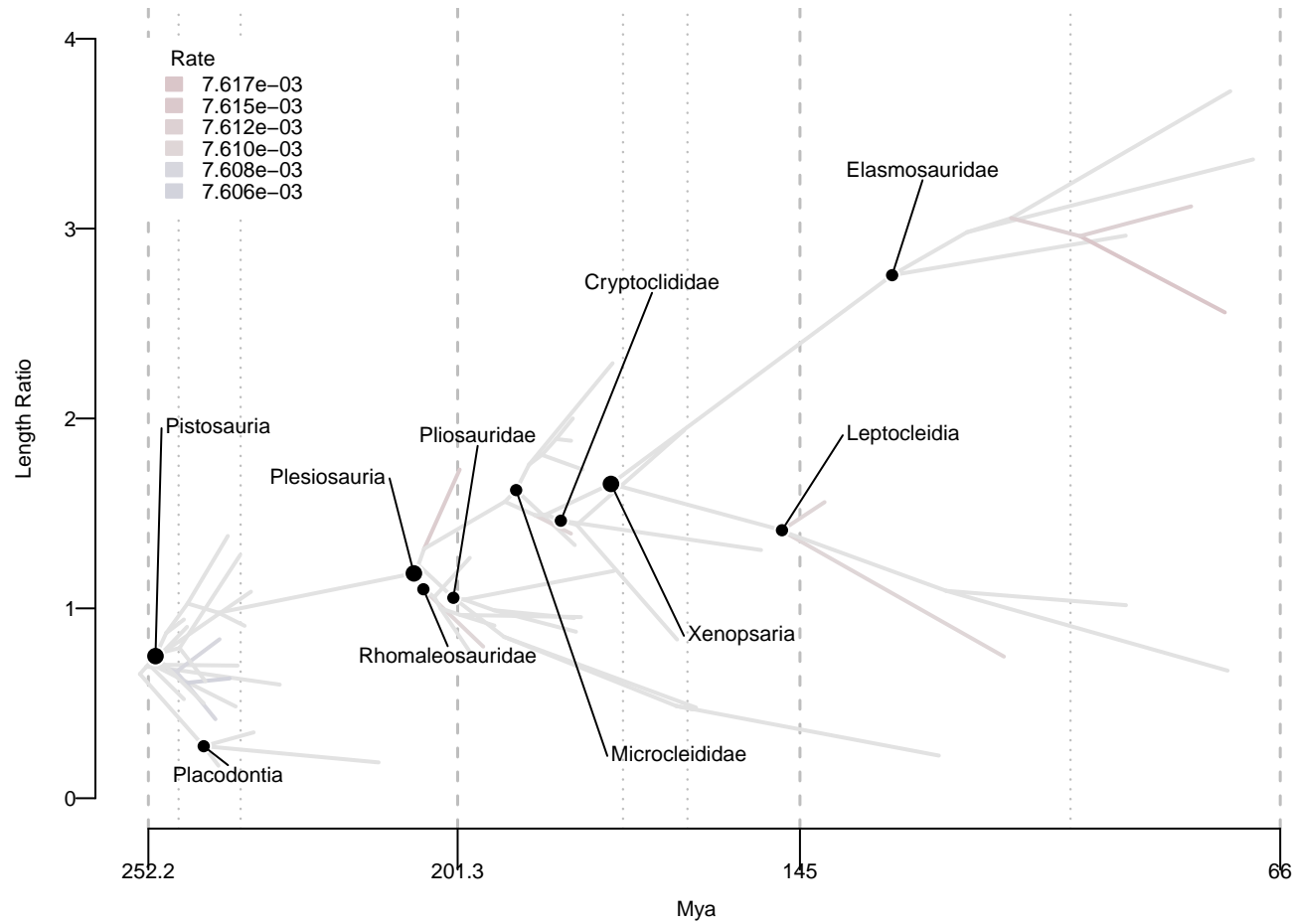

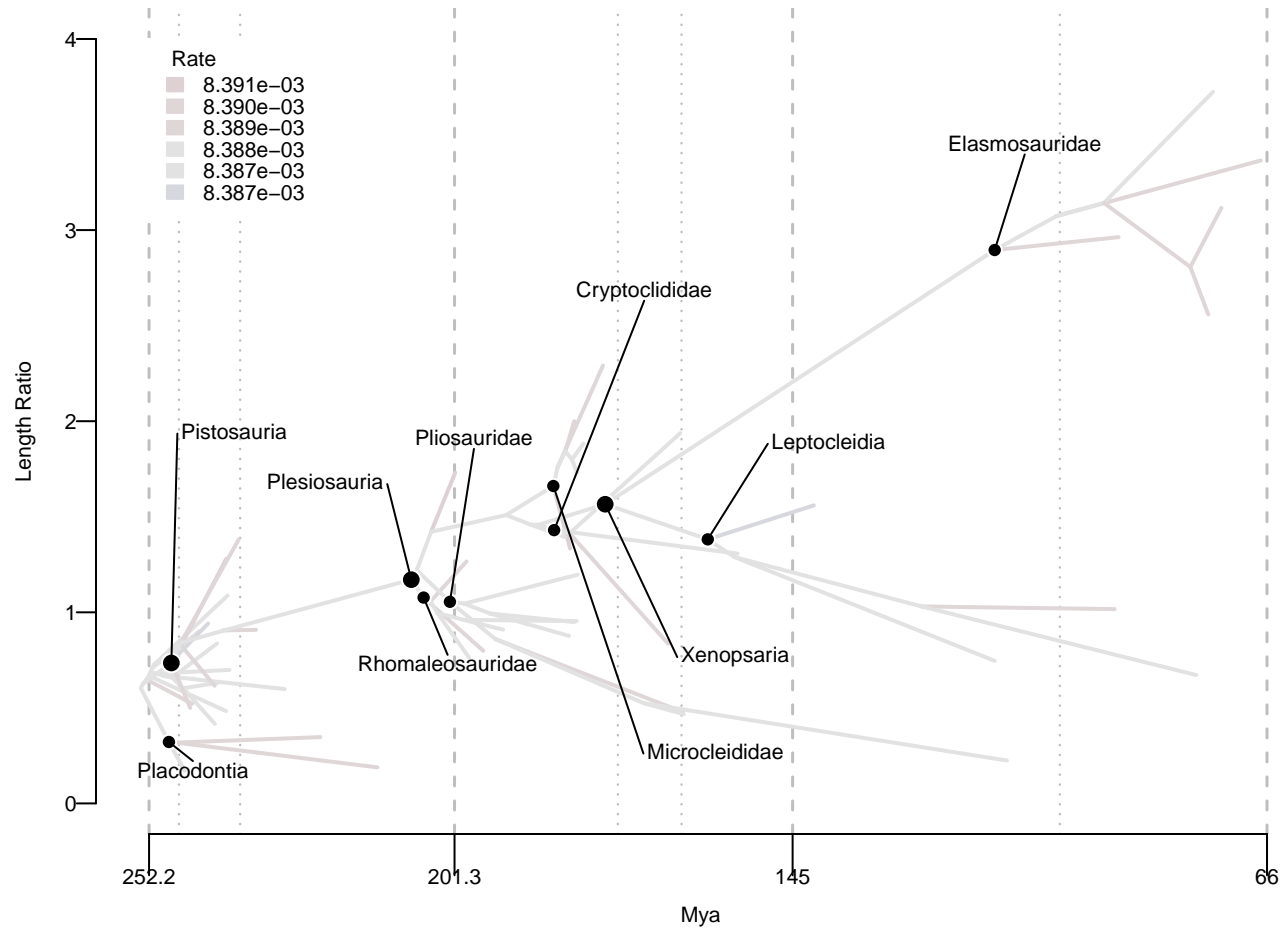

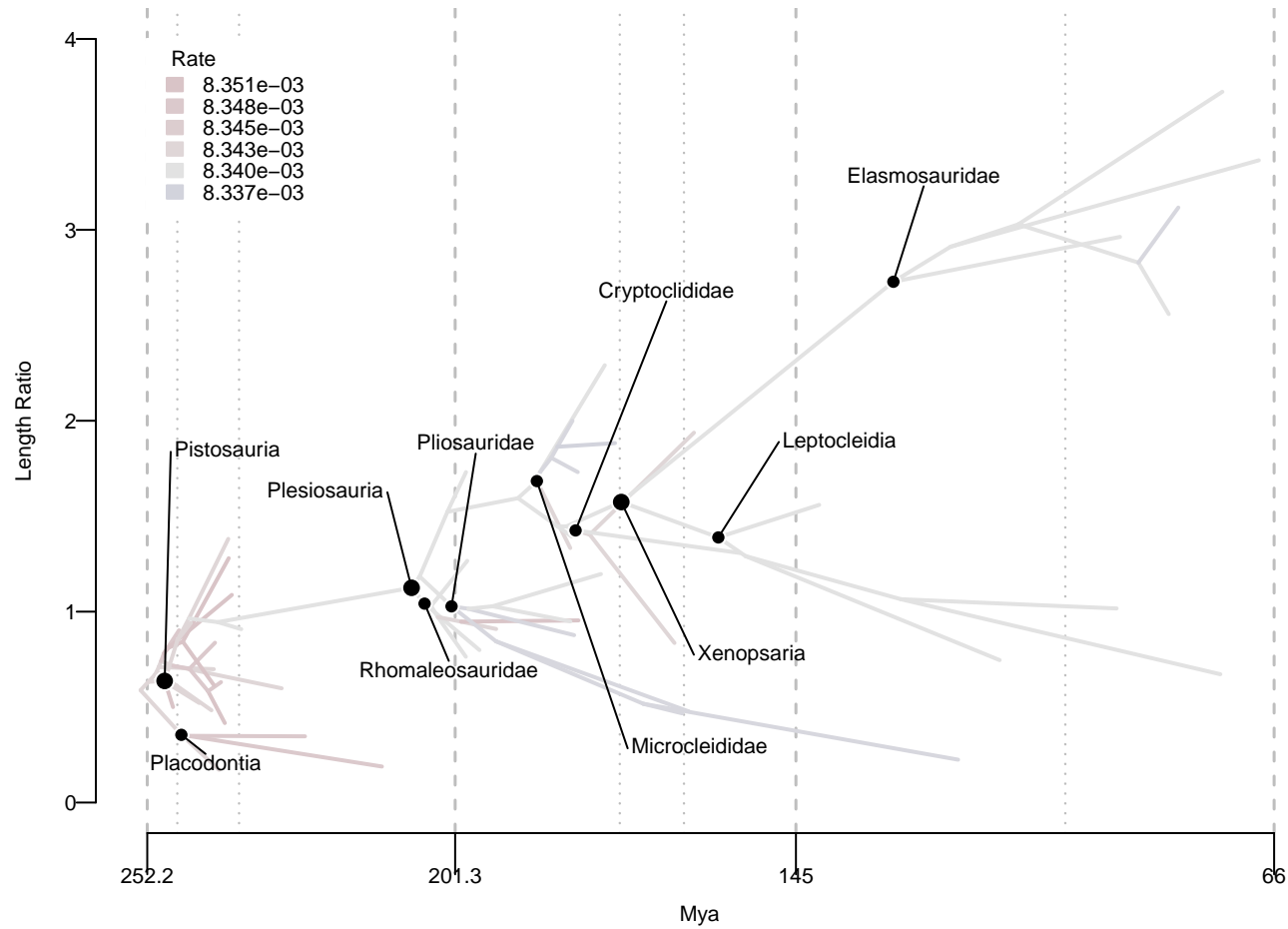

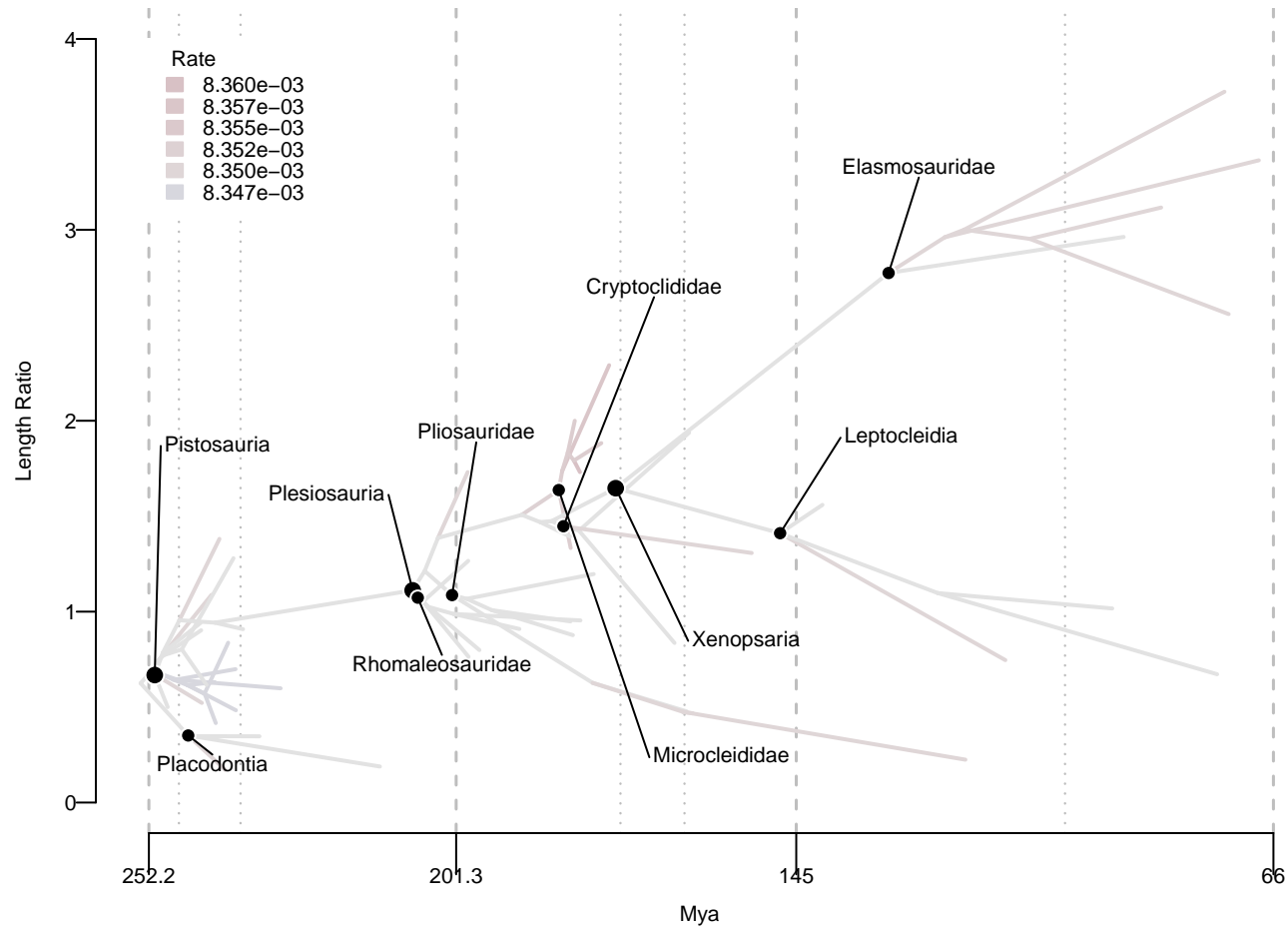

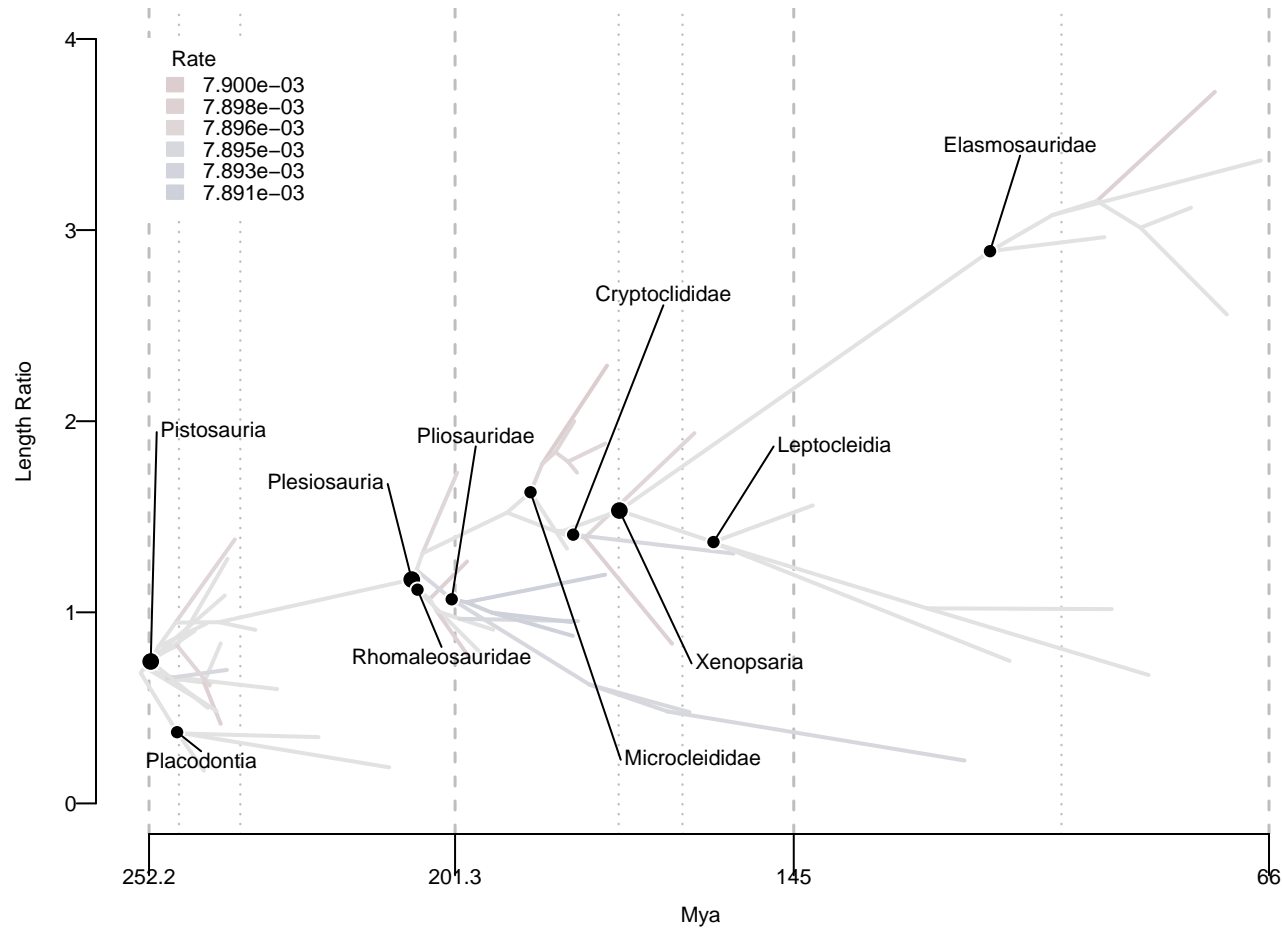

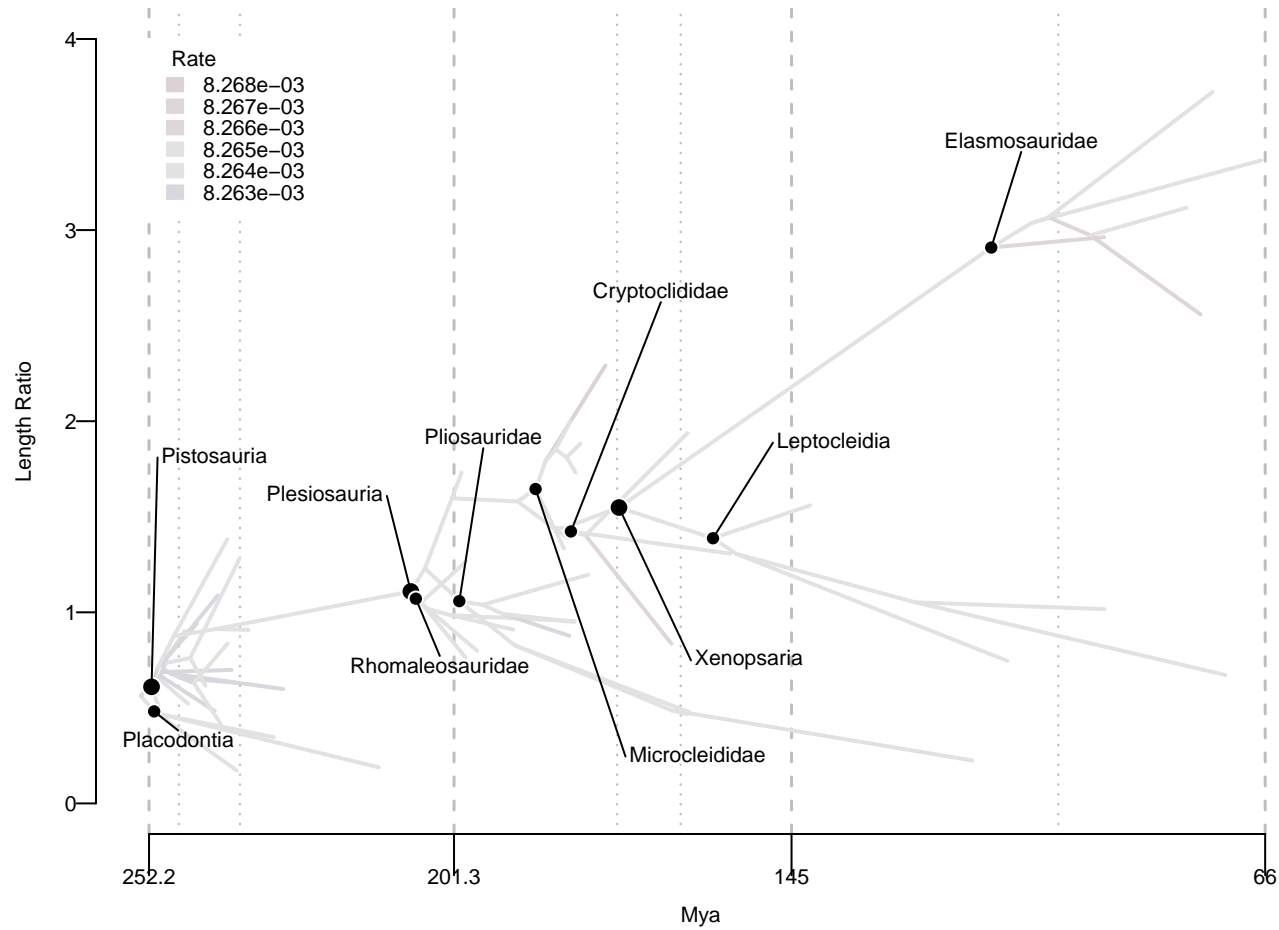

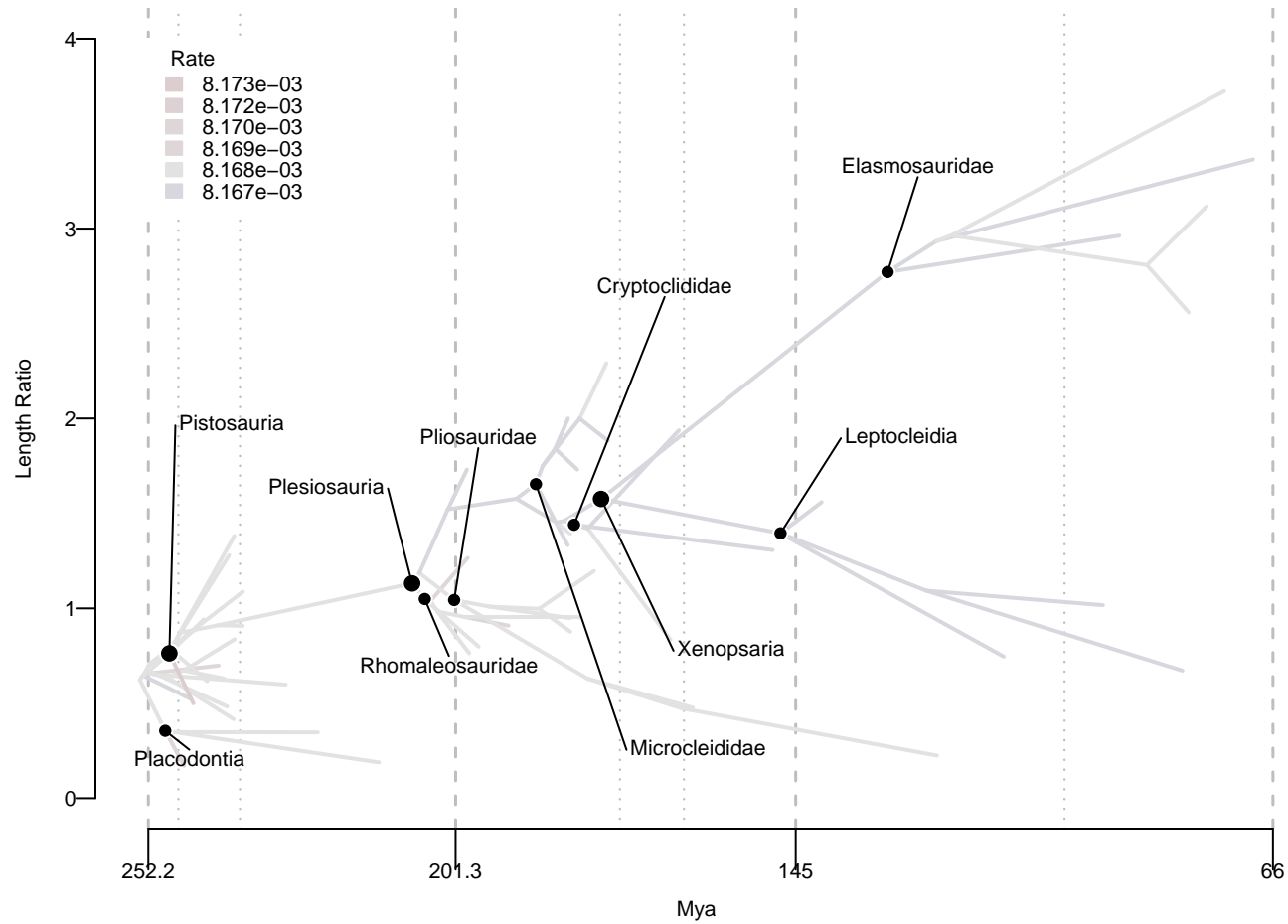

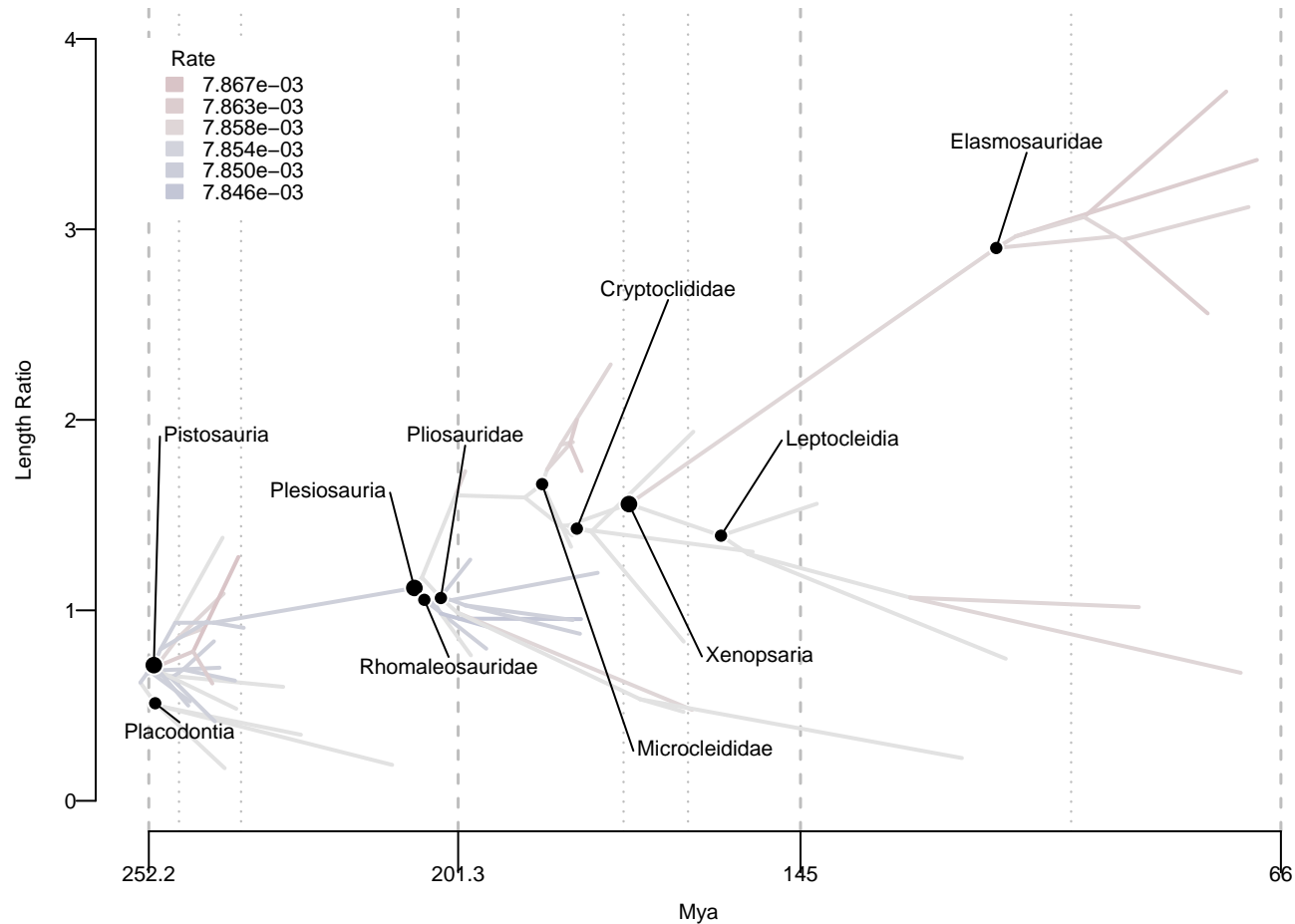

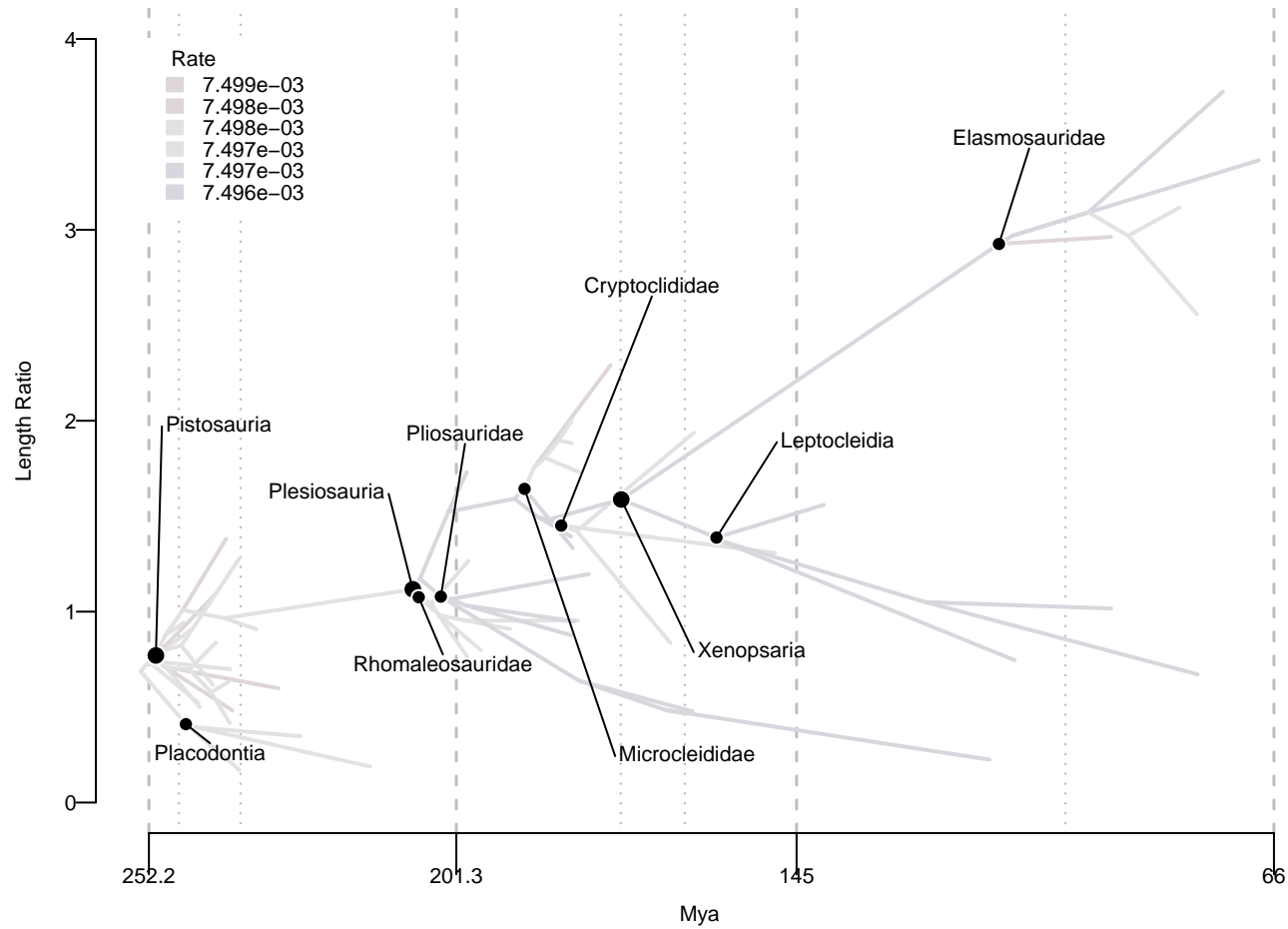

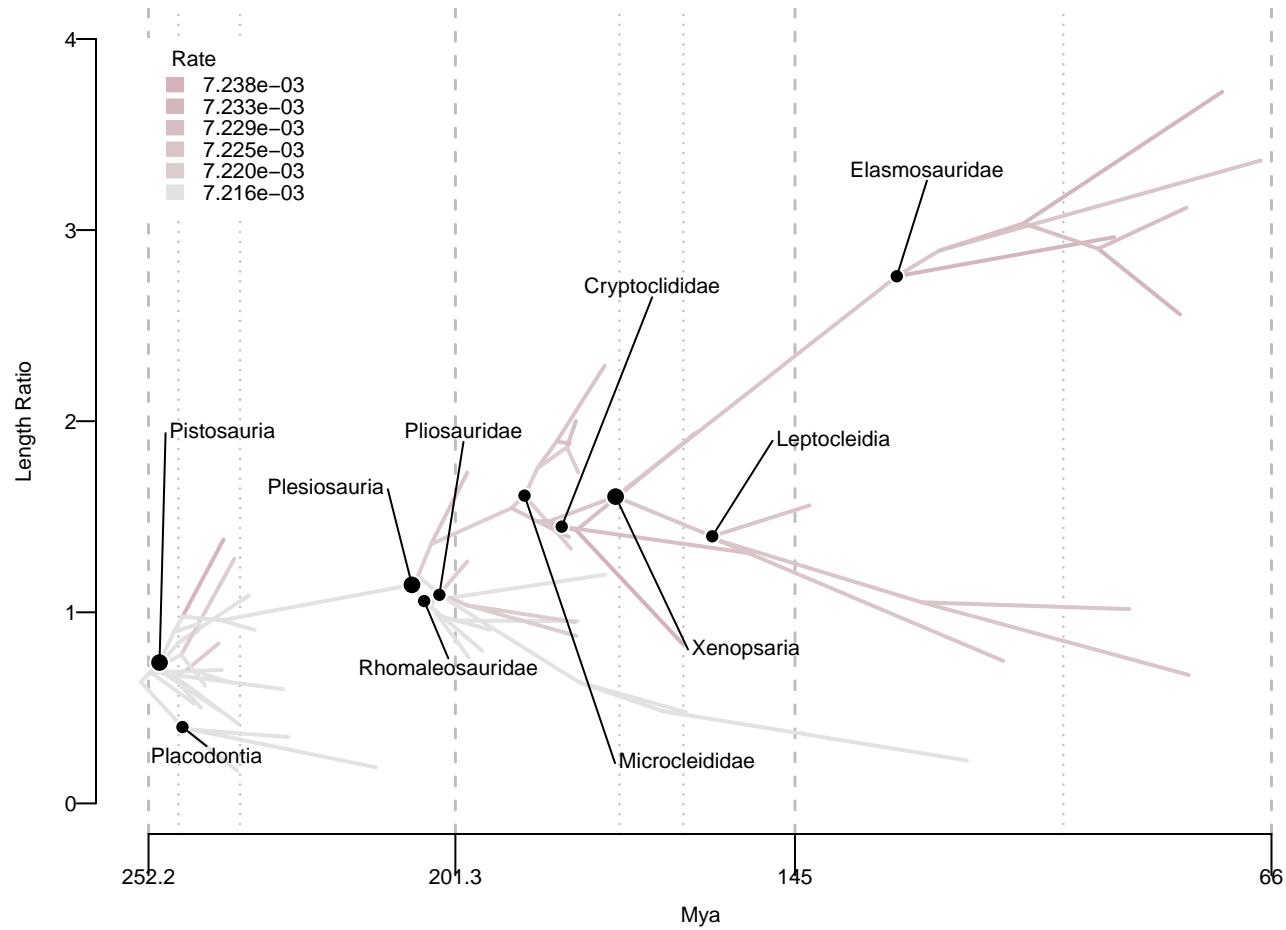

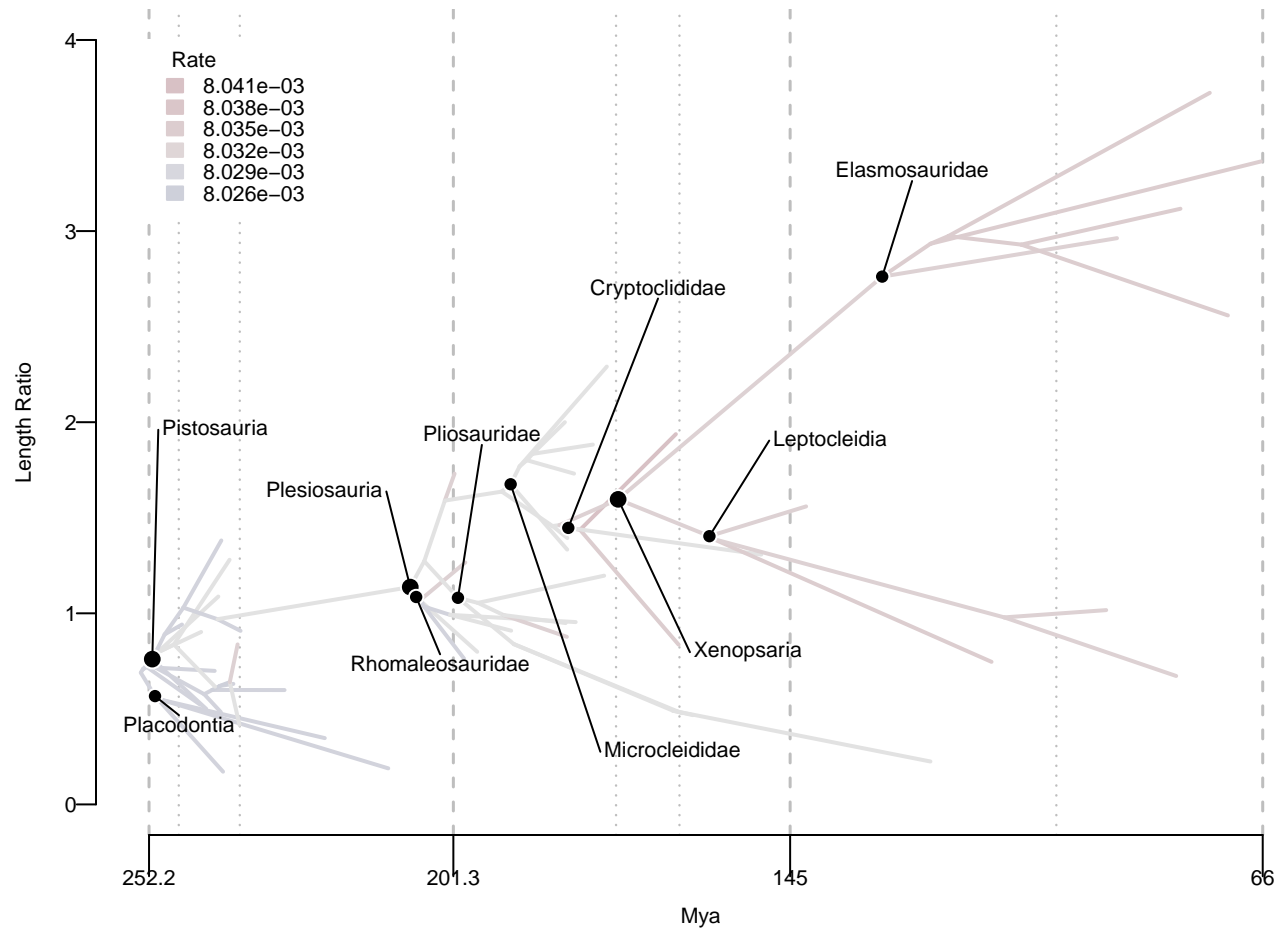

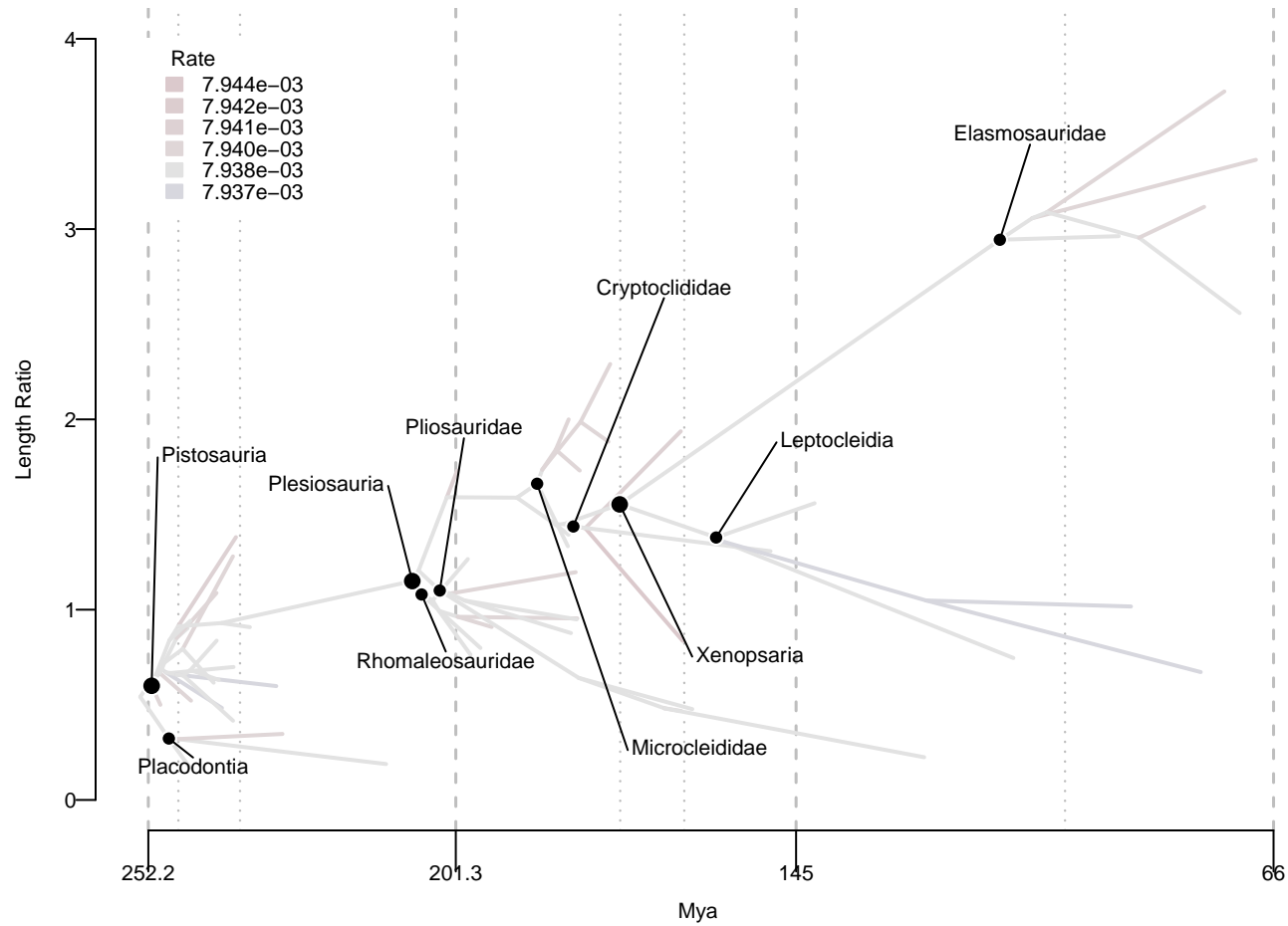

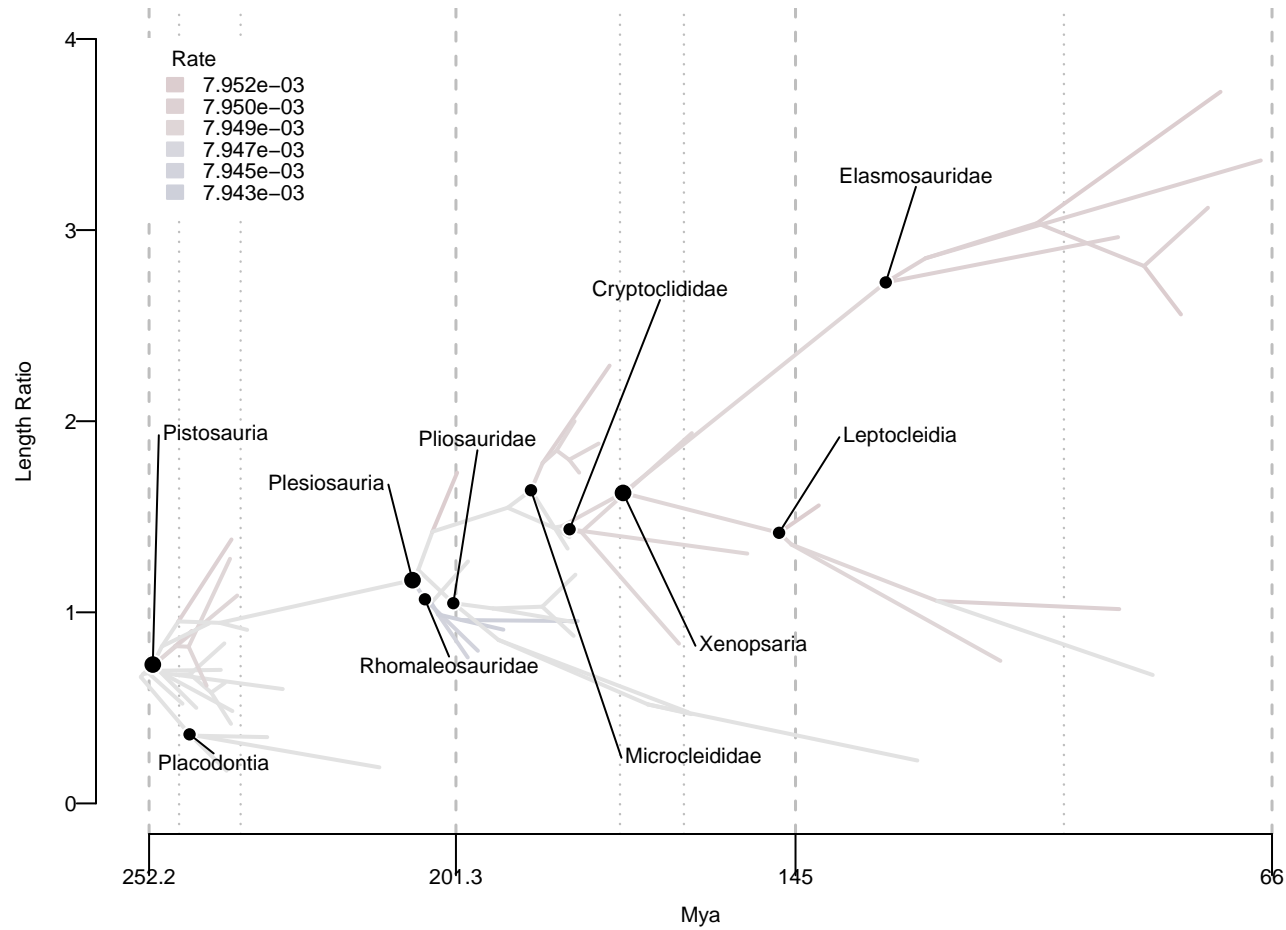

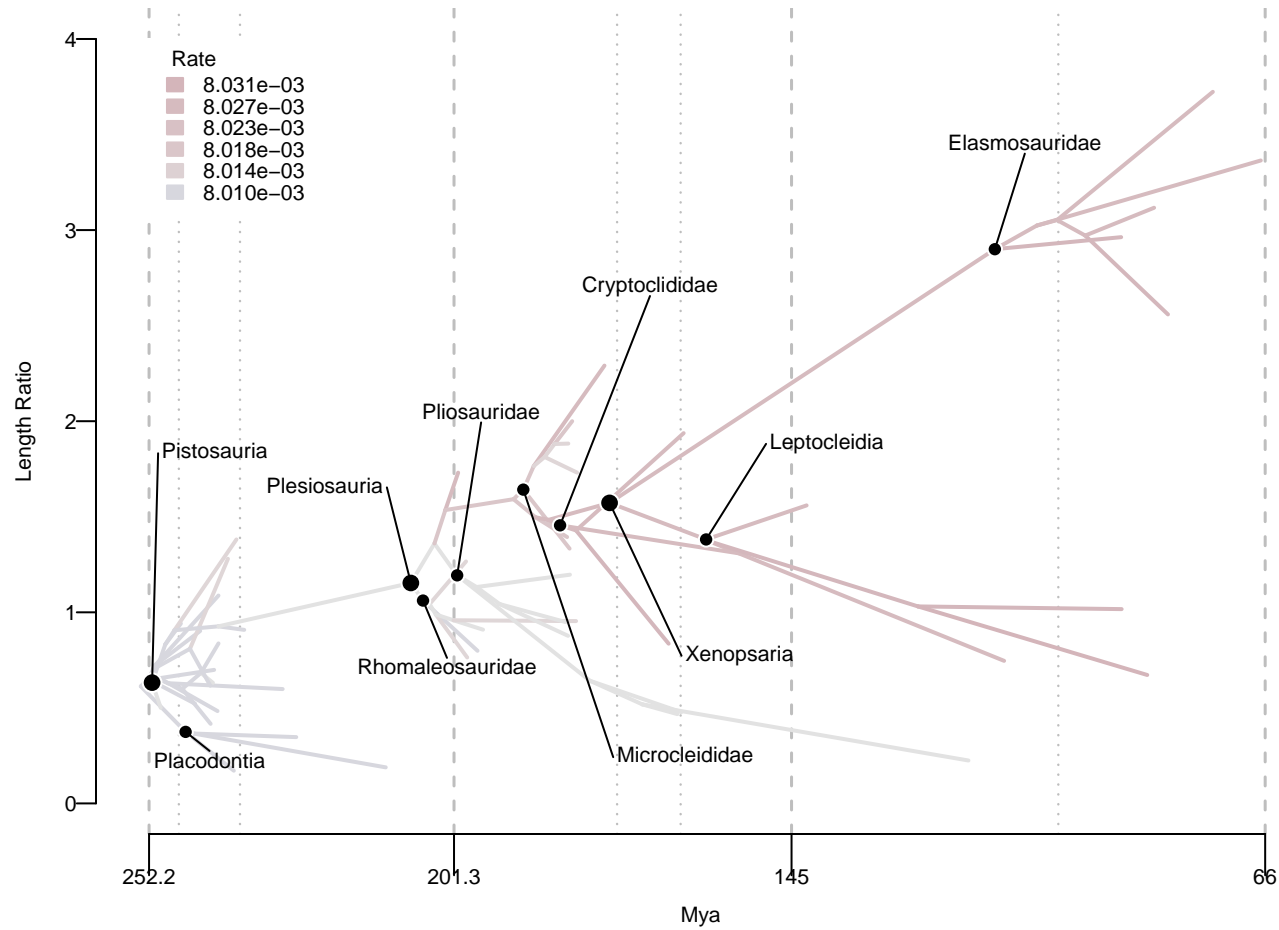

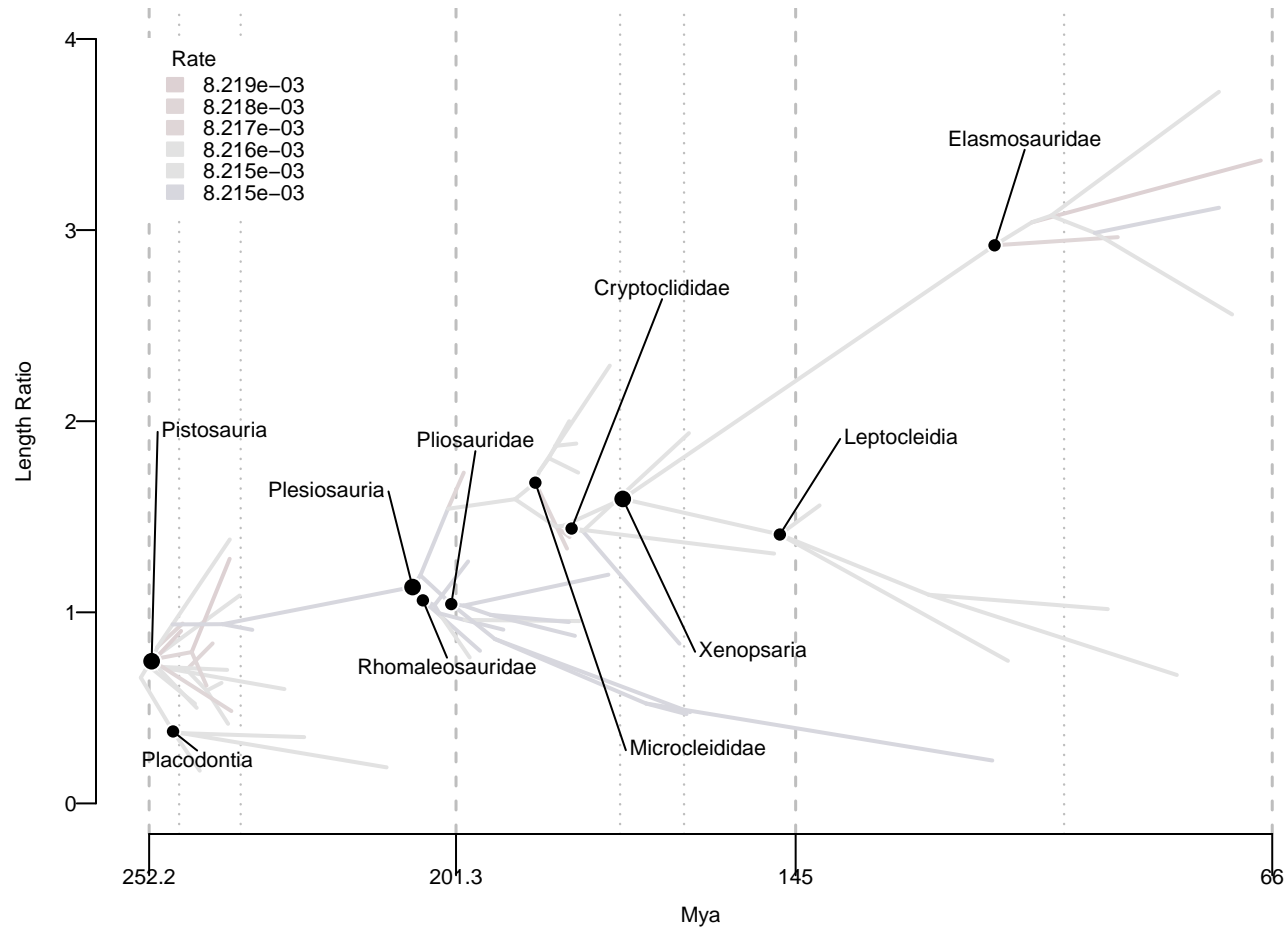

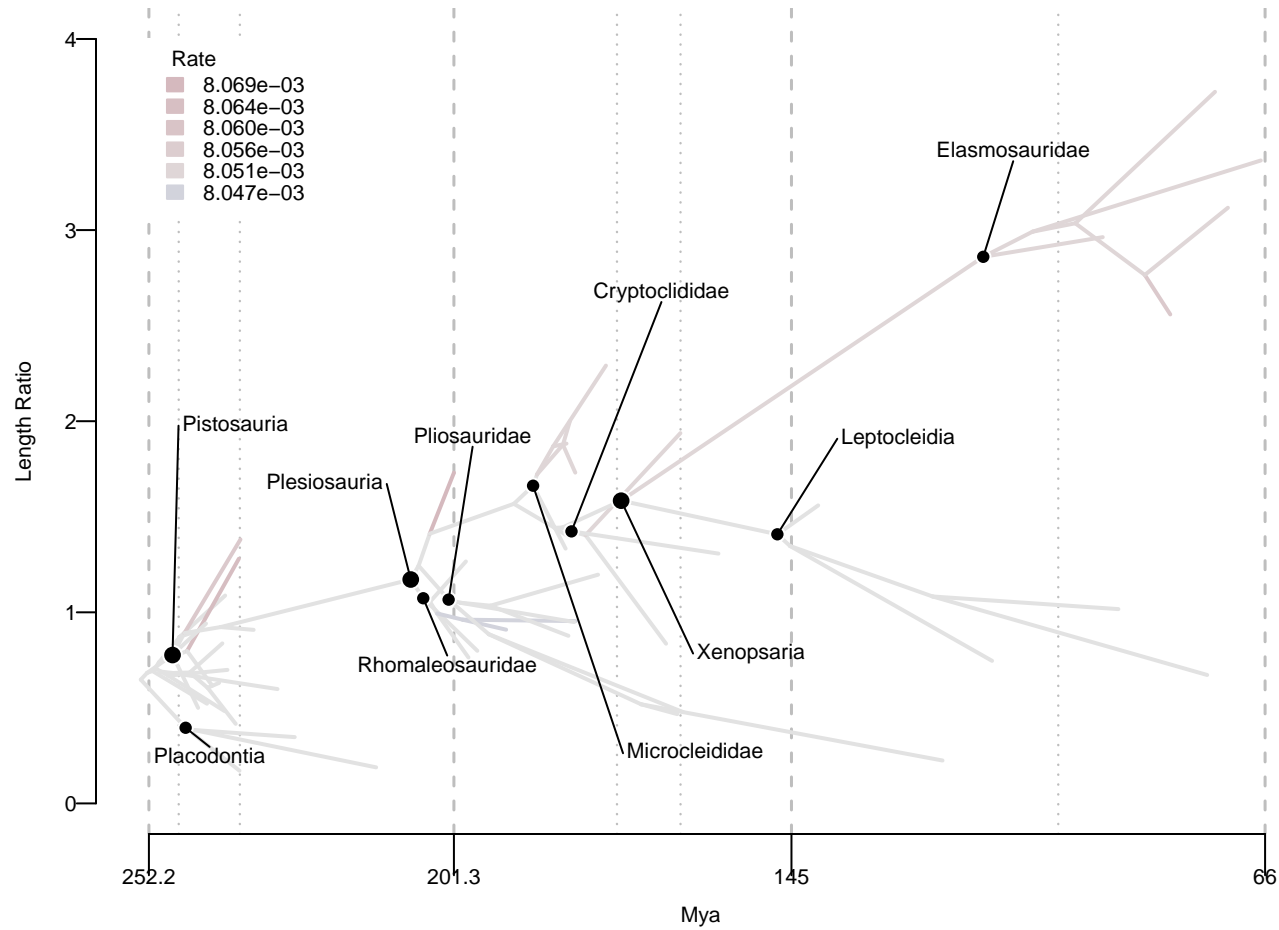

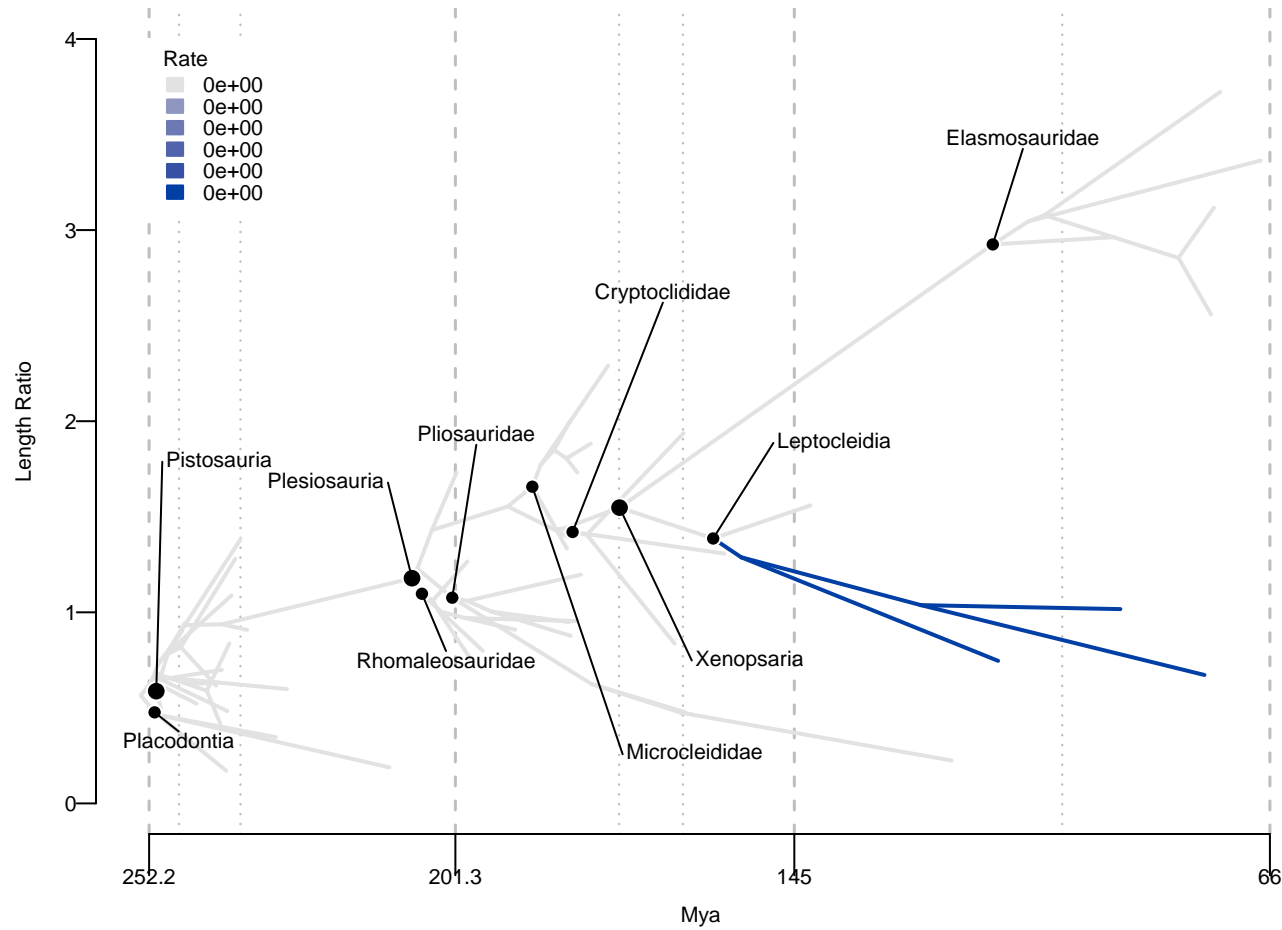

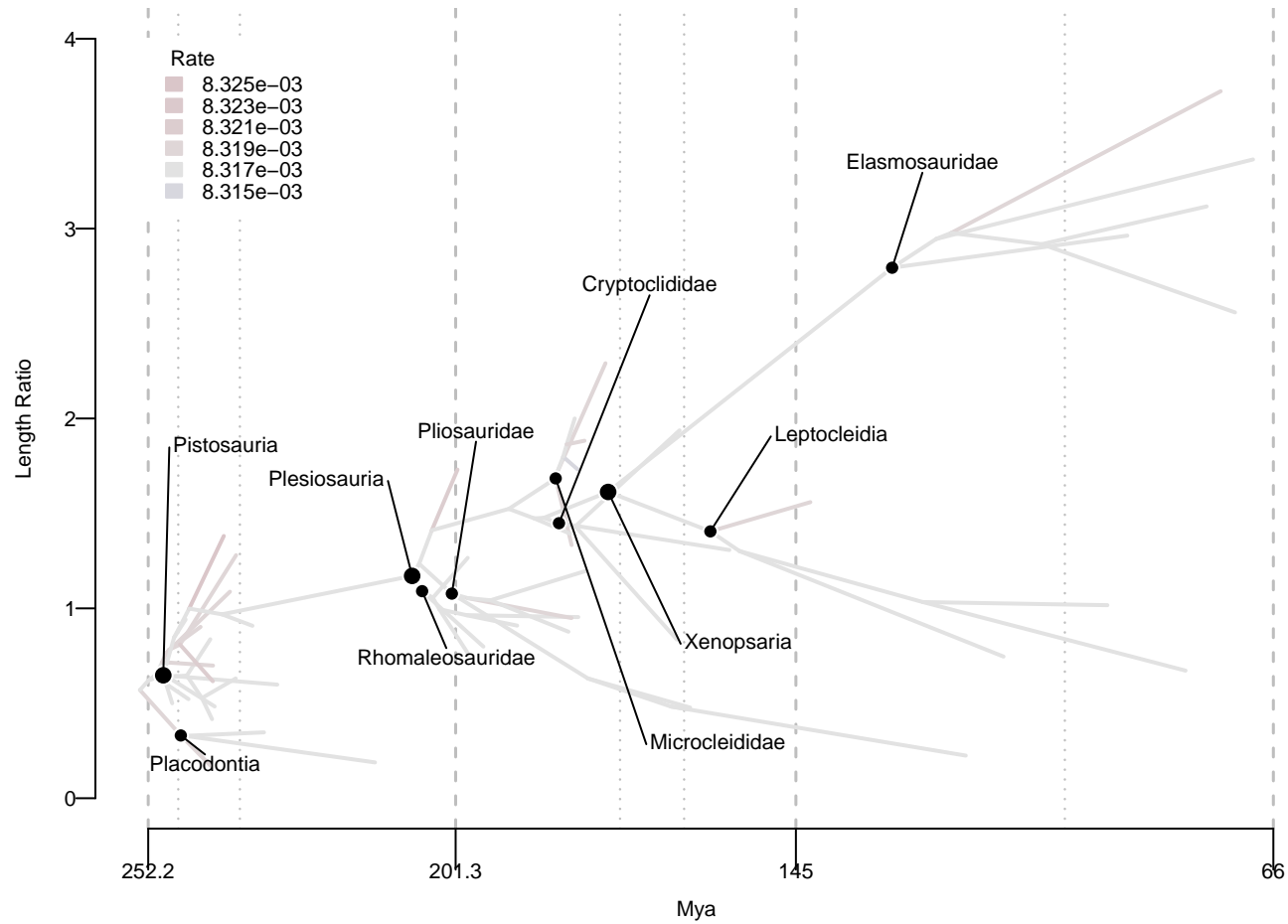

Supplement: Supplementary file 8 — Figure S8. [file EVO-71-1164-s008.pdf]
