# Supplementary material for: Developmental mechanisms of macroevolutionary change in the tetrapod axis: A case study of Sauropterygia
Source: Evolution. 2017 Mar 21;71(5):1164–77. doi: 10.1111/evo.13217 (PMC5485078; doi:10.1111/evo.13217)
Supplement: Supplementary file 6 — Figure S6. [file EVO-71-1164-s006.pdf]

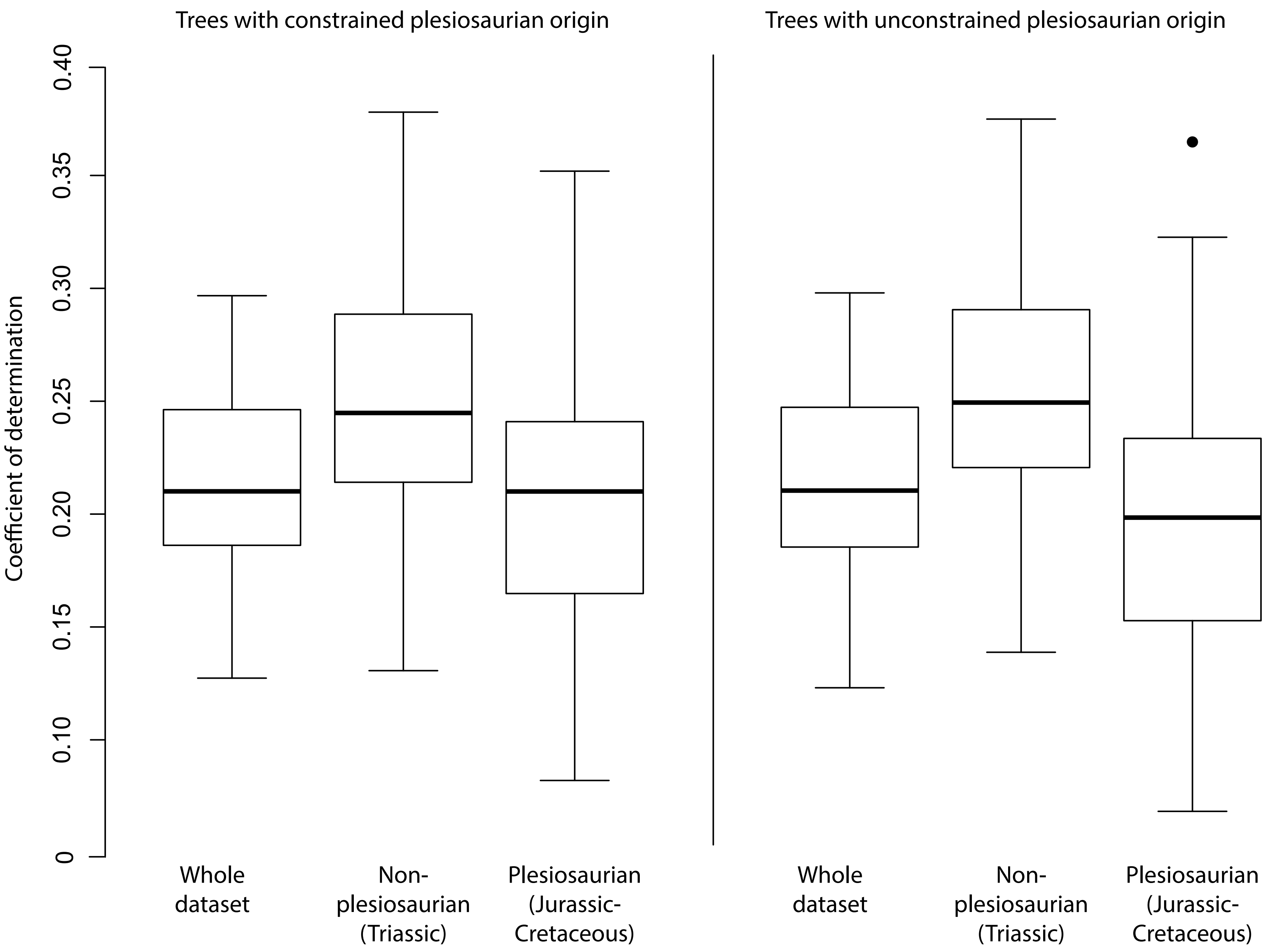
Trees with constrained plesiosaurian origin
Trees with unconstrained plesiosaurian origin
Coefficient of determination
0.40
0.35
0.30
0.25
0.20
0.15
0.10
0
Whole dataset
Non-plesiosaurian (Triassic)
Plesiosaurian (Jurassic-Cretaceous)
Whole dataset
Non-plesiosaurian (Triassic)
Plesiosaurian (Jurassic-Cretaceous)
