# Supplementary material for: Developmental mechanisms of macroevolutionary change in the tetrapod axis: A case study of Sauropterygia
Source: Evolution. 2017 Mar 21;71(5):1164–77. doi: 10.1111/evo.13217 (PMC5485078; doi:10.1111/evo.13217)
Supplement: Supplementary file 9 — Figure S9. [file EVO-71-1164-s009.pdf]

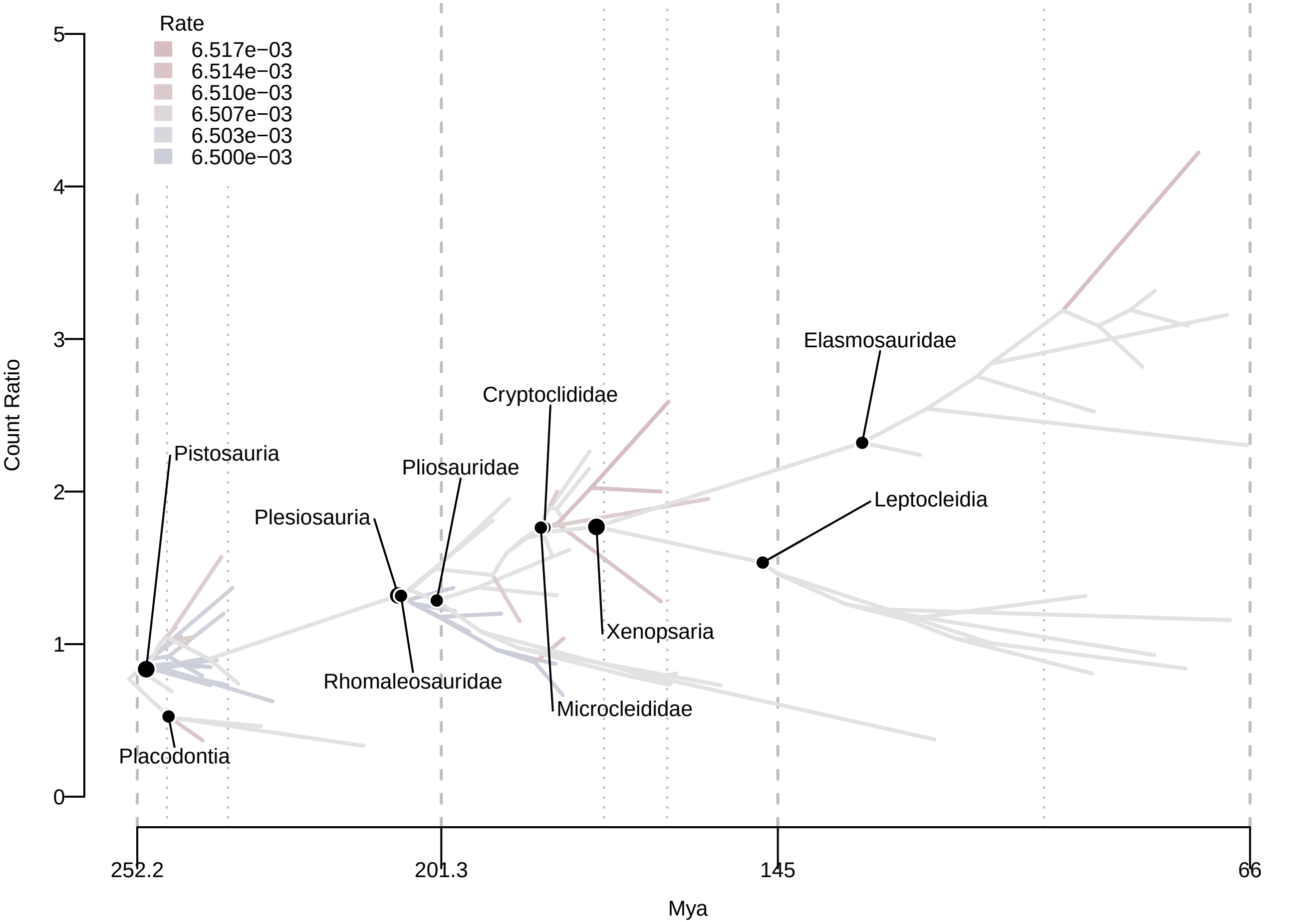

Rate
6.517e−03
6.514e−03
6.510e−03
6.507e−03
6.503e−03
6.500e−03
Count Ratio
5
4
3
2
1
0
Elasmosauridae
Cryptoclididae
Pistosauria
Pliosauridae
Leptocleidia
Plesiosauria
Xenopsaria
Rhomaleosauridae
Microcleididae
Placodontia
252.2
201.3
145
66
Mya

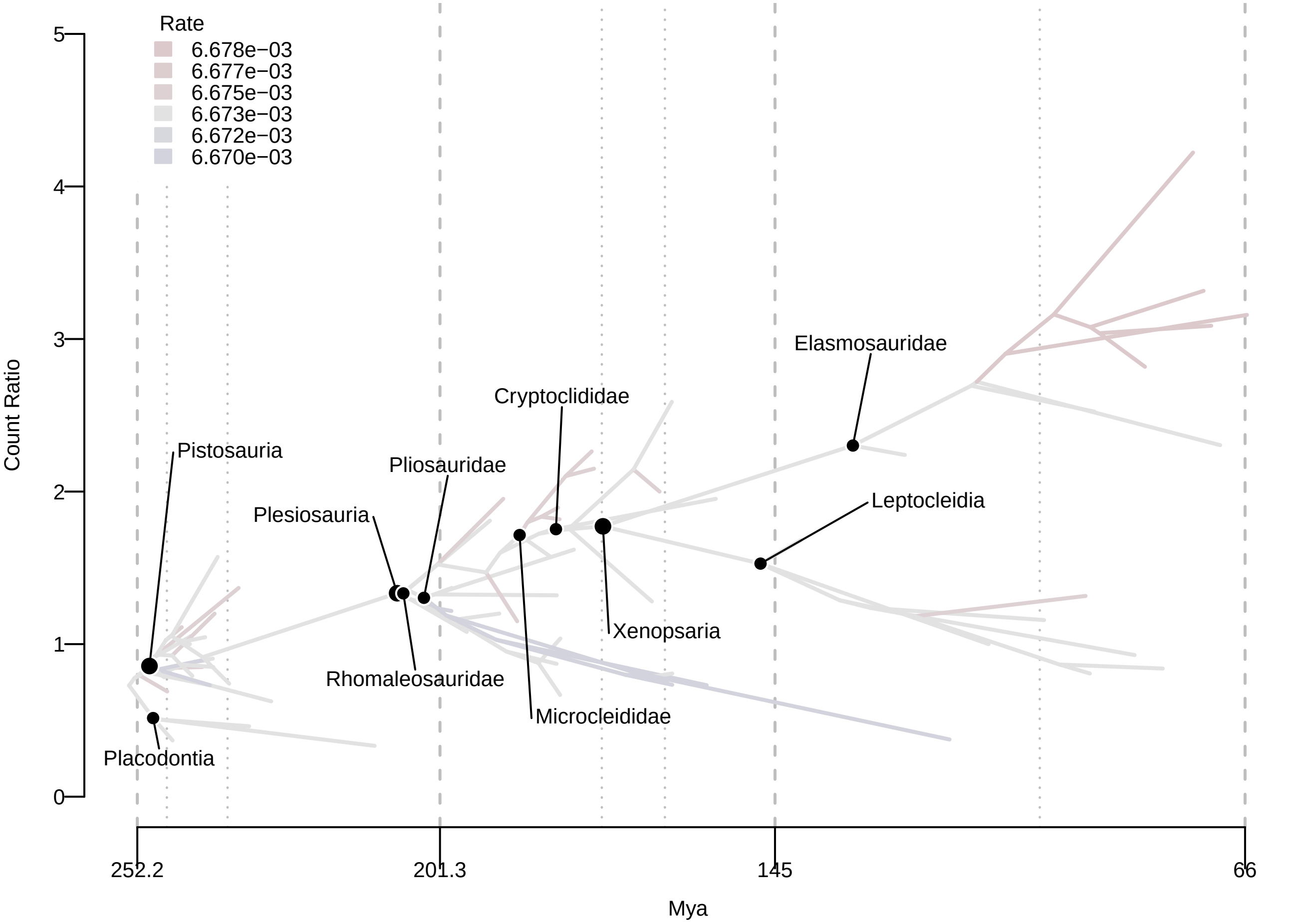

Rate
6.678e−03
6.677e−03
6.675e−03
6.673e−03
6.672e−03
6.670e−03
Count Ratio
5
4
3
2
1
0
Pistosauria
Plesiosauria
Pliosauridae
Rhomaleosauridae
Placodontia
Cryptoclididae
Microcleididae
Xenopsaria
Elasmosauridae
Leptocleidia
252.2
201.3
145
66
Mya

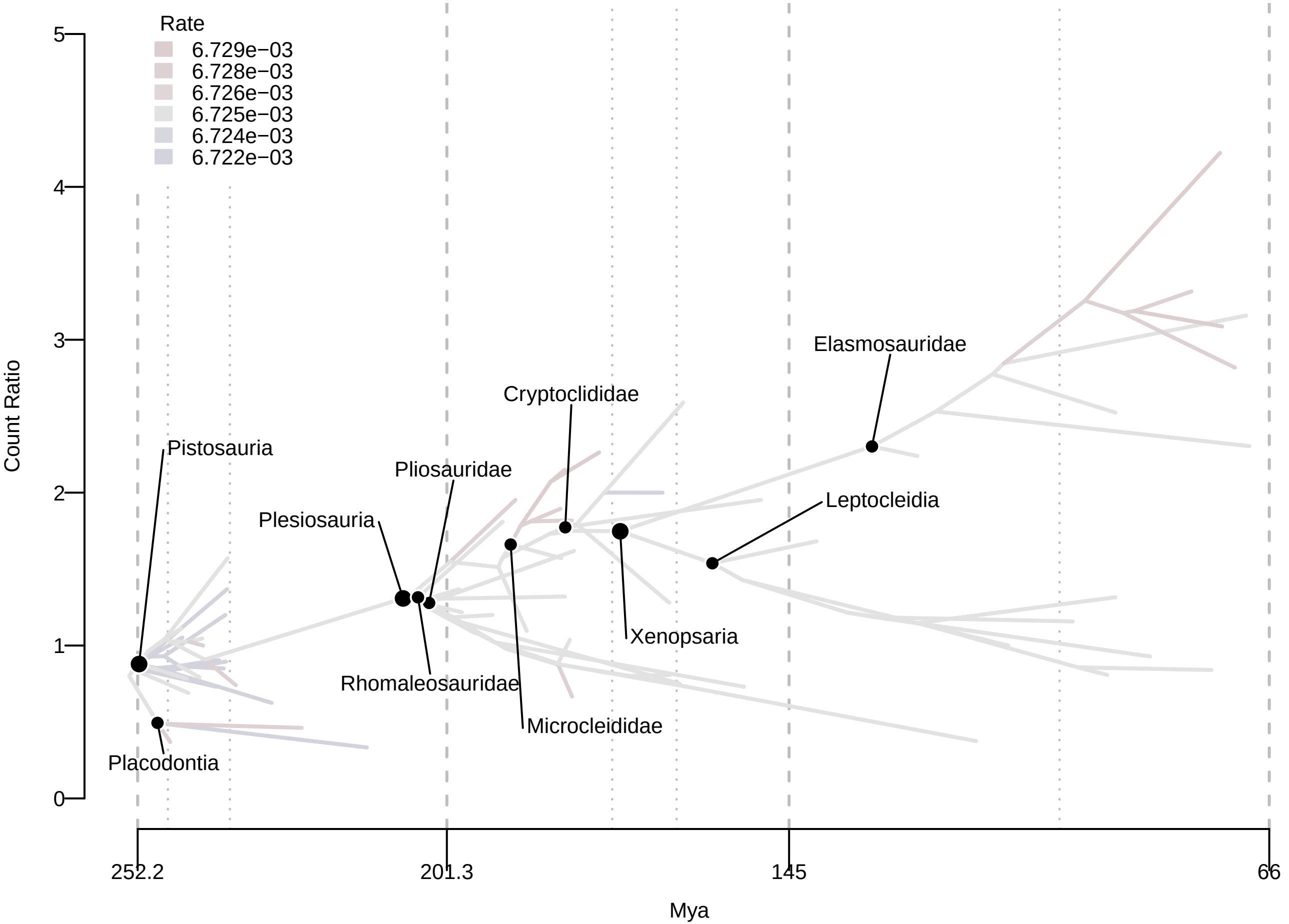
Rate
6.729e−03
6.728e−03
6.726e−03
6.725e−03
6.724e−03
6.722e−03
Count Ratio
0
1
2
3
4
5
Pistosauria
Placodontia
Plesiosauria
Pliosauridae
Rhomaleosauridae
Microcleididae
Cryptoclididae
Xenopsaria
Leptocleidia
Elasmosauridae
252.2
201.3
145
66
Mya

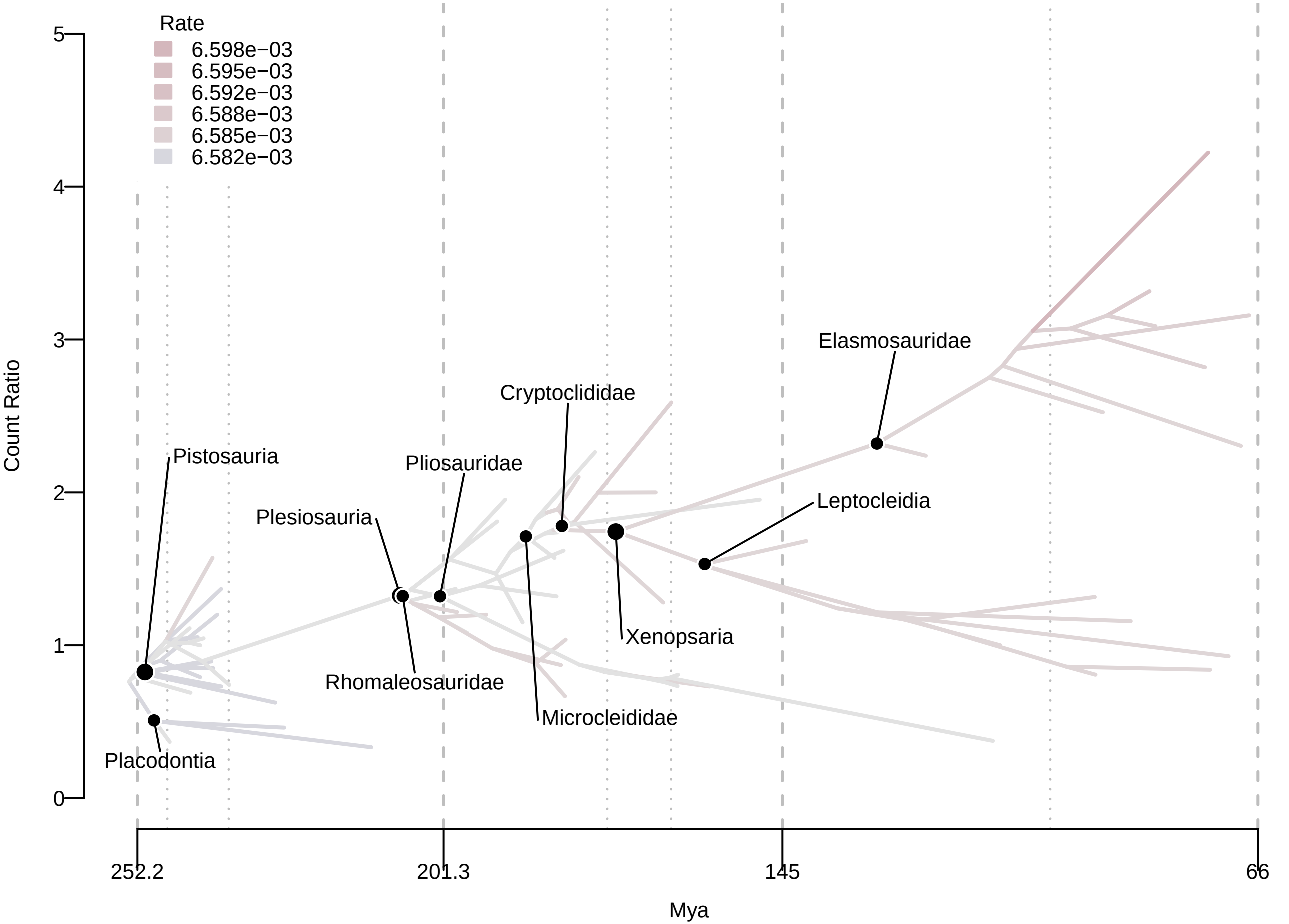

Rate
6.598e−03
6.595e−03
6.592e−03
6.588e−03
6.585e−03
6.582e−03
Count Ratio
0
1
2
3
4
5
Pistosauria
Plesiosauria
Pliosauridae
Cryptoclididae
Elasmosauridae
Leptocleidia
Xenopsaria
Microcleididae
Rhomaleosauridae
Placodontia
252.2
201.3
145
66
Mya

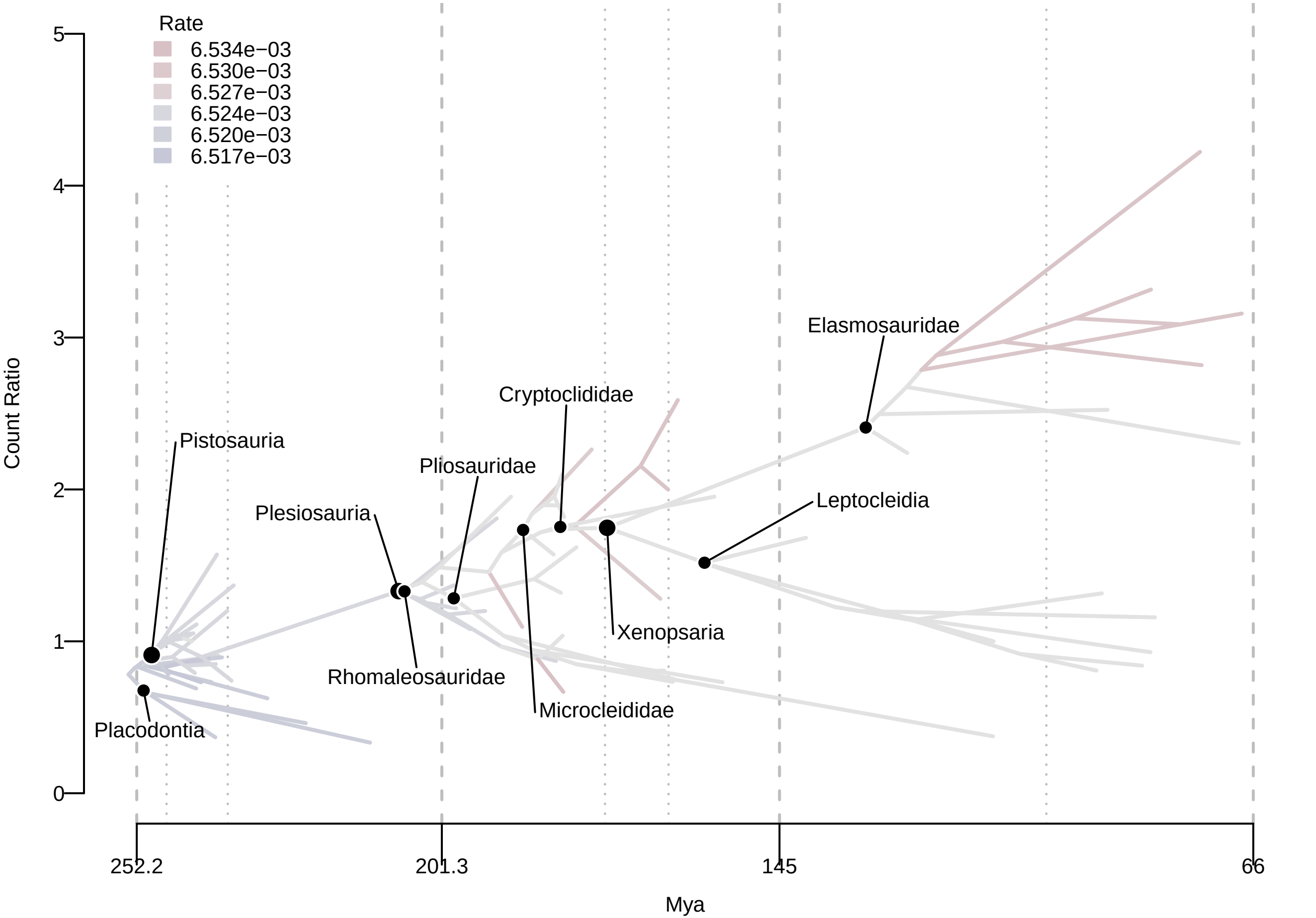

Rate
6.534e−03
6.530e−03
6.527e−03
6.524e−03
6.520e−03
6.517e−03
Count Ratio
5
4
3
2
1
0
Pistosauria
Placodontia
Plesiosauria
Rhomaleosauridae
Pliosauridae
Cryptoclididae
Microcleididae
Xenopsaria
Leptocleidia
Elasmosauridae
252.2
201.3
145
66
Mya

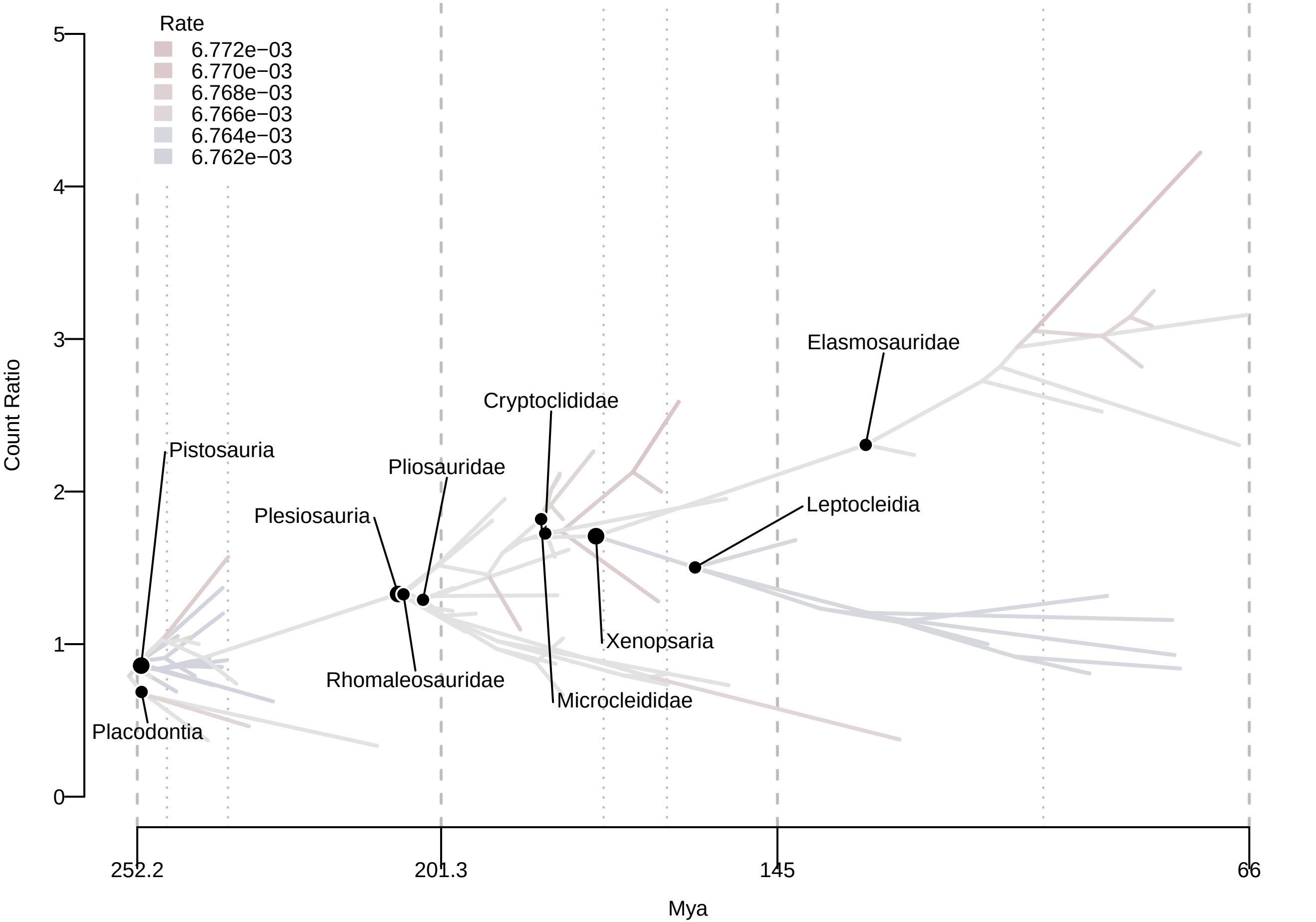

Rate
6.772e−03
6.770e−03
6.768e−03
6.766e−03
6.764e−03
6.762e−03
Count Ratio
0
1
2
3
4
5
Pistosauria
Placodontia
Plesiosauria
Pliosauridae
Rhomaleosauridae
Cryptoclididae
Microcleididae
Xenopsaria
Leptocleidia
Elasmosauridae
252.2
201.3
145
66
Mya

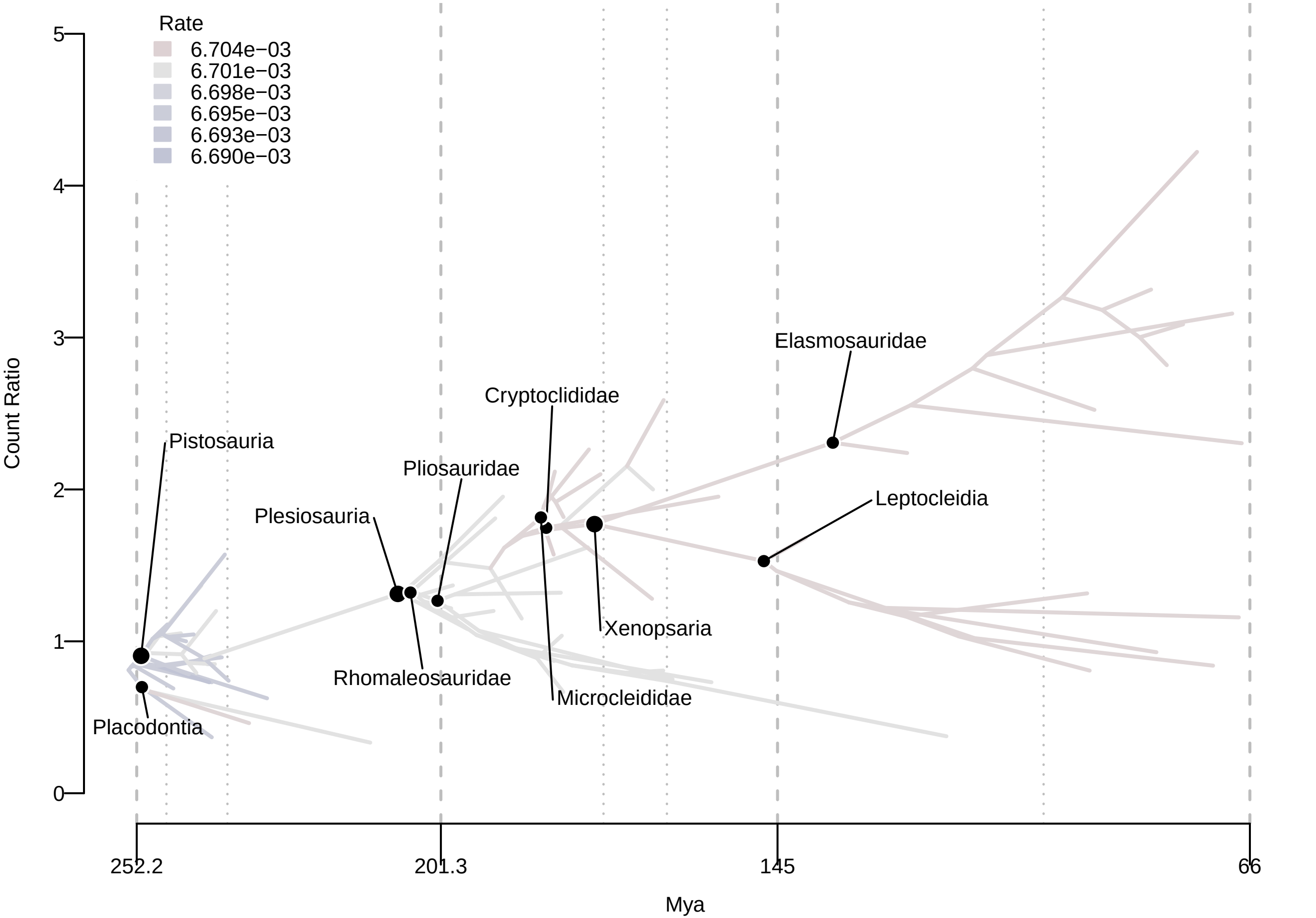

Rate
6.704e−03
6.701e−03
6.698e−03
6.695e−03
6.693e−03
6.690e−03
Count Ratio
0
1
2
3
4
5
Pistosauria
Placodontia
Plesiosauria
Rhomaleosauridae
Pliosauridae
Cryptoclididae
Microcleididae
Xenopsaria
Elasmosauridae
Leptocleidia
252.2
201.3
145
66
Mya

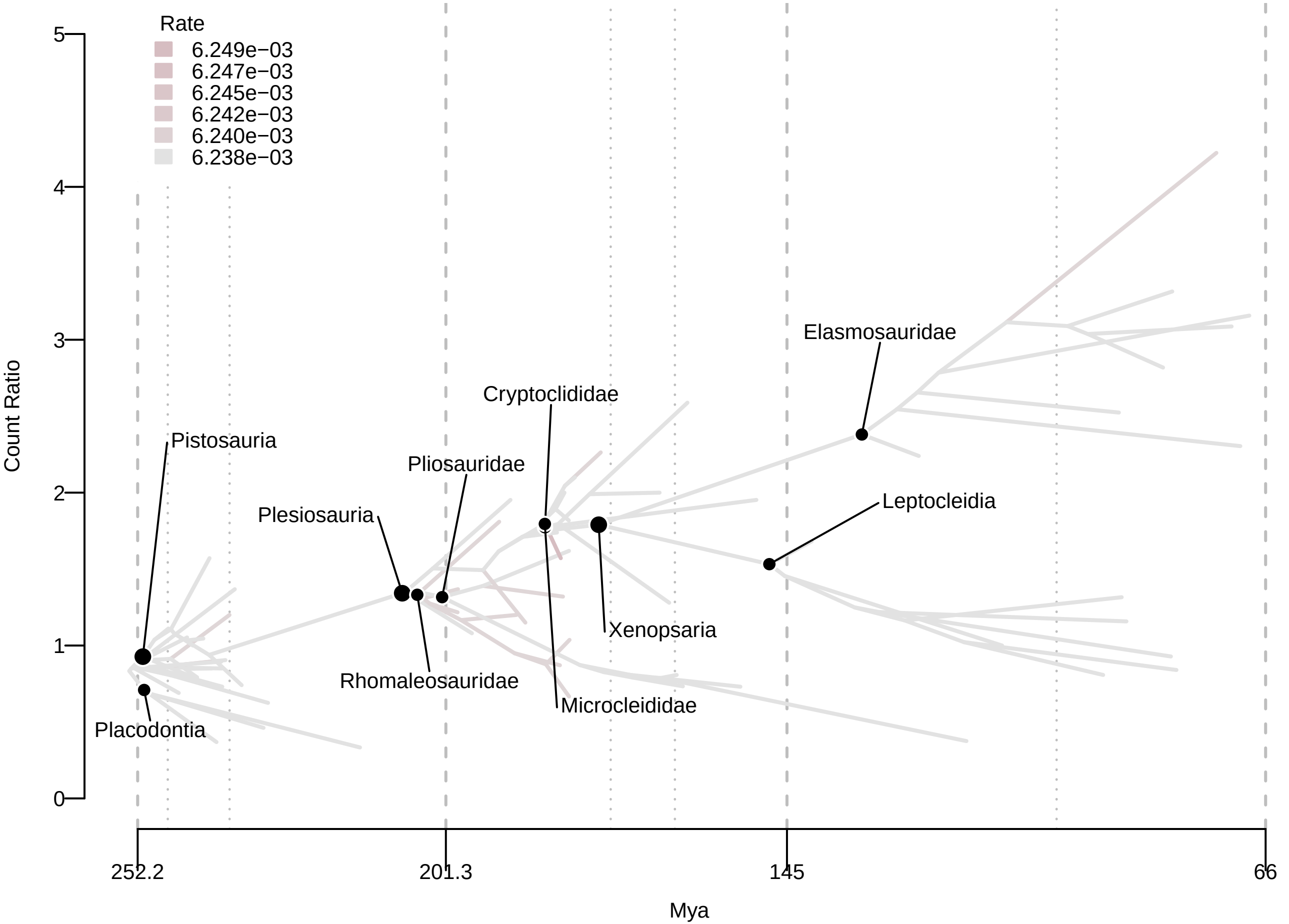

Rate
6.249e−03
6.247e−03
6.245e−03
6.242e−03
6.240e−03
6.238e−03
Count Ratio
0
1
2
3
4
5
Pistosauria
Placodontia
Plesiosauria
Pliosauridae
Rhomaleosauridae
Cryptoclididae
Microcleididae
Xenopsaria
Elasmosauridae
Leptocleidia
252.2
201.3
145
66
Mya

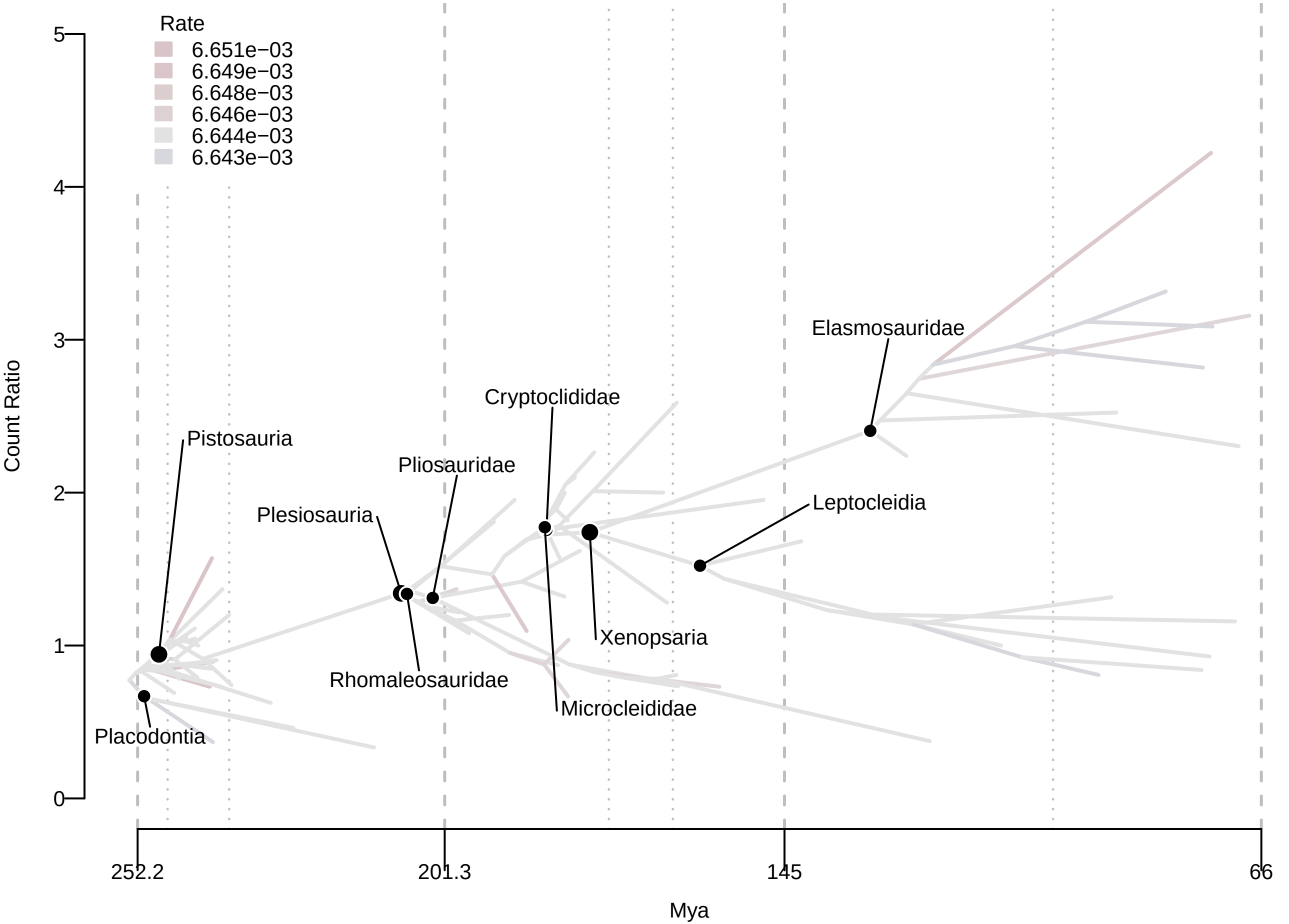
Rate
6.651e−03
6.649e−03
6.648e−03
6.646e−03
6.644e−03
6.643e−03
Count Ratio
0
1
2
3
4
5
Mya
252.2
201.3
145
66
Pistosauria
Placodontia
Plesiosauria
Pliosauridae
Rhomaleosauridae
Cryptoclididae
Microcleididae
Xenopsaria
Leptocleidia
Elasmosauridae

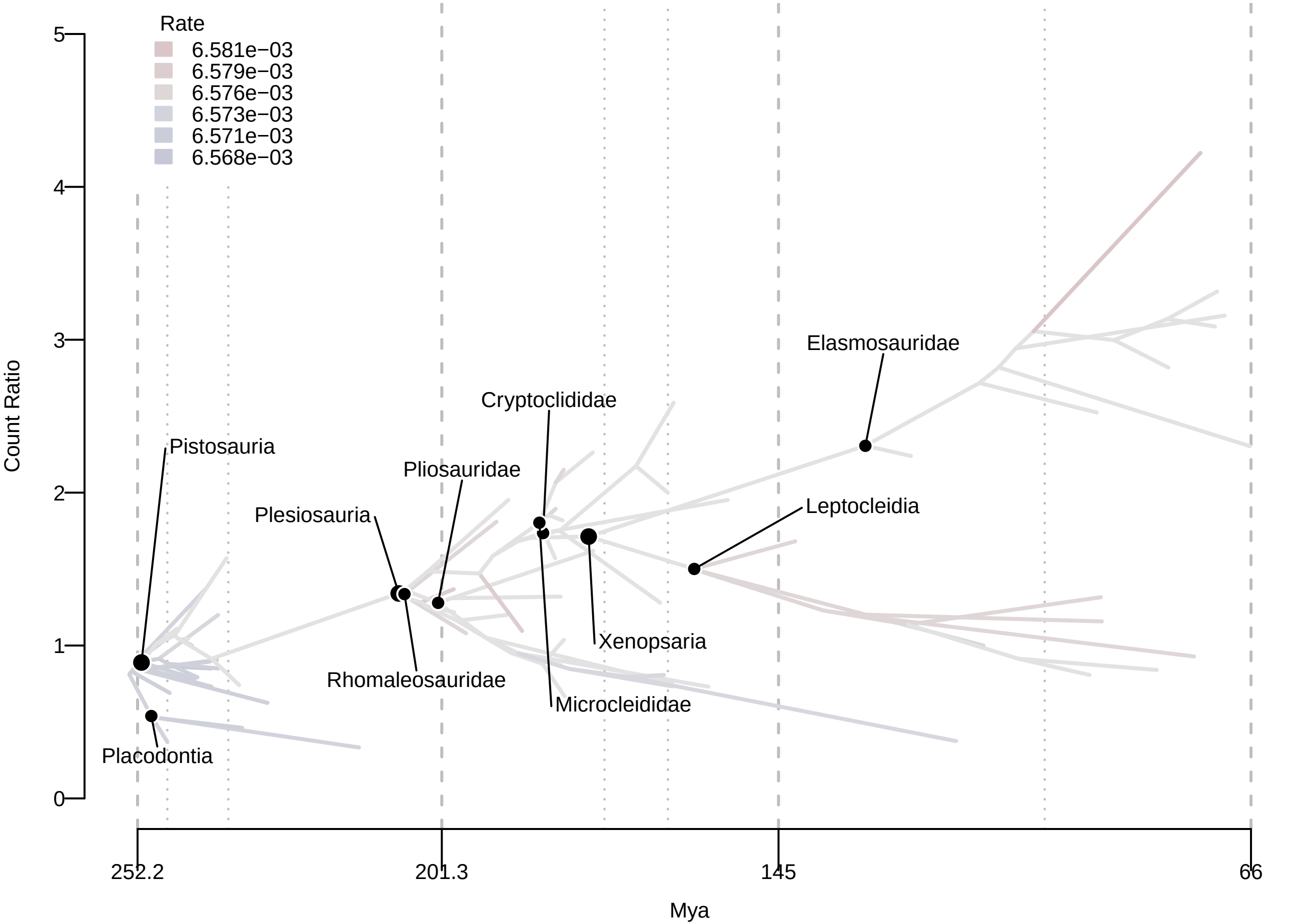

Rate
6.581e−03
6.579e−03
6.576e−03
6.573e−03
6.571e−03
6.568e−03
Count Ratio
0
1
2
3
4
5
Mya
252.2
201.3
145
66
Pistosauria
Placodontia
Plesiosauria
Pliosauridae
Rhomaleosauridae
Cryptoclididae
Microcleididae
Xenopsaria
Leptocleidia
Elasmosauridae

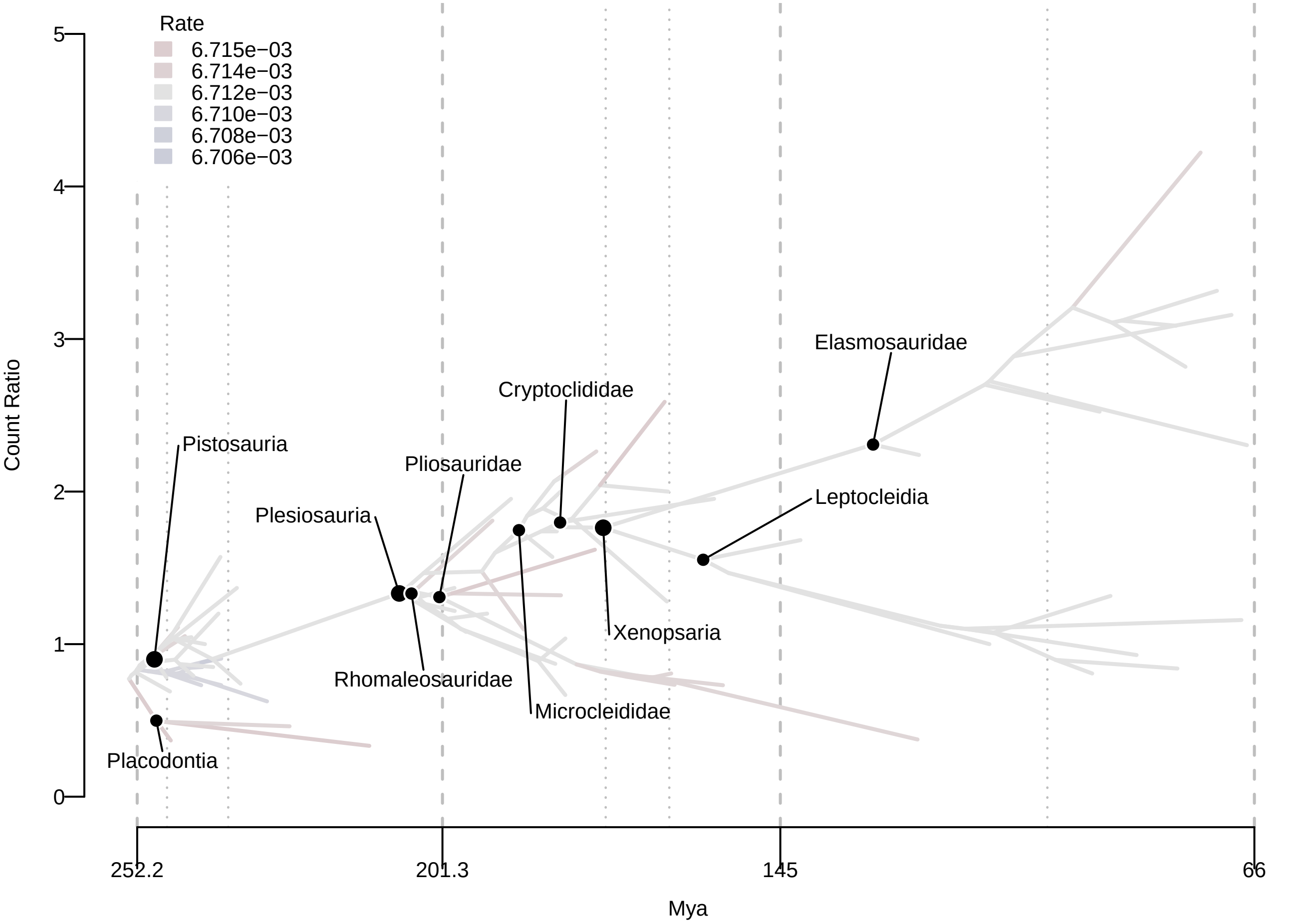

Rate
6.715e−03
6.714e−03
6.712e−03
6.710e−03
6.708e−03
6.706e−03
Count Ratio
0
1
2
3
4
5
Pistosauria
Placodontia
Plesiosauria
Rhomaleosauridae
Pliosauridae
Microcleididae
Cryptoclididae
Xenopsaria
Leptocleidia
Elasmosauridae
252.2
201.3
145
66
Mya

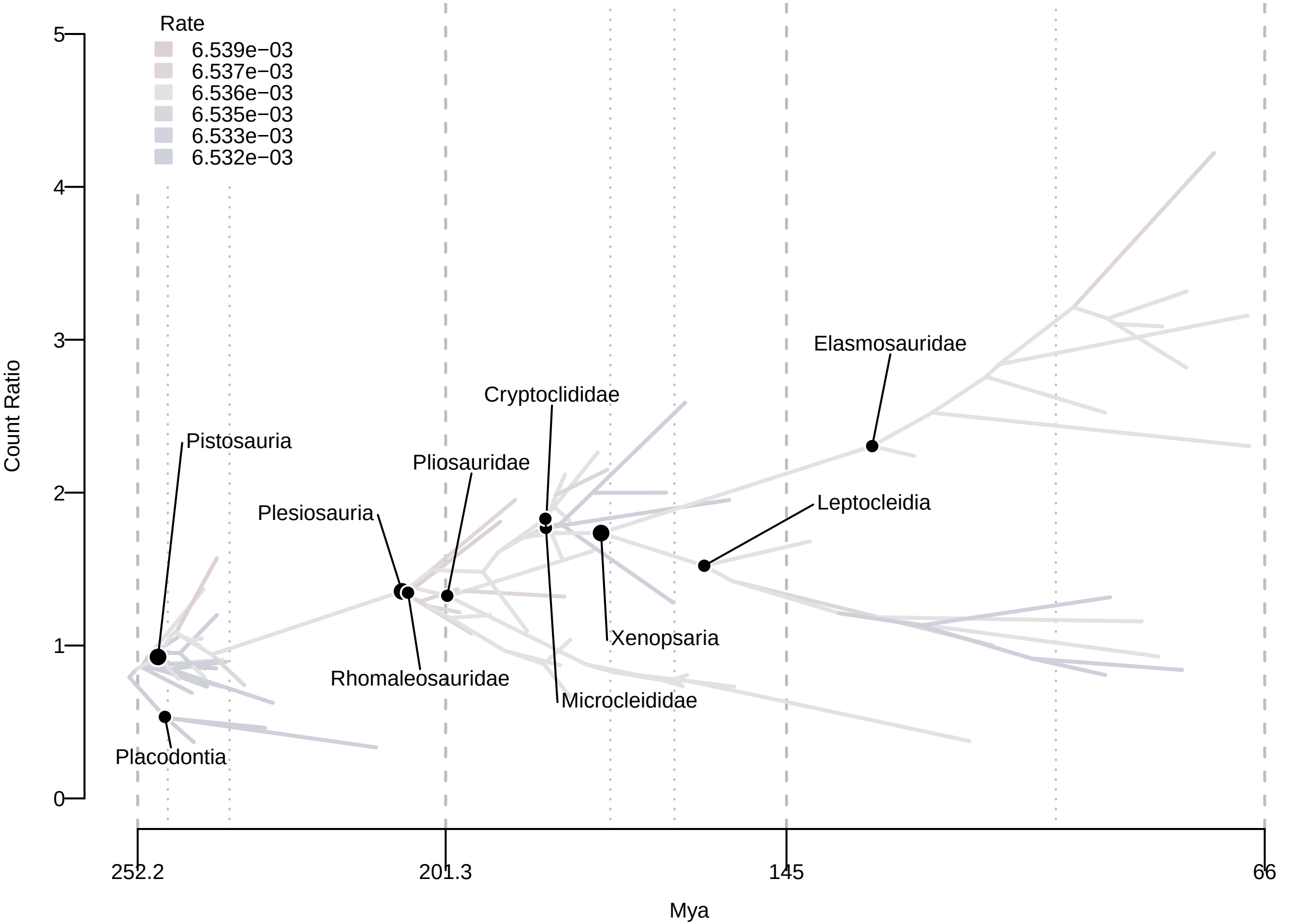

Rate
6.539e−03
6.537e−03
6.536e−03
6.535e−03
6.533e−03
6.532e−03
Count Ratio
0
1
2
3
4
5
Pistosauria
Placodontia
Plesiosauria
Rhomaleosauridae
Pliosauridae
Cryptoclididae
Microcleididae
Xenopsaria
Leptocleidia
Elasmosauridae
252.2
201.3
145
66
Mya

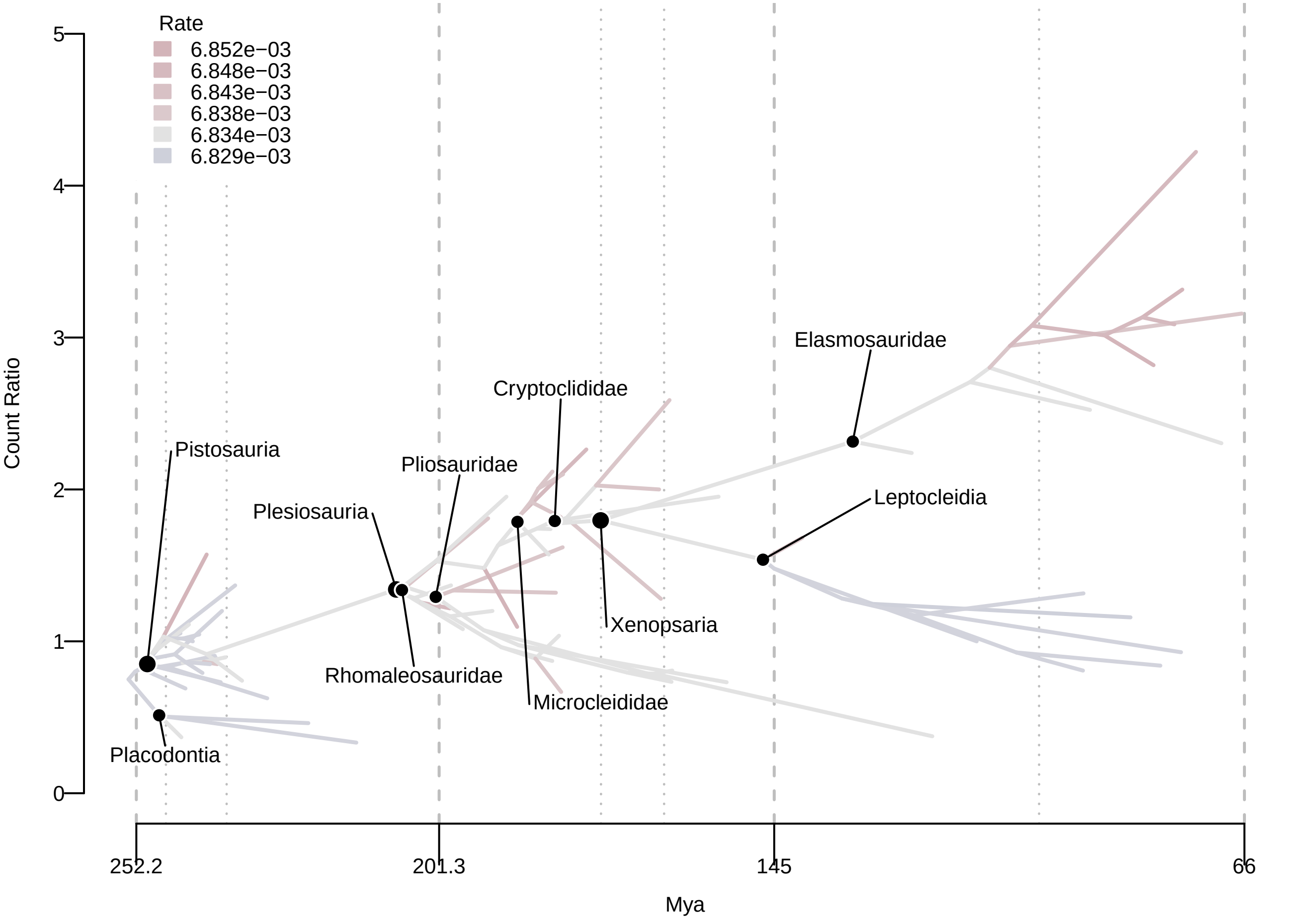
Rate
6.852e−03
6.848e−03
6.843e−03
6.838e−03
6.834e−03
6.829e−03
Count Ratio
0
1
2
3
4
5
Mya
252.2
201.3
145
66
Pistosauria
Placodontia
Plesiosauria
Pliosauridae
Rhomaleosauridae
Cryptoclididae
Microcleididae
Xenopsaria
Elasmosauridae
Leptocleidia

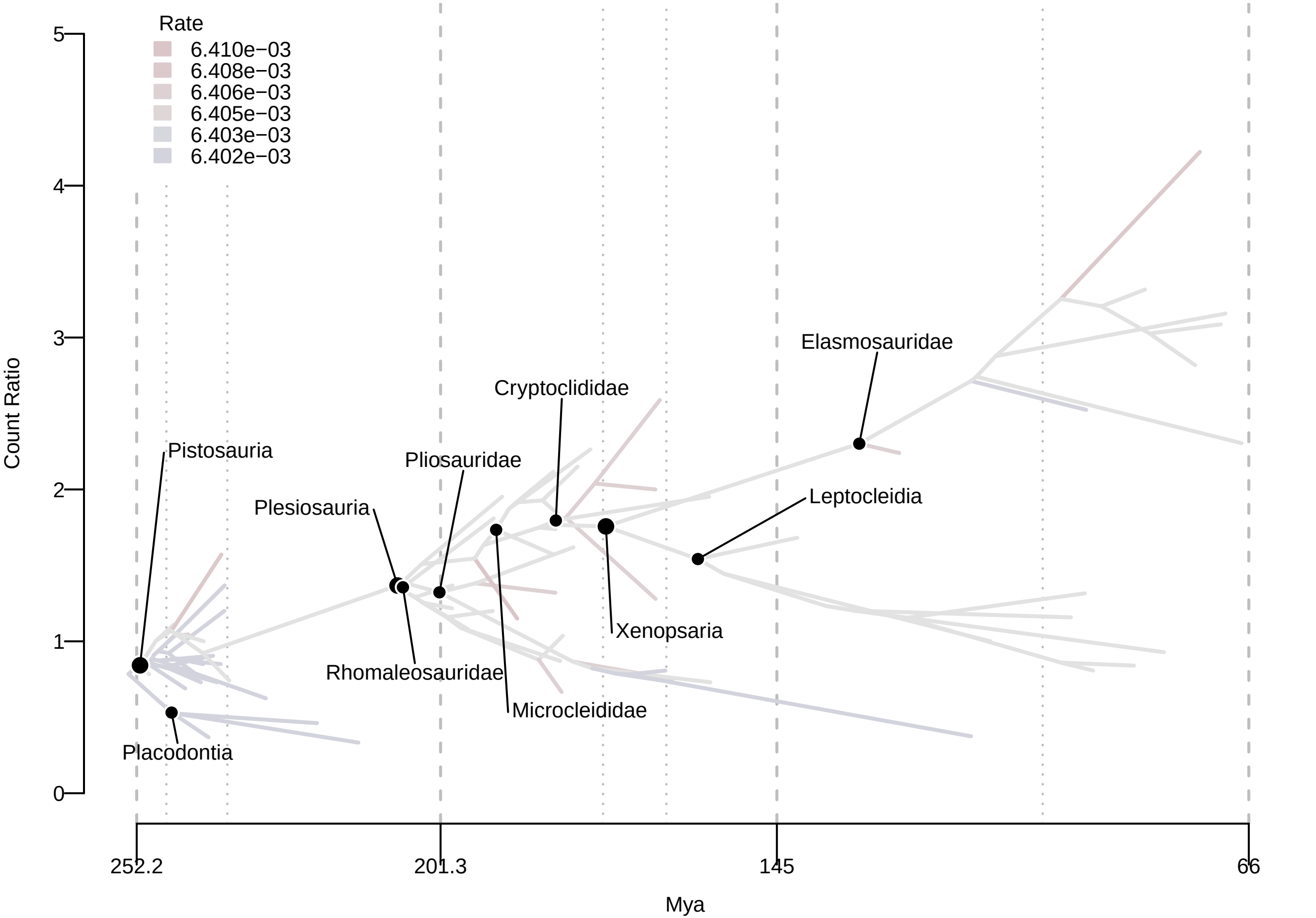

Rate
6.410e−03
6.408e−03
6.406e−03
6.405e−03
6.403e−03
6.402e−03
Count Ratio
0
1
2
3
4
5
Mya
252.2
201.3
145
66
Pistosauria
Placodontia
Plesiosauria
Rhomaleosauridae
Pliosauridae
Microcleididae
Cryptoclididae
Xenopsaria
Leptocleidia
Elasmosauridae

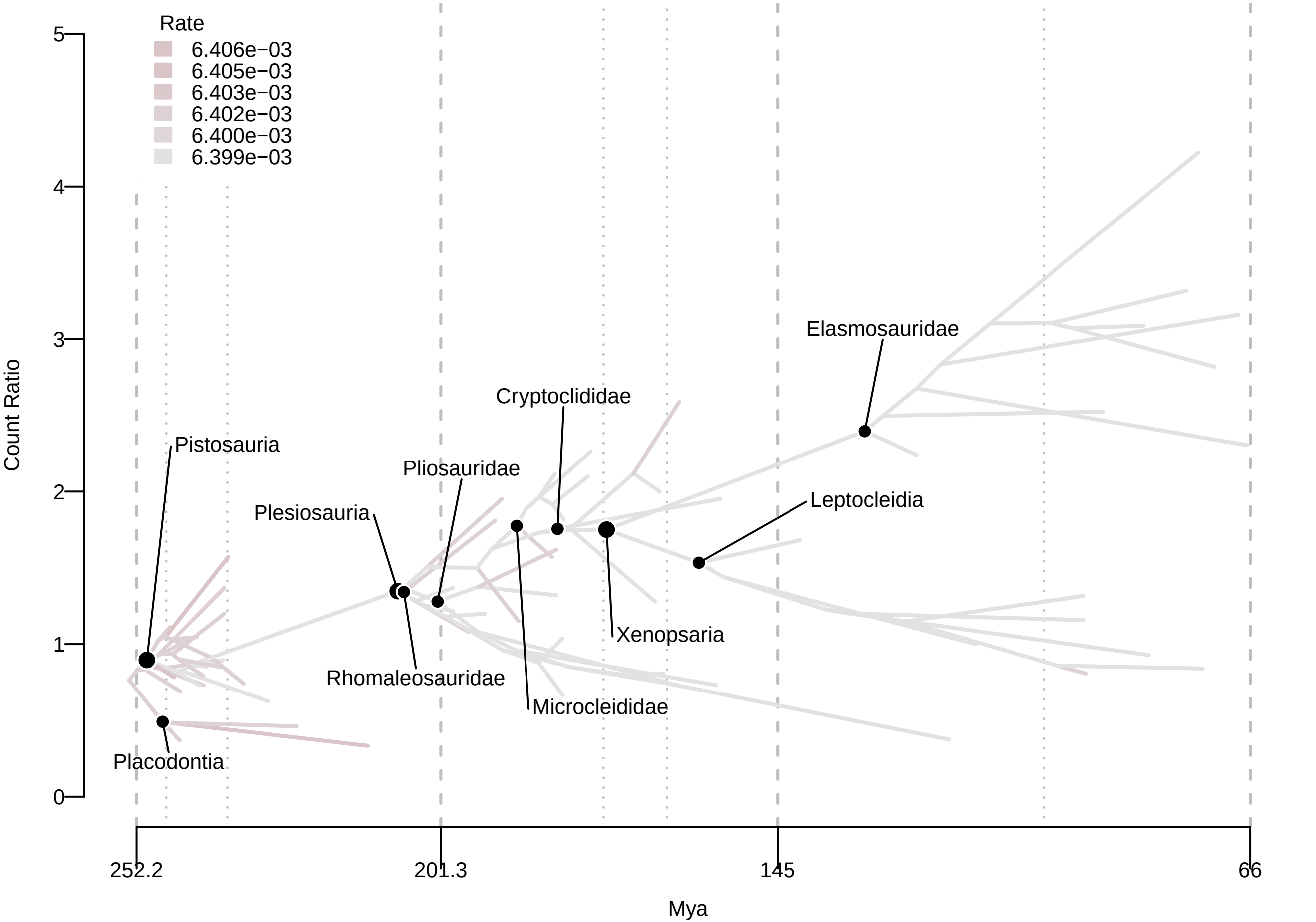

Rate
6.406e−03
6.405e−03
6.403e−03
6.402e−03
6.400e−03
6.399e−03
Count Ratio
0
1
2
3
4
5
Pistosauria
Placodontia
Plesiosauria
Rhomaleosauridae
Pliosauridae
Microcleididae
Cryptoclididae
Xenopsaria
Leptocleidia
Elasmosauridae
252.2
201.3
145
66
Mya

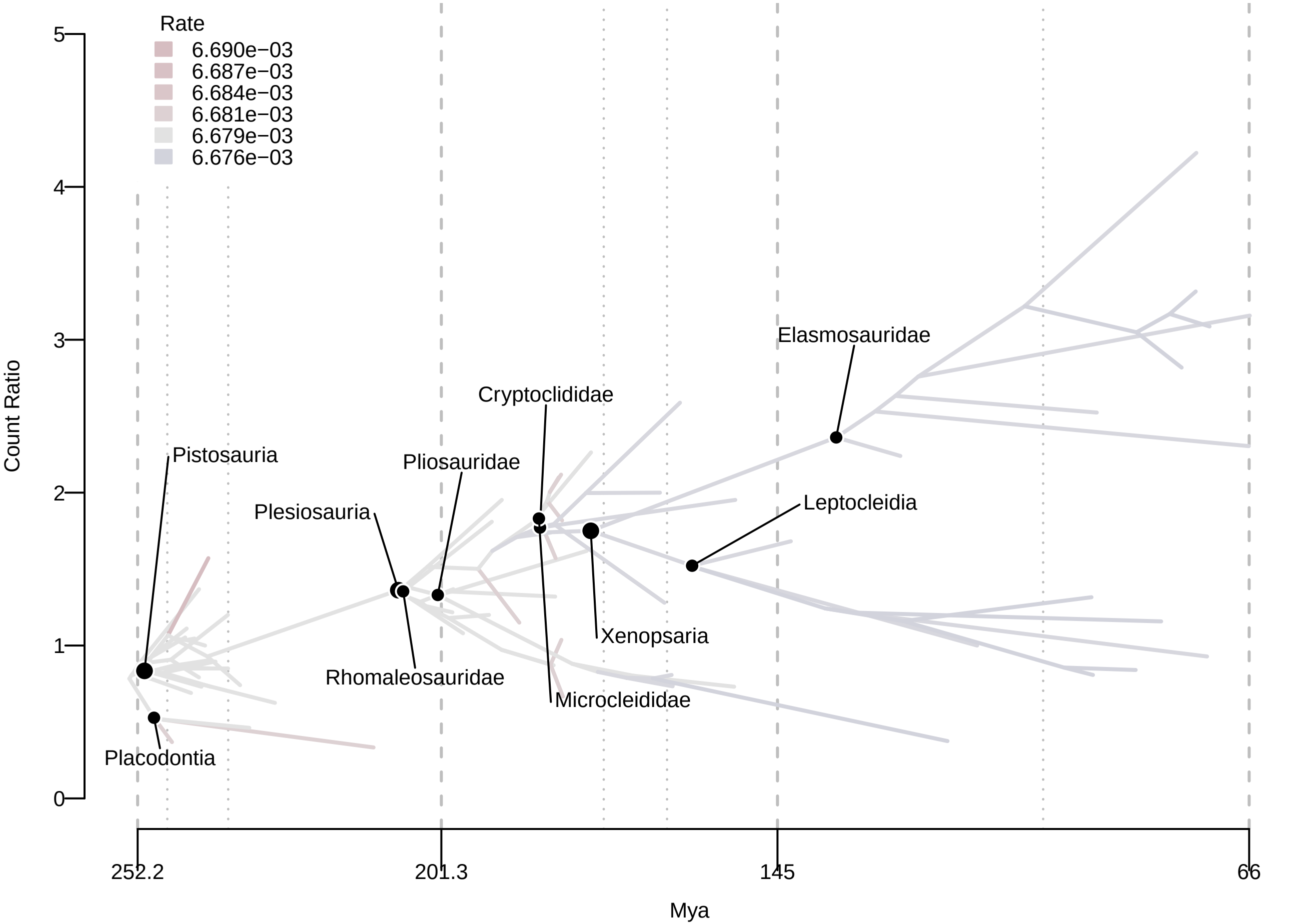

Rate
6.690e−03
6.687e−03
6.684e−03
6.681e−03
6.679e−03
6.676e−03
Count Ratio
0
1
2
3
4
5
Pistosauria
Plesiosauria
Pliosauridae
Cryptoclididae
Elasmosauridae
Leptocleidia
Xenopsaria
Rhomaleosauridae
Microcleididae
Placodontia
252.2
201.3
145
66
Mya

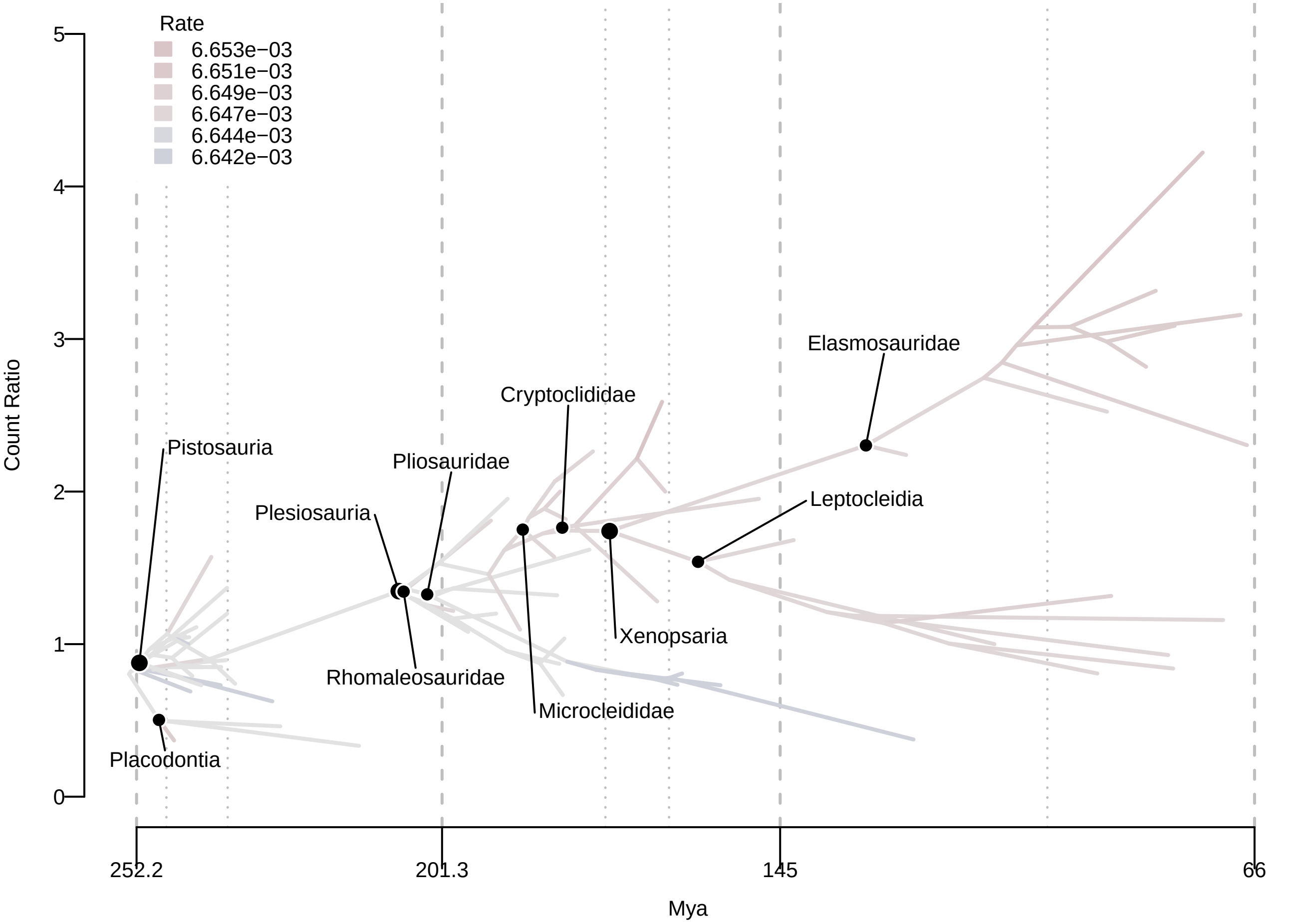

Rate
6.653e−03
6.651e−03
6.649e−03
6.647e−03
6.644e−03
6.642e−03
Count Ratio
0
1
2
3
4
5
Pistosauria
Placodontia
Plesiosauria
Pliosauridae
Rhomaleosauridae
Microcleididae
Cryptoclididae
Xenopsaria
Leptocleidia
Elasmosauridae
252.2
201.3
145
66
Mya

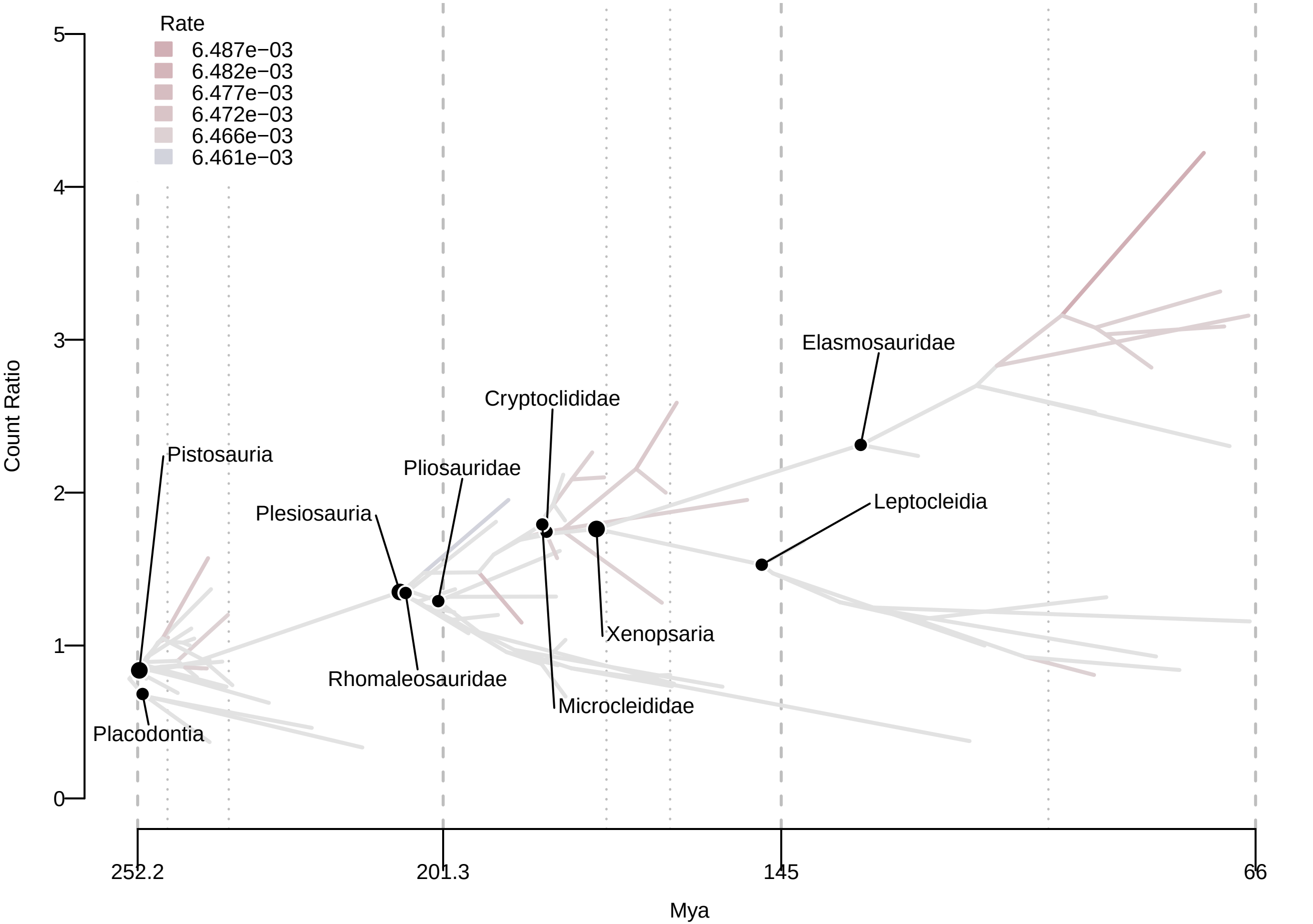

Rate
6.487e−03
6.482e−03
6.477e−03
6.472e−03
6.466e−03
6.461e−03
Count Ratio
5
4
3
2
1
0
Pistosauria
Placodontia
Plesiosauria
Pliosauridae
Rhomaleosauridae
Cryptoclididae
Microcleididae
Xenopsaria
Elasmosauridae
Leptocleidia
252.2
201.3
145
66
Mya

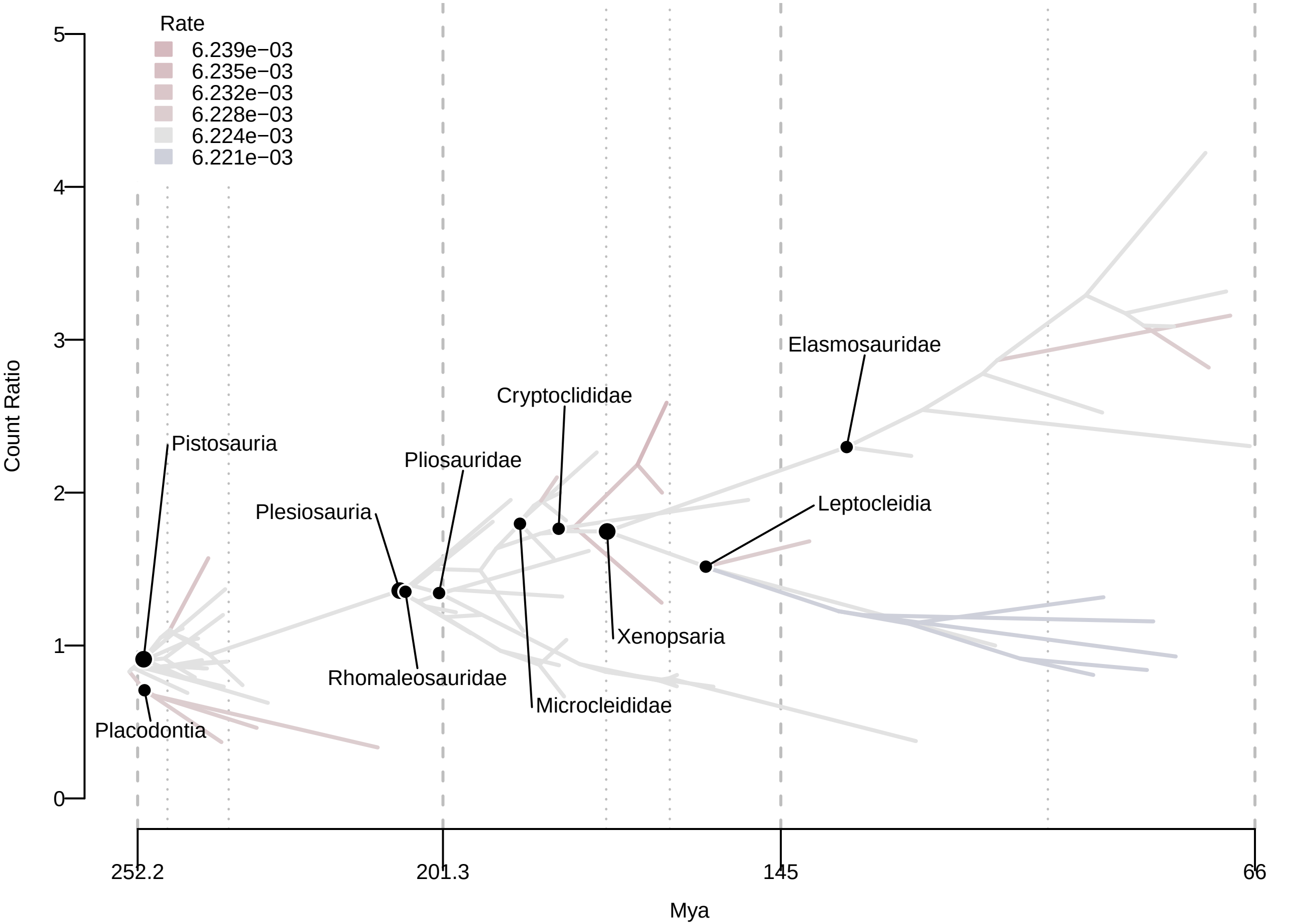

Rate
6.239e−03
6.235e−03
6.232e−03
6.228e−03
6.224e−03
6.221e−03
Count Ratio
0
1
2
3
4
5
Pistosauria
Placodontia
Plesiosauria
Rhomaleosauridae
Pliosauridae
Cryptoclididae
Microcleididae
Xenopsaria
Leptocleidia
Elasmosauridae
252.2
201.3
145
66
Mya

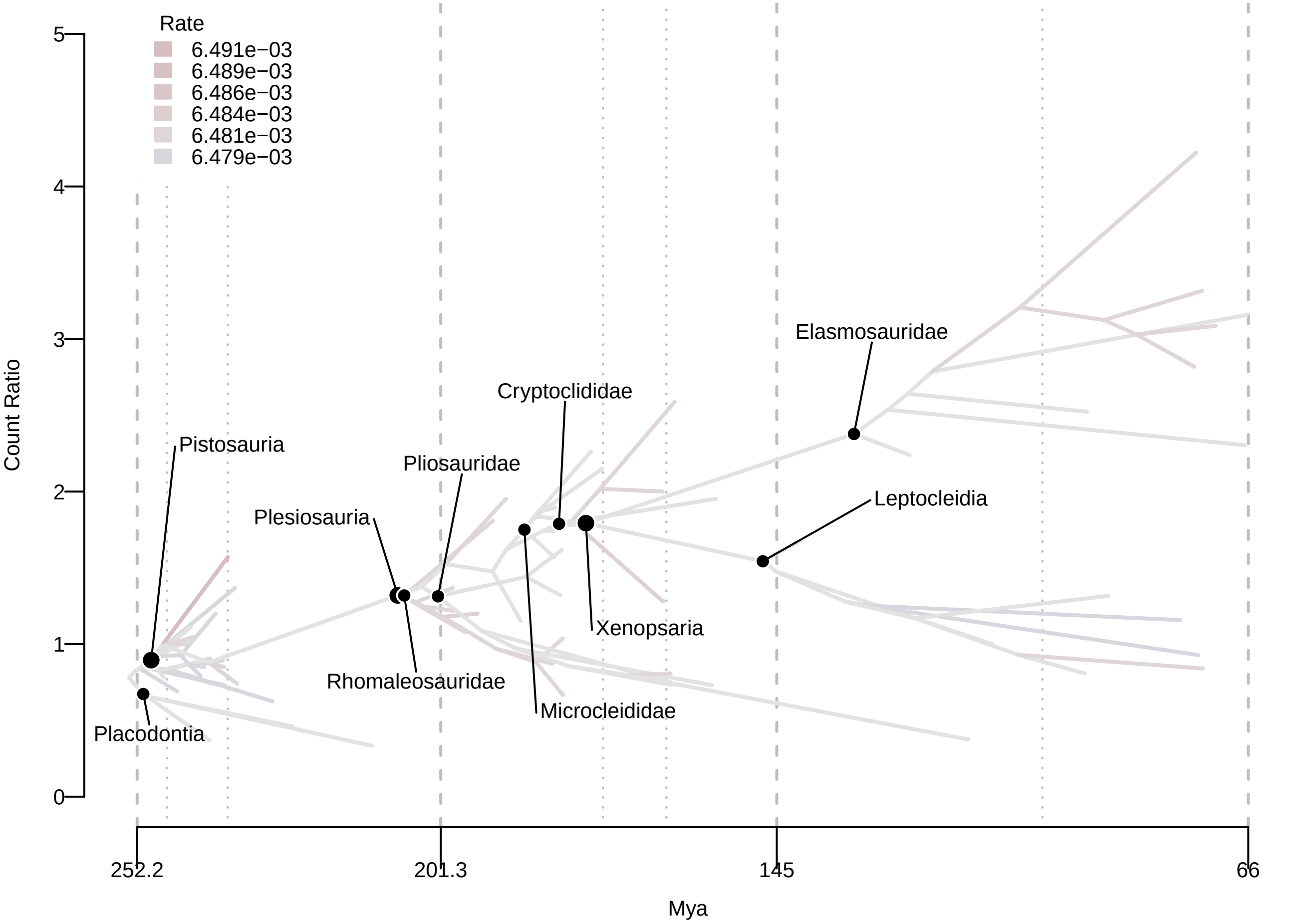

Rate
6.491e−03
6.489e−03
6.486e−03
6.484e−03
6.481e−03
6.479e−03
Count Ratio
0
1
2
3
4
5
Pistosauria
Placodontia
Plesiosauria
Rhomaleosauridae
Pliosauridae
Cryptoclididae
Microcleididae
Xenopsaria
Elasmosauridae
Leptocleidia
252.2
201.3
145
66
Mya

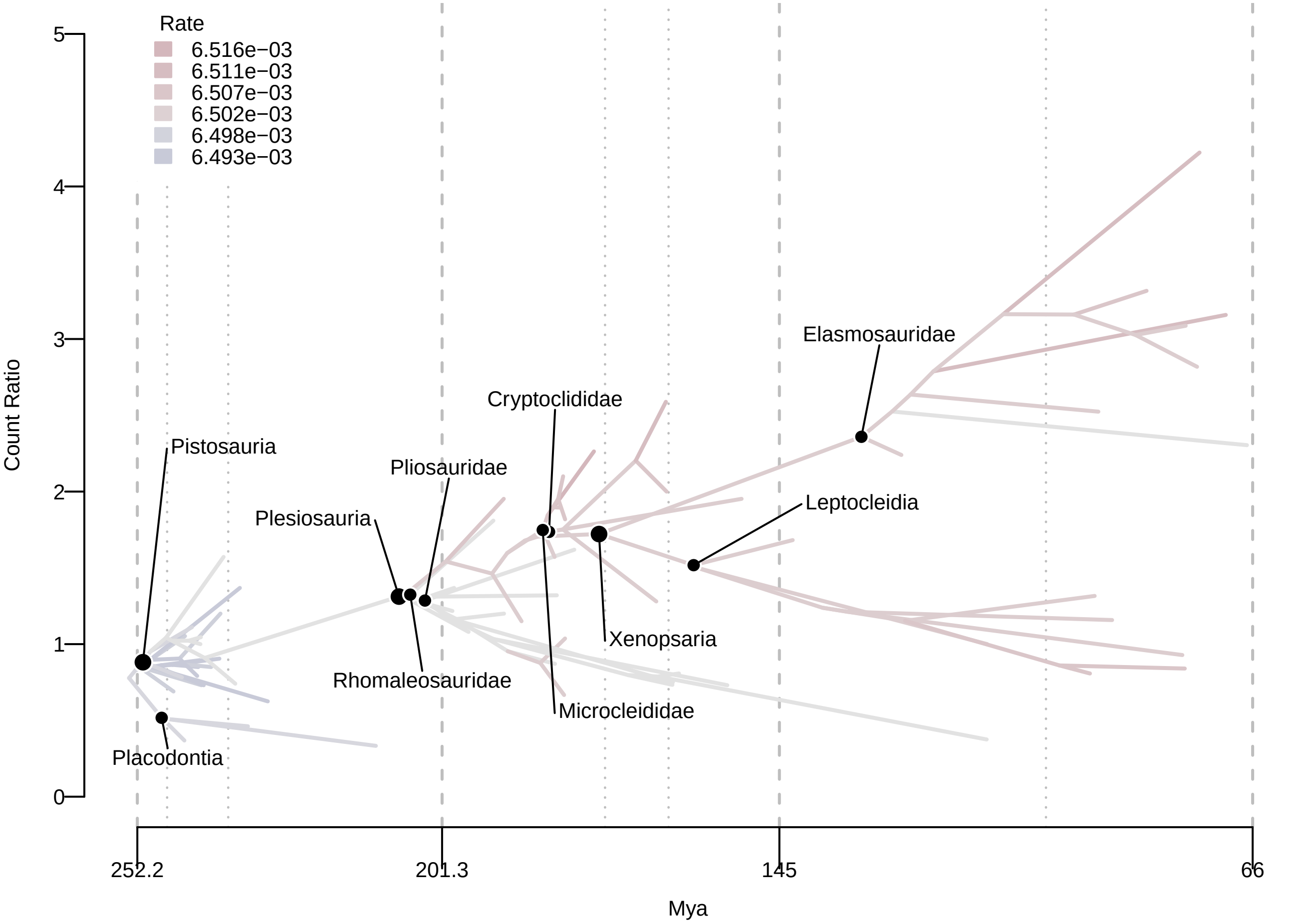

Rate
6.516e−03
6.511e−03
6.507e−03
6.502e−03
6.498e−03
6.493e−03
Count Ratio
0
1
2
3
4
5
Pistosauria
Placodontia
Plesiosauria
Pliosauridae
Rhomaleosauridae
Cryptoclididae
Microcleididae
Xenopsaria
Leptocleidia
Elasmosauridae
252.2
201.3
145
66
Mya

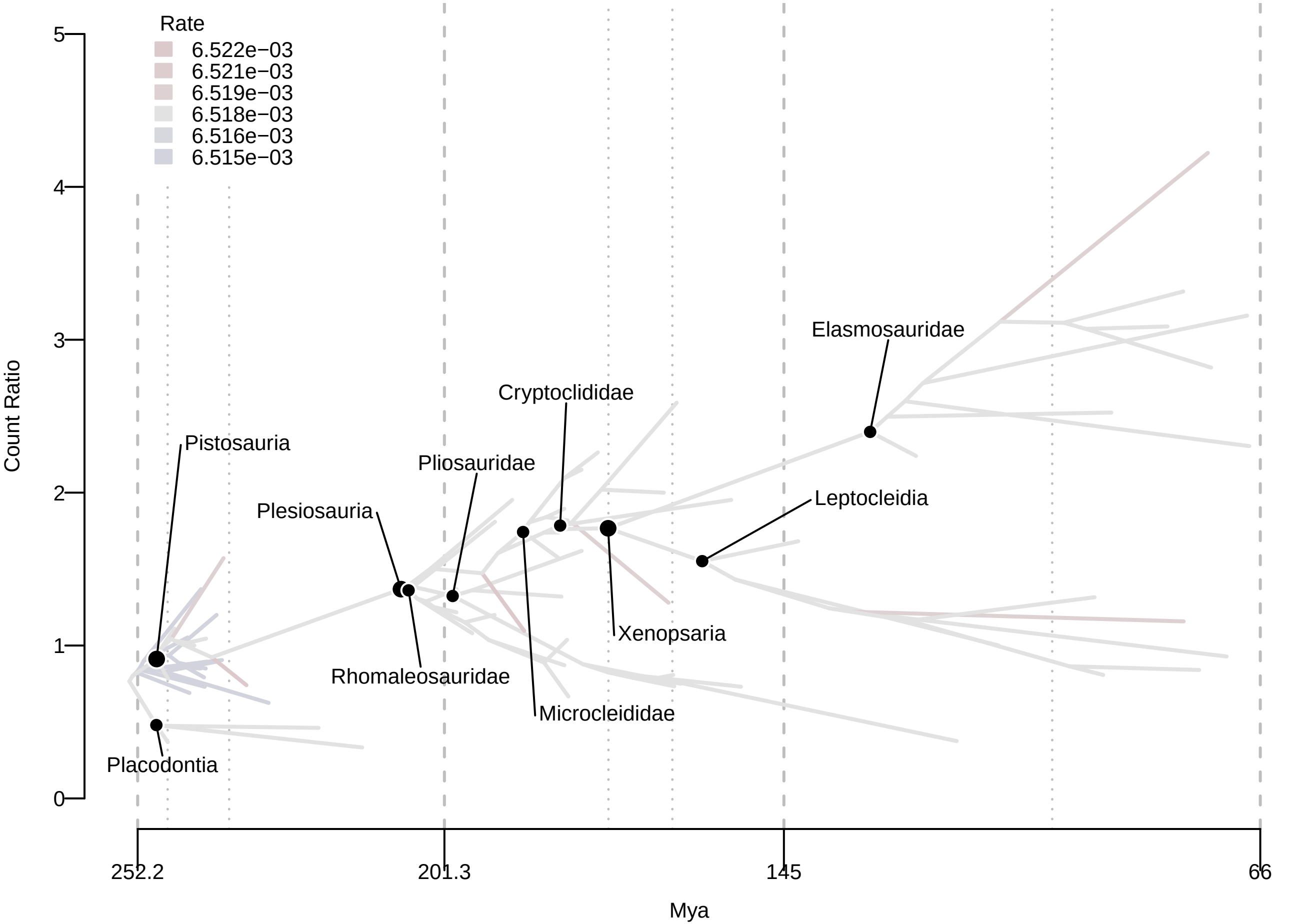

Rate
6.522e−03
6.521e−03
6.519e−03
6.518e−03
6.516e−03
6.515e−03
Count Ratio
0
1
2
3
4
5
Pistosauria
Placodontia
Plesiosauria
Rhomaleosauridae
Pliosauridae
Cryptoclididae
Microcleididae
Xenopsaria
Leptocleidia
Elasmosauridae
252.2
201.3
145
66
Mya

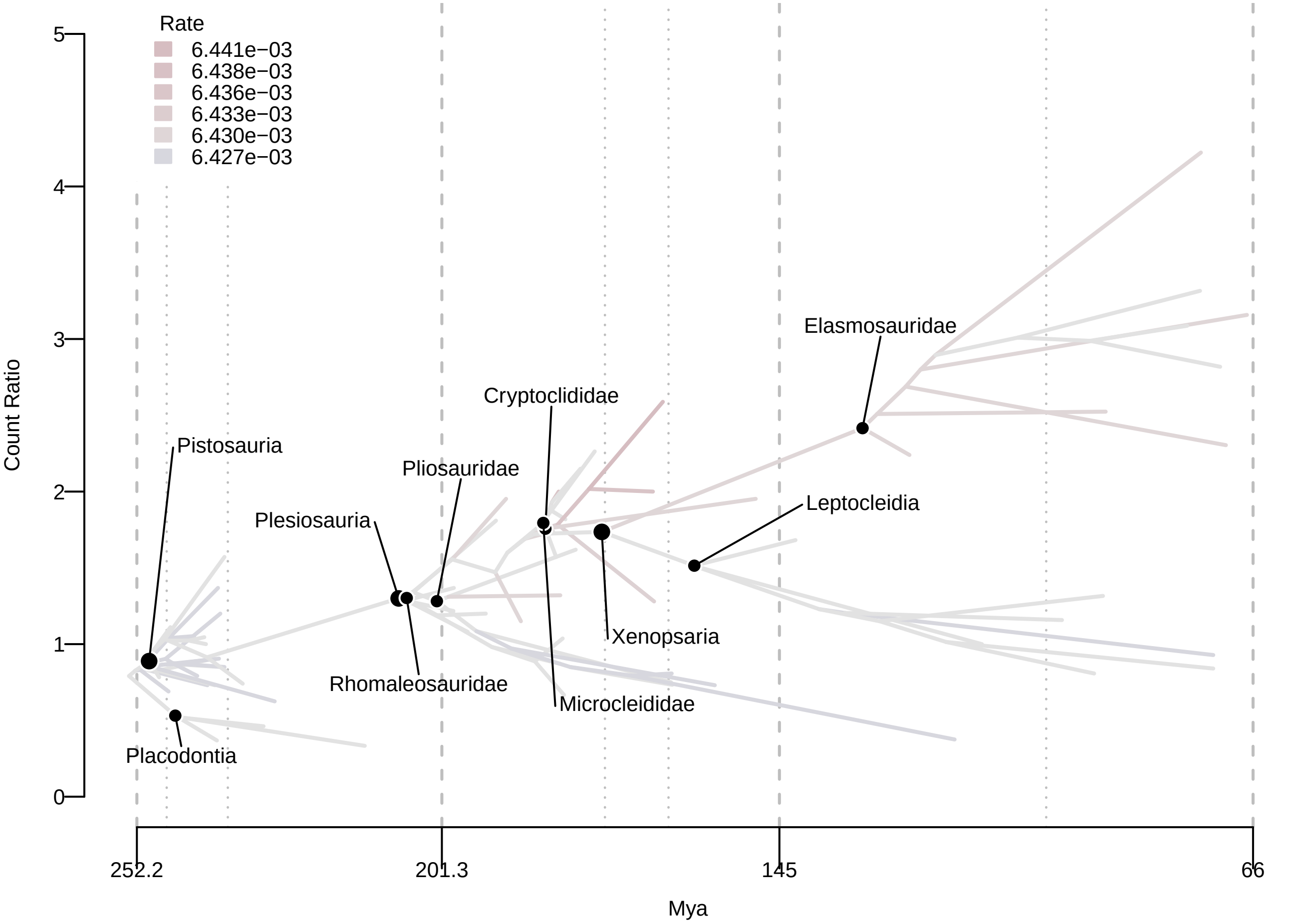
Rate
6.441e−03
6.438e−03
6.436e−03
6.433e−03
6.430e−03
6.427e−03
Count Ratio
0
1
2
3
4
5
Pistosauria
Placodontia
Plesiosauria
Rhomaleosauridae
Pliosauridae
Cryptoclididae
Microcleididae
Xenopsaria
Leptocleidia
Elasmosauridae
252.2
201.3
145
66
Mya

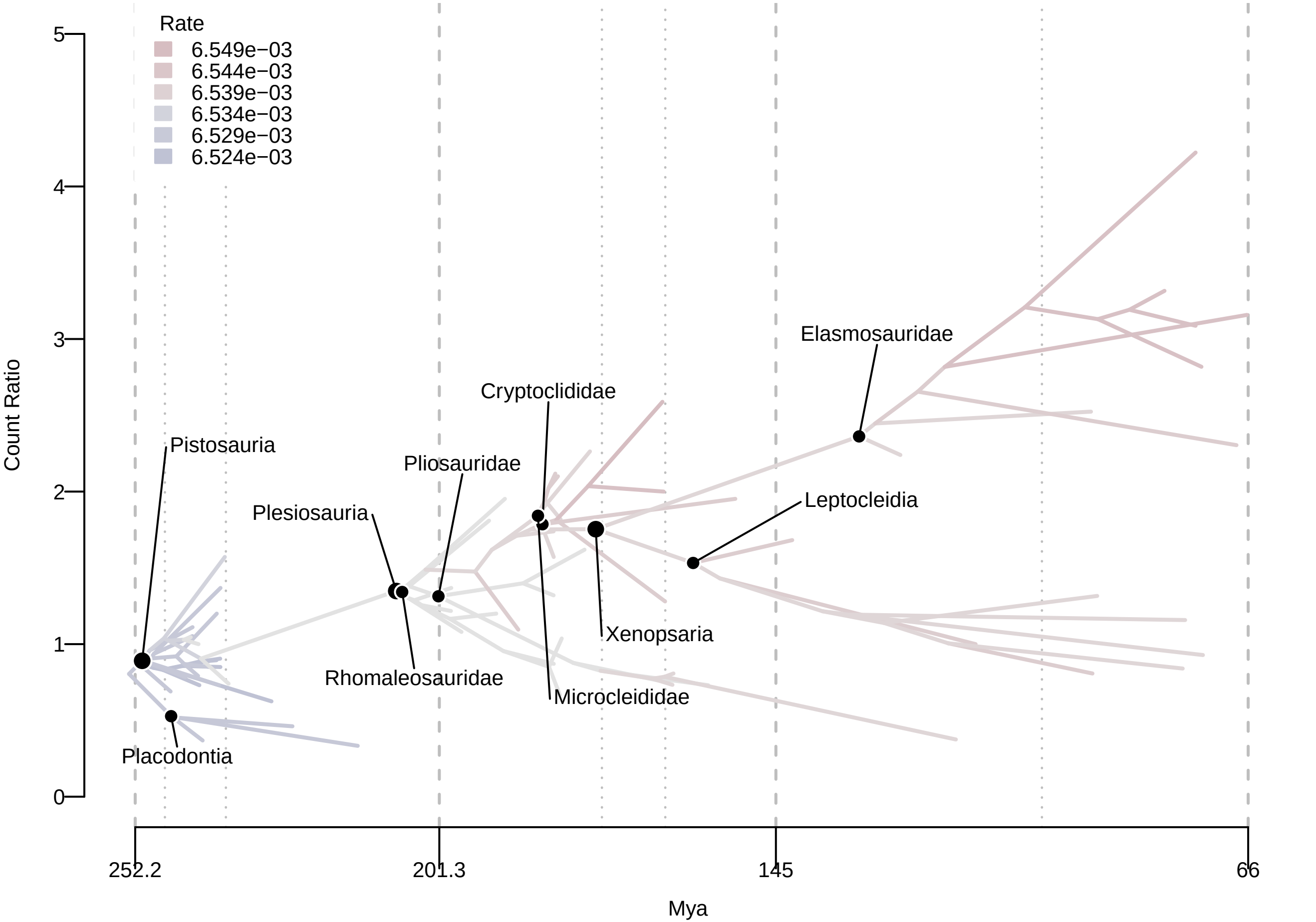
Rate
6.549e−03
6.544e−03
6.539e−03
6.534e−03
6.529e−03
6.524e−03
Count Ratio
0
1
2
3
4
5
Mya
252.2
201.3
145
66
Pistosauria
Placodontia
Plesiosauria
Rhomaleosauridae
Pliosauridae
Cryptoclididae
Microcleididae
Xenopsaria
Leptocleidia
Elasmosauridae

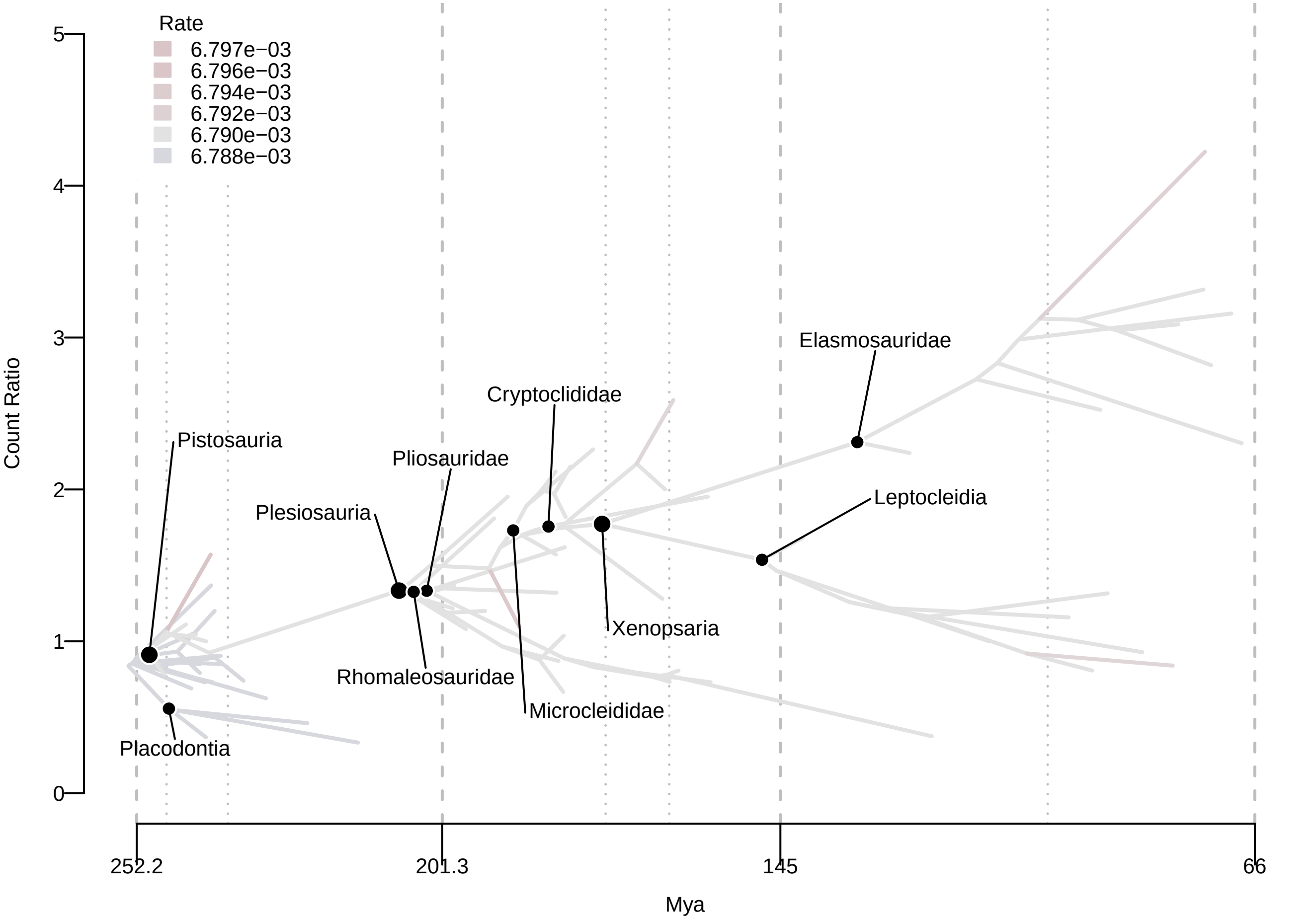
Rate
6.797e−03
6.796e−03
6.794e−03
6.792e−03
6.790e−03
6.788e−03
Count Ratio
0
1
2
3
4
5
Mya
252.2
201.3
145
66
Pistosauria
Placodontia
Plesiosauria
Pliosauridae
Rhomaleosauridae
Cryptoclididae
Microcleididae
Xenopsaria
Elasmosauridae
Leptocleidia

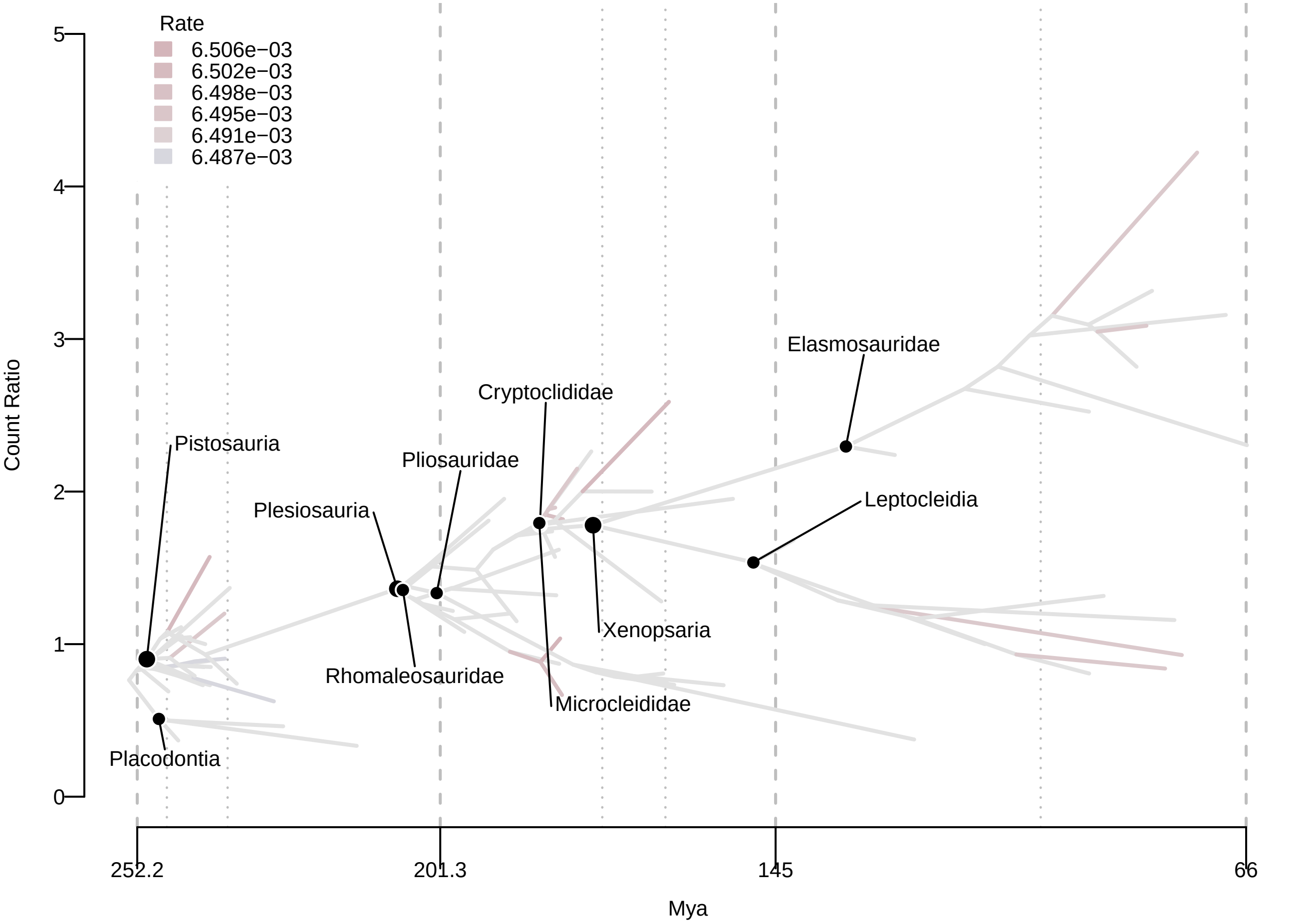
Rate
6.506e−03
6.502e−03
6.498e−03
6.495e−03
6.491e−03
6.487e−03
Count Ratio
0
1
2
3
4
5
Pistosauria
Placodontia
Plesiosauria
Rhomaleosauridae
Pliosauridae
Cryptoclididae
Microcleididae
Xenopsaria
Elasmosauridae
Leptocleidia
252.2
201.3
145
66
Mya

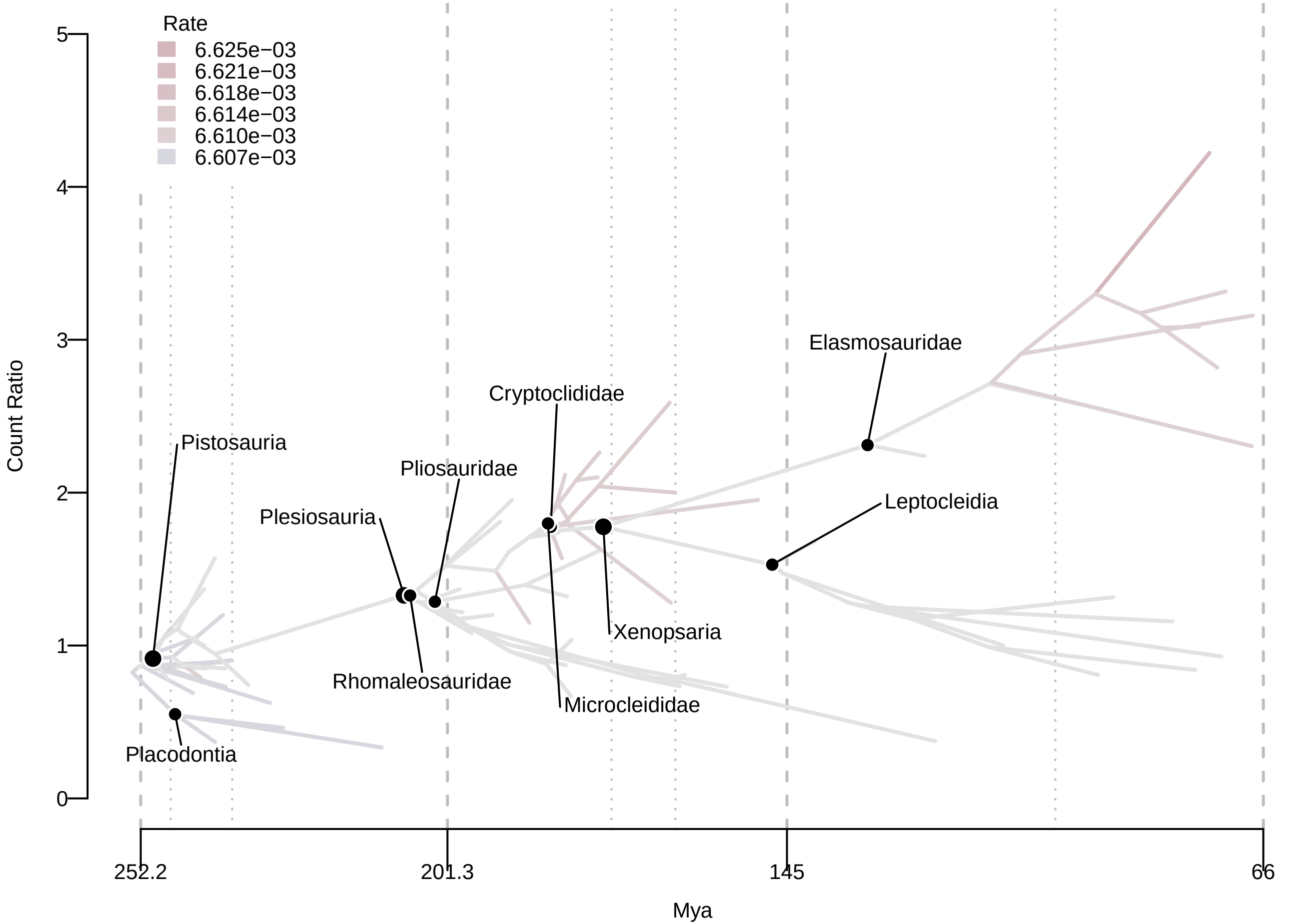

Rate
6.625e−03
6.621e−03
6.618e−03
6.614e−03
6.610e−03
6.607e−03
Count Ratio
0
1
2
3
4
5
Pistosauria
Placodontia
Plesiosauria
Pliosauridae
Rhomaleosauridae
Cryptoclididae
Microcleididae
Xenopsaria
Leptocleidia
Elasmosauridae
252.2
201.3
145
66
Mya

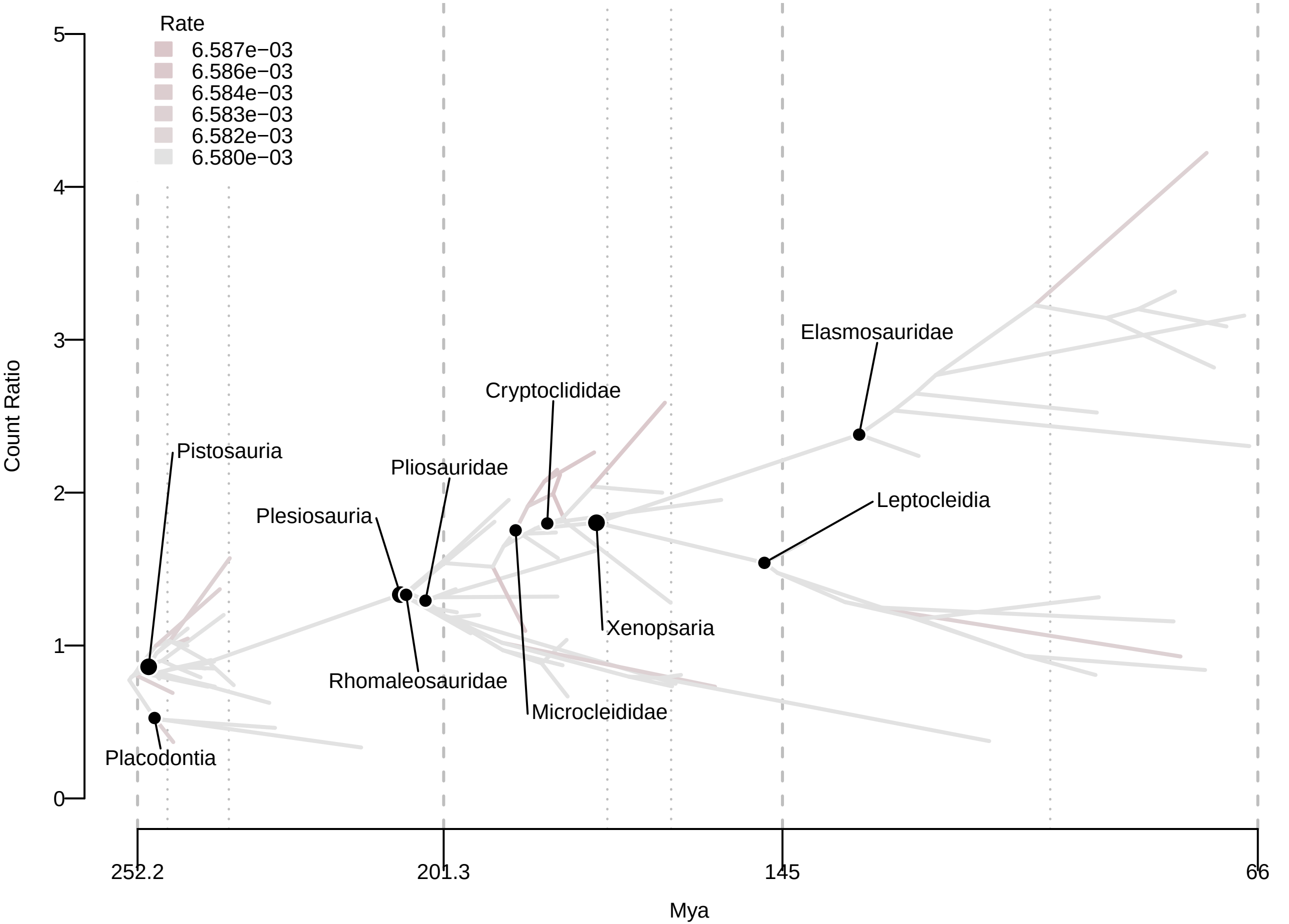
Rate
6.587e−03
6.586e−03
6.584e−03
6.583e−03
6.582e−03
6.580e−03
Count Ratio
0
1
2
3
4
5
Pistosauria
Placodontia
Plesiosauria
Pliosauridae
Rhomaleosauridae
Cryptoclididae
Microcleididae
Xenopsaria
Elasmosauridae
Leptocleidia
252.2
201.3
145
66
Mya

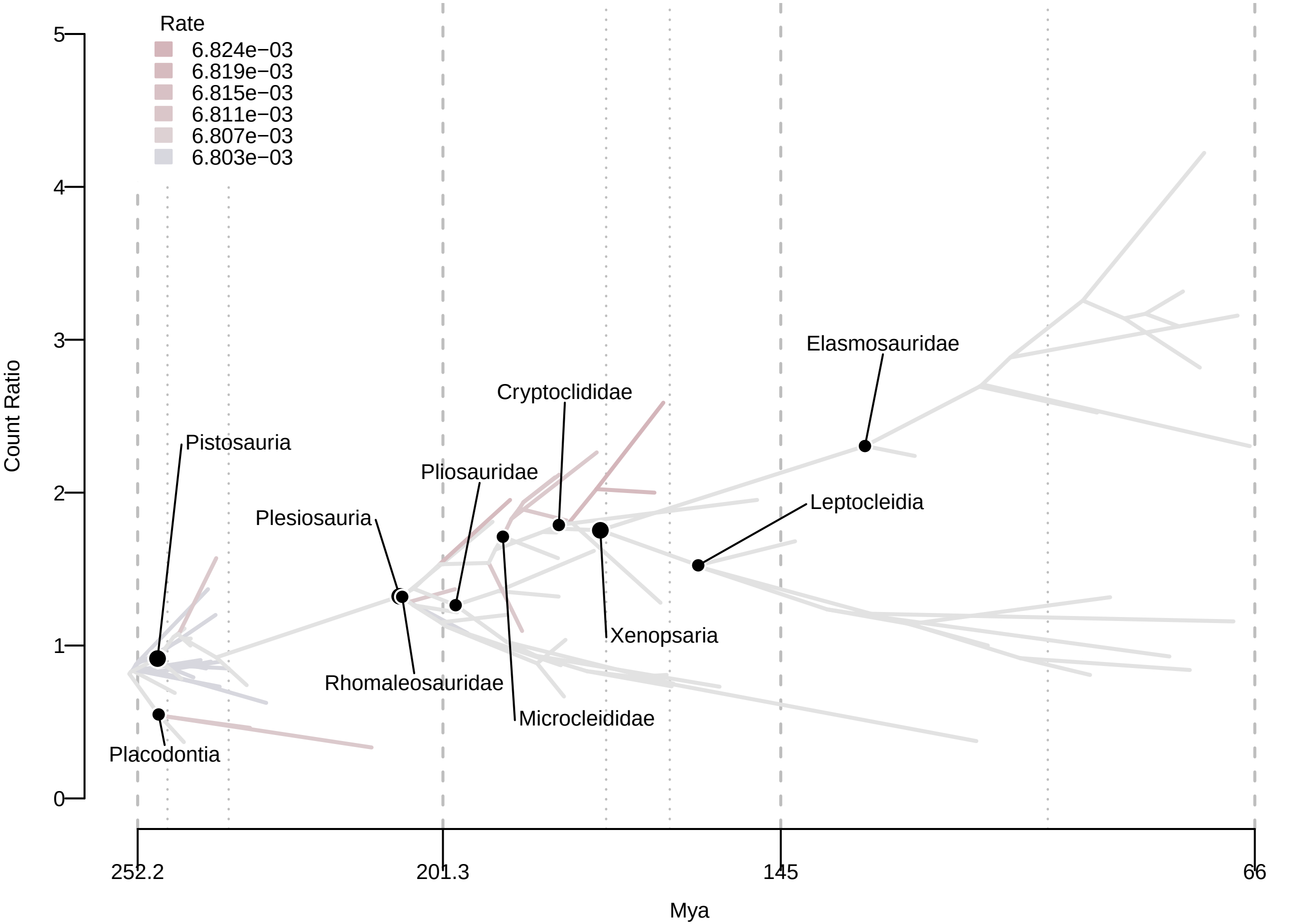

Rate
6.824e−03
6.819e−03
6.815e−03
6.811e−03
6.807e−03
6.803e−03
Count Ratio
0
1
2
3
4
5
Pistosauria
Placodontia
Plesiosauria
Rhomaleosauridae
Pliosauridae
Microcleididae
Cryptoclididae
Xenopsaria
Leptocleidia
Elasmosauridae
252.2
201.3
145
66
Mya

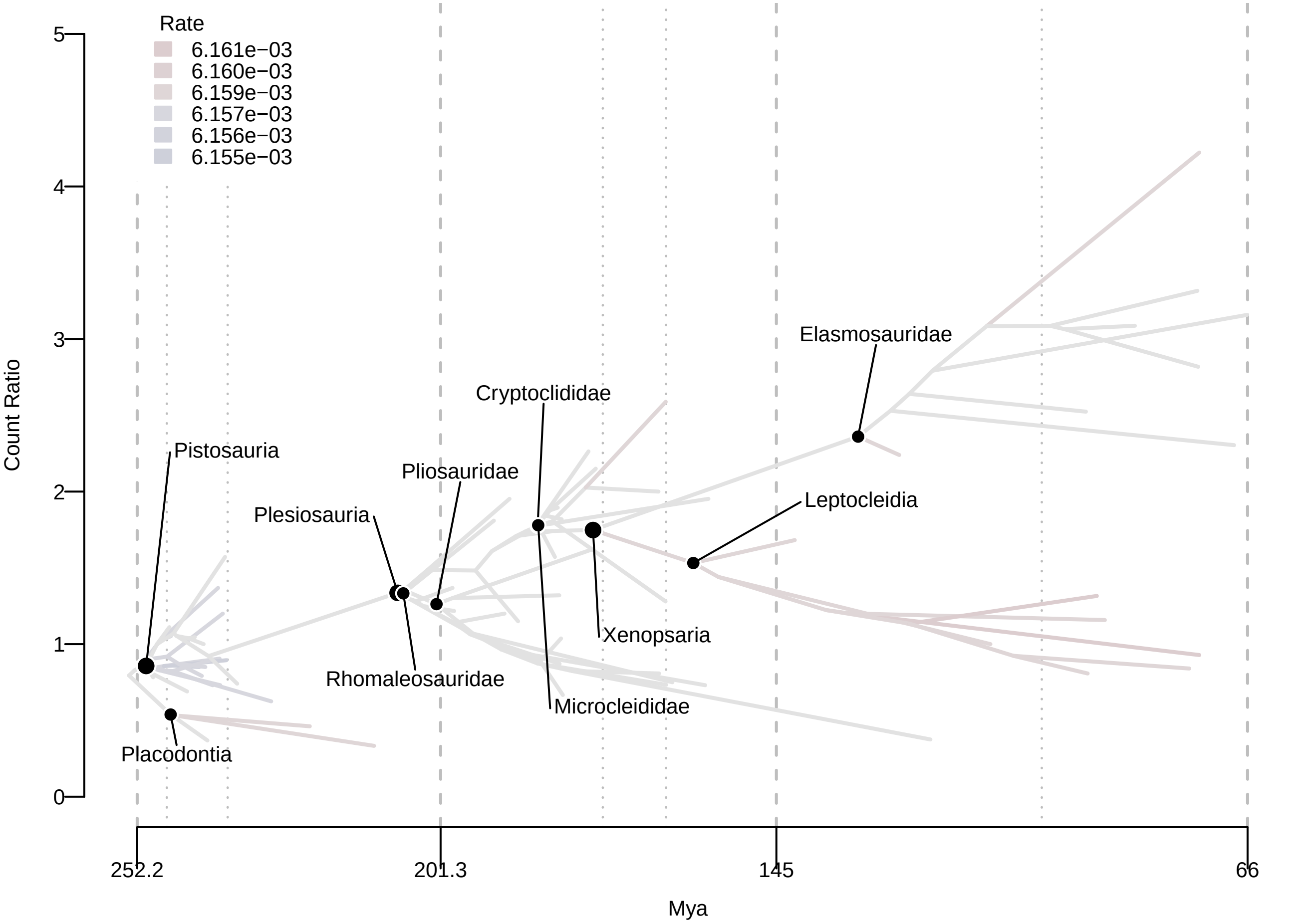
Rate
6.161e−03
6.160e−03
6.159e−03
6.157e−03
6.156e−03
6.155e−03
Count Ratio
0
1
2
3
4
5
Pistosauria
Placodontia
Plesiosauria
Pliosauridae
Rhomaleosauridae
Cryptoclididae
Microcleididae
Xenopsaria
Leptocleidia
Elasmosauridae
252.2
201.3
145
66
Mya

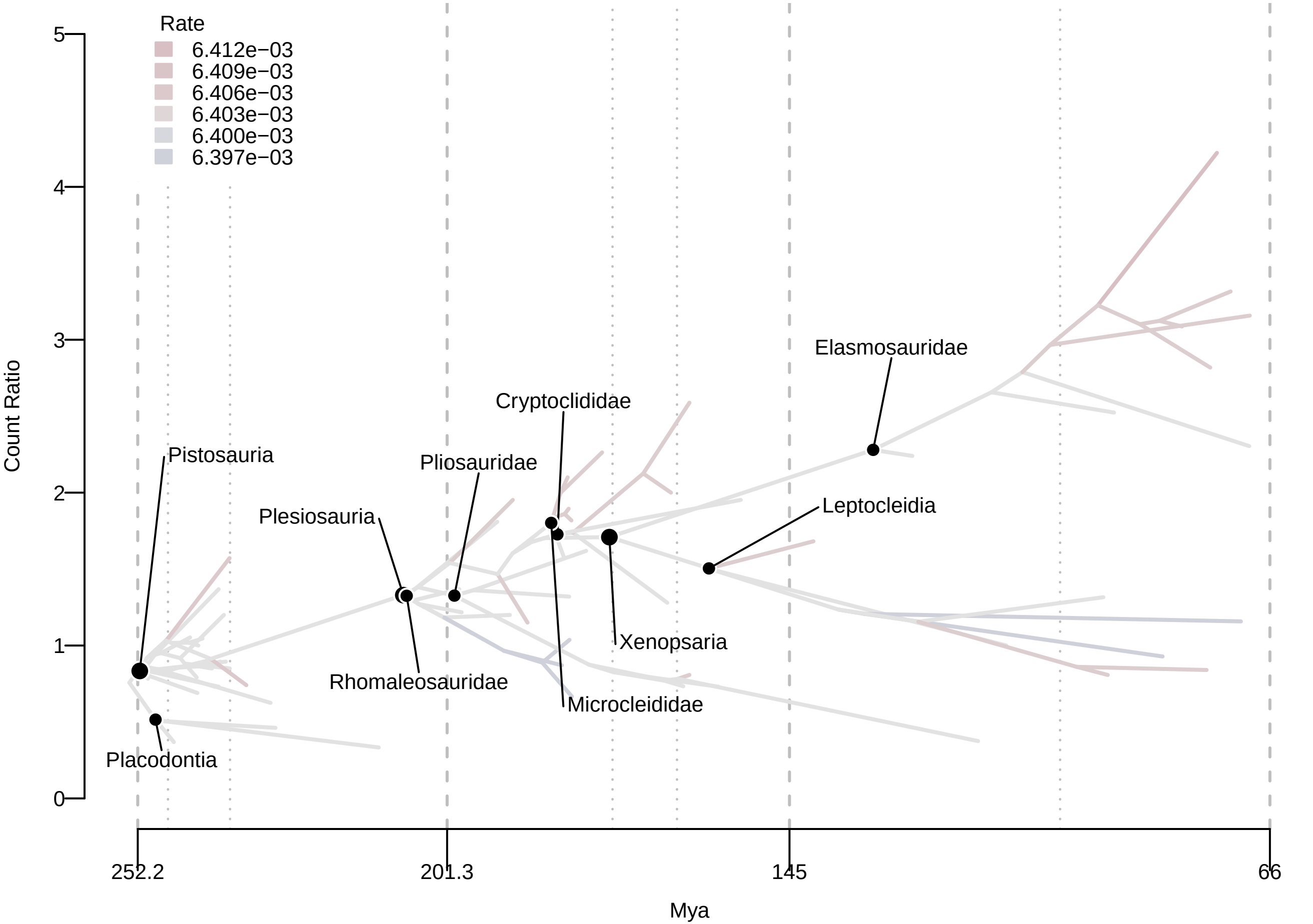

Rate
6.412e−03
6.409e−03
6.406e−03
6.403e−03
6.400e−03
6.397e−03
Count Ratio
0
1
2
3
4
5
Pistosauria
Placodontia
Plesiosauria
Rhomaleosauridae
Pliosauridae
Cryptoclididae
Microcleididae
Xenopsaria
Leptocleidia
Elasmosauridae
252.2
201.3
145
66
Mya

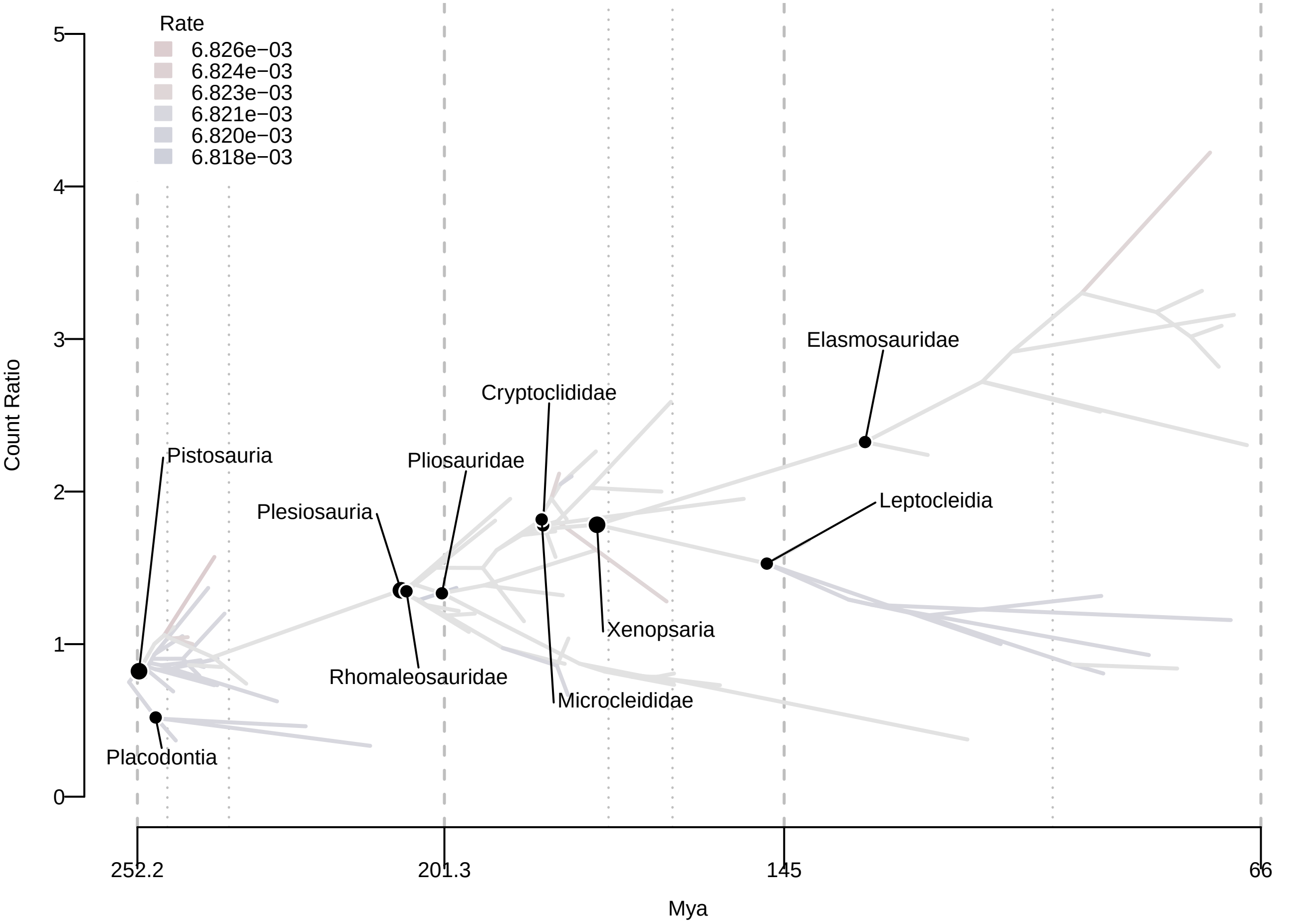

Rate
6.826e−03
6.824e−03
6.823e−03
6.821e−03
6.820e−03
6.818e−03
Count Ratio
0
1
2
3
4
5
Pistosauria
Placodontia
Plesiosauria
Rhomaleosauridae
Pliosauridae
Cryptoclididae
Microcleididae
Xenopsaria
Elasmosauridae
Leptocleidia
252.2
201.3
145
66
Mya

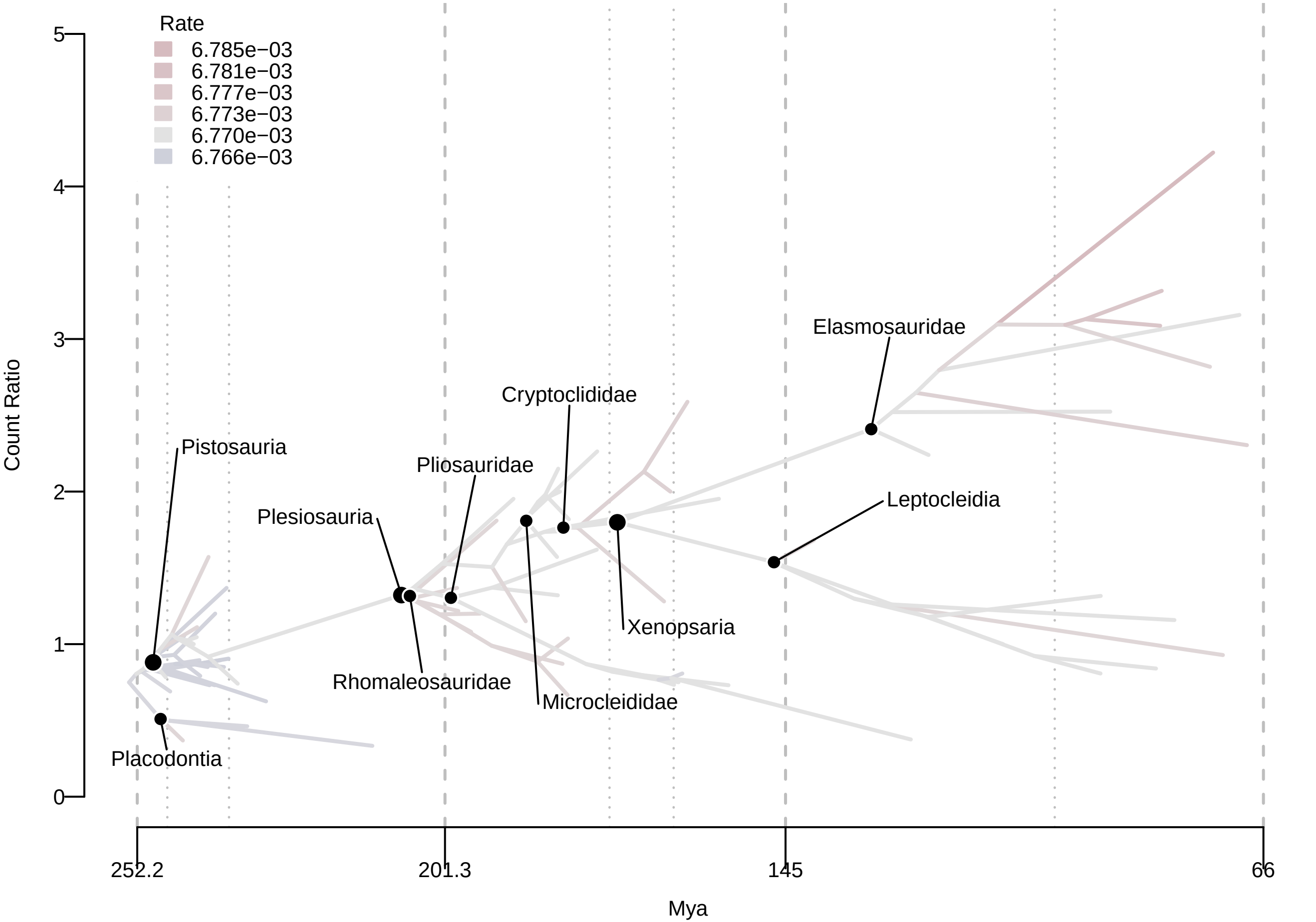

Rate
6.785e−03
6.781e−03
6.777e−03
6.773e−03
6.770e−03
6.766e−03
Count Ratio
0
1
2
3
4
5
Pistosauria
Placodontia
Plesiosauria
Rhomaleosauridae
Pliosauridae
Microcleididae
Cryptoclididae
Xenopsaria
Leptocleidia
Elasmosauridae
252.2
201.3
145
66
Mya

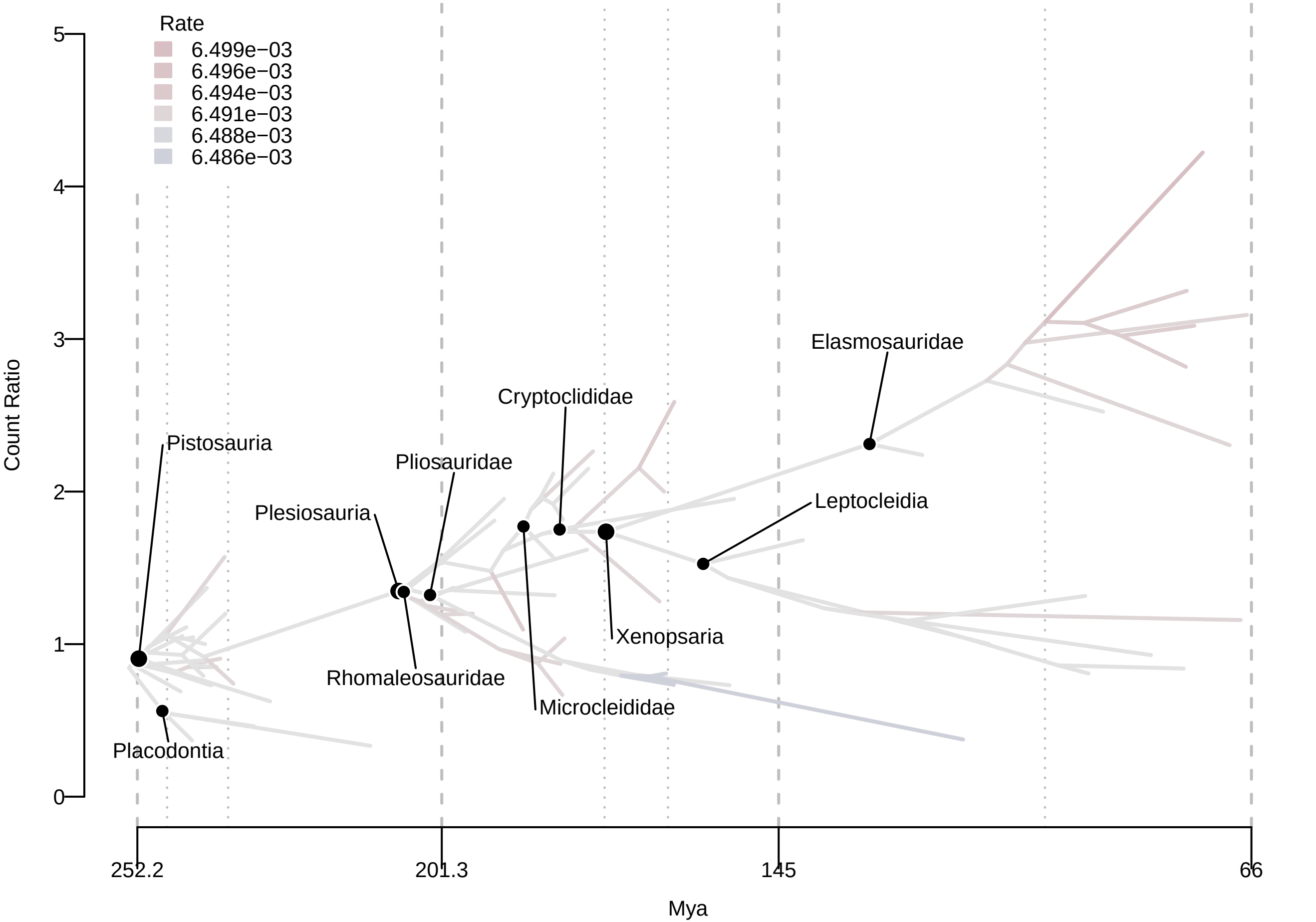

Rate
6.499e−03
6.496e−03
6.494e−03
6.491e−03
6.488e−03
6.486e−03
Count Ratio
0
1
2
3
4
5
Pistosauria
Placodontia
Plesiosauria
Rhomaleosauridae
Pliosauridae
Microcleididae
Cryptoclididae
Xenopsaria
Leptocleidia
Elasmosauridae
252.2
201.3
145
66
Mya

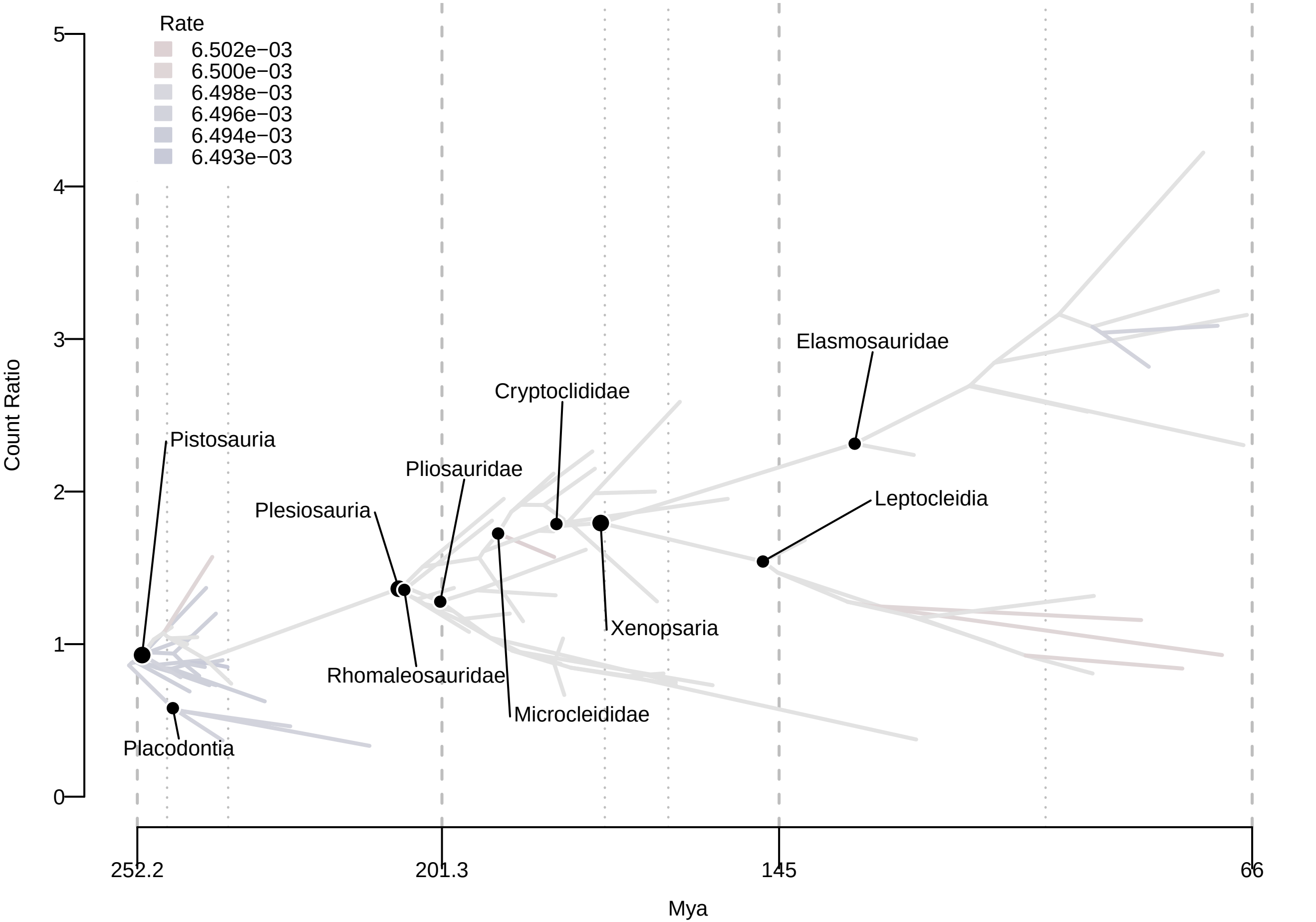

Rate
6.502e−03
6.500e−03
6.498e−03
6.496e−03
6.494e−03
6.493e−03
Count Ratio
0
1
2
3
4
5
Pistosauria
Placodontia
Plesiosauria
Rhomaleosauridae
Pliosauridae
Microcleididae
Cryptoclididae
Xenopsaria
Elasmosauridae
Leptocleidia
252.2
201.3
145
66
Mya

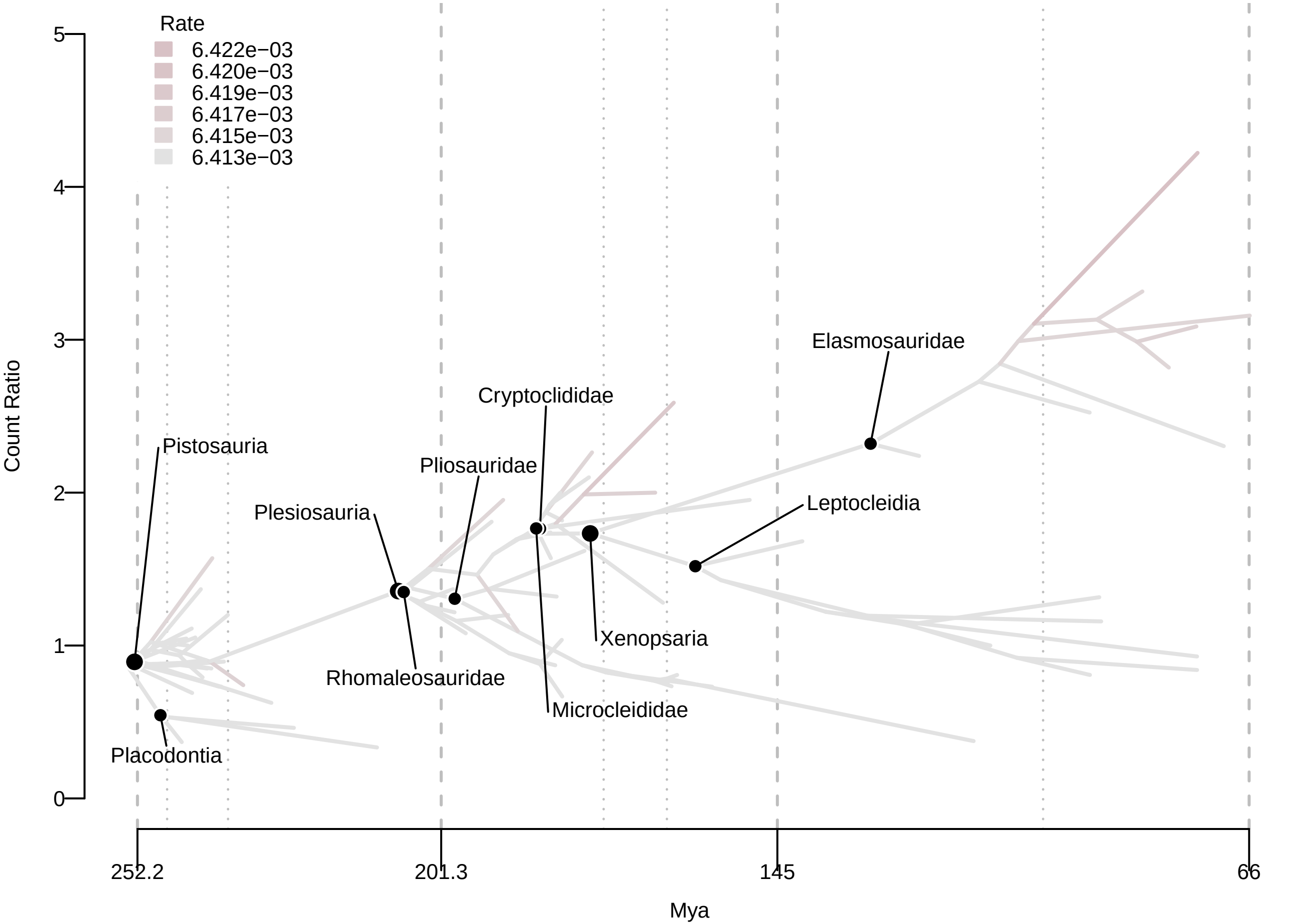

Rate
6.422e−03
6.420e−03
6.419e−03
6.417e−03
6.415e−03
6.413e−03
Count Ratio
0
1
2
3
4
5
Pistosauria
Placodontia
Plesiosauria
Rhomaleosauridae
Pliosauridae
Cryptoclididae
Microcleididae
Xenopsaria
Leptocleidia
Elasmosauridae
252.2
201.3
145
66
Mya

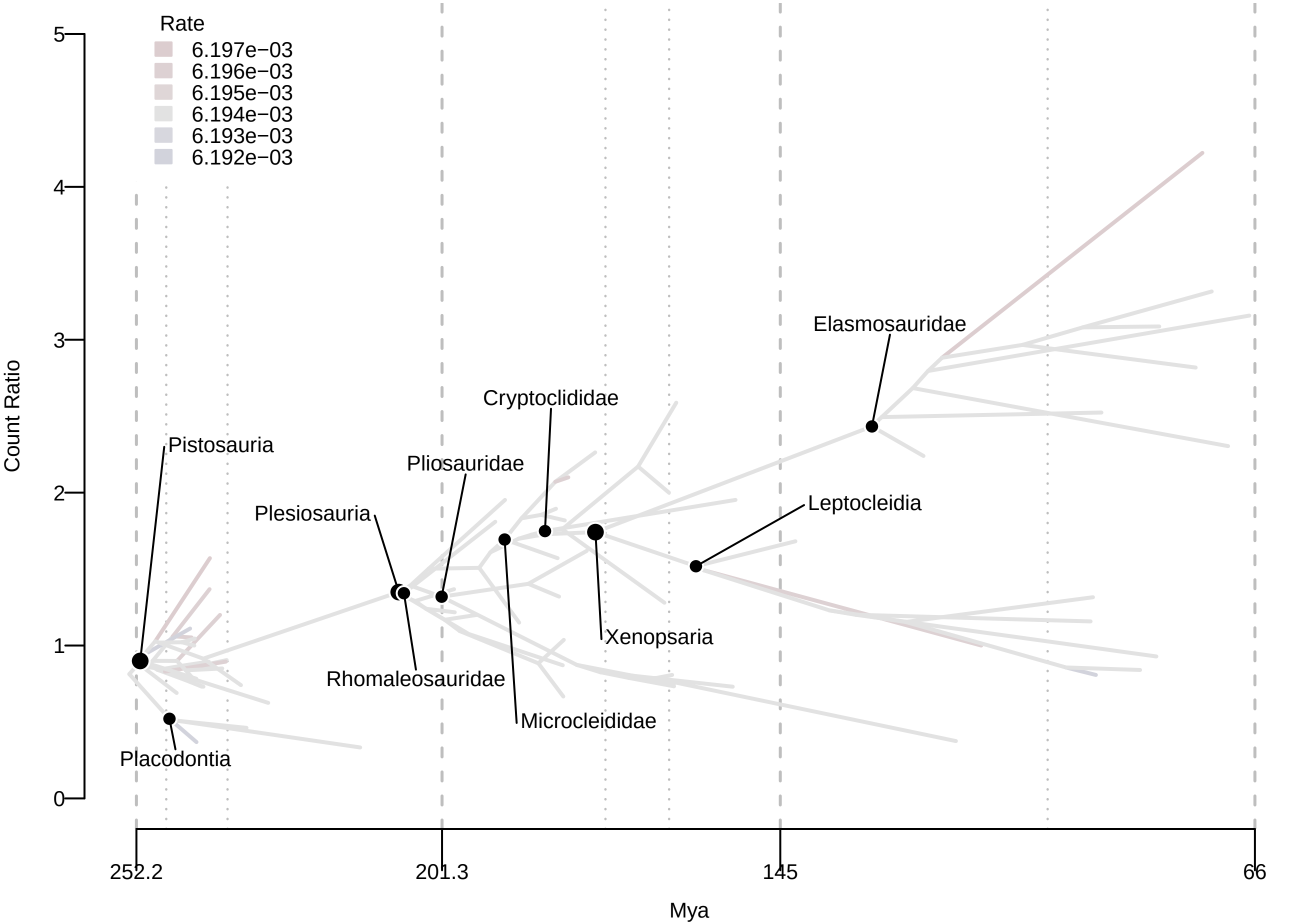

Rate
6.197e−03
6.196e−03
6.195e−03
6.194e−03
6.193e−03
6.192e−03
Count Ratio
0
1
2
3
4
5
Pistosauria
Placodontia
Plesiosauria
Rhomaleosauridae
Pliosauridae
Microcleididae
Cryptoclididae
Xenopsaria
Leptocleidia
Elasmosauridae
252.2
201.3
145
66
Mya

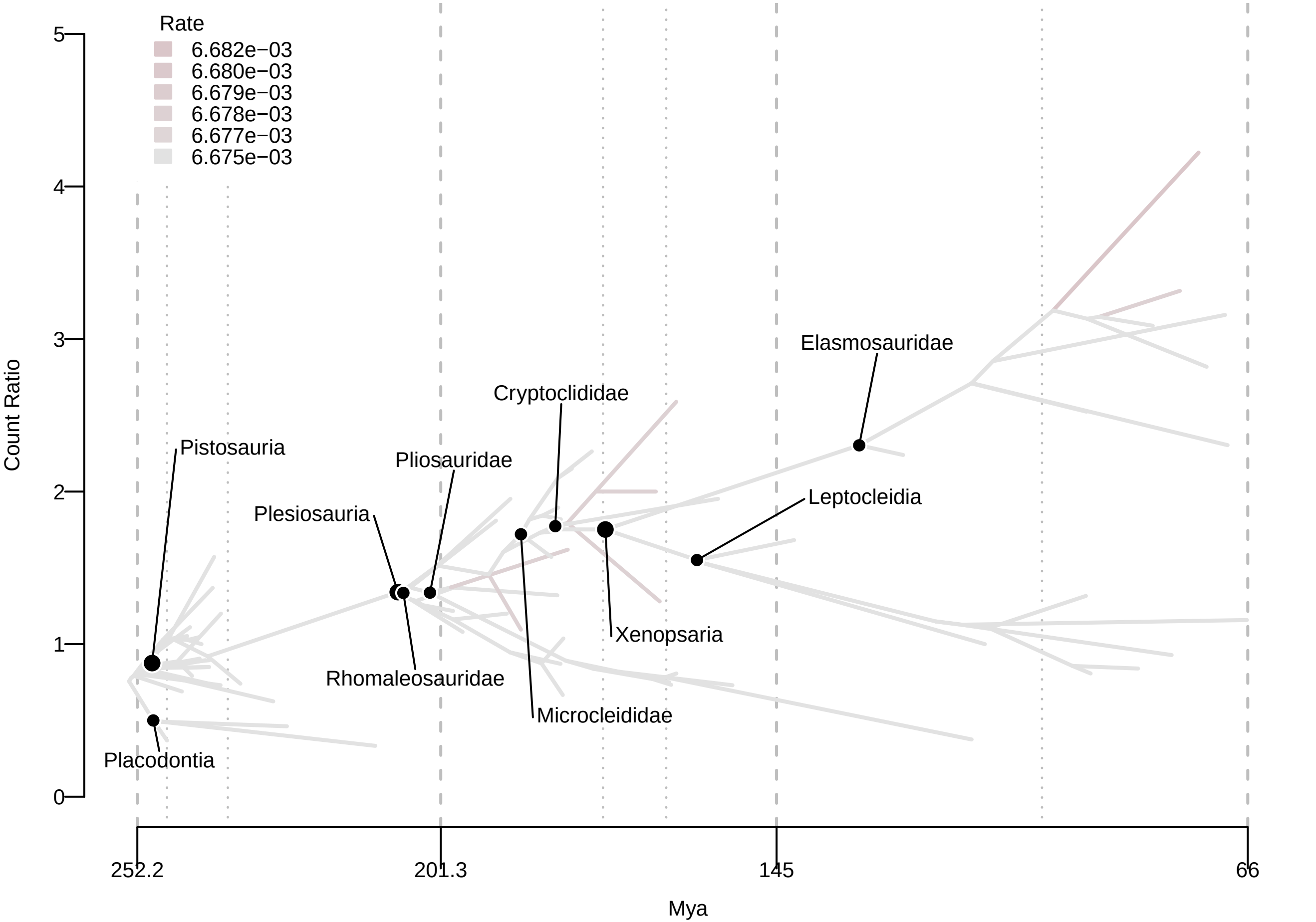
Rate
6.682e−03
6.680e−03
6.679e−03
6.678e−03
6.677e−03
6.675e−03
Count Ratio
0
1
2
3
4
5
Pistosauria
Placodontia
Plesiosauria
Rhomaleosauridae
Pliosauridae
Microcleididae
Cryptoclididae
Xenopsaria
Leptocleidia
Elasmosauridae
252.2
201.3
145
66
Mya

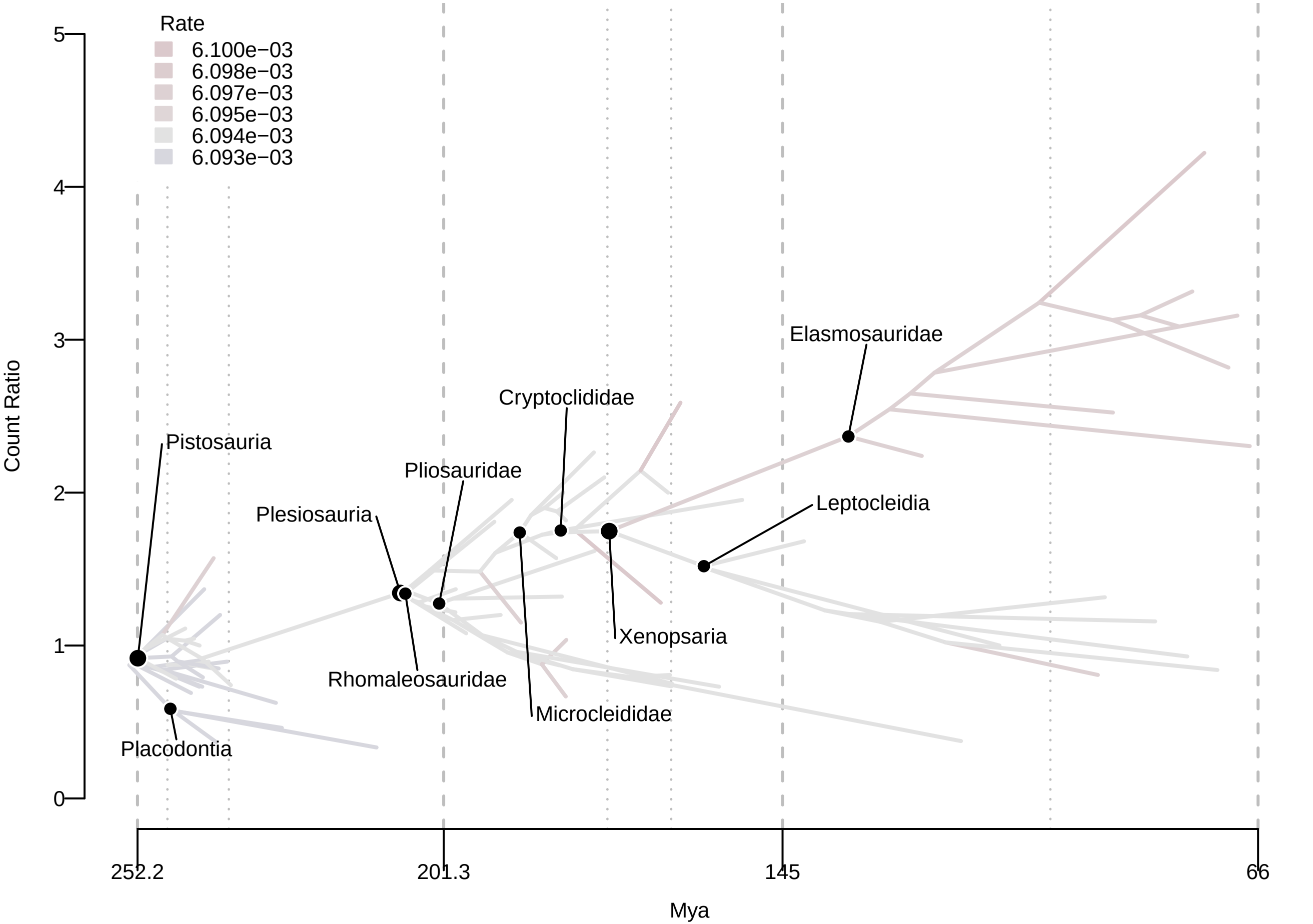

Rate
6.100e−03
6.098e−03
6.097e−03
6.095e−03
6.094e−03
6.093e−03
Count Ratio
0
1
2
3
4
5
Pistosauria
Placodontia
Plesiosauria
Rhomaleosauridae
Pliosauridae
Cryptoclididae
Microcleididae
Xenopsaria
Leptocleidia
Elasmosauridae
252.2
201.3
145
66
Mya

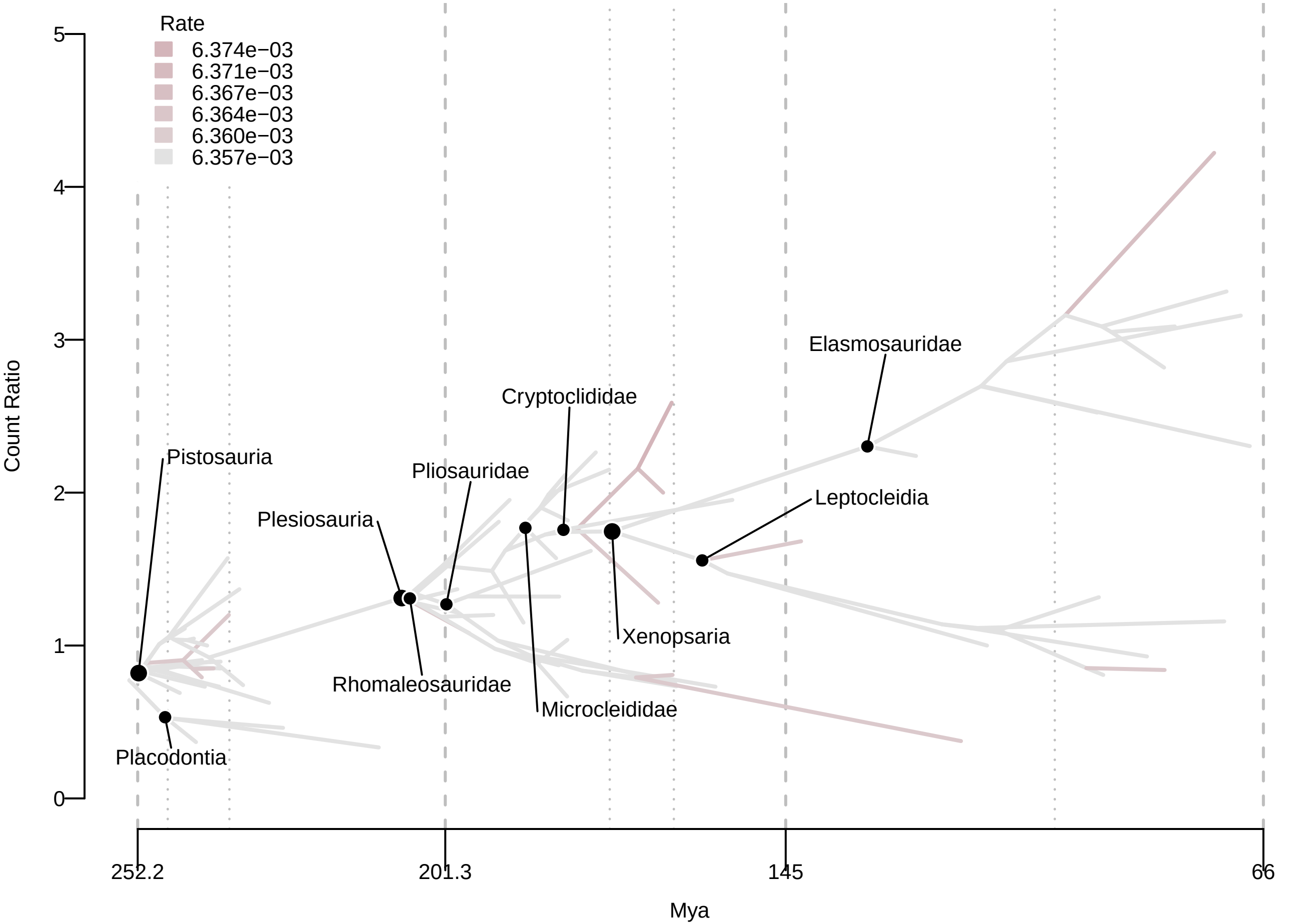

Rate
6.374e−03
6.371e−03
6.367e−03
6.364e−03
6.360e−03
6.357e−03
Count Ratio
0
1
2
3
4
5
Mya
252.2
201.3
145
66
Pistosauria
Placodontia
Plesiosauria
Rhomaleosauridae
Pliosauridae
Microcleididae
Cryptoclididae
Xenopsaria
Leptocleidia
Elasmosauridae

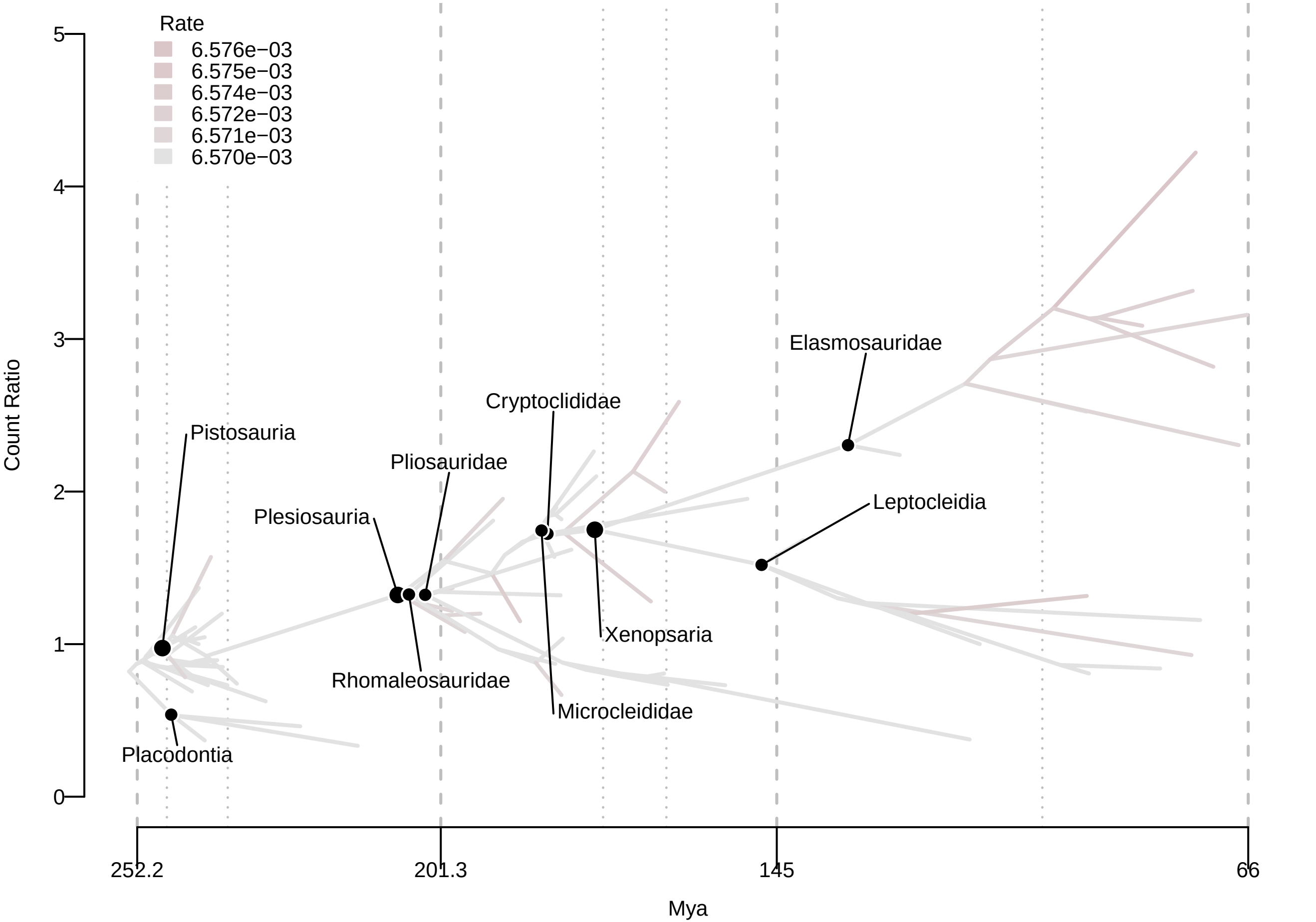

Rate
6.576e−03
6.575e−03
6.574e−03
6.572e−03
6.571e−03
6.570e−03
Count Ratio
0
1
2
3
4
5
Pistosauria
Placodontia
Plesiosauria
Rhomaleosauridae
Pliosauridae
Cryptoclididae
Microcleididae
Xenopsaria
Leptocleidia
Elasmosauridae
252.2
201.3
145
66
Mya

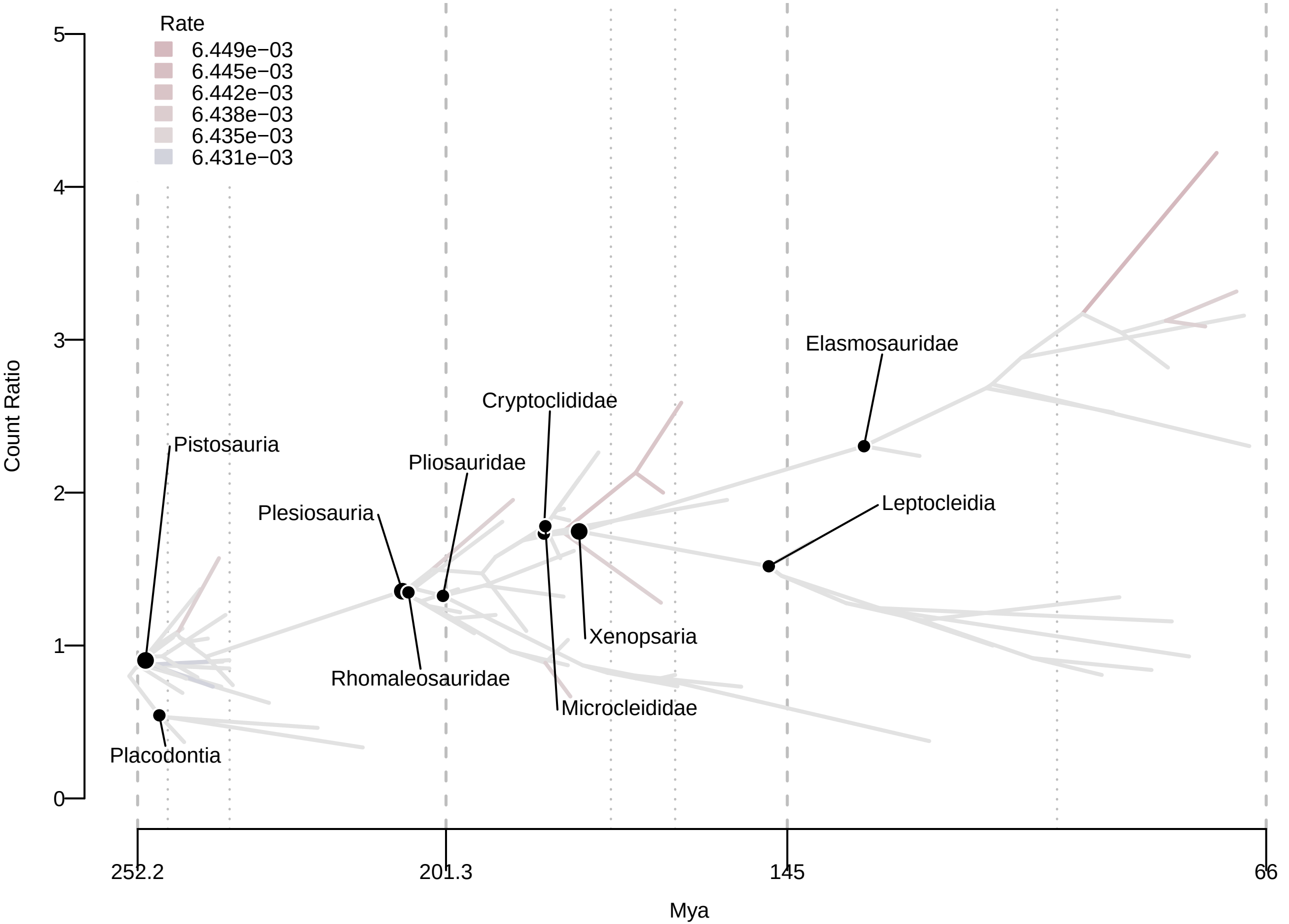

Rate
6.449e−03
6.445e−03
6.442e−03
6.438e−03
6.435e−03
6.431e−03
Count Ratio
0
1
2
3
4
5
Elasmosauridae
Cryptoclididae
Pistosauria
Pliosauridae
Plesiosauria
Leptocleidia
Xenopsaria
Rhomaleosauridae
Microcleididae
Placodontia
252.2
201.3
145
66
Mya

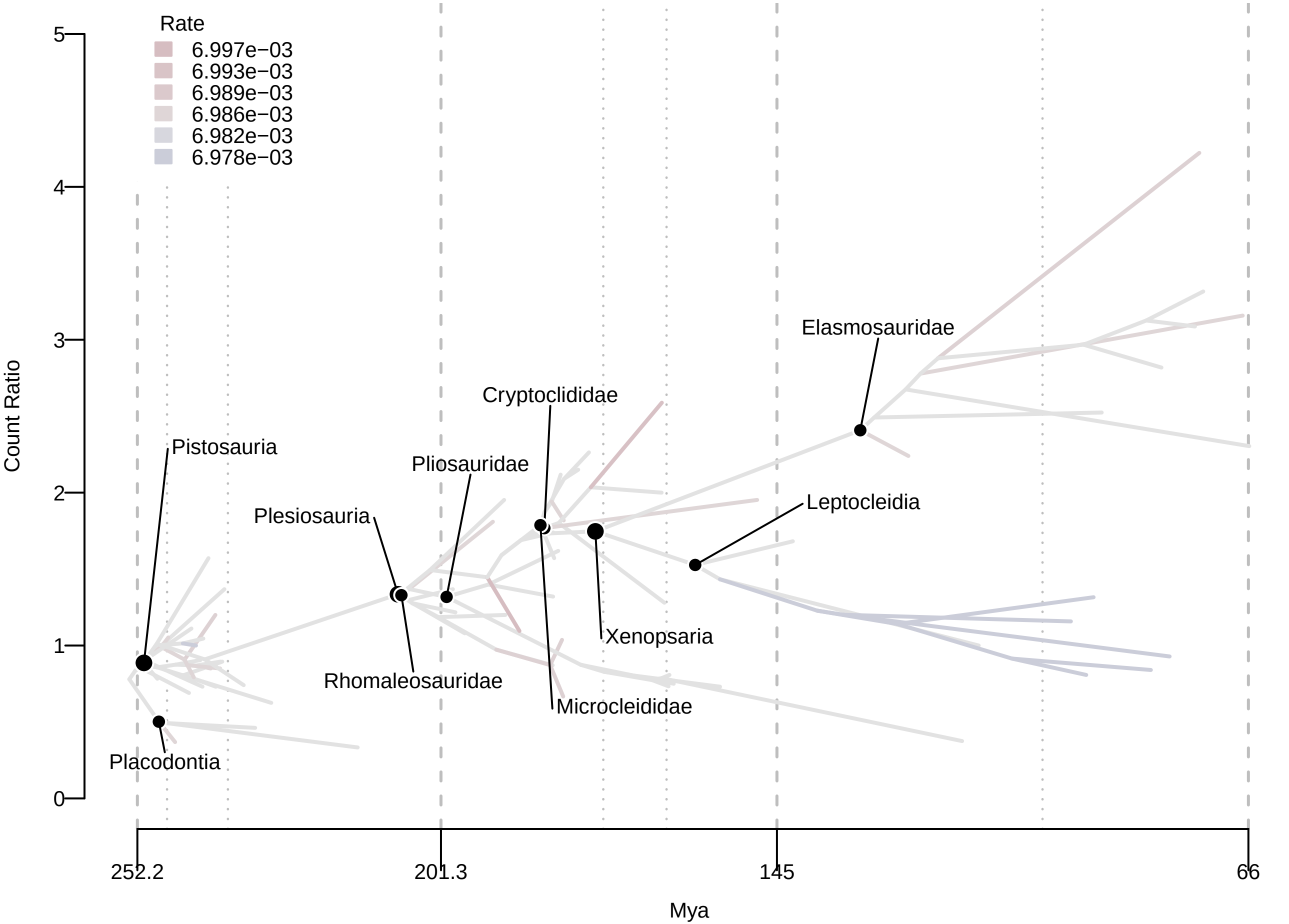

Rate
6.997e−03
6.993e−03
6.989e−03
6.986e−03
6.982e−03
6.978e−03
Count Ratio
0
1
2
3
4
5
Pistosauria
Placodontia
Plesiosauria
Rhomaleosauridae
Pliosauridae
Cryptoclididae
Microcleididae
Xenopsaria
Leptocleidia
Elasmosauridae
252.2
201.3
145
66
Mya

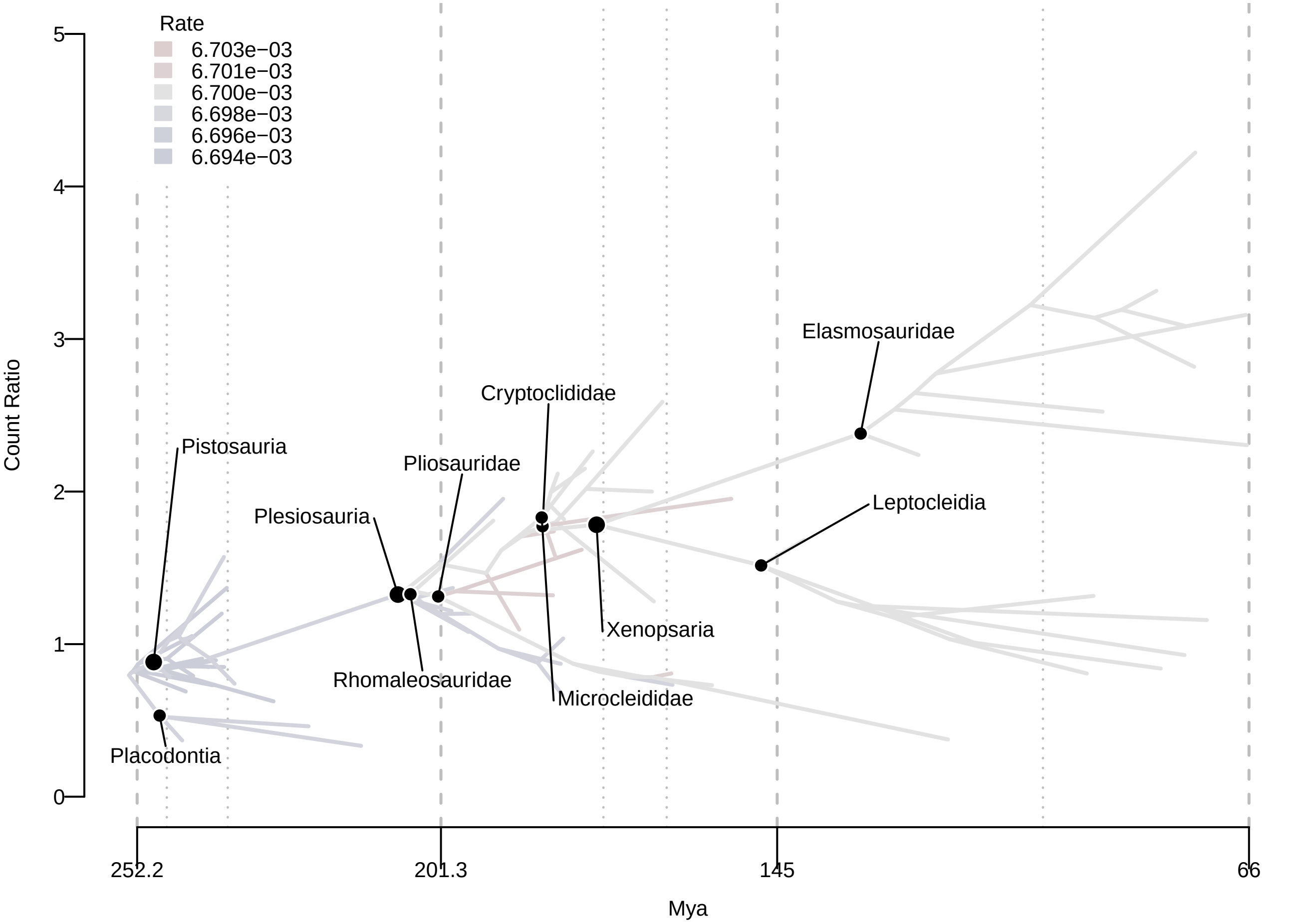

Rate
6.703e−03
6.701e−03
6.700e−03
6.698e−03
6.696e−03
6.694e−03
Count Ratio
0
1
2
3
4
5
Mya
252.2
201.3
145
66
Pistosauria
Placodontia
Plesiosauria
Rhomaleosauridae
Pliosauridae
Cryptoclididae
Microcleididae
Xenopsaria
Leptocleidia
Elasmosauridae

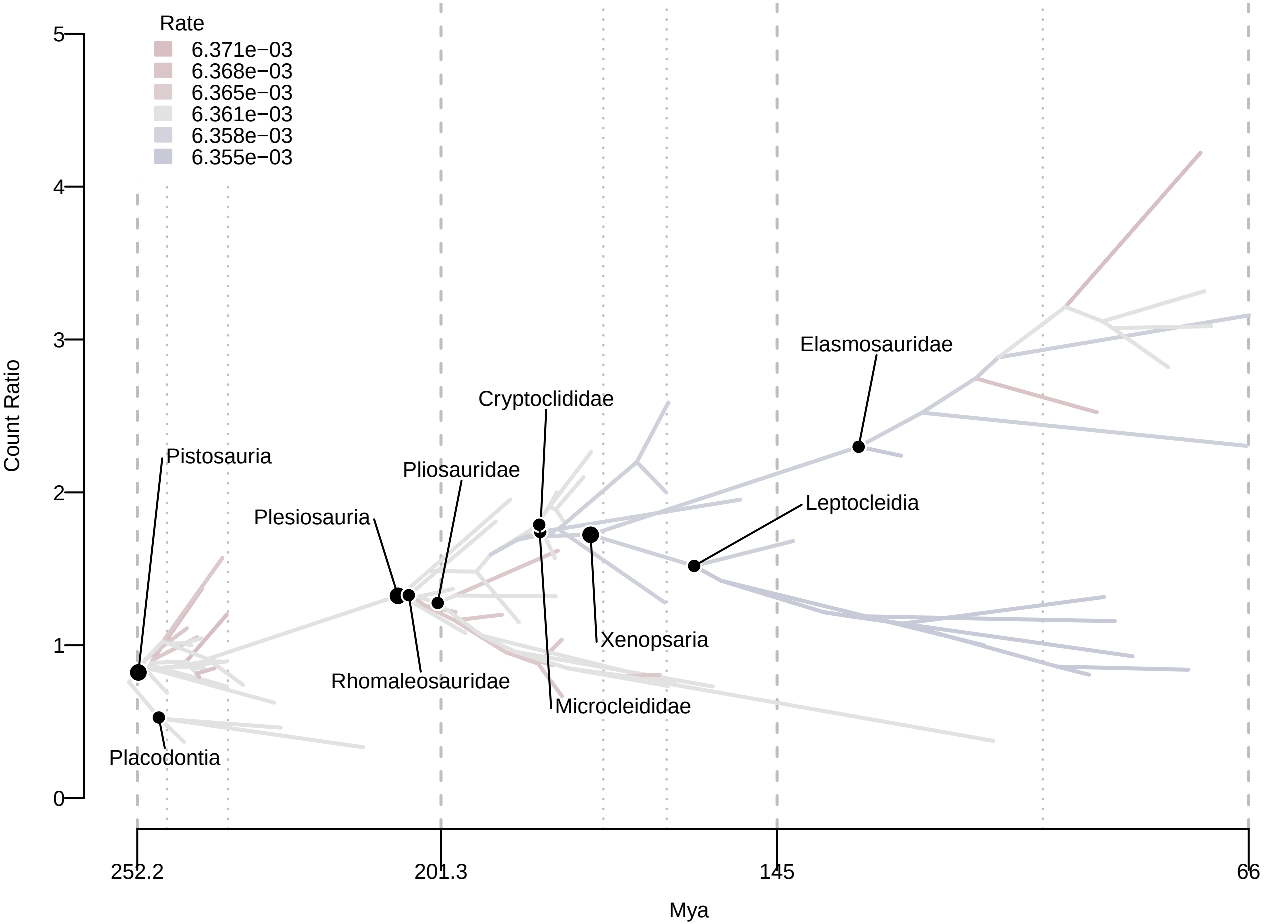

Rate
6.371e−03
6.368e−03
6.365e−03
6.361e−03
6.358e−03
6.355e−03
Count Ratio
0
1
2
3
4
5
Pistosauria
Placodontia
Plesiosauria
Pliosauridae
Rhomaleosauridae
Cryptoclididae
Microcleididae
Xenopsaria
Leptocleidia
Elasmosauridae
252.2
201.3
145
66
Mya

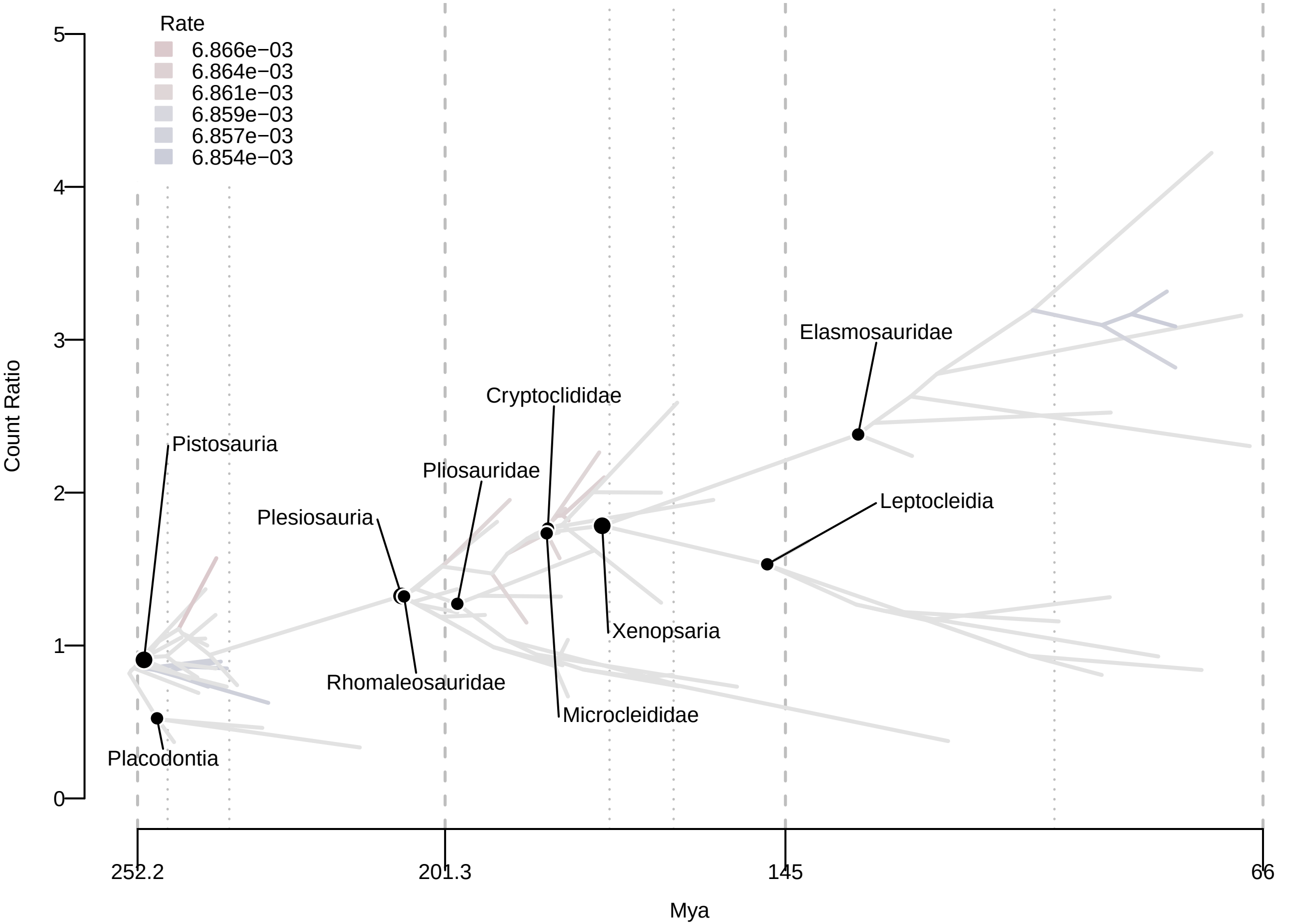

Rate
6.866e−03
6.864e−03
6.861e−03
6.859e−03
6.857e−03
6.854e−03
Count Ratio
5
4
3
2
1
0
Pistosauria
Placodontia
Plesiosauria
Rhomaleosauridae
Pliosauridae
Cryptoclididae
Microcleididae
Xenopsaria
Elasmosauridae
Leptocleidia
252.2
201.3
145
66
Mya

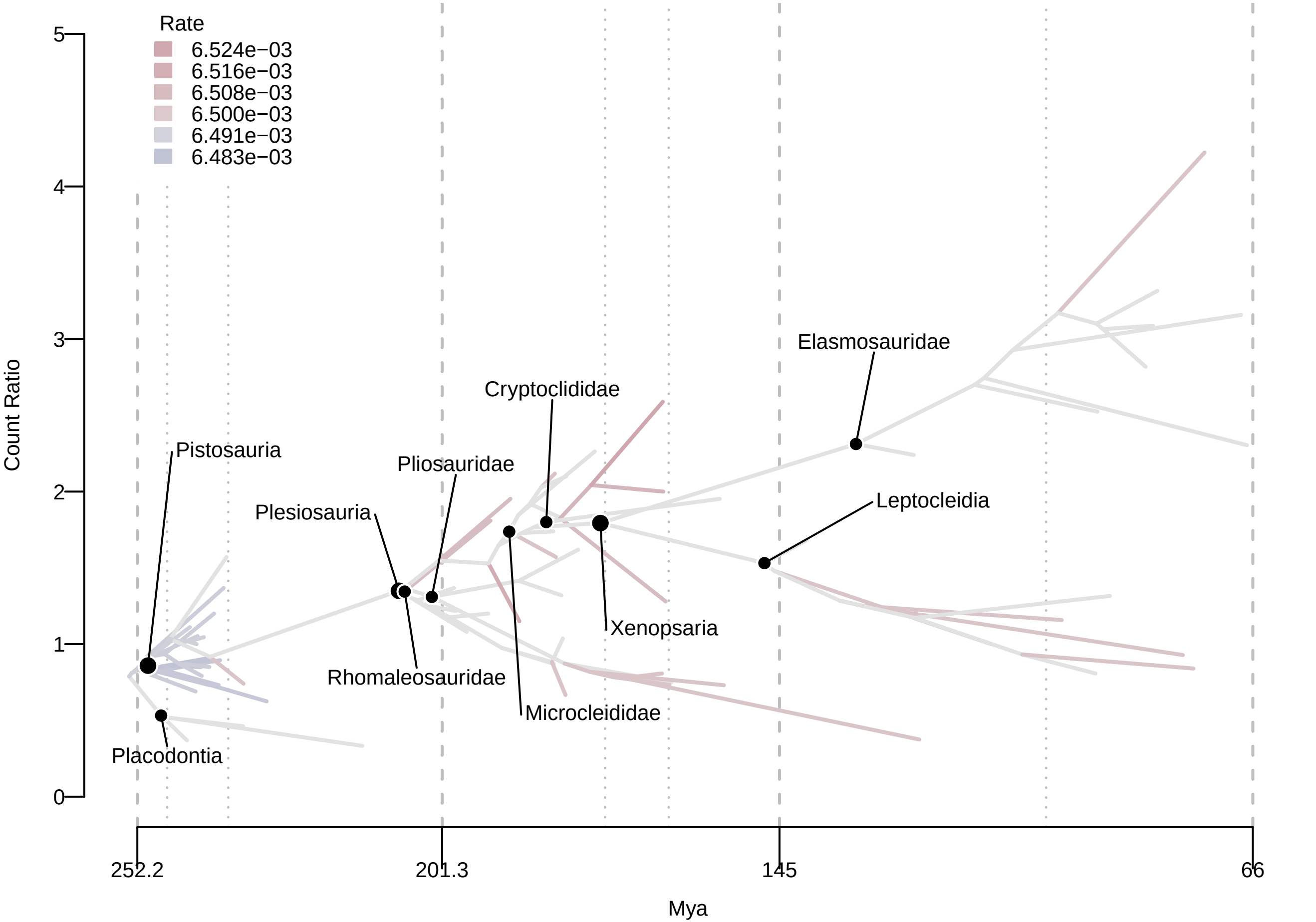
Rate
6.524e−03
6.516e−03
6.508e−03
6.500e−03
6.491e−03
6.483e−03
Count Ratio
0
1
2
3
4
5
Pistosauria
Placodontia
Plesiosauria
Rhomaleosauridae
Pliosauridae
Microcleididae
Cryptoclididae
Xenopsaria
Elasmosauridae
Leptocleidia
252.2
201.3
145
66
Mya

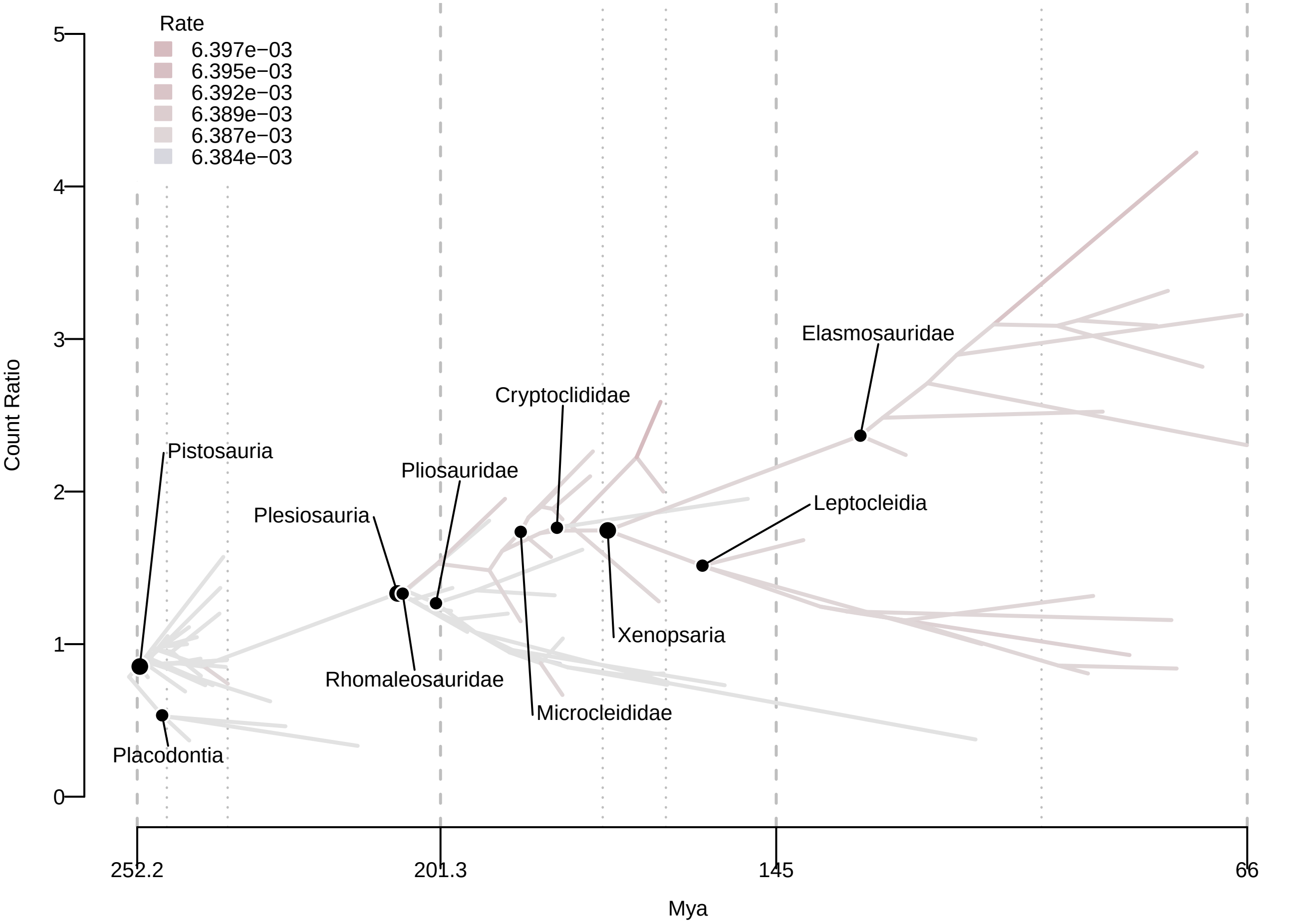

Rate
6.397e−03
6.395e−03
6.392e−03
6.389e−03
6.387e−03
6.384e−03
Count Ratio
0
1
2
3
4
5
Pistosauria
Placodontia
Plesiosauria
Pliosauridae
Rhomaleosauridae
Cryptoclididae
Microcleididae
Xenopsaria
Leptocleidia
Elasmosauridae
252.2
201.3
145
66
Mya

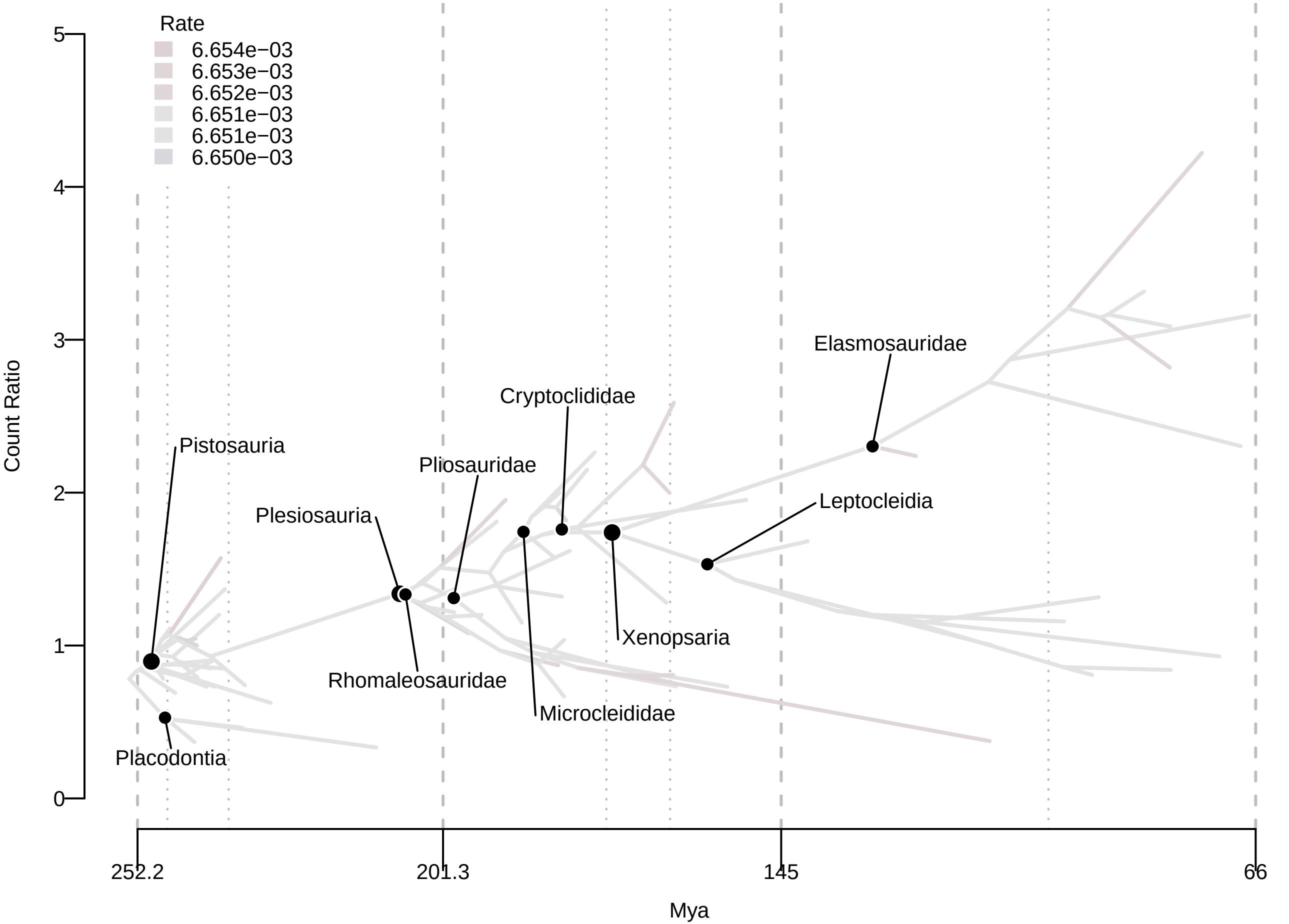

Rate
6.654e−03
6.653e−03
6.652e−03
6.651e−03
6.651e−03
6.650e−03
Count Ratio
5
4
3
2
1
0
Pistosauria
Placodontia
Plesiosauria
Rhomaleosauridae
Pliosauridae
Microcleididae
Cryptoclididae
Xenopsaria
Leptocleidia
Elasmosauridae
252.2
201.3
145
66
Mya

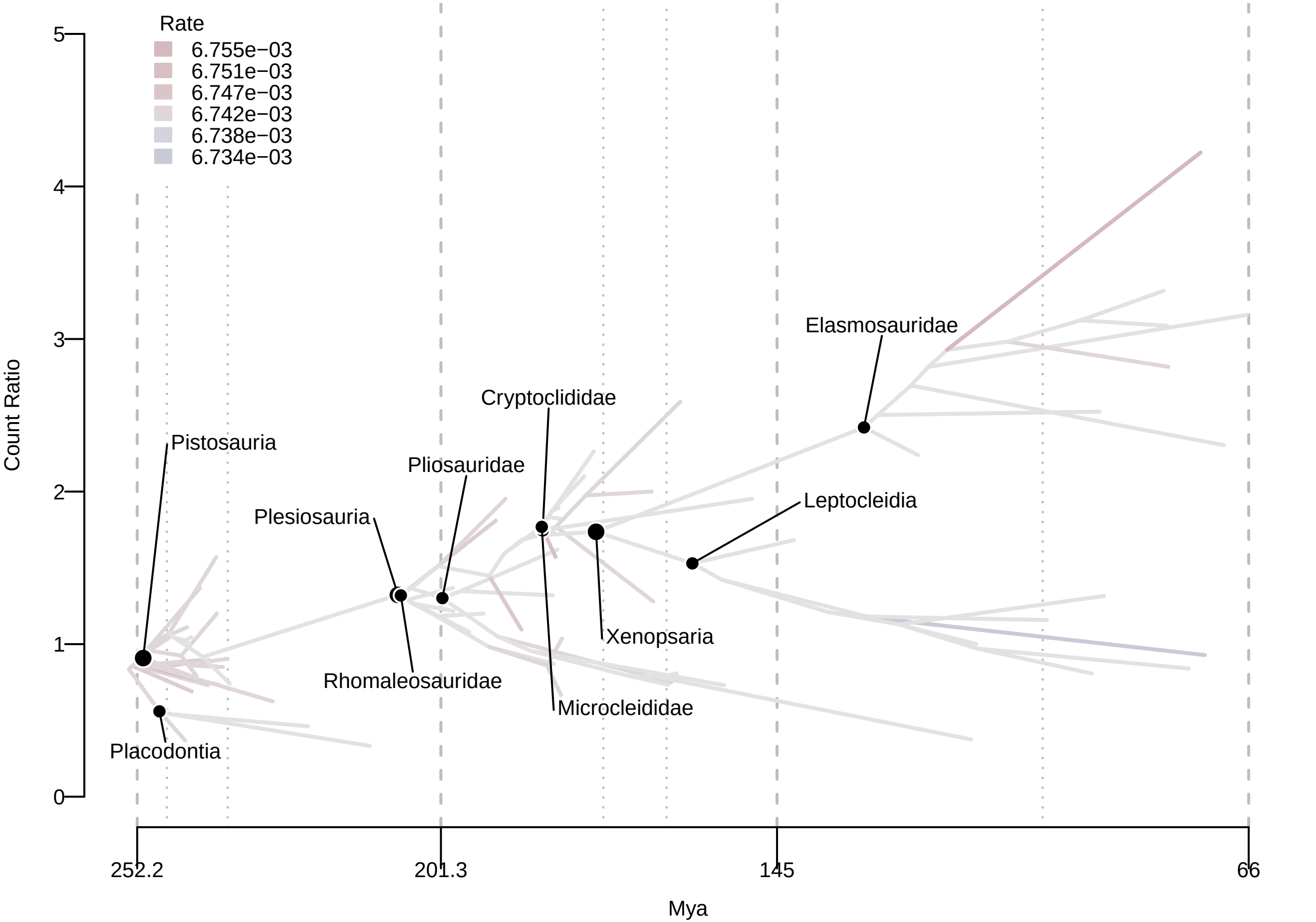

Rate
6.755e−03
6.751e−03
6.747e−03
6.742e−03
6.738e−03
6.734e−03
Count Ratio
0
1
2
3
4
5
Pistosauria
Placodontia
Plesiosauria
Rhomaleosauridae
Pliosauridae
Cryptoclididae
Microcleididae
Xenopsaria
Leptocleidia
Elasmosauridae
252.2
201.3
145
66
Mya

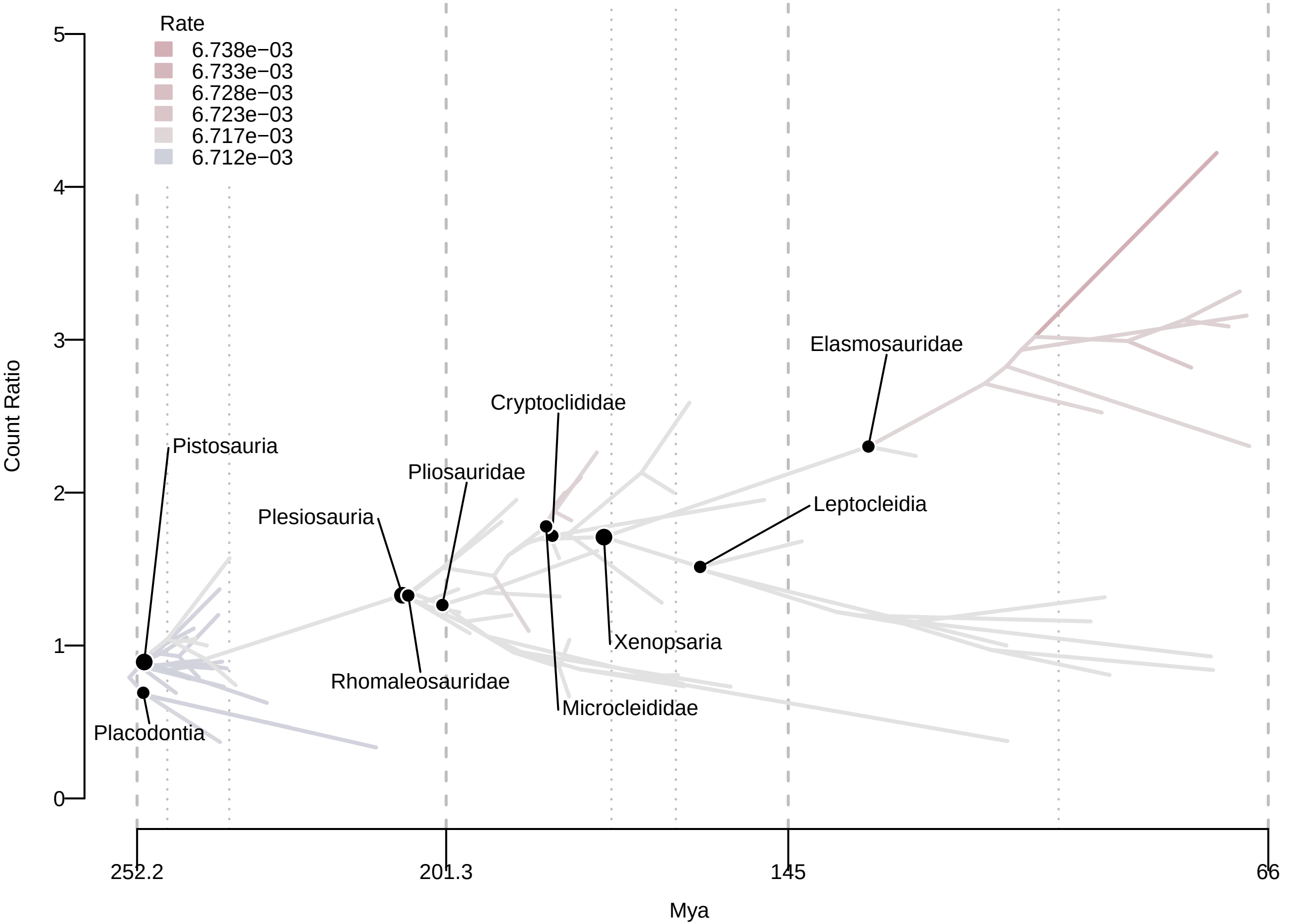

Rate
6.738e−03
6.733e−03
6.728e−03
6.723e−03
6.717e−03
6.712e−03
Count Ratio
0
1
2
3
4
5
Pistosauria
Placodontia
Plesiosauria
Pliosauridae
Rhomaleosauridae
Cryptoclididae
Microcleididae
Xenopsaria
Leptocleidia
Elasmosauridae
252.2
201.3
145
66
Mya

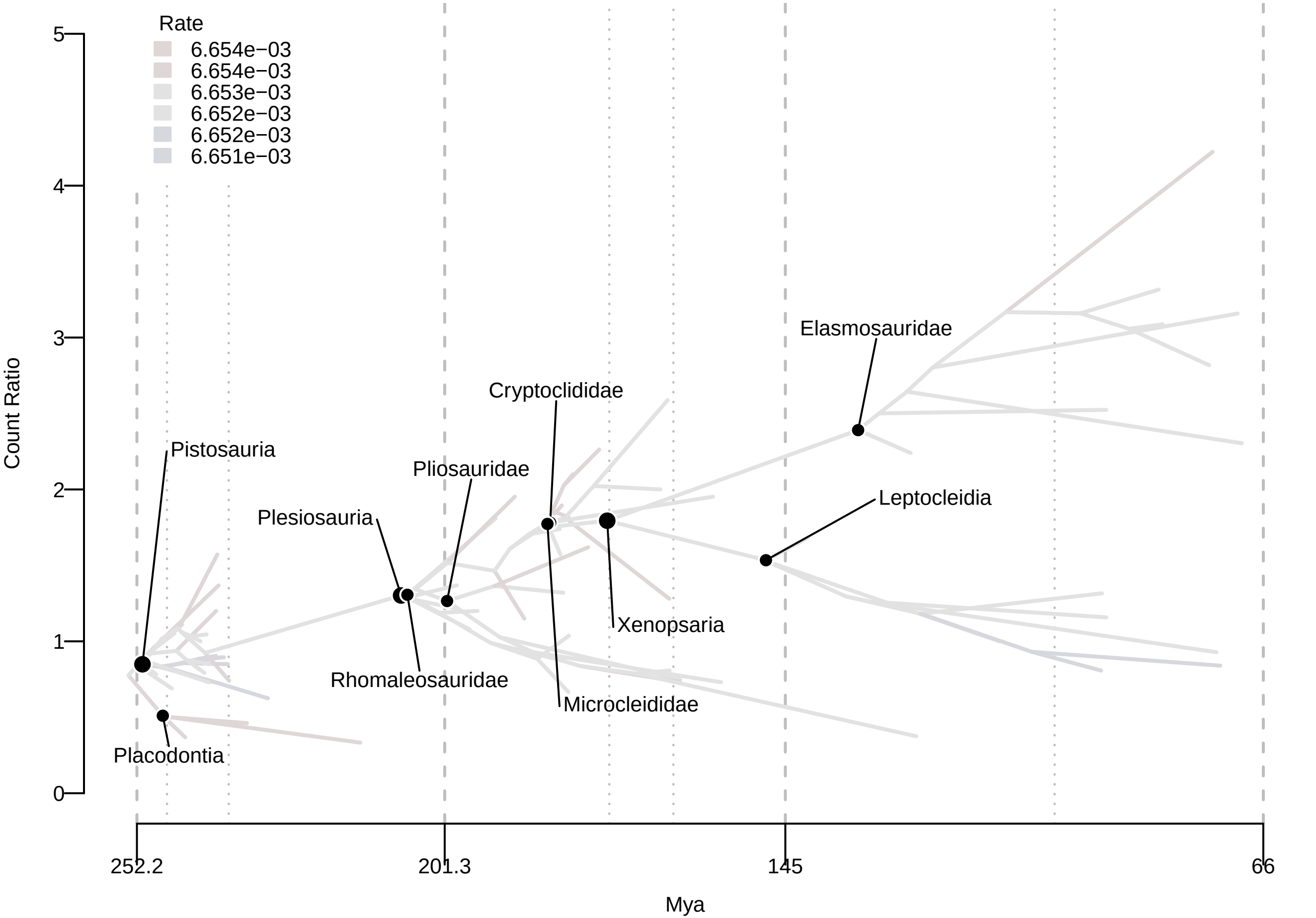

Rate
6.654e−03
6.654e−03
6.653e−03
6.652e−03
6.652e−03
6.651e−03
Count Ratio
0
1
2
3
4
5
Pistosauria
Placodontia
Plesiosauria
Rhomaleosauridae
Pliosauridae
Cryptoclididae
Microcleididae
Xenopsaria
Elasmosauridae
Leptocleidia
252.2
201.3
145
66
Mya

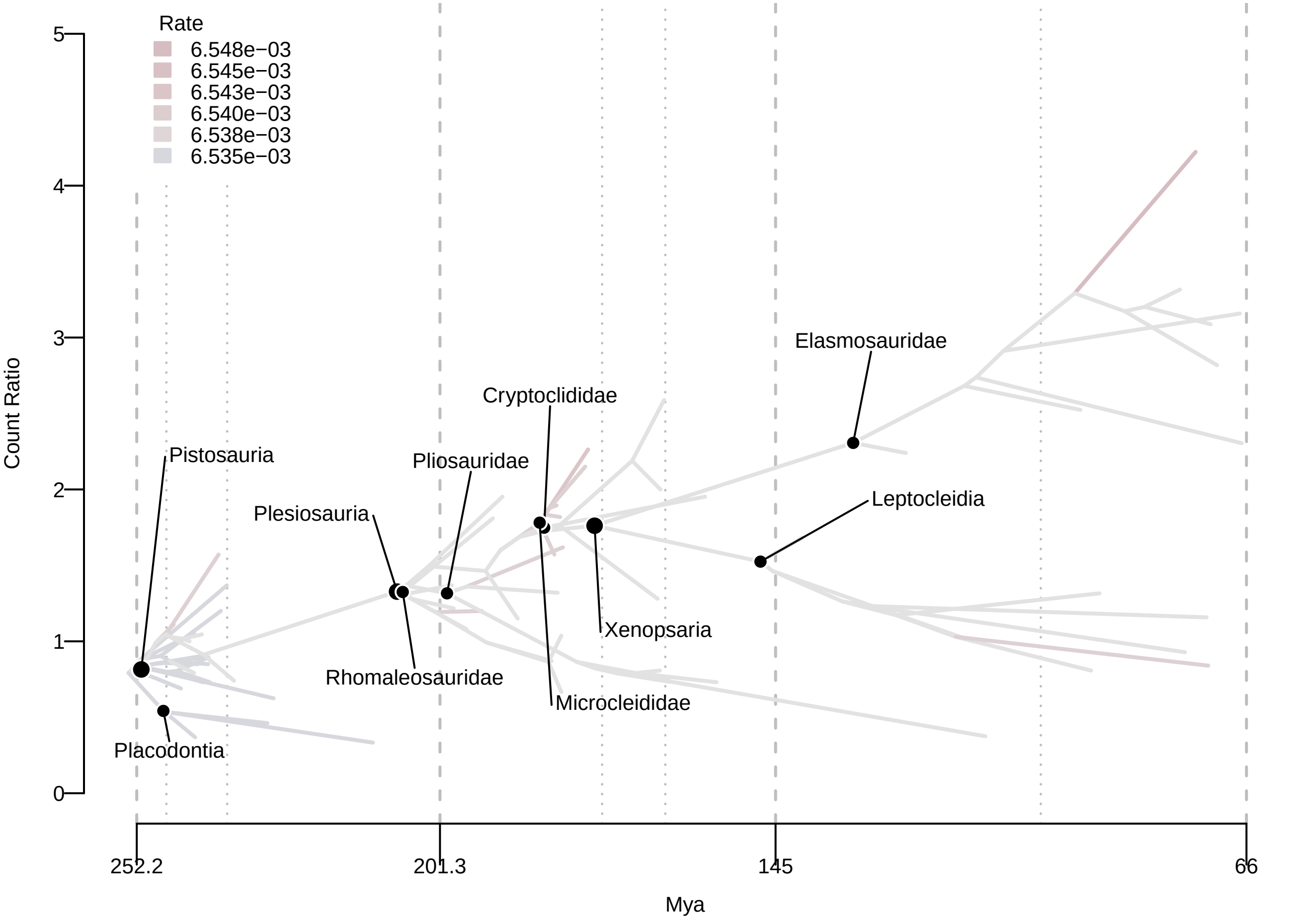

Rate
6.548e−03
6.545e−03
6.543e−03
6.540e−03
6.538e−03
6.535e−03
Count Ratio
0
1
2
3
4
5
Pistosauria
Placodontia
Plesiosauria
Rhomaleosauridae
Pliosauridae
Cryptoclididae
Microcleididae
Xenopsaria
Elasmosauridae
Leptocleidia
252.2
201.3
145
66
Mya

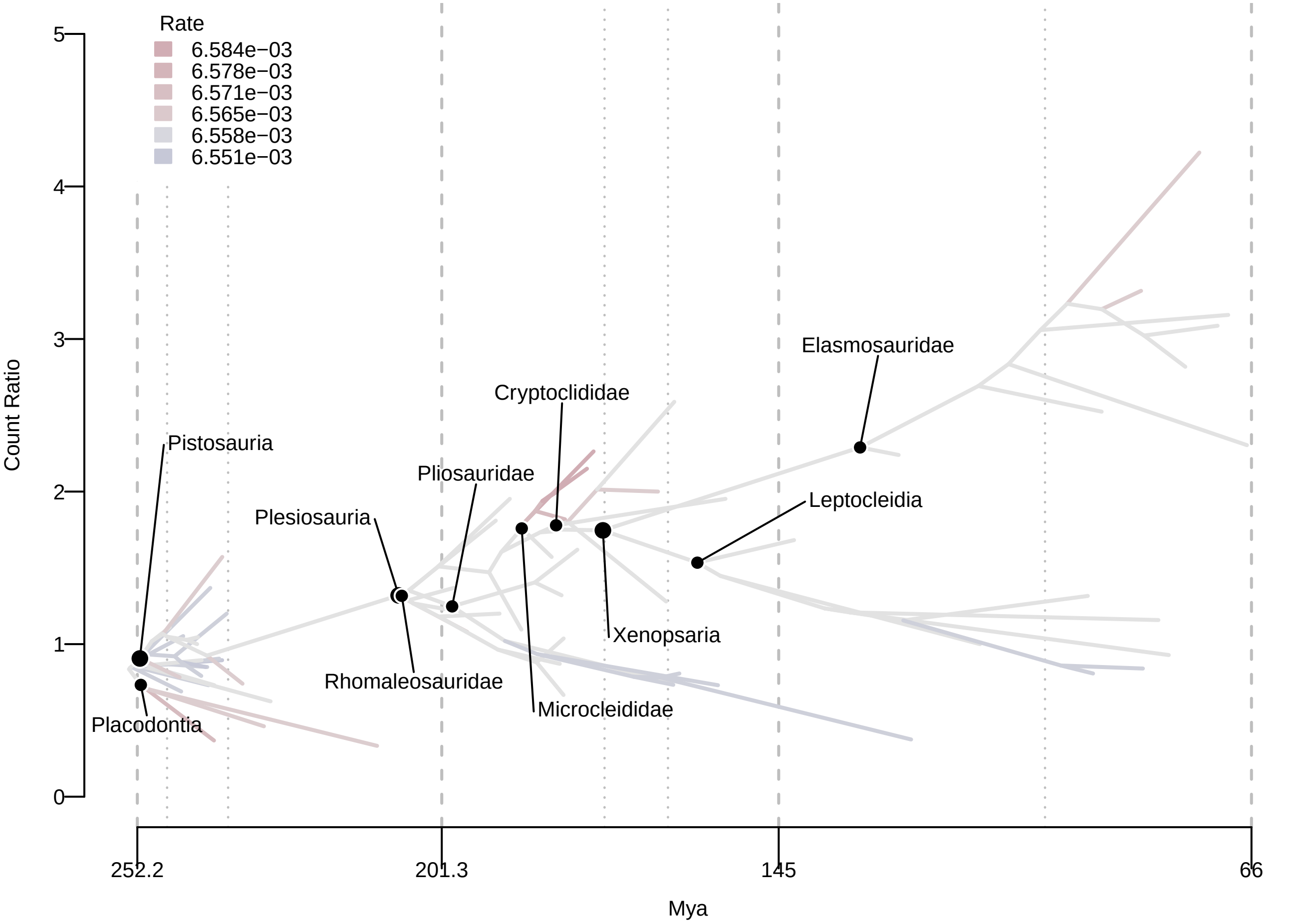

Rate
6.584e−03
6.578e−03
6.571e−03
6.565e−03
6.558e−03
6.551e−03
Count Ratio
0
1
2
3
4
5
Pistosauria
Placodontia
Plesiosauria
Rhomaleosauridae
Pliosauridae
Microcleididae
Cryptoclididae
Xenopsaria
Leptocleidia
Elasmosauridae
252.2
201.3
145
66
Mya

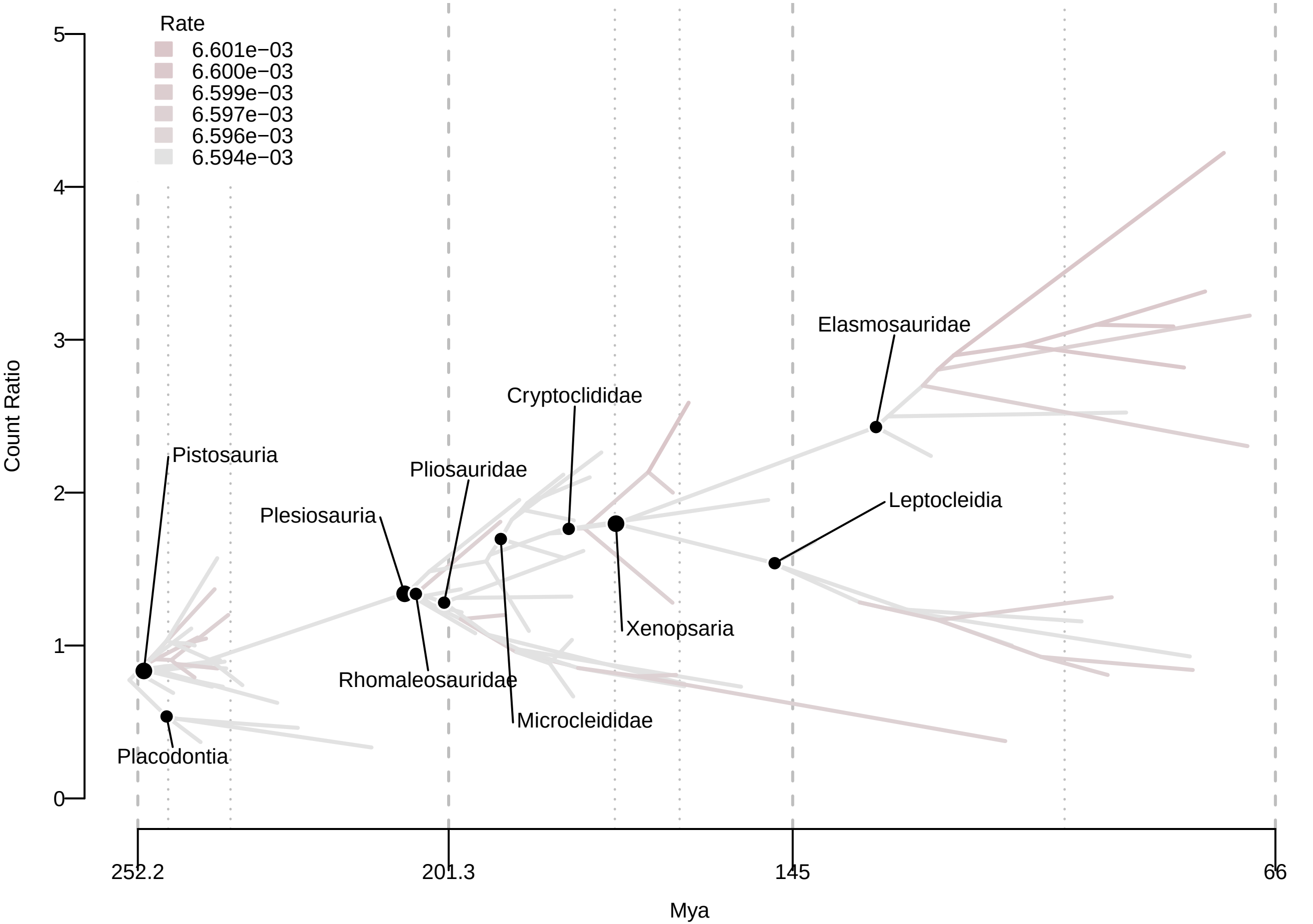

Rate
6.601e−03
6.600e−03
6.599e−03
6.597e−03
6.596e−03
6.594e−03
Count Ratio
0
1
2
3
4
5
Elasmosauridae
Cryptoclididae
Pistosauria
Pliosauridae
Plesiosauria
Leptocleidia
Xenopsaria
Rhomaleosauridae
Microcleididae
Placodontia
252.2
201.3
145
66
Mya

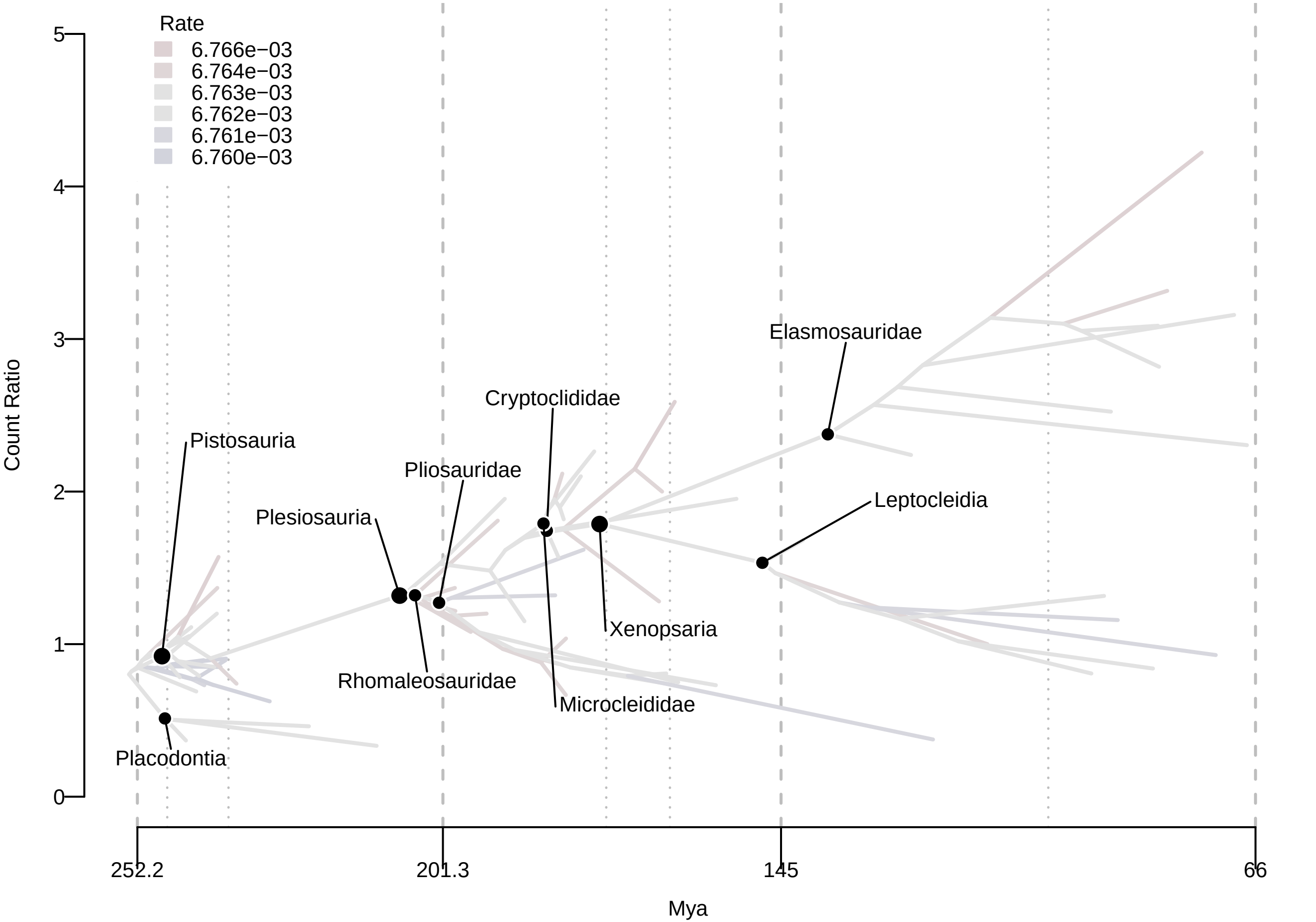

Rate
6.766e−03
6.764e−03
6.763e−03
6.762e−03
6.761e−03
6.760e−03
Count Ratio
0
1
2
3
4
5
Pistosauria
Placodontia
Plesiosauria
Rhomaleosauridae
Pliosauridae
Cryptoclididae
Microcleididae
Xenopsaria
Elasmosauridae
Leptocleidia
252.2
201.3
145
66
Mya

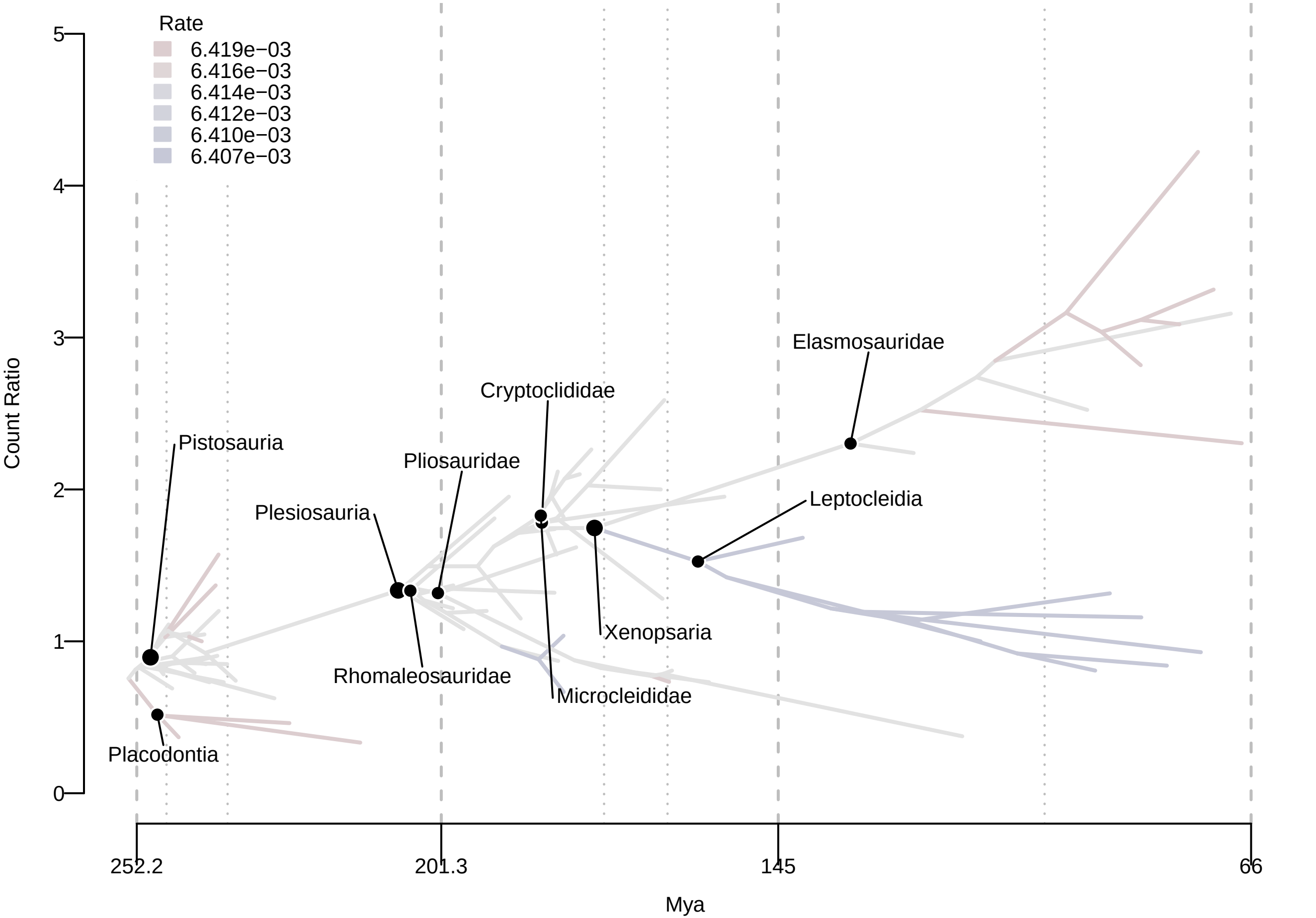
Rate
6.419e−03
6.416e−03
6.414e−03
6.412e−03
6.410e−03
6.407e−03
Count Ratio
0
1
2
3
4
5
Pistosauria
Plesiosauria
Pliosauridae
Cryptoclididae
Elasmosauridae
Leptocleidia
Xenopsaria
Microcleididae
Rhomaleosauridae
Placodontia
252.2
201.3
145
66
Mya

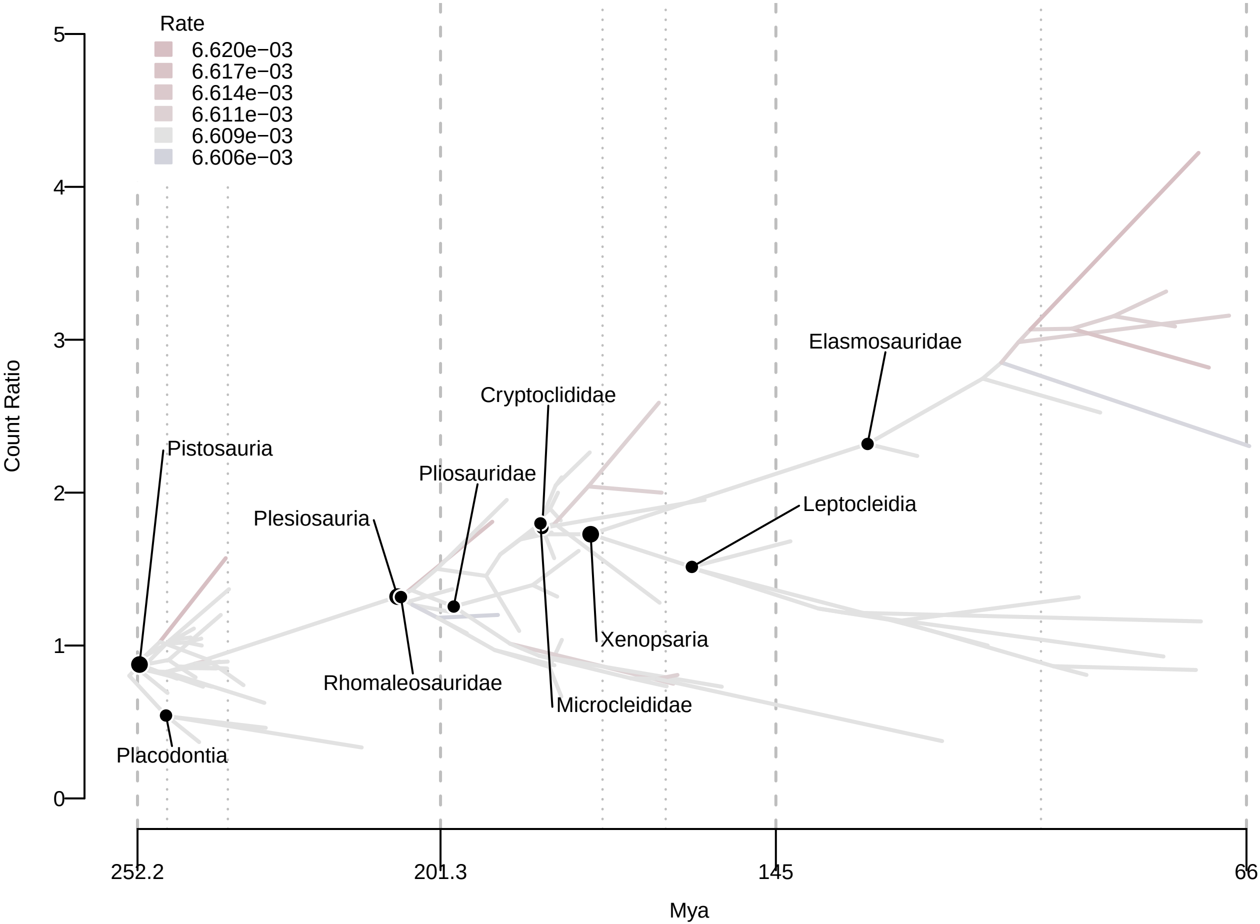

Rate
6.620e−03
6.617e−03
6.614e−03
6.611e−03
6.609e−03
6.606e−03
Count Ratio
5
4
3
2
1
0
Pistosauria
Plesiosauria
Pliosauridae
Cryptoclididae
Elasmosauridae
Leptocleidia
Xenopsaria
Microcleididae
Rhomaleosauridae
Placodontia
252.2
201.3
145
66
Mya

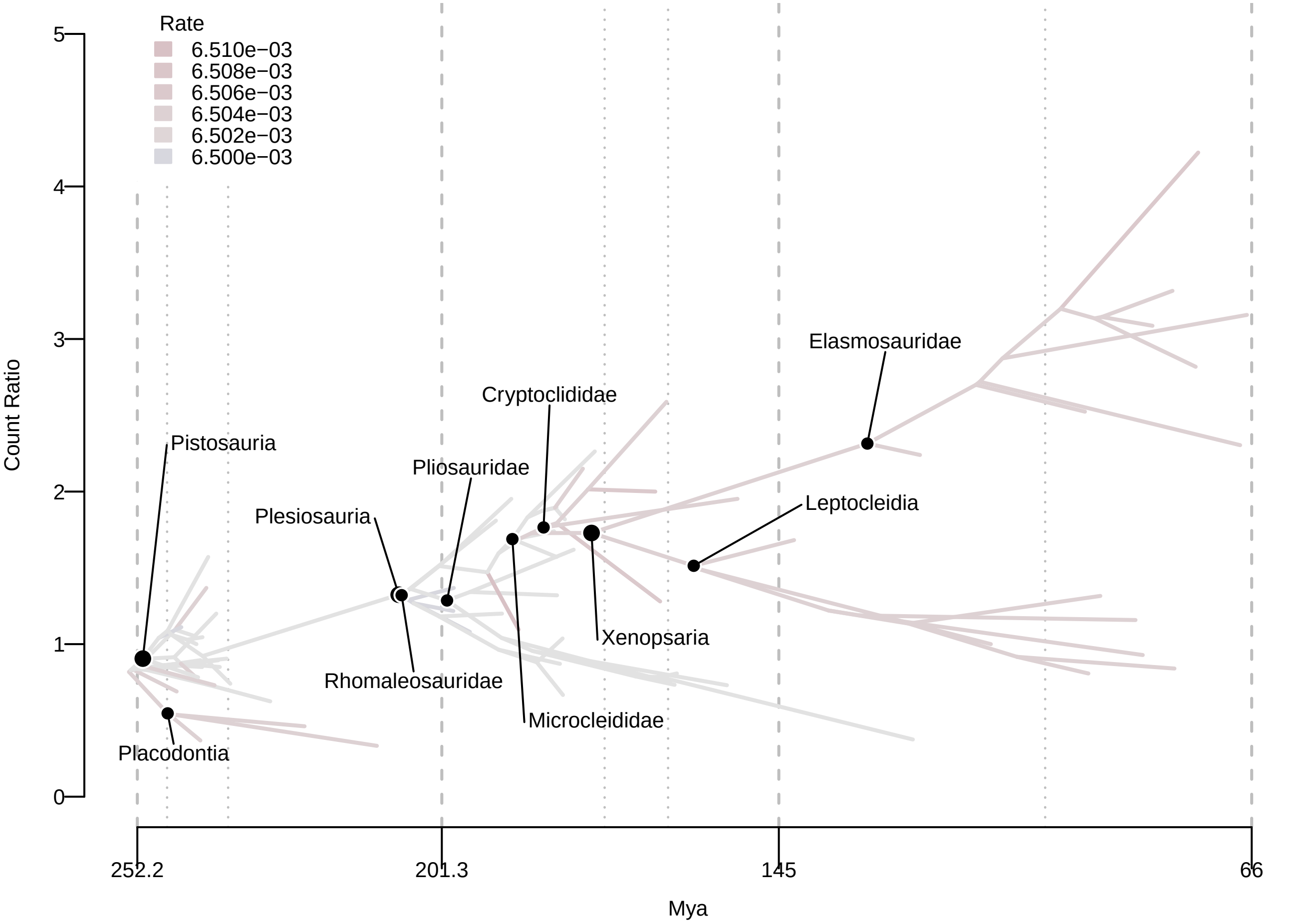

Rate
6.510e−03
6.508e−03
6.506e−03
6.504e−03
6.502e−03
6.500e−03
Count Ratio
0
1
2
3
4
5
Pistosauria
Placodontia
Plesiosauria
Rhomaleosauridae
Pliosauridae
Cryptoclididae
Microcleididae
Xenopsaria
Leptocleidia
Elasmosauridae
252.2
201.3
145
66
Mya

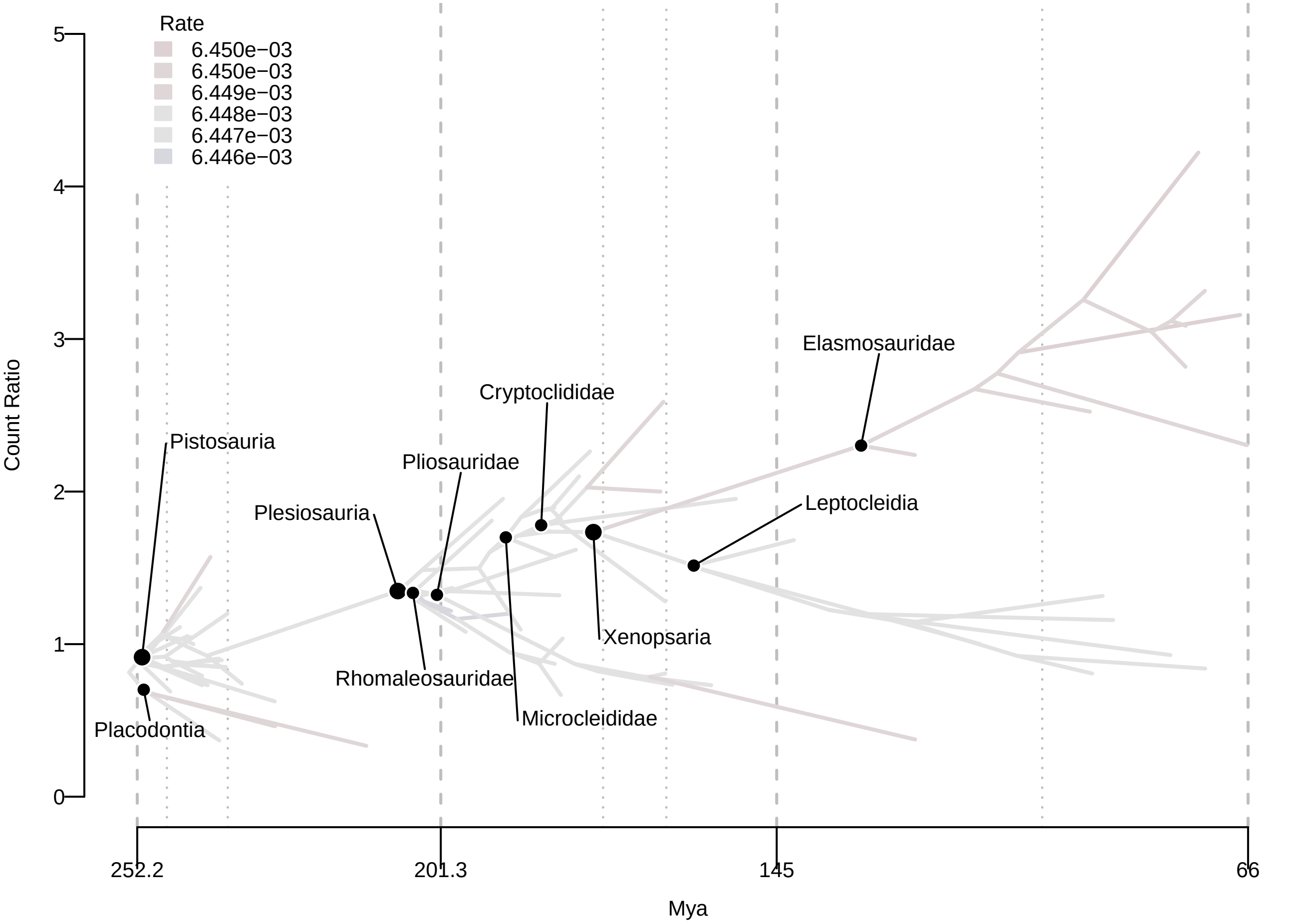
Rate
6.450e−03
6.450e−03
6.449e−03
6.448e−03
6.447e−03
6.446e−03
Count Ratio
0
1
2
3
4
5
Pistosauria
Placodontia
Plesiosauria
Rhomaleosauridae
Pliosauridae
Microcleididae
Cryptoclididae
Xenopsaria
Leptocleidia
Elasmosauridae
252.2
201.3
145
66
Mya

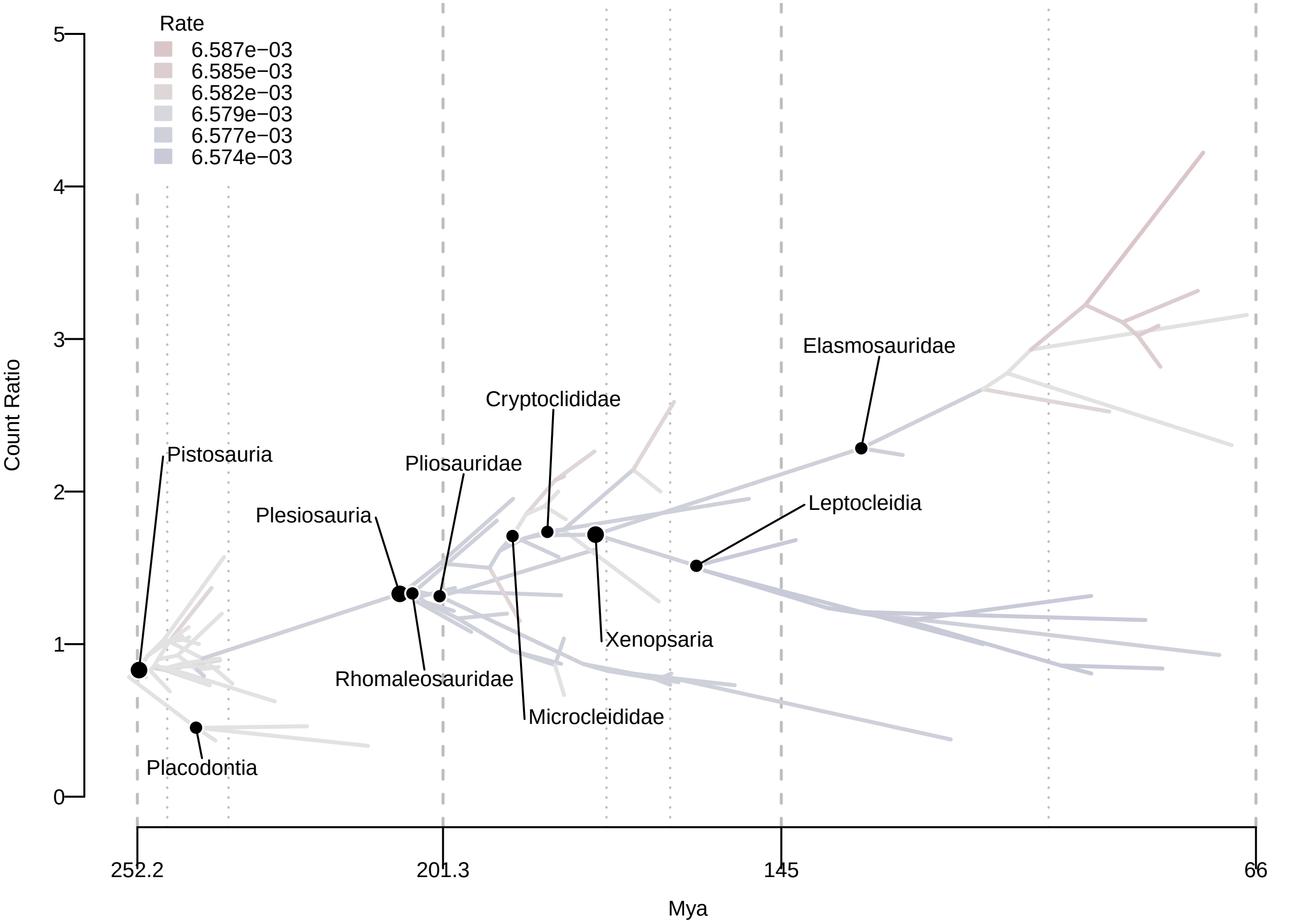

Rate
6.587e−03
6.585e−03
6.582e−03
6.579e−03
6.577e−03
6.574e−03
Count Ratio
0
1
2
3
4
5
Pistosauria
Placodontia
Plesiosauria
Rhomaleosauridae
Pliosauridae
Microcleididae
Cryptoclididae
Xenopsaria
Leptocleidia
Elasmosauridae
252.2
201.3
145
66
Mya

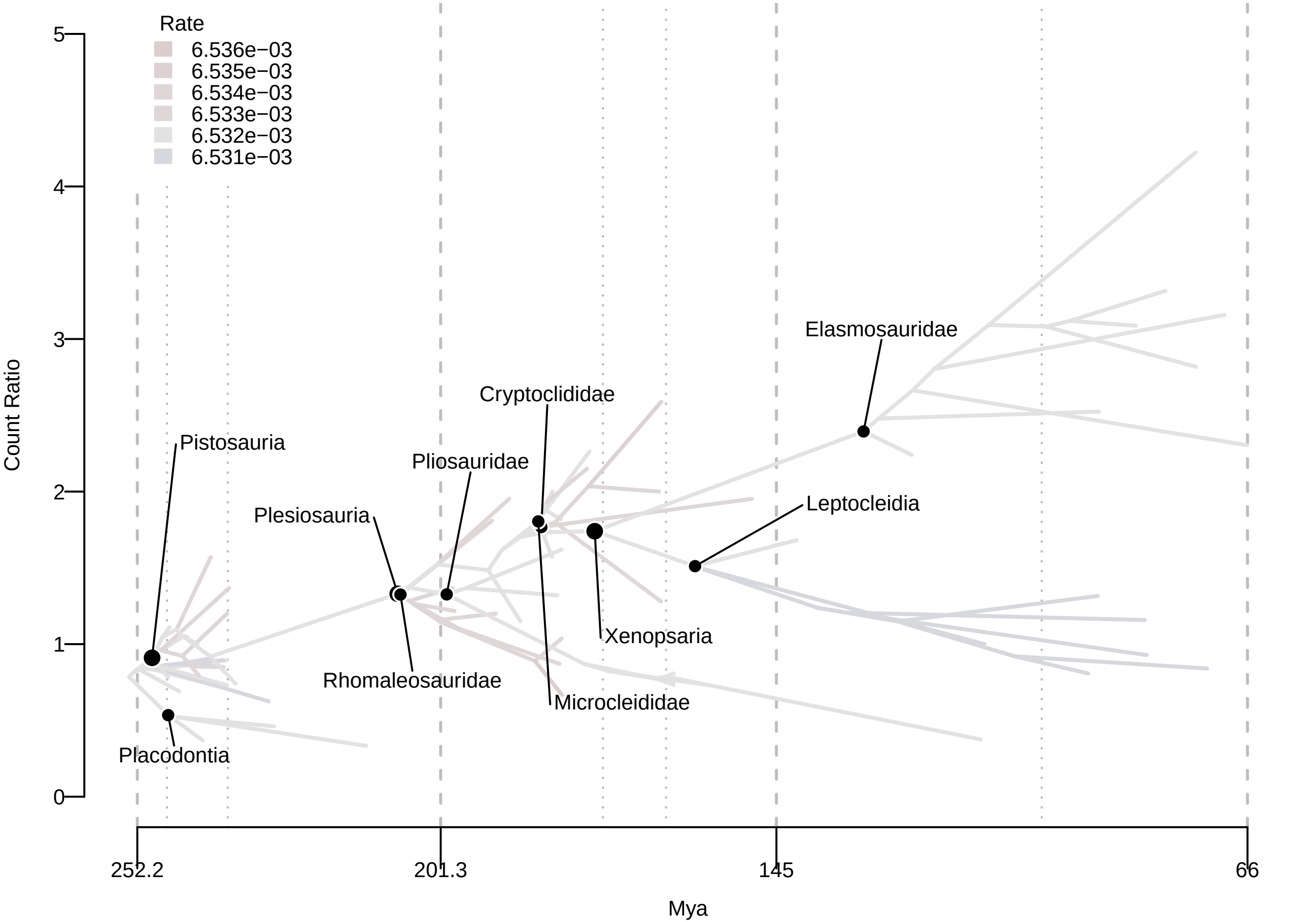

Rate
6.536e−03
6.535e−03
6.534e−03
6.533e−03
6.532e−03
6.531e−03
Count Ratio
0
1
2
3
4
5
Pistosauria
Placodontia
Plesiosauria
Rhomaleosauridae
Pliosauridae
Cryptoclididae
Microcleididae
Xenopsaria
Leptocleidia
Elasmosauridae
252.2
201.3
145
66
Mya

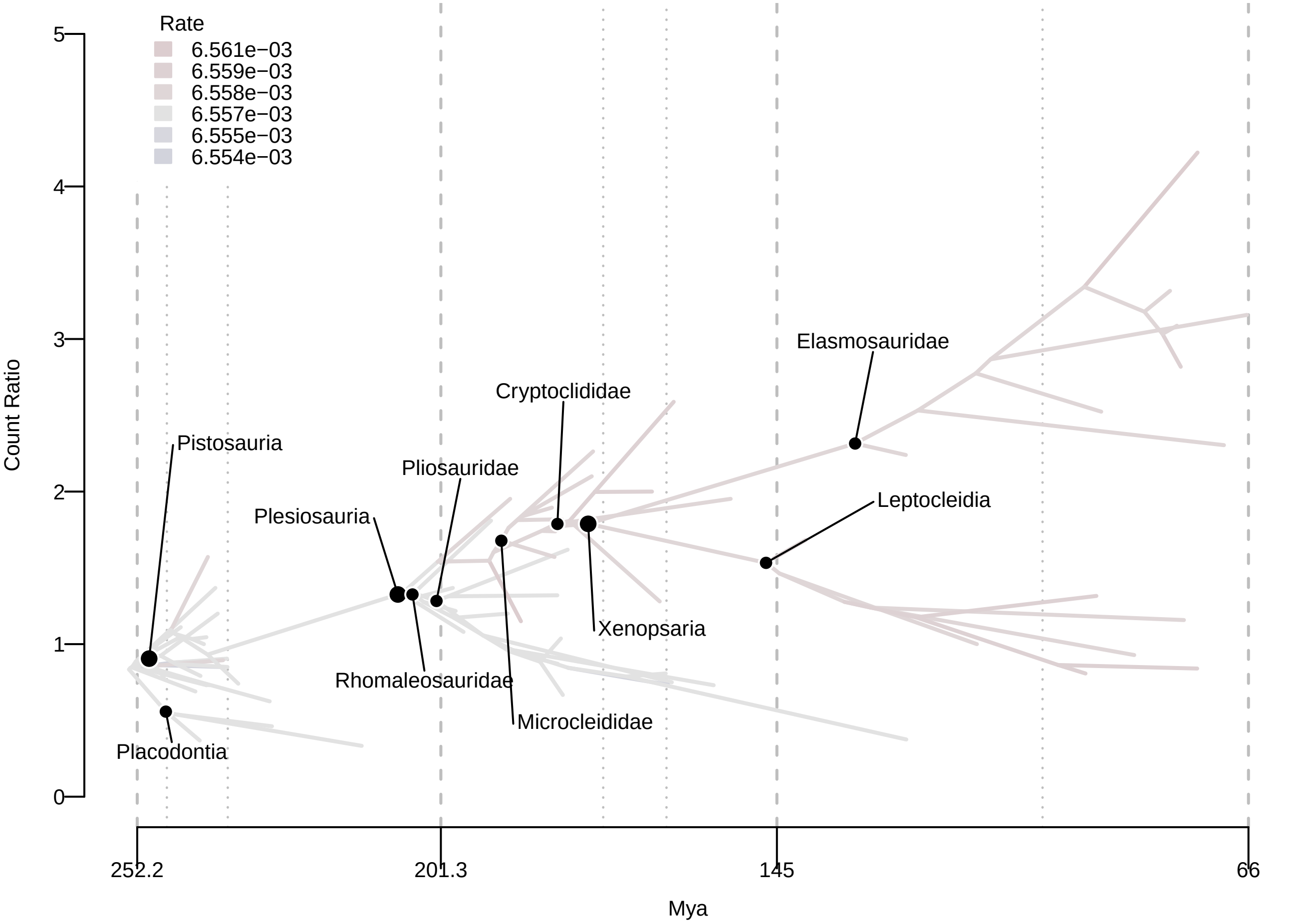
Rate
6.561e−03
6.559e−03
6.558e−03
6.557e−03
6.555e−03
6.554e−03
Count Ratio
0
1
2
3
4
5
Pistosauria
Placodontia
Plesiosauria
Rhomaleosauridae
Pliosauridae
Microcleididae
Cryptoclididae
Xenopsaria
Elasmosauridae
Leptocleidia
252.2
201.3
145
66
Mya

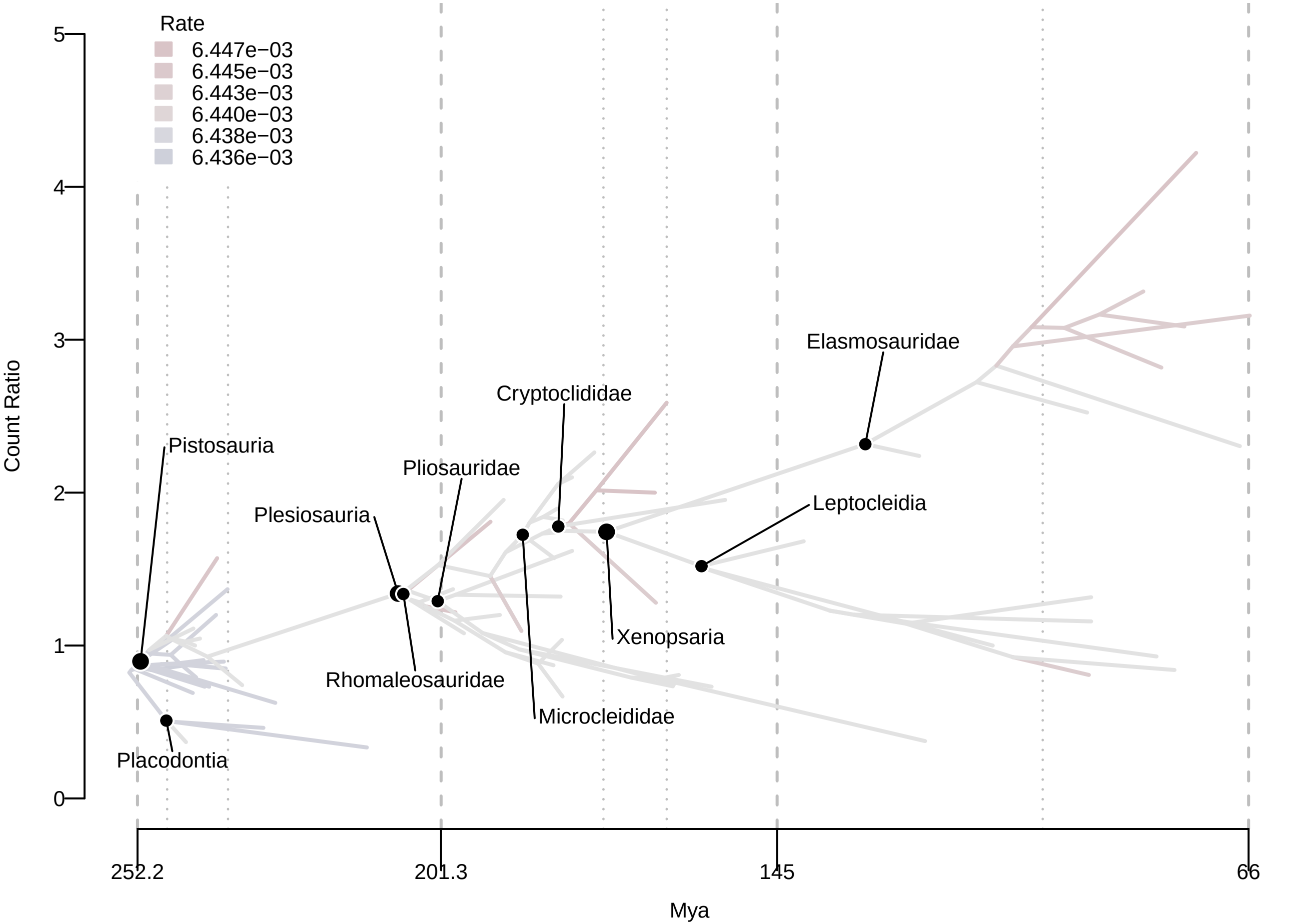
Rate
6.447e−03
6.445e−03
6.443e−03
6.440e−03
6.438e−03
6.436e−03
Count Ratio
0
1
2
3
4
5
Pistosauria
Placodontia
Plesiosauria
Rhomaleosauridae
Pliosauridae
Cryptoclididae
Microcleididae
Xenopsaria
Leptocleidia
Elasmosauridae
252.2
201.3
145
66
Mya

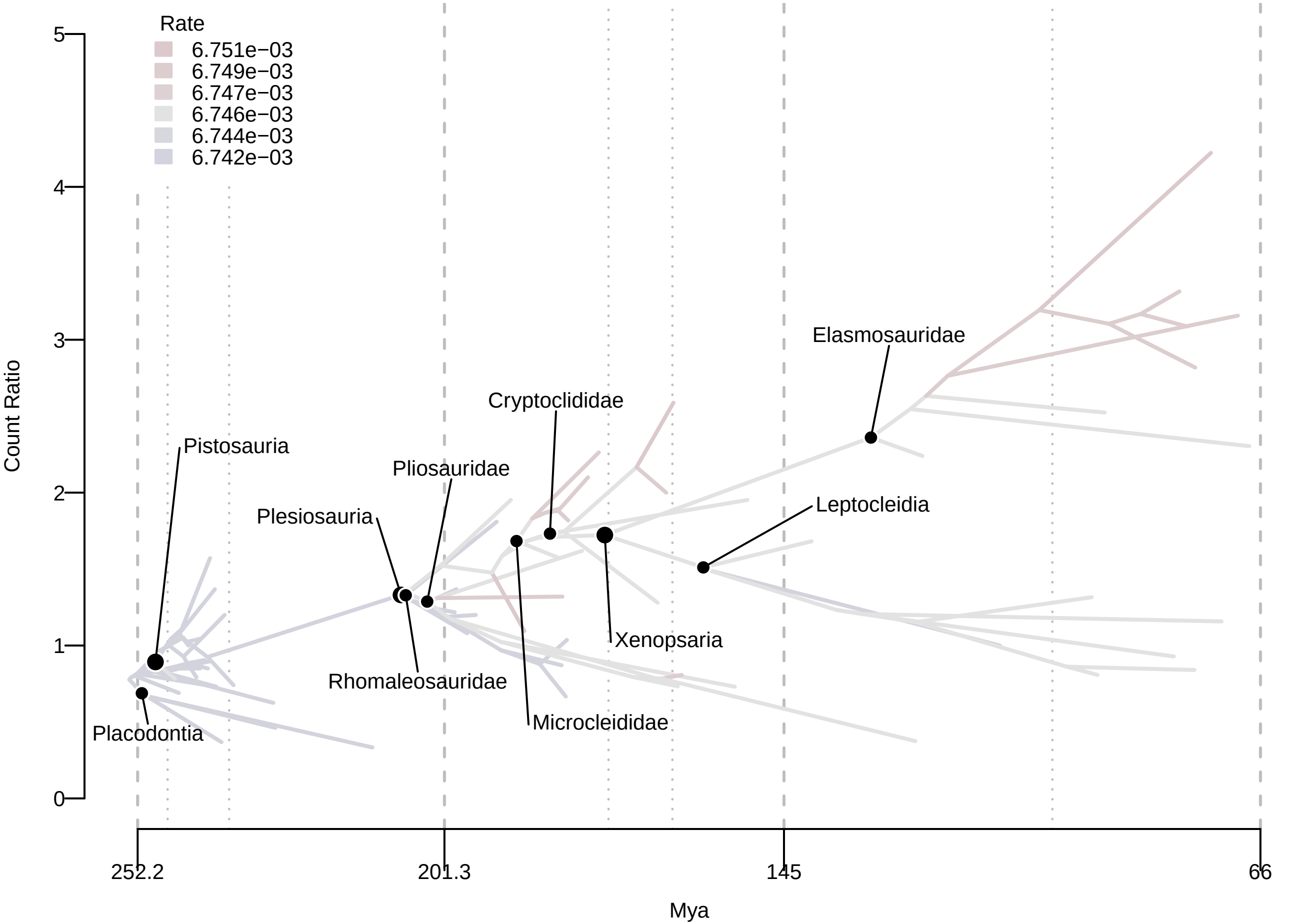
Rate
6.751e−03
6.749e−03
6.747e−03
6.746e−03
6.744e−03
6.742e−03
Count Ratio
0
1
2
3
4
5
Pistosauria
Placodontia
Plesiosauria
Rhomaleosauridae
Pliosauridae
Cryptoclididae
Microcleididae
Xenopsaria
Leptocleidia
Elasmosauridae
252.2
201.3
145
66
Mya

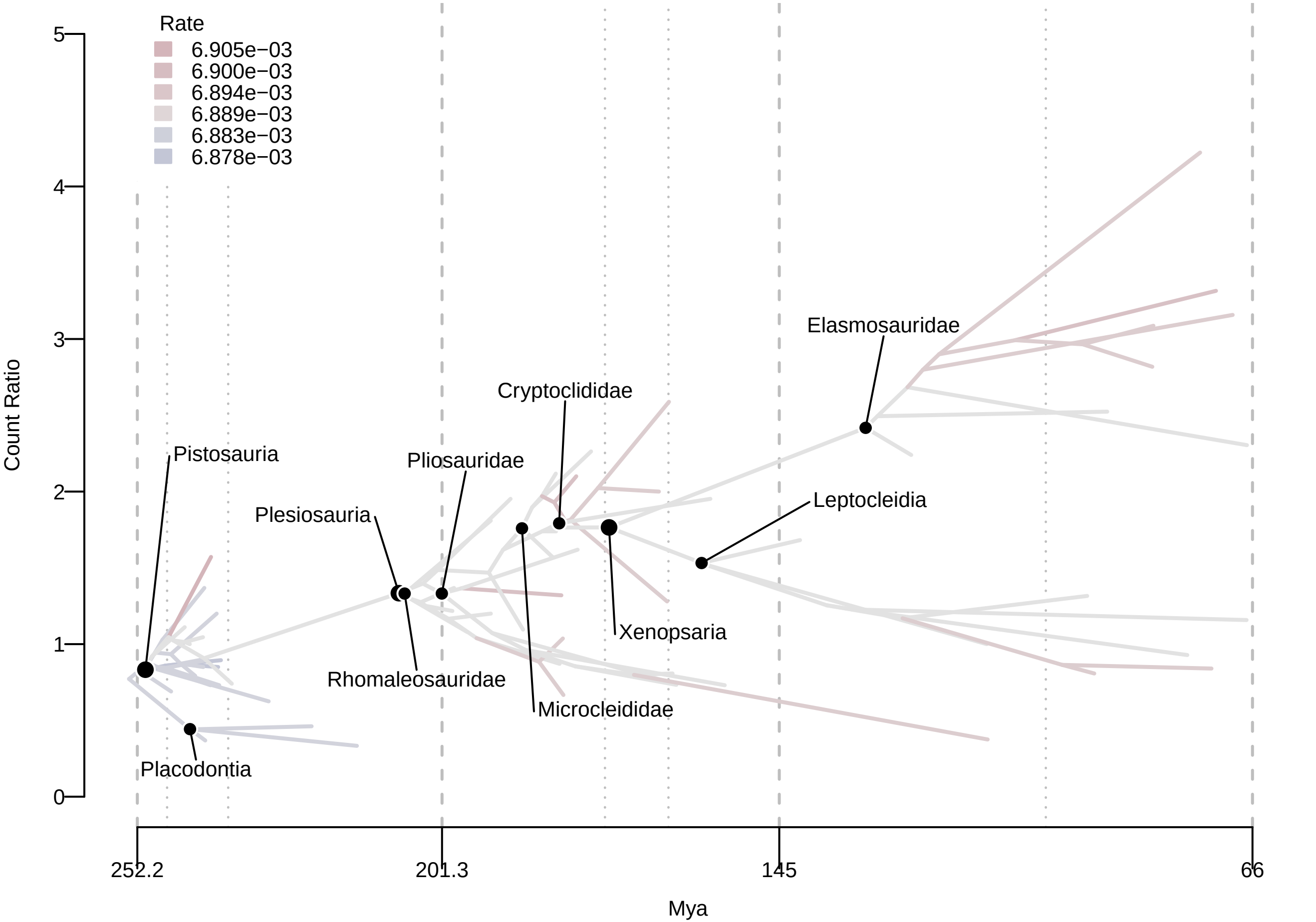

Rate
6.905e−03
6.900e−03
6.894e−03
6.889e−03
6.883e−03
6.878e−03
Count Ratio
0
1
2
3
4
5
Pistosauria
Plesiosauria
Pliosauridae
Rhomaleosauridae
Cryptoclididae
Microcleididae
Xenopsaria
Elasmosauridae
Leptocleidia
Placodontia
252.2
201.3
145
66
Mya

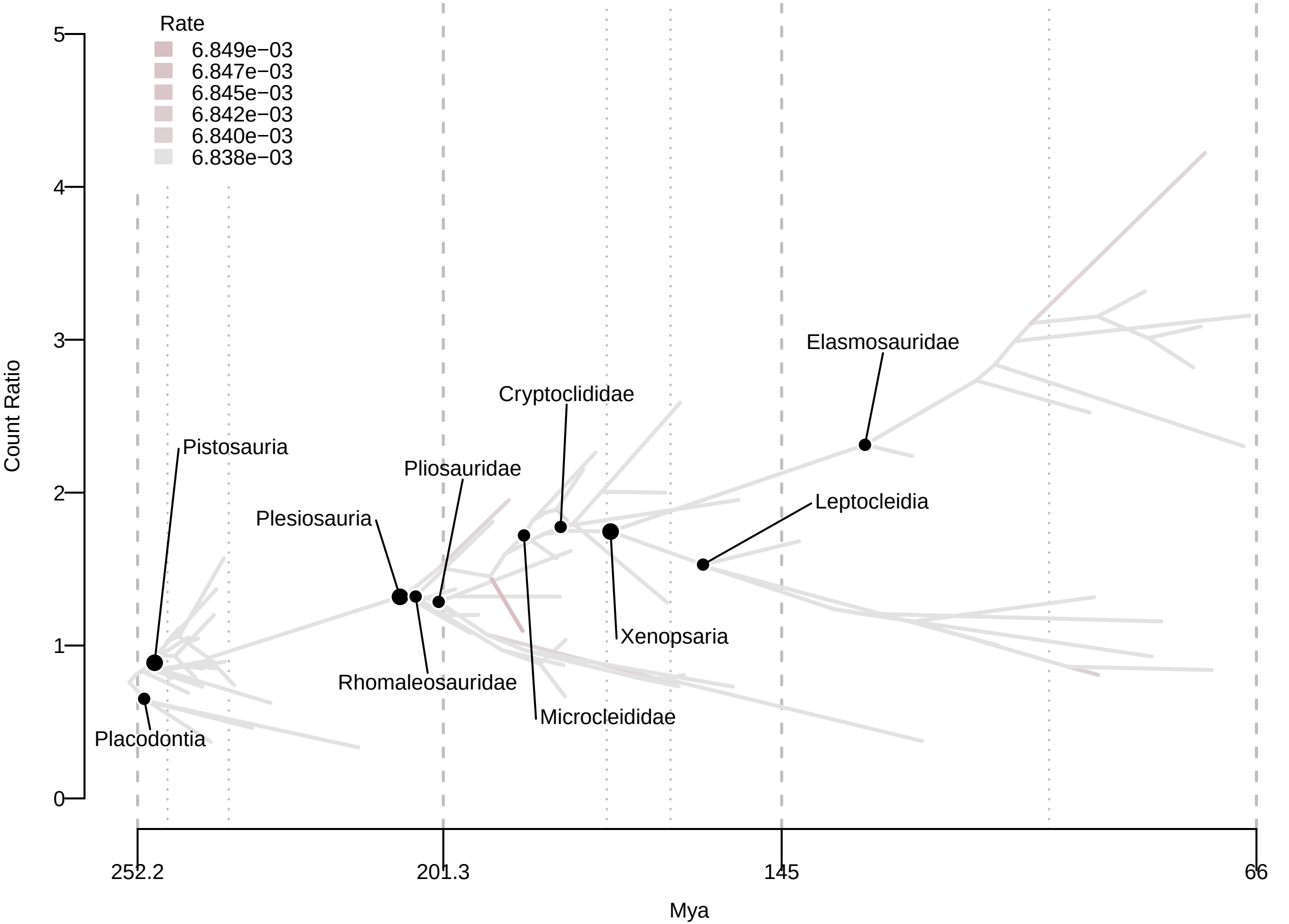

Rate
6.849e−03
6.847e−03
6.845e−03
6.842e−03
6.840e−03
6.838e−03
Count Ratio
Mya
252.2
201.3
145
66
Pistosauria
Placodontia
Plesiosauria
Pliosauridae
Rhomaleosauridae
Cryptoclididae
Microcleididae
Xenopsaria
Leptocleidia
Elasmosauridae

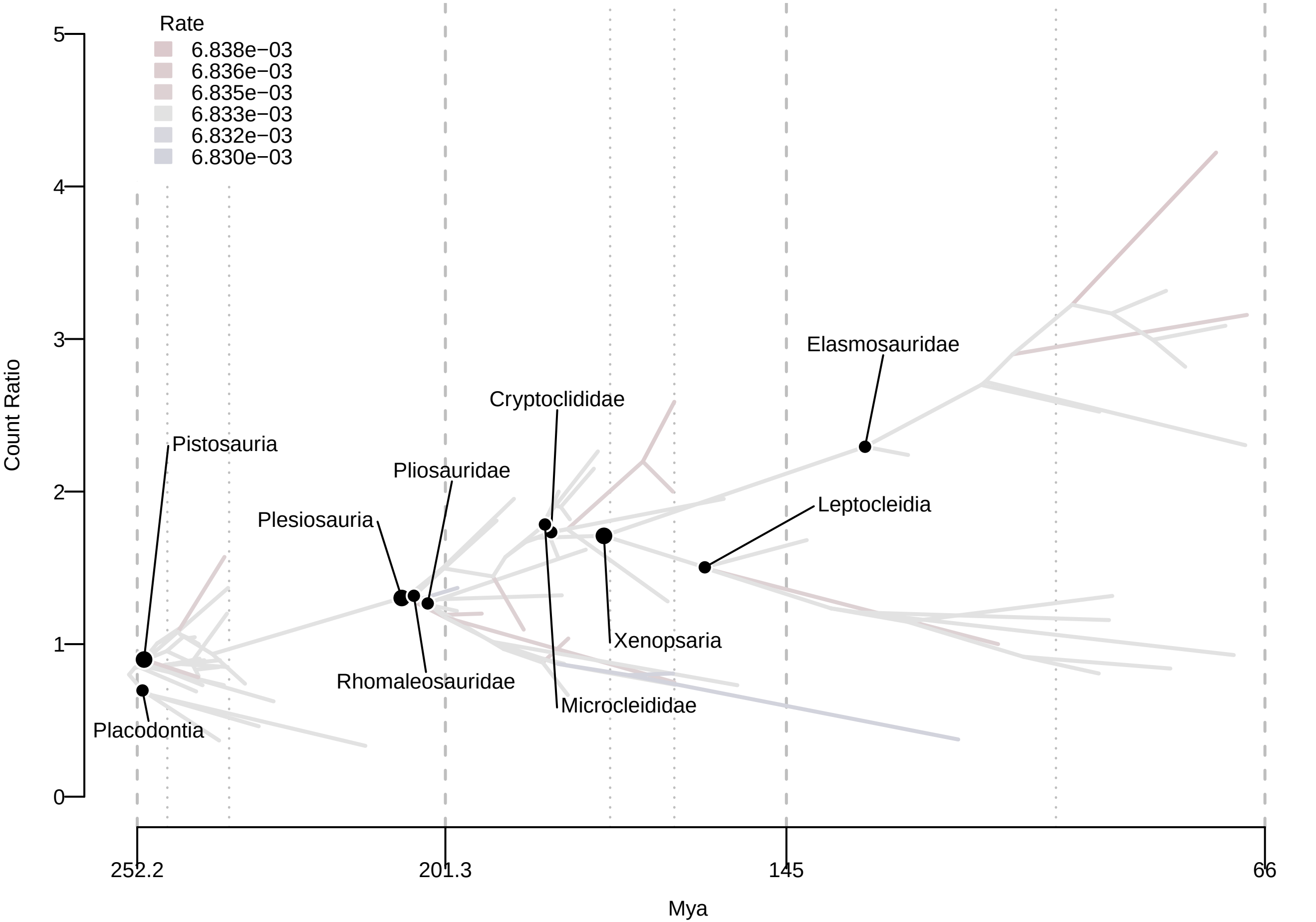

Rate
6.838e−03
6.836e−03
6.835e−03
6.833e−03
6.832e−03
6.830e−03
Count Ratio
0
1
2
3
4
5
Pistosauria
Placodontia
Plesiosauria
Pliosauridae
Rhomaleosauridae
Cryptoclididae
Microcleididae
Xenopsaria
Leptocleidia
Elasmosauridae
252.2
201.3
145
66
Mya

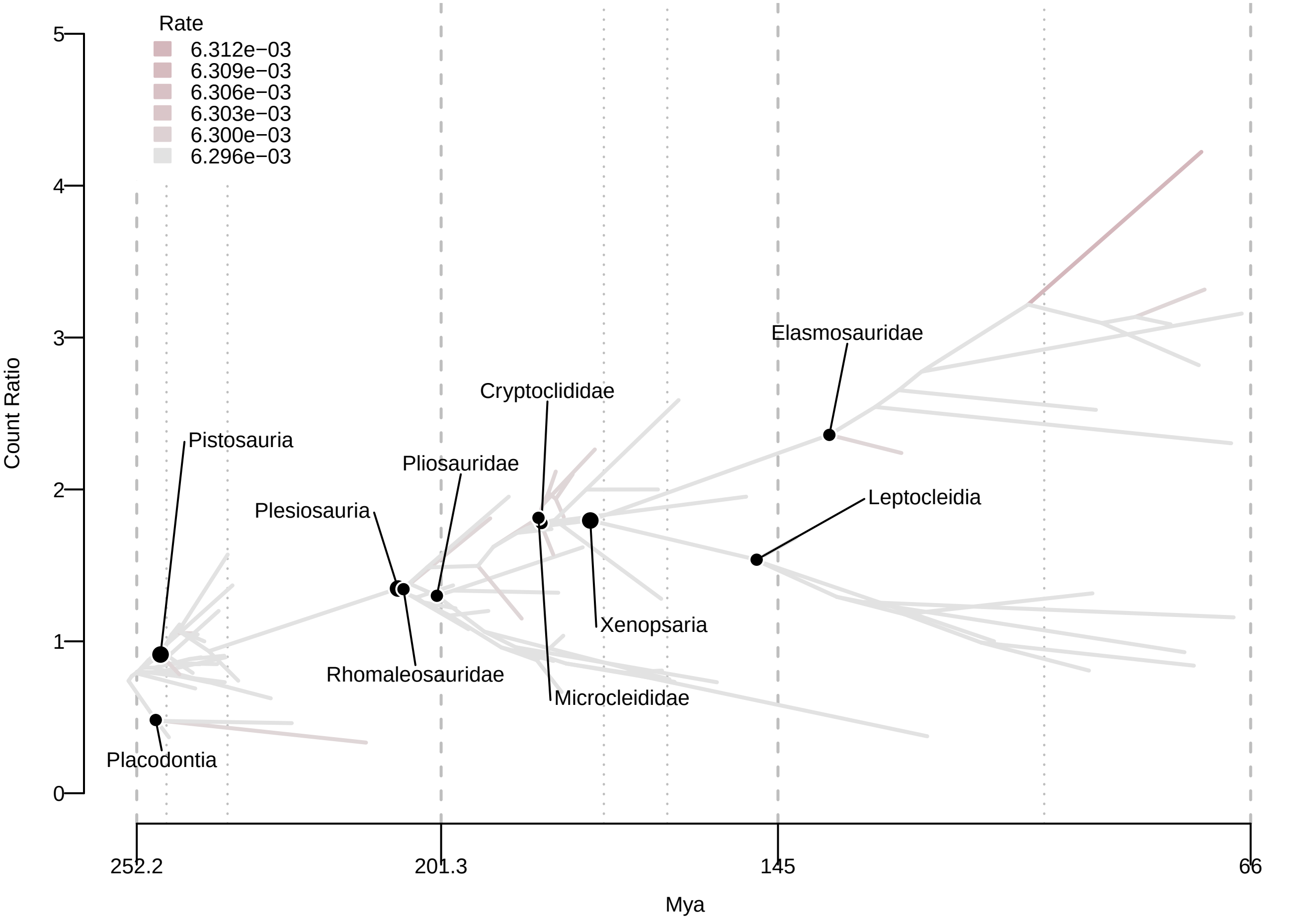

Rate
6.312e−03
6.309e−03
6.306e−03
6.303e−03
6.300e−03
6.296e−03
Count Ratio
0
1
2
3
4
5
Pistosauria
Plesiosauria
Pliosauridae
Rhomaleosauridae
Cryptoclididae
Microcleididae
Xenopsaria
Elasmosauridae
Leptocleidia
Placodontia
252.2
201.3
145
66
Mya

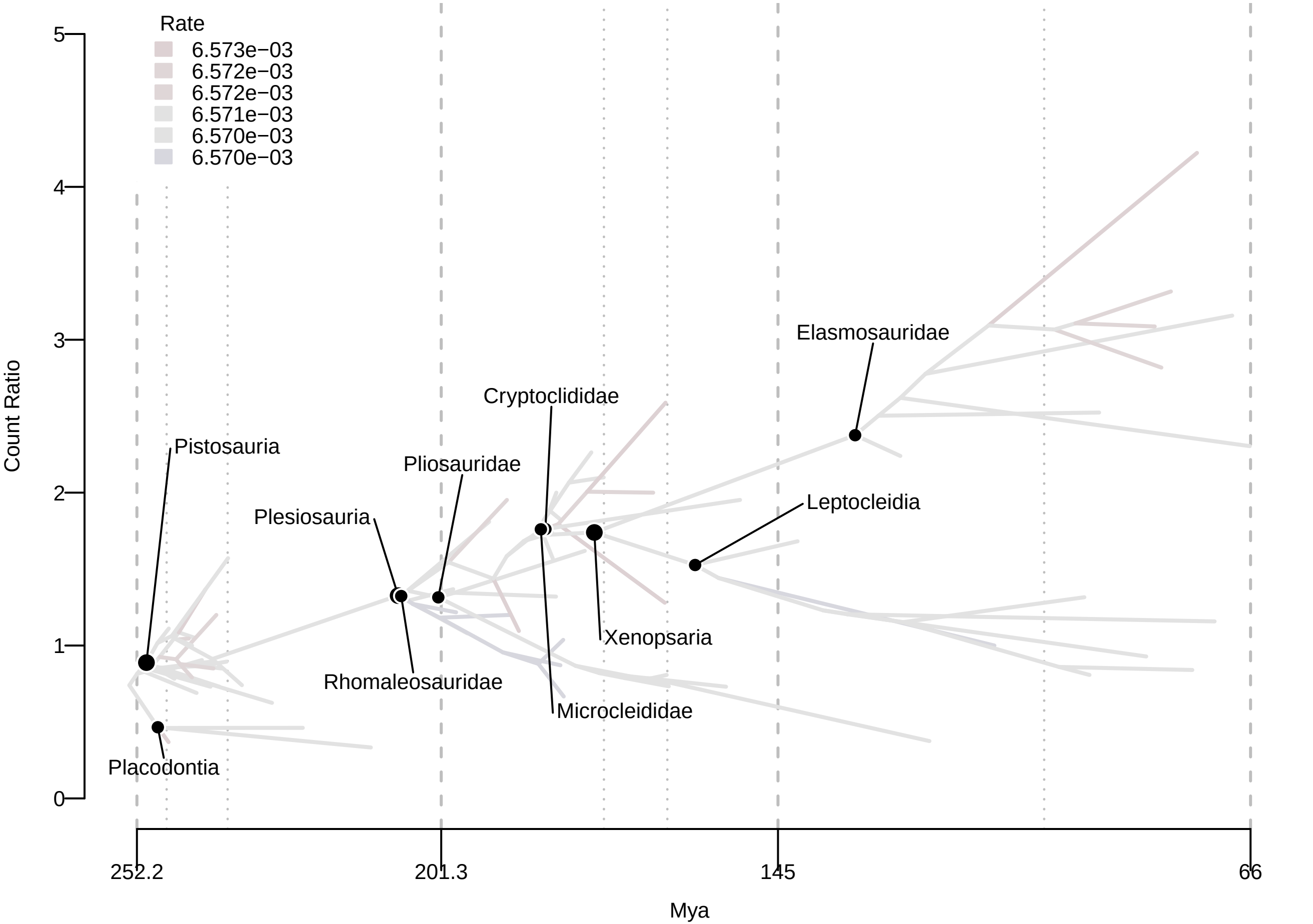

Rate
6.573e−03
6.572e−03
6.572e−03
6.571e−03
6.570e−03
6.570e−03
Count Ratio
0
1
2
3
4
5
Pistosauria
Placodontia
Plesiosauria
Rhomaleosauridae
Pliosauridae
Cryptoclididae
Microcleididae
Xenopsaria
Leptocleidia
Elasmosauridae
252.2
201.3
145
66
Mya

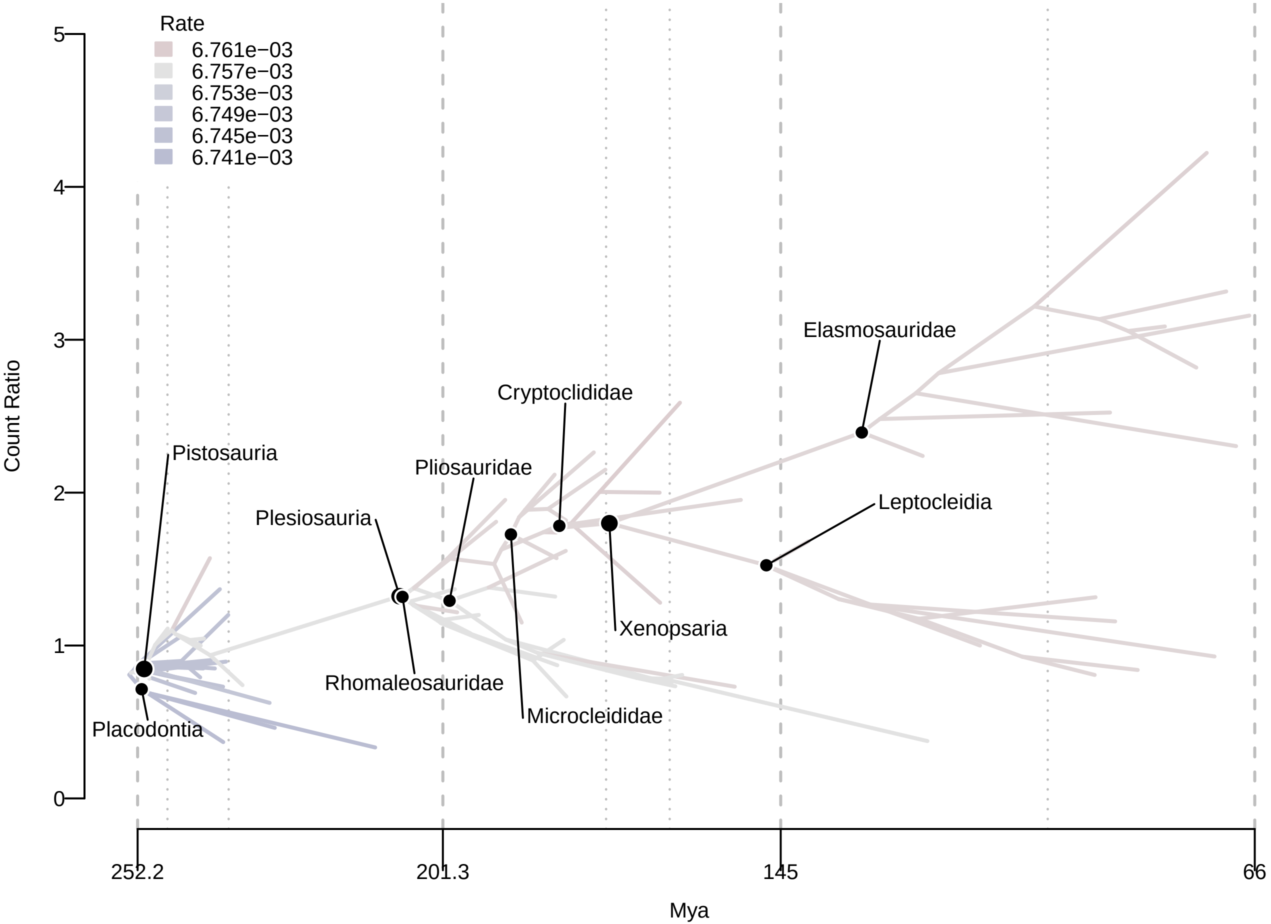

Rate
6.761e−03
6.757e−03
6.753e−03
6.749e−03
6.745e−03
6.741e−03
Count Ratio
0
1
2
3
4
5
Pistosauria
Placodontia
Plesiosauria
Rhomaleosauridae
Pliosauridae
Microcleididae
Cryptoclididae
Xenopsaria
Elasmosauridae
Leptocleidia
252.2
201.3
145
66
Mya

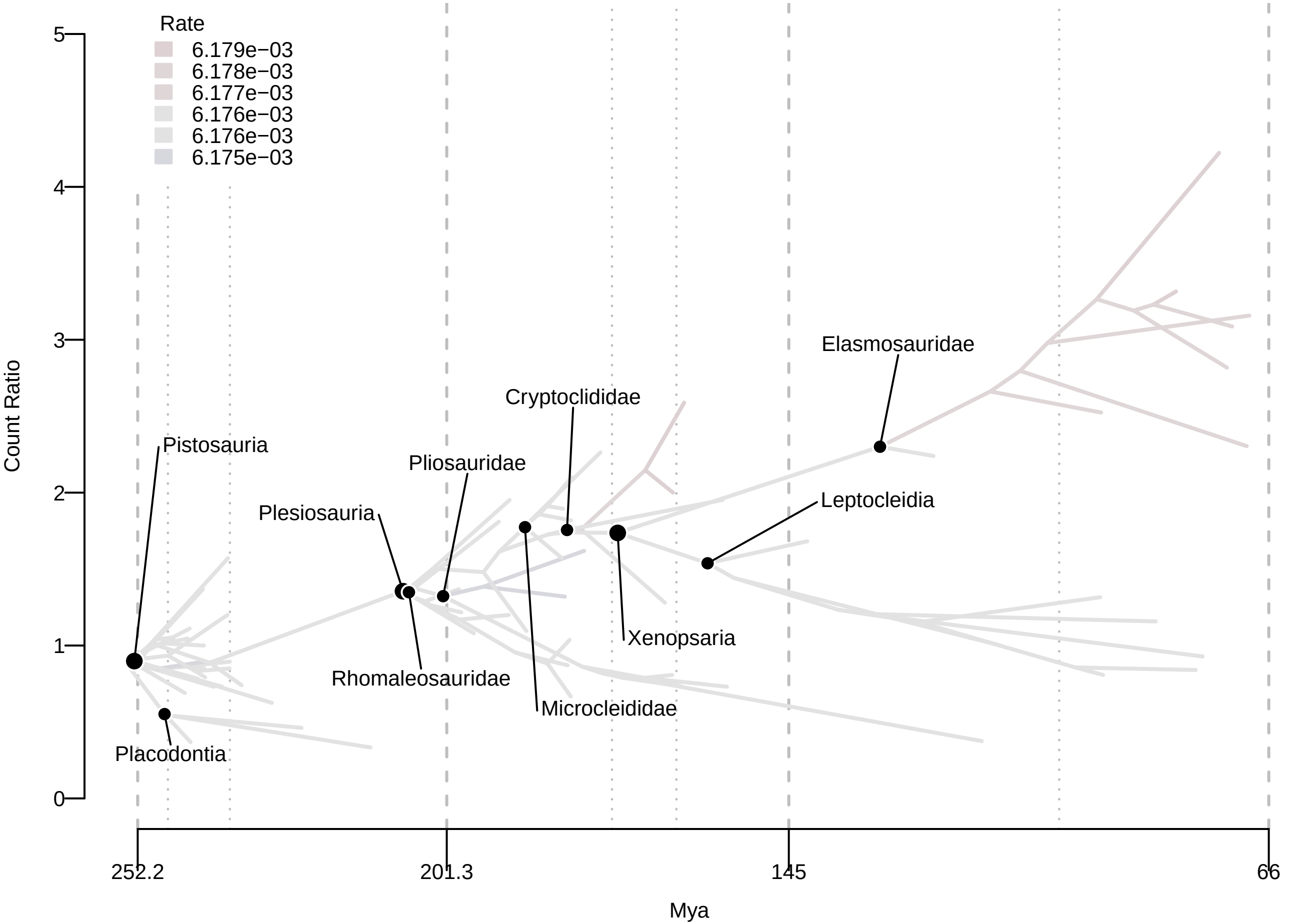

Rate
6.179e−03
6.178e−03
6.177e−03
6.176e−03
6.176e−03
6.175e−03
Count Ratio
0
1
2
3
4
5
Pistosauria
Placodontia
Plesiosauria
Rhomaleosauridae
Pliosauridae
Microcleididae
Cryptoclididae
Xenopsaria
Leptocleidia
Elasmosauridae
252.2
201.3
145
66
Mya

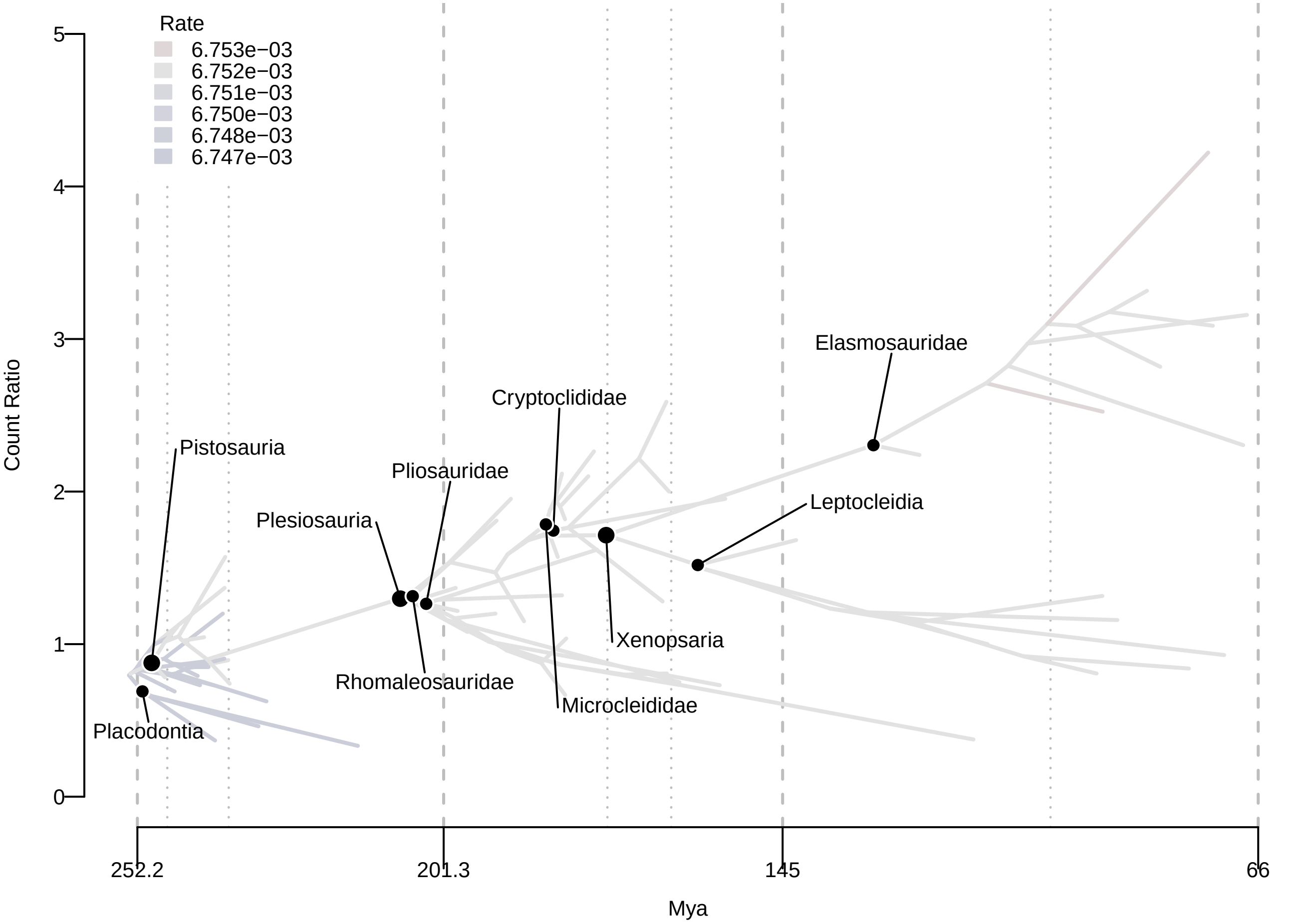

Rate
6.753e−03
6.752e−03
6.751e−03
6.750e−03
6.748e−03
6.747e−03
Count Ratio
0
1
2
3
4
5
Pistosauria
Placodontia
Plesiosauria
Pliosauridae
Rhomaleosauridae
Cryptoclididae
Microcleididae
Xenopsaria
Leptocleidia
Elasmosauridae
252.2
201.3
145
66
Mya

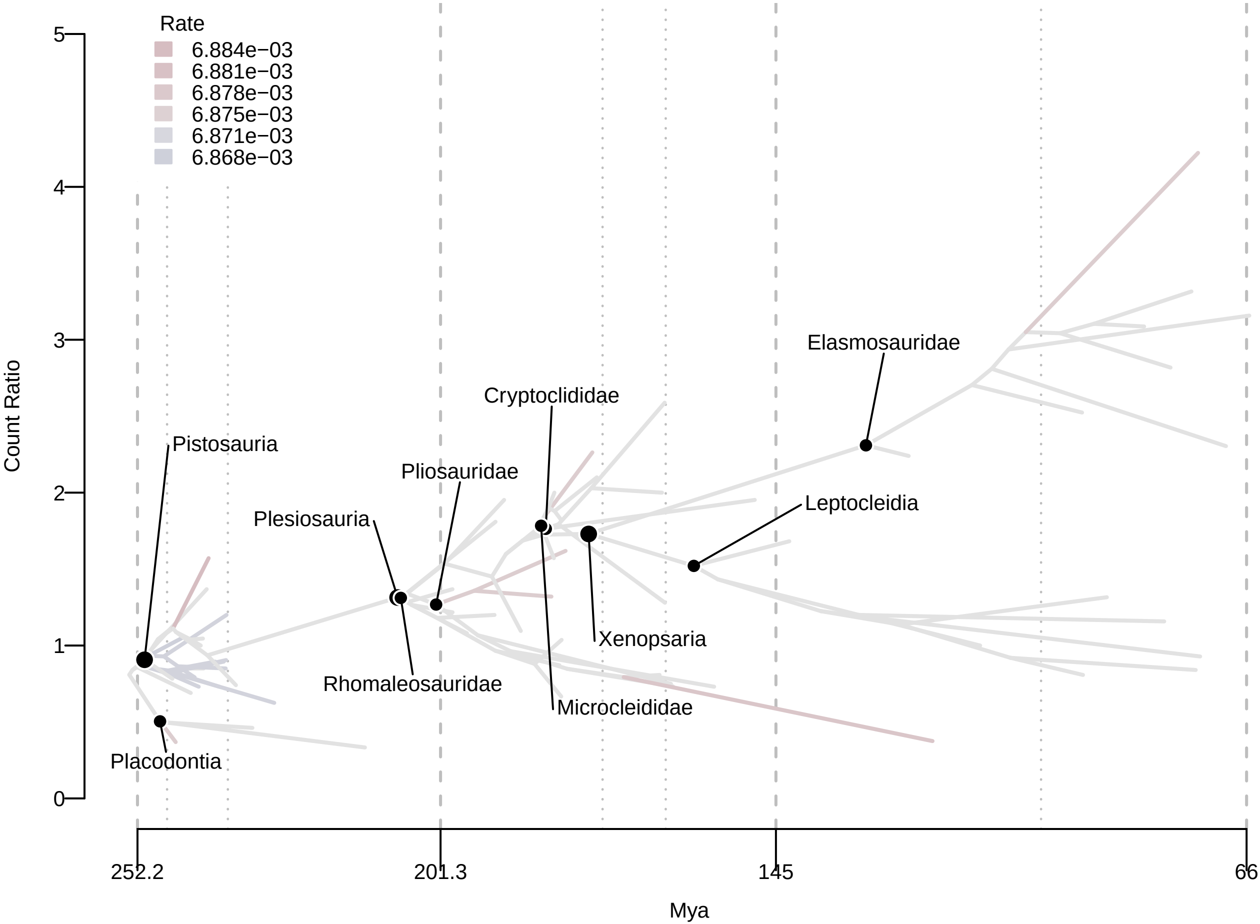

Rate
6.884e−03
6.881e−03
6.878e−03
6.875e−03
6.871e−03
6.868e−03
Count Ratio
5
4
3
2
1
0
Pistosauria
Placodontia
Plesiosauria
Rhomaleosauridae
Pliosauridae
Cryptoclididae
Microcleididae
Xenopsaria
Leptocleidia
Elasmosauridae
252.2
201.3
145
66
Mya

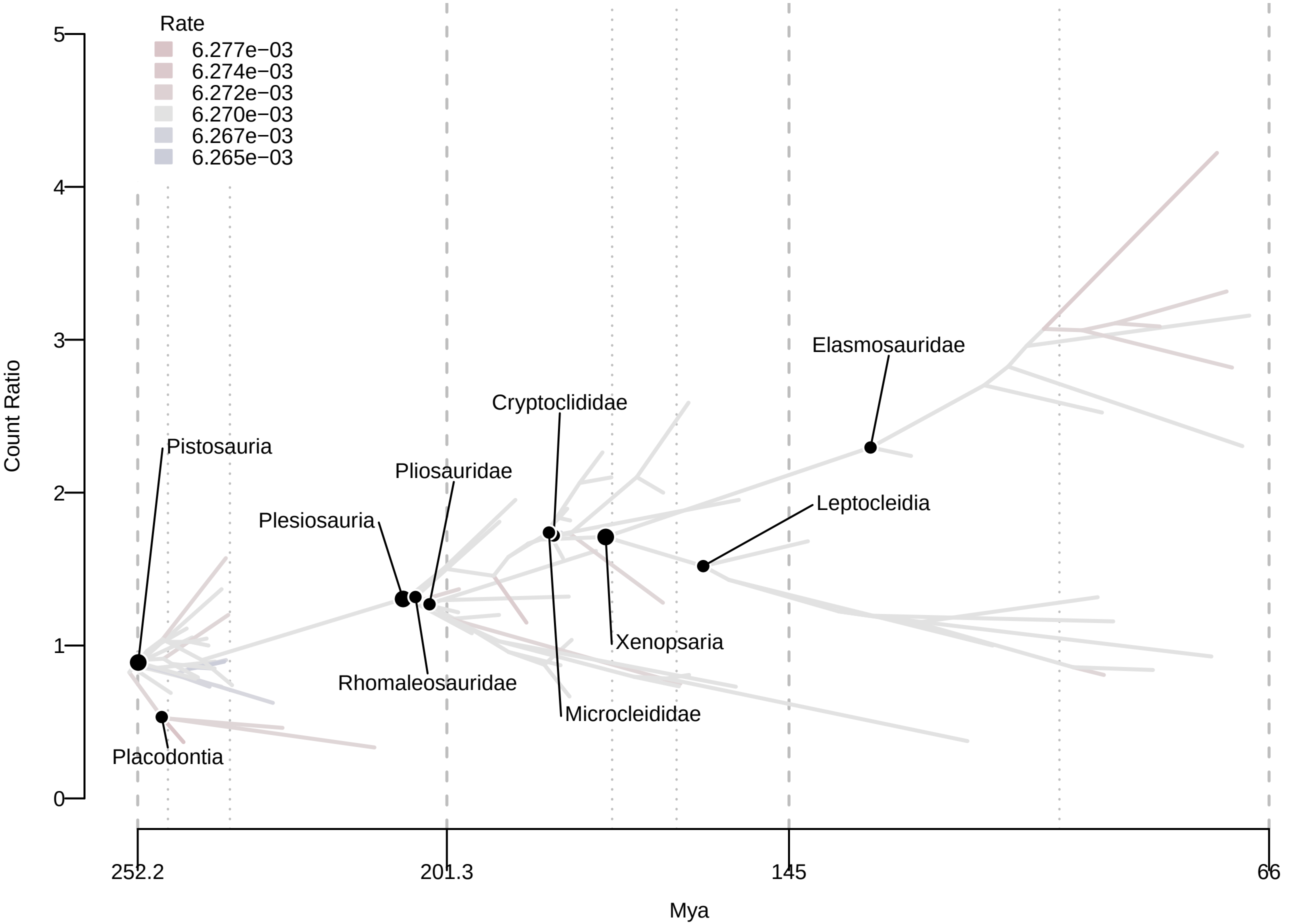

Rate
6.277e−03
6.274e−03
6.272e−03
6.270e−03
6.267e−03
6.265e−03
Count Ratio
0
1
2
3
4
5
Pistosauria
Placodontia
Plesiosauria
Pliosauridae
Rhomaleosauridae
Cryptoclididae
Microcleididae
Xenopsaria
Leptocleidia
Elasmosauridae
252.2
201.3
145
66
Mya

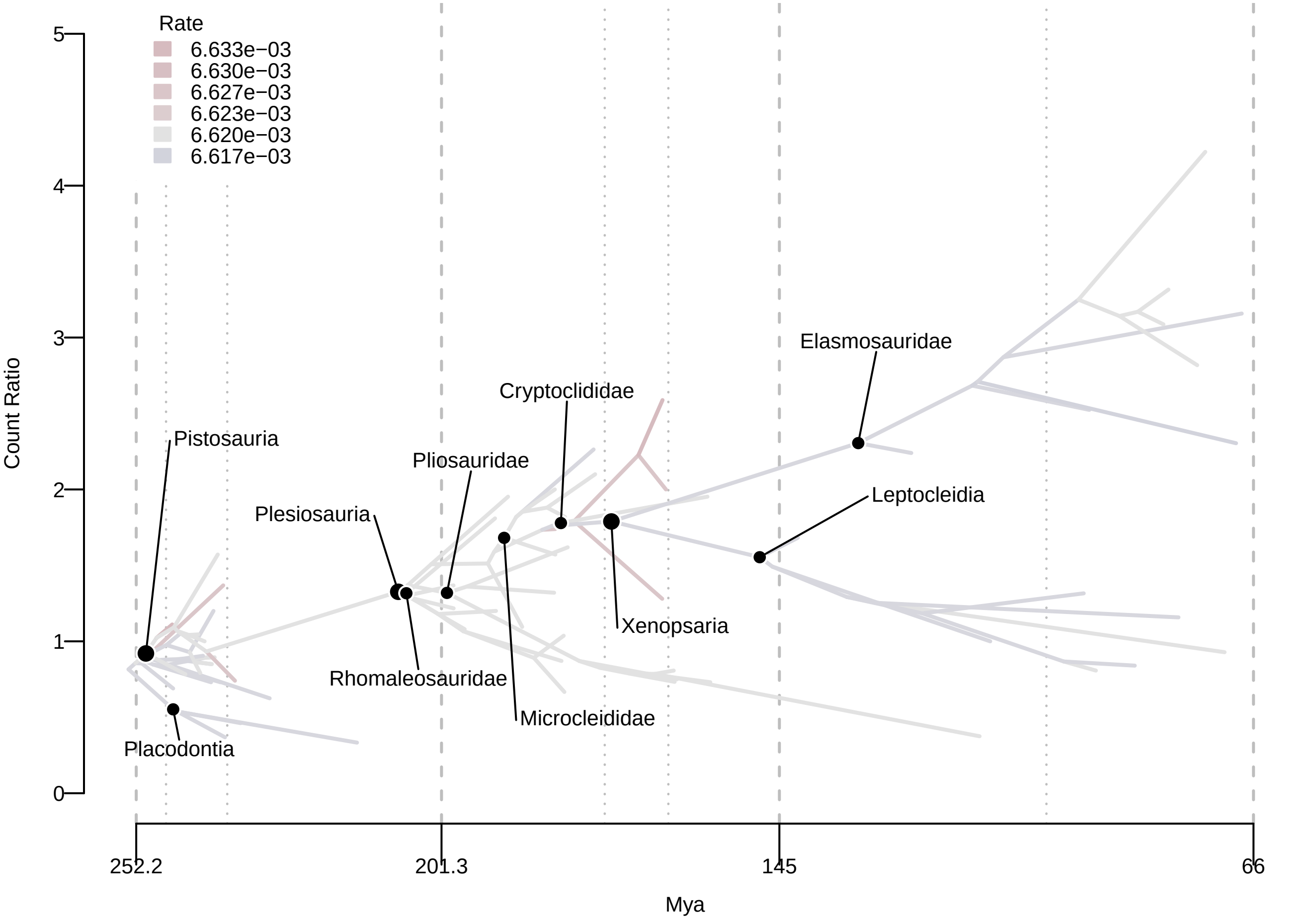
Rate
6.633e−03
6.630e−03
6.627e−03
6.623e−03
6.620e−03
6.617e−03
Count Ratio
0
1
2
3
4
5
Mya
252.2
201.3
145
66
Pistosauria
Placodontia
Plesiosauria
Rhomaleosauridae
Pliosauridae
Microcleididae
Cryptoclididae
Xenopsaria
Elasmosauridae
Leptocleidia

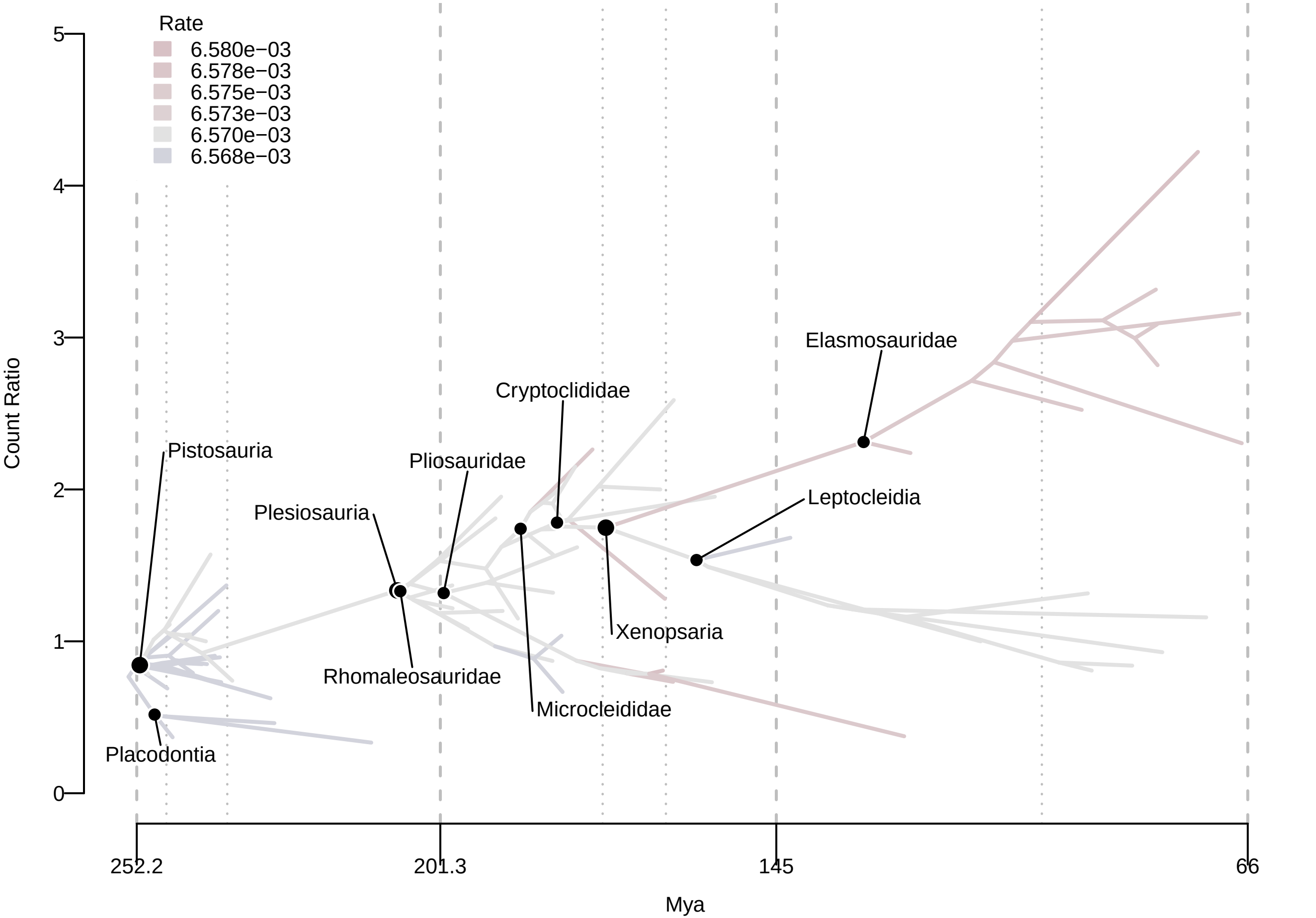

Rate
6.580e−03
6.578e−03
6.575e−03
6.573e−03
6.570e−03
6.568e−03
Count Ratio
0
1
2
3
4
5
Pistosauria
Plesiosauria
Pliosauridae
Cryptoclididae
Elasmosauridae
Leptocleidia
Xenopsaria
Rhomaleosauridae
Microcleididae
Placodontia
252.2
201.3
145
66
Mya

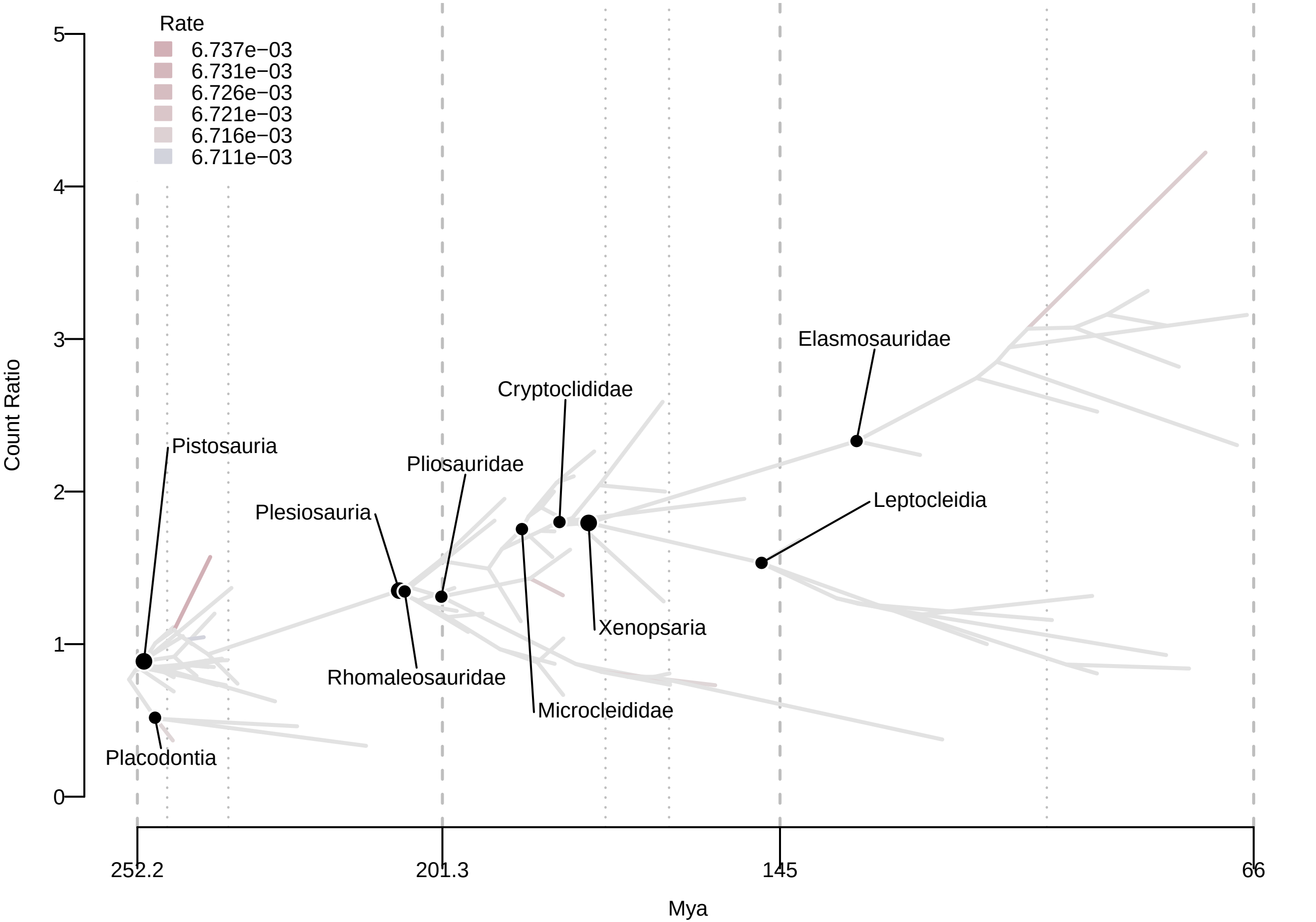
Rate
6.737e−03
6.731e−03
6.726e−03
6.721e−03
6.716e−03
6.711e−03
Count Ratio
0
1
2
3
4
5
Pistosauria
Placodontia
Plesiosauria
Rhomaleosauridae
Pliosauridae
Microcleididae
Cryptoclididae
Xenopsaria
Elasmosauridae
Leptocleidia
252.2
201.3
145
66
Mya

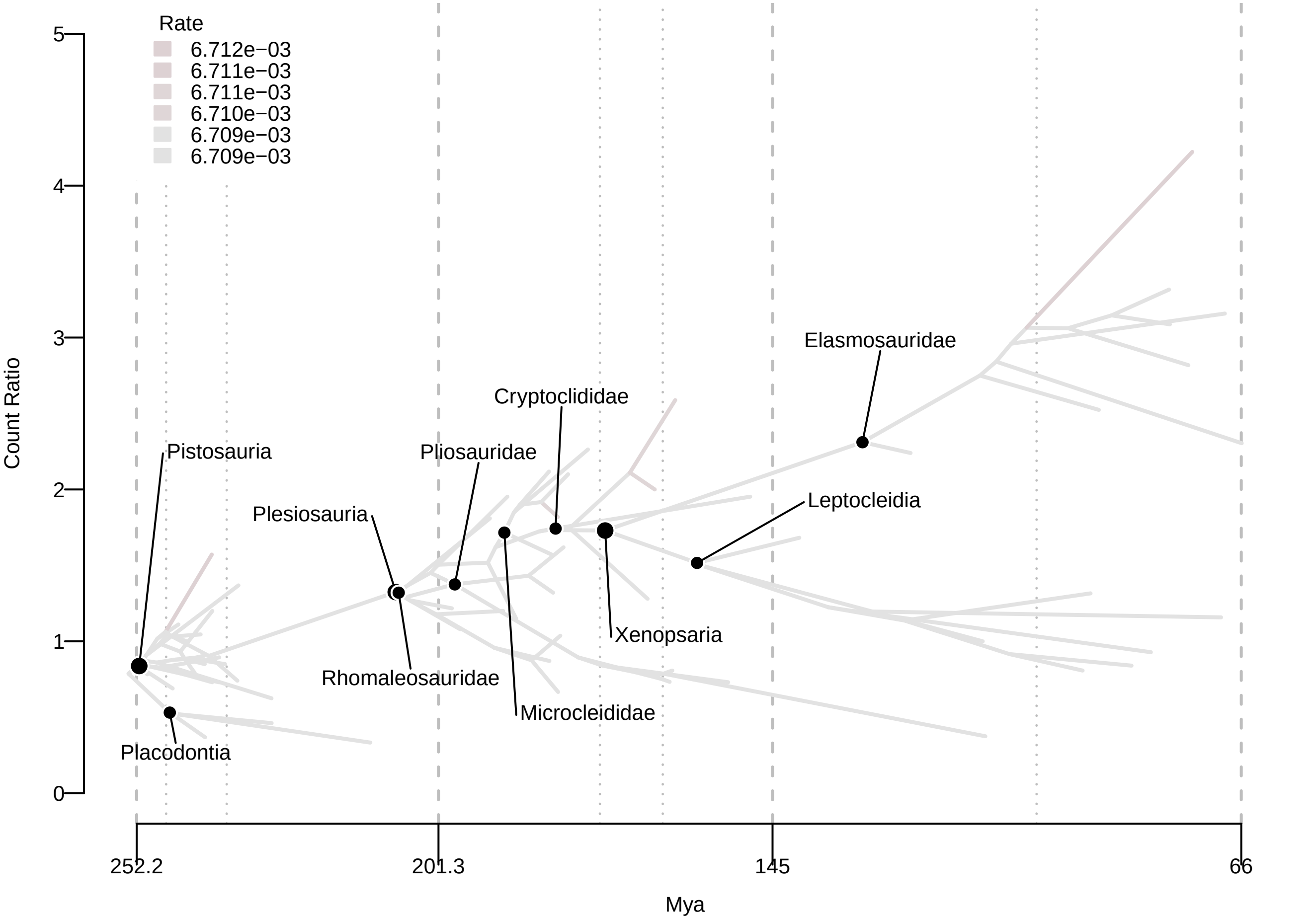

Rate
6.712e−03
6.711e−03
6.711e−03
6.710e−03
6.709e−03
6.709e−03
Count Ratio
0
1
2
3
4
5
Pistosauria
Plesiosauria
Pliosauridae
Cryptoclididae
Elasmosauridae
Leptocleidia
Xenopsaria
Microcleididae
Rhomaleosauridae
Placodontia
252.2
201.3
145
66
Mya

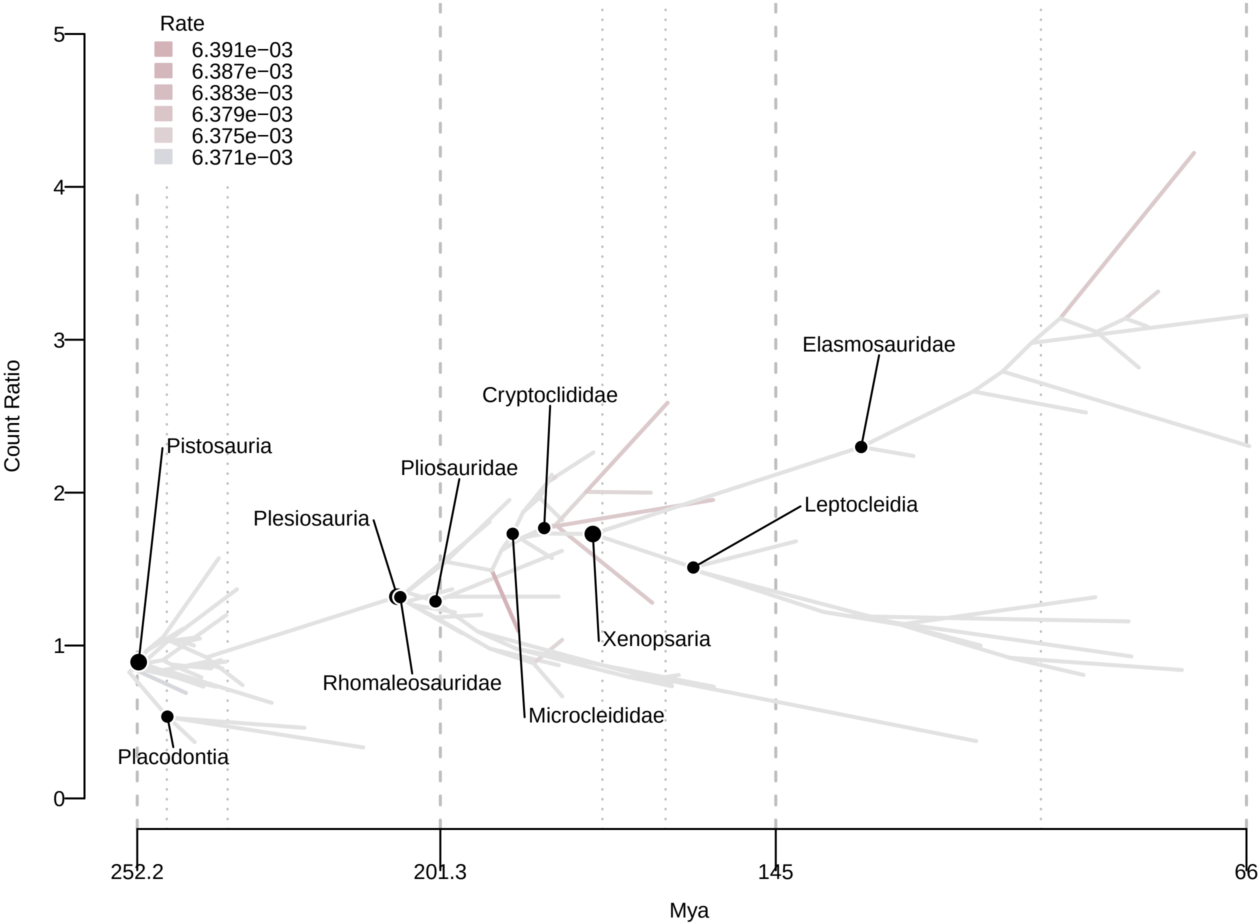
Rate
6.391e−03
6.387e−03
6.383e−03
6.379e−03
6.375e−03
6.371e−03
Count Ratio
0
1
2
3
4
5
Pistosauria
Placodontia
Plesiosauria
Rhomaleosauridae
Pliosauridae
Microcleididae
Cryptoclididae
Xenopsaria
Leptocleidia
Elasmosauridae
252.2
201.3
145
66
Mya

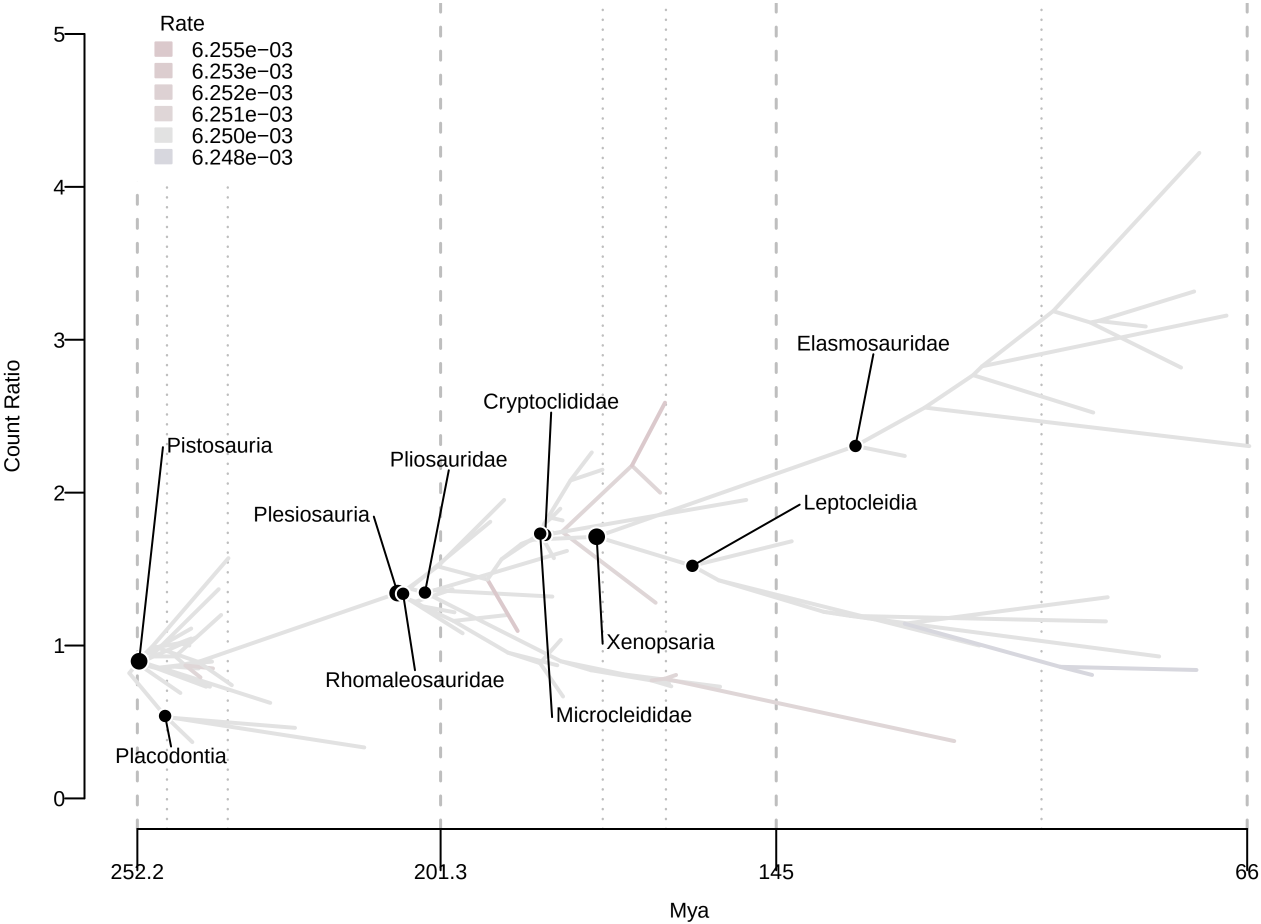

Rate
6.255e−03
6.253e−03
6.252e−03
6.251e−03
6.250e−03
6.248e−03
Count Ratio
5
4
3
2
1
0
Pistosauria
Placodontia
Plesiosauria
Pliosauridae
Rhomaleosauridae
Cryptoclididae
Microcleididae
Xenopsaria
Leptocleidia
Elasmosauridae
252.2
201.3
145
66
Mya

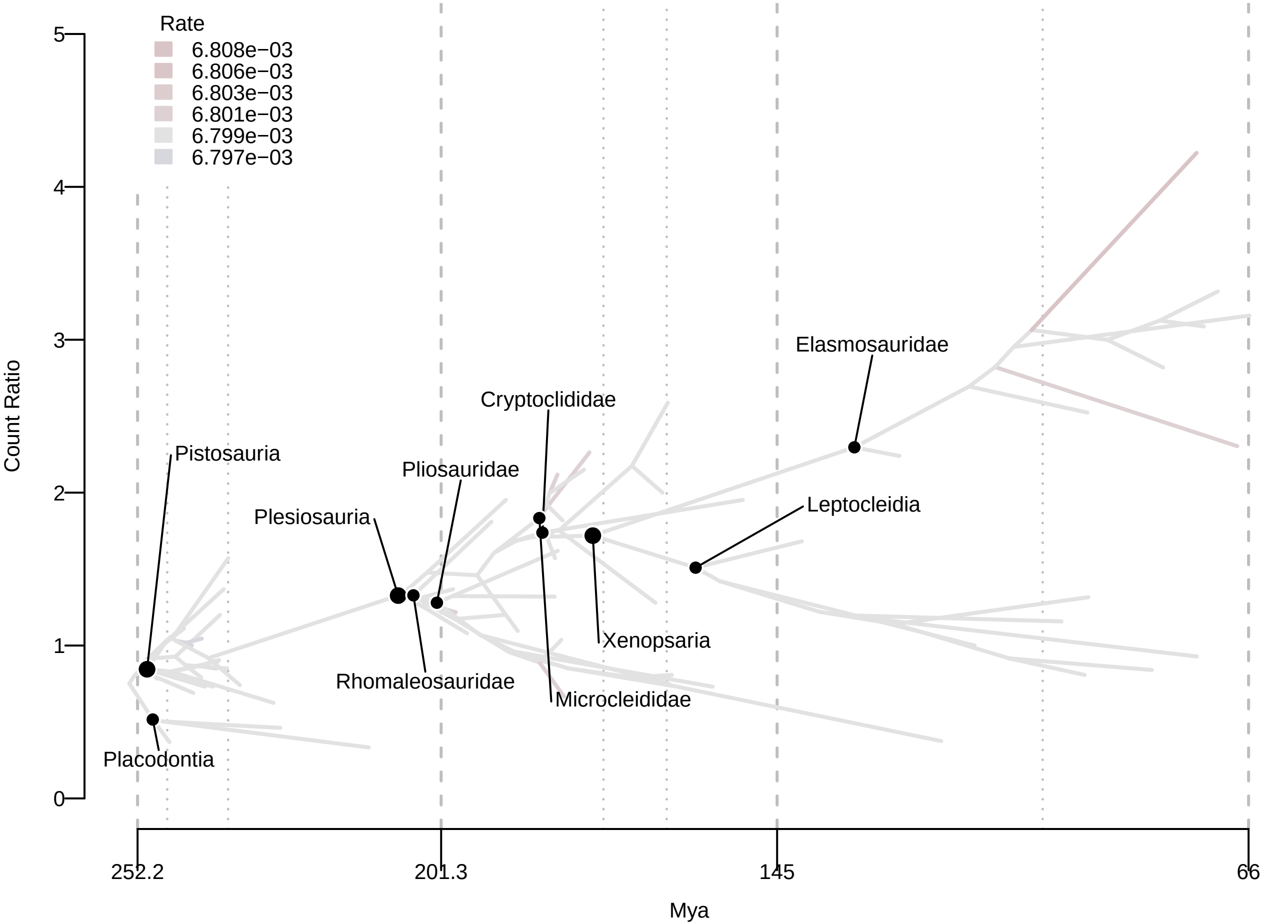

Rate
6.808e−03
6.806e−03
6.803e−03
6.801e−03
6.799e−03
6.797e−03
Count Ratio
0
1
2
3
4
5
Pistosauria
Placodontia
Plesiosauria
Pliosauridae
Rhomaleosauridae
Cryptoclididae
Microcleididae
Xenopsaria
Leptocleidia
Elasmosauridae
252.2
201.3
145
66
Mya

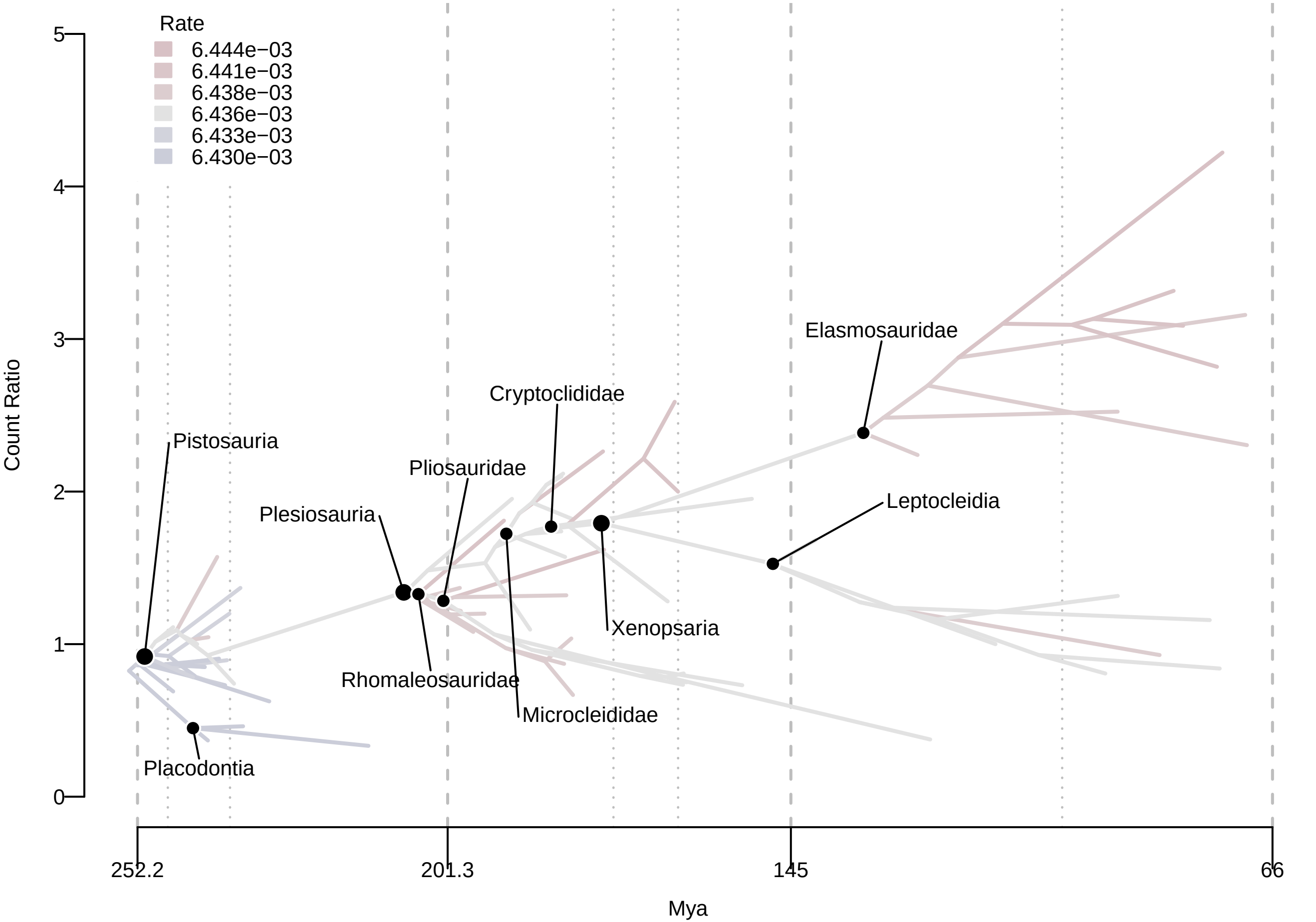

Rate
6.444e−03
6.441e−03
6.438e−03
6.436e−03
6.433e−03
6.430e−03
Count Ratio
0
1
2
3
4
5
Mya
252.2
201.3
145
66
Pistosauria
Placodontia
Plesiosauria
Pliosauridae
Rhomaleosauridae
Microcleididae
Cryptoclididae
Xenopsaria
Leptocleidia
Elasmosauridae

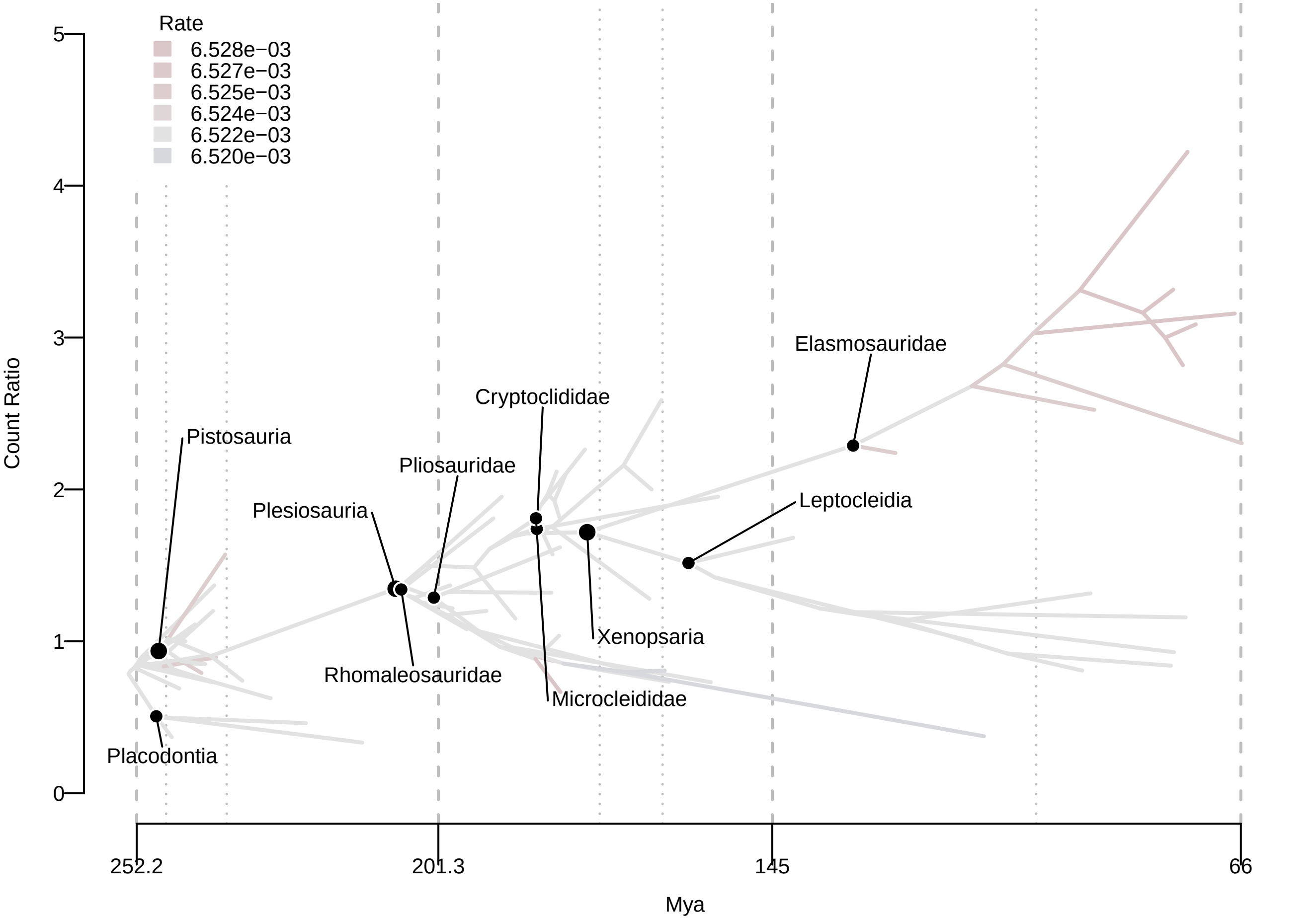
Rate
6.528e−03
6.527e−03
6.525e−03
6.524e−03
6.522e−03
6.520e−03
Count Ratio
0
1
2
3
4
5
Pistosauria
Placodontia
Plesiosauria
Rhomaleosauridae
Pliosauridae
Cryptoclididae
Microcleididae
Xenopsaria
Leptocleidia
Elasmosauridae
252.2
201.3
145
66
Mya

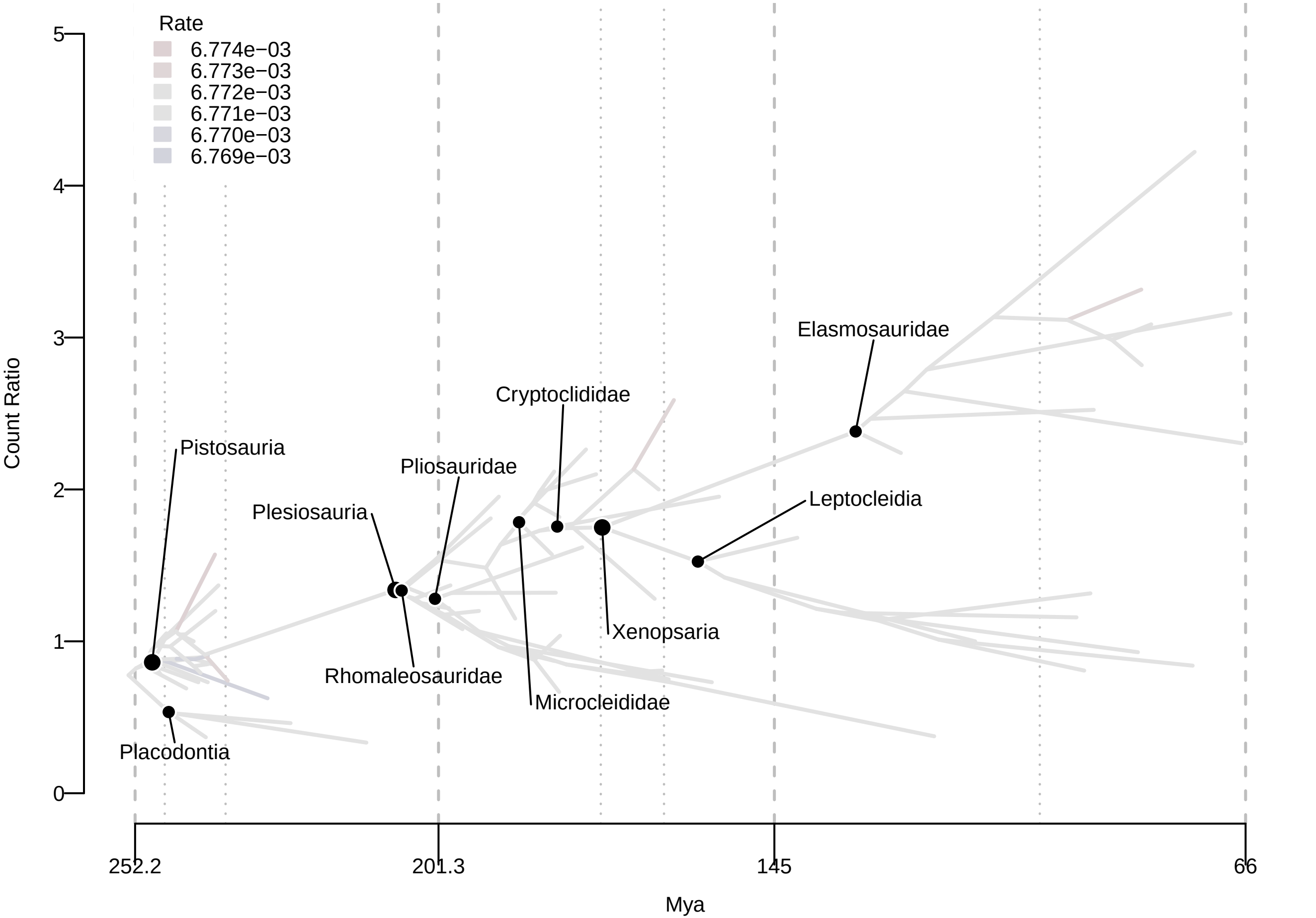

Rate
6.774e−03
6.773e−03
6.772e−03
6.771e−03
6.770e−03
6.769e−03
Count Ratio
0
1
2
3
4
5
Mya
252.2
201.3
145
66
Pistosauria
Placodontia
Plesiosauria
Rhomaleosauridae
Pliosauridae
Microcleididae
Cryptoclididae
Xenopsaria
Leptocleidia
Elasmosauridae

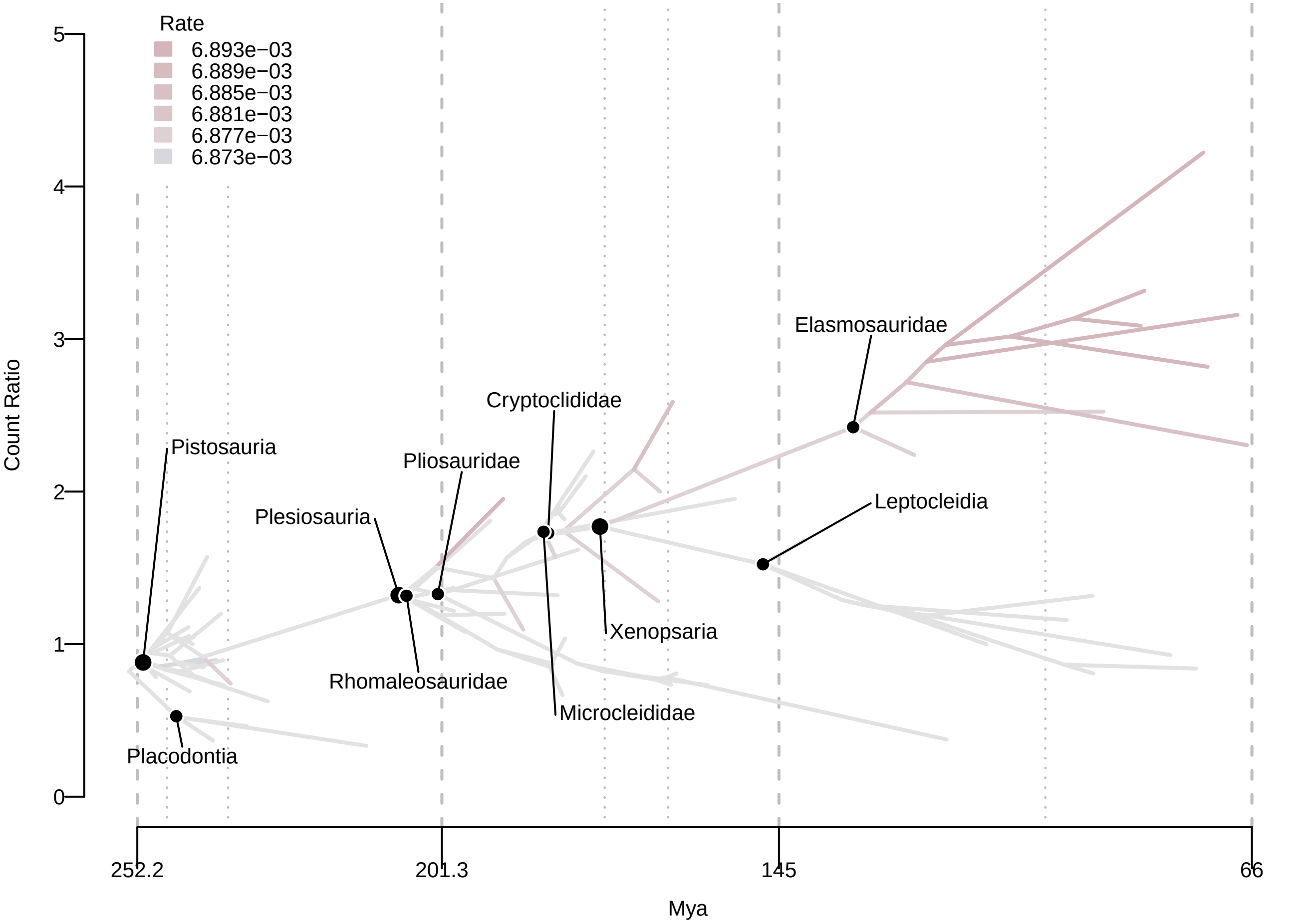

Rate
6.893e−03
6.889e−03
6.885e−03
6.881e−03
6.877e−03
6.873e−03
Count Ratio
0
1
2
3
4
5
Pistosauria
Placodontia
Plesiosauria
Rhomaleosauridae
Pliosauridae
Cryptoclididae
Microcleididae
Xenopsaria
Leptocleidia
Elasmosauridae
252.2
201.3
145
66
Mya

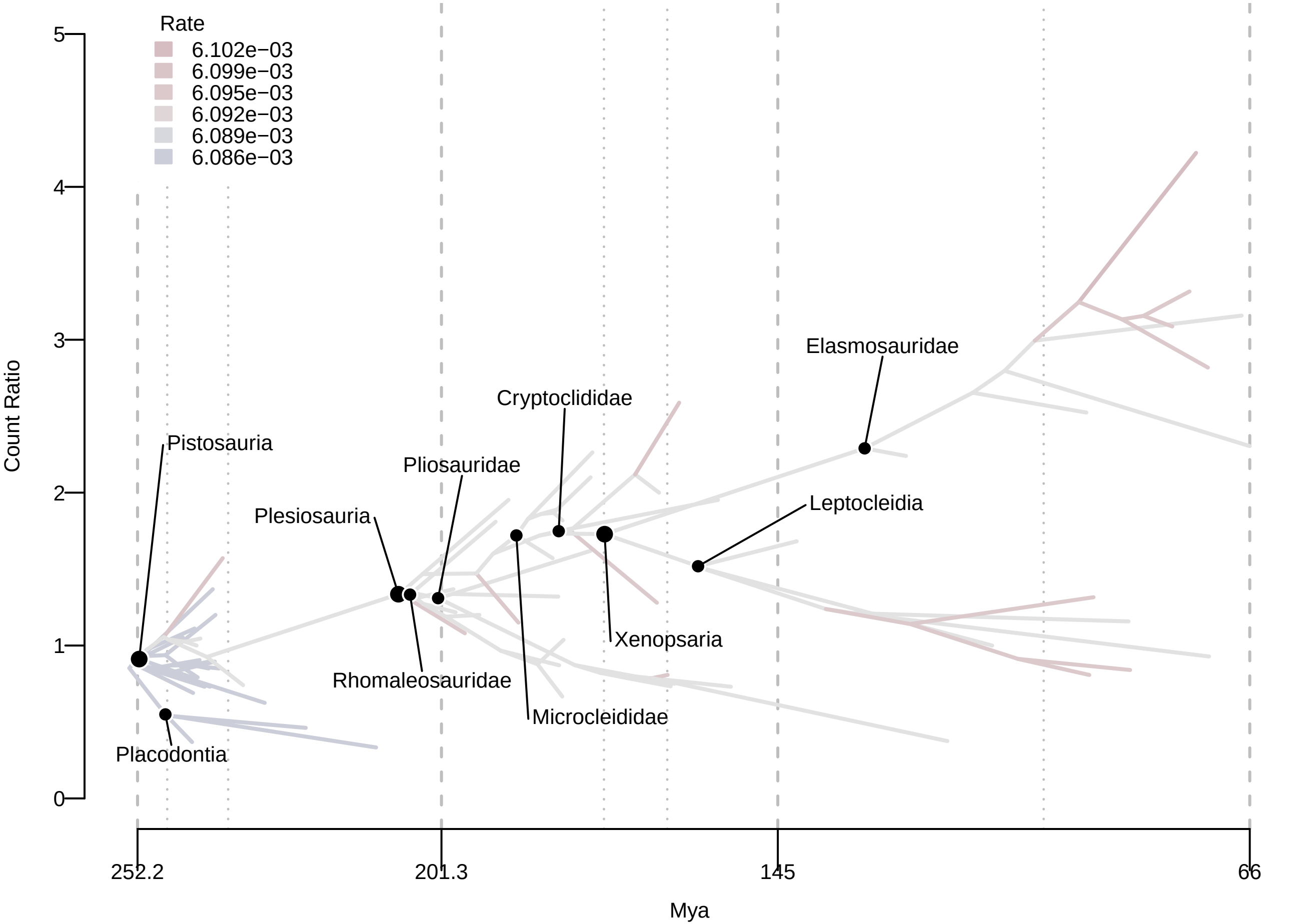

Rate
6.102e−03
6.099e−03
6.095e−03
6.092e−03
6.089e−03
6.086e−03
Count Ratio
0
1
2
3
4
5
Pistosauria
Placodontia
Plesiosauria
Pliosauridae
Rhomaleosauridae
Cryptoclididae
Microcleididae
Xenopsaria
Leptocleidia
Elasmosauridae
252.2
201.3
145
66
Mya

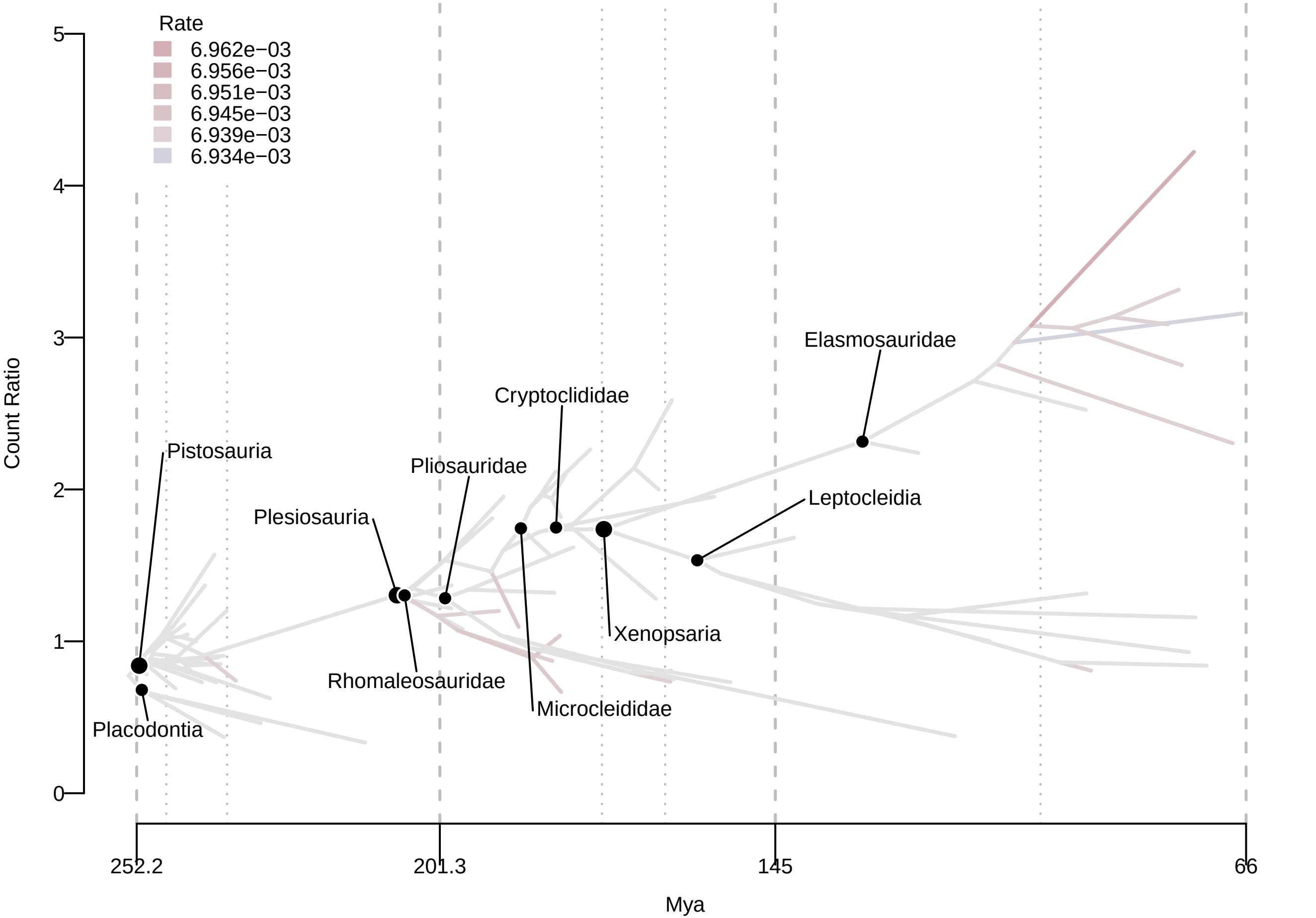

Rate
6.962e−03
6.956e−03
6.951e−03
6.945e−03
6.939e−03
6.934e−03
Count Ratio
5
4
3
2
1
0
Pistosauria
Placodontia
Plesiosauria
Rhomaleosauridae
Pliosauridae
Microcleididae
Cryptoclididae
Xenopsaria
Leptocleidia
Elasmosauridae
252.2
201.3
145
66
Mya

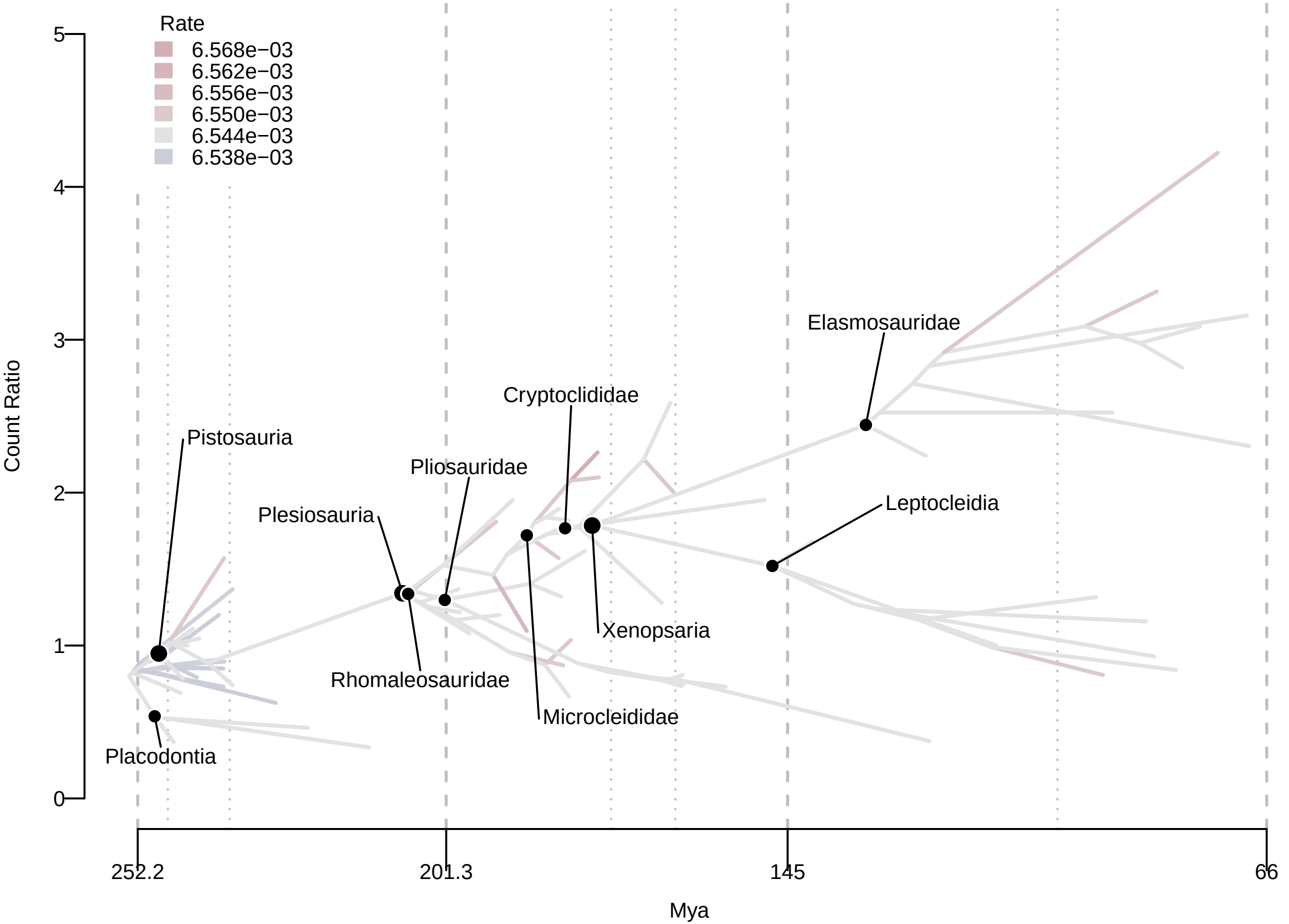

Rate
6.568e−03
6.562e−03
6.556e−03
6.550e−03
6.544e−03
6.538e−03
Count Ratio
0
1
2
3
4
5
Pistosauria
Pliosauridae
Plesiosauria
Cryptoclididae
Elasmosauridae
Leptocleidia
Xenopsaria
Rhomaleosauridae
Microcleididae
Placodontia
252.2
201.3
145
66
Mya

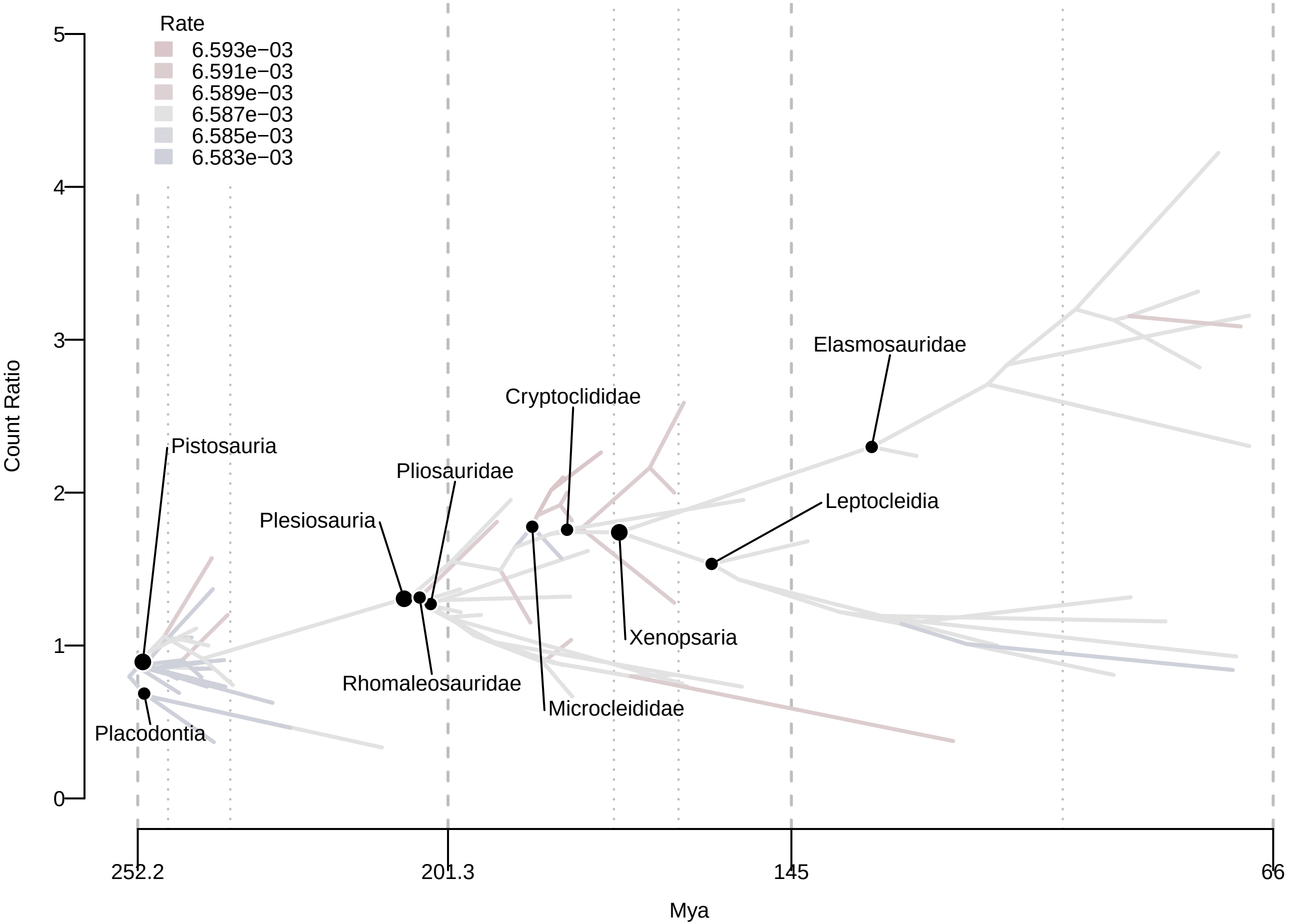

Rate
6.593e−03
6.591e−03
6.589e−03
6.587e−03
6.585e−03
6.583e−03
Count Ratio
0
1
2
3
4
5
Pistosauria
Placodontia
Plesiosauria
Pliosauridae
Rhomaleosauridae
Microcleididae
Cryptoclididae
Xenopsaria
Leptocleidia
Elasmosauridae
252.2
201.3
145
66
Mya

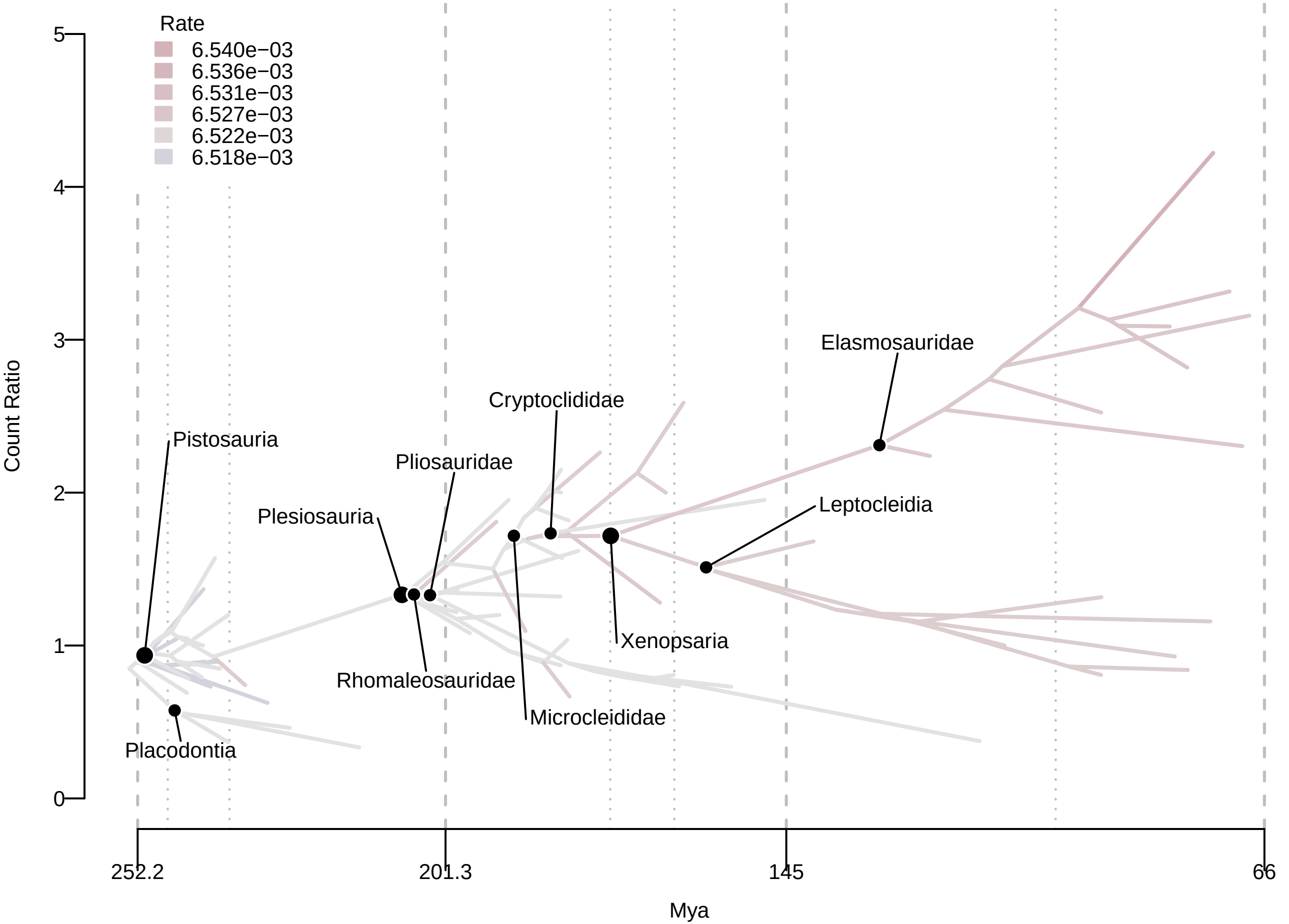

Rate
6.540e−03
6.536e−03
6.531e−03
6.527e−03
6.522e−03
6.518e−03
Count Ratio
0
1
2
3
4
5
Pistosauria
Placodontia
Plesiosauria
Rhomaleosauridae
Pliosauridae
Cryptoclididae
Microcleididae
Xenopsaria
Leptocleidia
Elasmosauridae
252.2
201.3
145
66
Mya

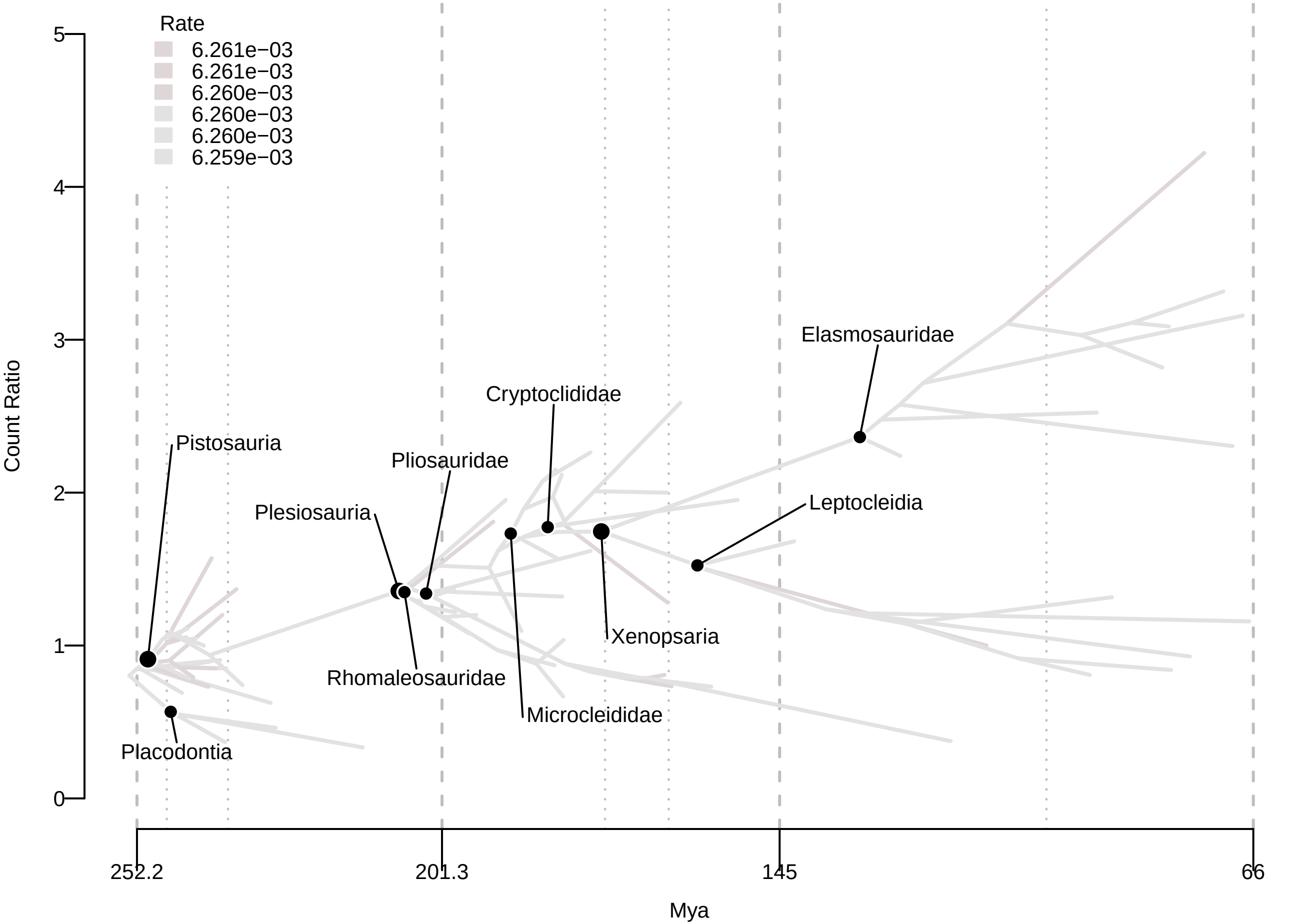

Rate
6.261e−03
6.261e−03
6.260e−03
6.260e−03
6.260e−03
6.259e−03
Count Ratio
0
1
2
3
4
5
Mya
252.2
201.3
145
66
Pistosauria
Placodontia
Plesiosauria
Pliosauridae
Rhomaleosauridae
Cryptoclididae
Microcleididae
Xenopsaria
Leptocleidia
Elasmosauridae

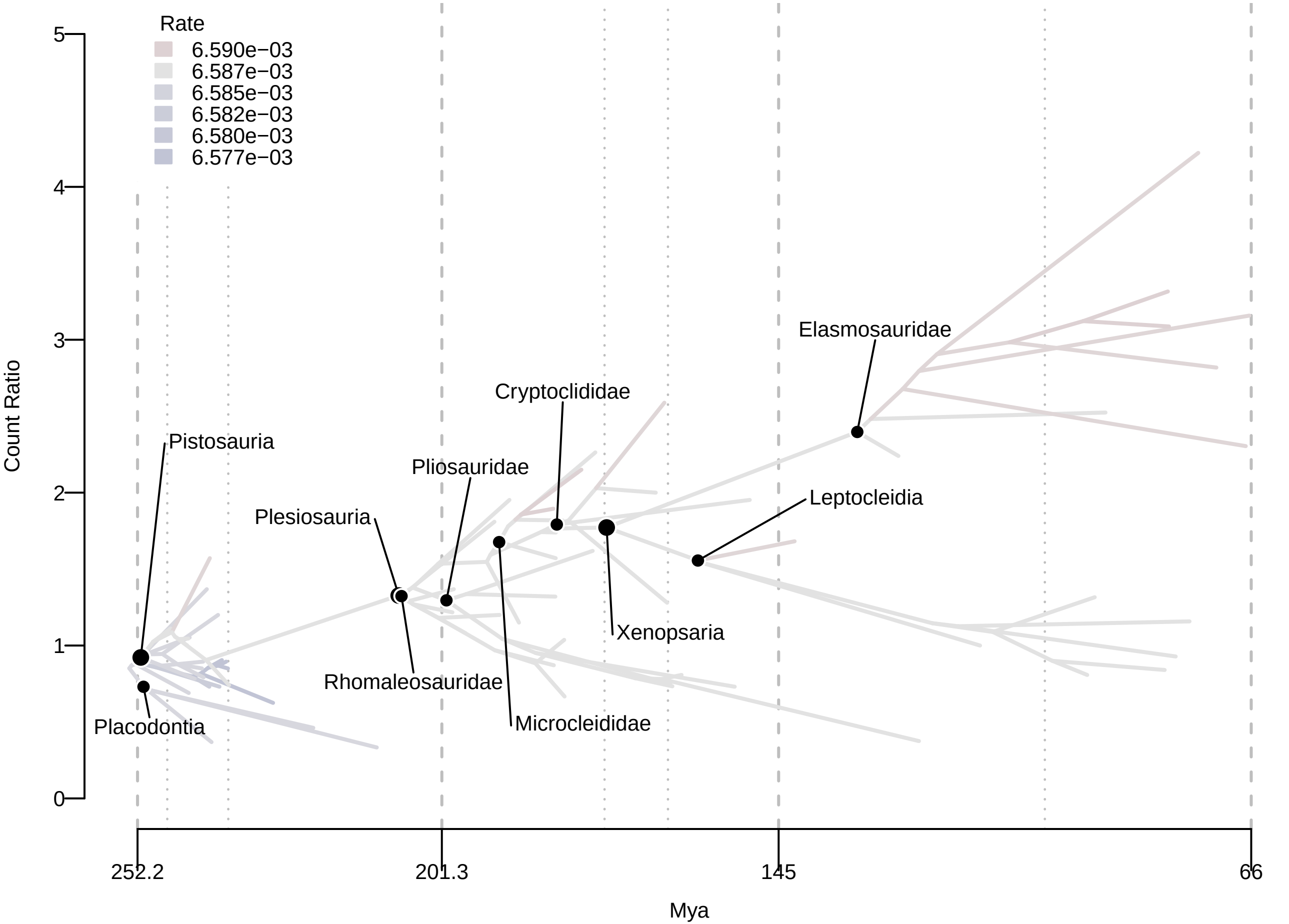

Rate
6.590e−03
6.587e−03
6.585e−03
6.582e−03
6.580e−03
6.577e−03
Count Ratio
5
4
3
2
1
0
Pistosauria
Placodontia
Plesiosauria
Rhomaleosauridae
Pliosauridae
Microcleididae
Cryptoclididae
Xenopsaria
Leptocleidia
Elasmosauridae
252.2
201.3
145
66
Mya

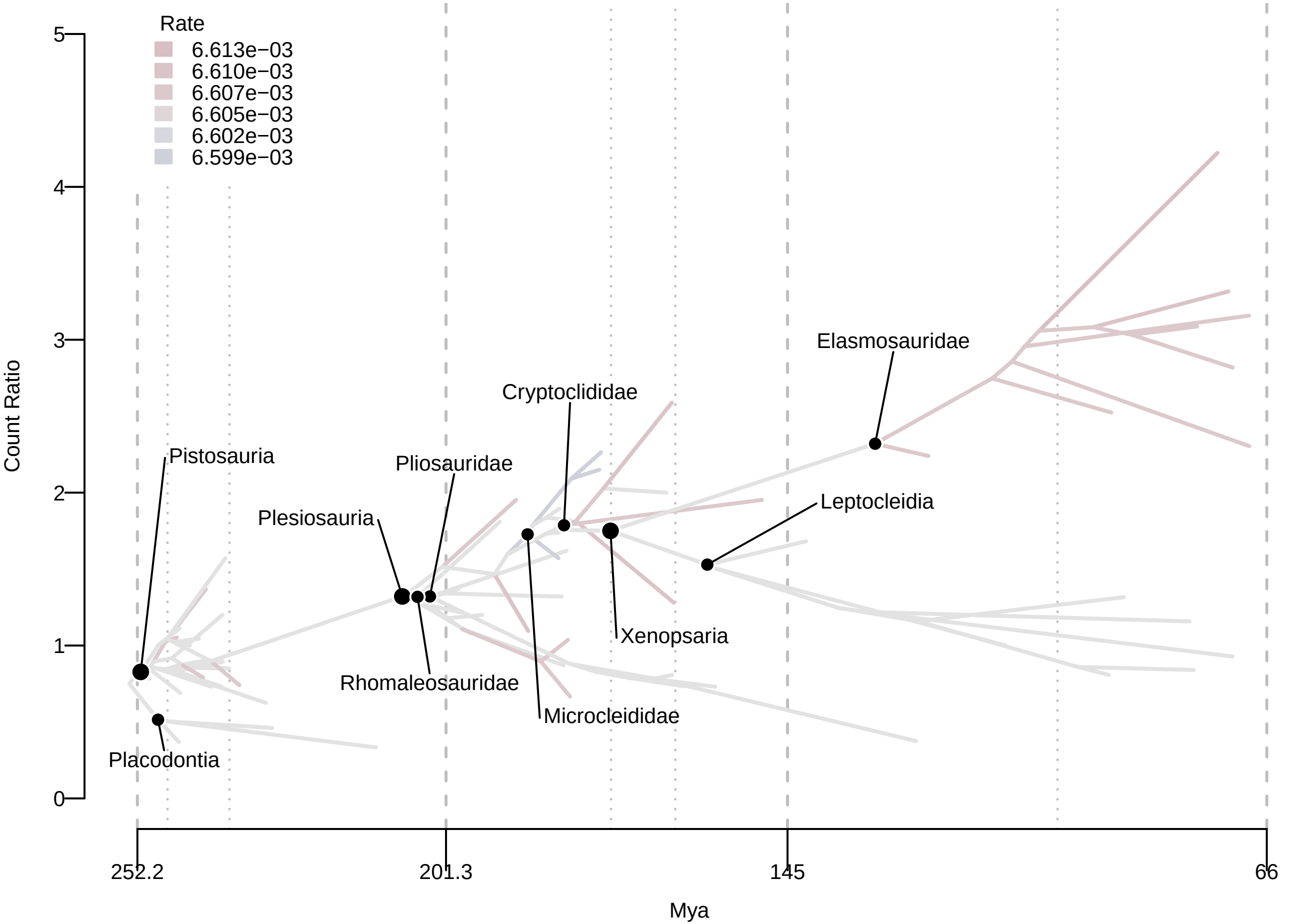

Rate
6.613e−03
6.610e−03
6.607e−03
6.605e−03
6.602e−03
6.599e−03
Count Ratio
0
1
2
3
4
5
Pistosauria
Placodontia
Plesiosauria
Pliosauridae
Rhomaleosauridae
Cryptoclididae
Microcleididae
Xenopsaria
Leptocleidia
Elasmosauridae
252.2
201.3
145
66
Mya

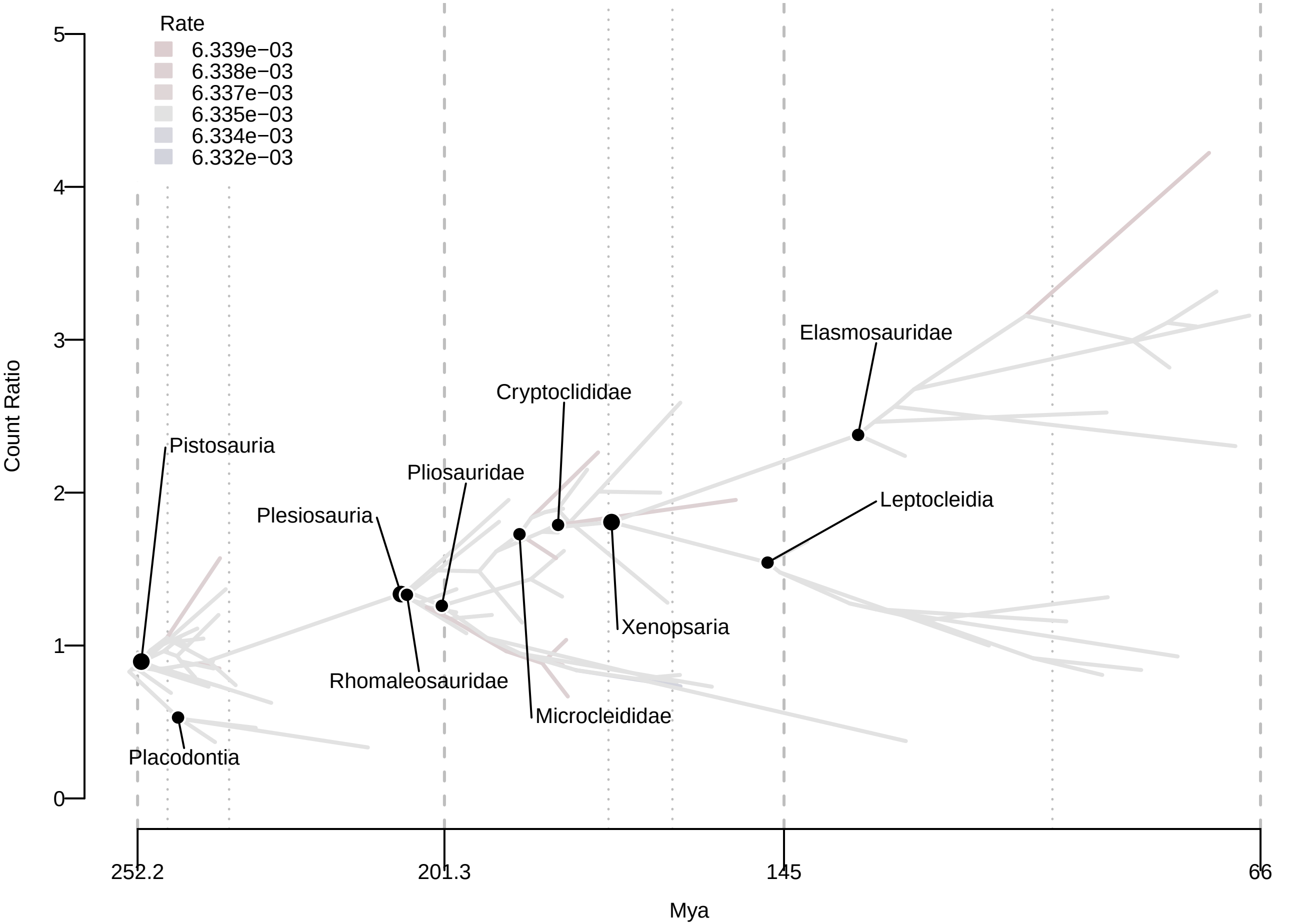

Rate
6.339e−03
6.338e−03
6.337e−03
6.335e−03
6.334e−03
6.332e−03
Count Ratio
5
4
3
2
1
0
Pistosauria
Placodontia
Plesiosauria
Rhomaleosauridae
Pliosauridae
Microcleididae
Cryptoclididae
Xenopsaria
Leptocleidia
Elasmosauridae
252.2
201.3
145
66
Mya

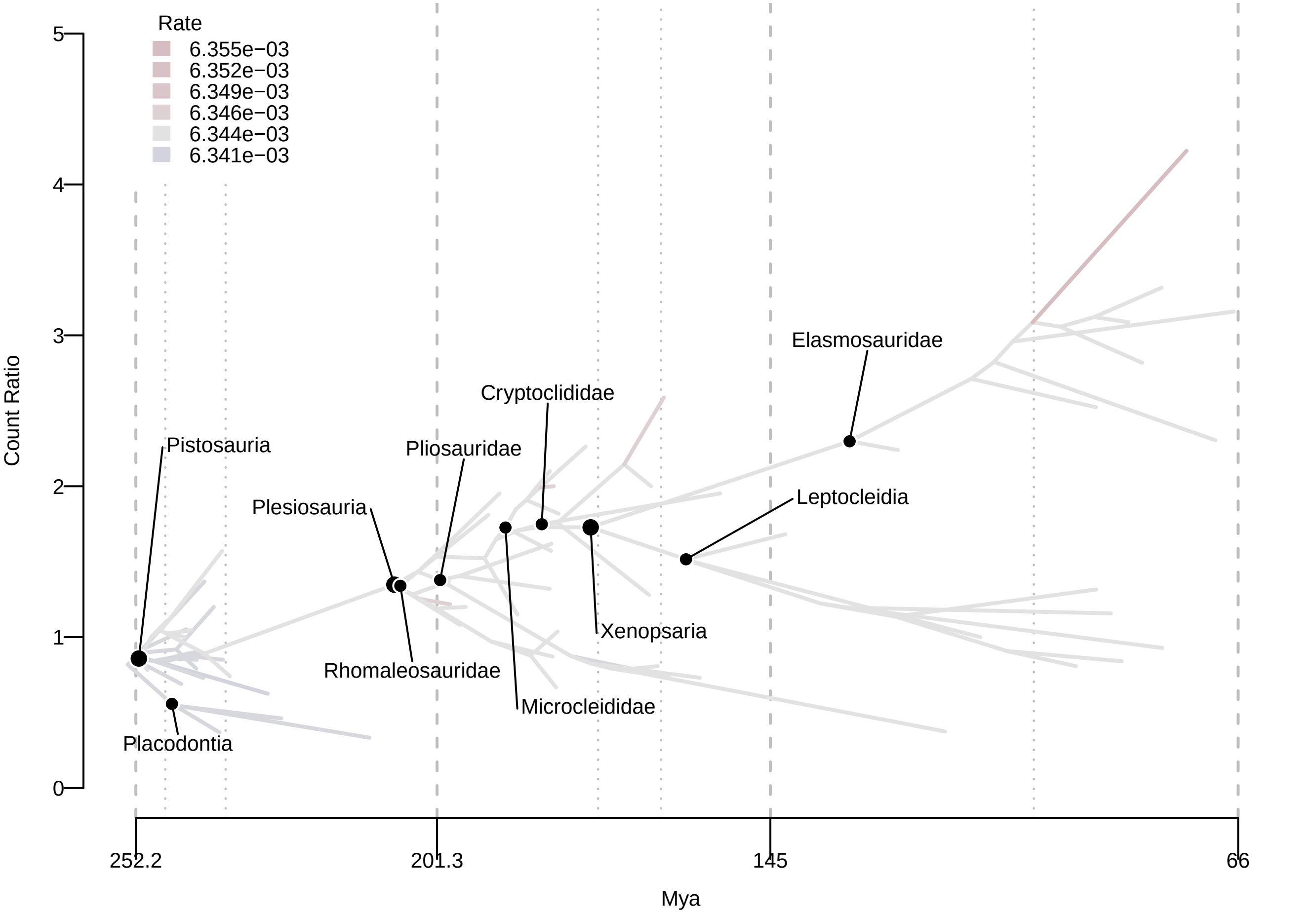

Rate
6.355e−03
6.352e−03
6.349e−03
6.346e−03
6.344e−03
6.341e−03
Count Ratio
0
1
2
3
4
5
Pistosauria
Placodontia
Plesiosauria
Rhomaleosauridae
Pliosauridae
Cryptoclididae
Microcleididae
Xenopsaria
Leptocleidia
Elasmosauridae
252.2
201.3
145
66
Mya

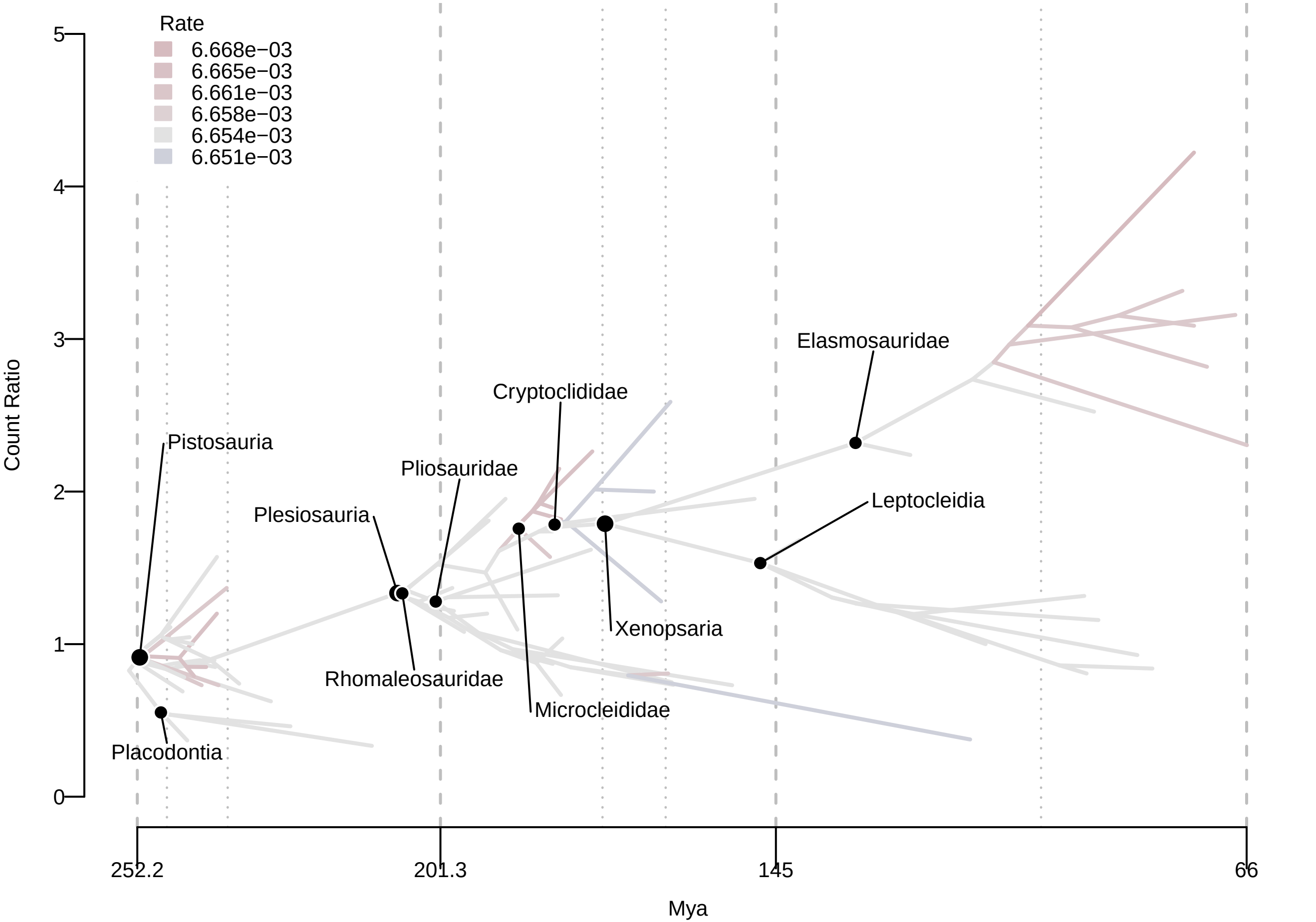

Rate
6.668e−03
6.665e−03
6.661e−03
6.658e−03
6.654e−03
6.651e−03
Count Ratio
5
4
3
2
1
0
Pistosauria
Placodontia
Plesiosauria
Rhomaleosauridae
Pliosauridae
Cryptoclididae
Microcleididae
Xenopsaria
Elasmosauridae
Leptocleidia
252.2
201.3
145
66
Mya

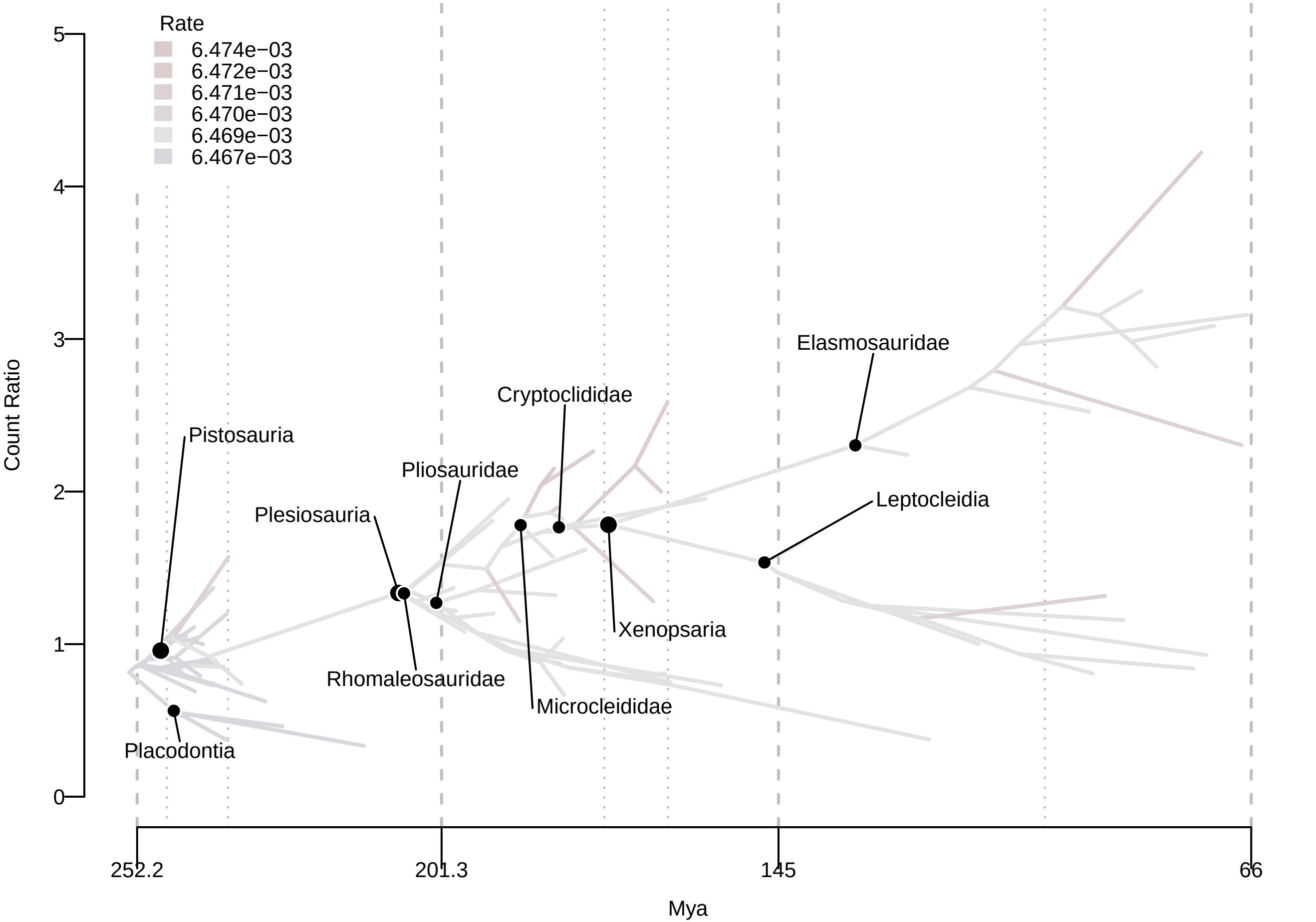

Rate
6.474e−03
6.472e−03
6.471e−03
6.470e−03
6.469e−03
6.467e−03
Count Ratio
0
1
2
3
4
5
Pistosauria
Placodontia
Plesiosauria
Rhomaleosauridae
Pliosauridae
Cryptoclididae
Microcleididae
Xenopsaria
Elasmosauridae
Leptocleidia
252.2
201.3
145
66
Mya

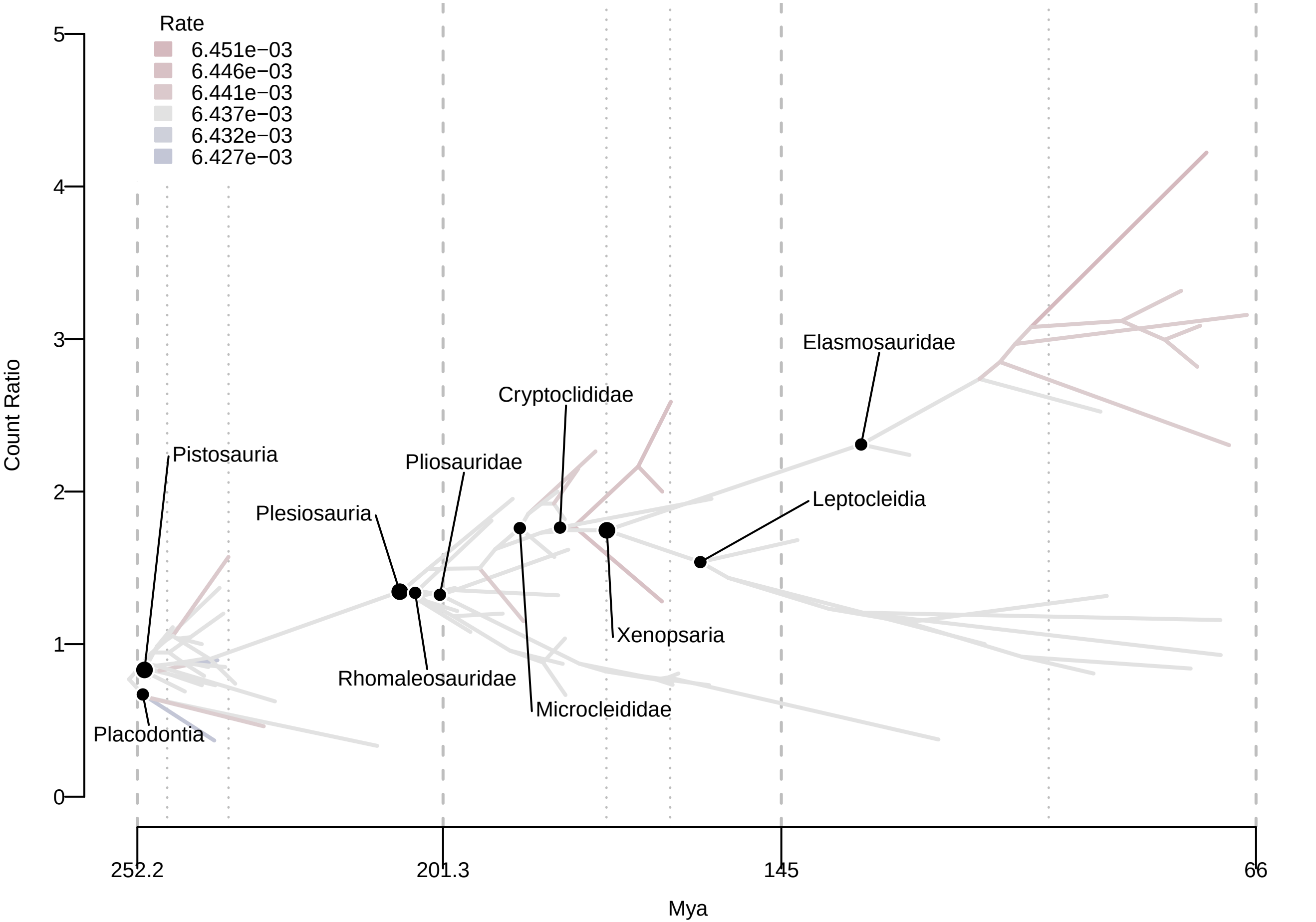

Rate
6.451e−03
6.446e−03
6.441e−03
6.437e−03
6.432e−03
6.427e−03
Count Ratio
0
1
2
3
4
5
Pistosauria
Placodontia
Plesiosauria
Rhomaleosauridae
Pliosauridae
Microcleididae
Cryptoclididae
Xenopsaria
Leptocleidia
Elasmosauridae
252.2
201.3
145
66
Mya

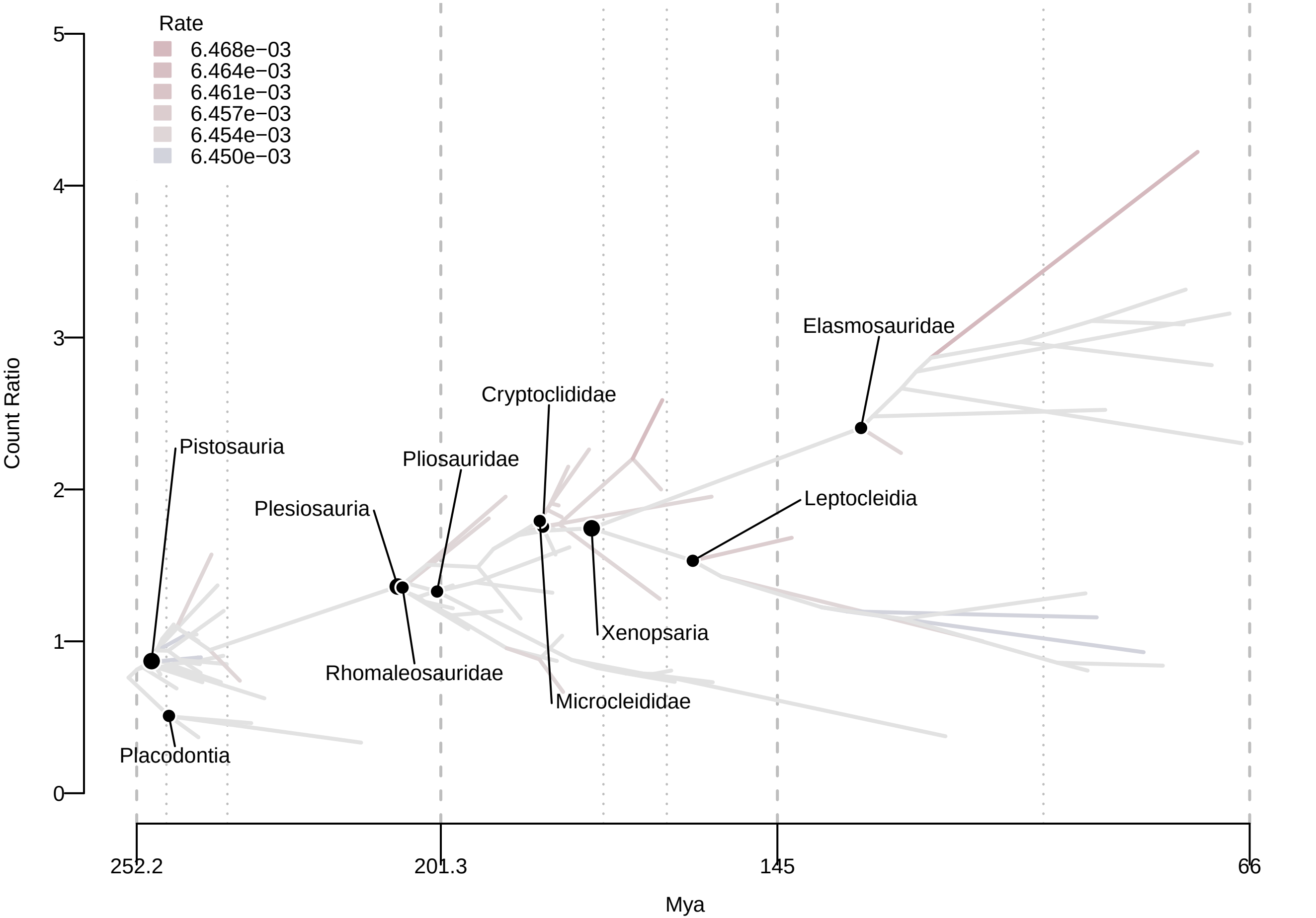

Rate
6.468e−03
6.464e−03
6.461e−03
6.457e−03
6.454e−03
6.450e−03
Count Ratio
0
1
2
3
4
5
Pistosauria
Plesiosauria
Pliosauridae
Cryptoclididae
Elasmosauridae
Leptocleidia
Xenopsaria
Rhomaleosauridae
Microcleididae
Placodontia
252.2
201.3
145
66
Mya
