# Supplementary material for: Developmental mechanisms of macroevolutionary change in the tetrapod axis: A case study of Sauropterygia
Source: Evolution. 2017 Mar 21;71(5):1164–77. doi: 10.1111/evo.13217 (PMC5485078; doi:10.1111/evo.13217)
Supplement: Supplementary file 10 — Figure S10. [file EVO-71-1164-s010.pdf]

Counterpart to figure 3

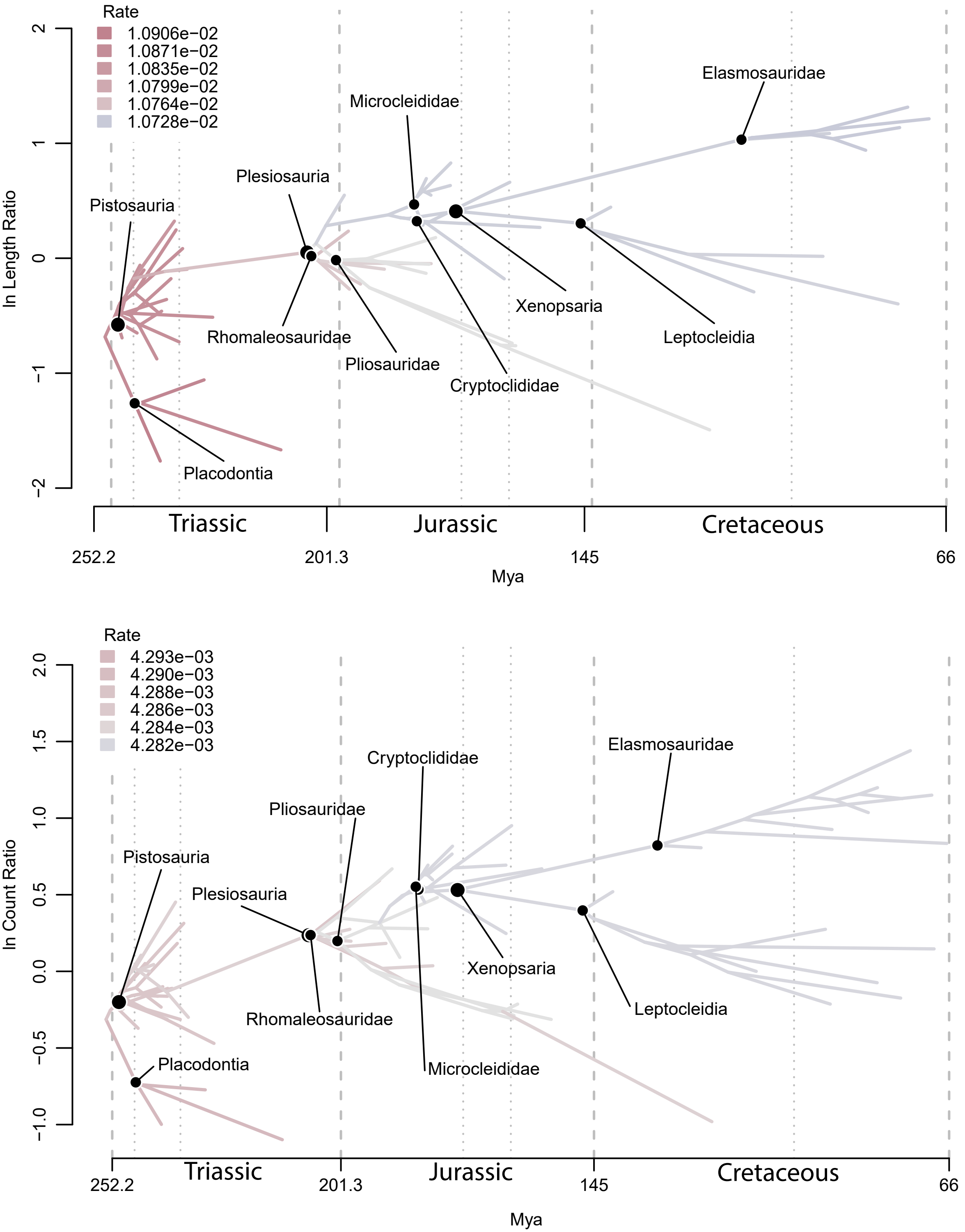

Counterpart to figure 4

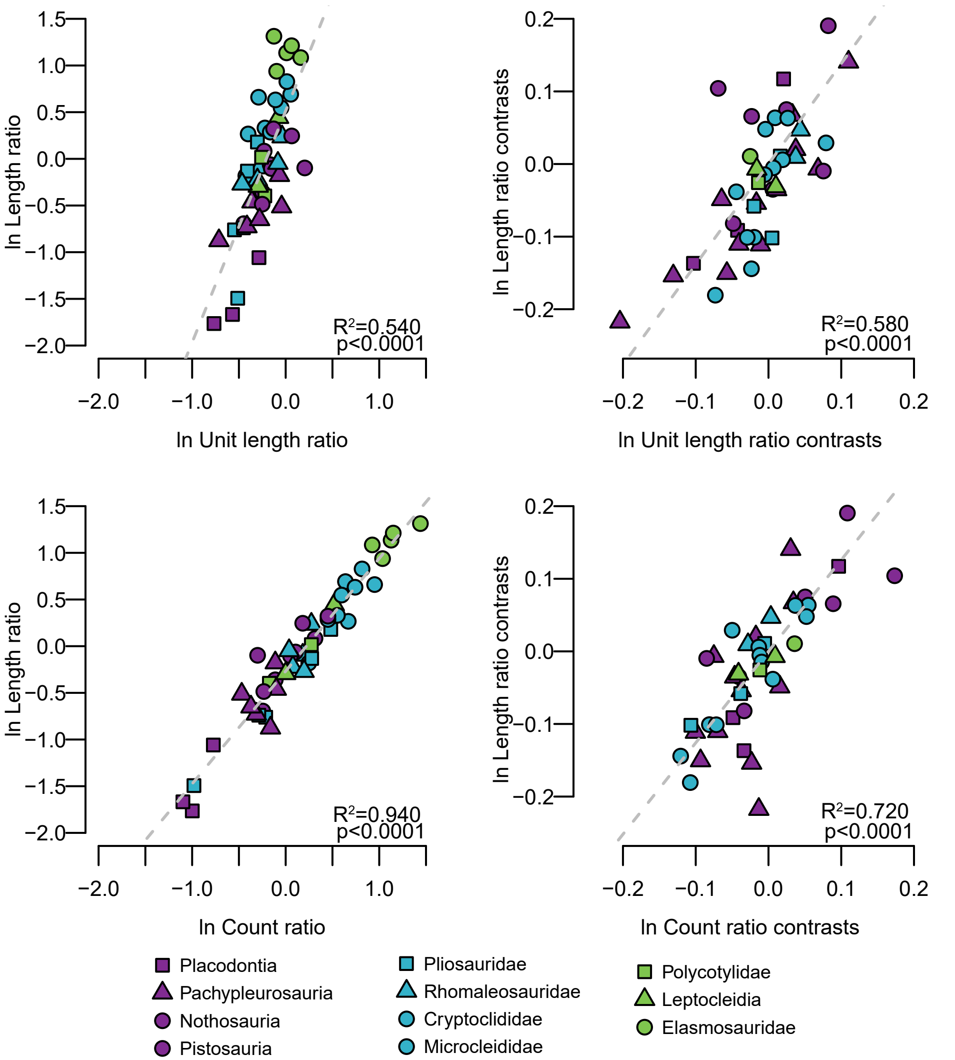

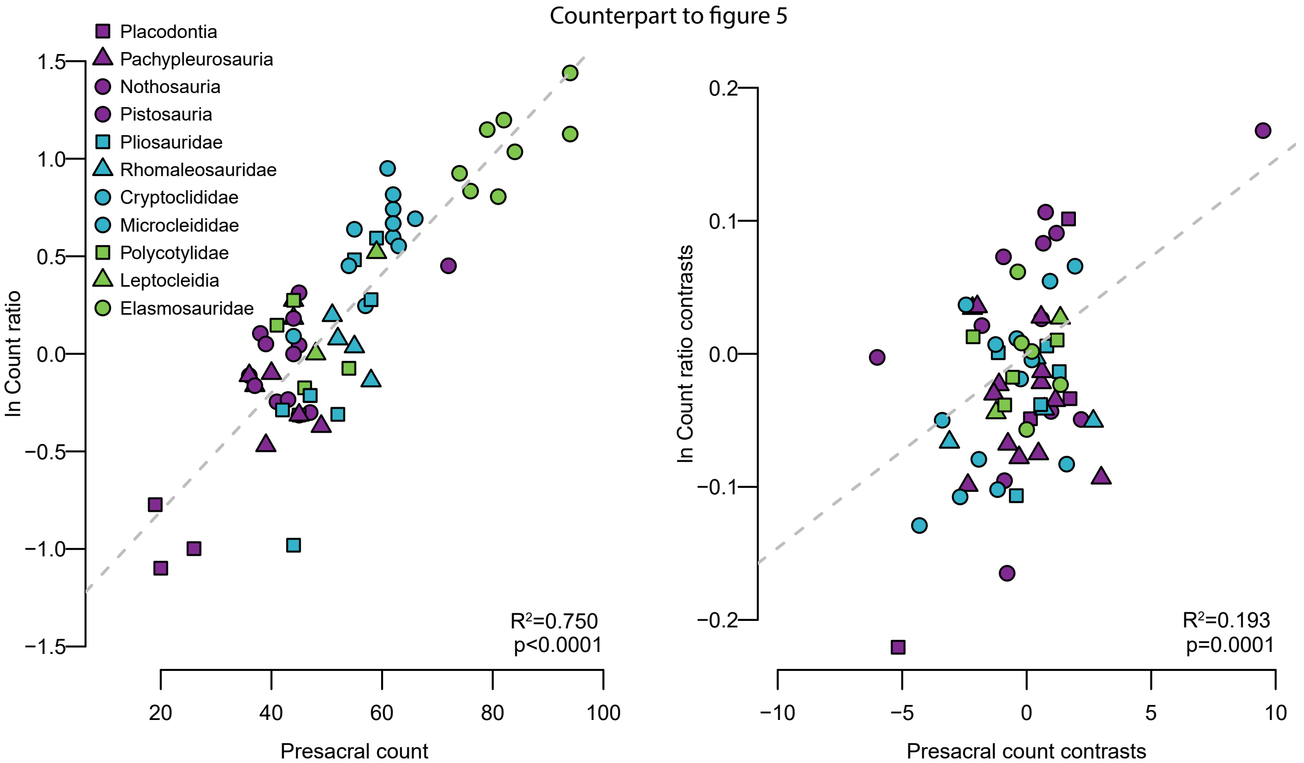
Counterpart to figure 5
Placodontia
Pachypleurosauria
Nothosauria
Pistosauria
Pliosauridae
Rhomaleosauridae
Cryptoclididae
Microcleididae
Polycotylidae
Leptocleidia
Elasmosauridae
ln Count ratio
Presacral count
R²=0.750
p<0.0001
ln Count ratio contrasts
Presacral count contrasts
R²=0.193
p=0.0001
